# Supplementary figures and images for: Comprehensive patient-level classification and quantification of driver events in TCGA PanCanAtlas cohorts (part 2 of 6)
Source: PLoS Genet. 2022 Jan 14;18(1):e1009996. doi: 10.1371/journal.pgen.1009996 (PMC8759692; doi:10.1371/journal.pgen.1009996)

# SARC

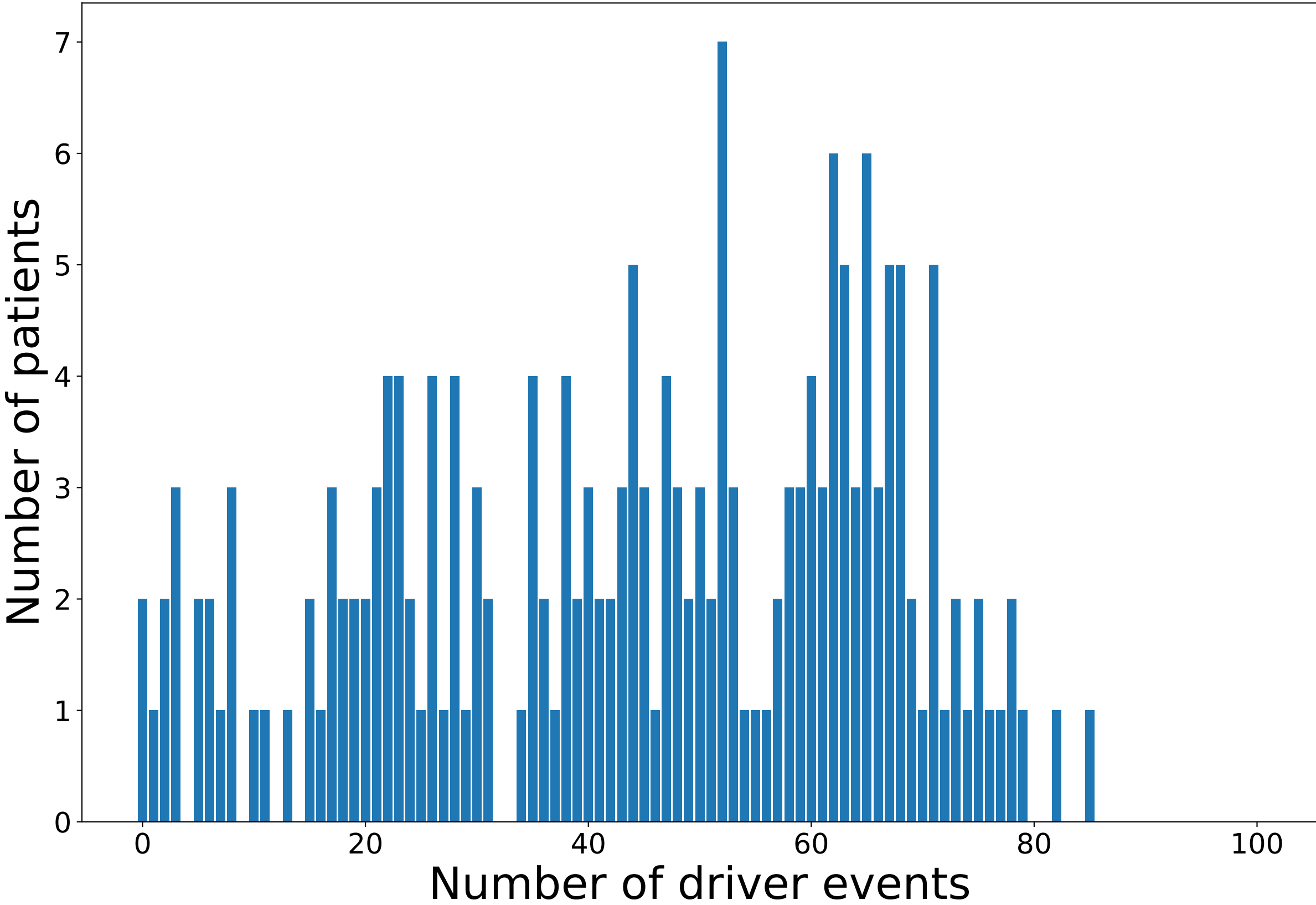

Supplement: S2 Files — (ZIP) [file pgen.1009996.s002.zip › PANCAN/patient distributions/2021_11_23_14_43_SARC.pdf]

# UCS

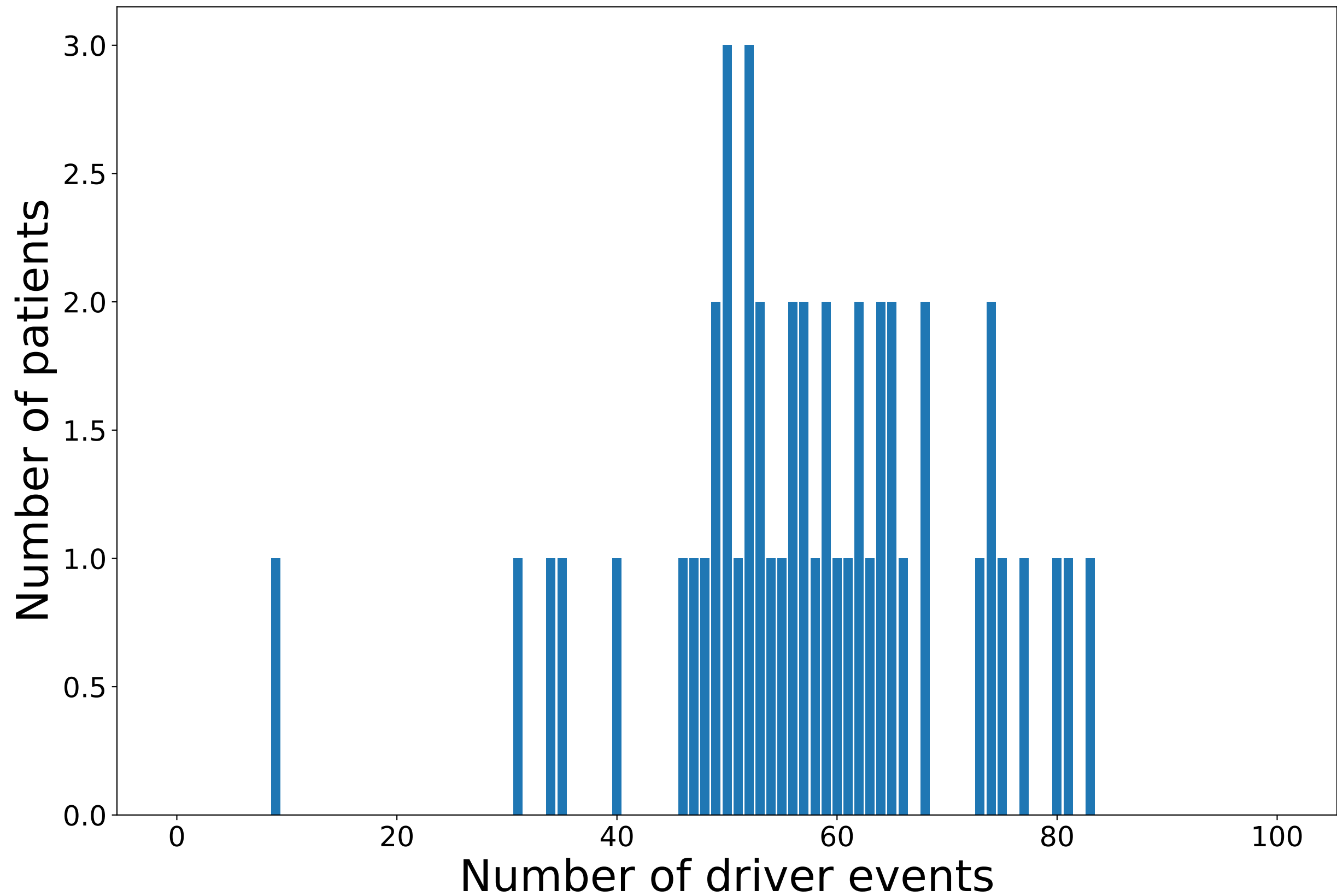

Supplement: S2 Files — (ZIP) [file pgen.1009996.s002.zip › PANCAN/patient distributions/2021_11_23_14_43_UCS.pdf]

# STAD\_FEMALE

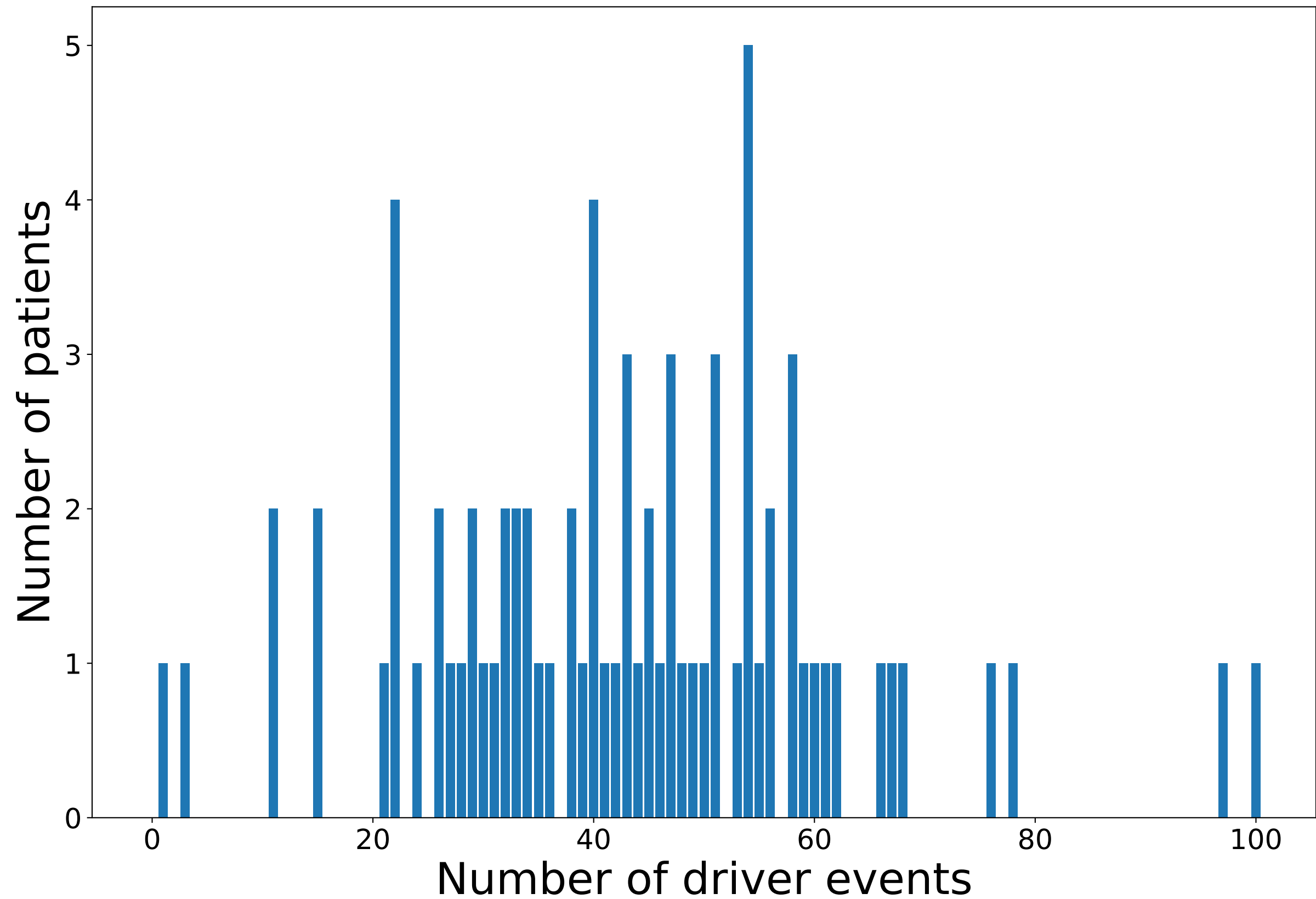

Supplement: S2 Files — (ZIP) [file pgen.1009996.s002.zip › PANCAN/patient distributions/2021_11_23_14_43_STAD_FEMALE.pdf]

# LUAD

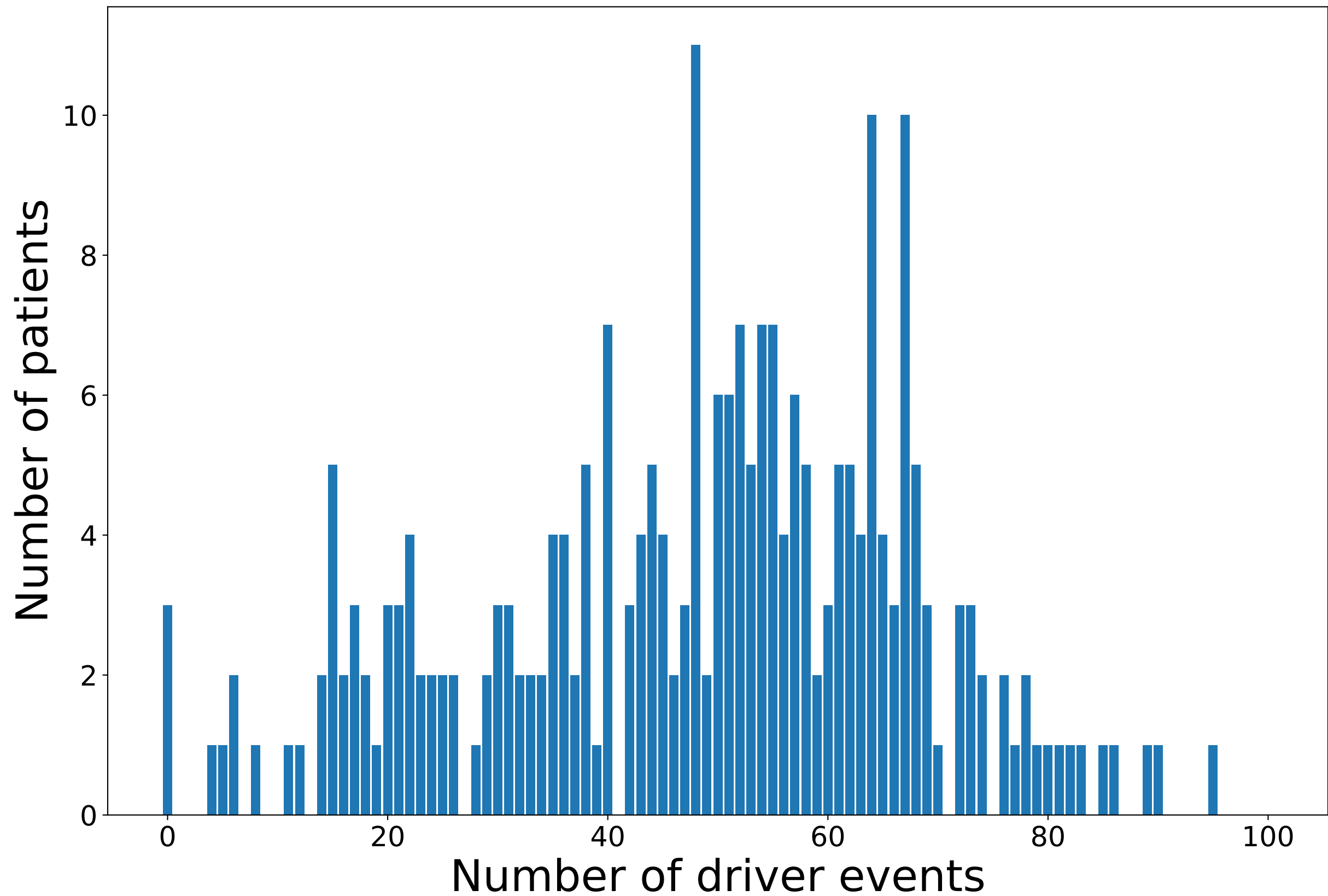

Supplement: S2 Files — (ZIP) [file pgen.1009996.s002.zip › PANCAN/patient distributions/2021_11_23_14_43_LUAD.pdf]

# ACC\_FEMALE

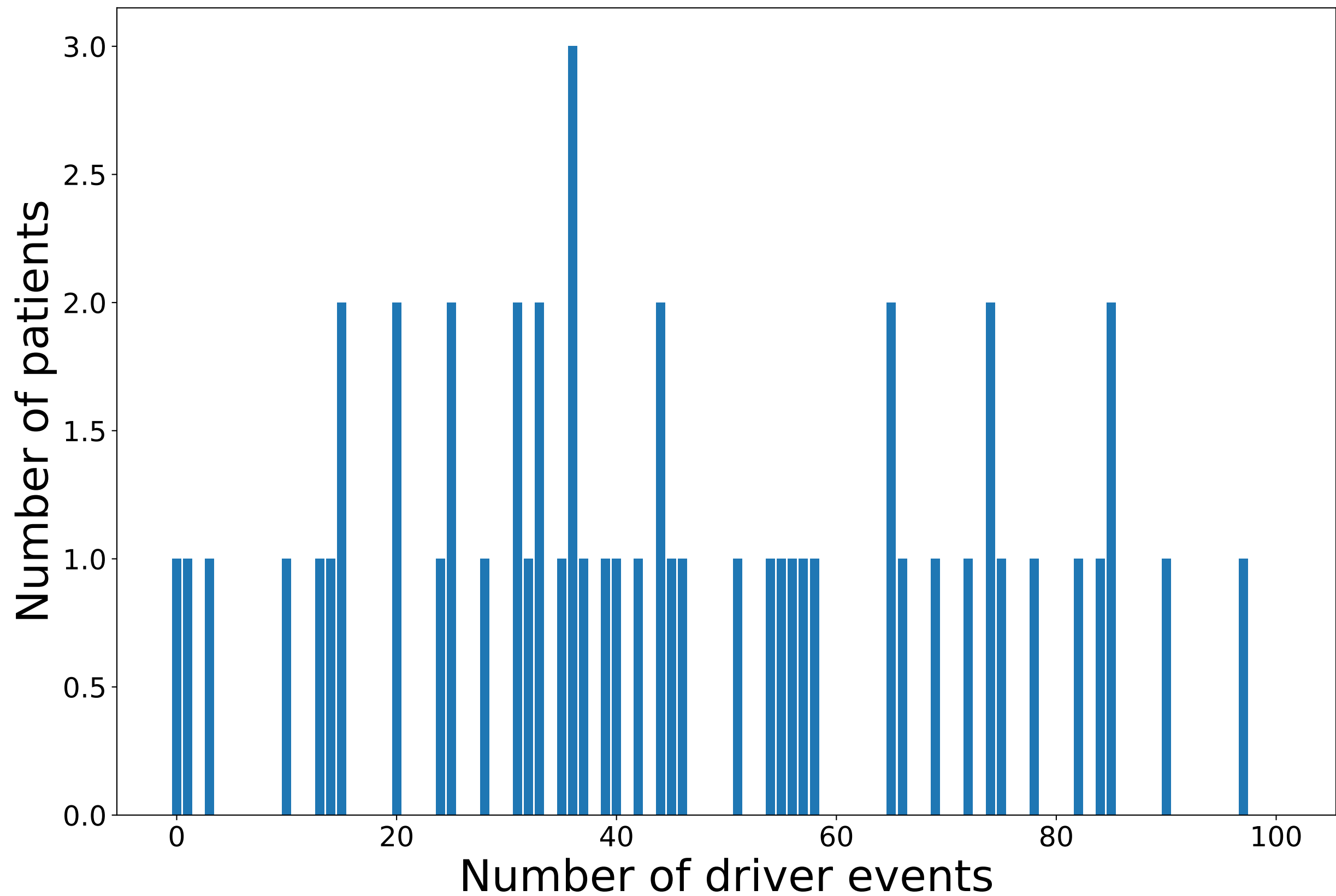

Supplement: S2 Files — (ZIP) [file pgen.1009996.s002.zip › PANCAN/patient distributions/2021_11_23_14_43_ACC_FEMALE.pdf]

# KIRC\_MALE

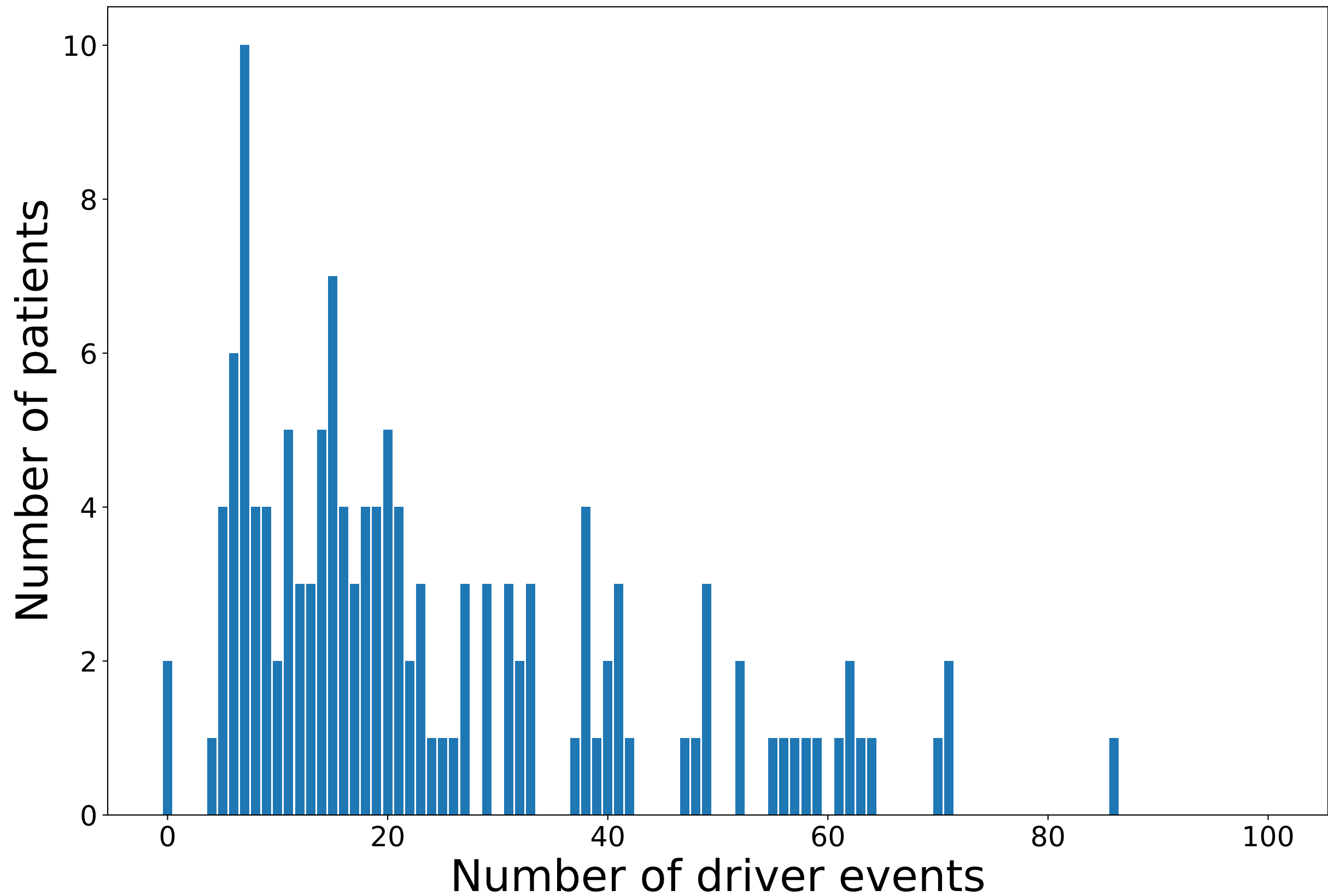

Supplement: S2 Files — (ZIP) [file pgen.1009996.s002.zip › PANCAN/patient distributions/2021_11_23_14_43_KIRC_MALE.pdf]

Driver event distribution by cancer stage BRCA

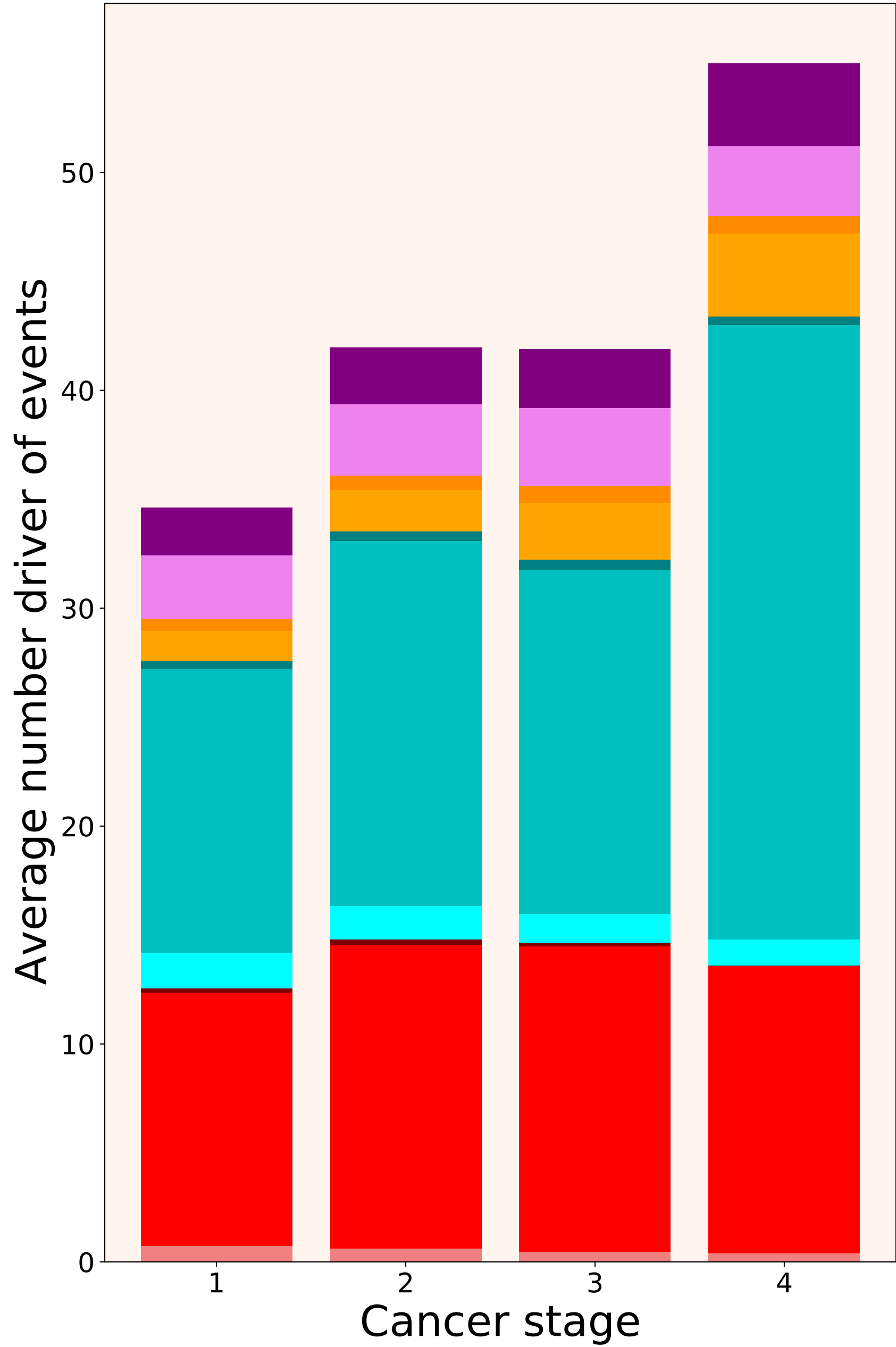

Supplement: S2 Files — (ZIP) [file pgen.1009996.s002.zip › PANCAN/cumulative histograms/Distribution_stages_cohorts/2021_11_23_14_43_distribution_stages_BRCA.pdf]

Driver event distribution by cancer stage in females DLBC

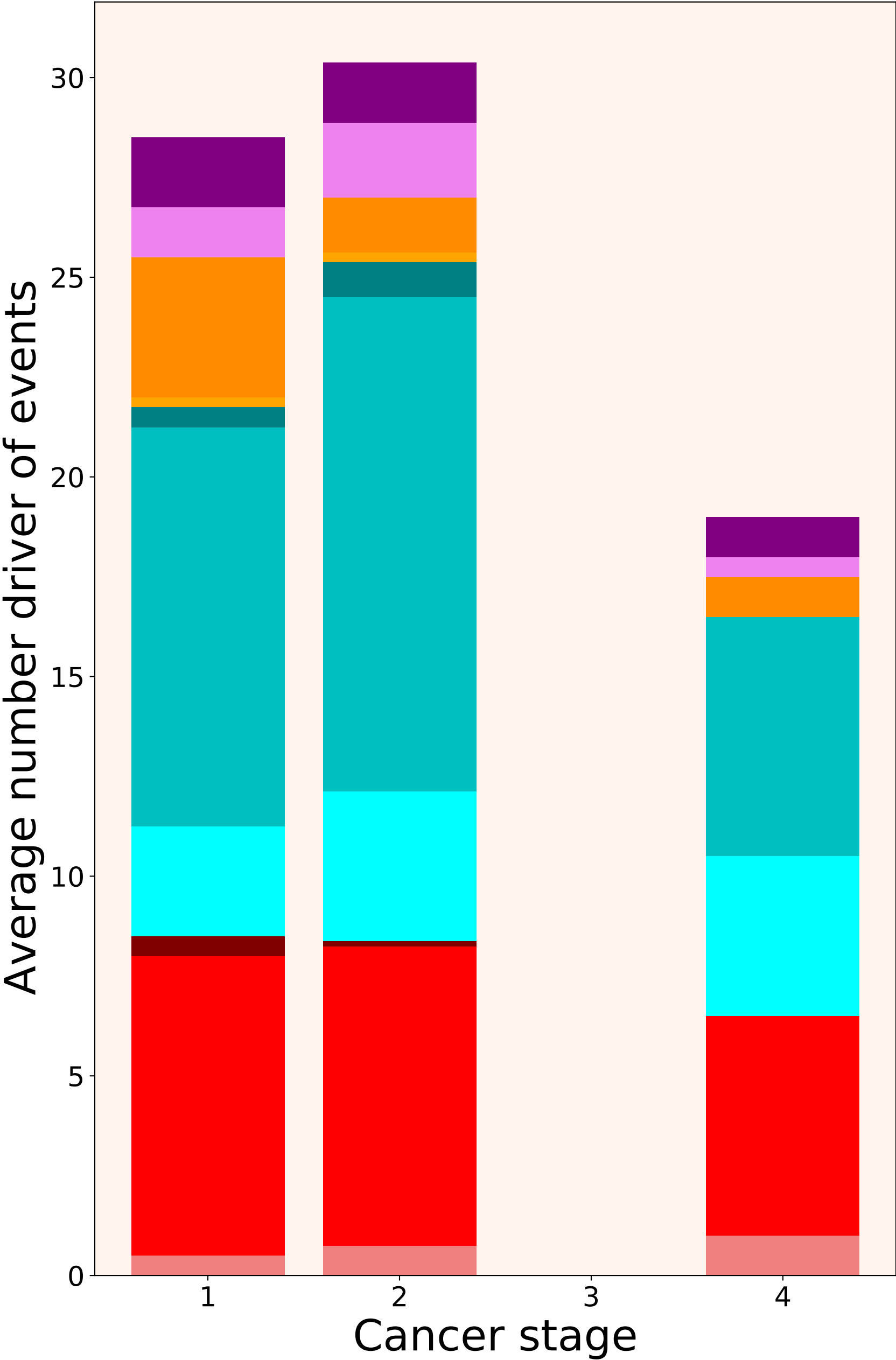

Supplement: S2 Files — (ZIP) [file pgen.1009996.s002.zip › PANCAN/cumulative histograms/Distribution_stages_cohorts/2021_11_23_14_43_distribution_stages_females_DLBC.pdf]

Driver event distribution by cancer stage in females MESO

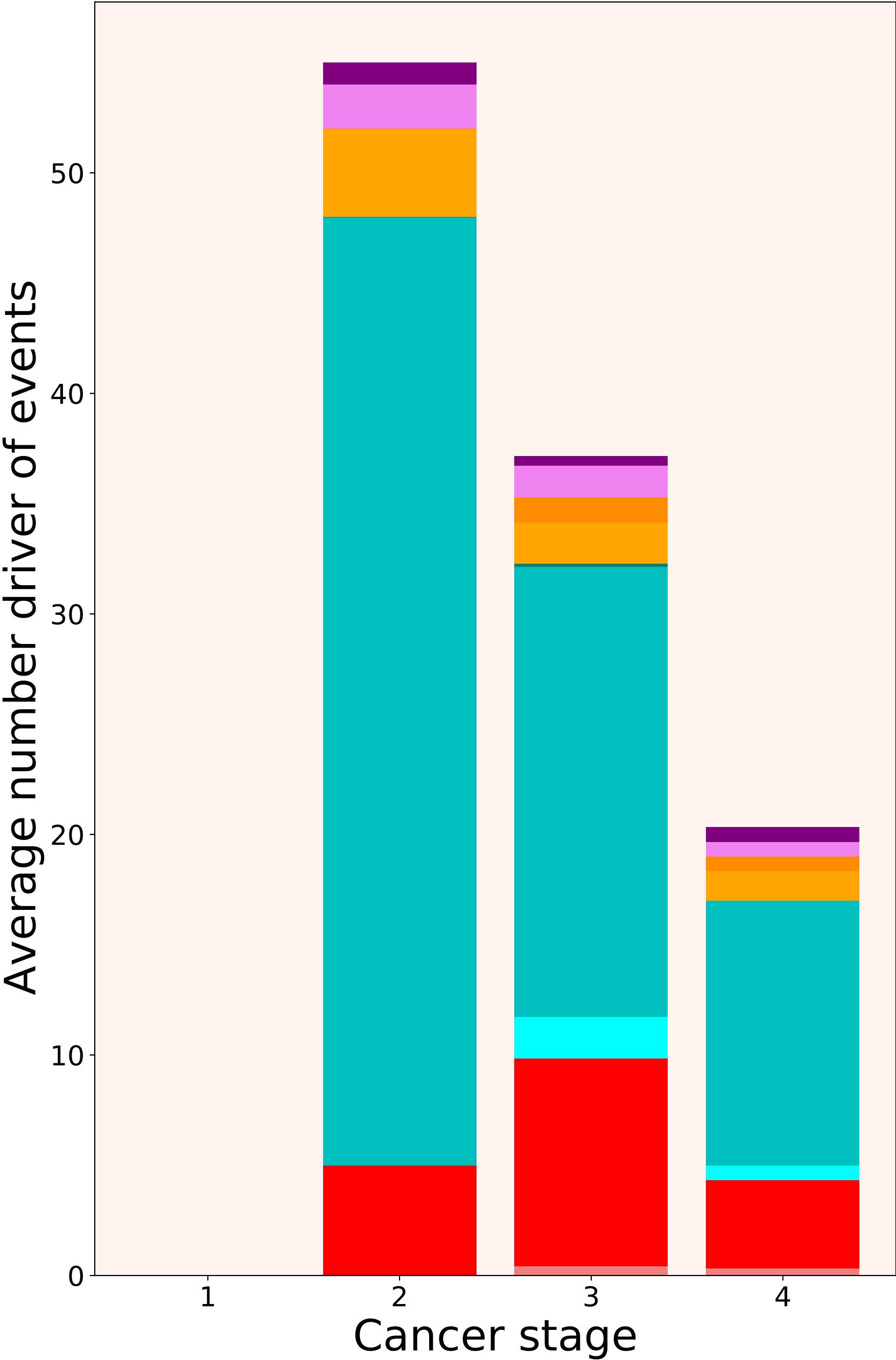

Supplement: S2 Files — (ZIP) [file pgen.1009996.s002.zip › PANCAN/cumulative histograms/Distribution_stages_cohorts/2021_11_23_14_43_distribution_stages_females_MESO.pdf]

Driver event distribution by cancer stage in females SKCM

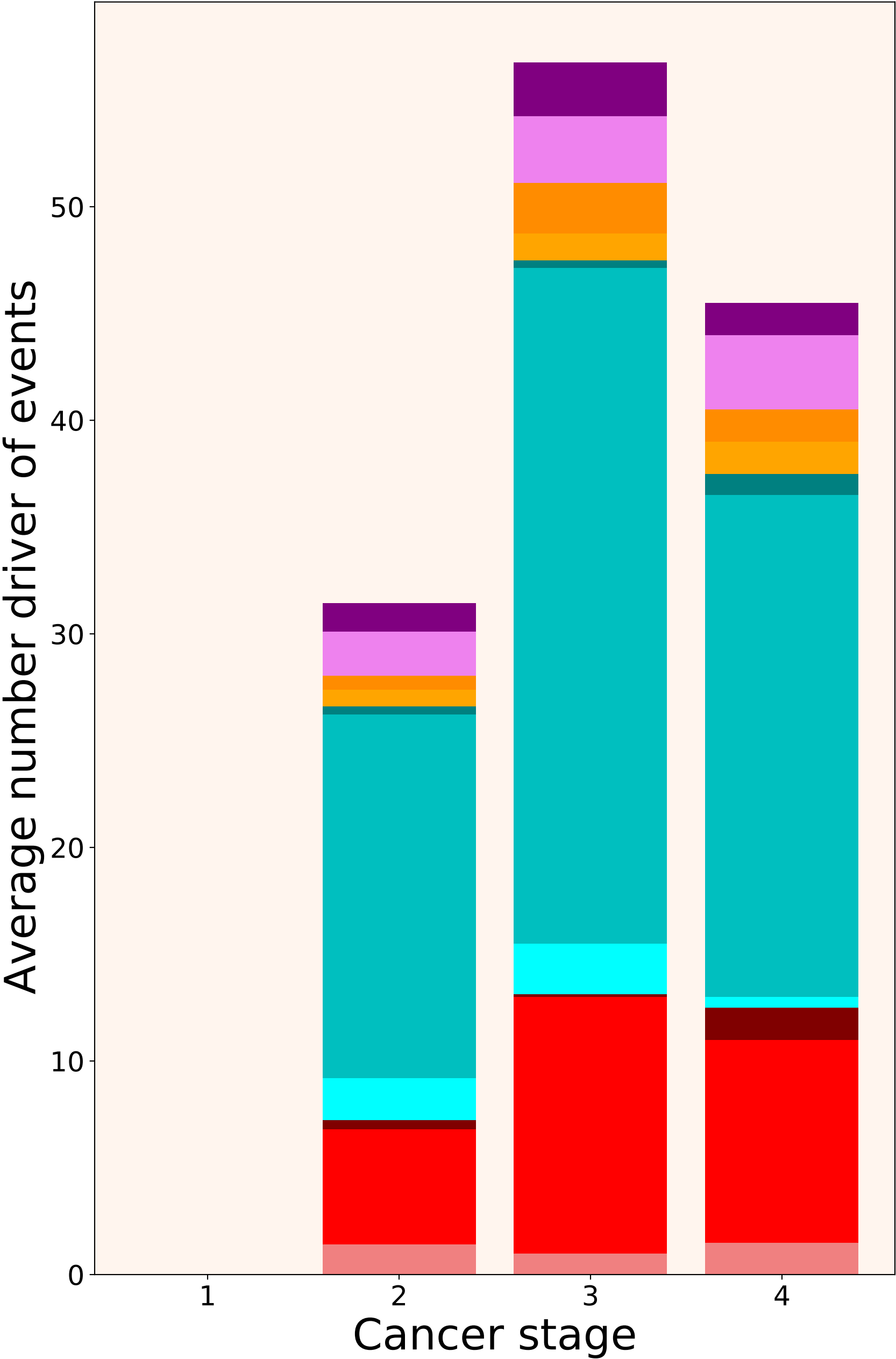

Supplement: S2 Files — (ZIP) [file pgen.1009996.s002.zip › PANCAN/cumulative histograms/Distribution_stages_cohorts/2021_11_23_14_43_distribution_stages_females_SKCM.pdf]

Driver event distribution by cancer stage MESO

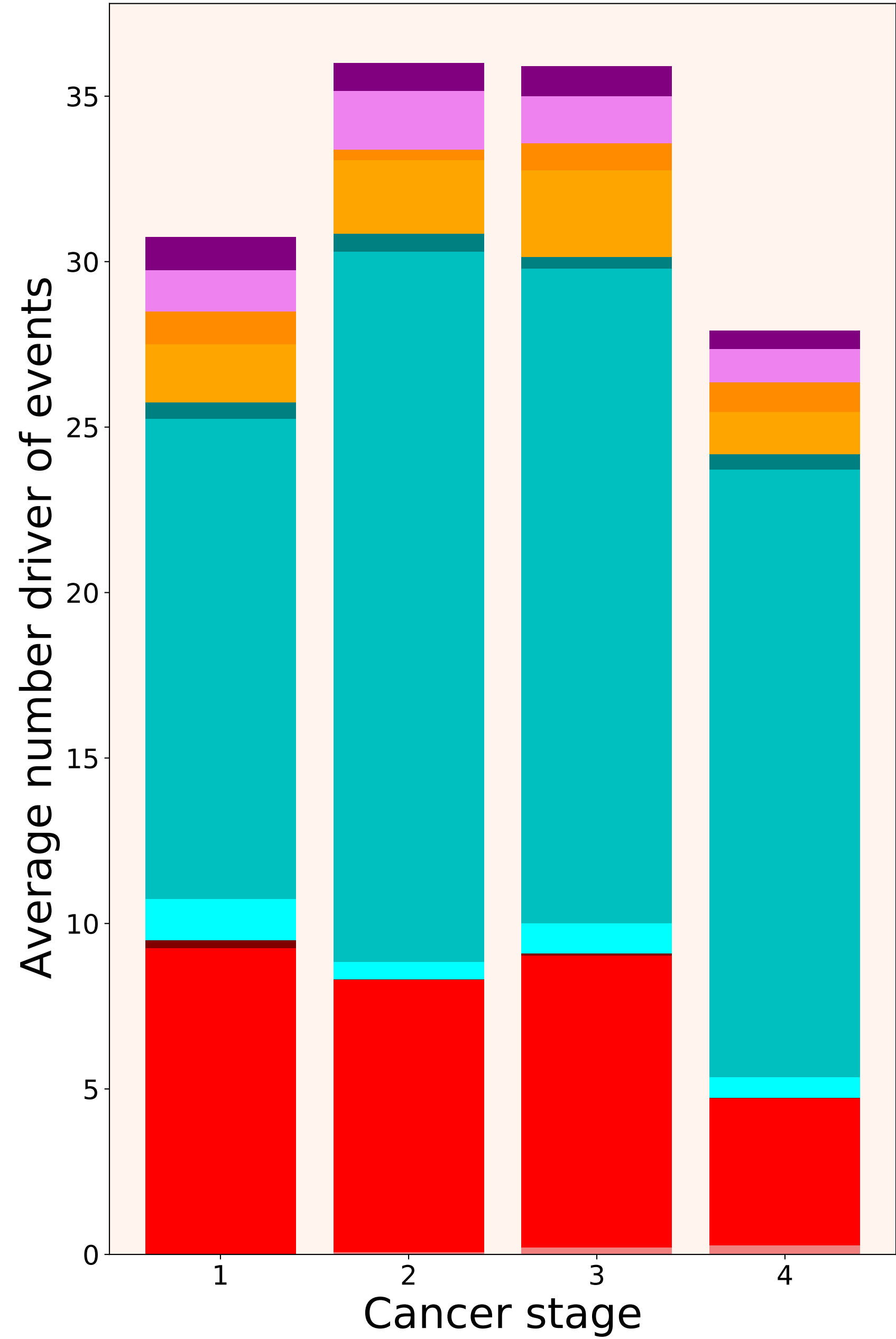

Supplement: S2 Files — (ZIP) [file pgen.1009996.s002.zip › PANCAN/cumulative histograms/Distribution_stages_cohorts/2021_11_23_14_43_distribution_stages_MESO.pdf]

Driver event distribution by cancer stage DLBC

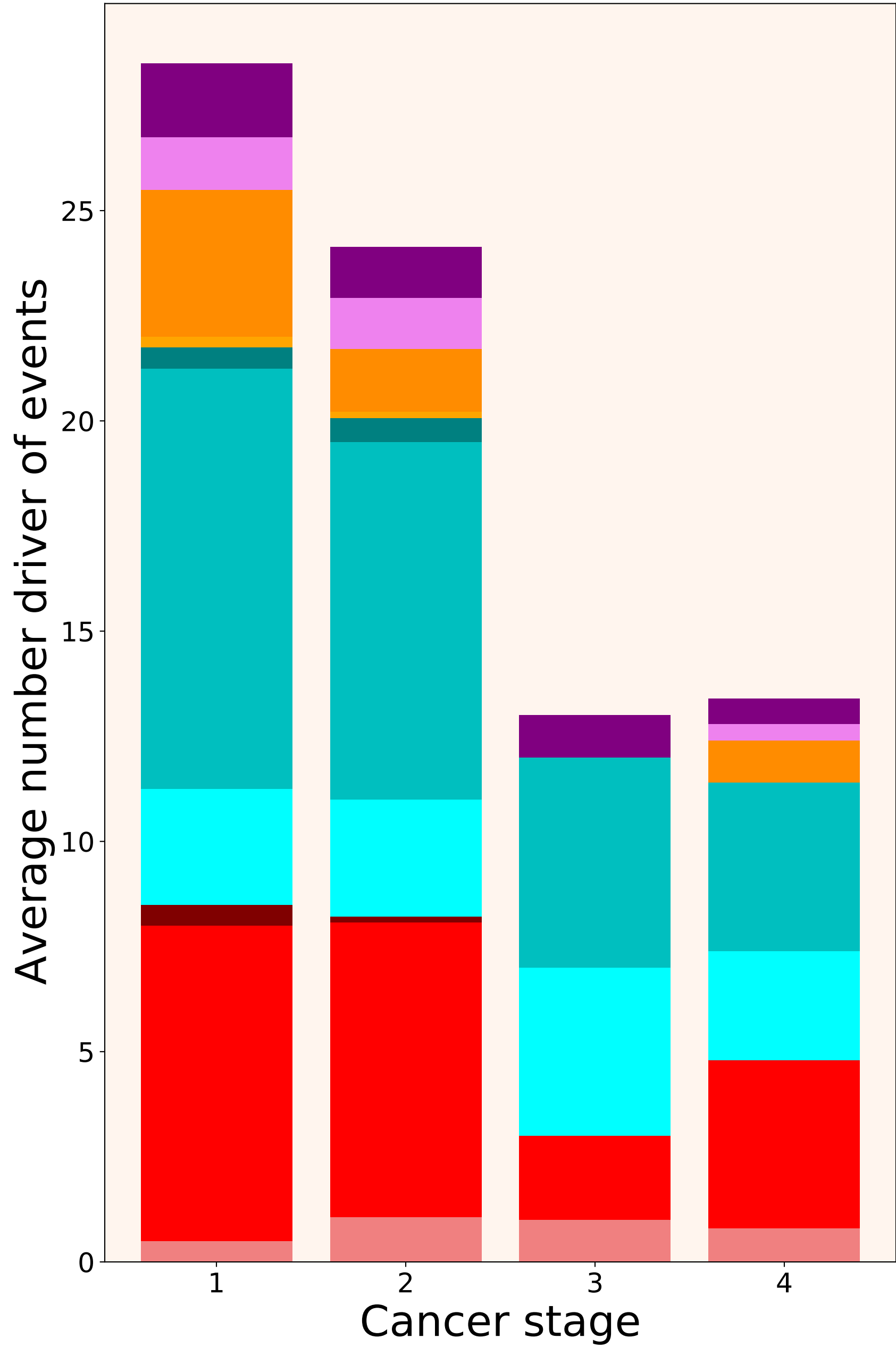

Supplement: S2 Files — (ZIP) [file pgen.1009996.s002.zip › PANCAN/cumulative histograms/Distribution_stages_cohorts/2021_11_23_14_43_distribution_stages_DLBC.pdf]

Driver event distribution by cancer stage in males LUSC

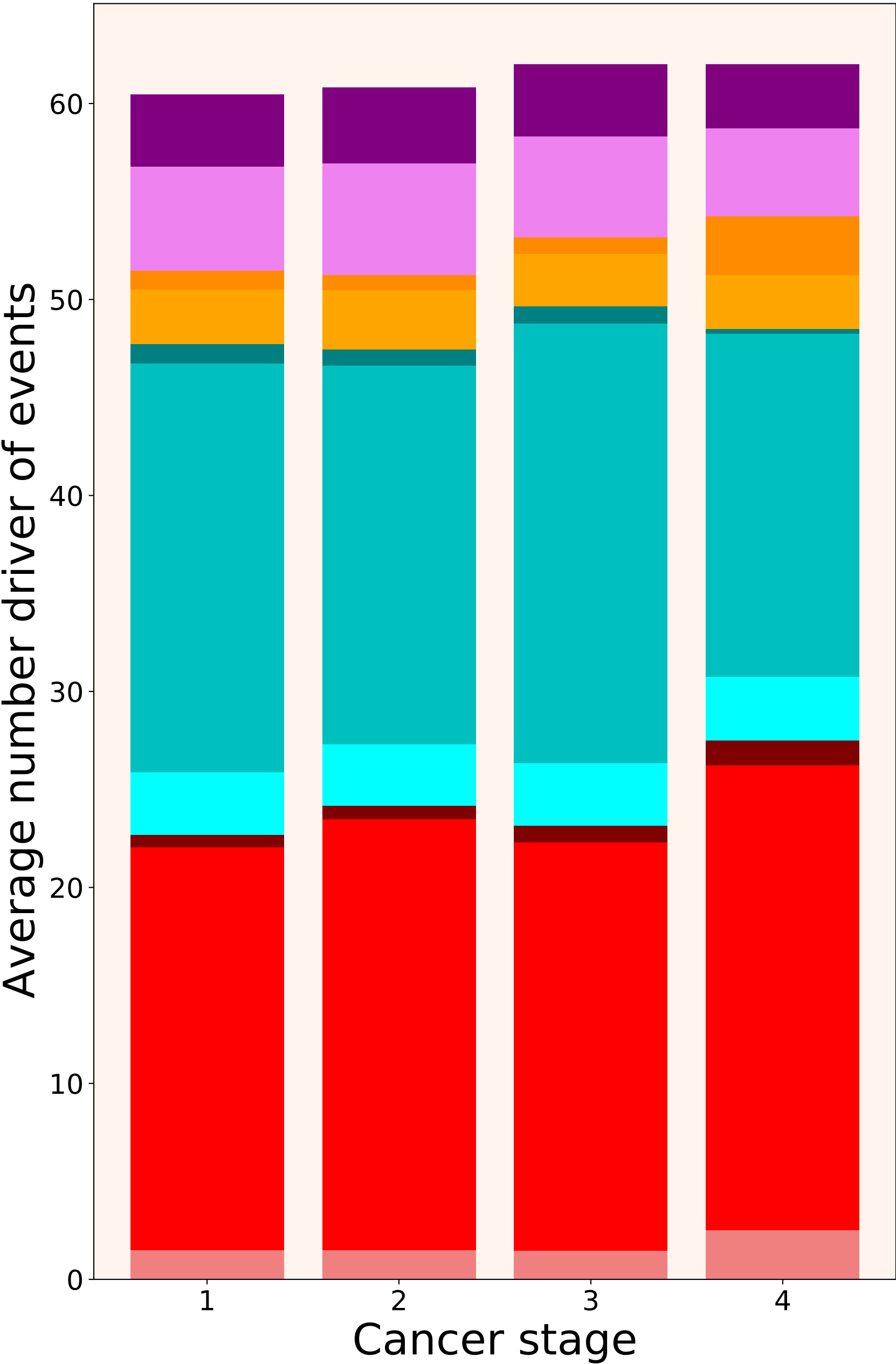

Supplement: S2 Files — (ZIP) [file pgen.1009996.s002.zip › PANCAN/cumulative histograms/Distribution_stages_cohorts/2021_11_23_14_43_distribution_stages_males_LUSC.pdf]

Driver event distribution by cancer stage SKCM

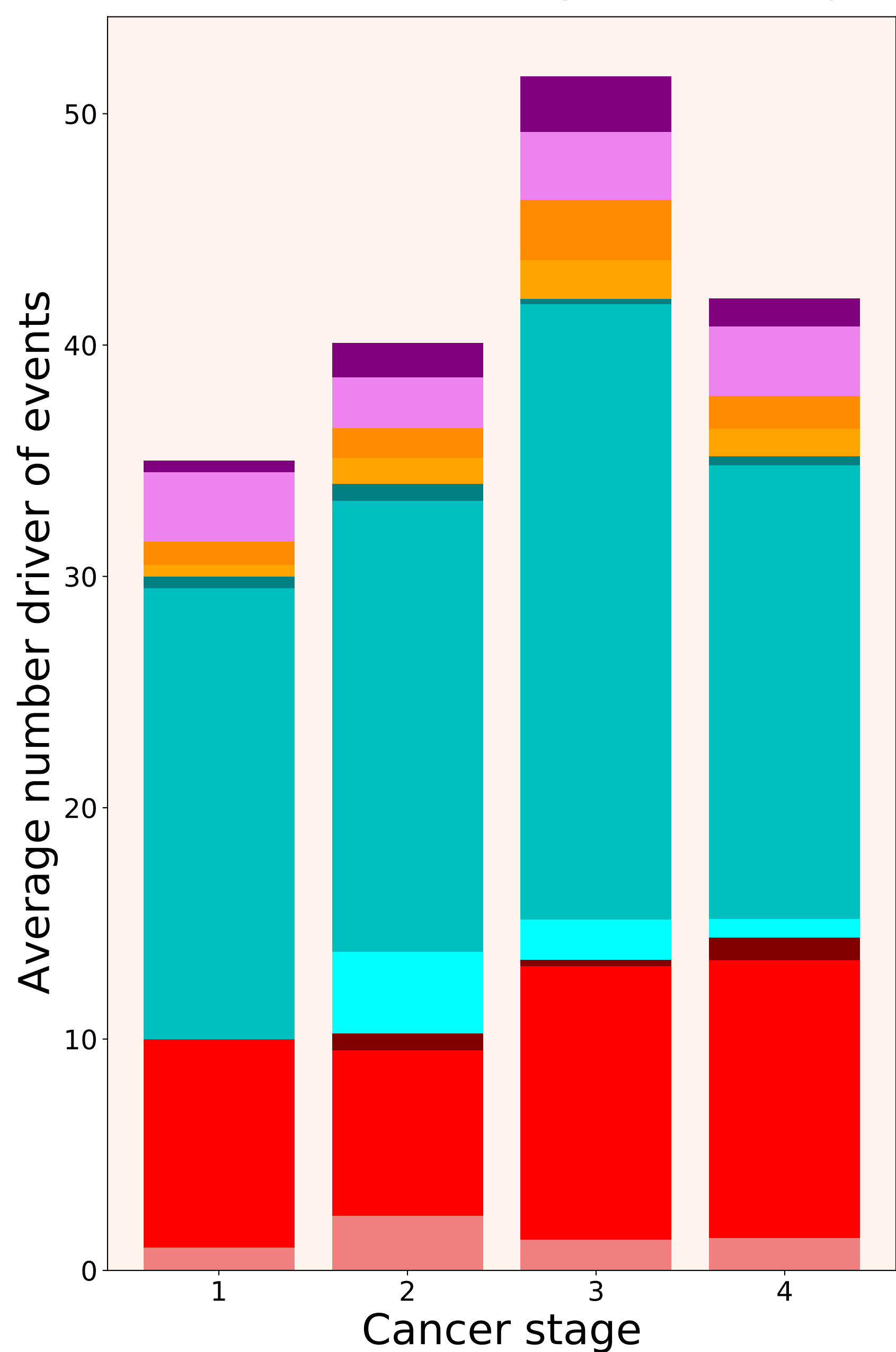

Supplement: S2 Files — (ZIP) [file pgen.1009996.s002.zip › PANCAN/cumulative histograms/Distribution_stages_cohorts/2021_11_23_14_43_distribution_stages_SKCM.pdf]

Driver event distribution by cancer stage in females BRCA

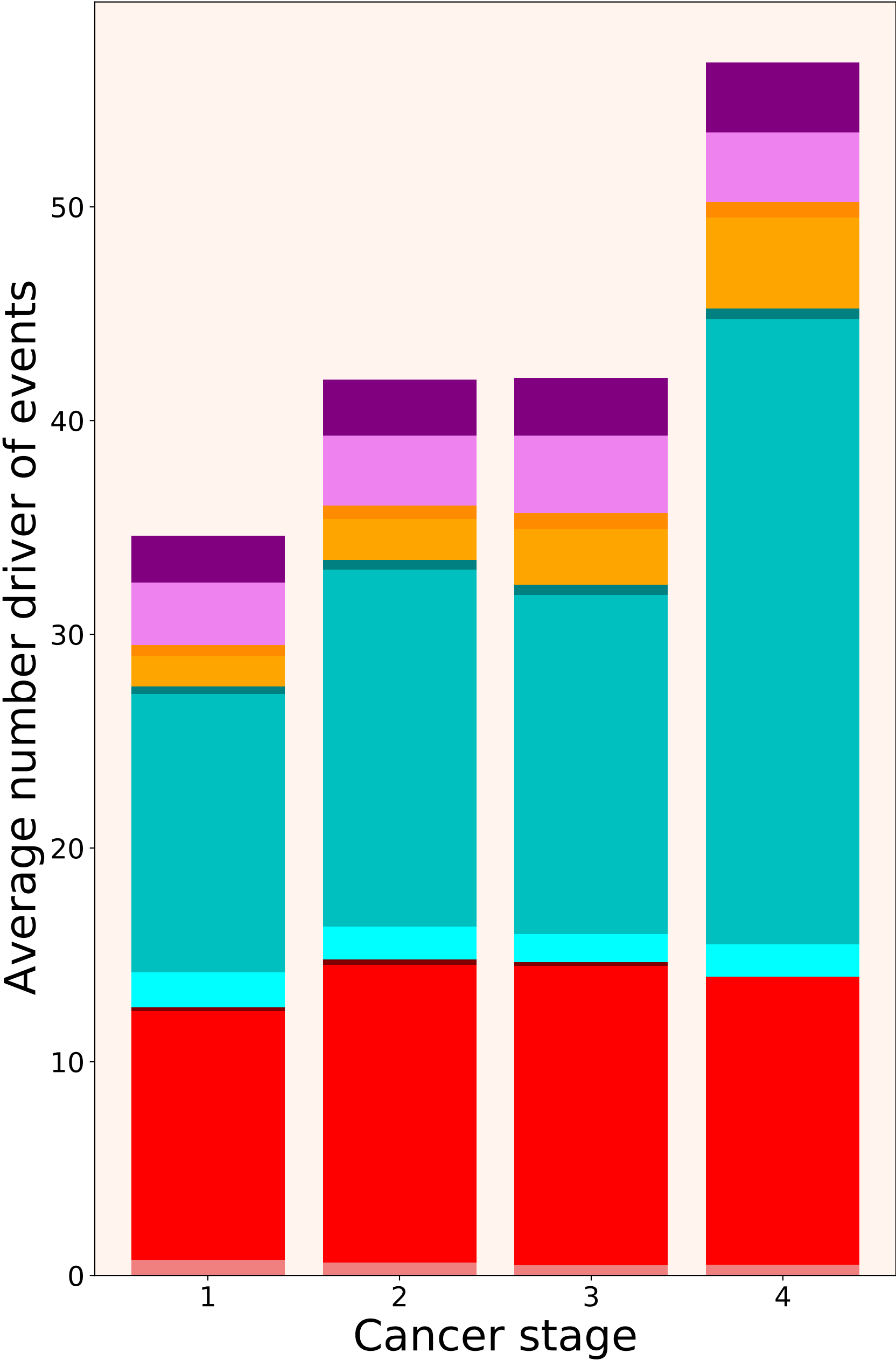

Supplement: S2 Files — (ZIP) [file pgen.1009996.s002.zip › PANCAN/cumulative histograms/Distribution_stages_cohorts/2021_11_23_14_43_distribution_stages_females_BRCA.pdf]

Driver event distribution by cancer stage in females OV

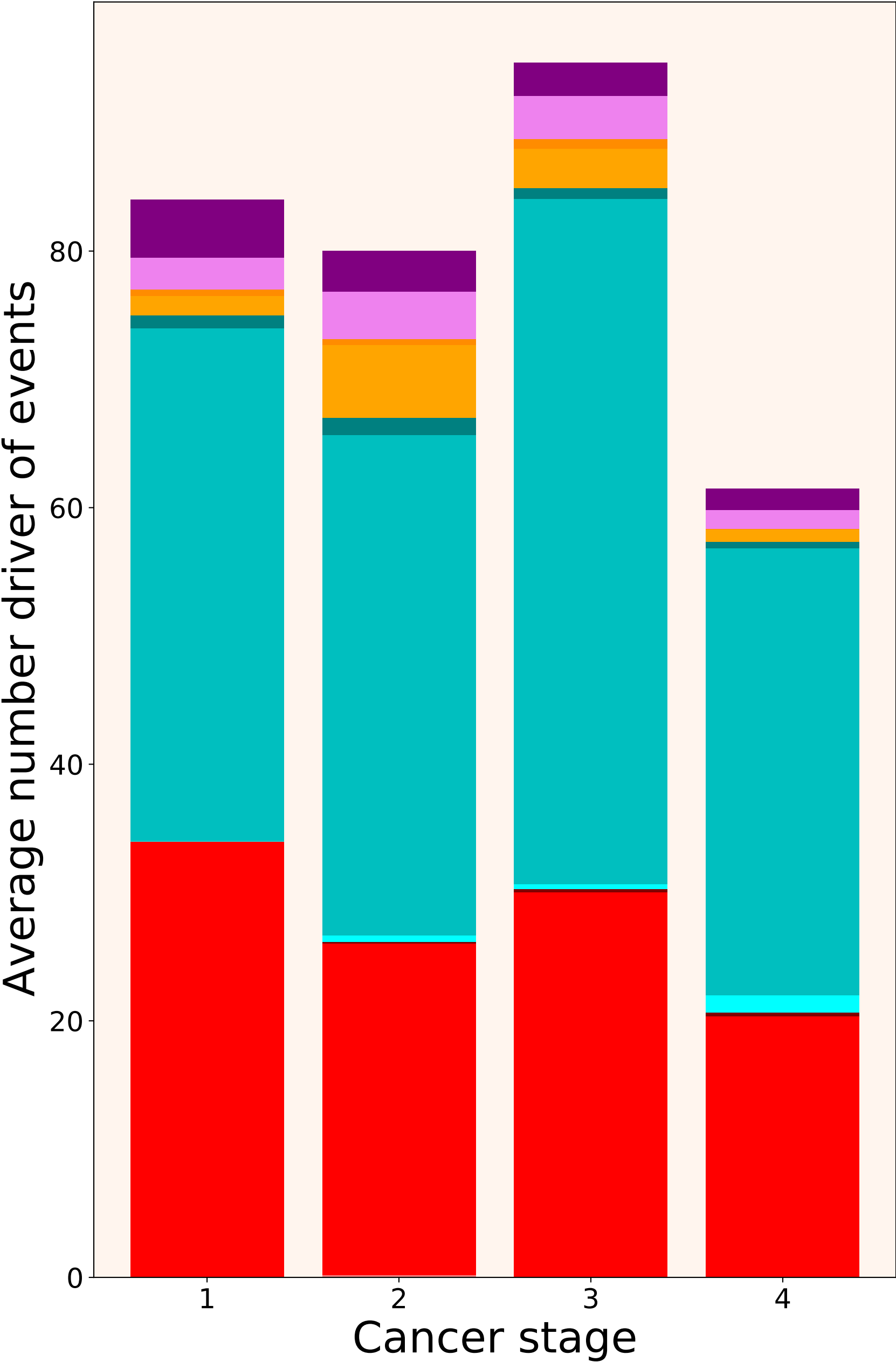

Supplement: S2 Files — (ZIP) [file pgen.1009996.s002.zip › PANCAN/cumulative histograms/Distribution_stages_cohorts/2021_11_23_14_43_distribution_stages_females_OV.pdf]

Driver event distribution by cancer stage in males TGCT

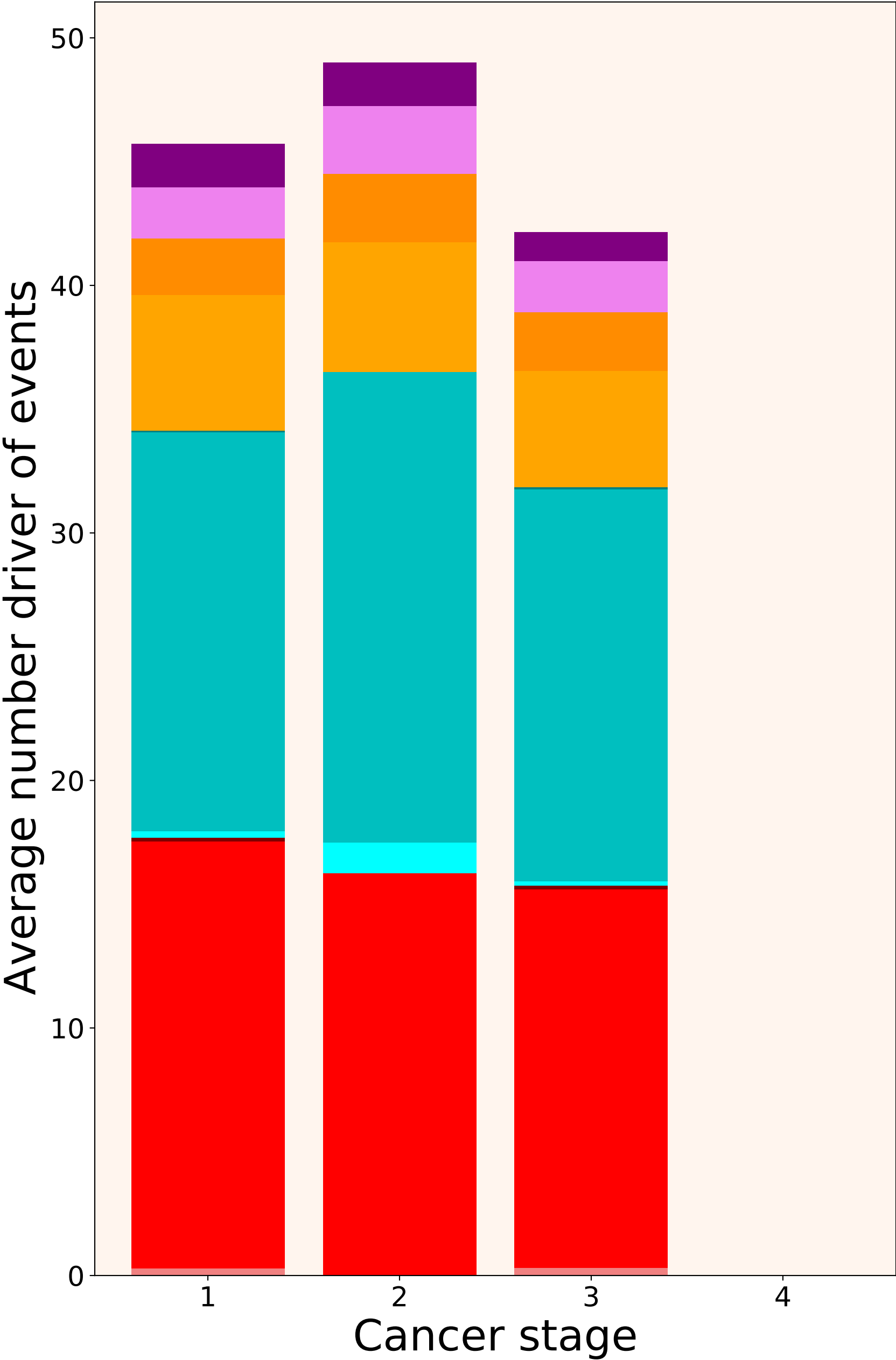

Supplement: S2 Files — (ZIP) [file pgen.1009996.s002.zip › PANCAN/cumulative histograms/Distribution_stages_cohorts/2021_11_23_14_43_distribution_stages_males_TGCT.pdf]

Driver event distribution by cancer stage STAD

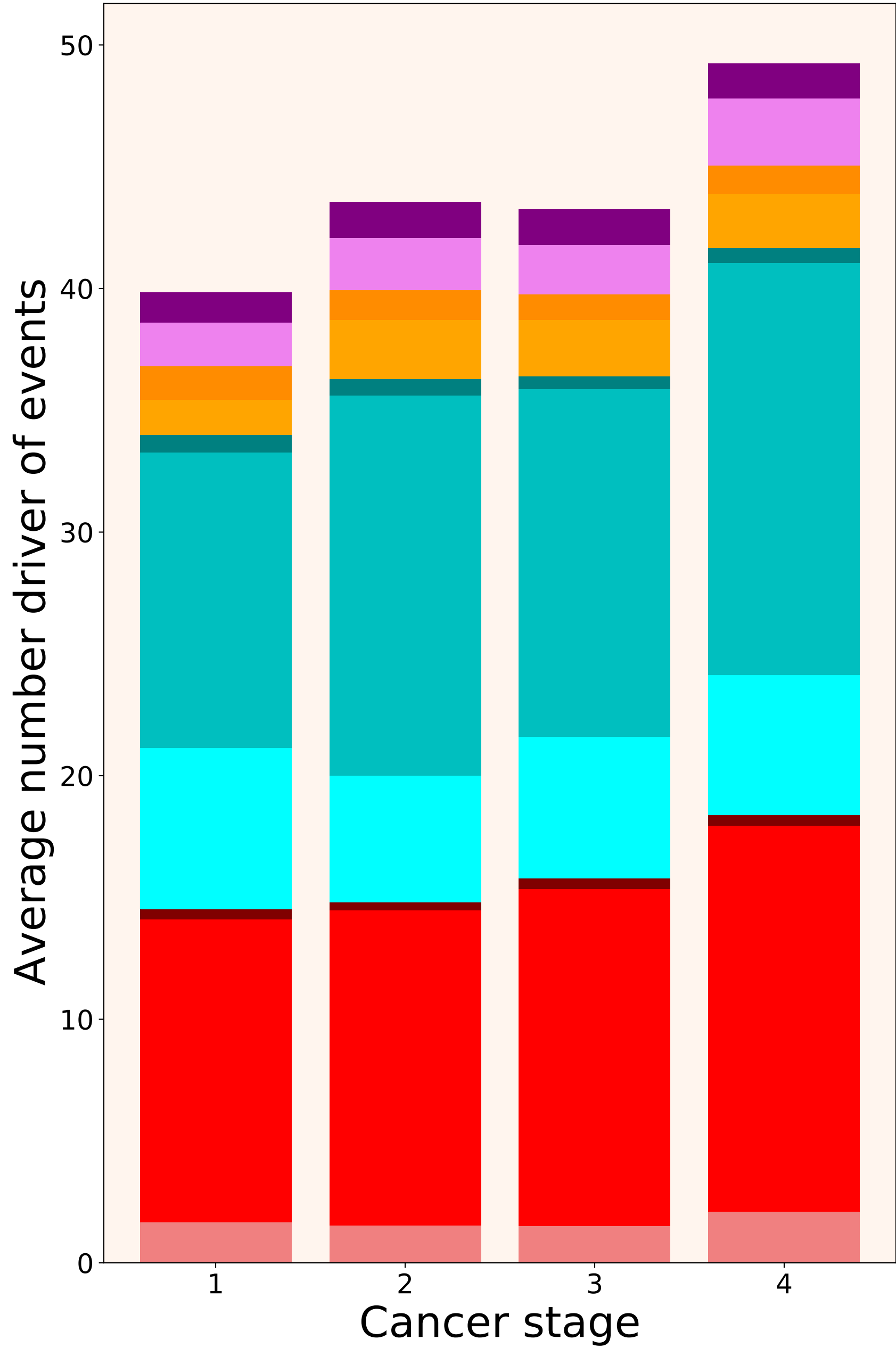

Supplement: S2 Files — (ZIP) [file pgen.1009996.s002.zip › PANCAN/cumulative histograms/Distribution_stages_cohorts/2021_11_23_14_43_distribution_stages_STAD.pdf]

Driver event distribution by cancer stage CHOL

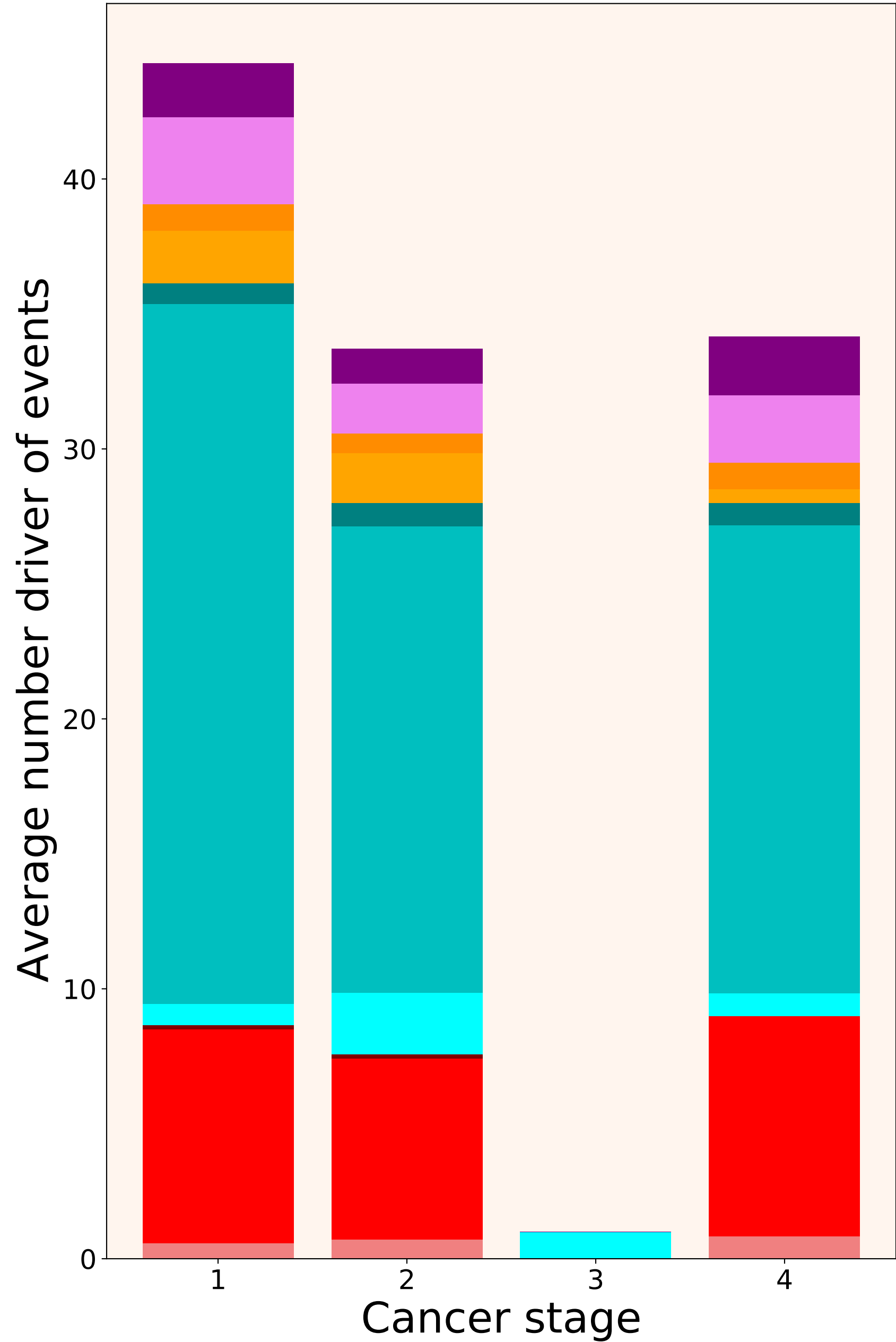

Supplement: S2 Files — (ZIP) [file pgen.1009996.s002.zip › PANCAN/cumulative histograms/Distribution_stages_cohorts/2021_11_23_14_43_distribution_stages_CHOL.pdf]

Driver event distribution by cancer stage in females UCEC

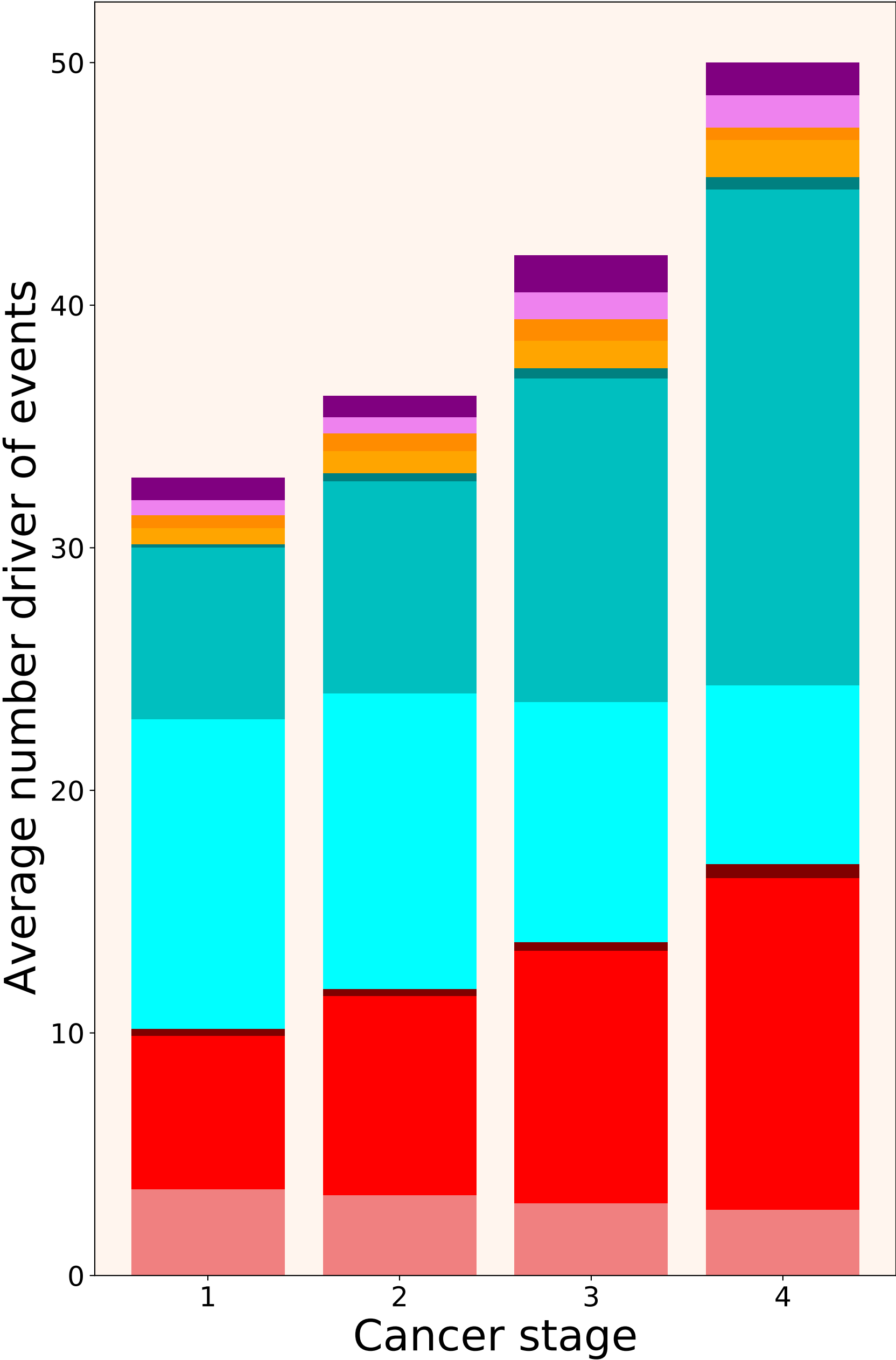

Supplement: S2 Files — (ZIP) [file pgen.1009996.s002.zip › PANCAN/cumulative histograms/Distribution_stages_cohorts/2021_11_23_14_43_distribution_stages_females_UCEC.pdf]

Driver event distribution by cancer stage in females KICH

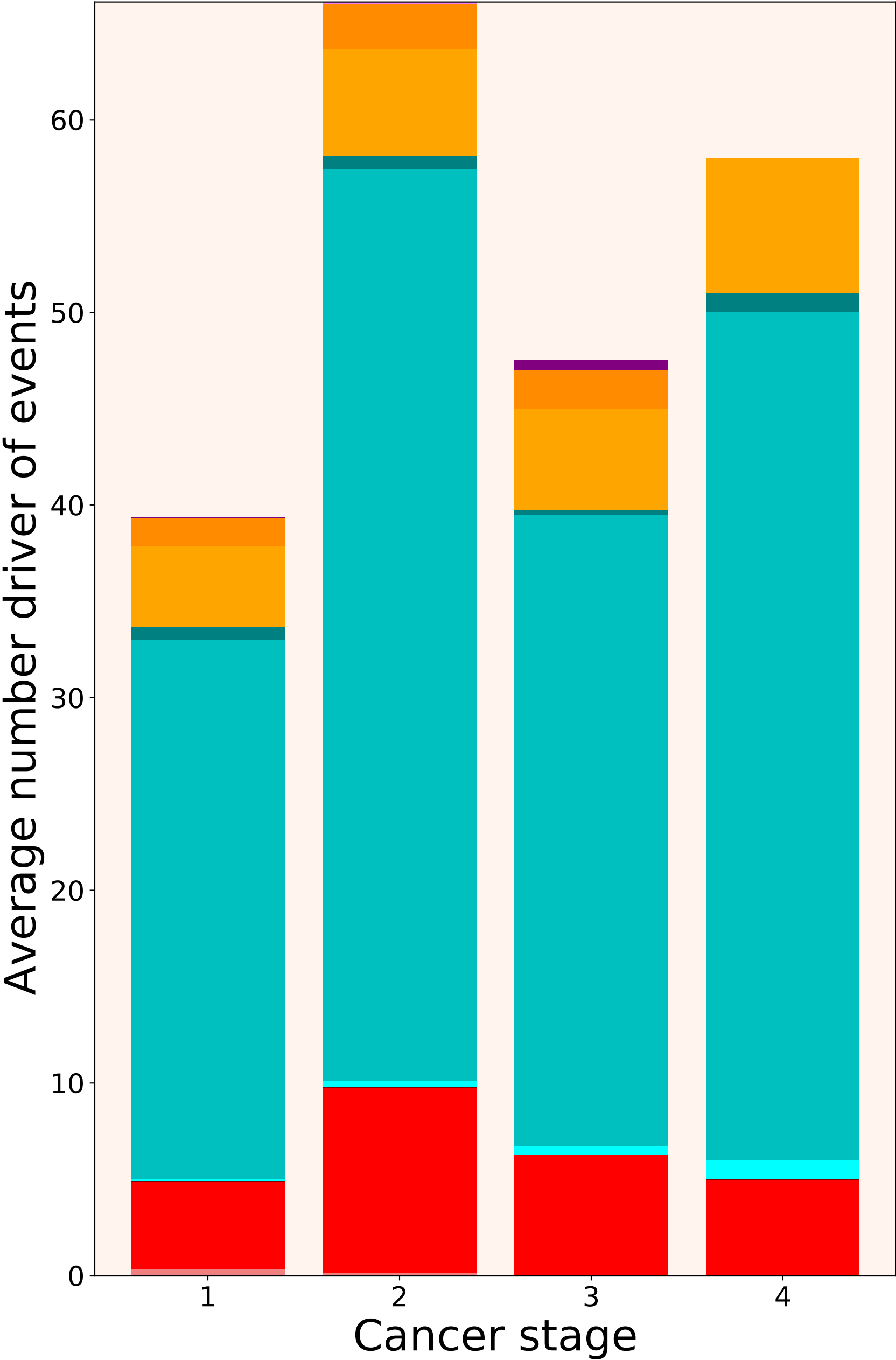

Supplement: S2 Files — (ZIP) [file pgen.1009996.s002.zip › PANCAN/cumulative histograms/Distribution_stages_cohorts/2021_11_23_14_43_distribution_stages_females_KICH.pdf]

Driver event distribution by cancer stage KICH

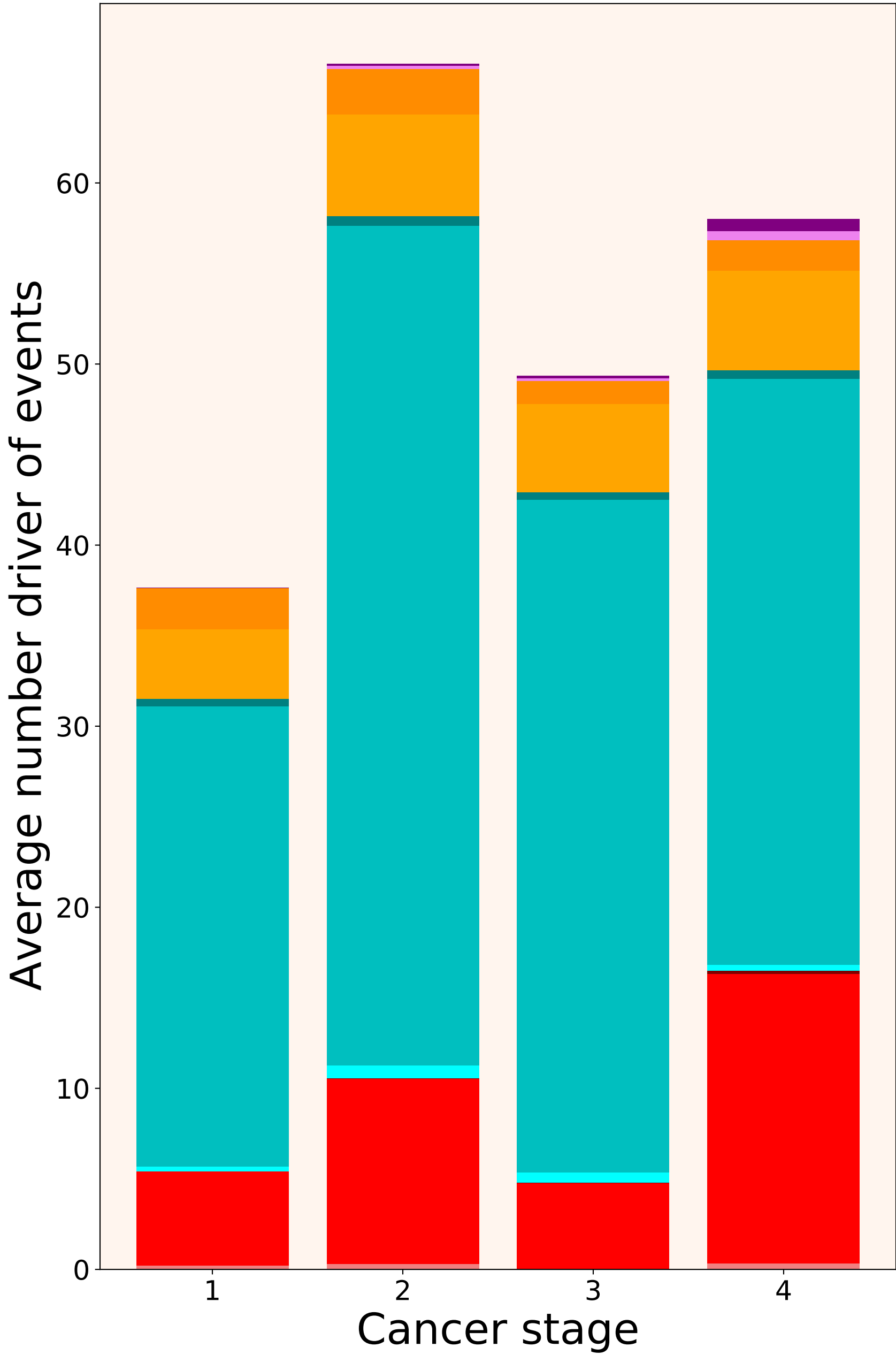

Supplement: S2 Files — (ZIP) [file pgen.1009996.s002.zip › PANCAN/cumulative histograms/Distribution_stages_cohorts/2021_11_23_14_43_distribution_stages_KICH.pdf]

Driver event distribution by cancer stage UCEC

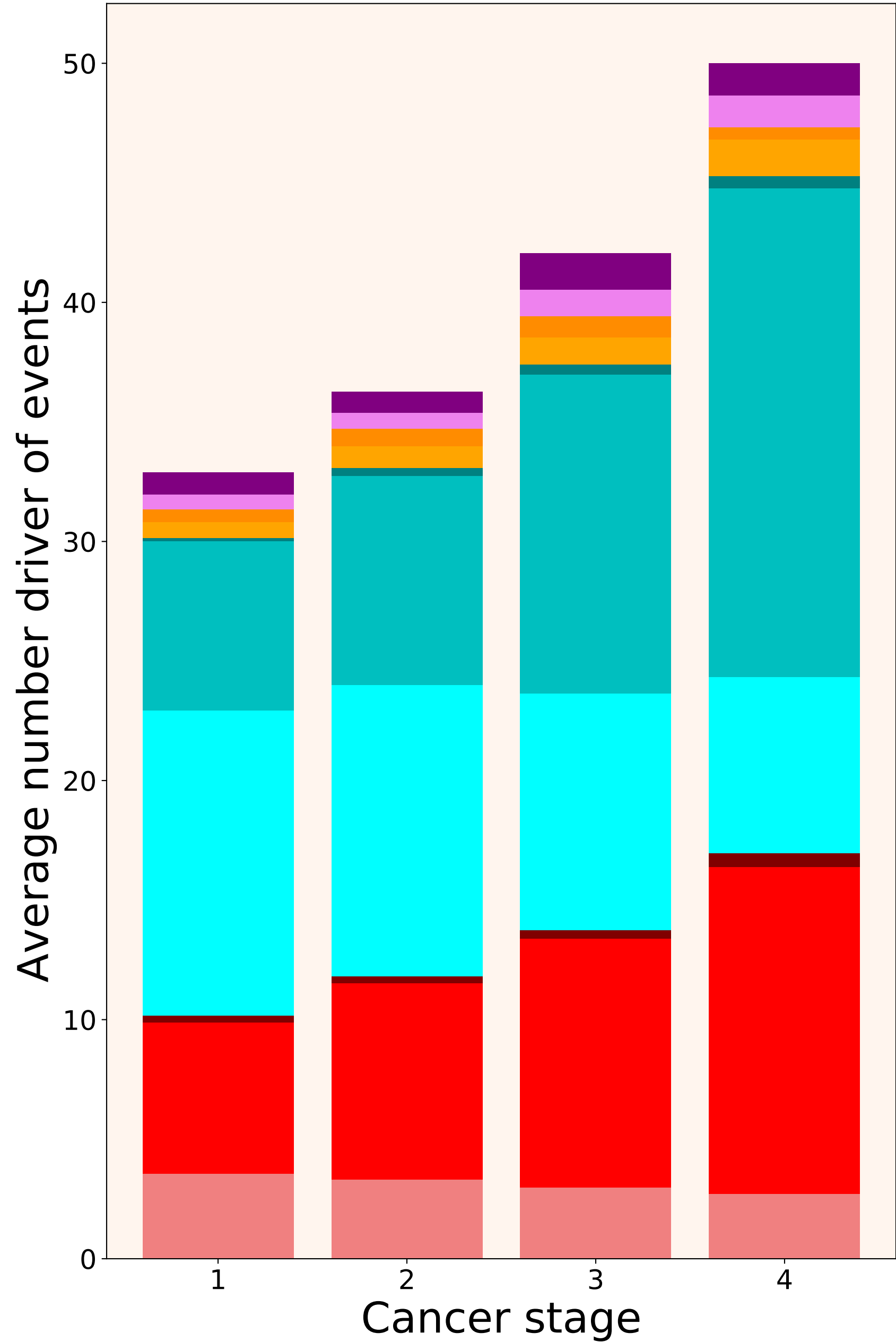

Supplement: S2 Files — (ZIP) [file pgen.1009996.s002.zip › PANCAN/cumulative histograms/Distribution_stages_cohorts/2021_11_23_14_43_distribution_stages_UCEC.pdf]

Driver event distribution by cancer stage in females STAD

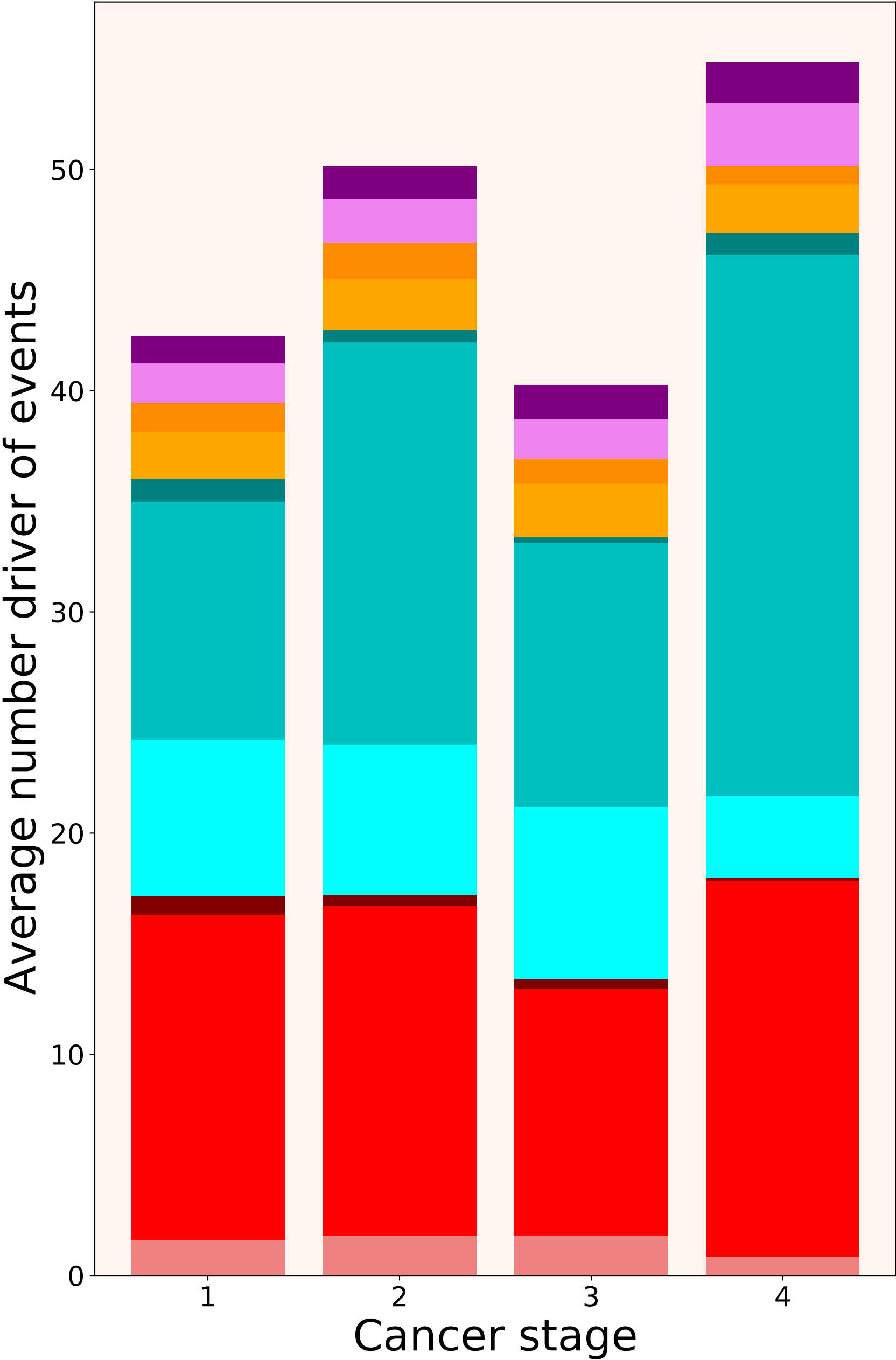

Supplement: S2 Files — (ZIP) [file pgen.1009996.s002.zip › PANCAN/cumulative histograms/Distribution_stages_cohorts/2021_11_23_14_43_distribution_stages_females_STAD.pdf]

Driver event distribution by cancer stage in females CHOL

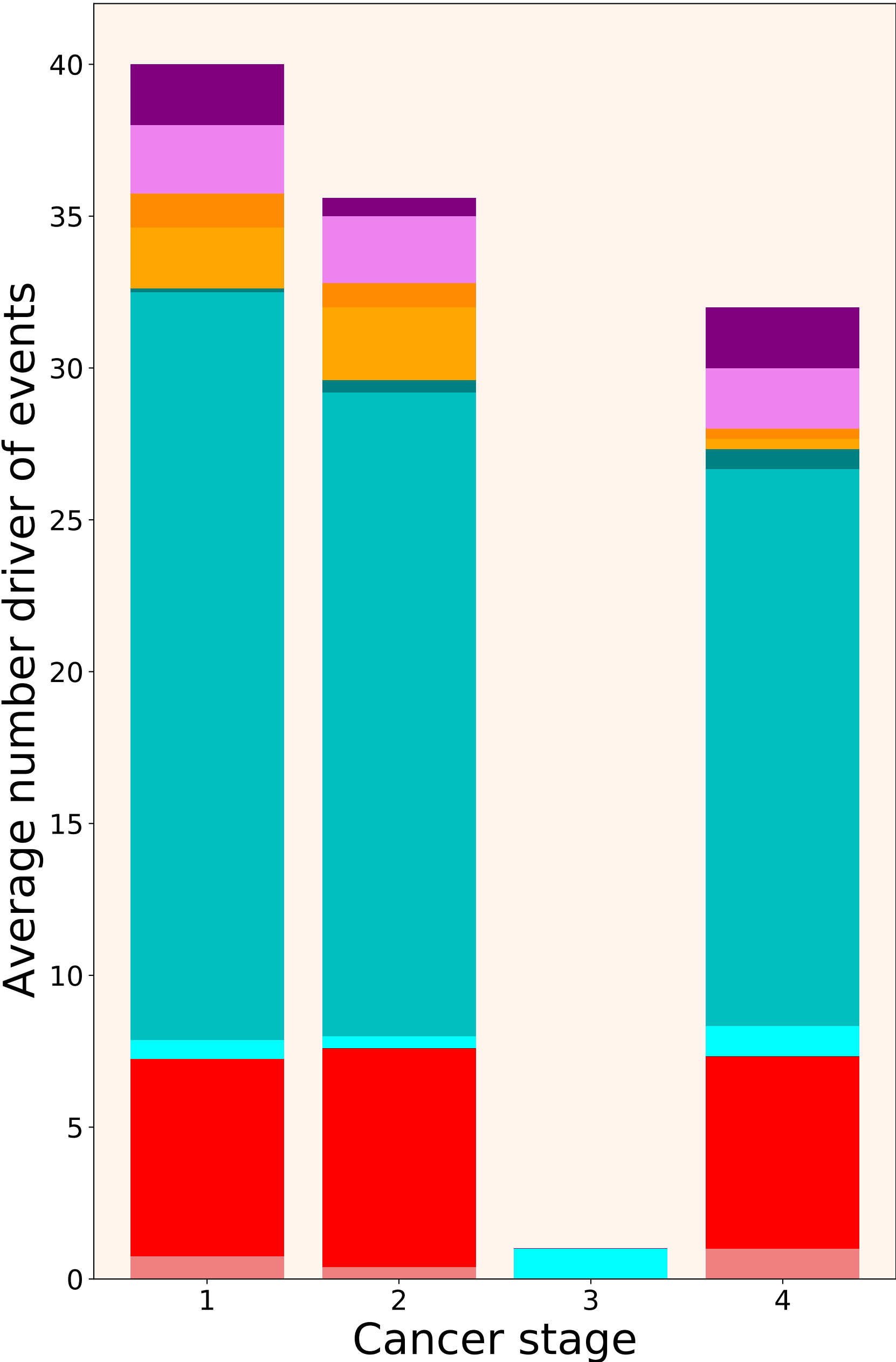

Supplement: S2 Files — (ZIP) [file pgen.1009996.s002.zip › PANCAN/cumulative histograms/Distribution_stages_cohorts/2021_11_23_14_43_distribution_stages_females_CHOL.pdf]

Driver event distribution by cancer stage UCS

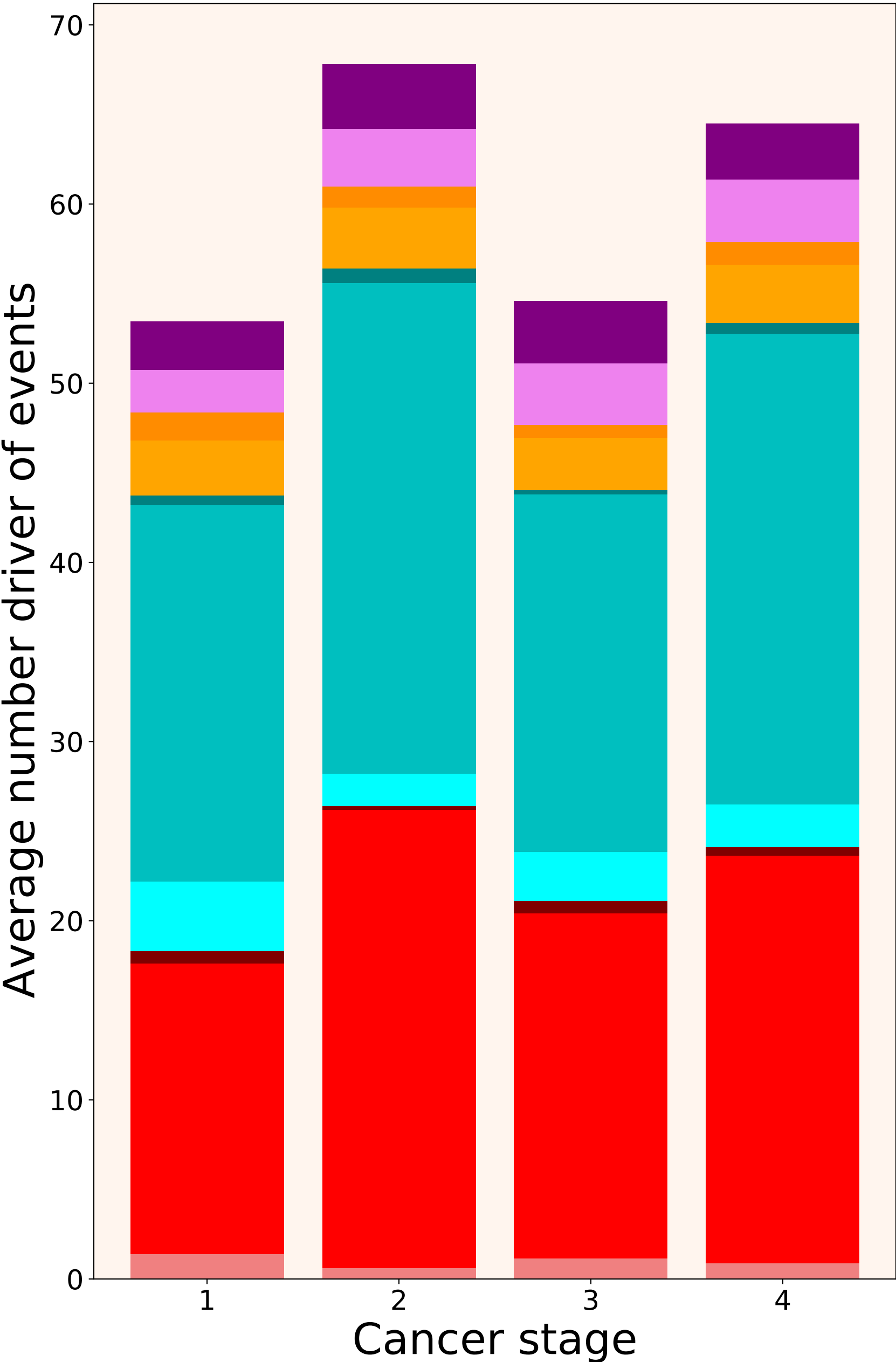

Supplement: S2 Files — (ZIP) [file pgen.1009996.s002.zip › PANCAN/cumulative histograms/Distribution_stages_cohorts/2021_11_23_14_43_distribution_stages_UCS.pdf]

Driver event distribution by cancer stage COAD

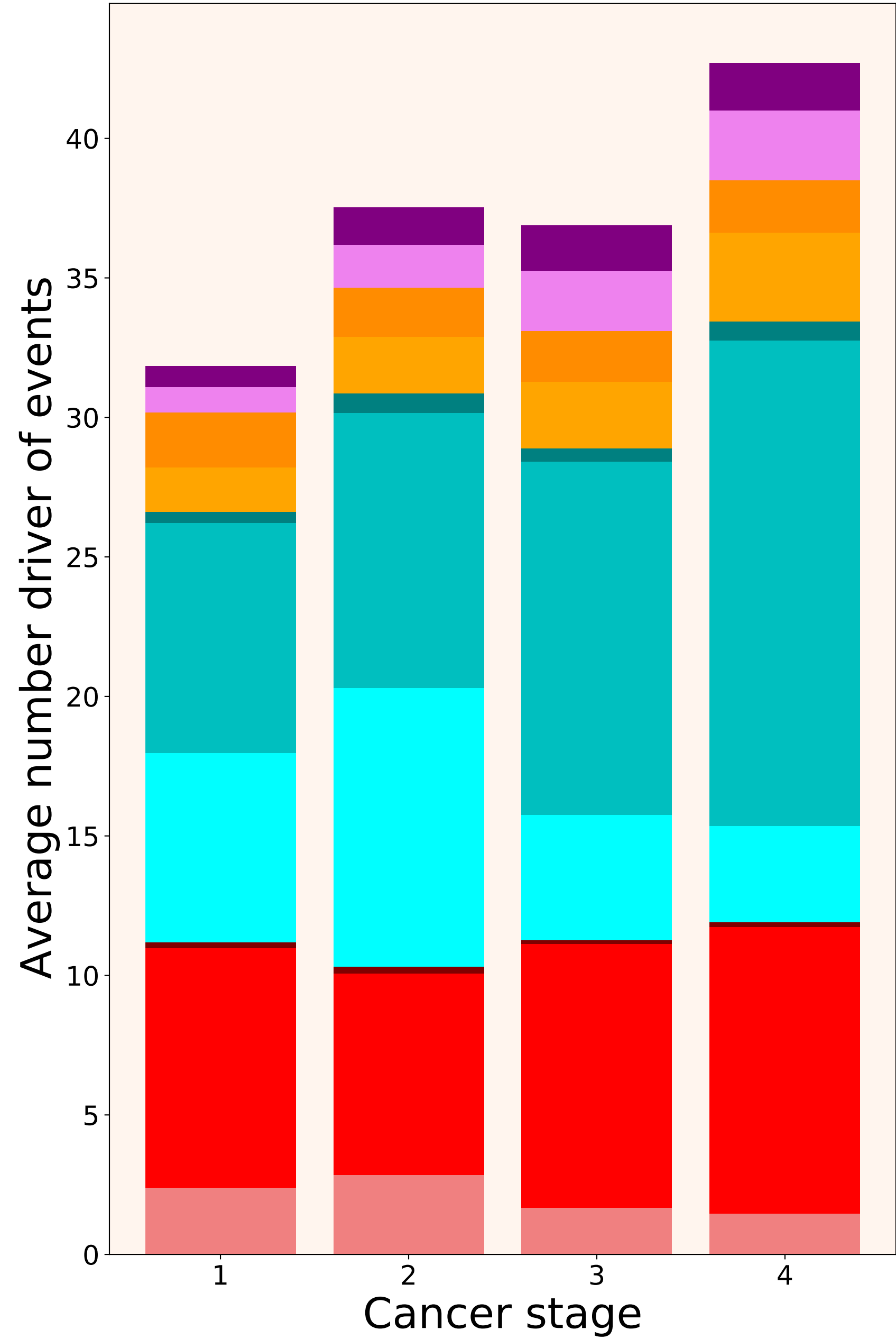

Supplement: S2 Files — (ZIP) [file pgen.1009996.s002.zip › PANCAN/cumulative histograms/Distribution_stages_cohorts/2021_11_23_14_43_distribution_stages_COAD.pdf]

Driver event distribution by cancer stage KIRP

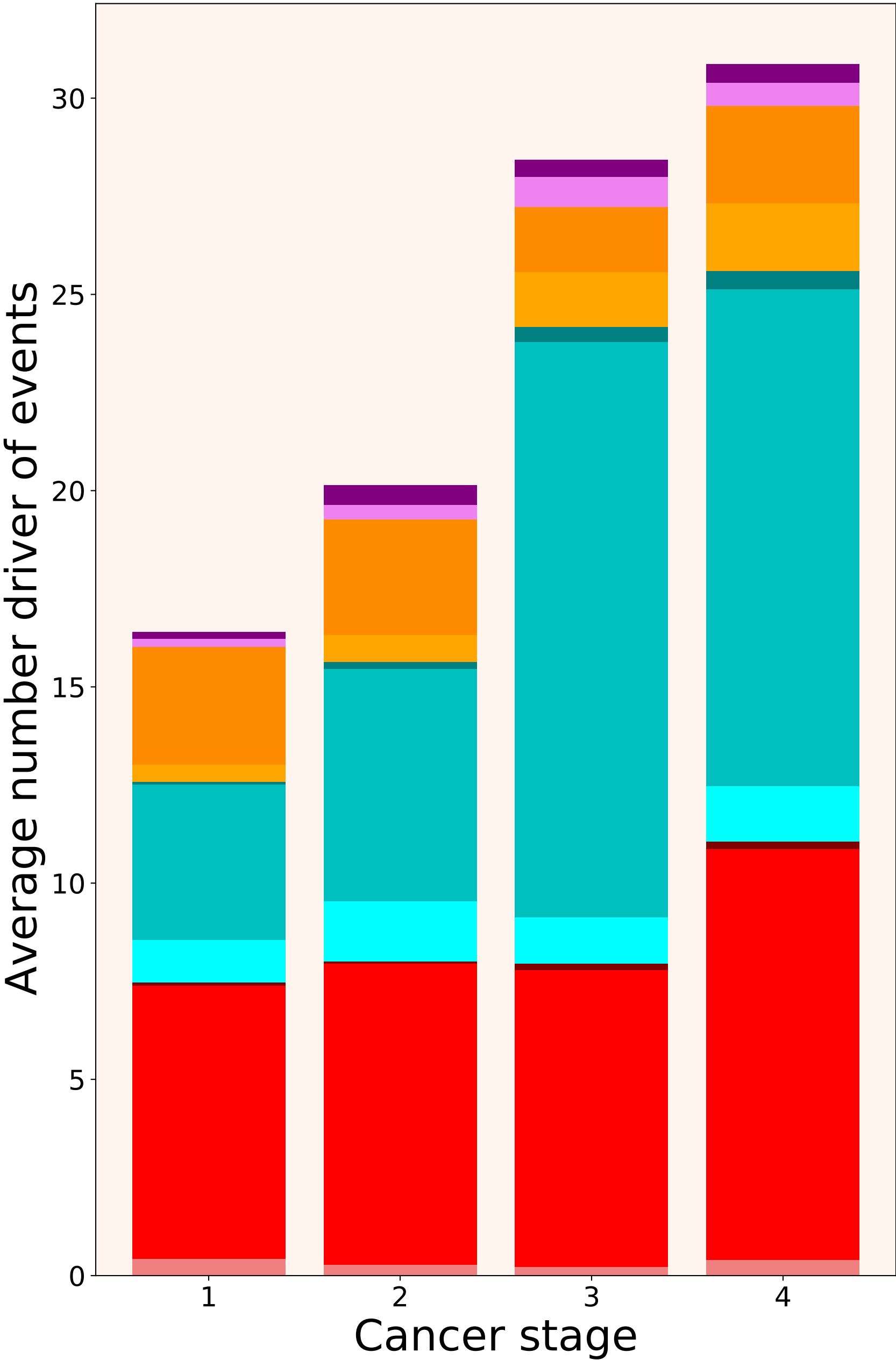

Supplement: S2 Files — (ZIP) [file pgen.1009996.s002.zip › PANCAN/cumulative histograms/Distribution_stages_cohorts/2021_11_23_14_43_distribution_stages_KIRP.pdf]

Driver event distribution by cancer stage in males PRAD

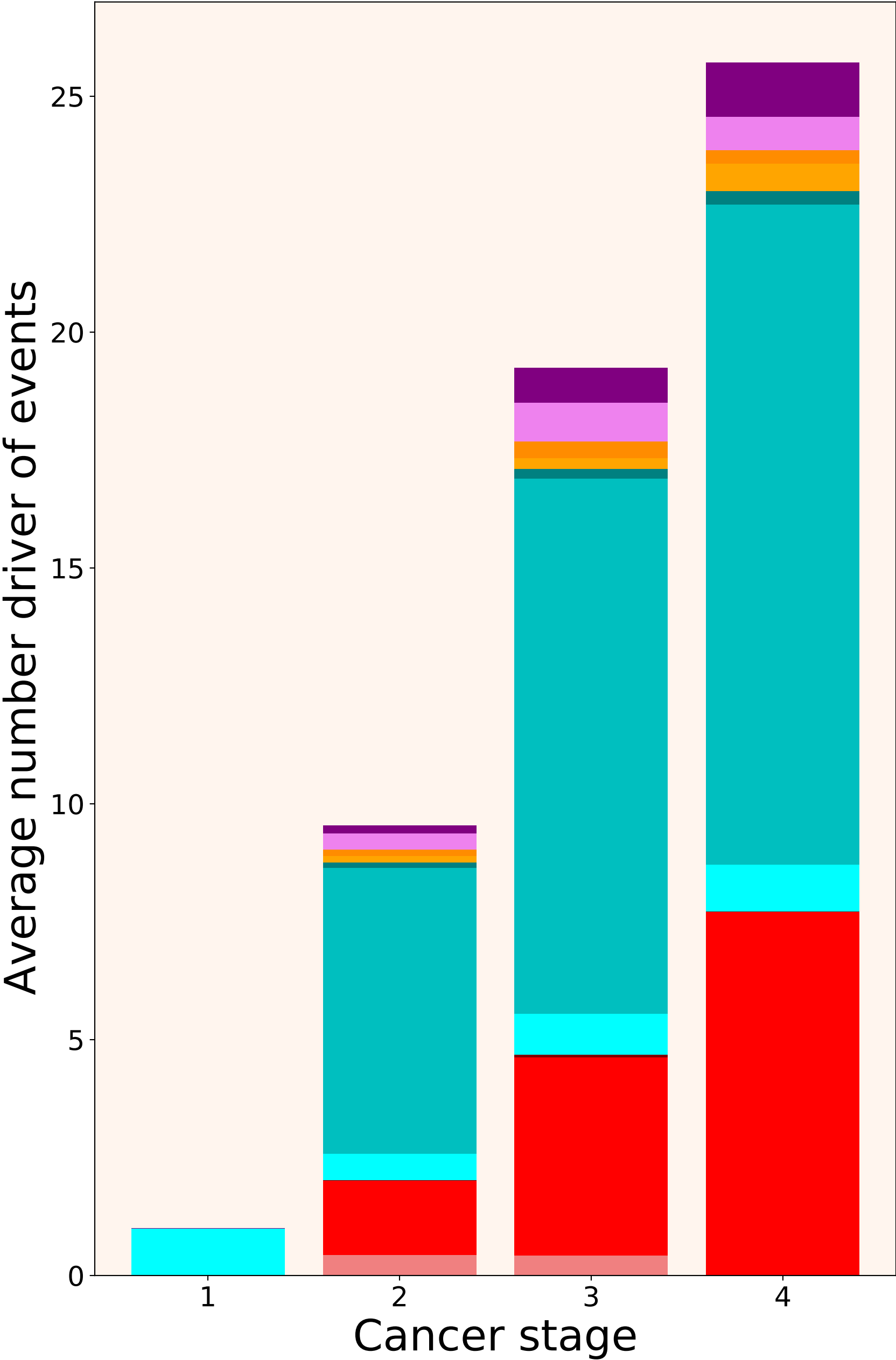

Supplement: S2 Files — (ZIP) [file pgen.1009996.s002.zip › PANCAN/cumulative histograms/Distribution_stages_cohorts/2021_11_23_14_43_distribution_stages_males_PRAD.pdf]

# Driver event distribution by cancer stage in females LIHC

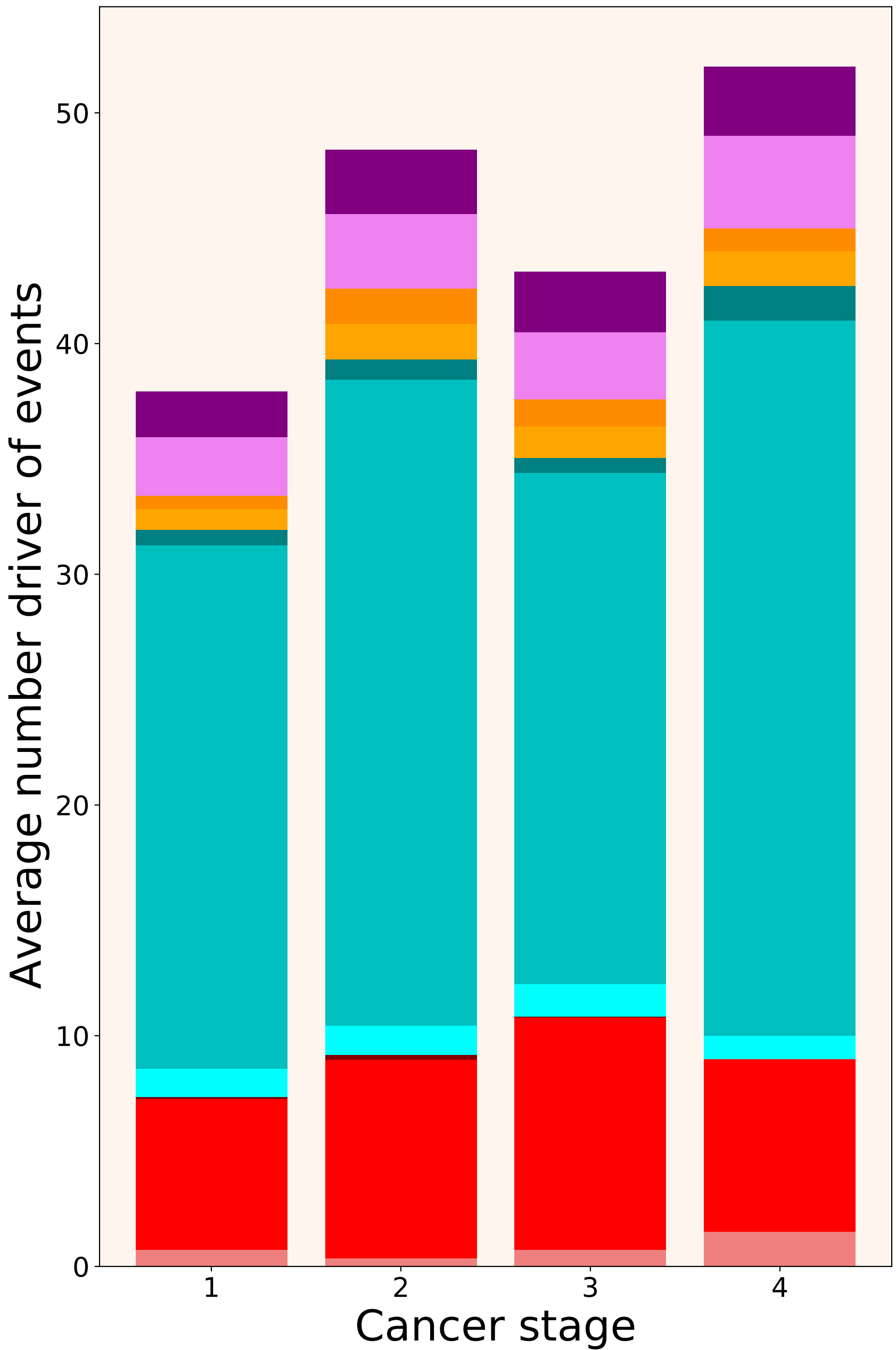

Supplement: S2 Files — (ZIP) [file pgen.1009996.s002.zip › PANCAN/cumulative histograms/Distribution_stages_cohorts/2021_11_23_14_43_distribution_stages_females_LIHC.pdf]

Driver event distribution by cancer stage LIHC

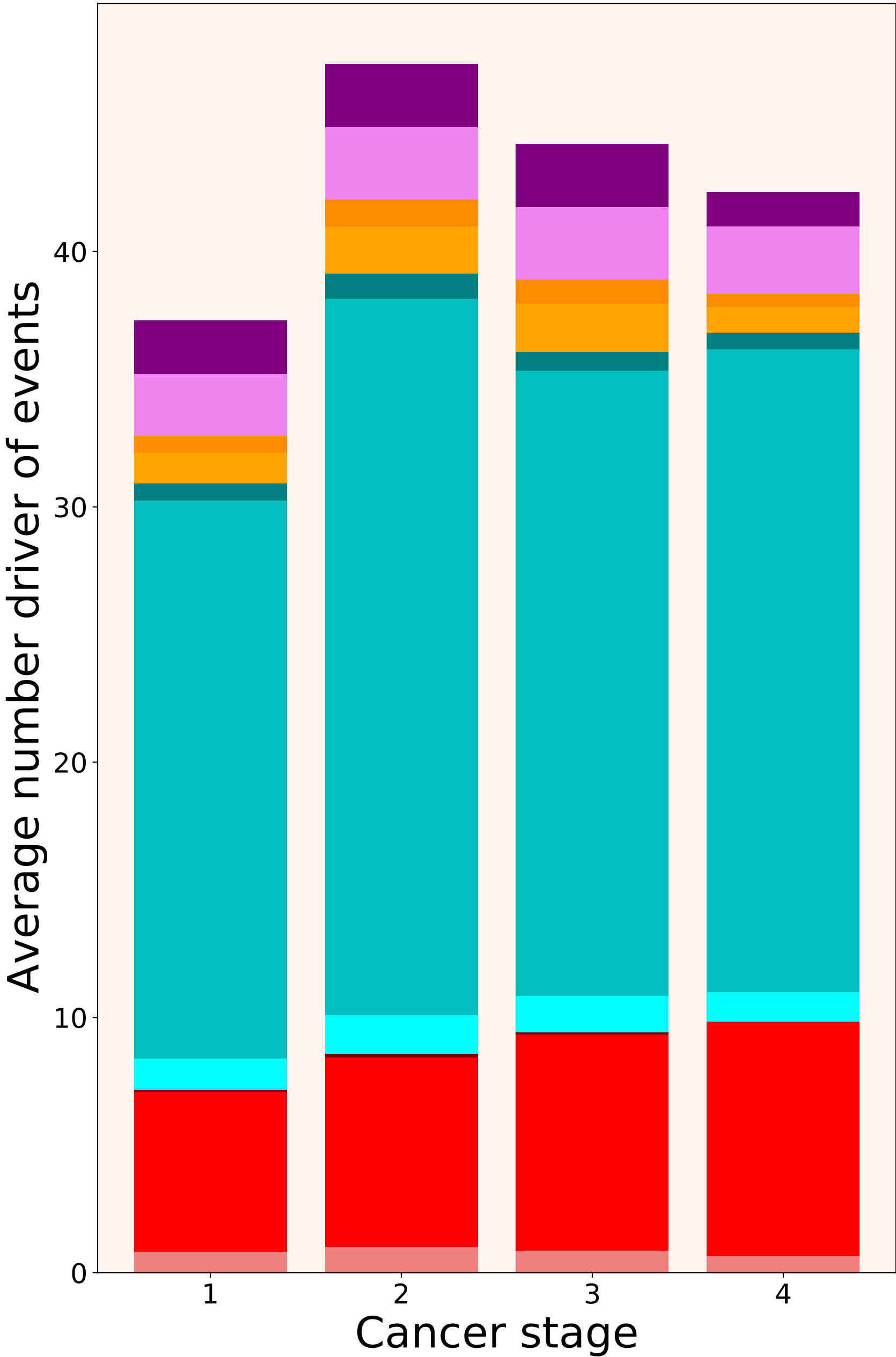

Supplement: S2 Files — (ZIP) [file pgen.1009996.s002.zip › PANCAN/cumulative histograms/Distribution_stages_cohorts/2021_11_23_14_43_distribution_stages_LIHC.pdf]

Driver event distribution by cancer stage in females KIRP

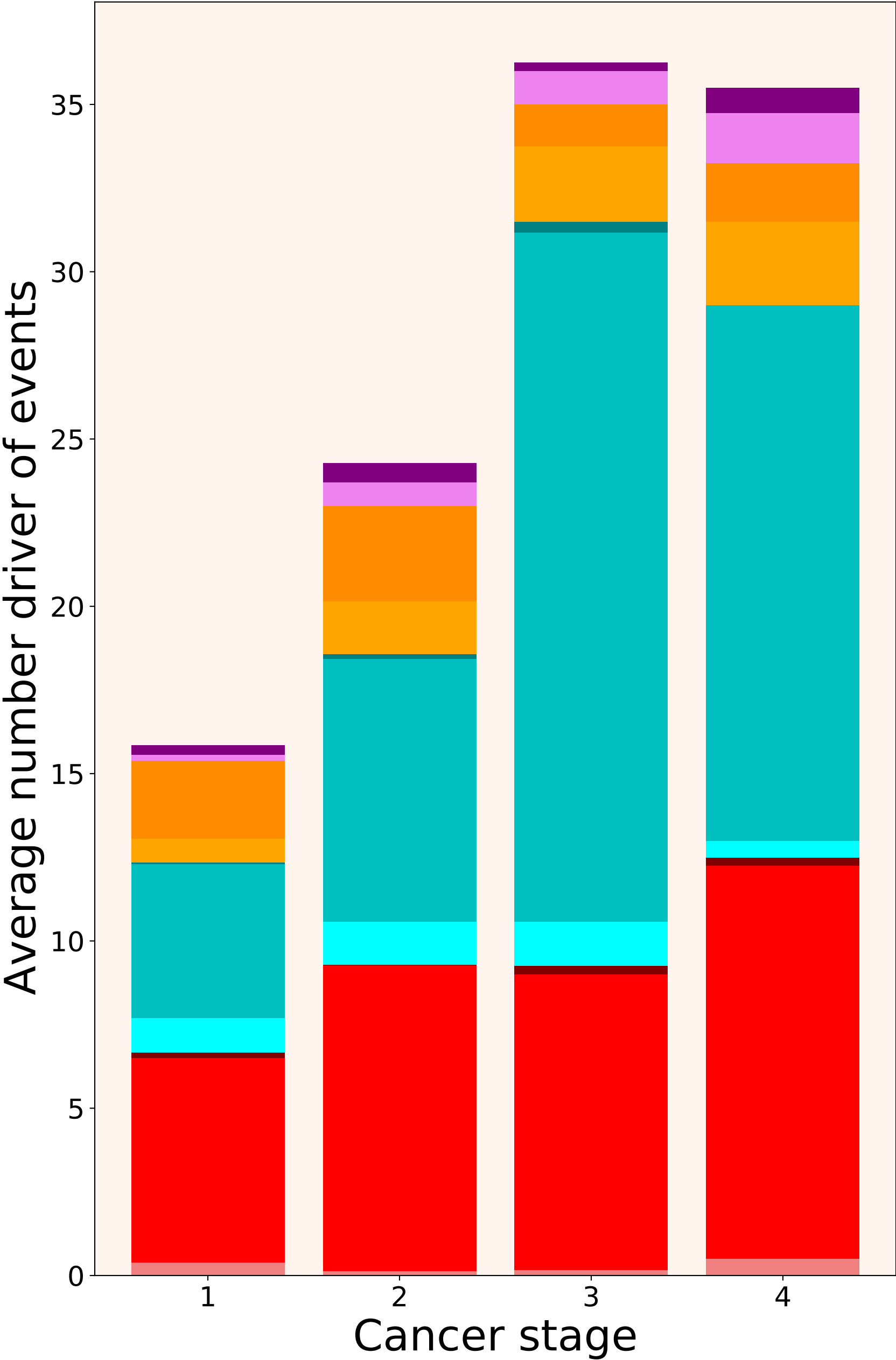

Supplement: S2 Files — (ZIP) [file pgen.1009996.s002.zip › PANCAN/cumulative histograms/Distribution_stages_cohorts/2021_11_23_14_43_distribution_stages_females_KIRP.pdf]

Driver event distribution by cancer stage in females COAD

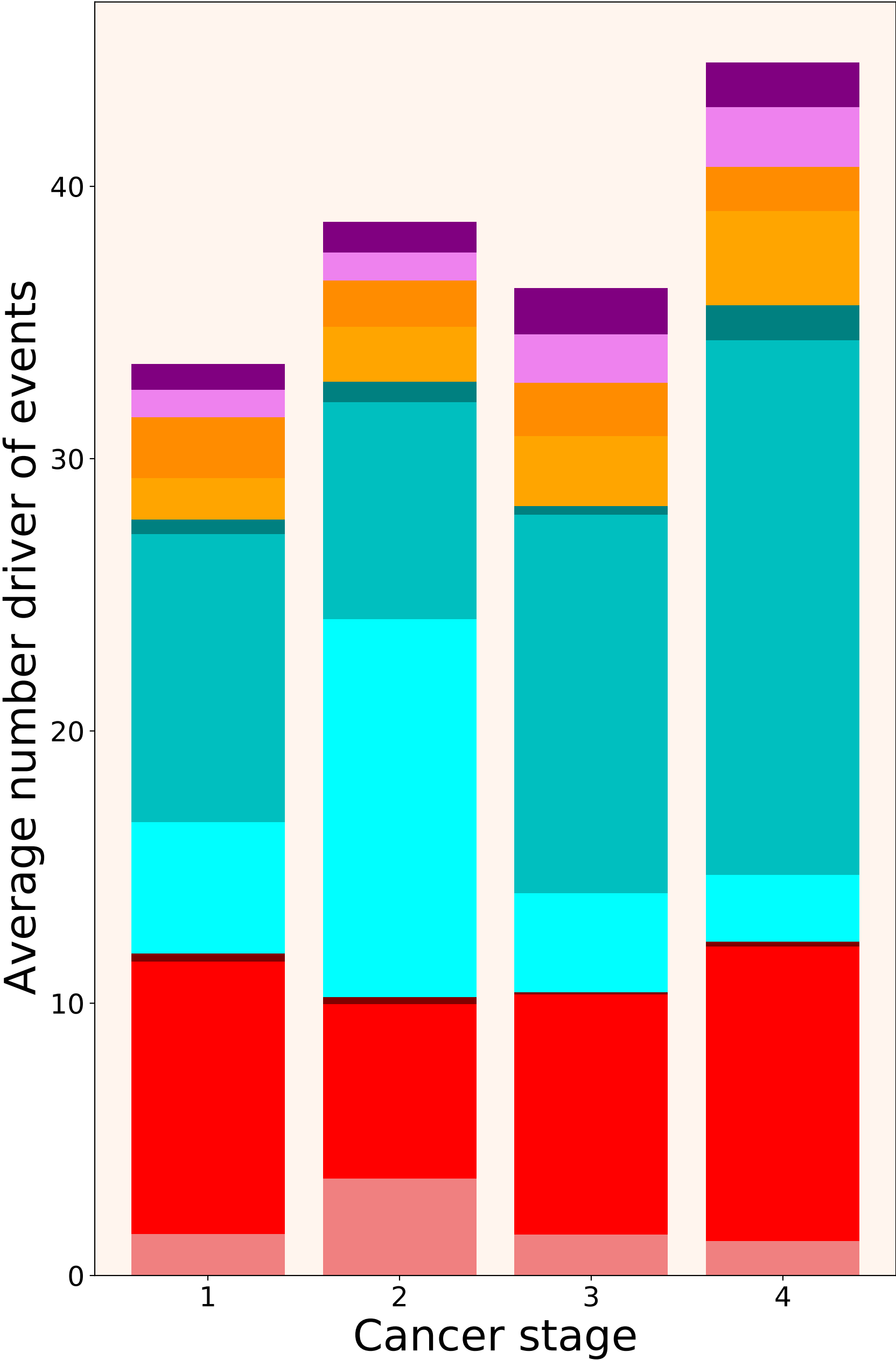

Supplement: S2 Files — (ZIP) [file pgen.1009996.s002.zip › PANCAN/cumulative histograms/Distribution_stages_cohorts/2021_11_23_14_43_distribution_stages_females_COAD.pdf]

Driver event distribution by cancer stage in males LUAD

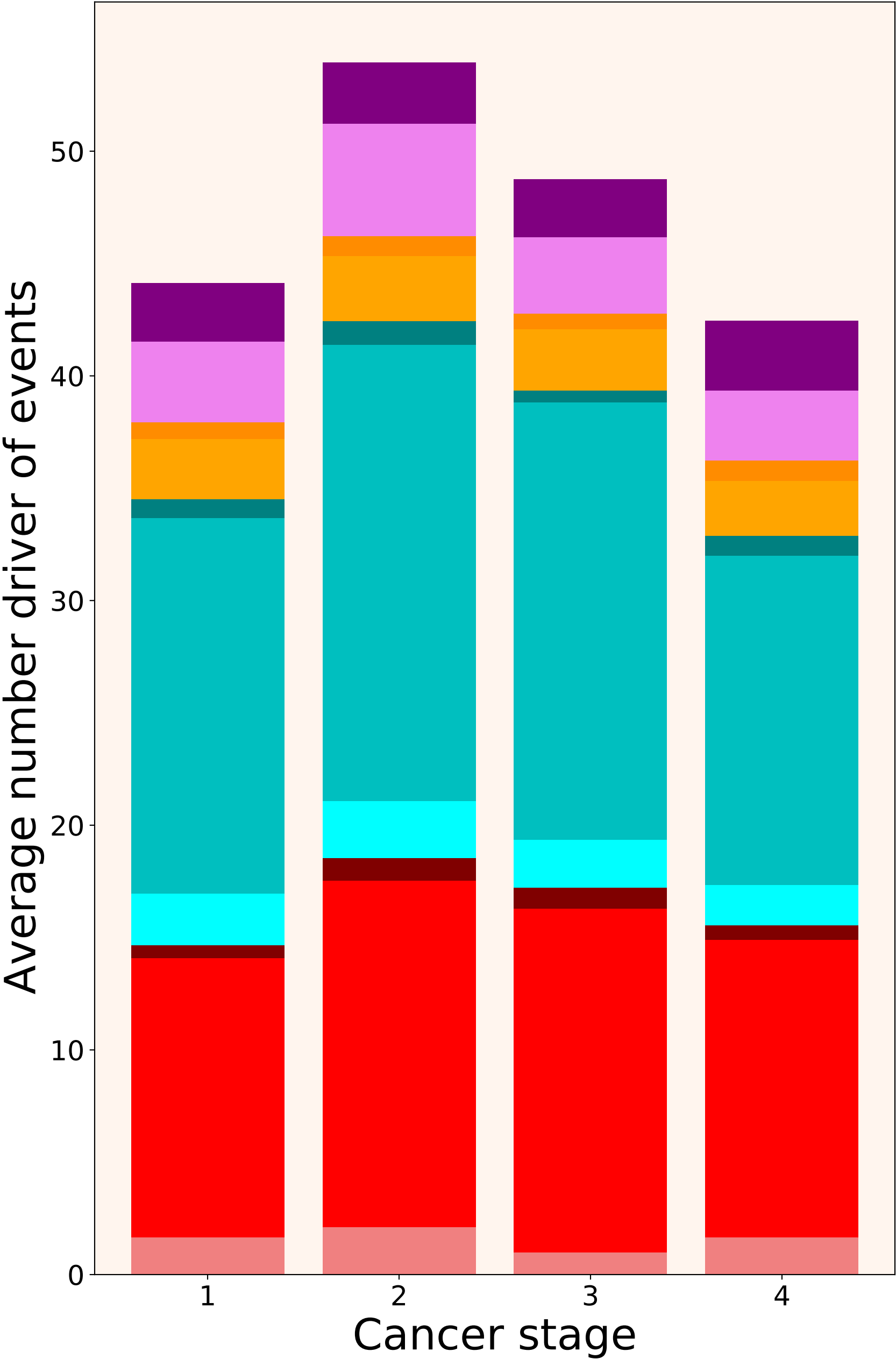

Supplement: S2 Files — (ZIP) [file pgen.1009996.s002.zip › PANCAN/cumulative histograms/Distribution_stages_cohorts/2021_11_23_14_43_distribution_stages_males_LUAD.pdf]

Driver event distribution by cancer stage READ

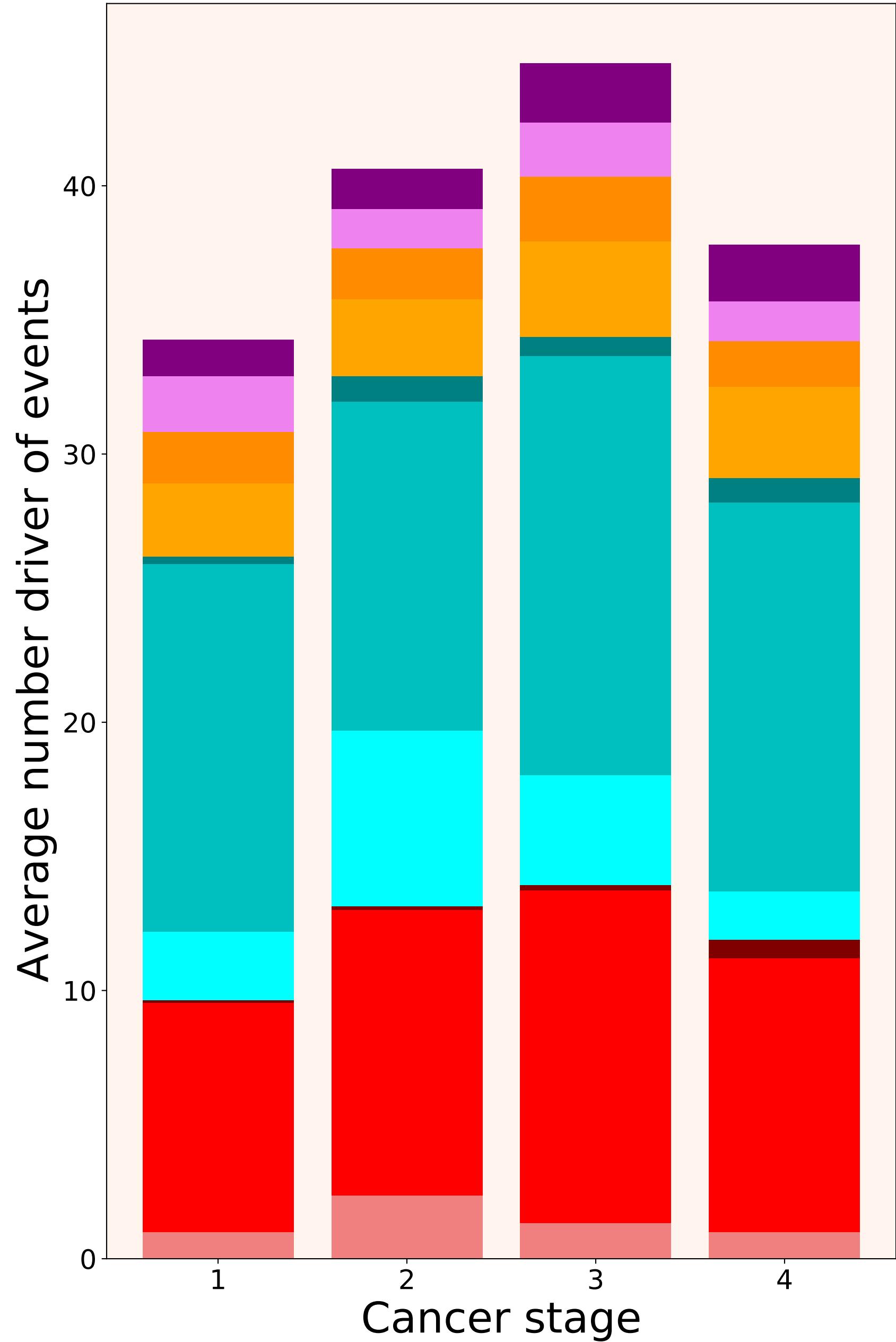

Supplement: S2 Files — (ZIP) [file pgen.1009996.s002.zip › PANCAN/cumulative histograms/Distribution_stages_cohorts/2021_11_23_14_43_distribution_stages_READ.pdf]

Driver event distribution by cancer stage in females KIRC

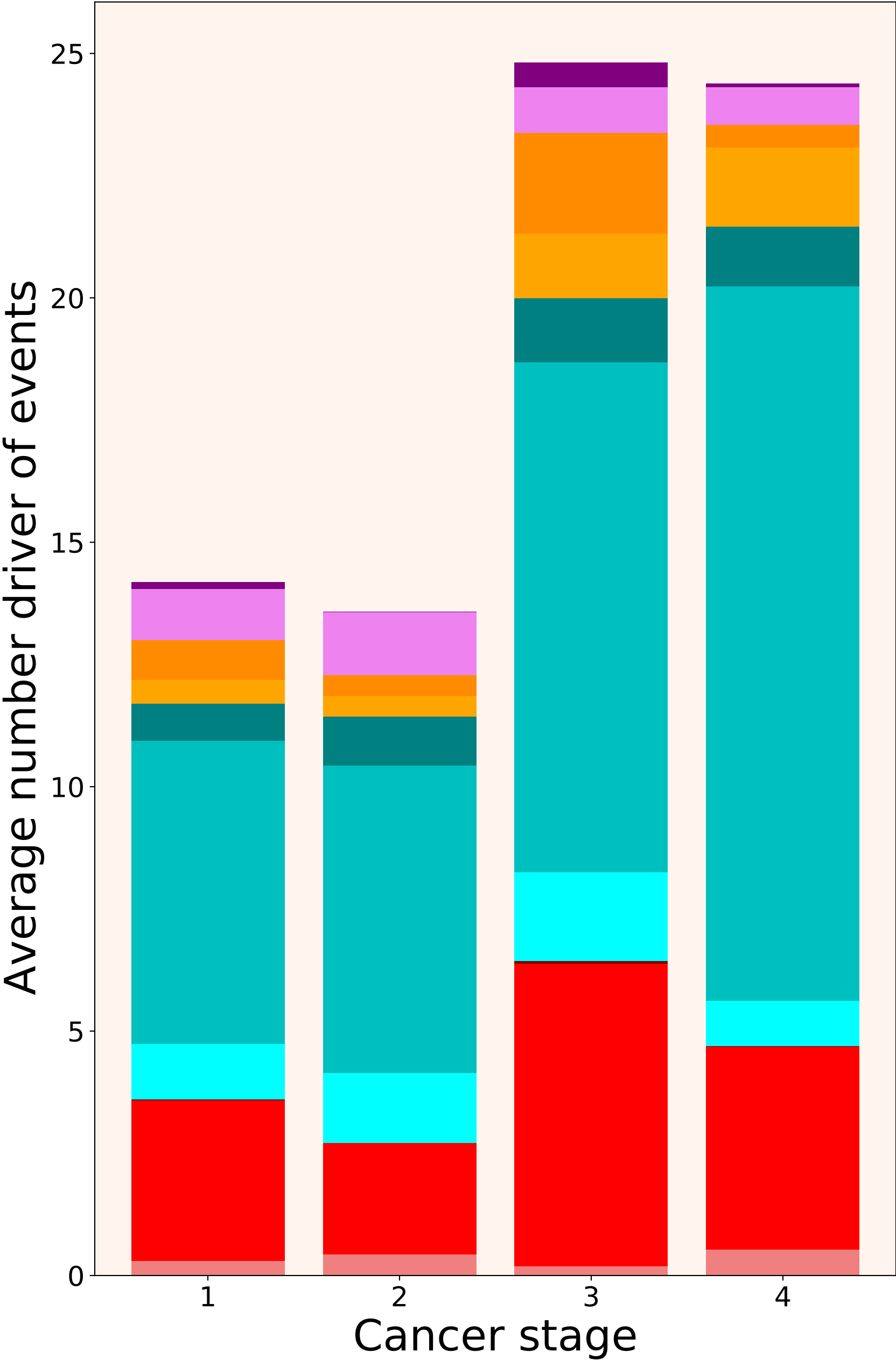

Supplement: S2 Files — (ZIP) [file pgen.1009996.s002.zip › PANCAN/cumulative histograms/Distribution_stages_cohorts/2021_11_23_14_43_distribution_stages_females_KIRC.pdf]

Driver event distribution by cancer stage BLCA

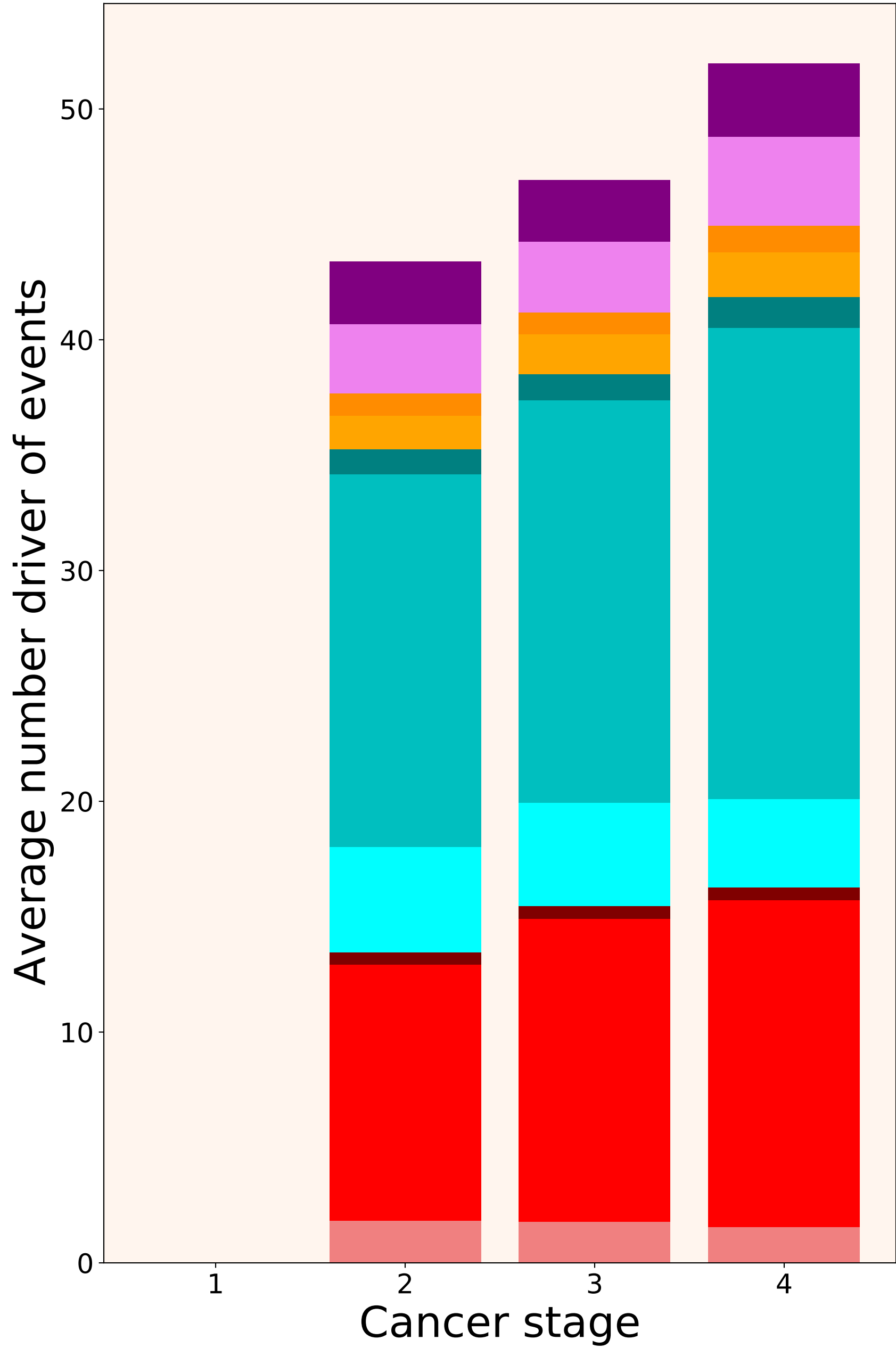

Supplement: S2 Files — (ZIP) [file pgen.1009996.s002.zip › PANCAN/cumulative histograms/Distribution_stages_cohorts/2021_11_23_14_43_distribution_stages_BLCA.pdf]

Driver event distribution by cancer stage PAAD

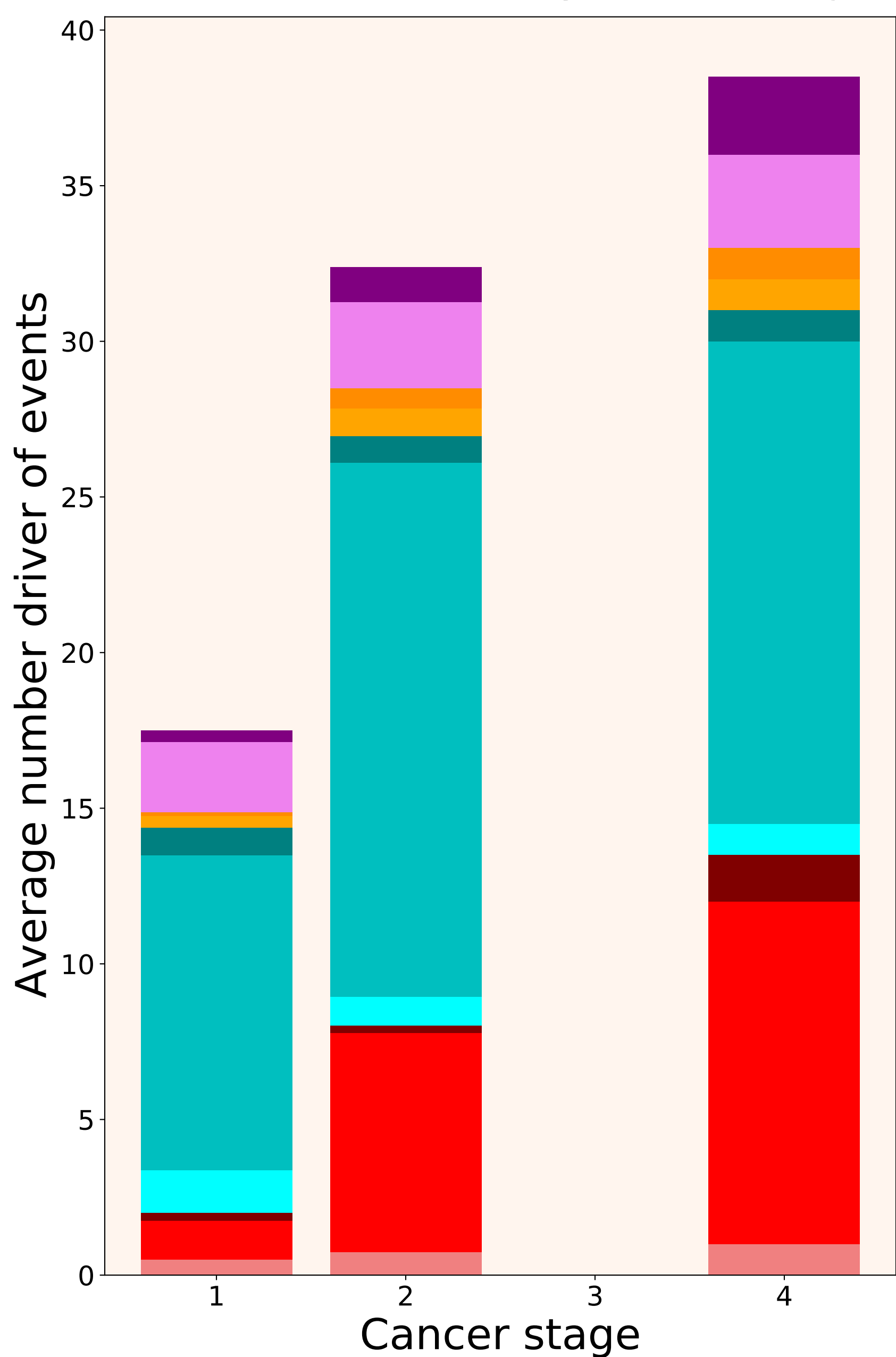

Supplement: S2 Files — (ZIP) [file pgen.1009996.s002.zip › PANCAN/cumulative histograms/Distribution_stages_cohorts/2021_11_23_14_43_distribution_stages_PAAD.pdf]

Driver event distribution by cancer stage THCA

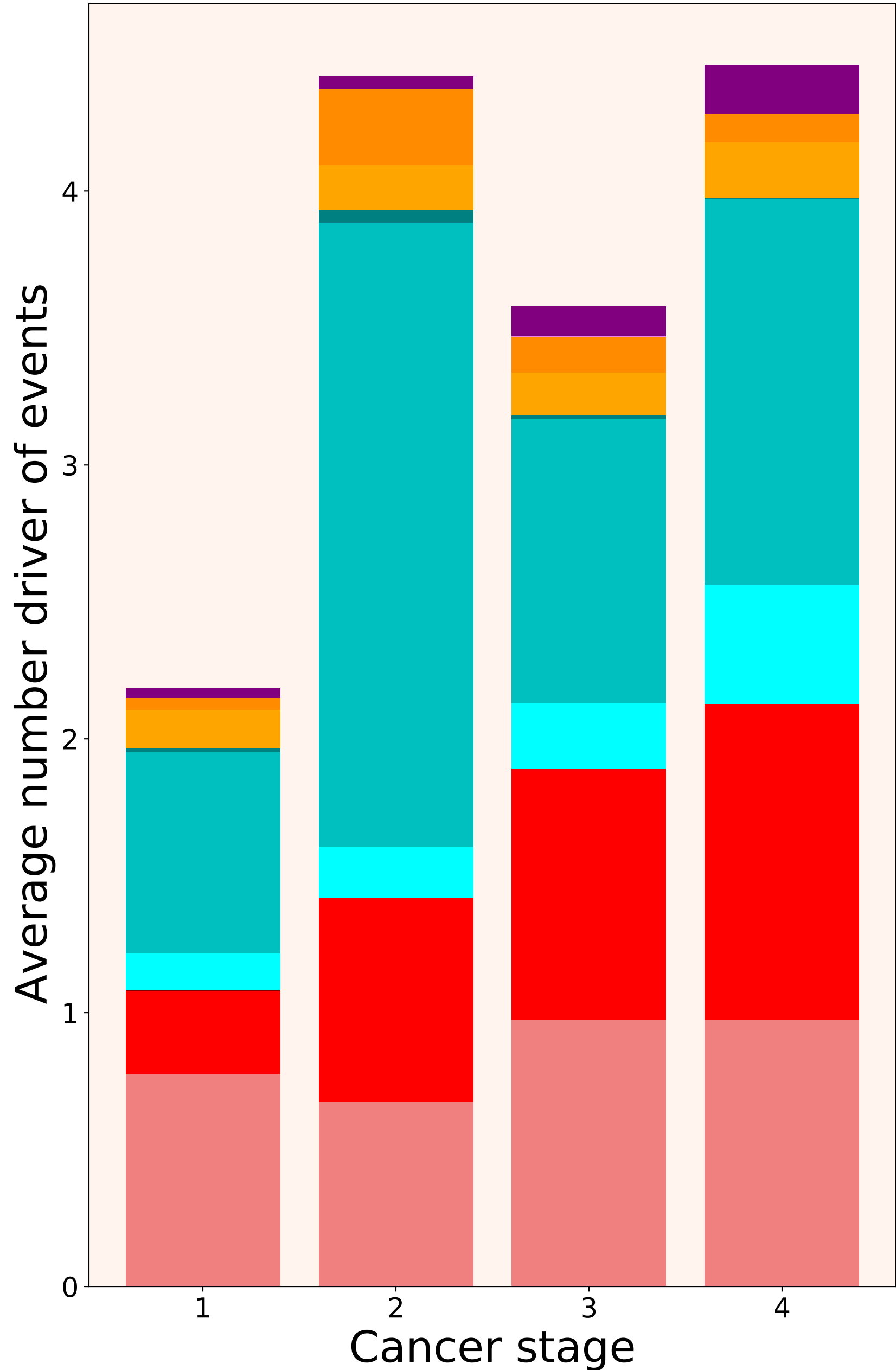

Supplement: S2 Files — (ZIP) [file pgen.1009996.s002.zip › PANCAN/cumulative histograms/Distribution_stages_cohorts/2021_11_23_14_43_distribution_stages_THCA.pdf]

Driver event distribution by cancer stage in females BLCA

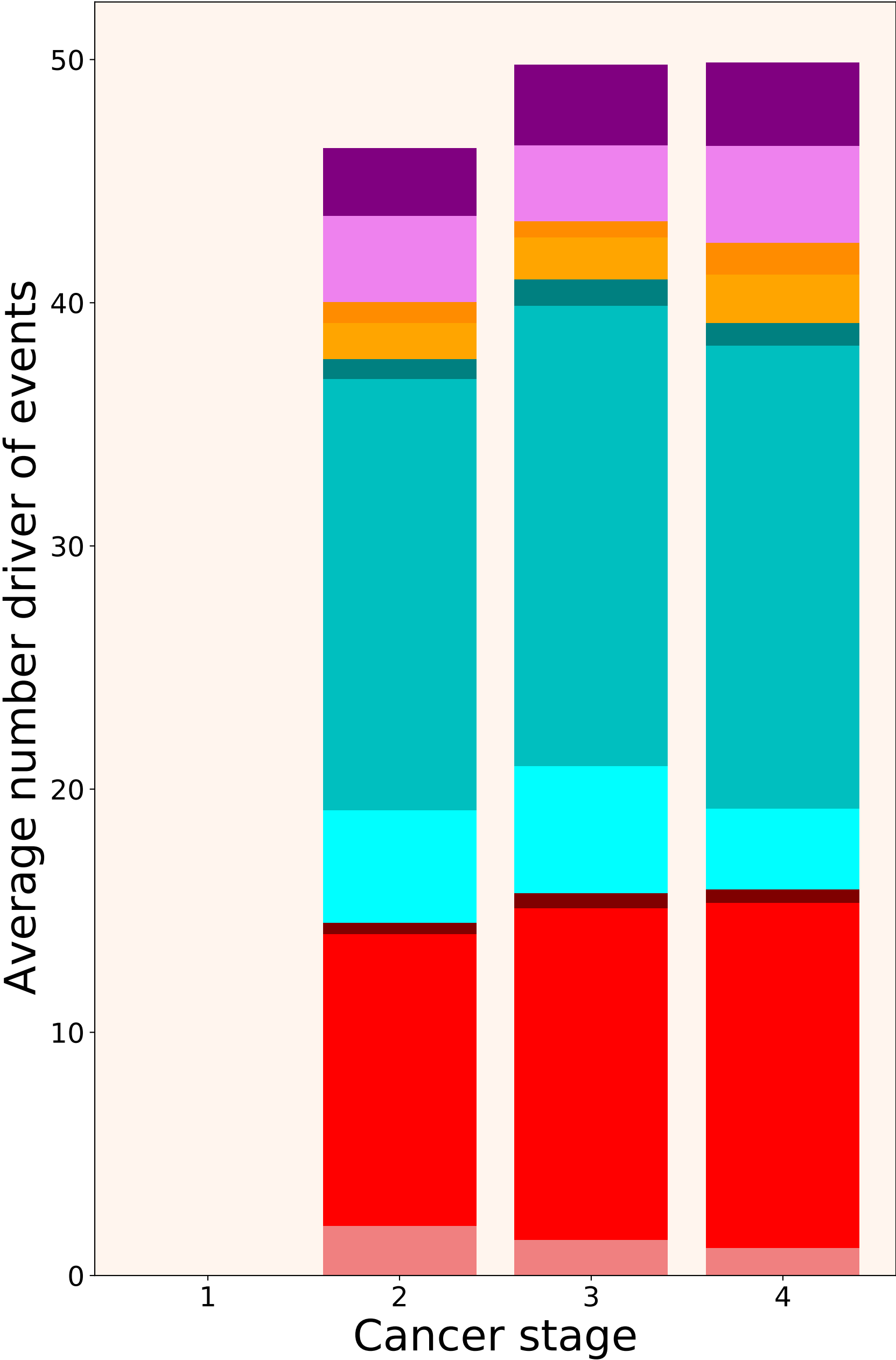

Supplement: S2 Files — (ZIP) [file pgen.1009996.s002.zip › PANCAN/cumulative histograms/Distribution_stages_cohorts/2021_11_23_14_43_distribution_stages_females_BLCA.pdf]

Driver event distribution by cancer stage in females THCA

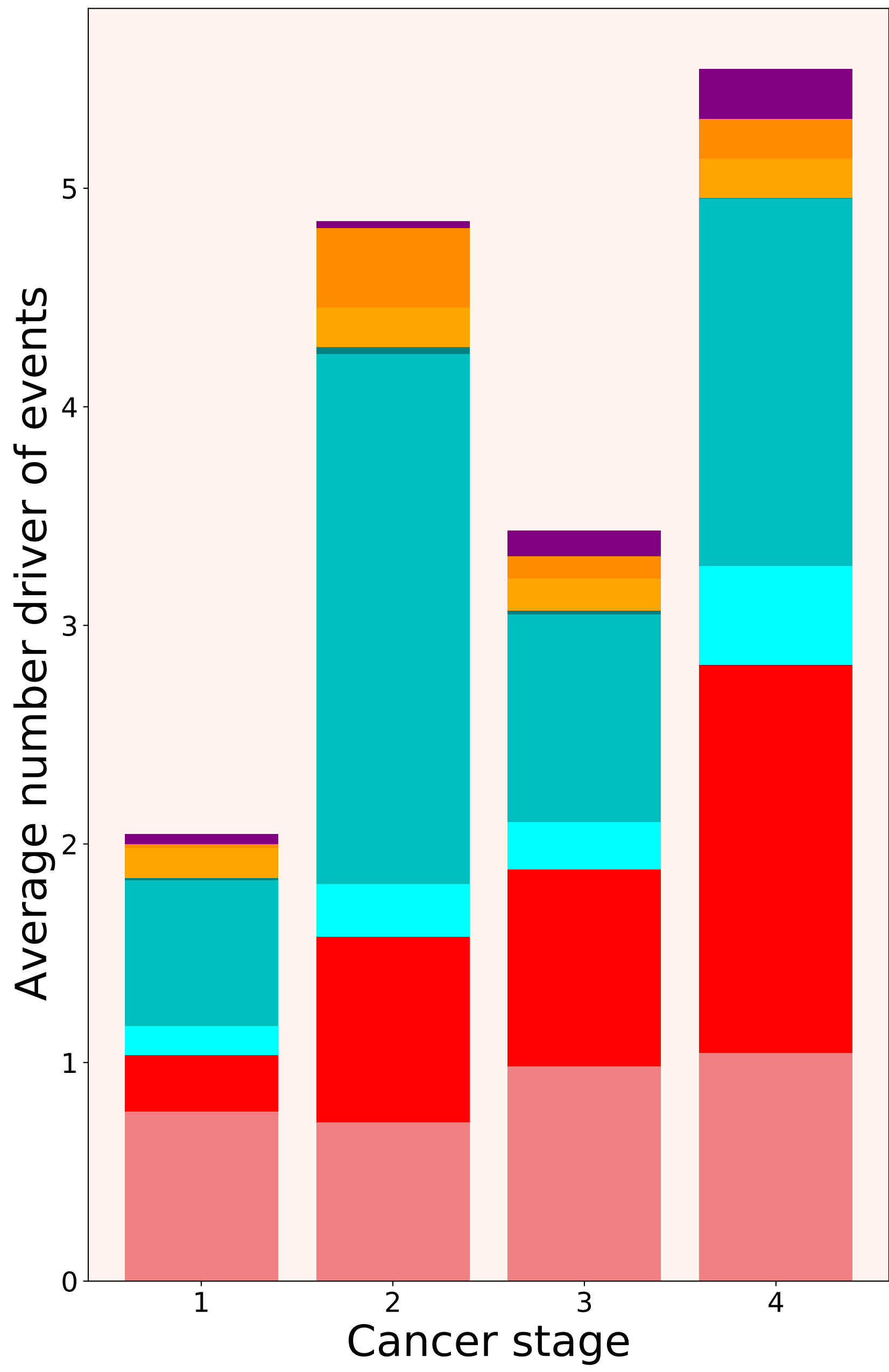

Supplement: S2 Files — (ZIP) [file pgen.1009996.s002.zip › PANCAN/cumulative histograms/Distribution_stages_cohorts/2021_11_23_14_43_distribution_stages_females_THCA.pdf]

Driver event distribution by cancer stage in females PAAD

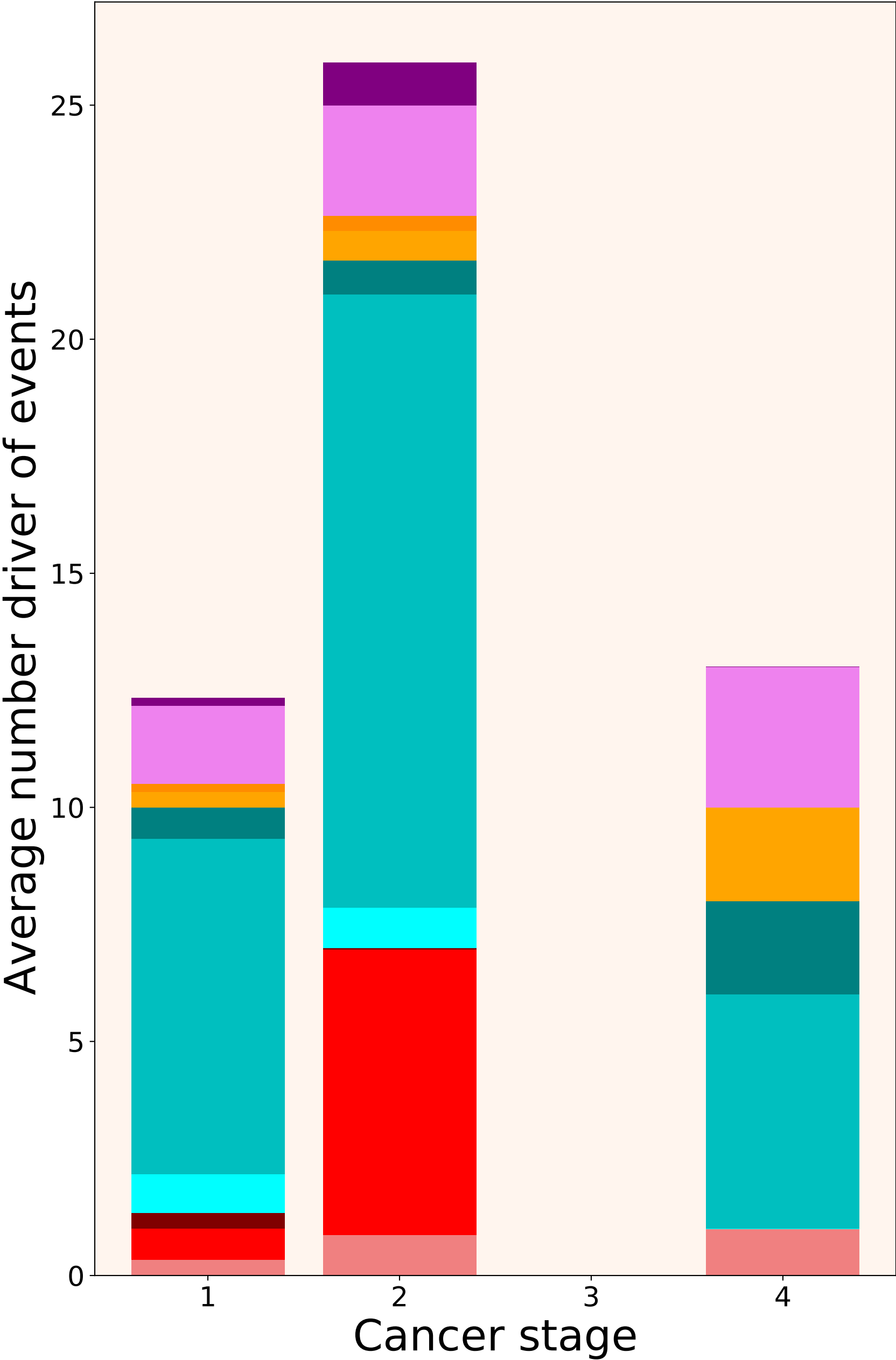

Supplement: S2 Files — (ZIP) [file pgen.1009996.s002.zip › PANCAN/cumulative histograms/Distribution_stages_cohorts/2021_11_23_14_43_distribution_stages_females_PAAD.pdf]

Driver event distribution by cancer stage in males ESCA

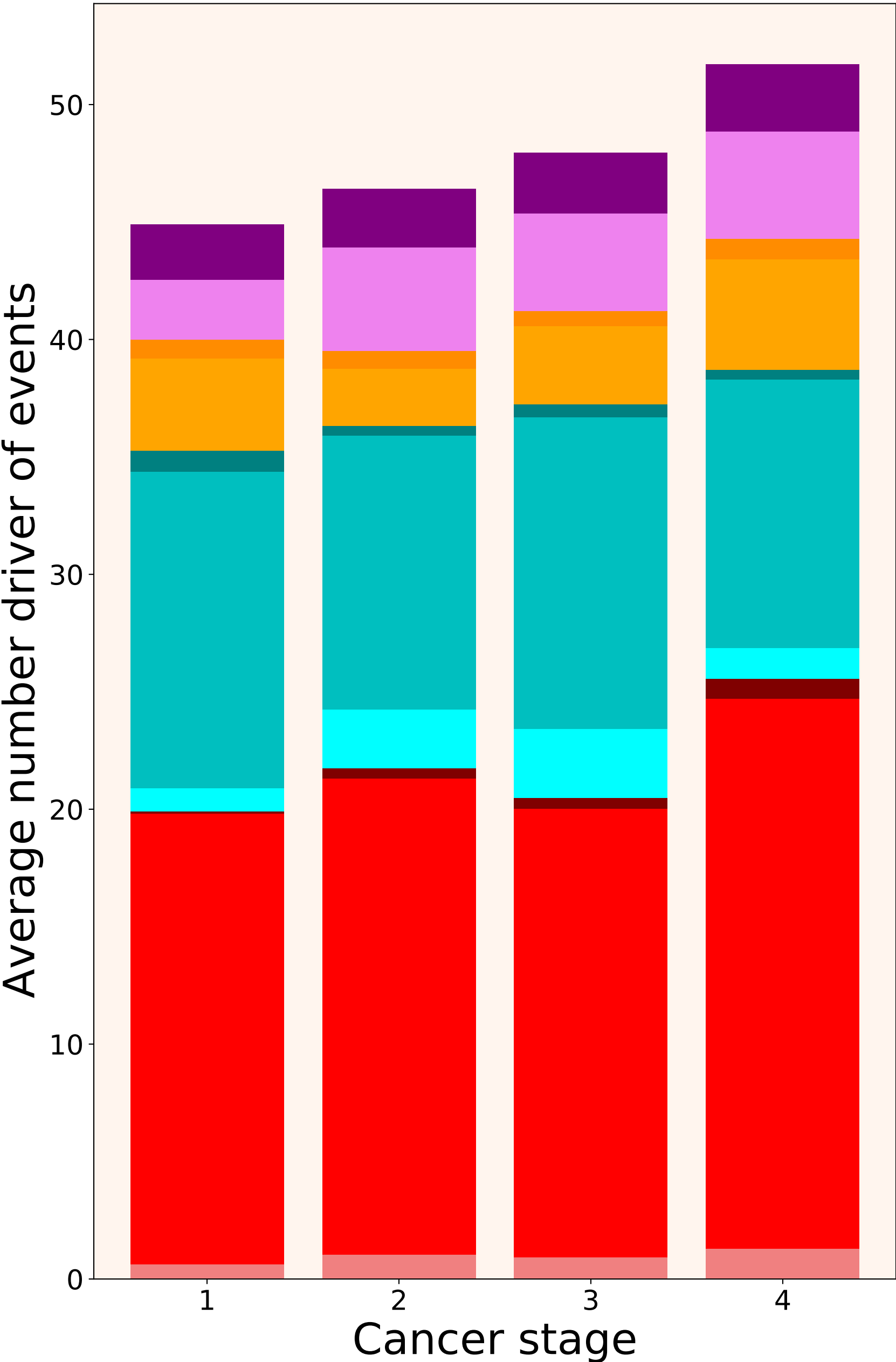

Supplement: S2 Files — (ZIP) [file pgen.1009996.s002.zip › PANCAN/cumulative histograms/Distribution_stages_cohorts/2021_11_23_14_43_distribution_stages_males_ESCA.pdf]

Driver event distribution by cancer stage KIRC

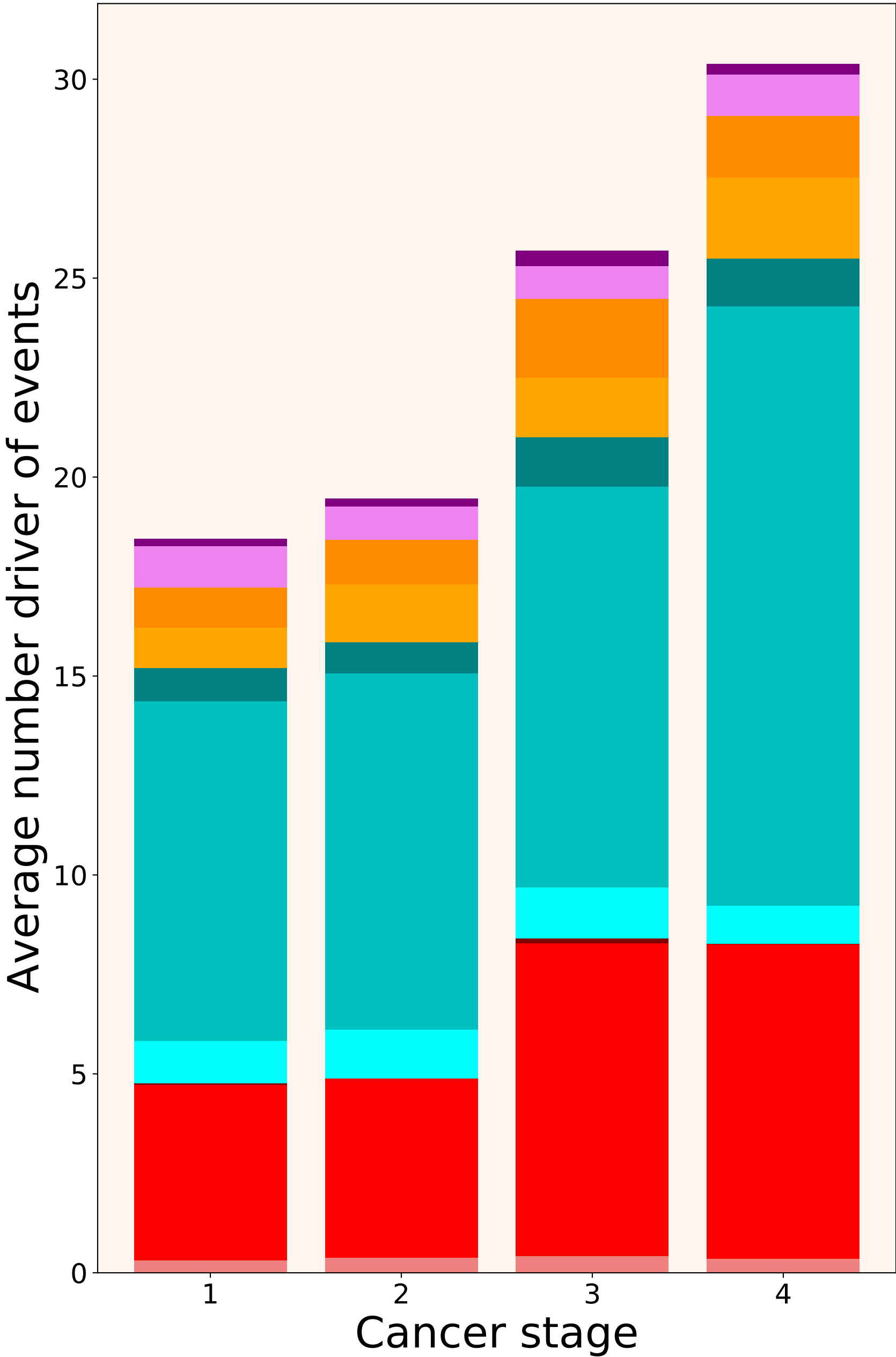

Supplement: S2 Files — (ZIP) [file pgen.1009996.s002.zip › PANCAN/cumulative histograms/Distribution_stages_cohorts/2021_11_23_14_43_distribution_stages_KIRC.pdf]

Driver event distribution by cancer stage in females READ

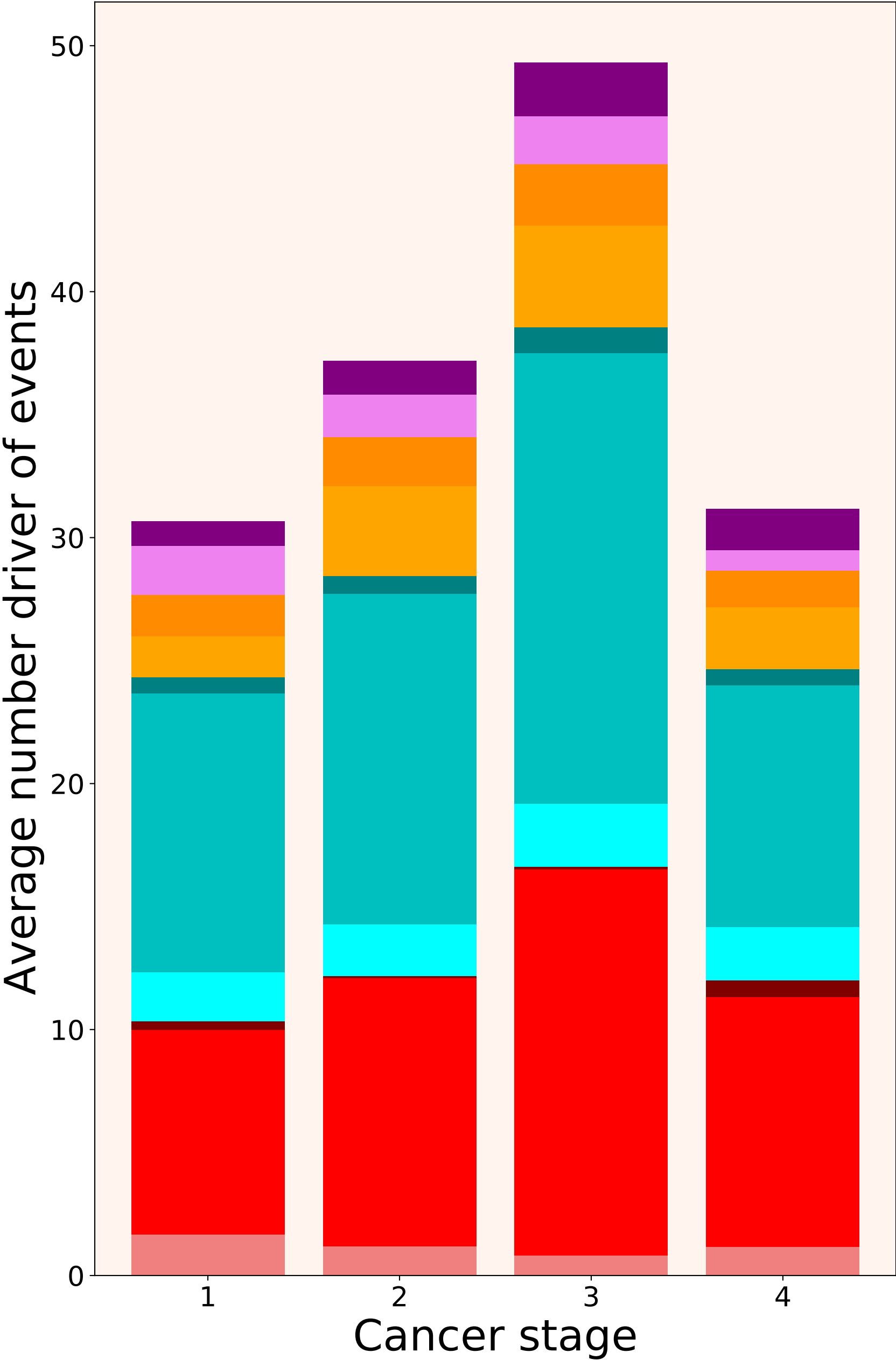

Supplement: S2 Files — (ZIP) [file pgen.1009996.s002.zip › PANCAN/cumulative histograms/Distribution_stages_cohorts/2021_11_23_14_43_distribution_stages_females_READ.pdf]

Driver event distribution by cancer stage in females UCS

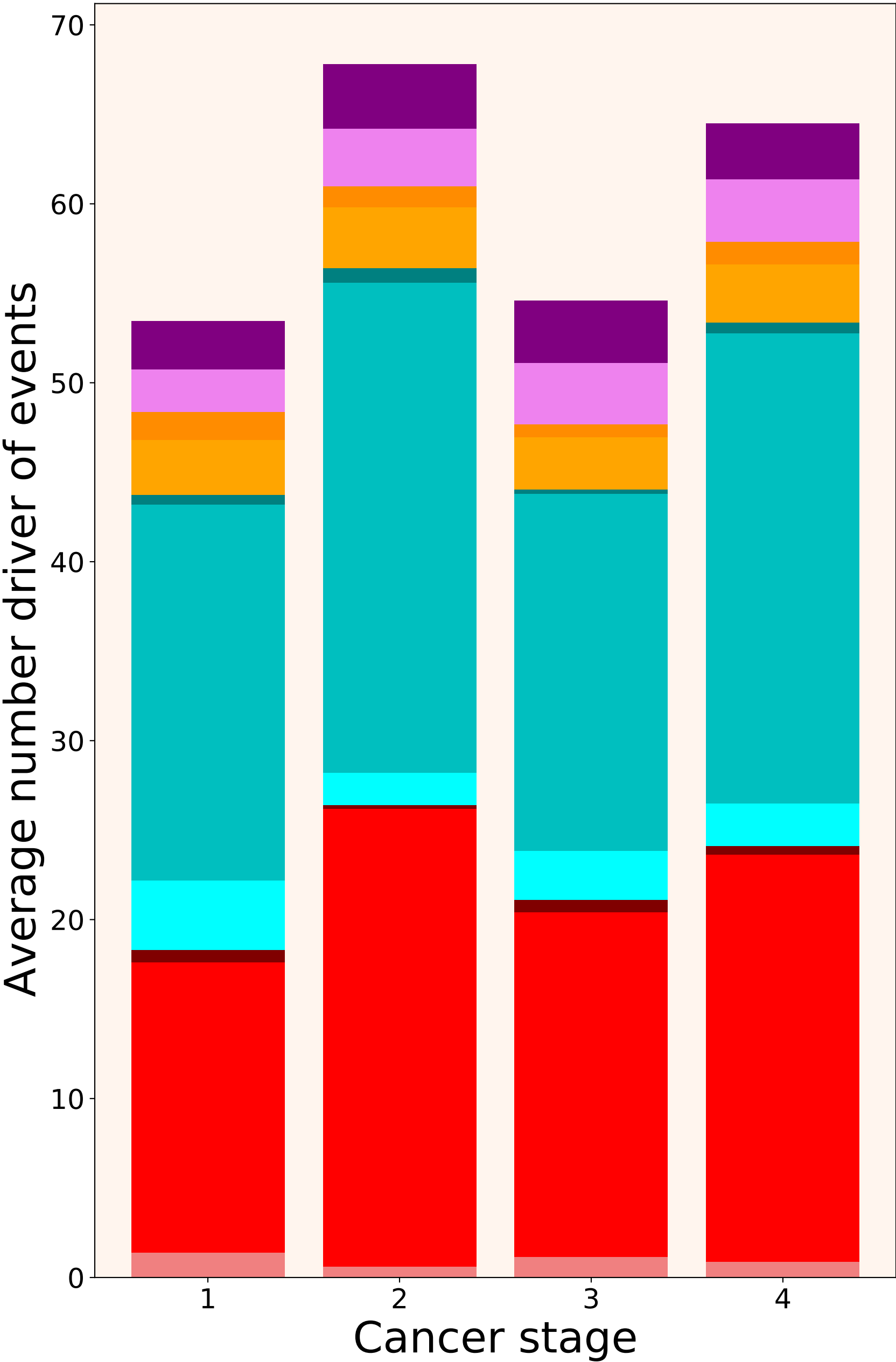

Supplement: S2 Files — (ZIP) [file pgen.1009996.s002.zip › PANCAN/cumulative histograms/Distribution_stages_cohorts/2021_11_23_14_43_distribution_stages_females_UCS.pdf]

Driver event distribution by cancer stage in males HNSC

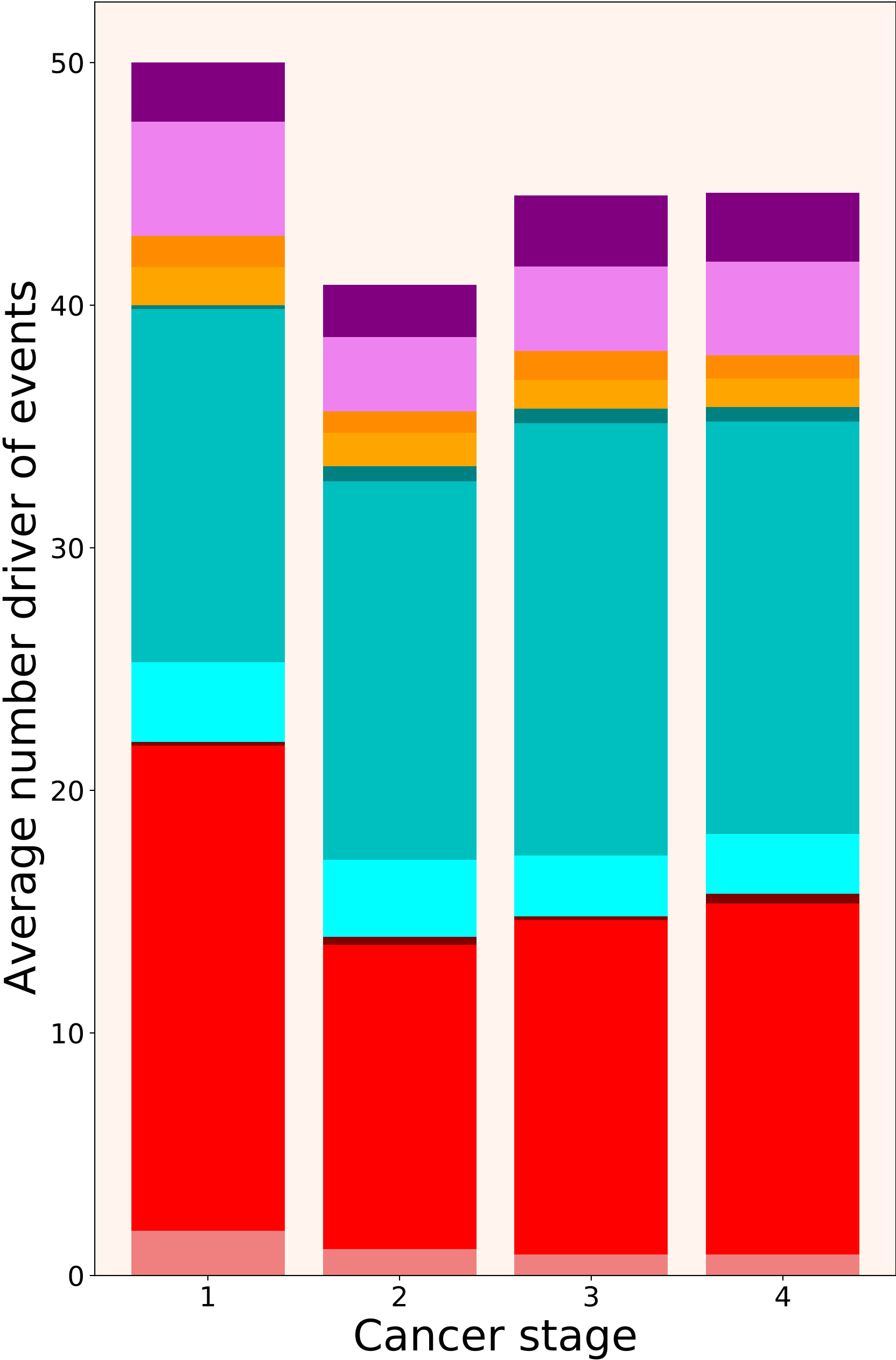

Supplement: S2 Files — (ZIP) [file pgen.1009996.s002.zip › PANCAN/cumulative histograms/Distribution_stages_cohorts/2021_11_23_14_43_distribution_stages_males_HNSC.pdf]

Driver event distribution by cancer stage in females HNSC

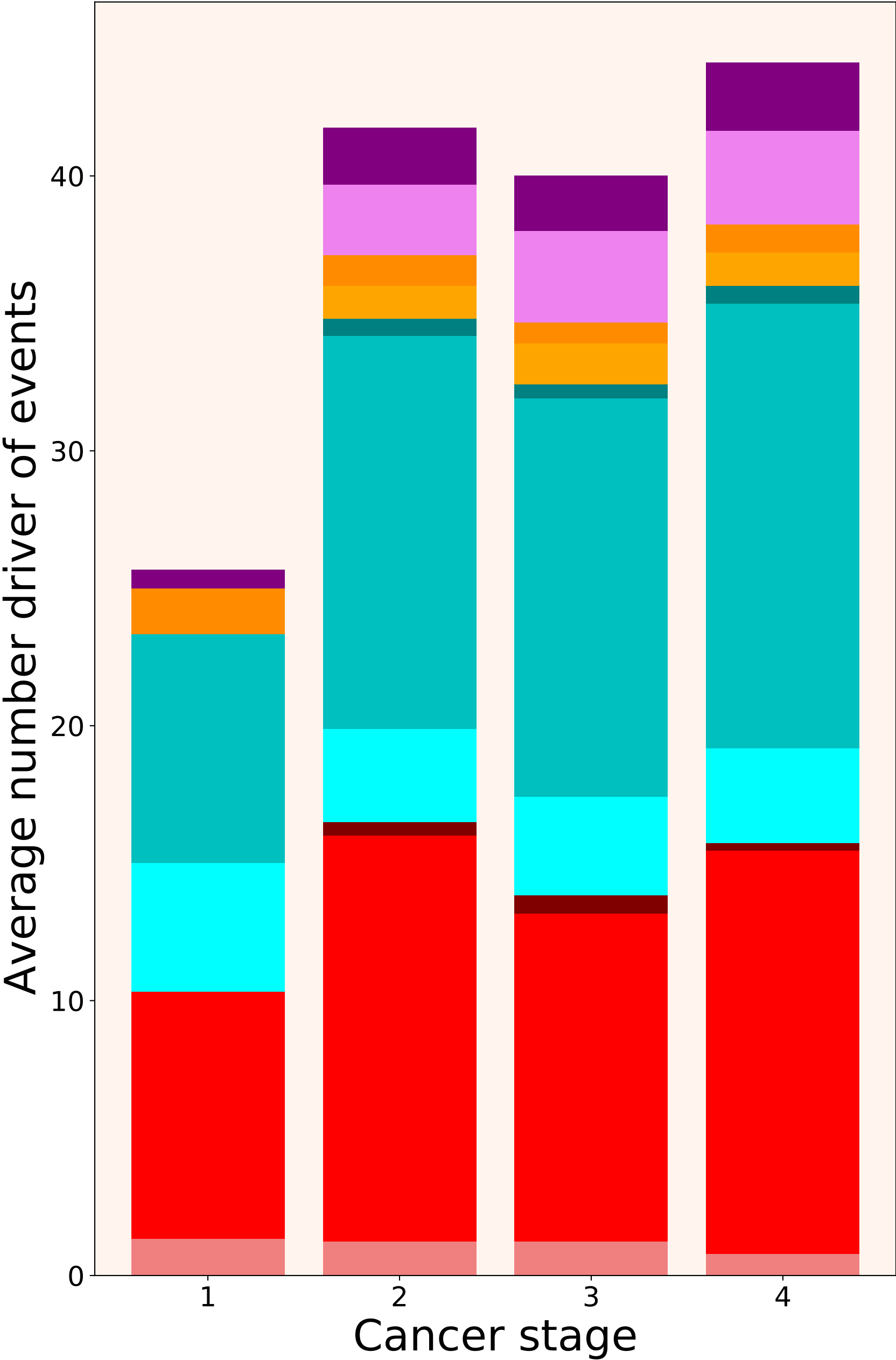

Supplement: S2 Files — (ZIP) [file pgen.1009996.s002.zip › PANCAN/cumulative histograms/Distribution_stages_cohorts/2021_11_23_14_43_distribution_stages_females_HNSC.pdf]

Driver event distribution by cancer stage in males READ

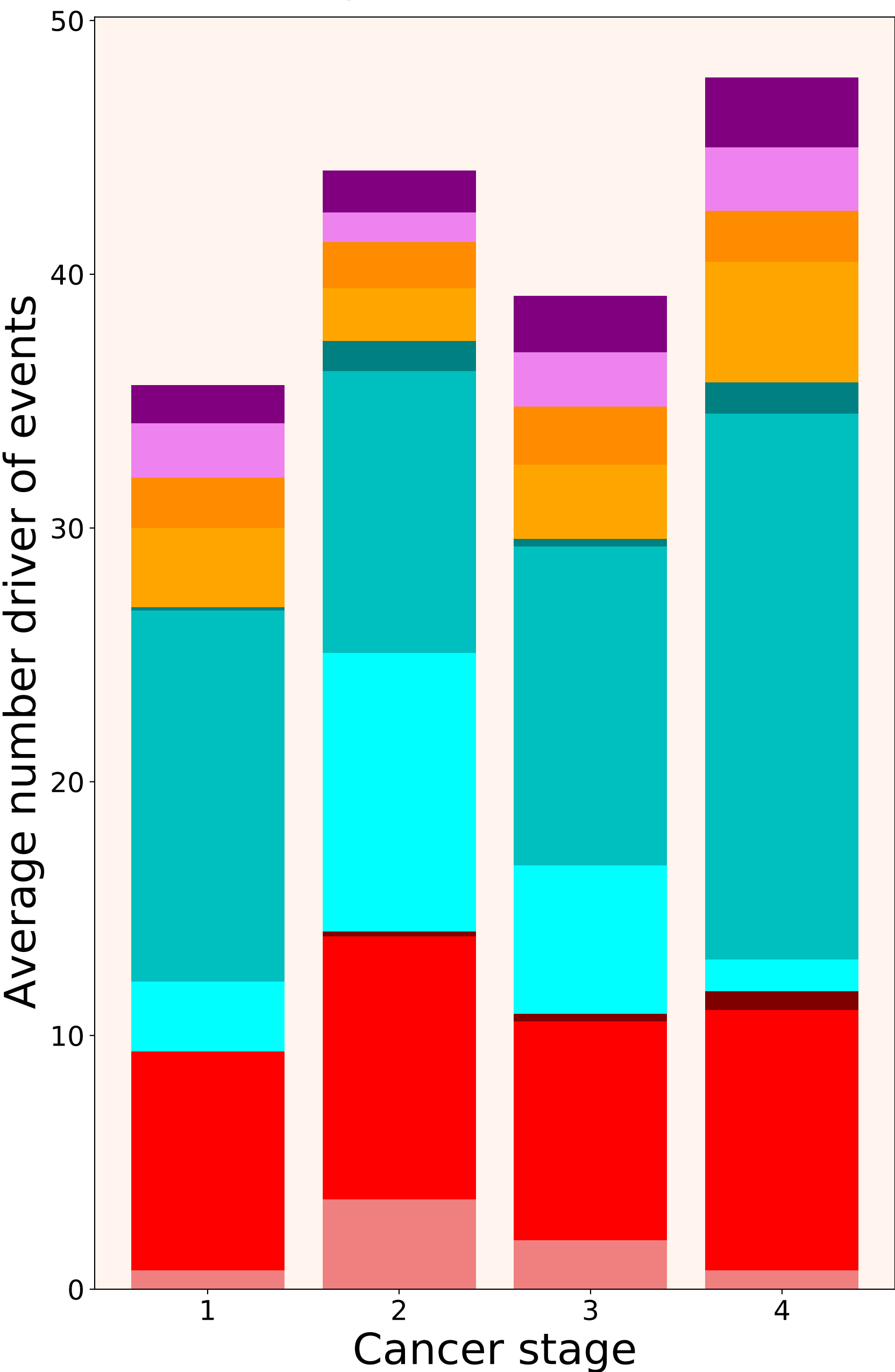

Supplement: S2 Files — (ZIP) [file pgen.1009996.s002.zip › PANCAN/cumulative histograms/Distribution_stages_cohorts/2021_11_23_14_43_distribution_stages_males_READ.pdf]

Driver event distribution by cancer stage UVM

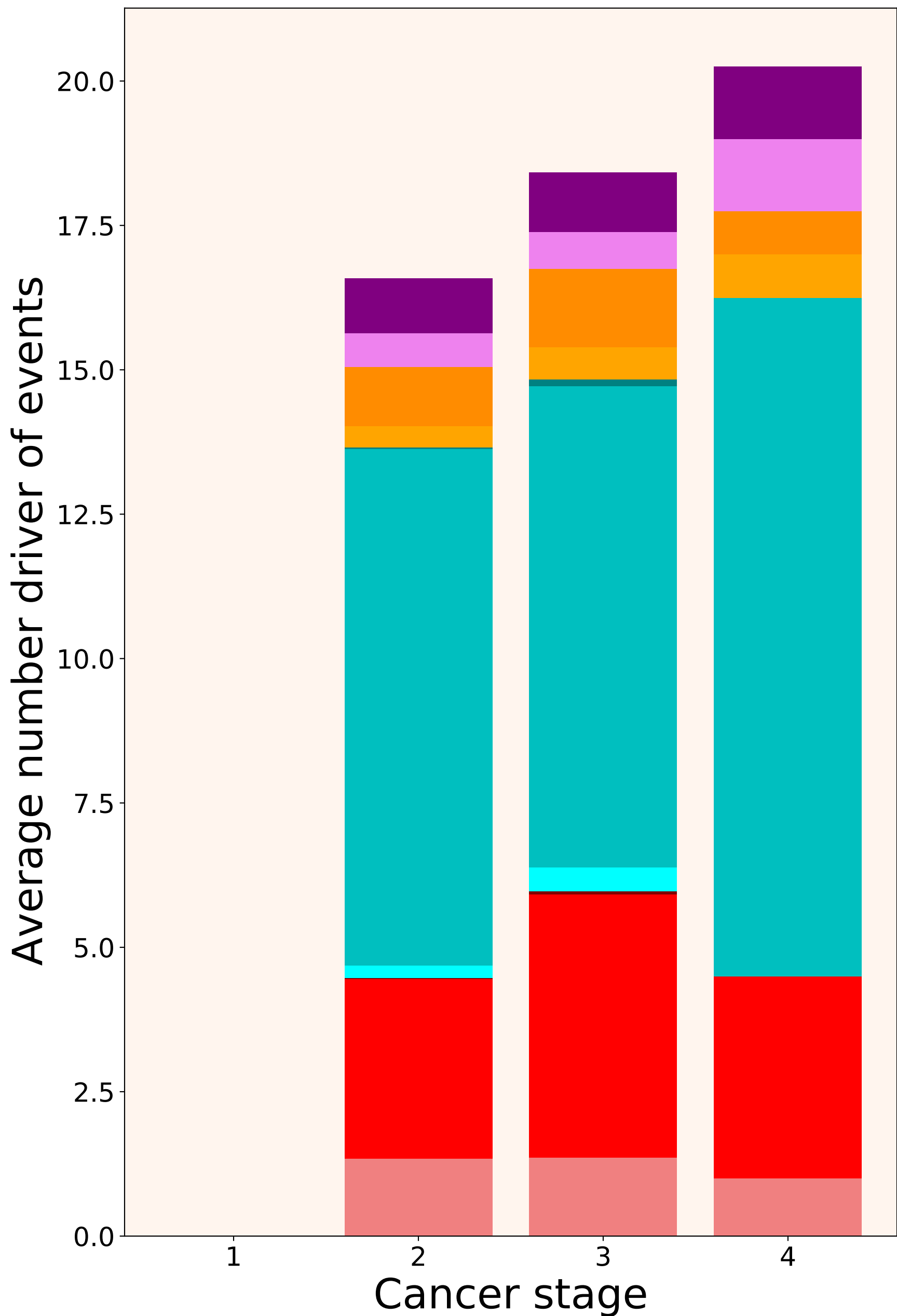

Supplement: S2 Files — (ZIP) [file pgen.1009996.s002.zip › PANCAN/cumulative histograms/Distribution_stages_cohorts/2021_11_23_14_43_distribution_stages_UVM.pdf]

Driver event distribution by cancer stage in males THCA

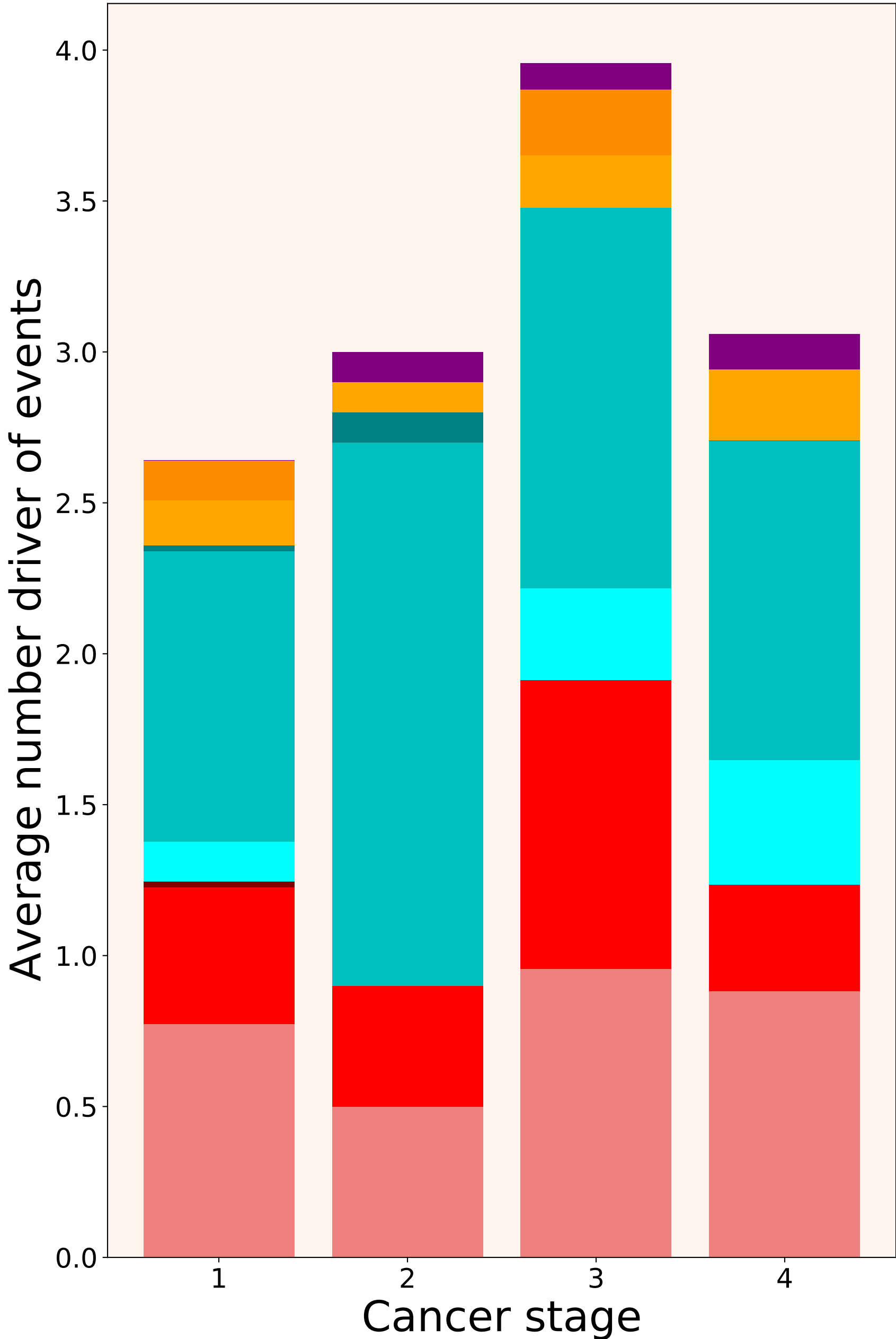

Supplement: S2 Files — (ZIP) [file pgen.1009996.s002.zip › PANCAN/cumulative histograms/Distribution_stages_cohorts/2021_11_23_14_43_distribution_stages_males_THCA.pdf]

Driver event distribution by cancer stage in males UVM

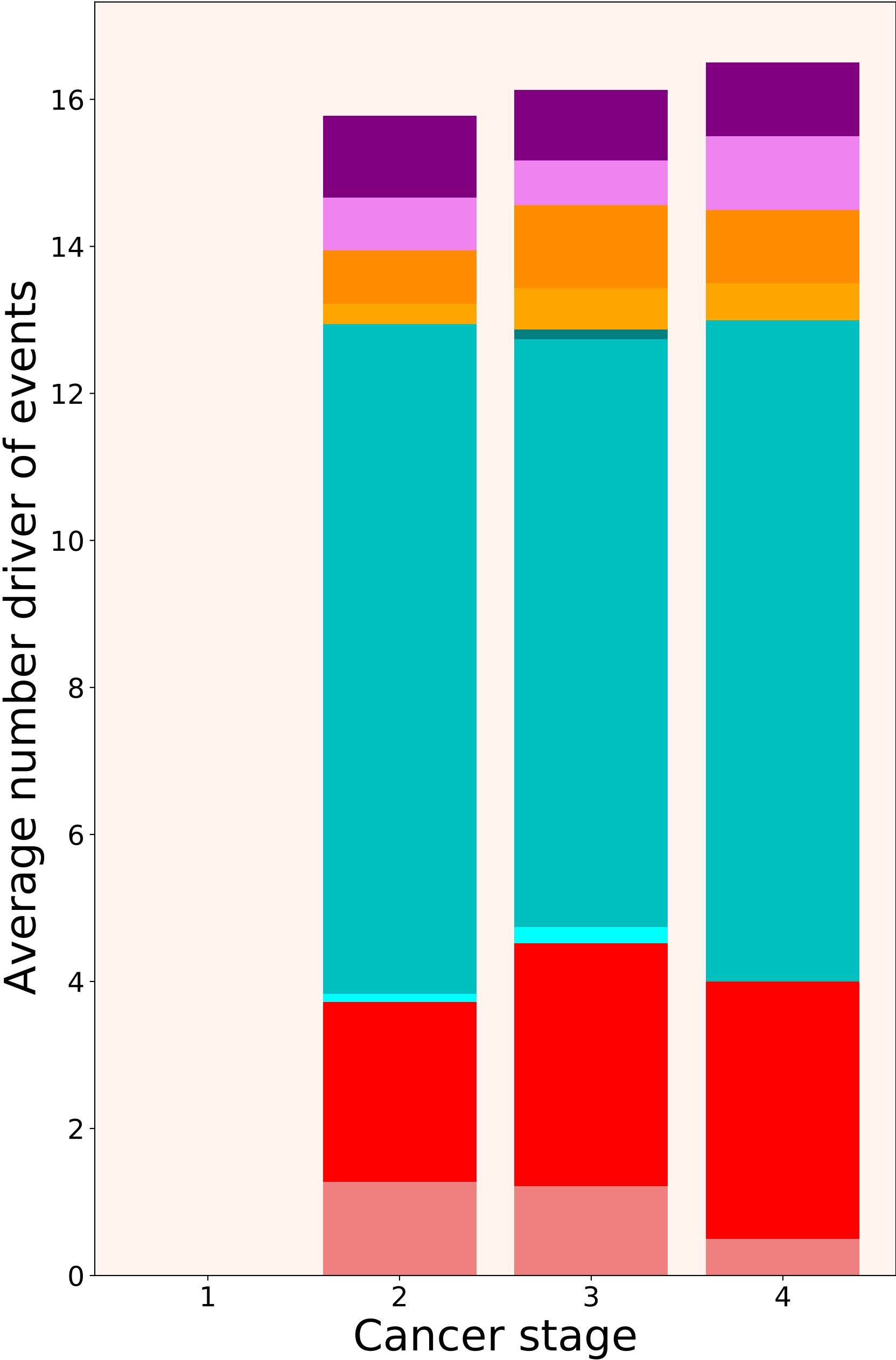

Supplement: S2 Files — (ZIP) [file pgen.1009996.s002.zip › PANCAN/cumulative histograms/Distribution_stages_cohorts/2021_11_23_14_43_distribution_stages_males_UVM.pdf]

Driver event distribution by cancer stage in males PAAD

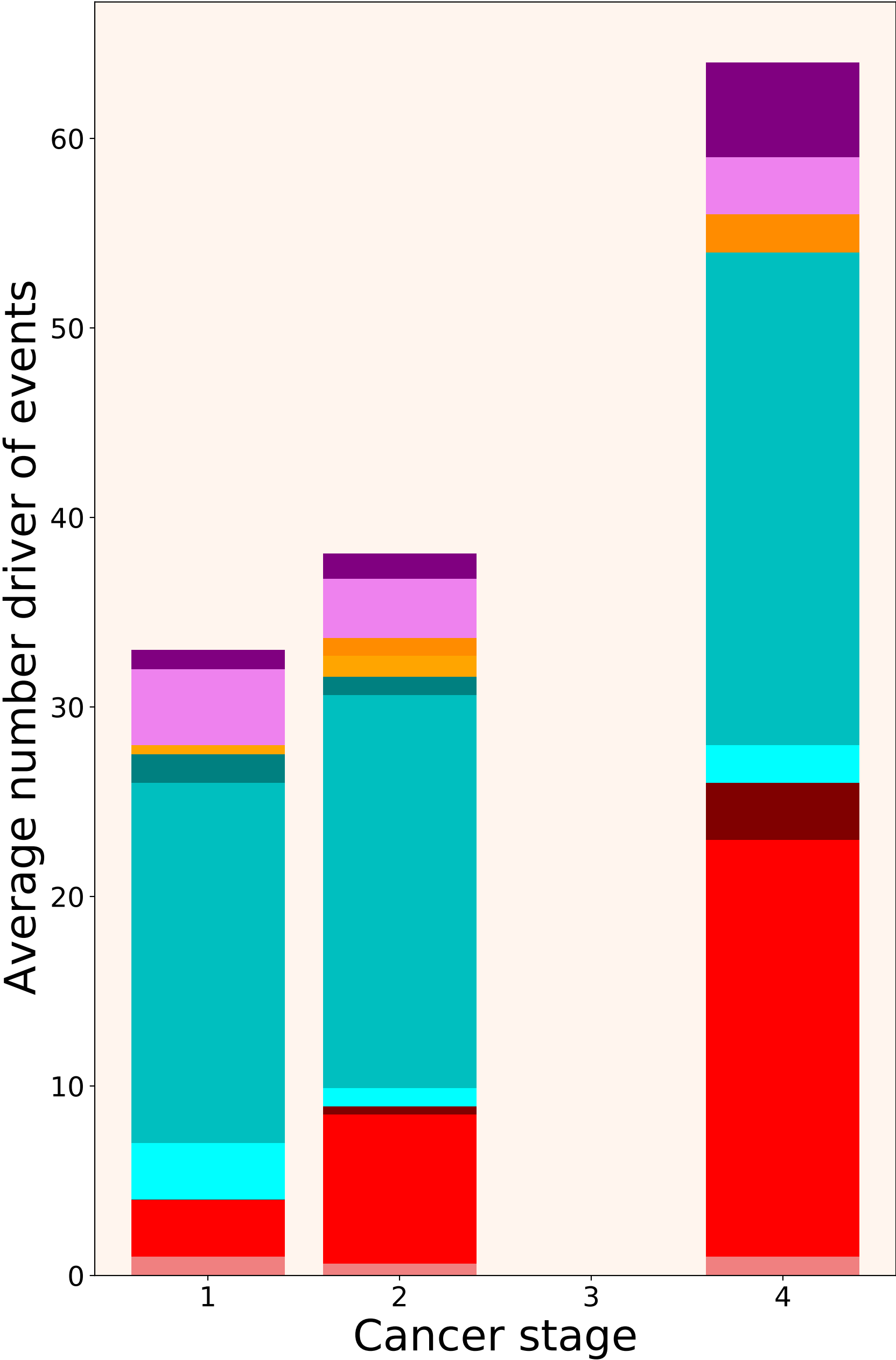

Supplement: S2 Files — (ZIP) [file pgen.1009996.s002.zip › PANCAN/cumulative histograms/Distribution_stages_cohorts/2021_11_23_14_43_distribution_stages_males_PAAD.pdf]

Driver event distribution by cancer stage in females ESCA

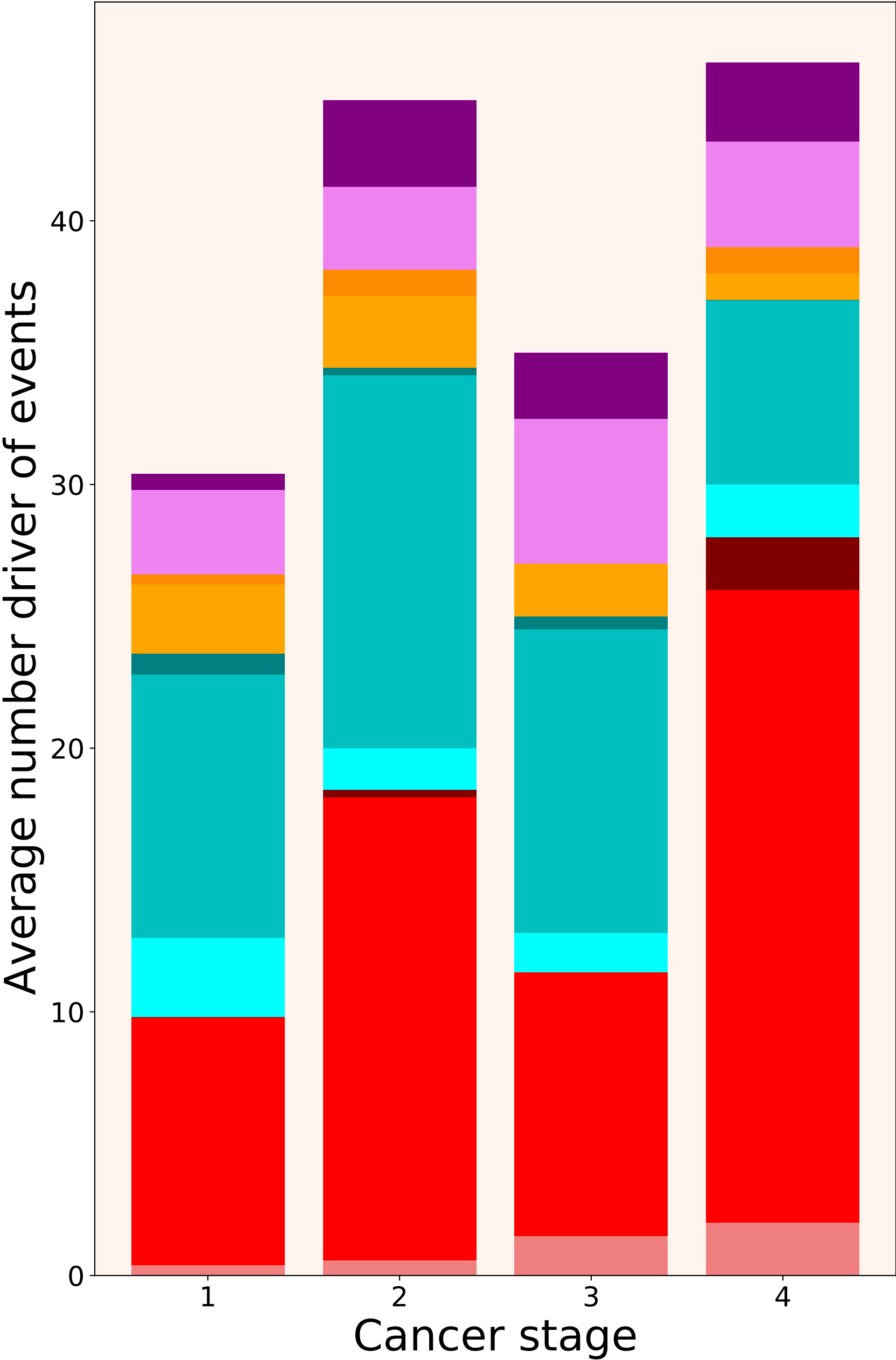

Supplement: S2 Files — (ZIP) [file pgen.1009996.s002.zip › PANCAN/cumulative histograms/Distribution_stages_cohorts/2021_11_23_14_43_distribution_stages_females_ESCA.pdf]

Driver event distribution by cancer stage in males BLCA

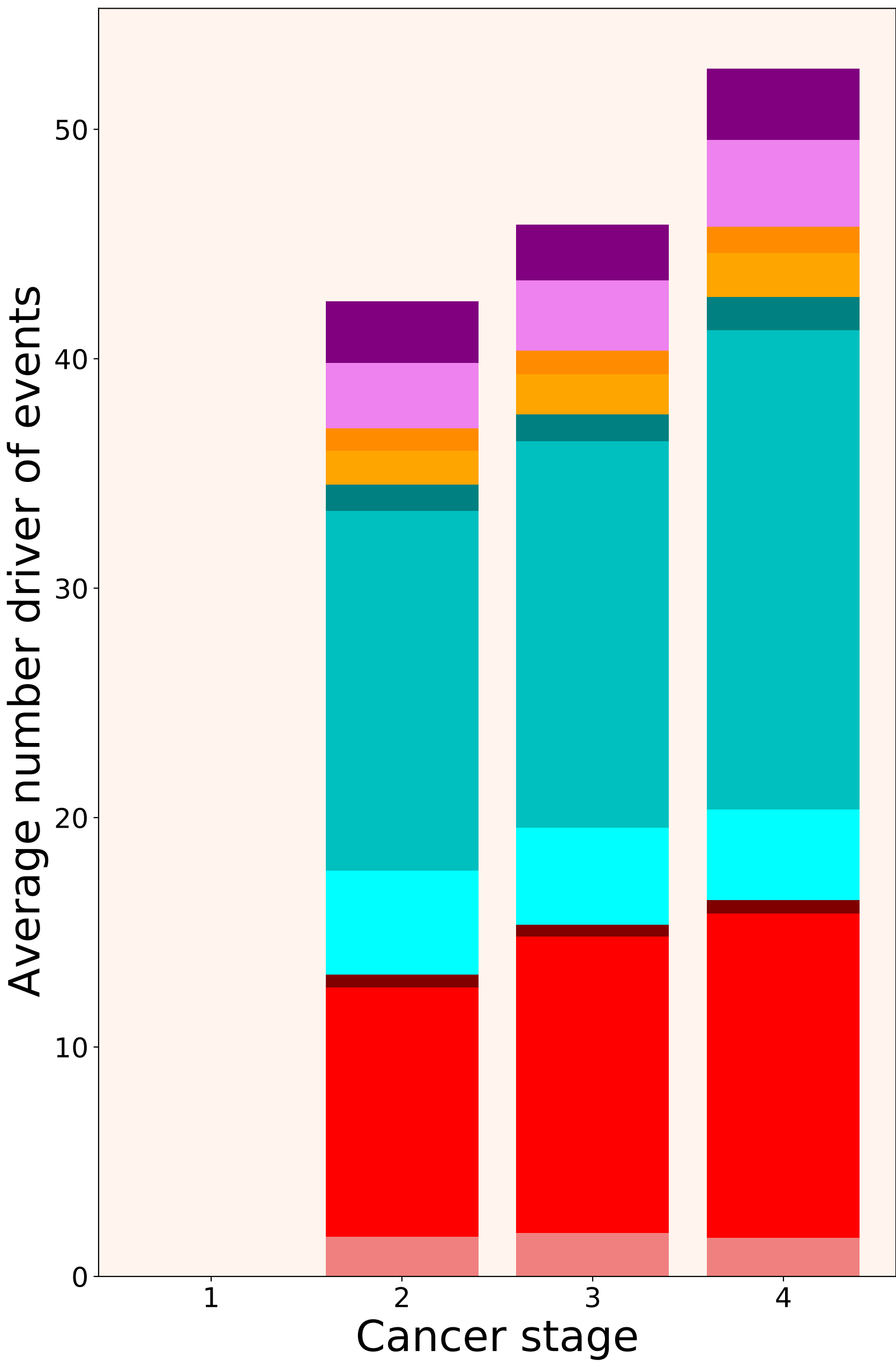

Supplement: S2 Files — (ZIP) [file pgen.1009996.s002.zip › PANCAN/cumulative histograms/Distribution_stages_cohorts/2021_11_23_14_43_distribution_stages_males_BLCA.pdf]

Driver event distribution by cancer stage ESCA

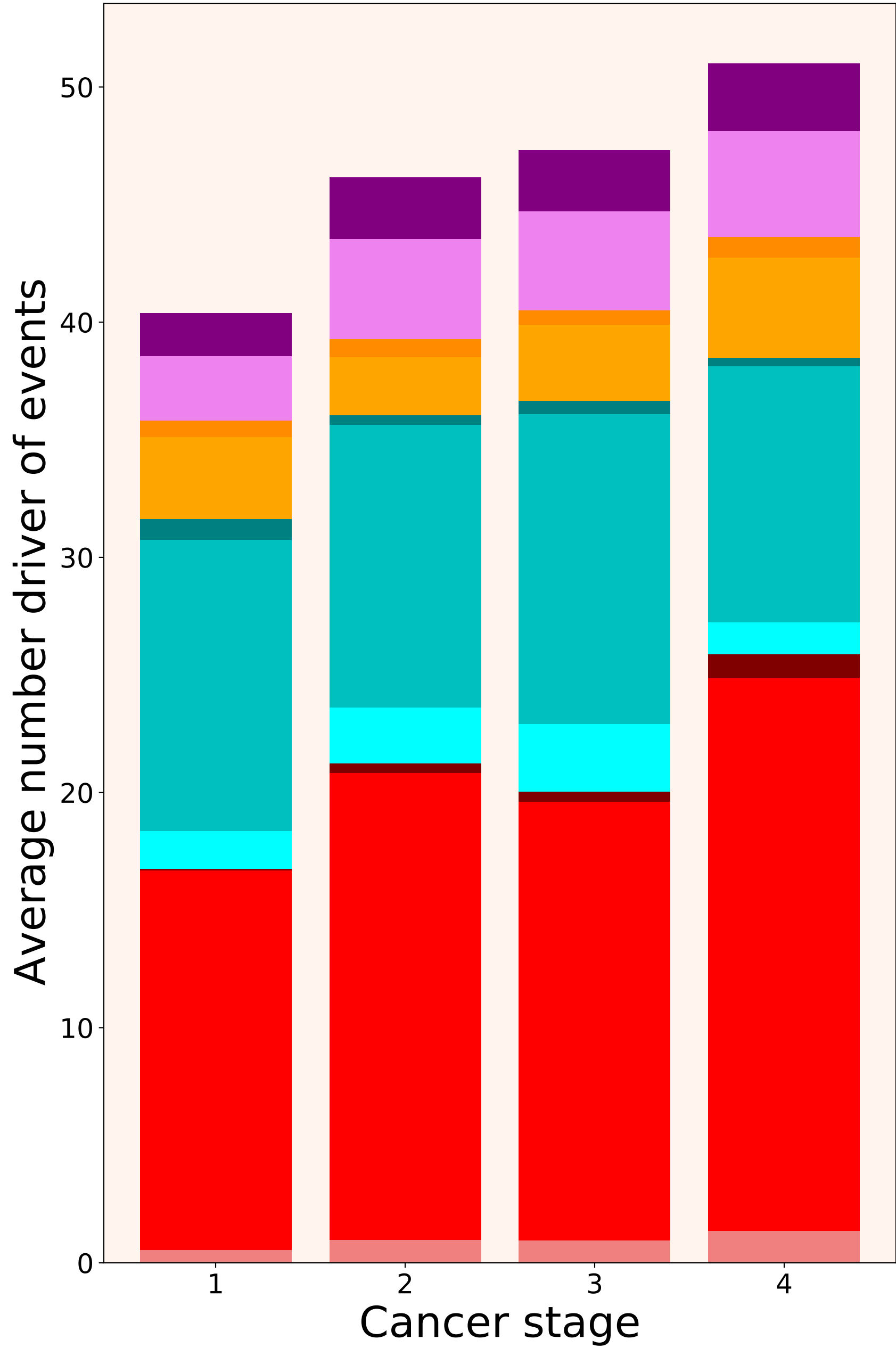

Supplement: S2 Files — (ZIP) [file pgen.1009996.s002.zip › PANCAN/cumulative histograms/Distribution_stages_cohorts/2021_11_23_14_43_distribution_stages_ESCA.pdf]

Driver event distribution by cancer stage HNSC

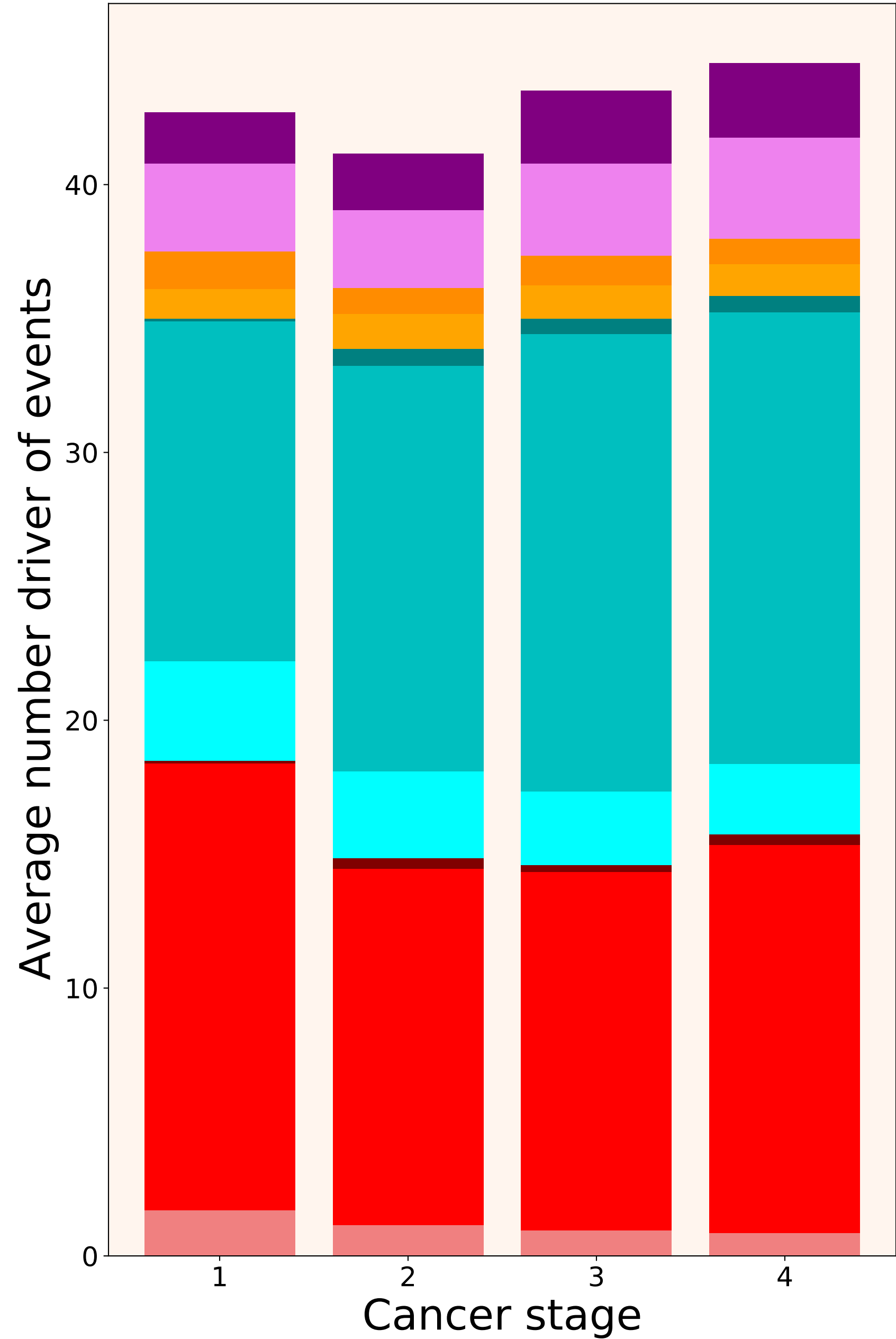

Supplement: S2 Files — (ZIP) [file pgen.1009996.s002.zip › PANCAN/cumulative histograms/Distribution_stages_cohorts/2021_11_23_14_43_distribution_stages_HNSC.pdf]

Driver event distribution by cancer stage in males KIRC

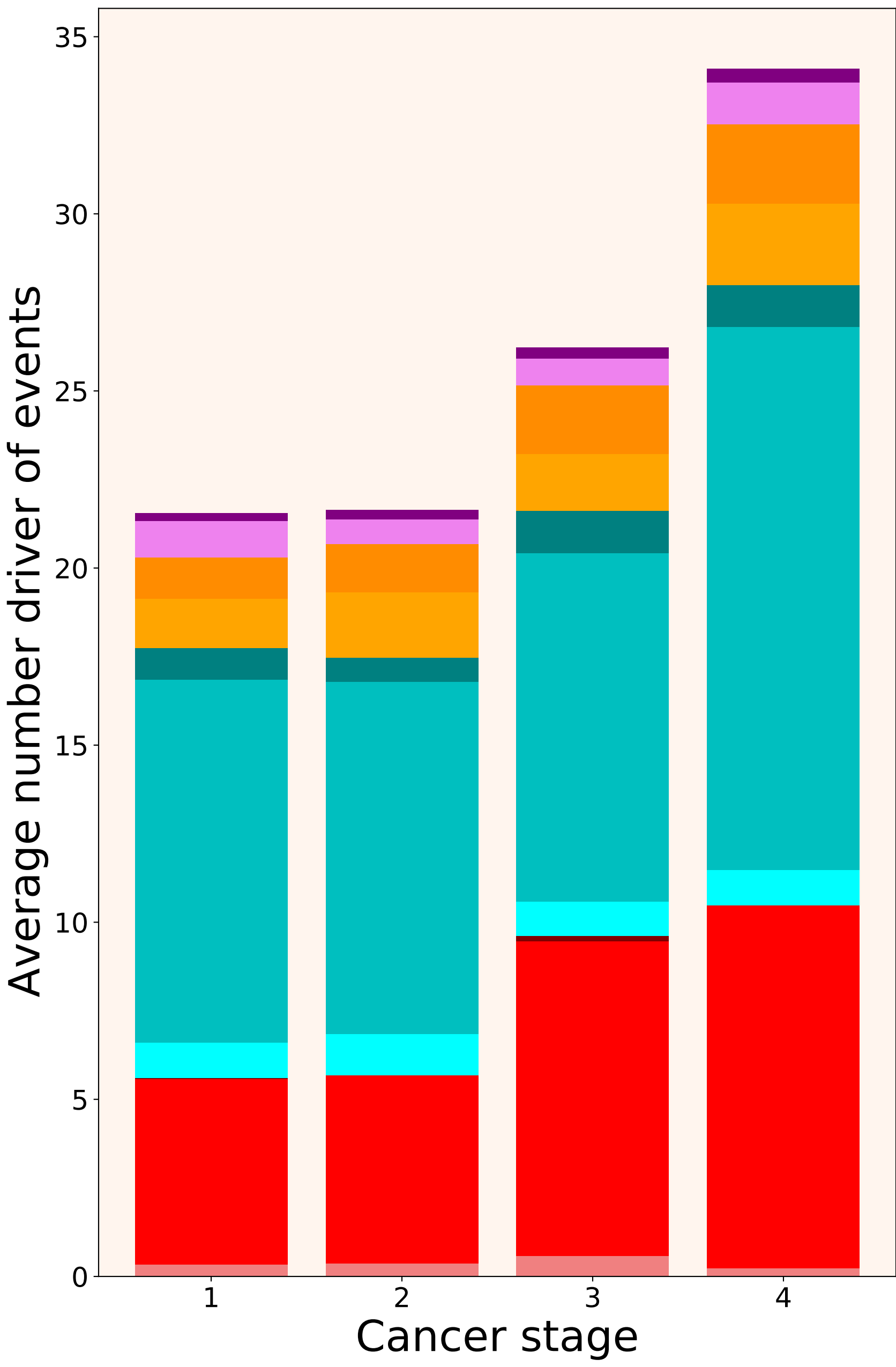

Supplement: S2 Files — (ZIP) [file pgen.1009996.s002.zip › PANCAN/cumulative histograms/Distribution_stages_cohorts/2021_11_23_14_43_distribution_stages_males_KIRC.pdf]

Driver event distribution by cancer stage in males COAD

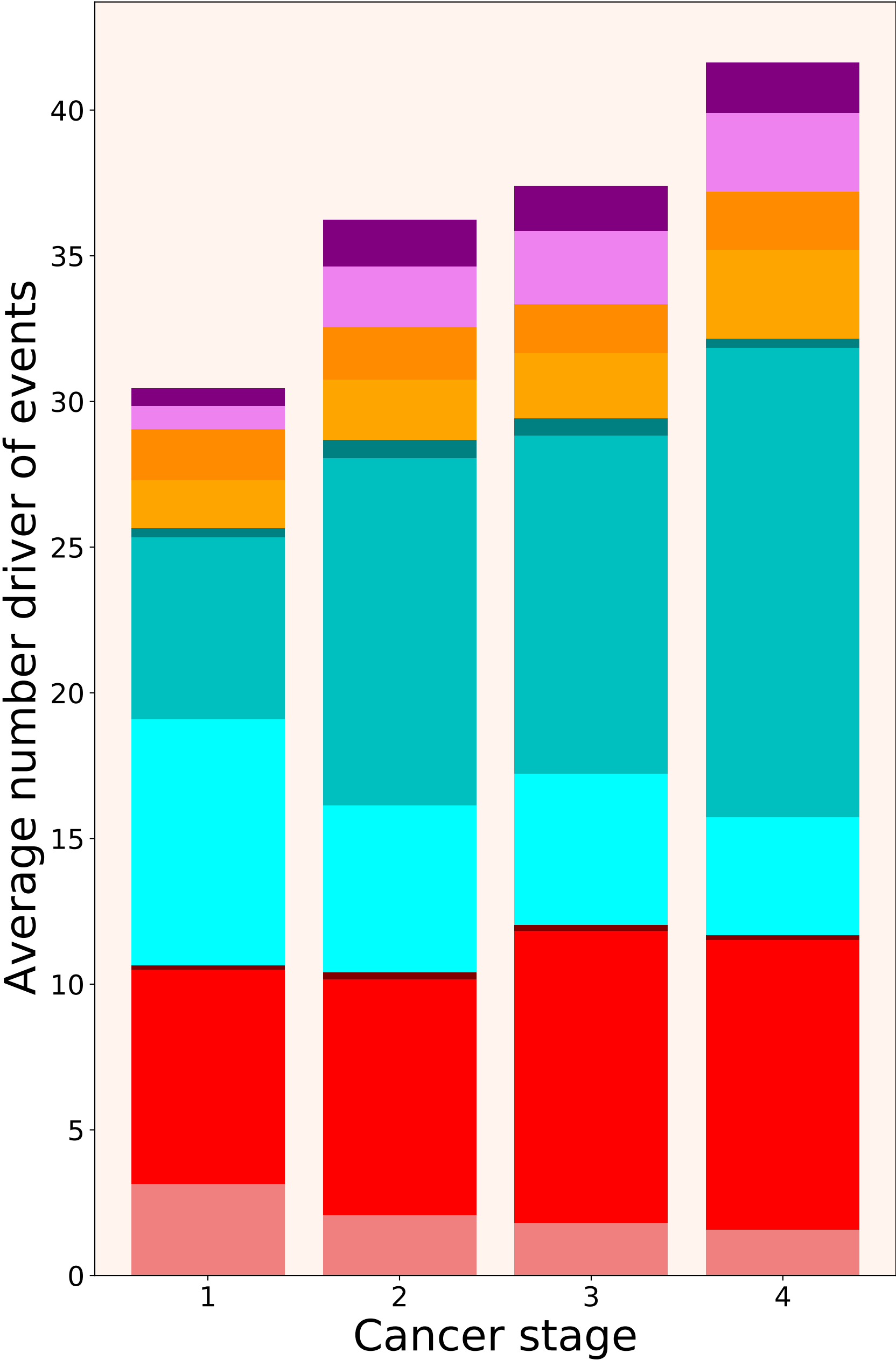

Supplement: S2 Files — (ZIP) [file pgen.1009996.s002.zip › PANCAN/cumulative histograms/Distribution_stages_cohorts/2021_11_23_14_43_distribution_stages_males_COAD.pdf]

Driver event distribution by cancer stage in males KIRP

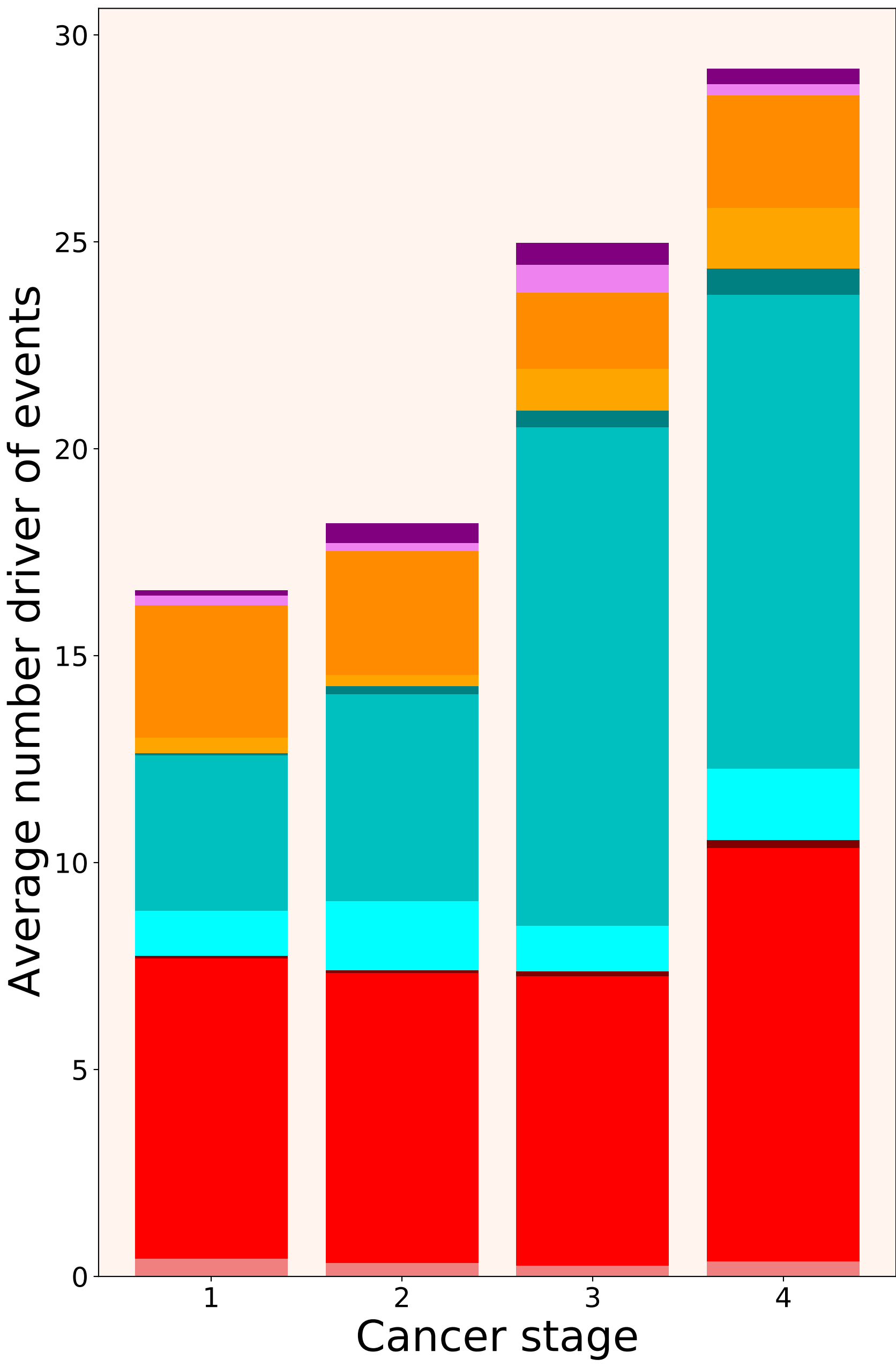

Supplement: S2 Files — (ZIP) [file pgen.1009996.s002.zip › PANCAN/cumulative histograms/Distribution_stages_cohorts/2021_11_23_14_43_distribution_stages_males_KIRP.pdf]

Driver event distribution by cancer stage in females LUAD

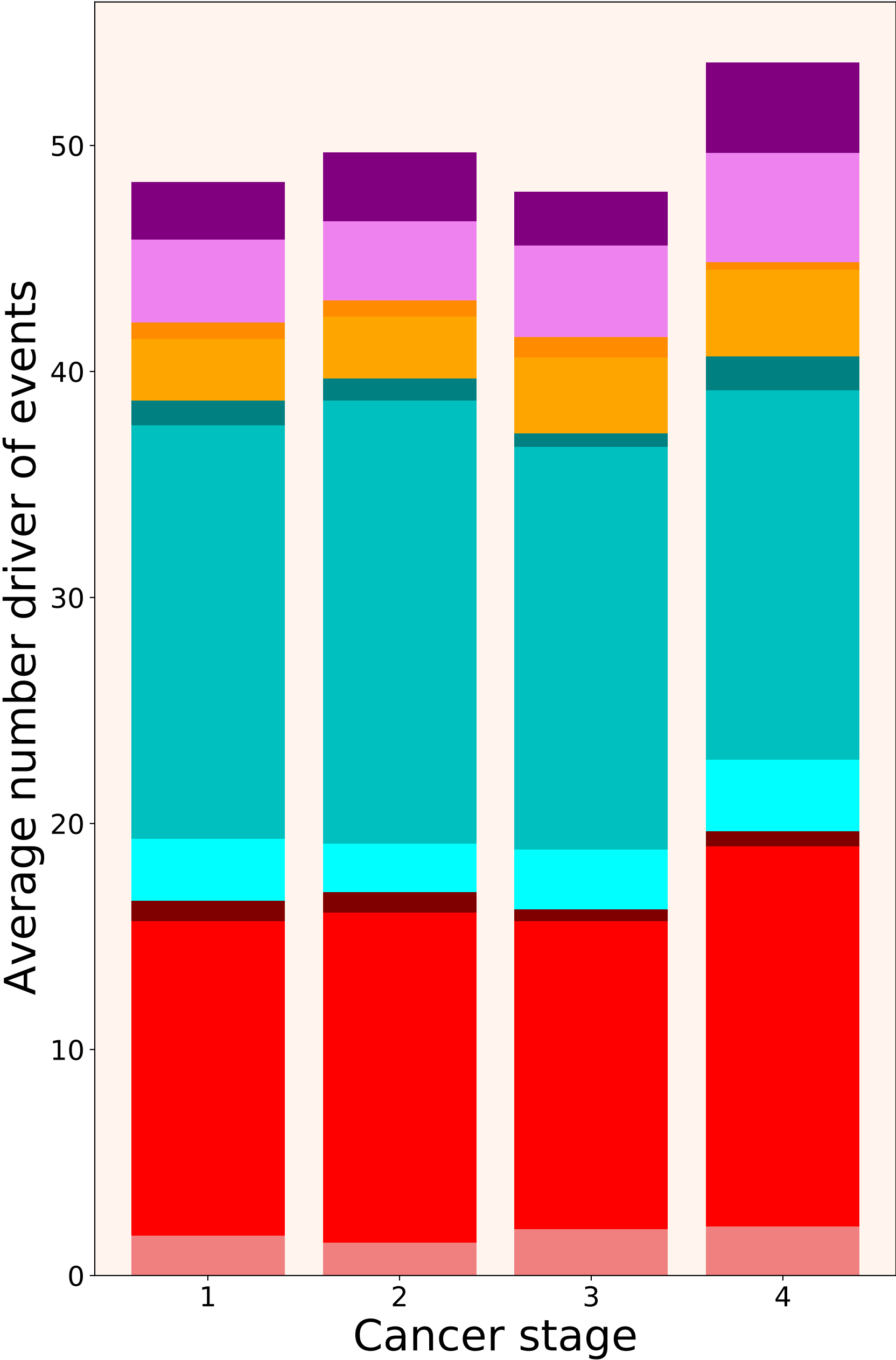

Supplement: S2 Files — (ZIP) [file pgen.1009996.s002.zip › PANCAN/cumulative histograms/Distribution_stages_cohorts/2021_11_23_14_43_distribution_stages_females_LUAD.pdf]

Driver event distribution by cancer stage PRAD

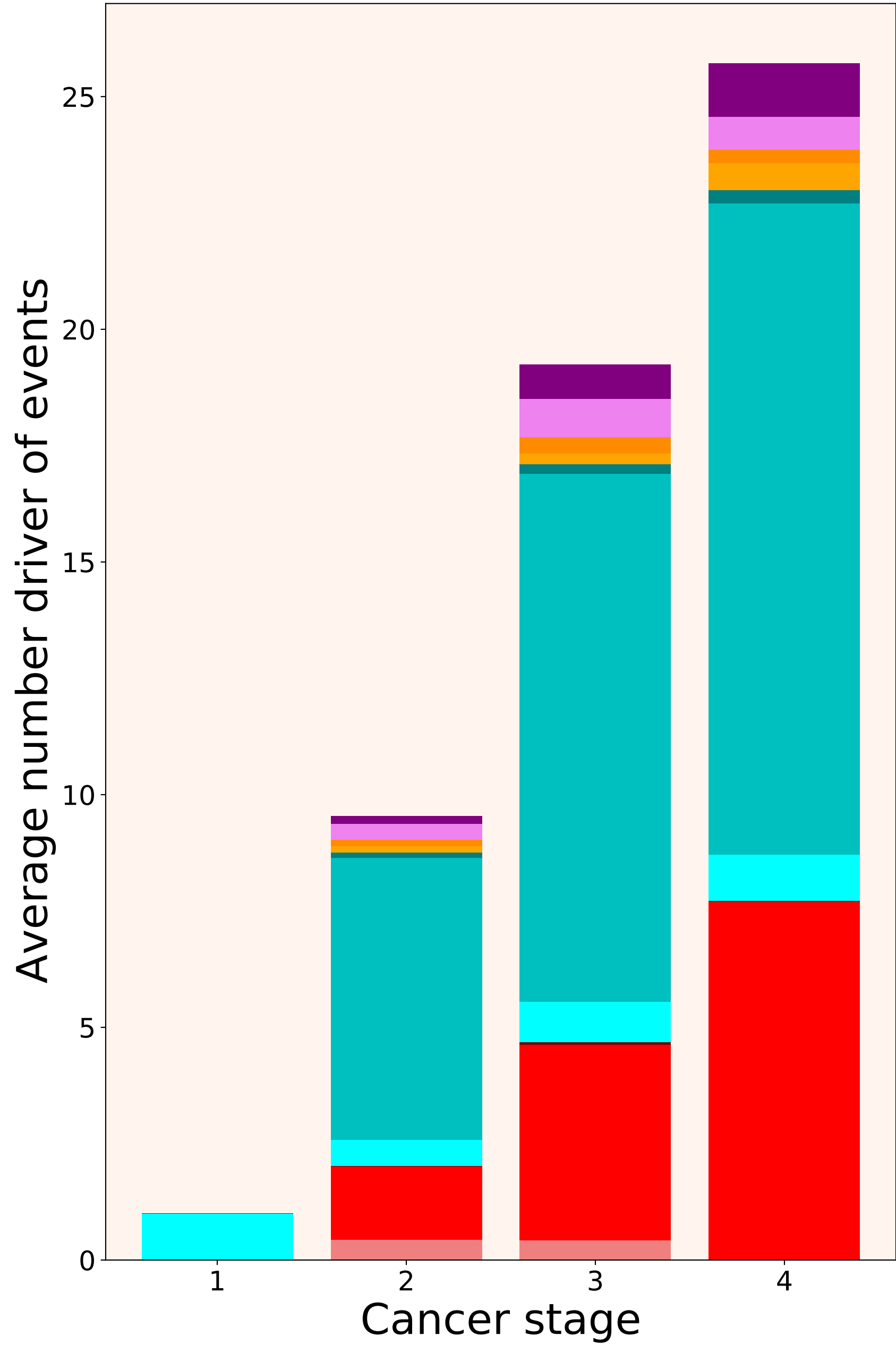

Supplement: S2 Files — (ZIP) [file pgen.1009996.s002.zip › PANCAN/cumulative histograms/Distribution_stages_cohorts/2021_11_23_14_43_distribution_stages_PRAD.pdf]

Driver event distribution by cancer stage in males LIHC

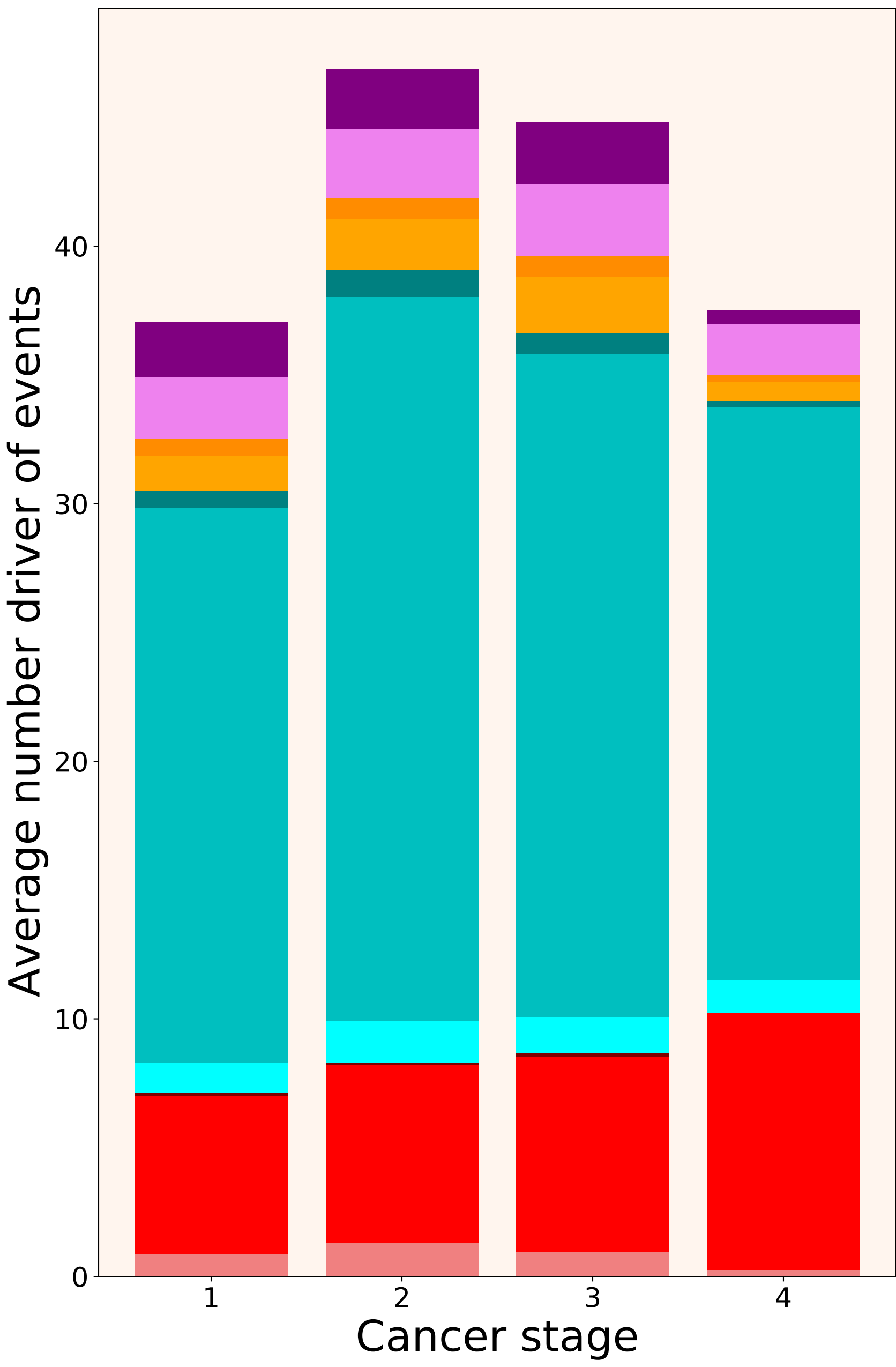

Supplement: S2 Files — (ZIP) [file pgen.1009996.s002.zip › PANCAN/cumulative histograms/Distribution_stages_cohorts/2021_11_23_14_43_distribution_stages_males_LIHC.pdf]

Driver event distribution by cancer stage in females UVM

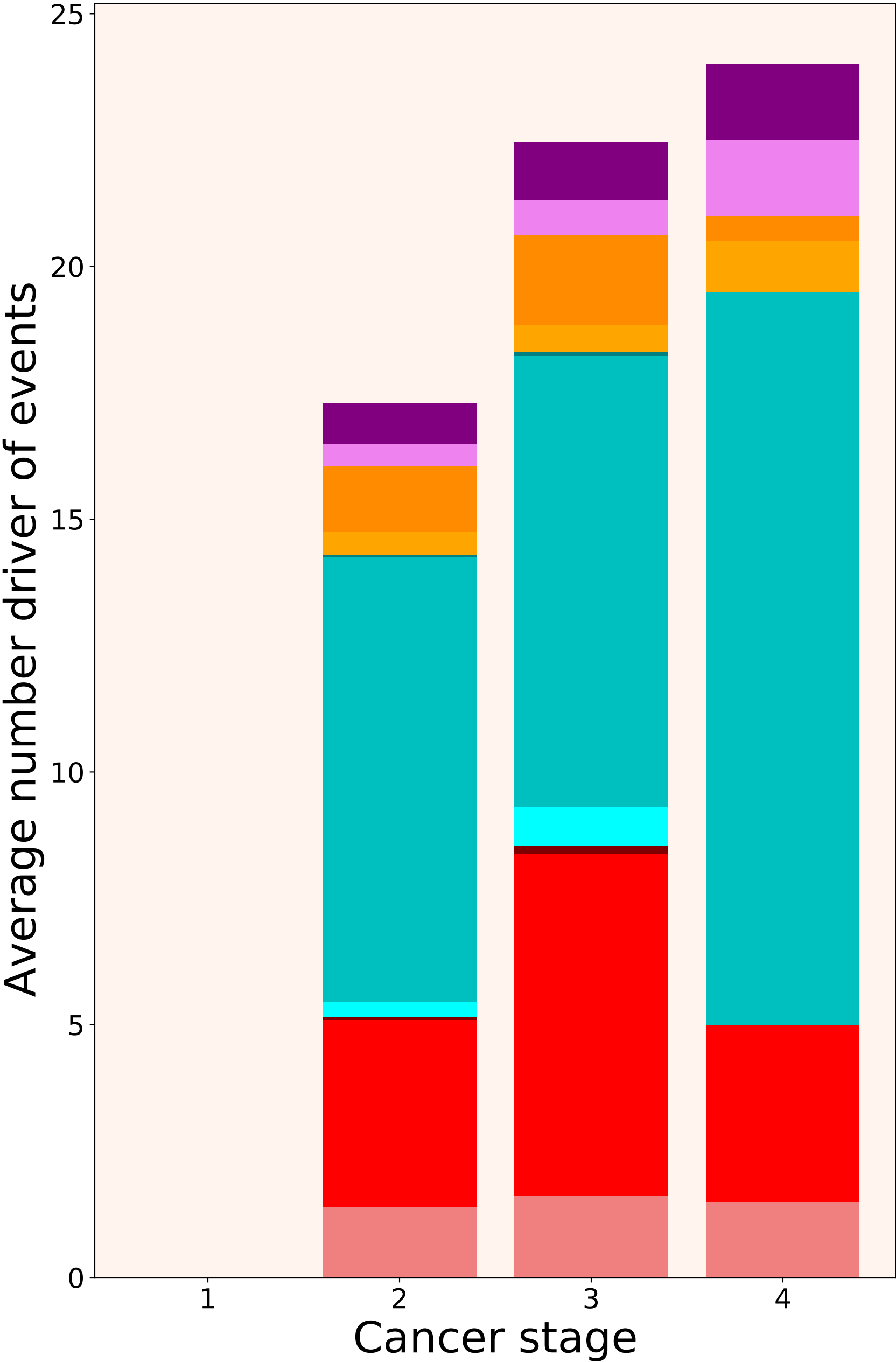

Supplement: S2 Files — (ZIP) [file pgen.1009996.s002.zip › PANCAN/cumulative histograms/Distribution_stages_cohorts/2021_11_23_14_43_distribution_stages_females_UVM.pdf]

Driver event distribution by cancer stage LUAD

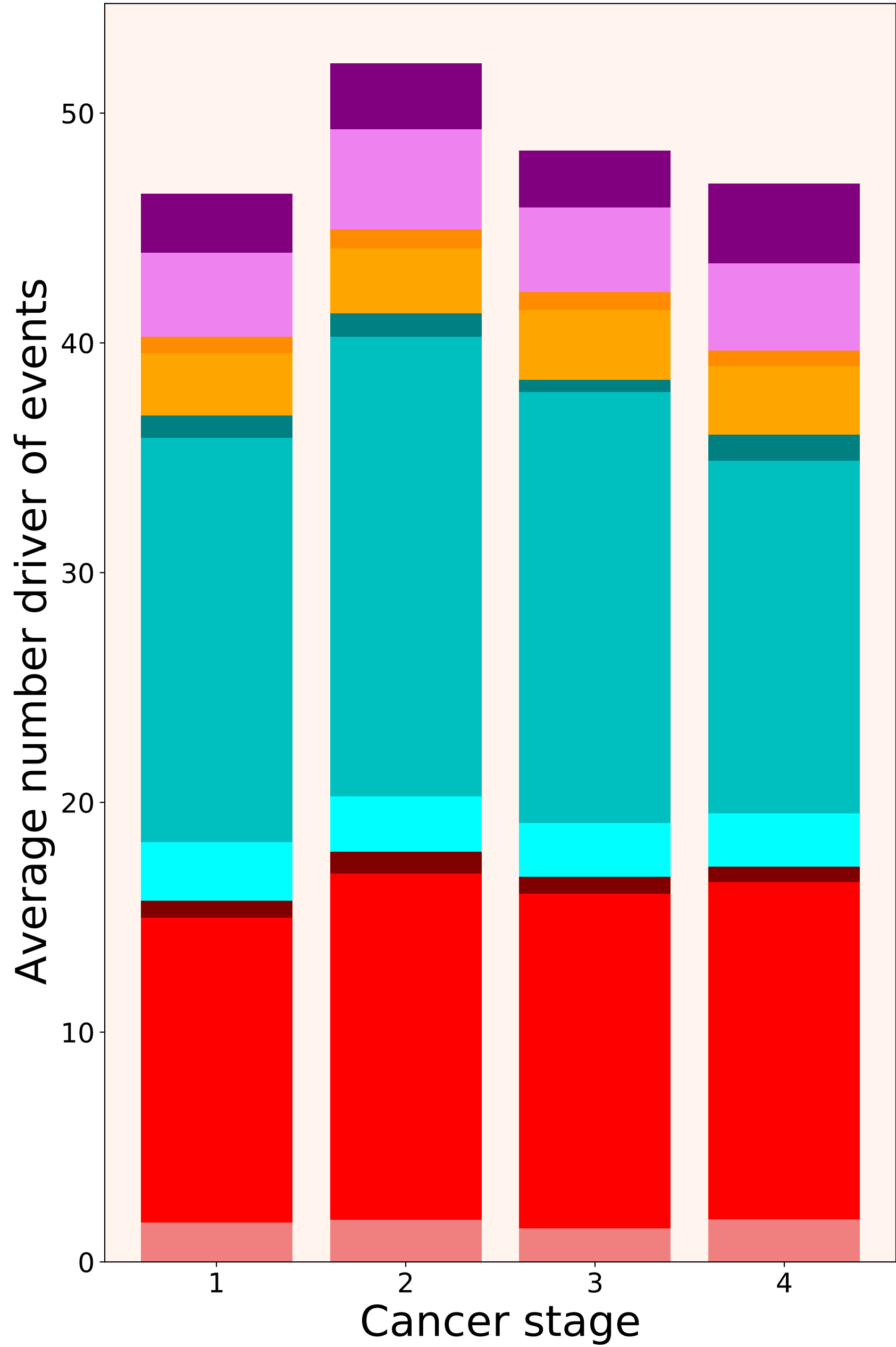

Supplement: S2 Files — (ZIP) [file pgen.1009996.s002.zip › PANCAN/cumulative histograms/Distribution_stages_cohorts/2021_11_23_14_43_distribution_stages_LUAD.pdf]

# Driver event distribution by cancer stage in males CHOL

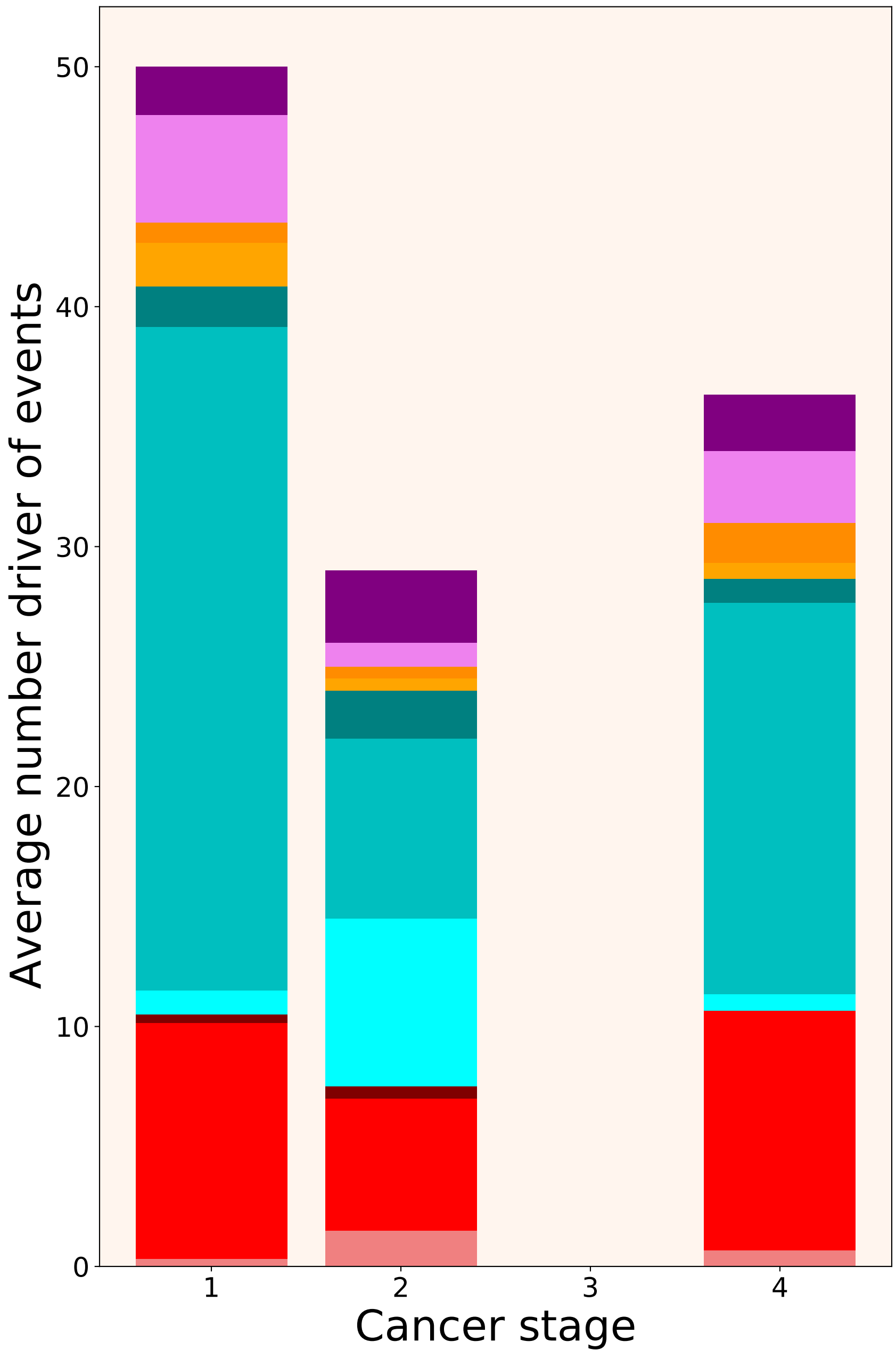

Supplement: S2 Files — (ZIP) [file pgen.1009996.s002.zip › PANCAN/cumulative histograms/Distribution_stages_cohorts/2021_11_23_14_43_distribution_stages_males_CHOL.pdf]

Driver event distribution by cancer stage in males STAD

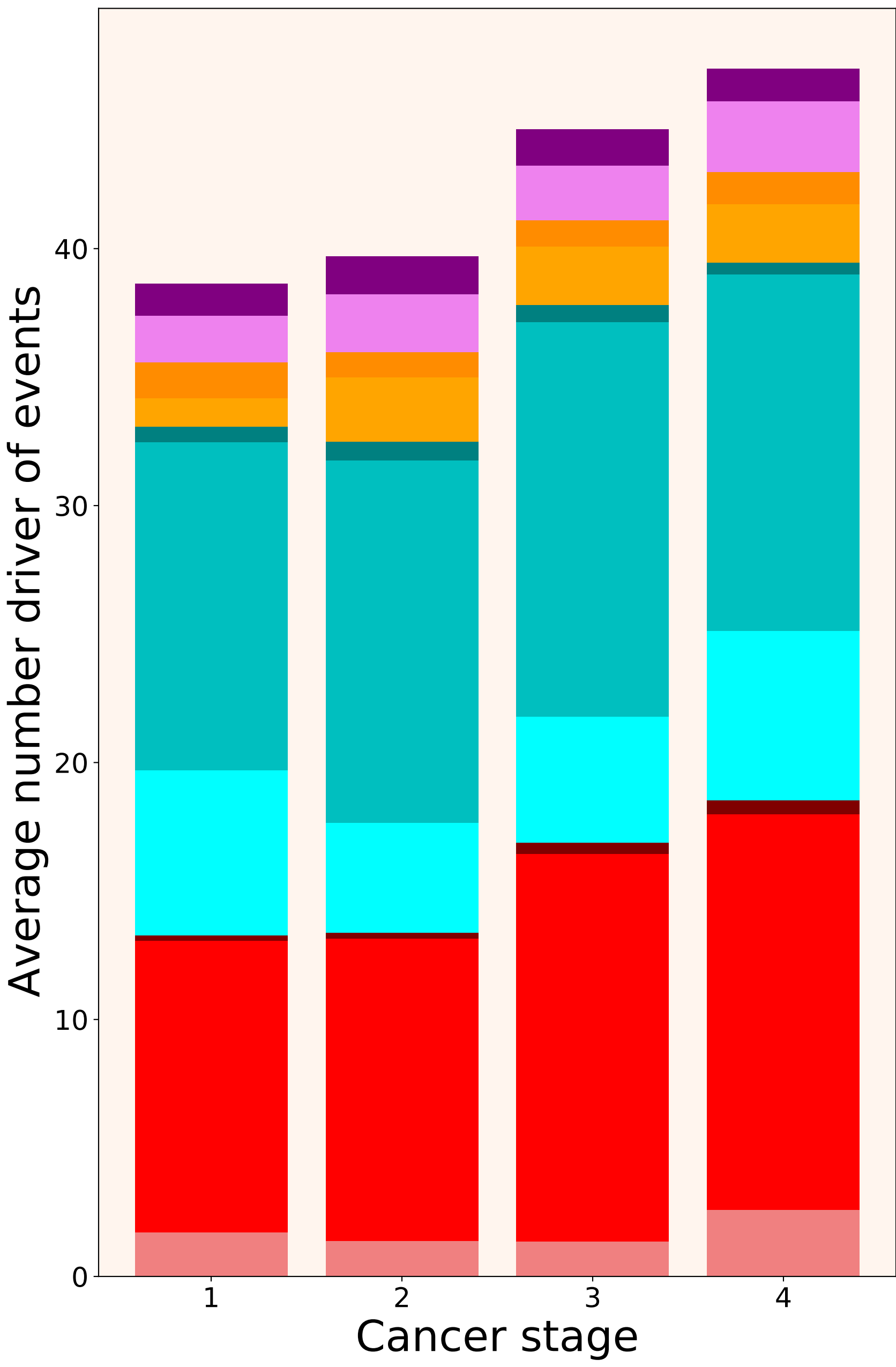

Supplement: S2 Files — (ZIP) [file pgen.1009996.s002.zip › PANCAN/cumulative histograms/Distribution_stages_cohorts/2021_11_23_14_43_distribution_stages_males_STAD.pdf]

Driver event distribution by cancer stage TGCT

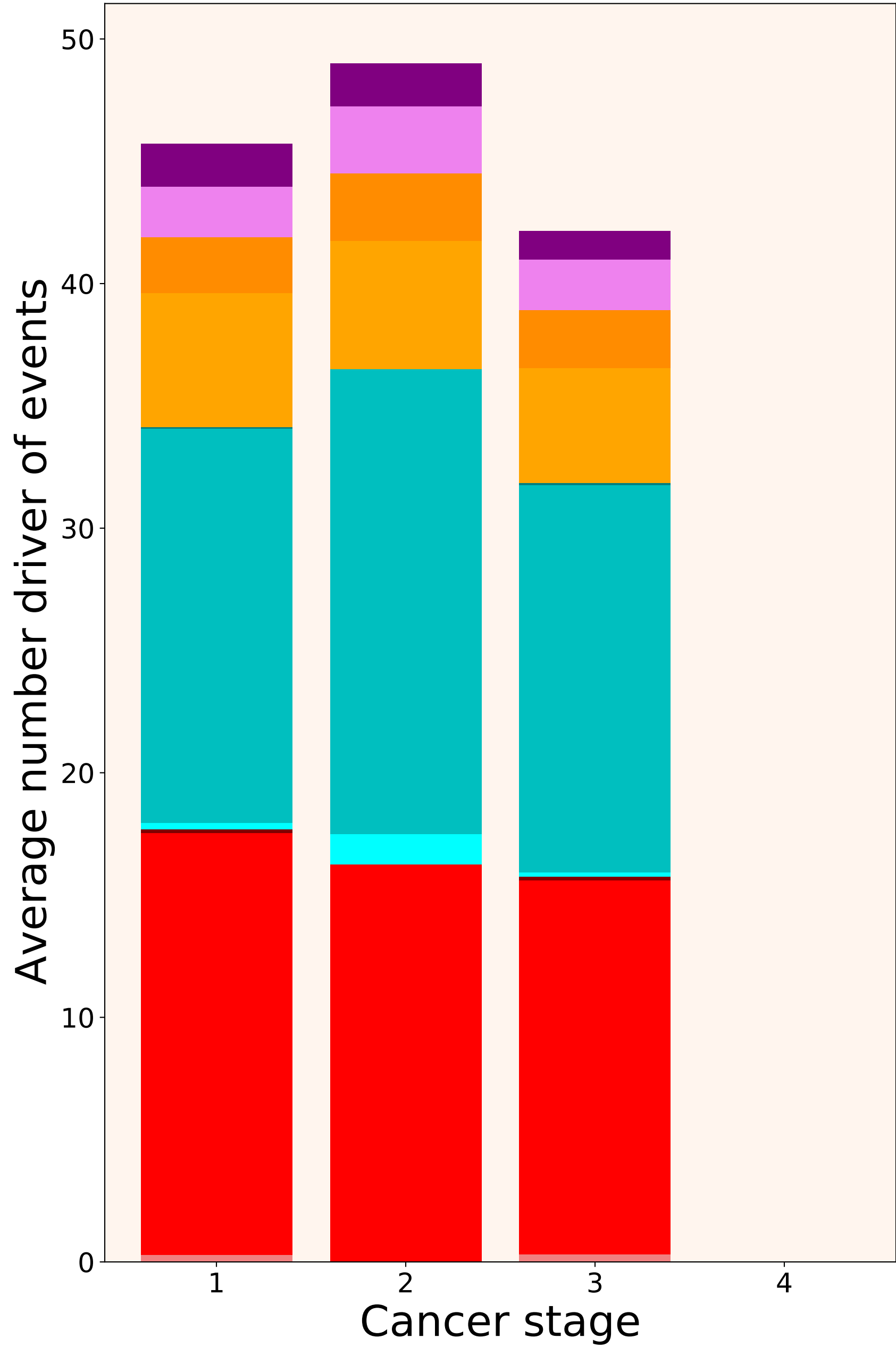

Supplement: S2 Files — (ZIP) [file pgen.1009996.s002.zip › PANCAN/cumulative histograms/Distribution_stages_cohorts/2021_11_23_14_43_distribution_stages_TGCT.pdf]

Driver event distribution by cancer stage in females CESC

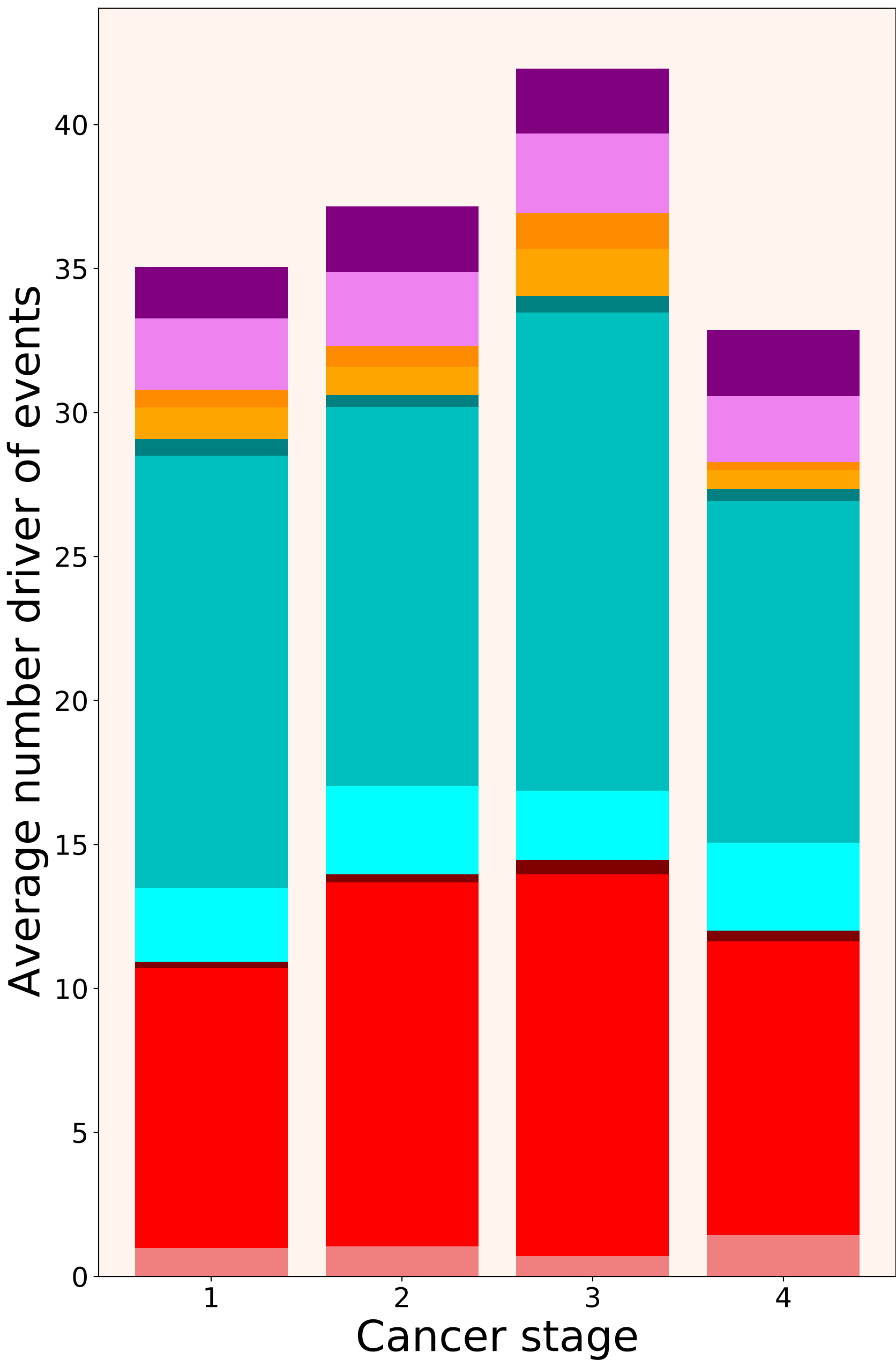

Supplement: S2 Files — (ZIP) [file pgen.1009996.s002.zip › PANCAN/cumulative histograms/Distribution_stages_cohorts/2021_11_23_14_43_distribution_stages_females_CESC.pdf]

Driver event distribution by cancer stage CESC

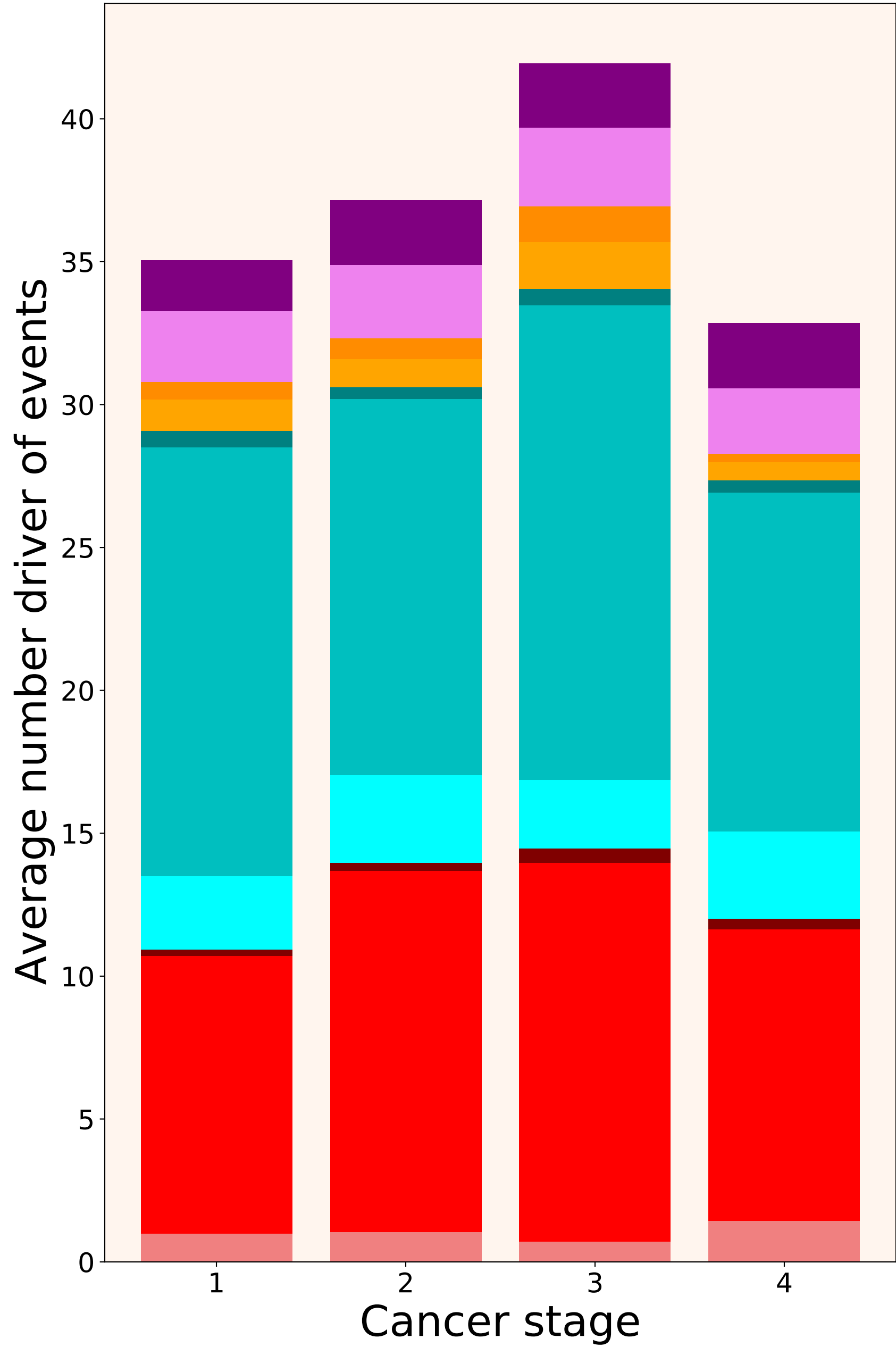

Supplement: S2 Files — (ZIP) [file pgen.1009996.s002.zip › PANCAN/cumulative histograms/Distribution_stages_cohorts/2021_11_23_14_43_distribution_stages_CESC.pdf]

Driver event distribution by cancer stage in males KICH

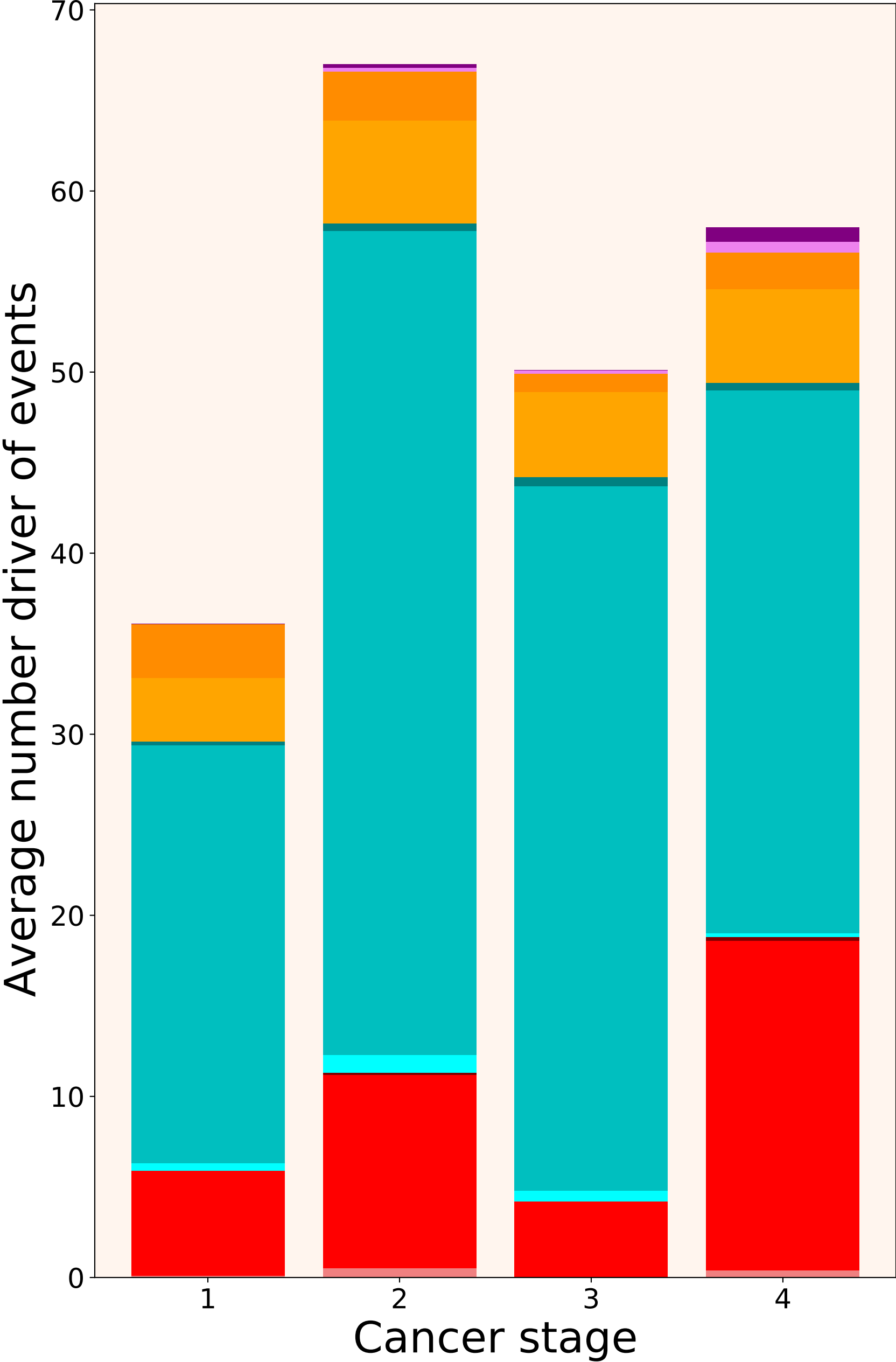

Supplement: S2 Files — (ZIP) [file pgen.1009996.s002.zip › PANCAN/cumulative histograms/Distribution_stages_cohorts/2021_11_23_14_43_distribution_stages_males_KICH.pdf]

Driver event distribution by cancer stage in males ACC

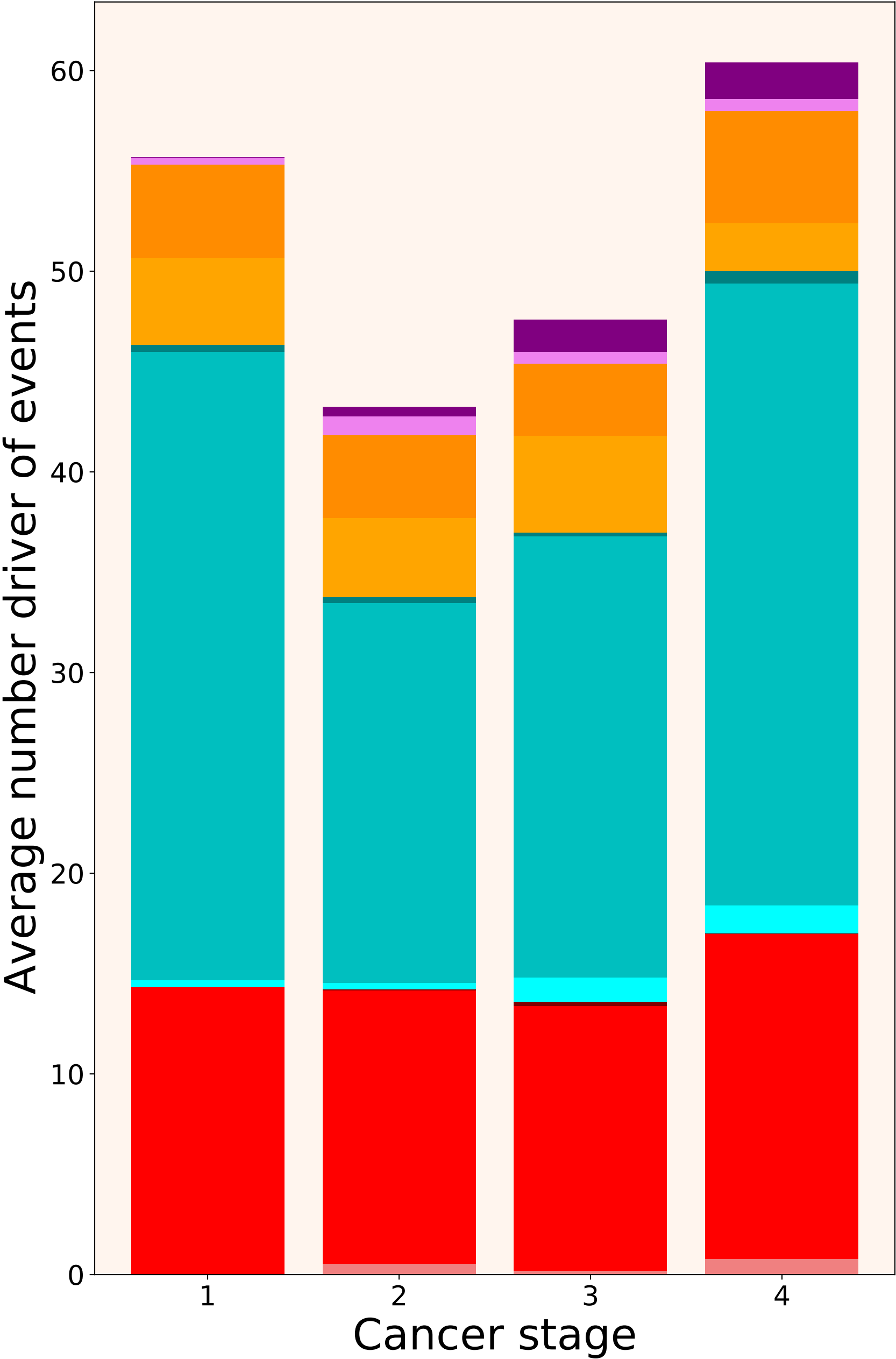

Supplement: S2 Files — (ZIP) [file pgen.1009996.s002.zip › PANCAN/cumulative histograms/Distribution_stages_cohorts/2021_11_23_14_43_distribution_stages_males_ACC.pdf]

Driver event distribution by cancer stage ACC

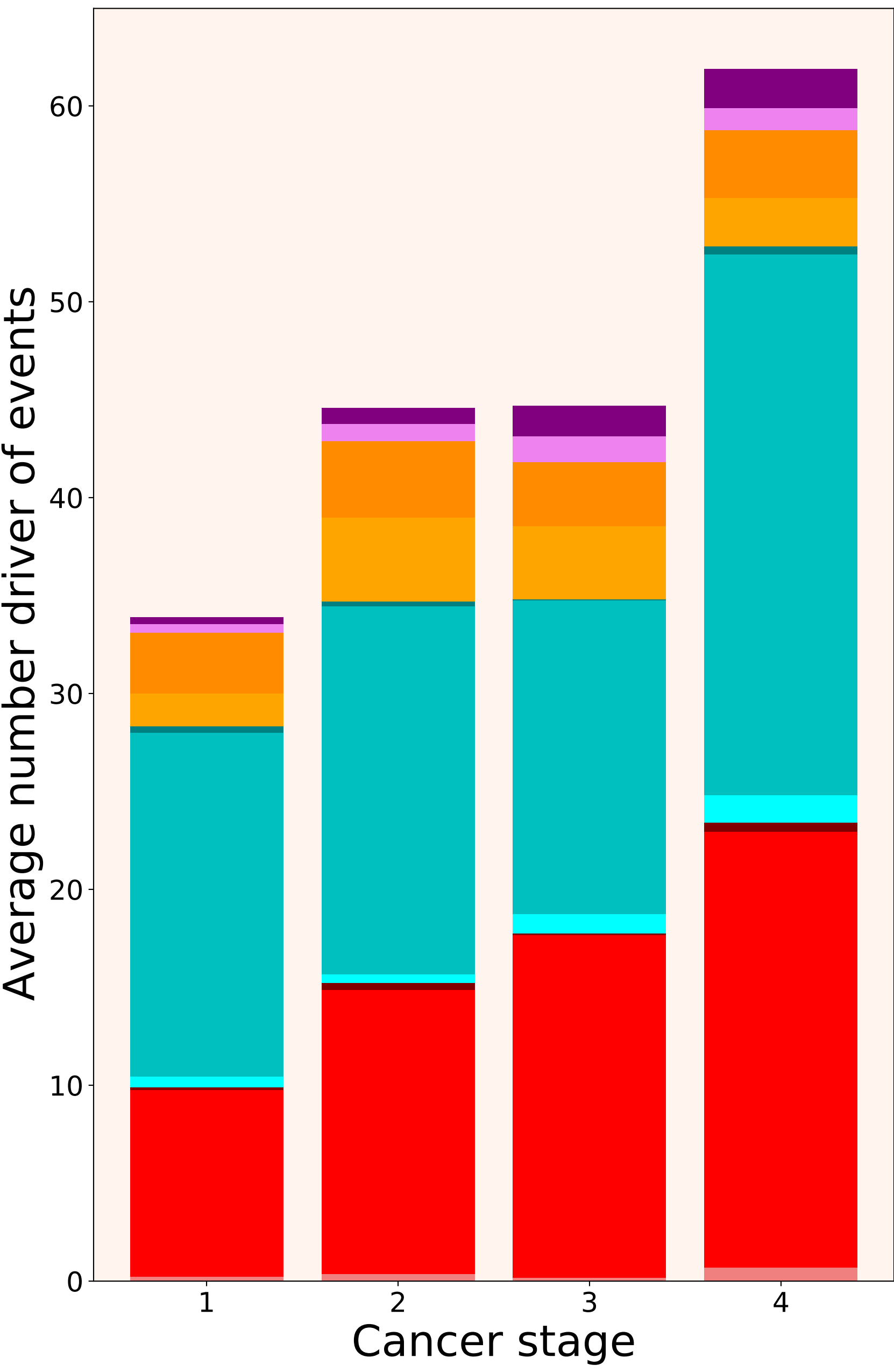

Supplement: S2 Files — (ZIP) [file pgen.1009996.s002.zip › PANCAN/cumulative histograms/Distribution_stages_cohorts/2021_11_23_14_43_distribution_stages_ACC.pdf]

Driver event distribution by cancer stage in females ACC

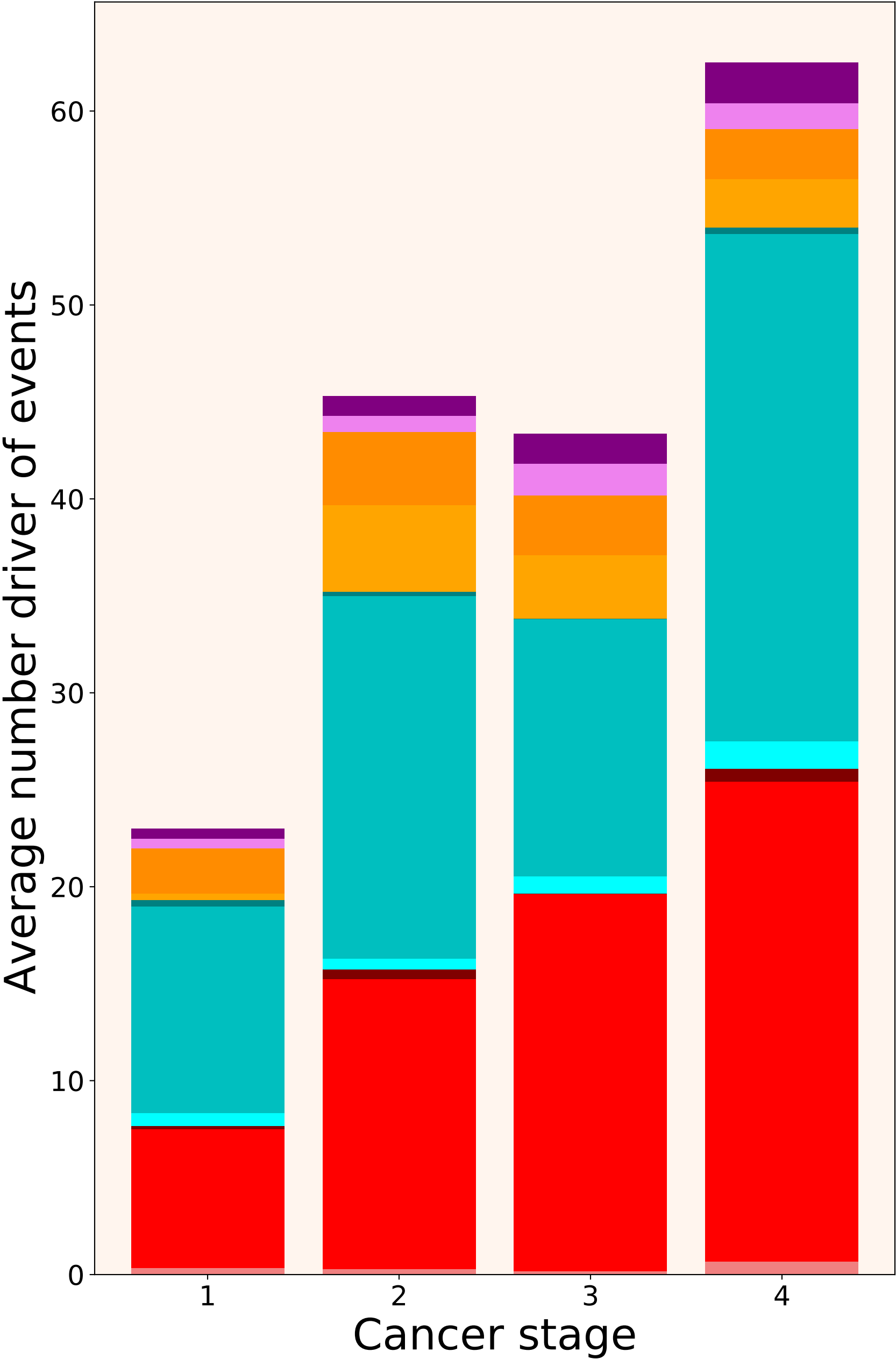

Supplement: S2 Files — (ZIP) [file pgen.1009996.s002.zip › PANCAN/cumulative histograms/Distribution_stages_cohorts/2021_11_23_14_43_distribution_stages_females_ACC.pdf]

Driver event distribution by cancer stage in males BRCA

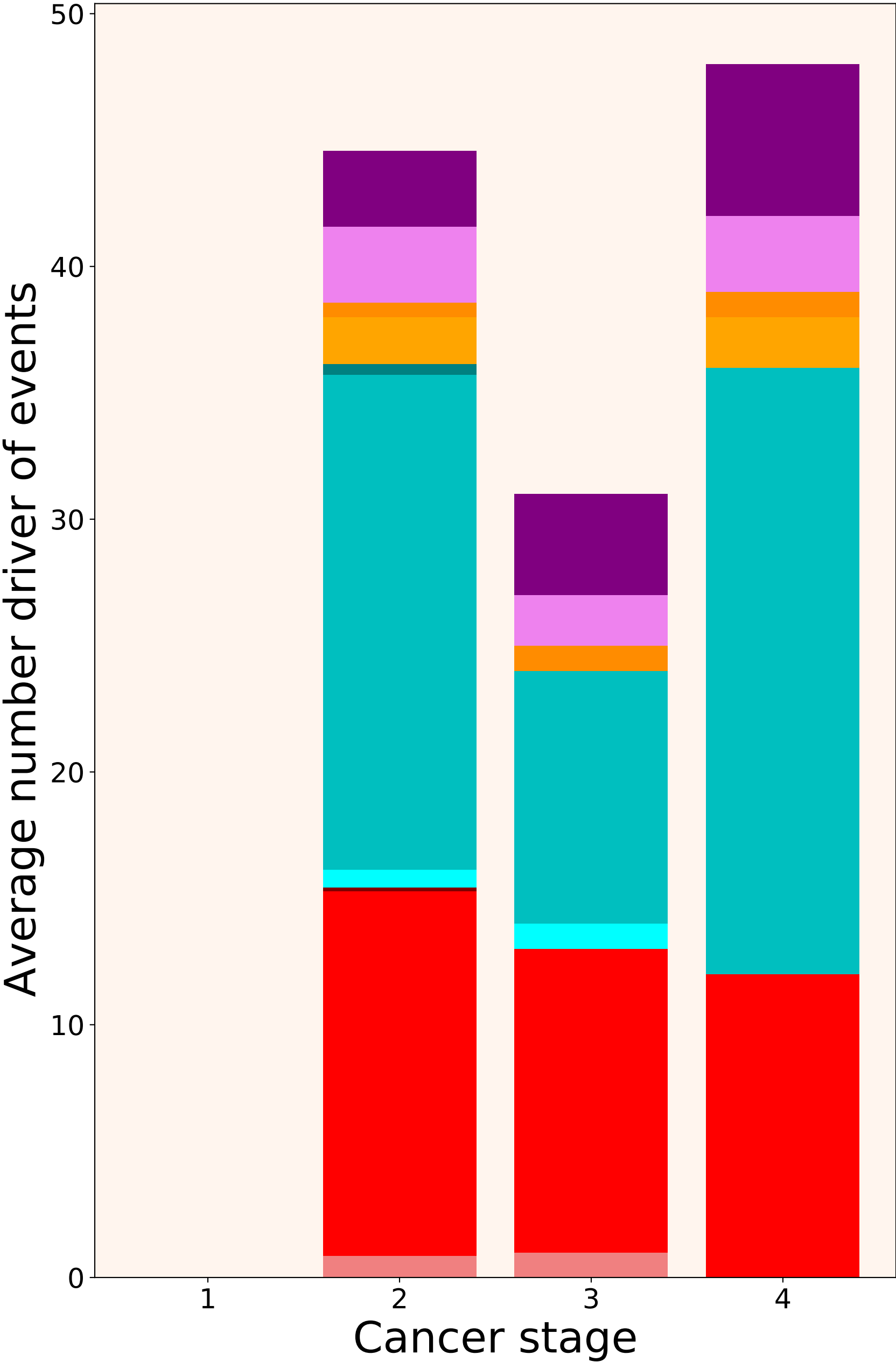

Supplement: S2 Files — (ZIP) [file pgen.1009996.s002.zip › PANCAN/cumulative histograms/Distribution_stages_cohorts/2021_11_23_14_43_distribution_stages_males_BRCA.pdf]

Driver event distribution by cancer stage in females LUSC

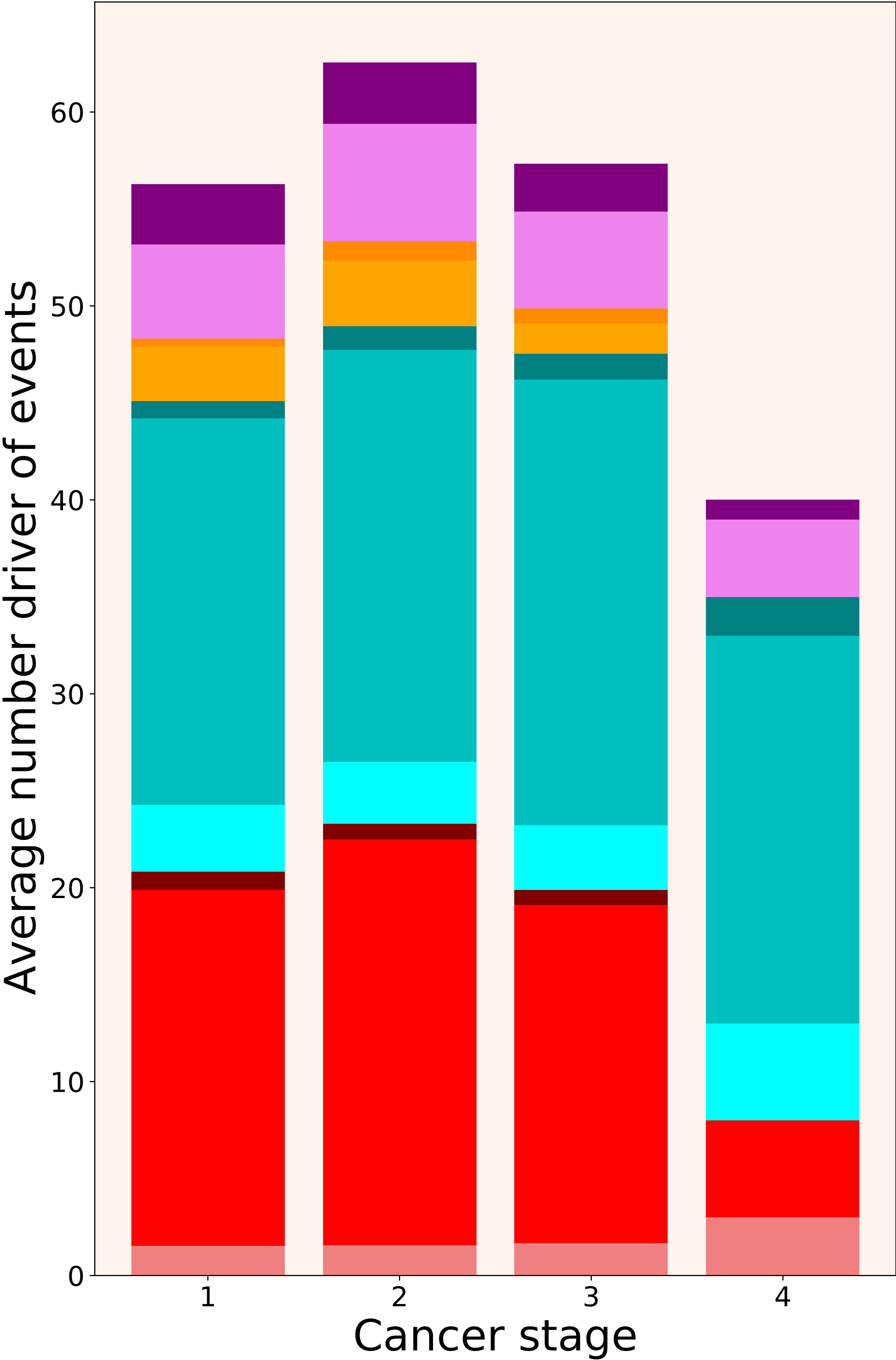

Supplement: S2 Files — (ZIP) [file pgen.1009996.s002.zip › PANCAN/cumulative histograms/Distribution_stages_cohorts/2021_11_23_14_43_distribution_stages_females_LUSC.pdf]

Driver event distribution by cancer stage in males SKCM

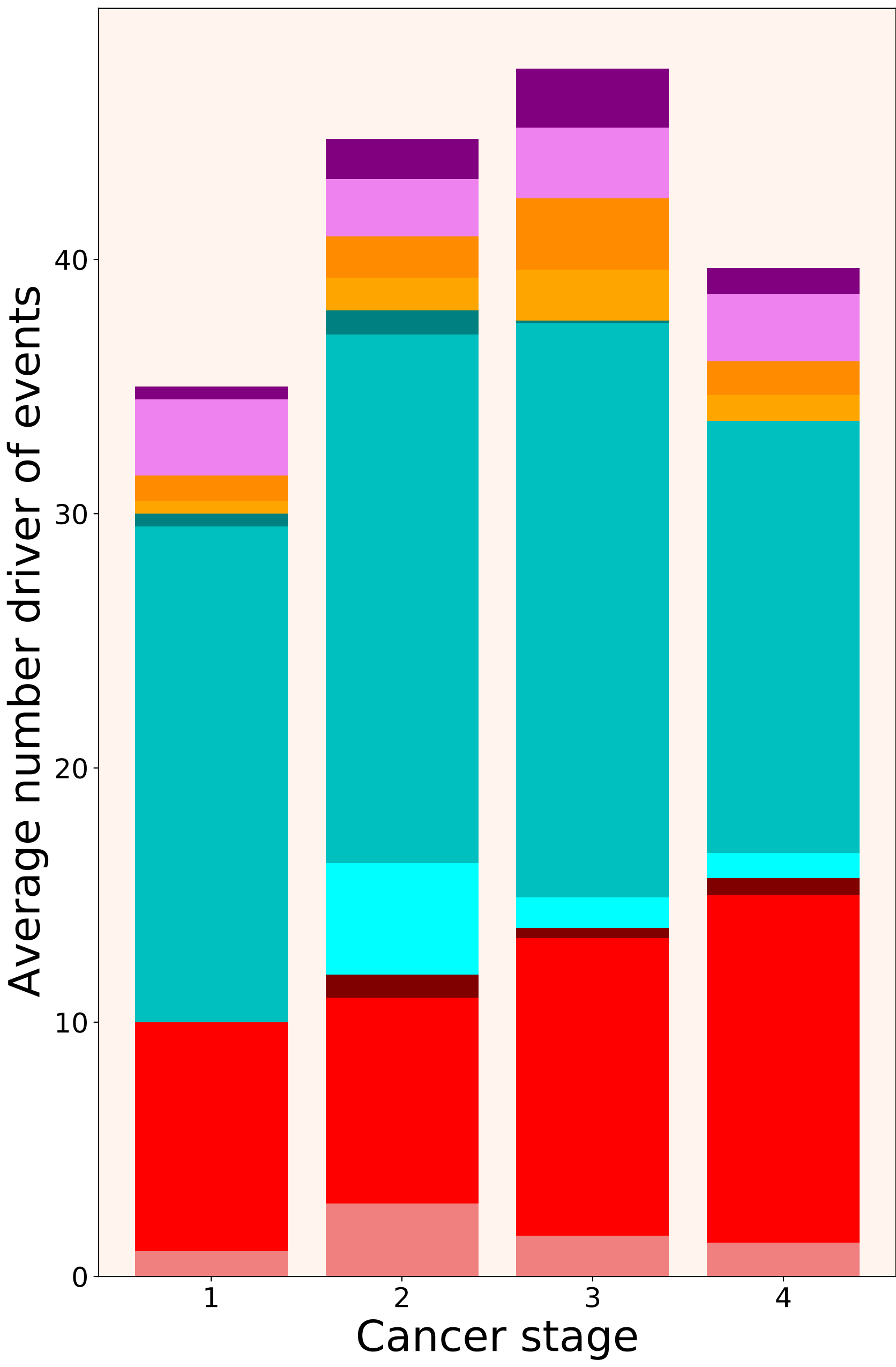

Supplement: S2 Files — (ZIP) [file pgen.1009996.s002.zip › PANCAN/cumulative histograms/Distribution_stages_cohorts/2021_11_23_14_43_distribution_stages_males_SKCM.pdf]

Driver event distribution by cancer stage LUSC

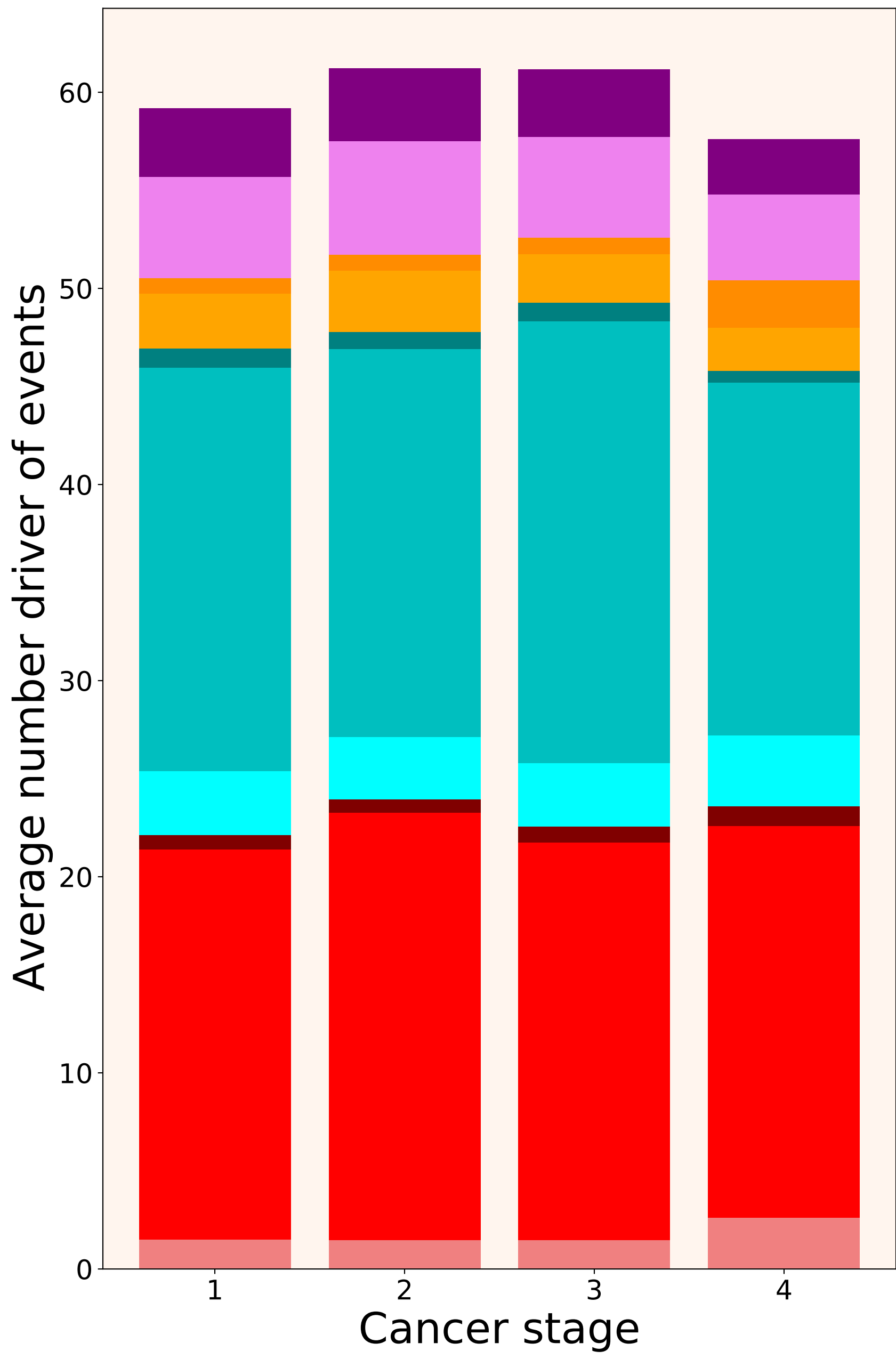

Supplement: S2 Files — (ZIP) [file pgen.1009996.s002.zip › PANCAN/cumulative histograms/Distribution_stages_cohorts/2021_11_23_14_43_distribution_stages_LUSC.pdf]

Driver event distribution by cancer stage in males DLBC

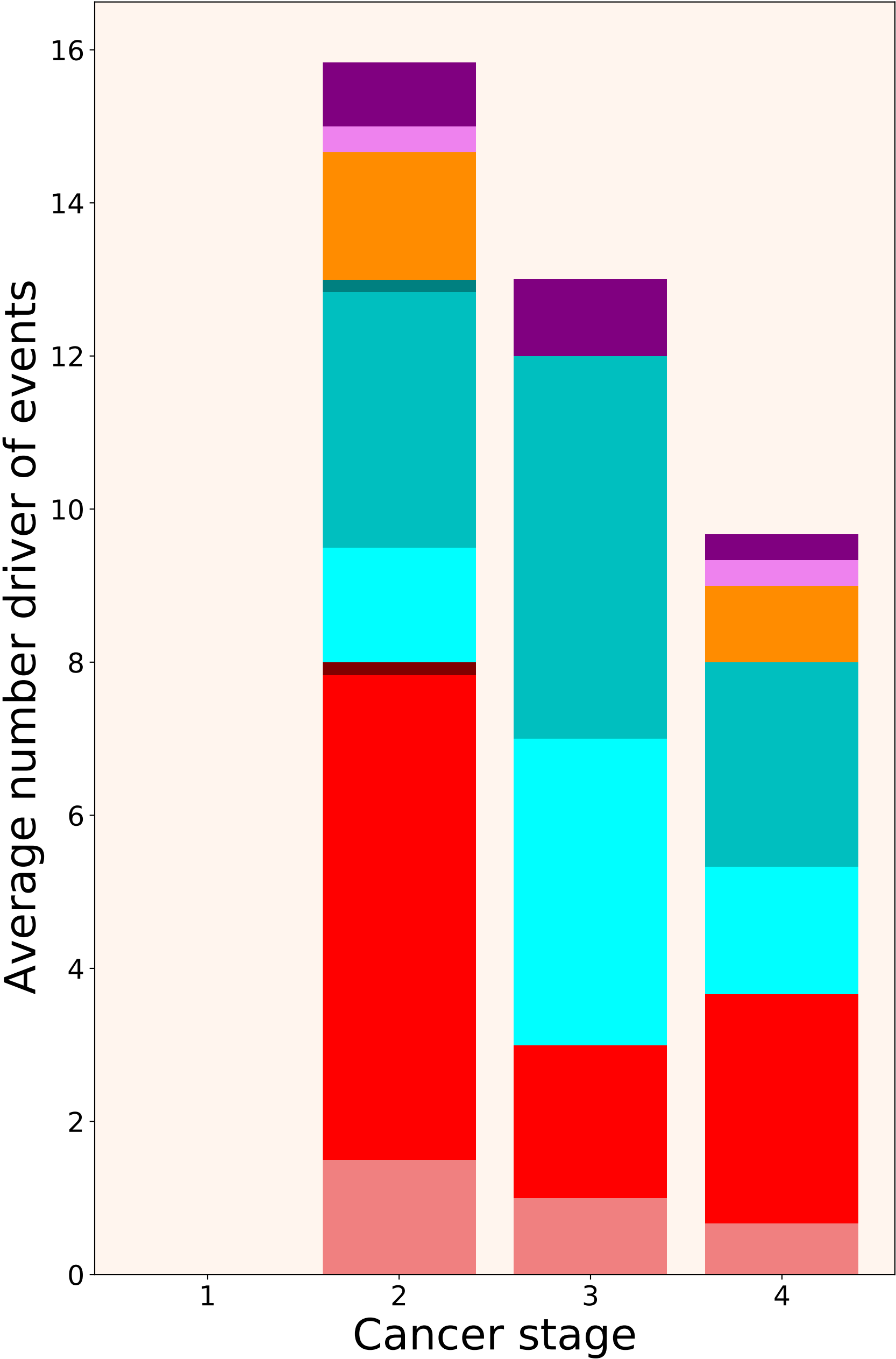

Supplement: S2 Files — (ZIP) [file pgen.1009996.s002.zip › PANCAN/cumulative histograms/Distribution_stages_cohorts/2021_11_23_14_43_distribution_stages_males_DLBC.pdf]

Driver event distribution by cancer type in males

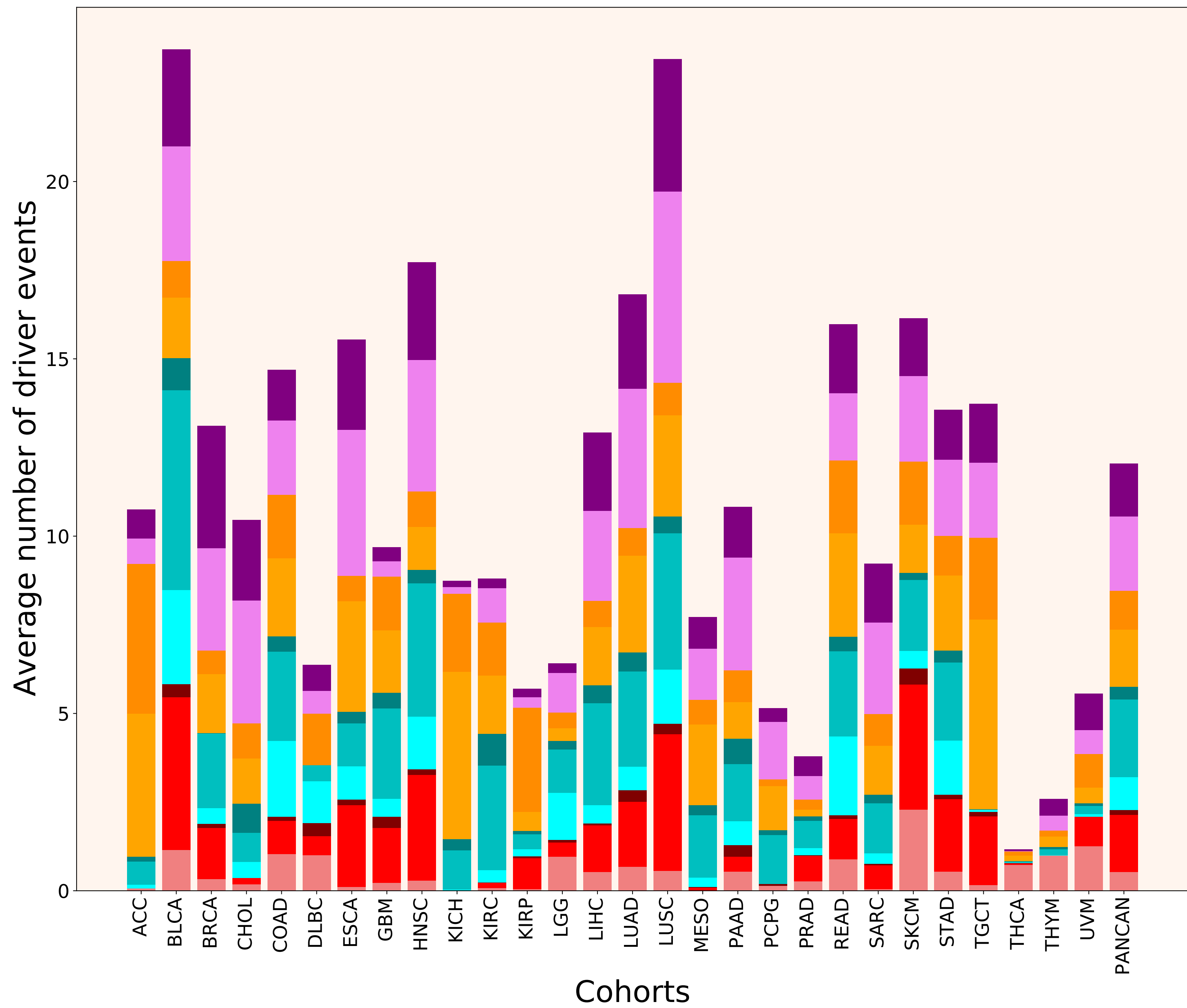

Supplement: S3 Files — (ZIP) [file pgen.1009996.s003.zip › COHORTS/cumulative histograms/2021_11_23_14_20_distribution_cohorts_males.pdf]

Driver event distribution by cancer stage in males

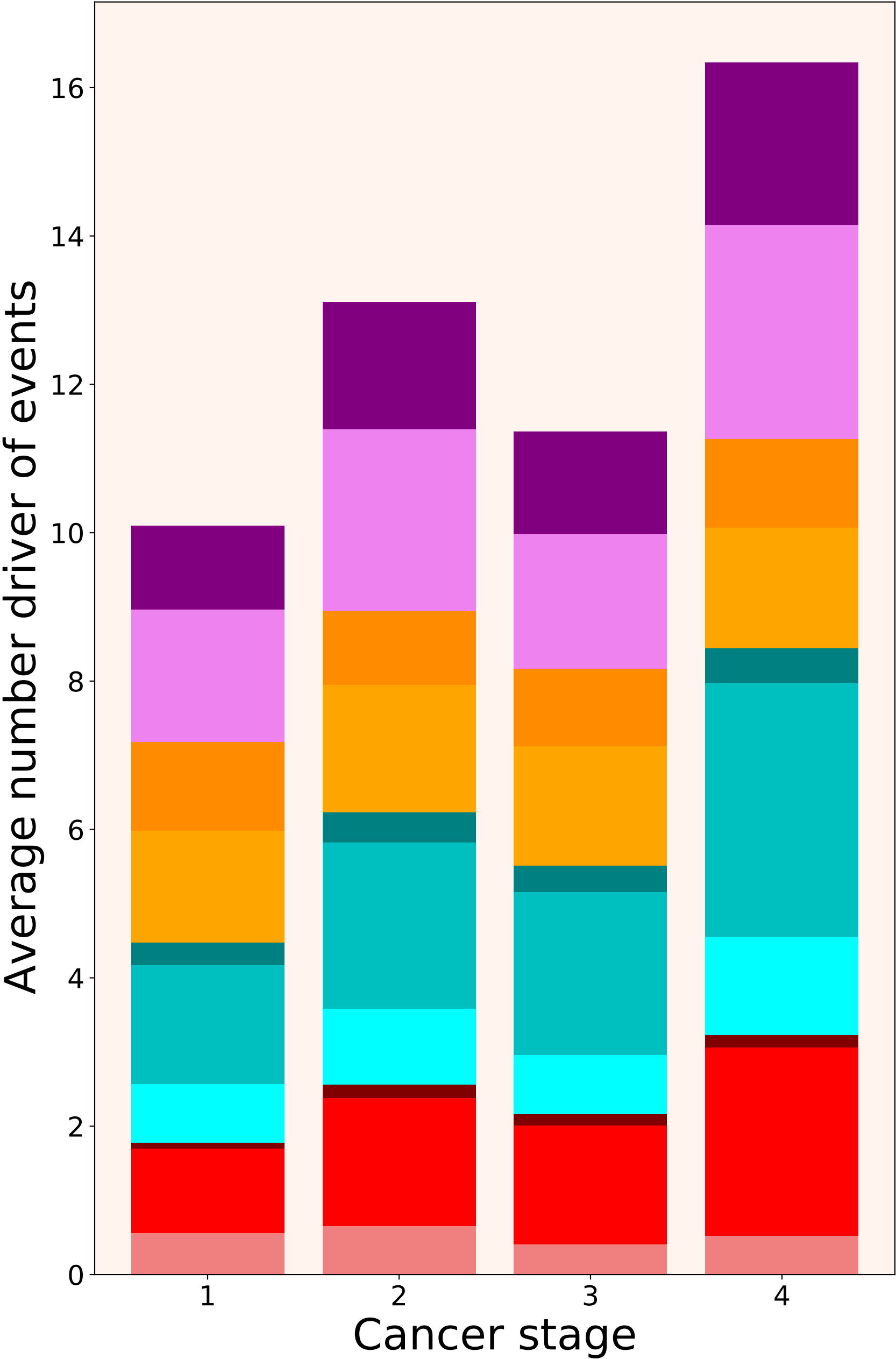

Supplement: S3 Files — (ZIP) [file pgen.1009996.s003.zip › COHORTS/cumulative histograms/2021_11_23_14_20_distribution_stages_males.pdf]

Driver event distribution by total number of driver events per patient

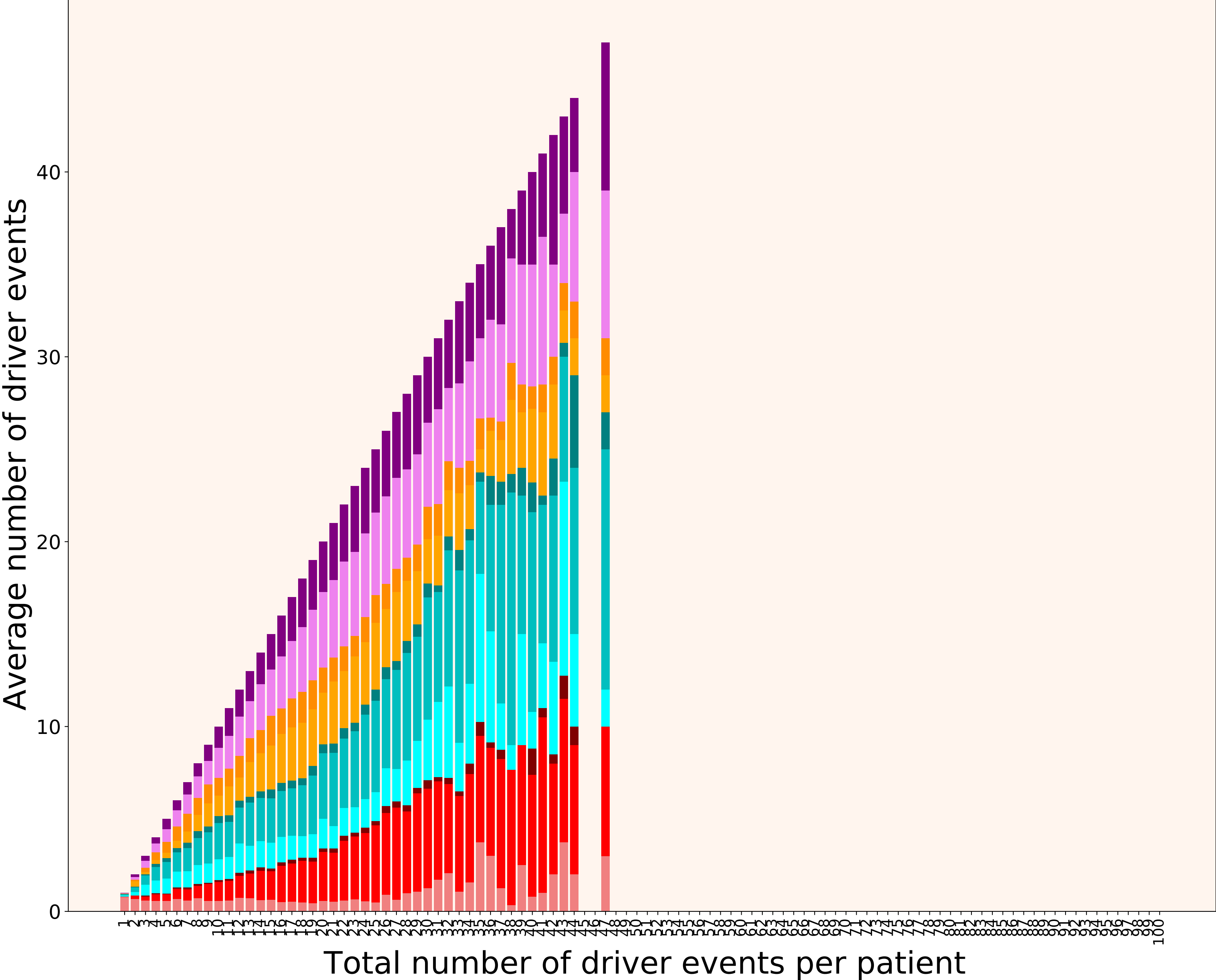

Supplement: S3 Files — (ZIP) [file pgen.1009996.s003.zip › COHORTS/cumulative histograms/2021_11_23_14_20_distribution_events_detailed.pdf]

Driver event distribution by age

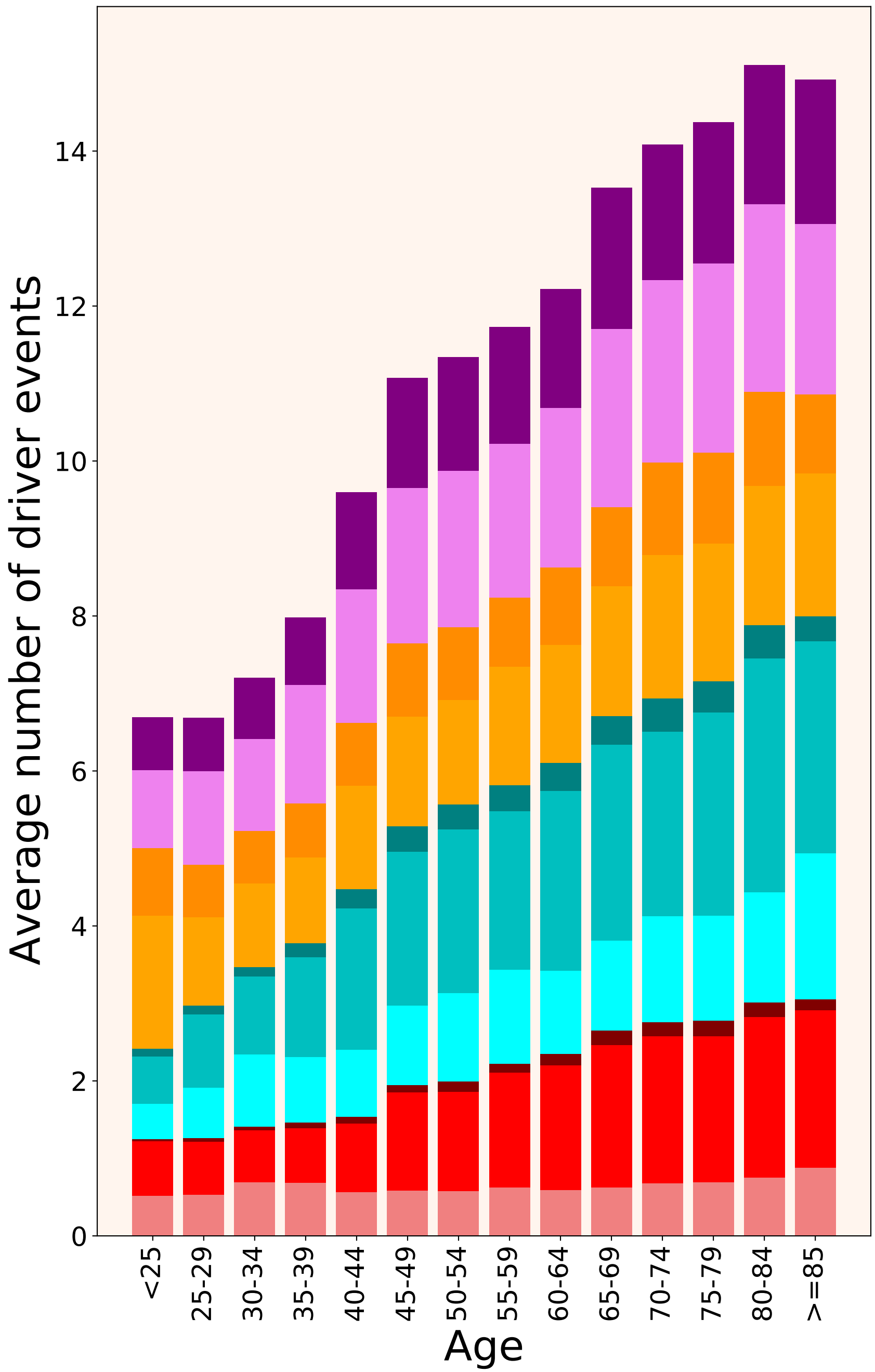

Supplement: S3 Files — (ZIP) [file pgen.1009996.s003.zip › COHORTS/cumulative histograms/2021_11_23_14_20_distribution_age.pdf]

Driver event distribution by age in males

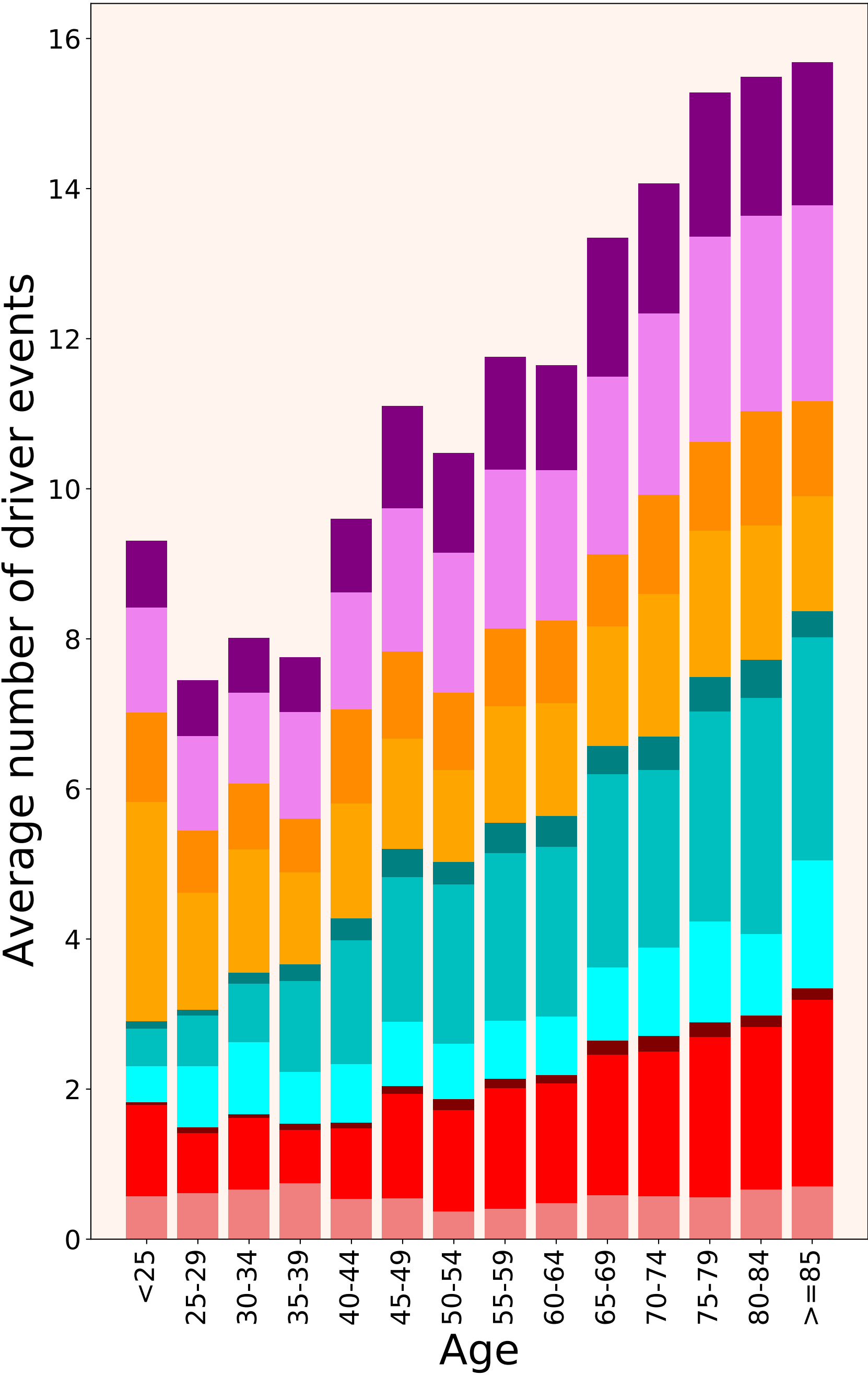

Supplement: S3 Files — (ZIP) [file pgen.1009996.s003.zip › COHORTS/cumulative histograms/2021_11_23_14_20_distribution_age_males.pdf]

Driver event distribution by cancer stage in females

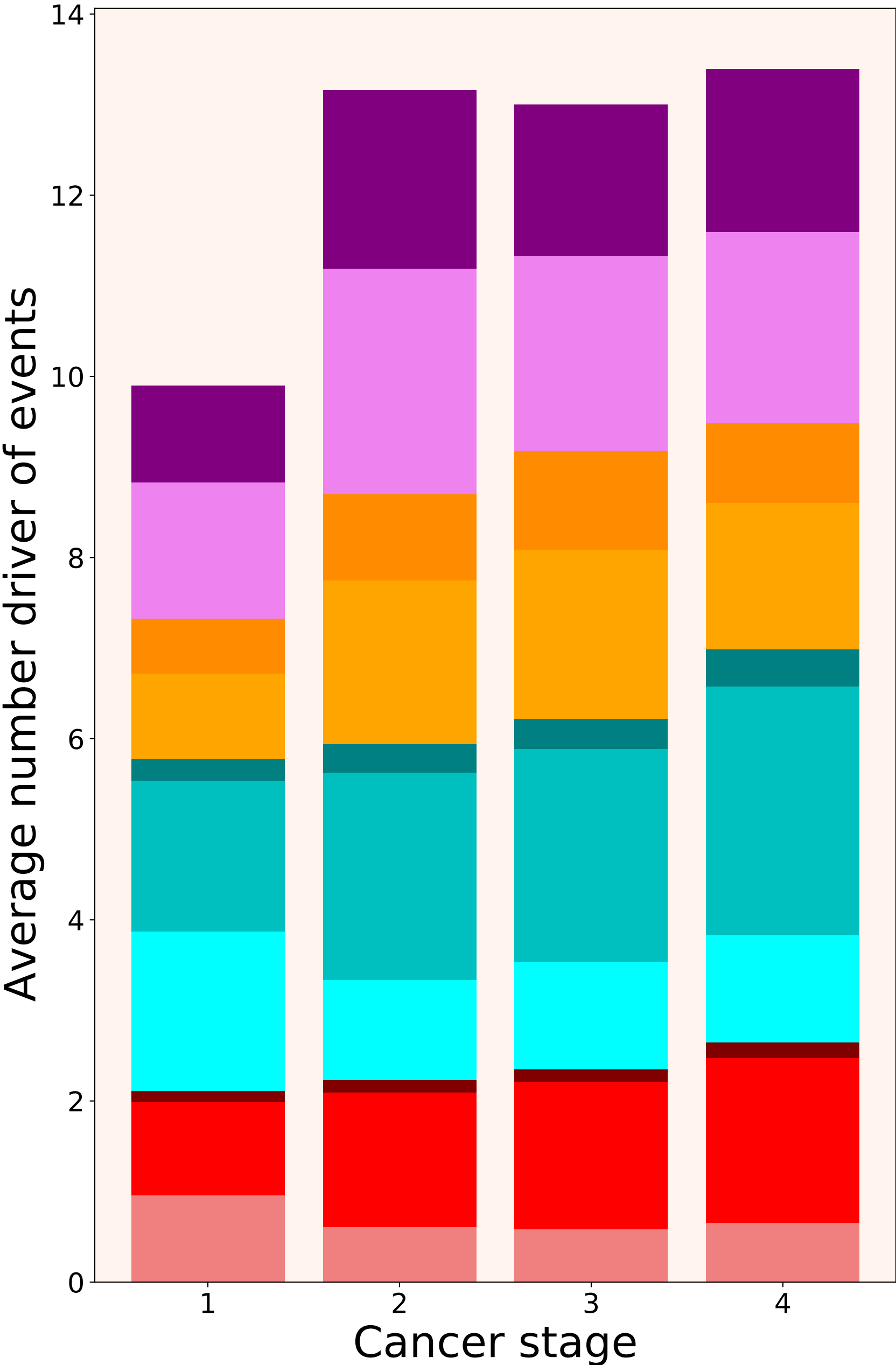

Supplement: S3 Files — (ZIP) [file pgen.1009996.s003.zip › COHORTS/cumulative histograms/2021_11_23_14_20_distribution_stages_females.pdf]

Driver event distribution by total number of driver events per patient in males

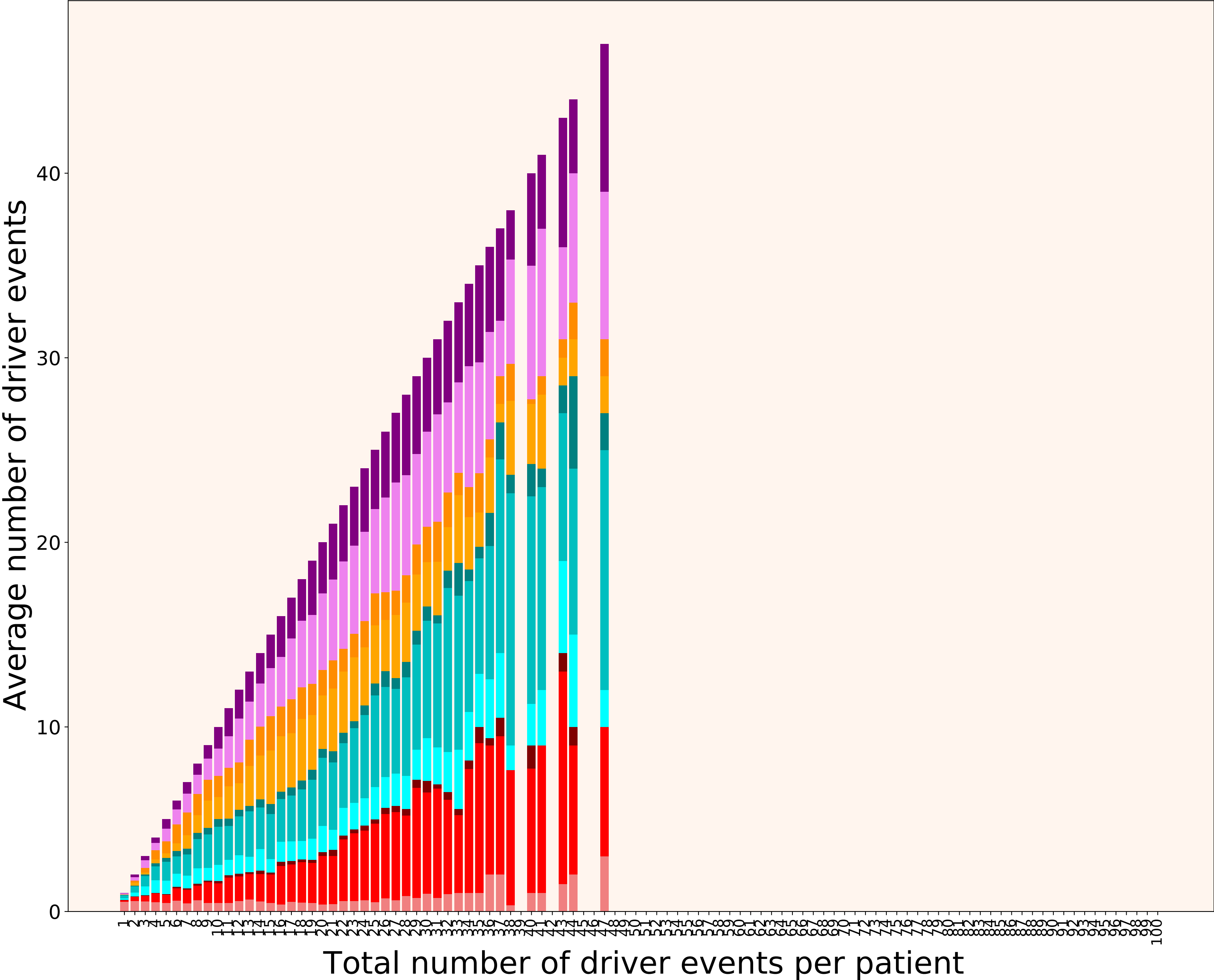

Supplement: S3 Files — (ZIP) [file pgen.1009996.s003.zip › COHORTS/cumulative histograms/2021_11_23_14_20_distribution_events_detailed_males.pdf]

Driver event distribution by cancer type

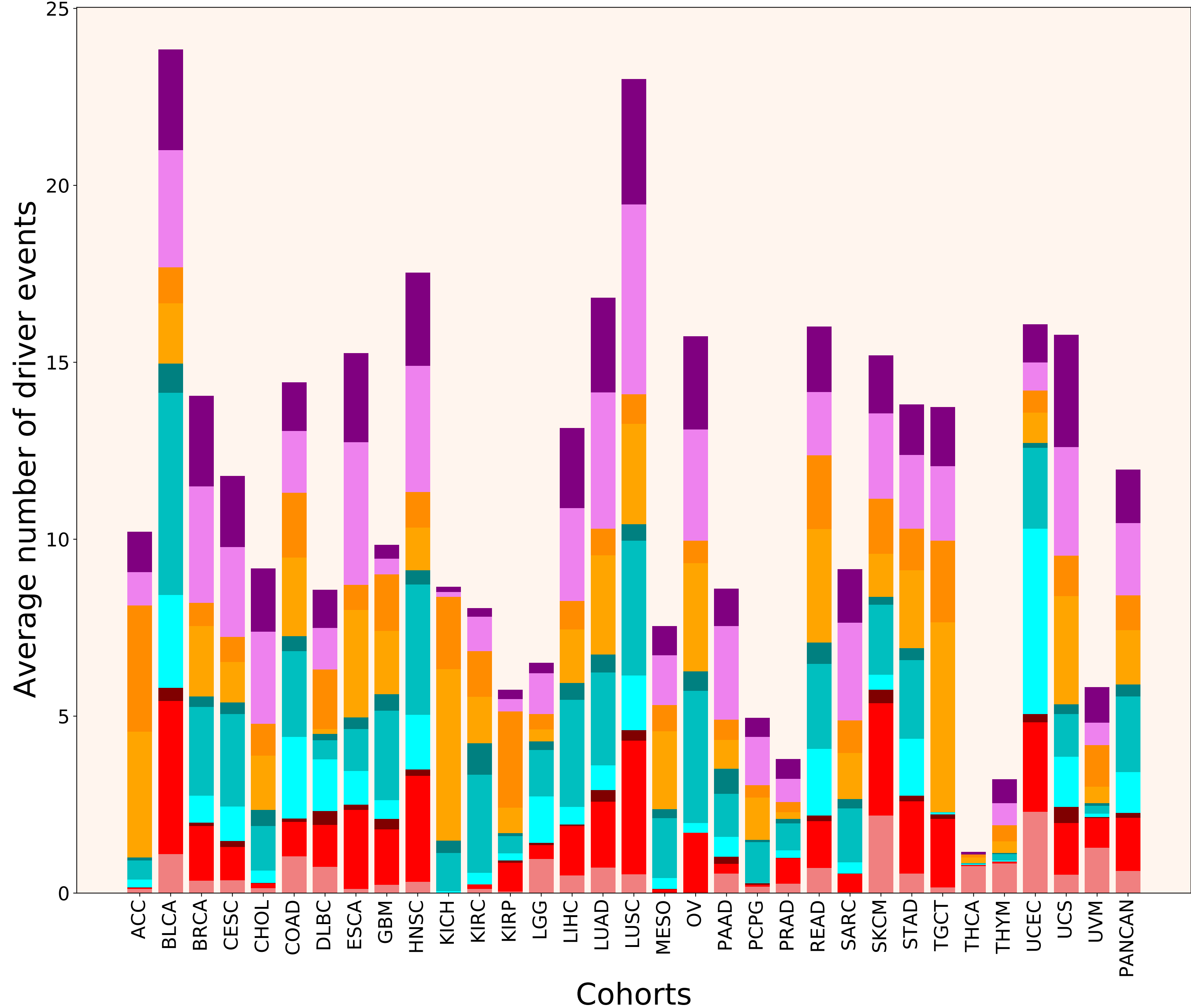

Supplement: S3 Files — (ZIP) [file pgen.1009996.s003.zip › COHORTS/cumulative histograms/2021_11_23_14_20_distribution_cohorts.pdf]

Driver event distribution by age in females

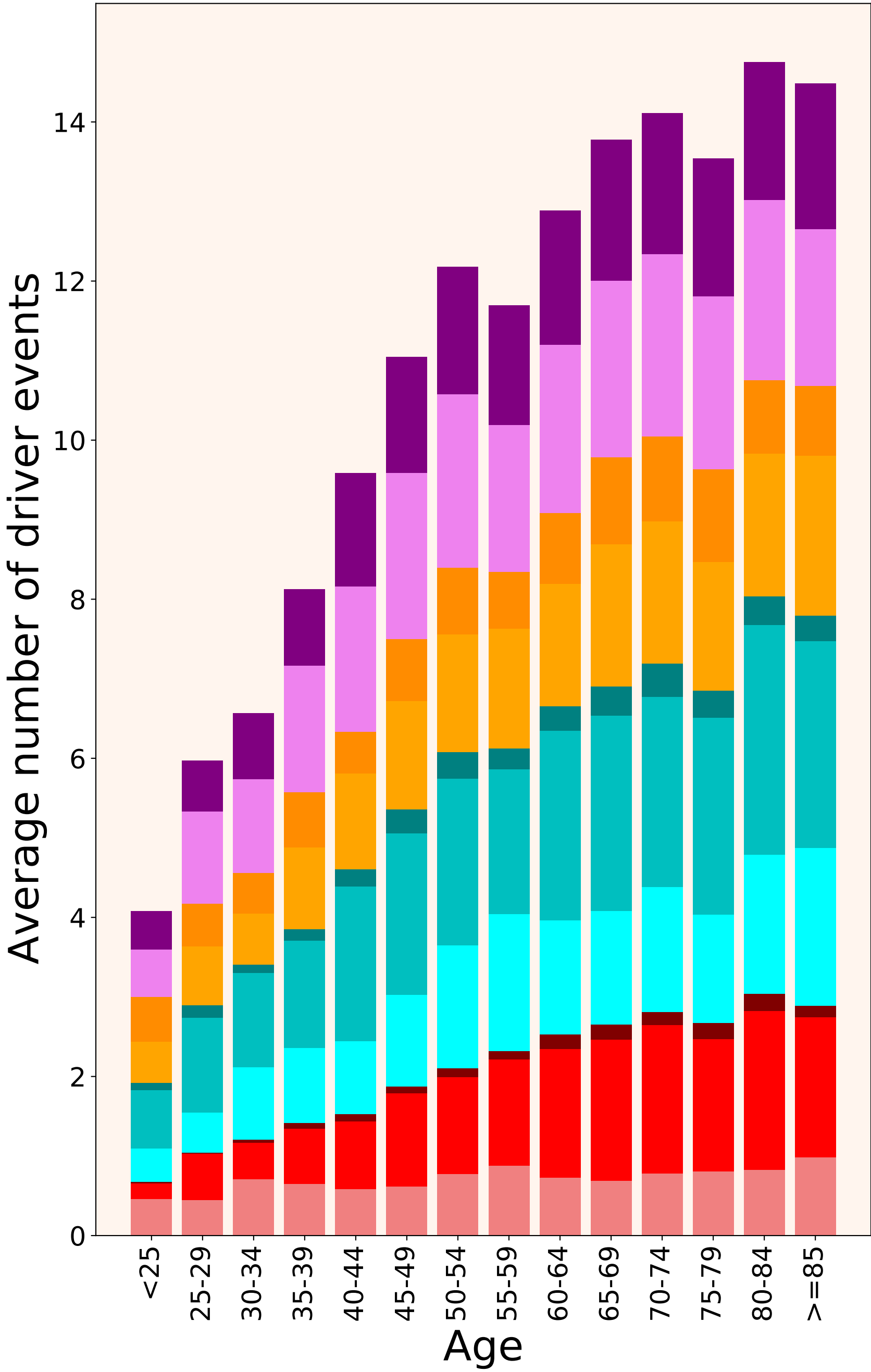

Supplement: S3 Files — (ZIP) [file pgen.1009996.s003.zip › COHORTS/cumulative histograms/2021_11_23_14_20_distribution_age_females.pdf]

Driver event distribution by cancer type in females

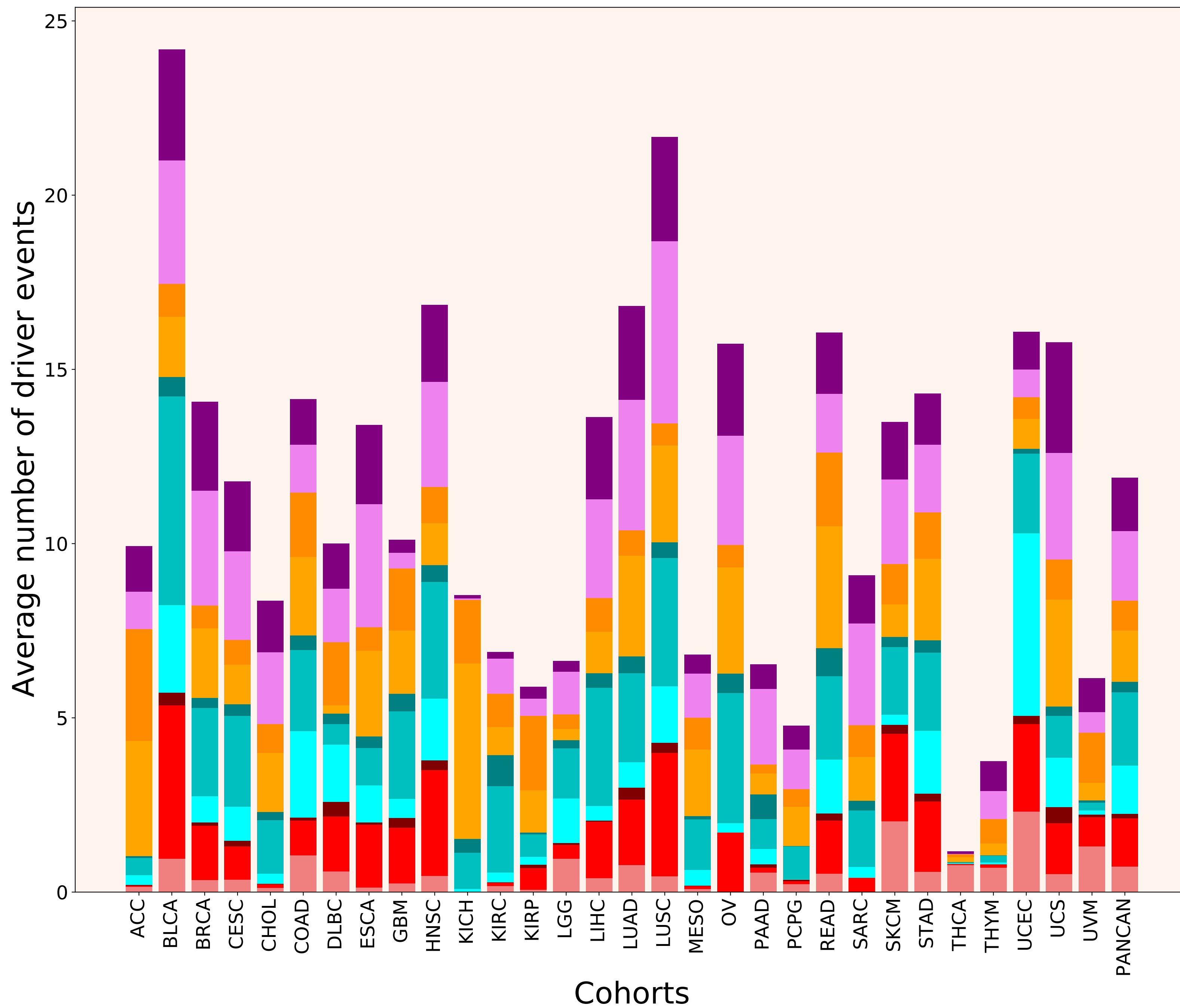

Supplement: S3 Files — (ZIP) [file pgen.1009996.s003.zip › COHORTS/cumulative histograms/2021_11_23_14_20_distribution_cohorts_females.pdf]

Driver event distribution by total number of driver events per patient in females

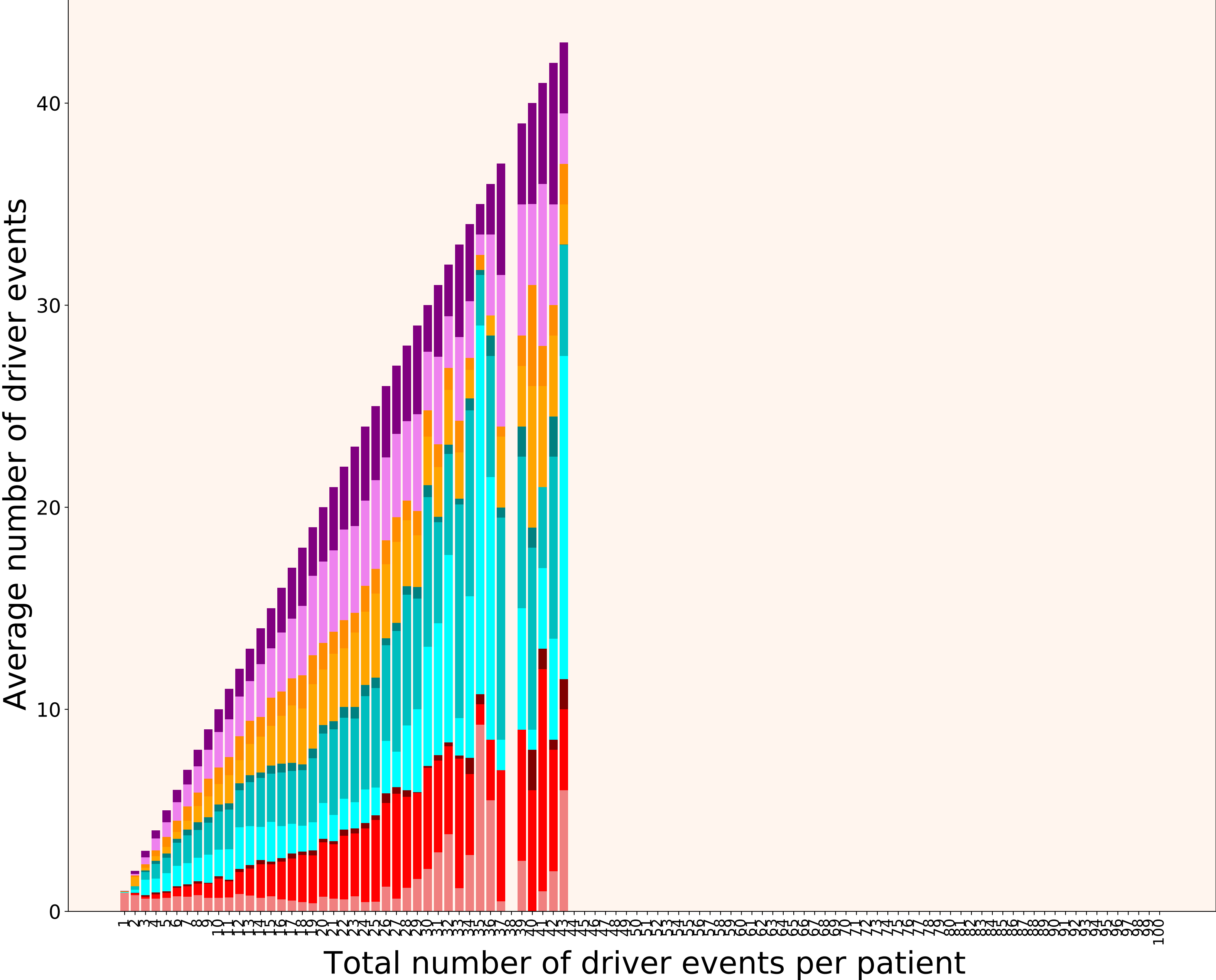

Supplement: S3 Files — (ZIP) [file pgen.1009996.s003.zip › COHORTS/cumulative histograms/2021_11_23_14_20_distribution_events_detailed_females.pdf]

Driver event distribution by cancer stage

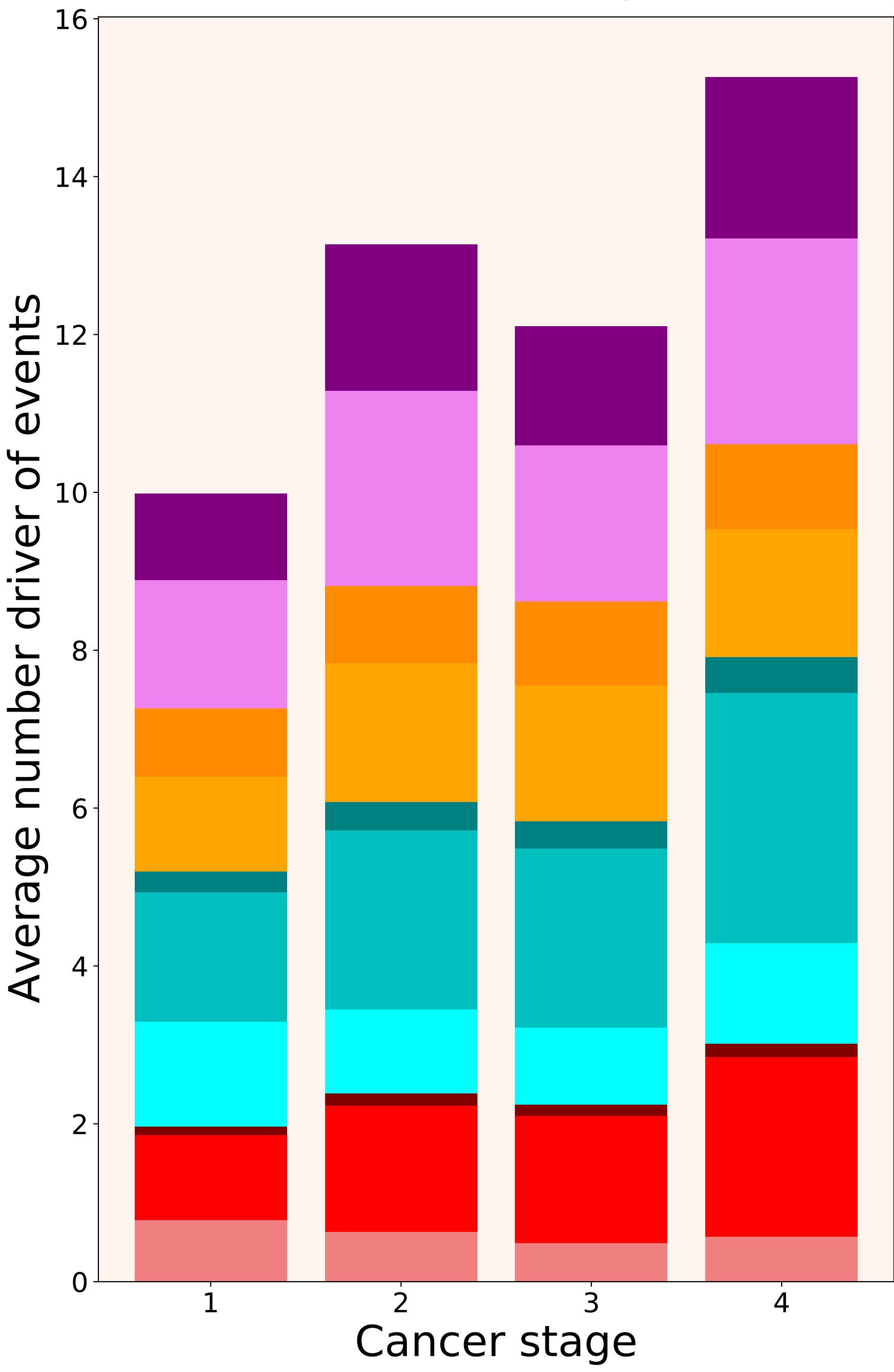

Supplement: S3 Files — (ZIP) [file pgen.1009996.s003.zip › COHORTS/cumulative histograms/2021_11_23_14_20_distribution_stages.pdf]

Driver event distribution by gender

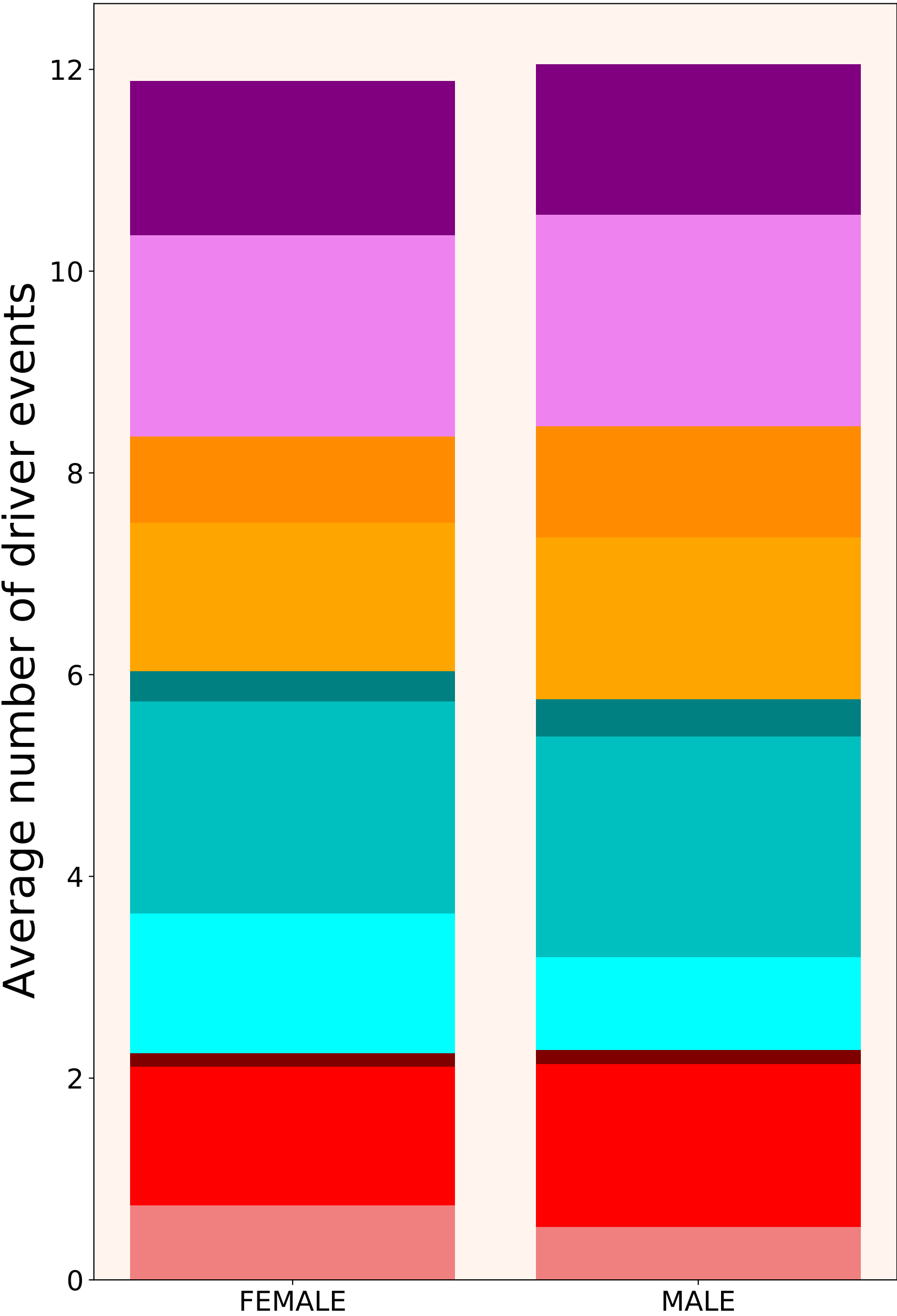

Supplement: S3 Files — (ZIP) [file pgen.1009996.s003.zip › COHORTS/cumulative histograms/2021_11_23_14_20_distribution_gender.pdf]

# PAAD\_MALE

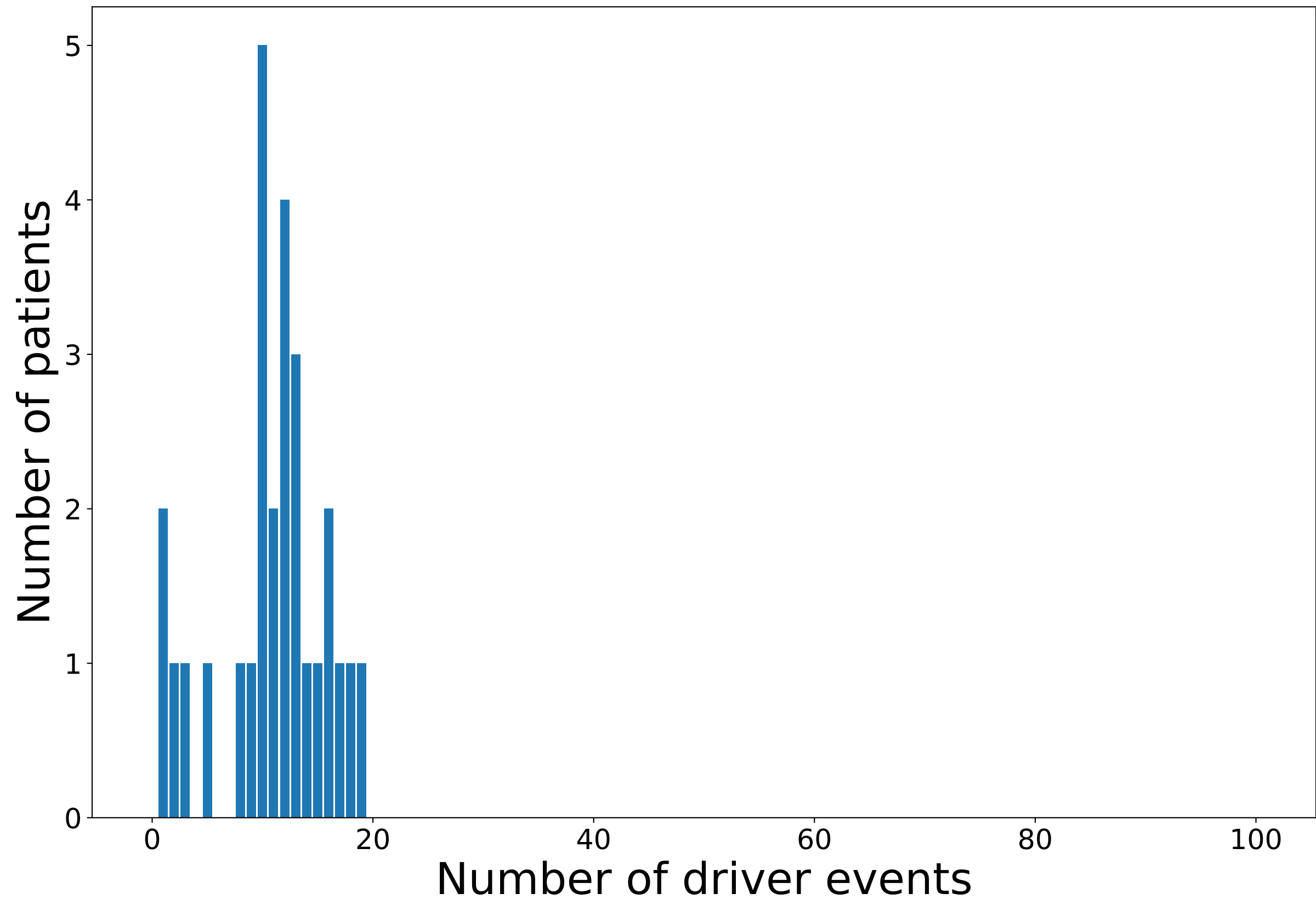

Supplement: S3 Files — (ZIP) [file pgen.1009996.s003.zip › COHORTS/patient distributions/2021_11_23_14_20_PAAD_MALE.pdf]

# LIHC\_FEMALE

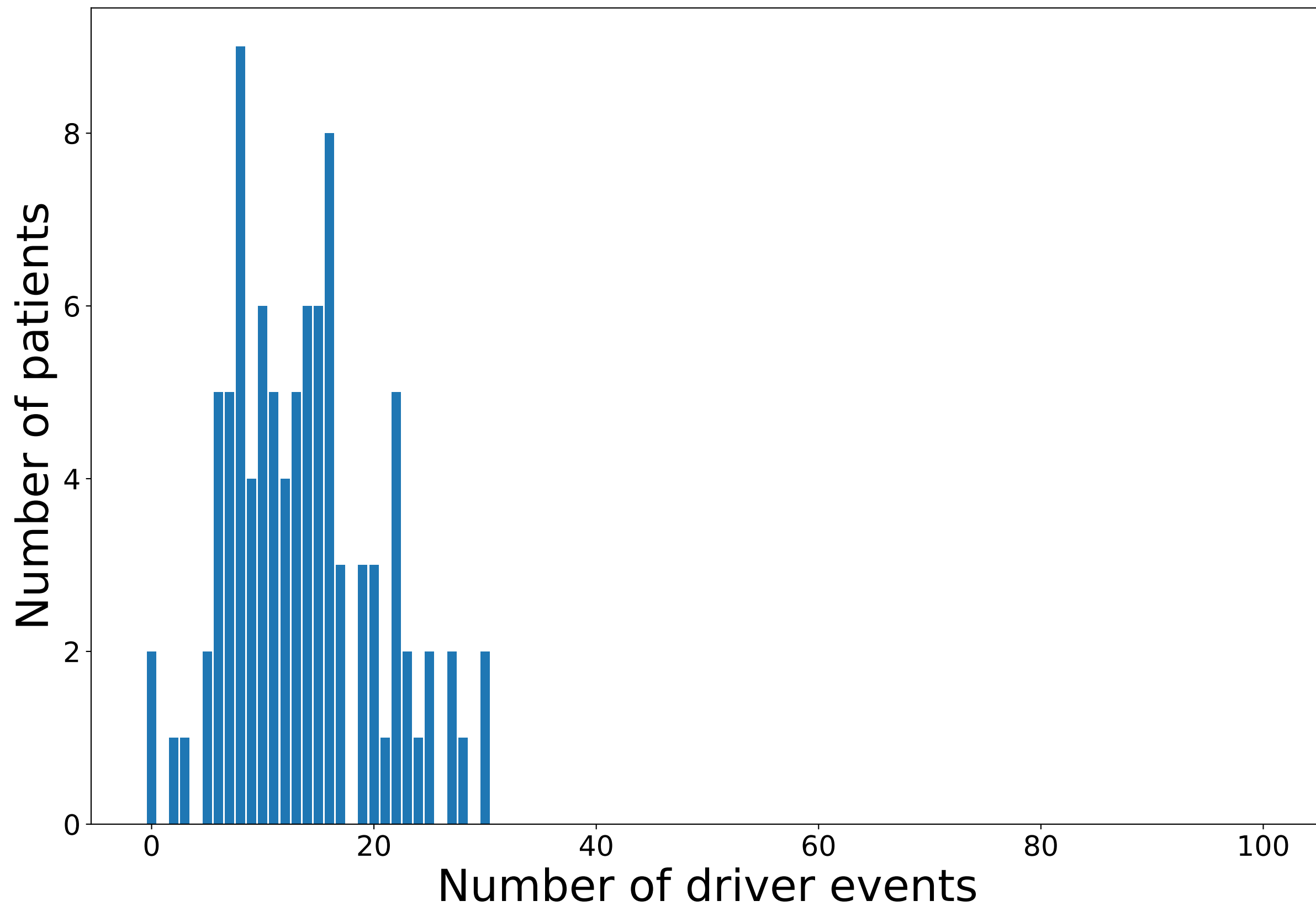

Supplement: S3 Files — (ZIP) [file pgen.1009996.s003.zip › COHORTS/patient distributions/2021_11_23_14_20_LIHC_FEMALE.pdf]

# KIRC\_FEMALE

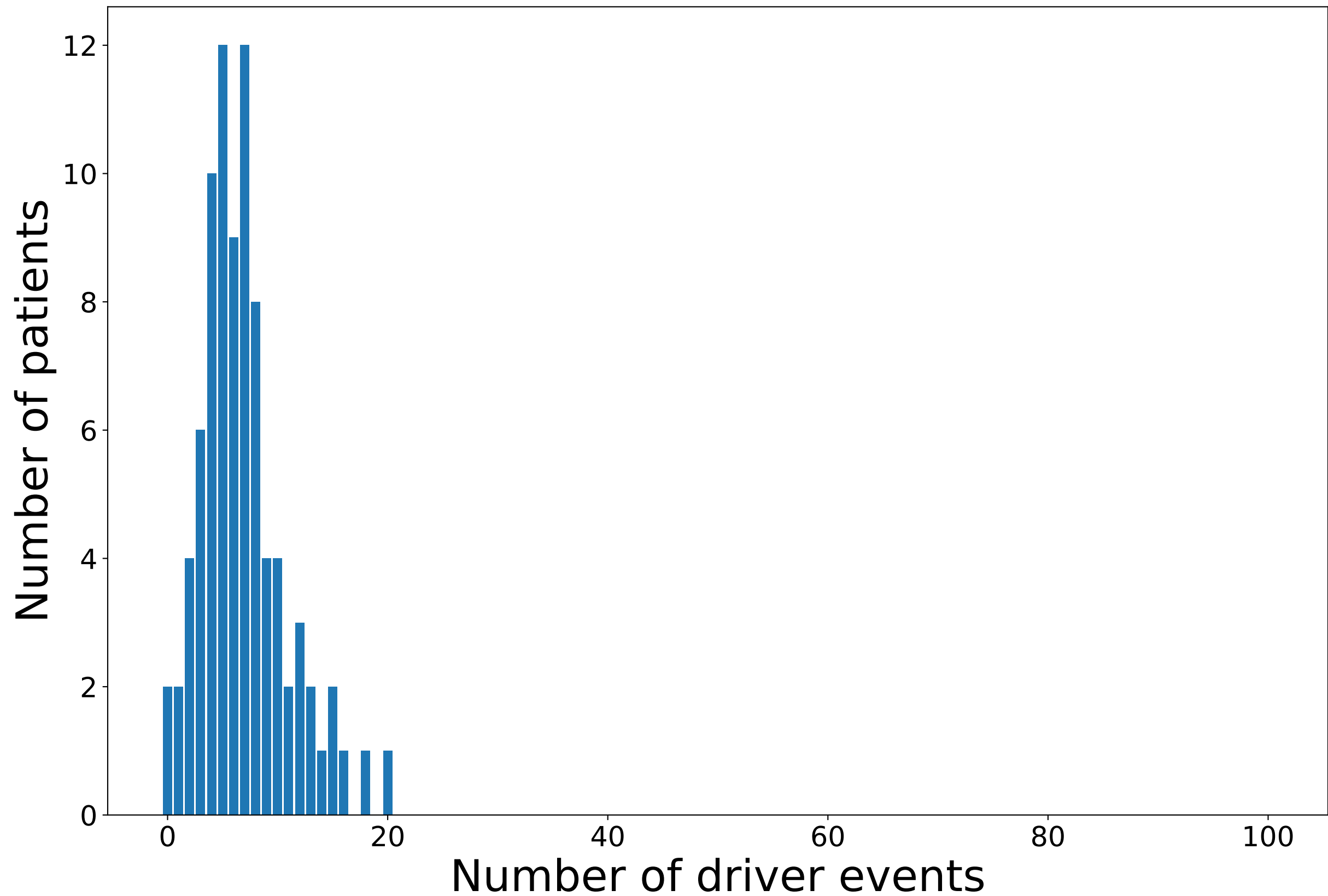

Supplement: S3 Files — (ZIP) [file pgen.1009996.s003.zip › COHORTS/patient distributions/2021_11_23_14_20_KIRC_FEMALE.pdf]

# LGG\_MALE

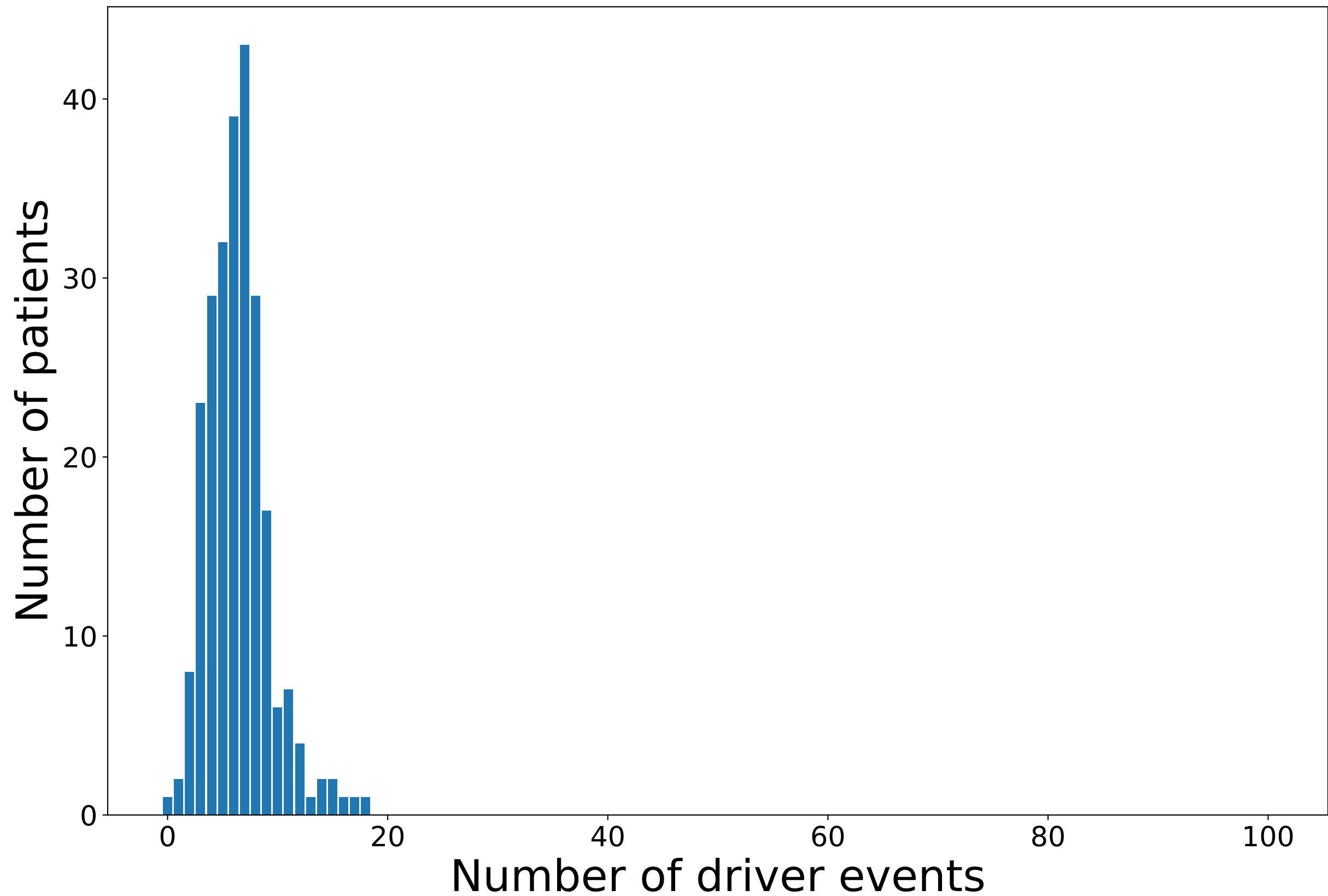

Supplement: S3 Files — (ZIP) [file pgen.1009996.s003.zip › COHORTS/patient distributions/2021_11_23_14_20_LGG_MALE.pdf]

# UVM

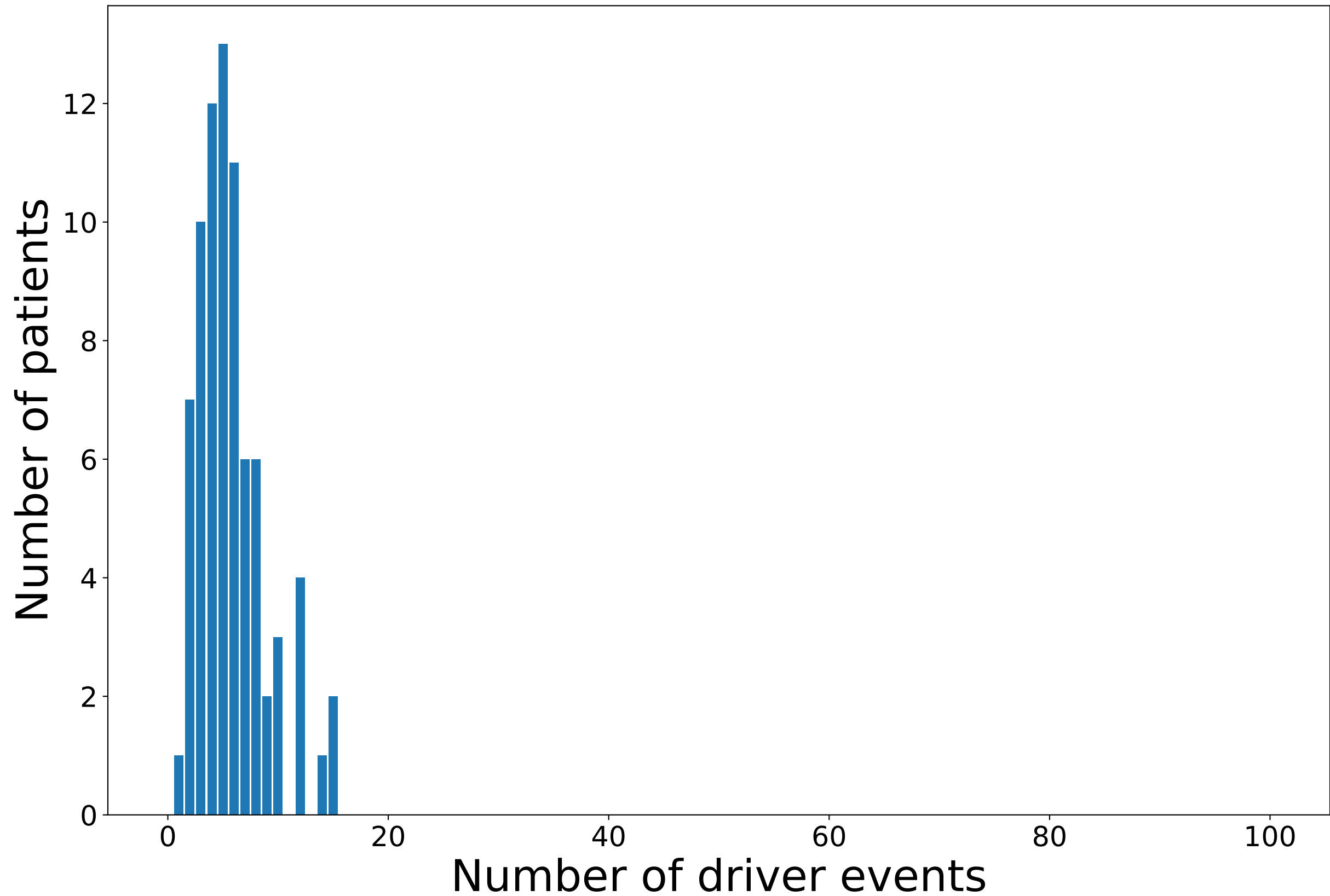

Supplement: S3 Files — (ZIP) [file pgen.1009996.s003.zip › COHORTS/patient distributions/2021_11_23_14_20_UVM.pdf]

# BLCA\_FEMALE

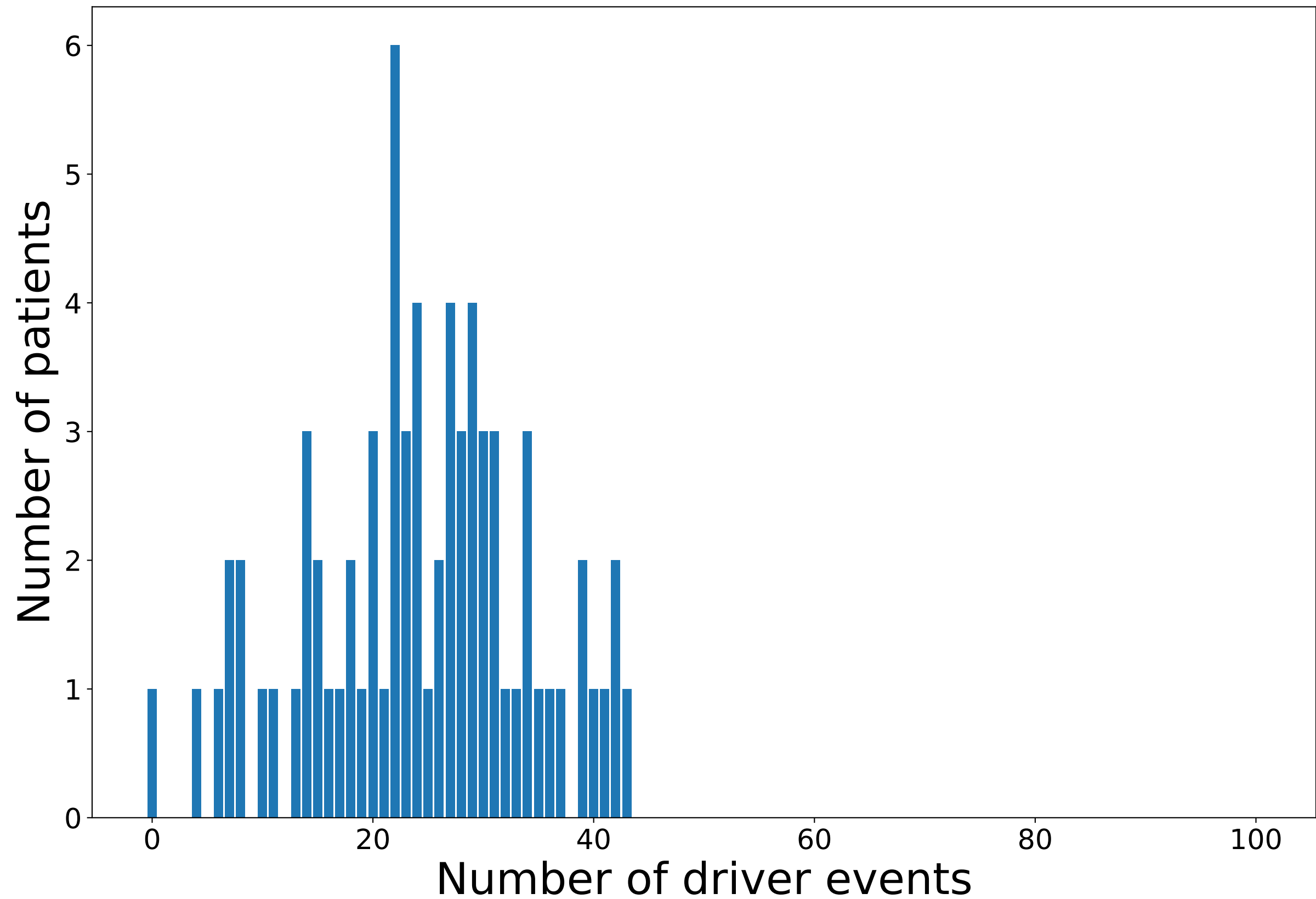

Supplement: S3 Files — (ZIP) [file pgen.1009996.s003.zip › COHORTS/patient distributions/2021_11_23_14_20_BLCA_FEMALE.pdf]

# CESC

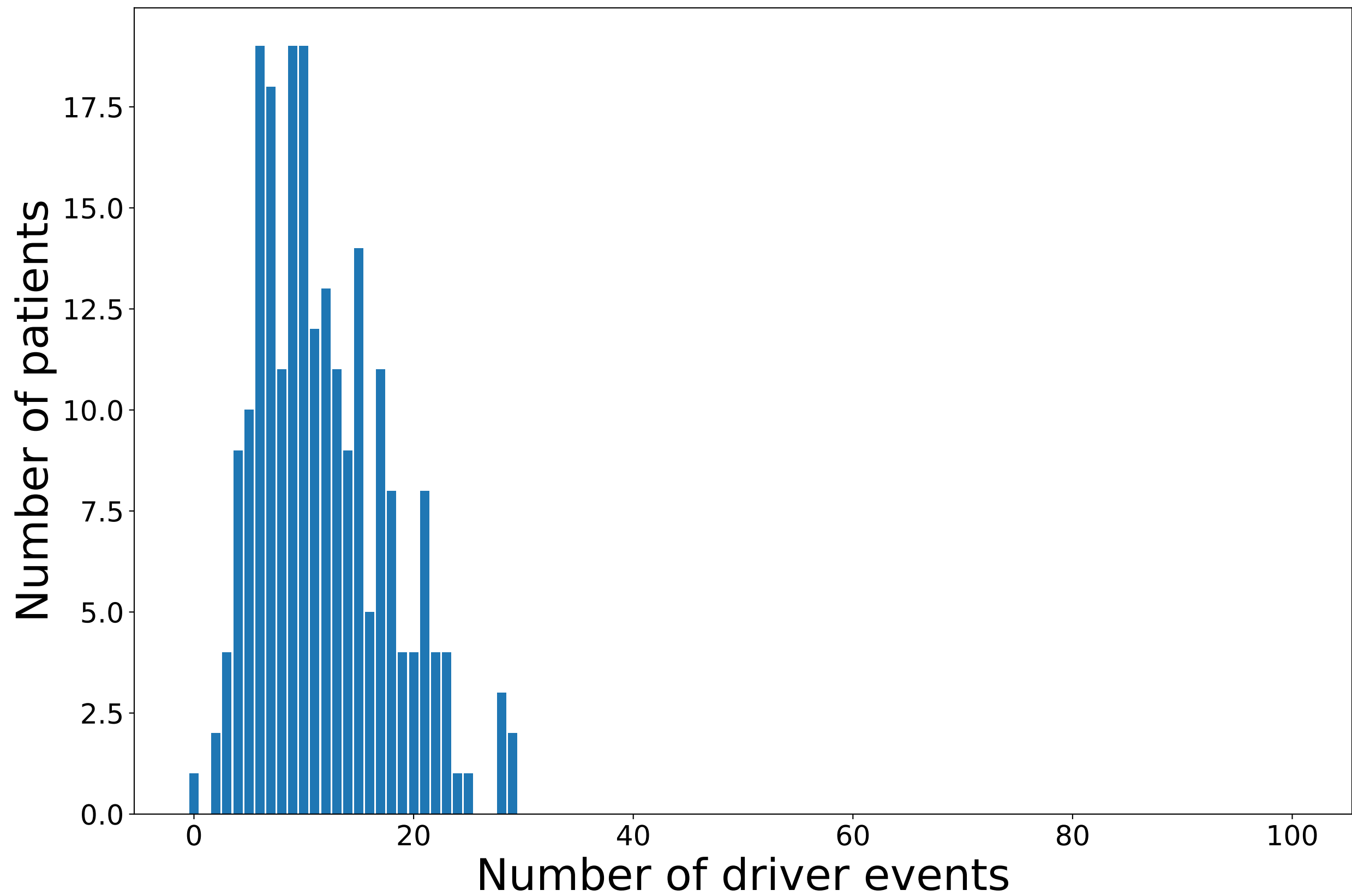

Supplement: S3 Files — (ZIP) [file pgen.1009996.s003.zip › COHORTS/patient distributions/2021_11_23_14_20_CESC.pdf]

# LUAD\_MALE

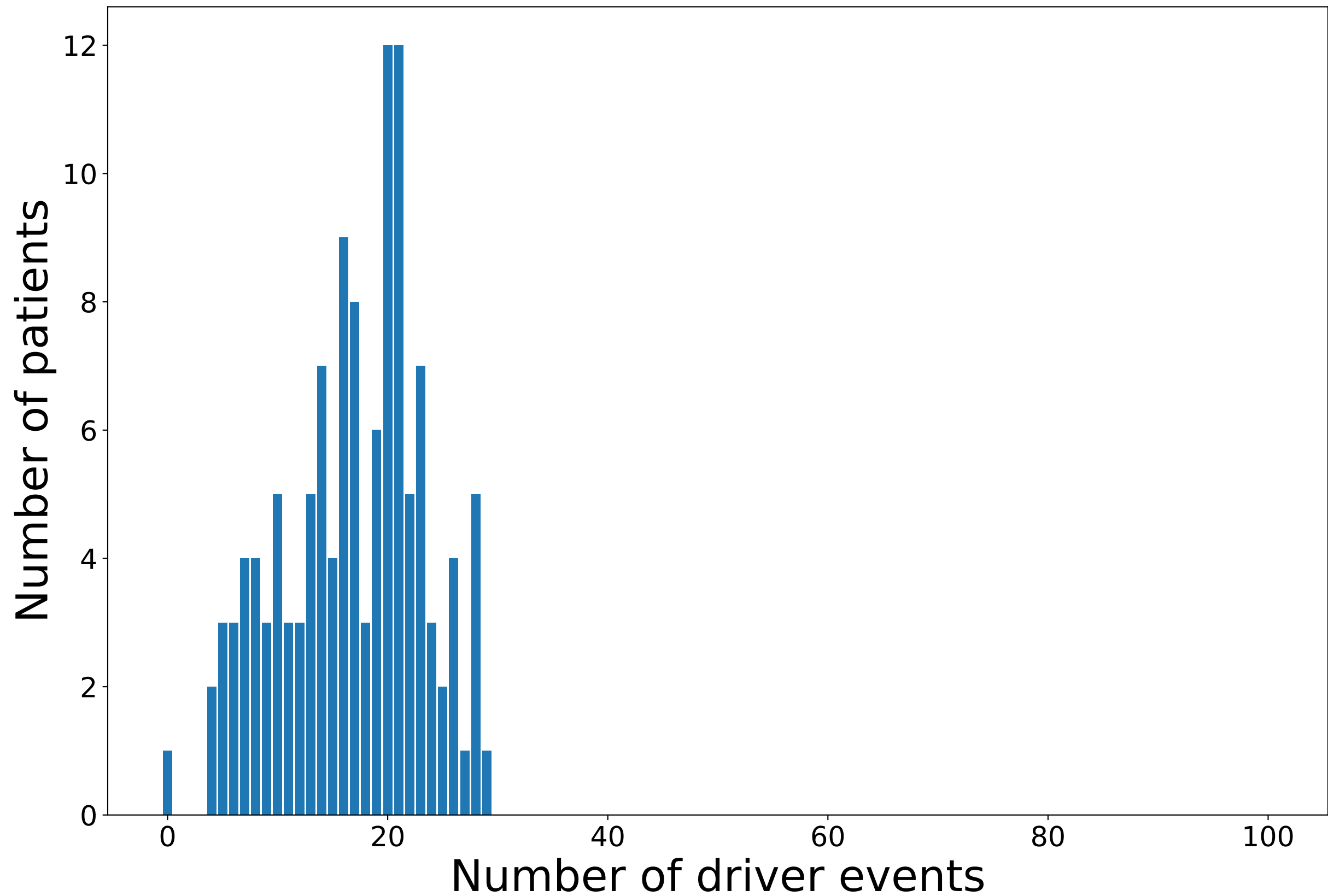

Supplement: S3 Files — (ZIP) [file pgen.1009996.s003.zip › COHORTS/patient distributions/2021_11_23_14_20_LUAD_MALE.pdf]
