# Supplementary figures and images for: Comprehensive patient-level classification and quantification of driver events in TCGA PanCanAtlas cohorts (part 1 of 6)
Source: PLoS Genet. 2022 Jan 14;18(1):e1009996. doi: 10.1371/journal.pgen.1009996 (PMC8759692; doi:10.1371/journal.pgen.1009996)

Driver event distribution by age in males

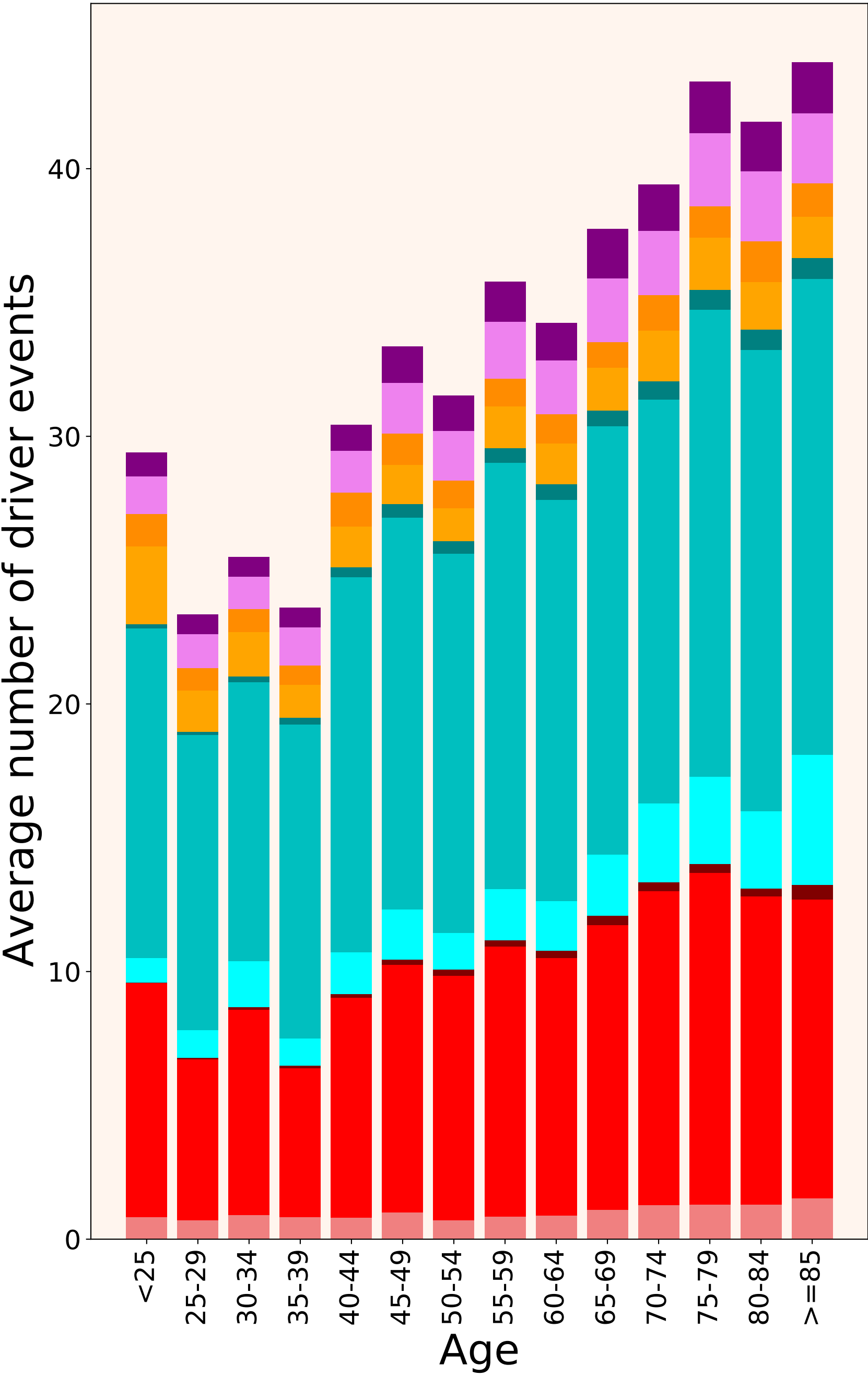

Supplement: S2 Files — (ZIP) [file pgen.1009996.s002.zip › PANCAN/cumulative histograms/2021_11_23_14_43_distribution_age_males.pdf]

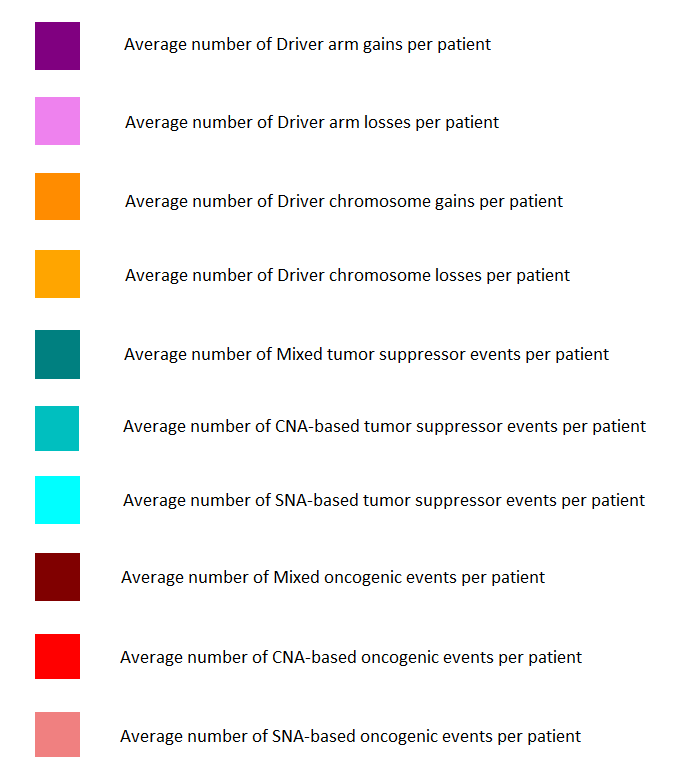

Supplement: S2 Files — (ZIP) [file pgen.1009996.s002.zip › PANCAN/cumulative histograms/legeng.png]

Driver event distribution by cancer stage in females

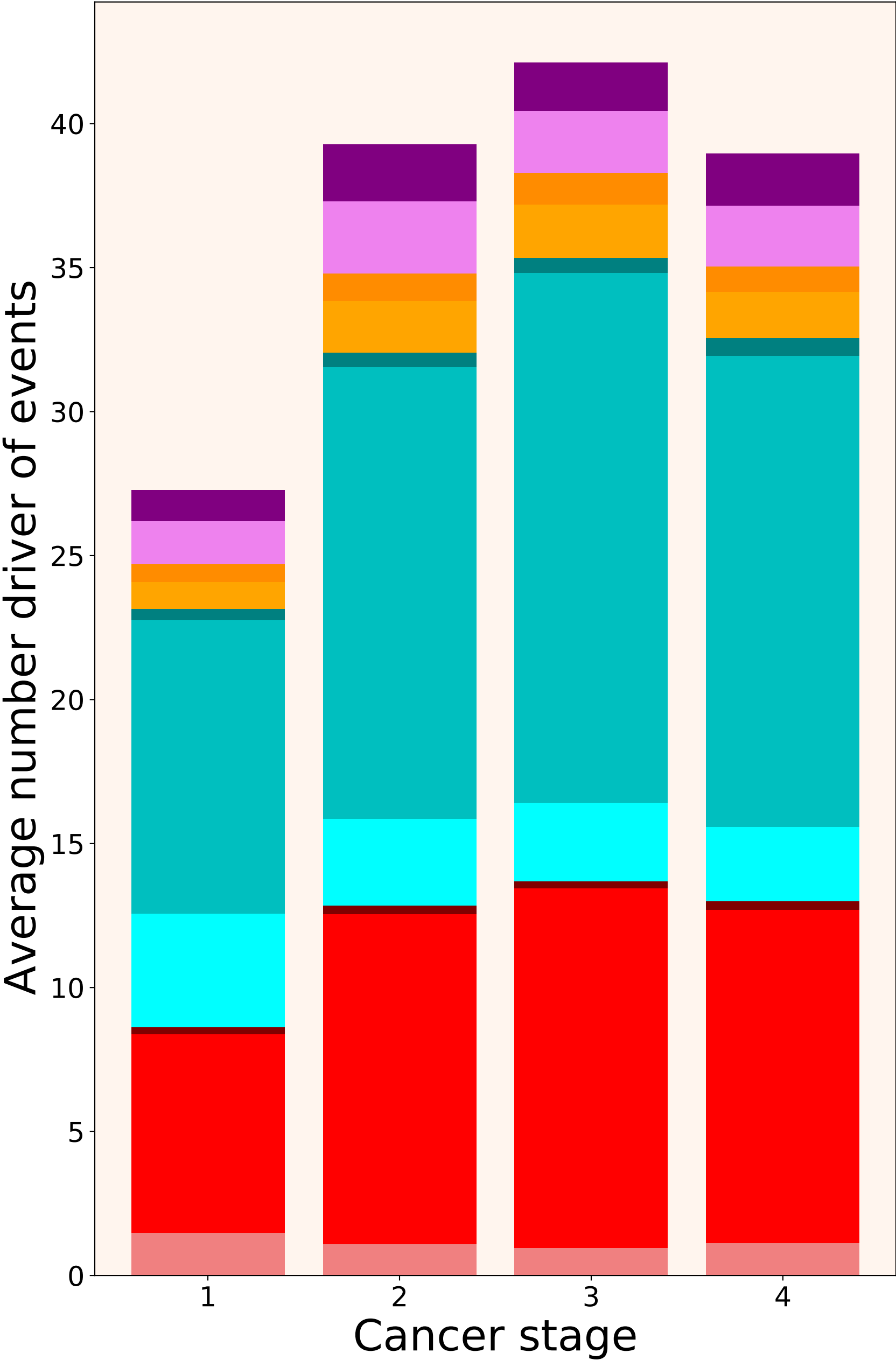

Supplement: S2 Files — (ZIP) [file pgen.1009996.s002.zip › PANCAN/cumulative histograms/2021_11_23_14_43_distribution_stages_females.pdf]

Driver event distribution by cancer stage

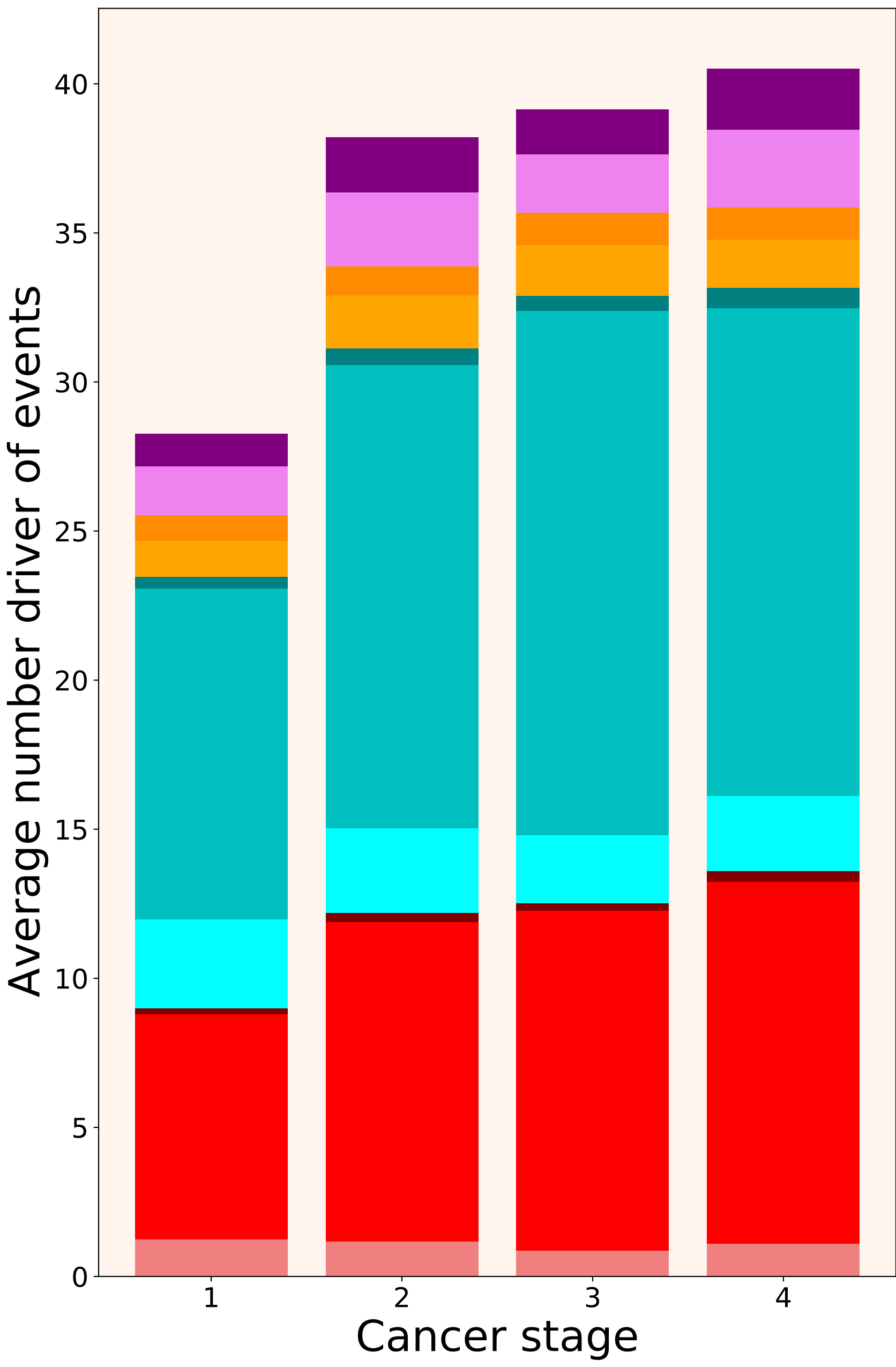

Supplement: S2 Files — (ZIP) [file pgen.1009996.s002.zip › PANCAN/cumulative histograms/2021_11_23_14_43_distribution_stages.pdf]

Driver event distribution by gender

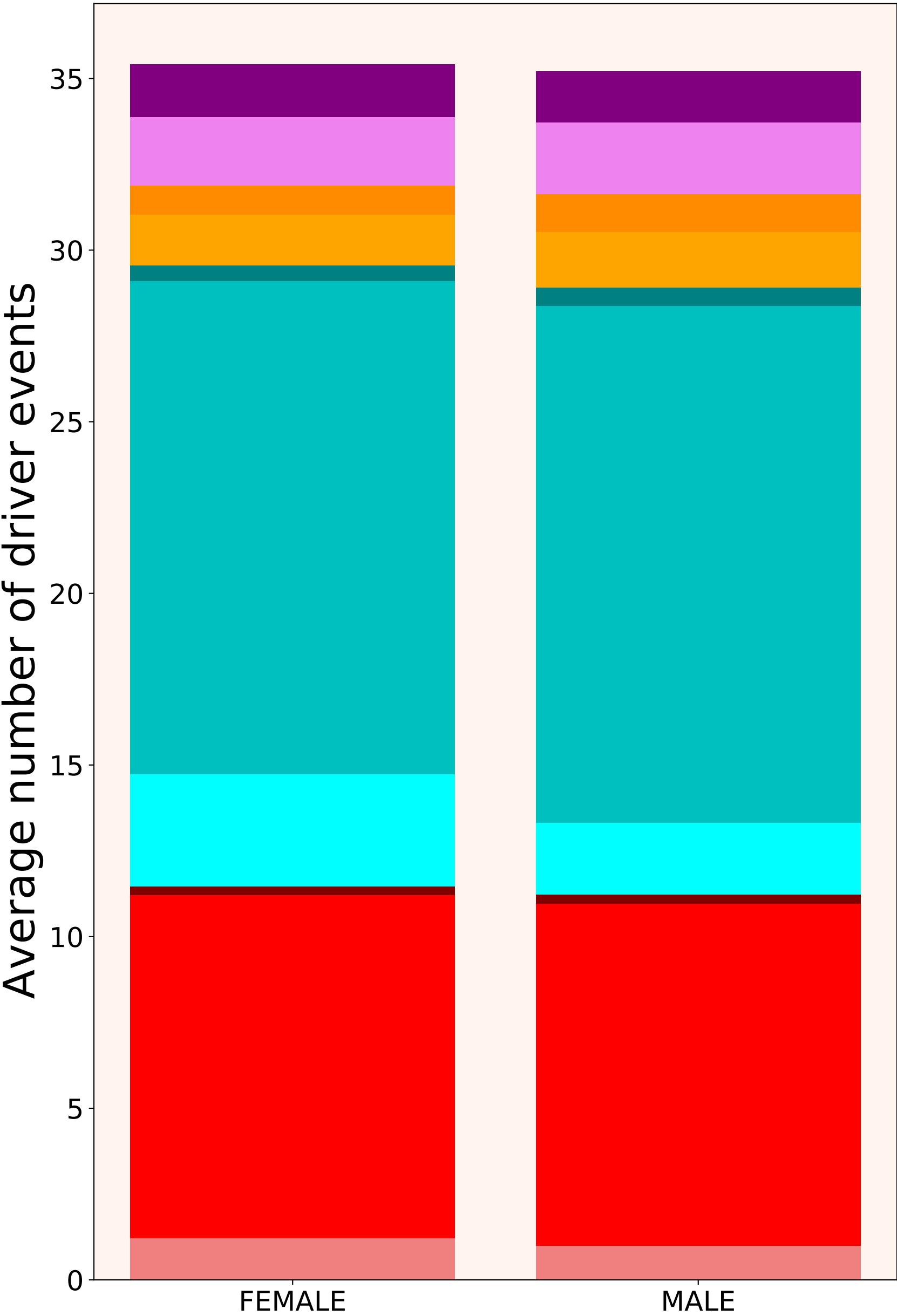

Supplement: S2 Files — (ZIP) [file pgen.1009996.s002.zip › PANCAN/cumulative histograms/2021_11_23_14_43_distribution_gender.pdf]

Driver event distribution by cancer type in females

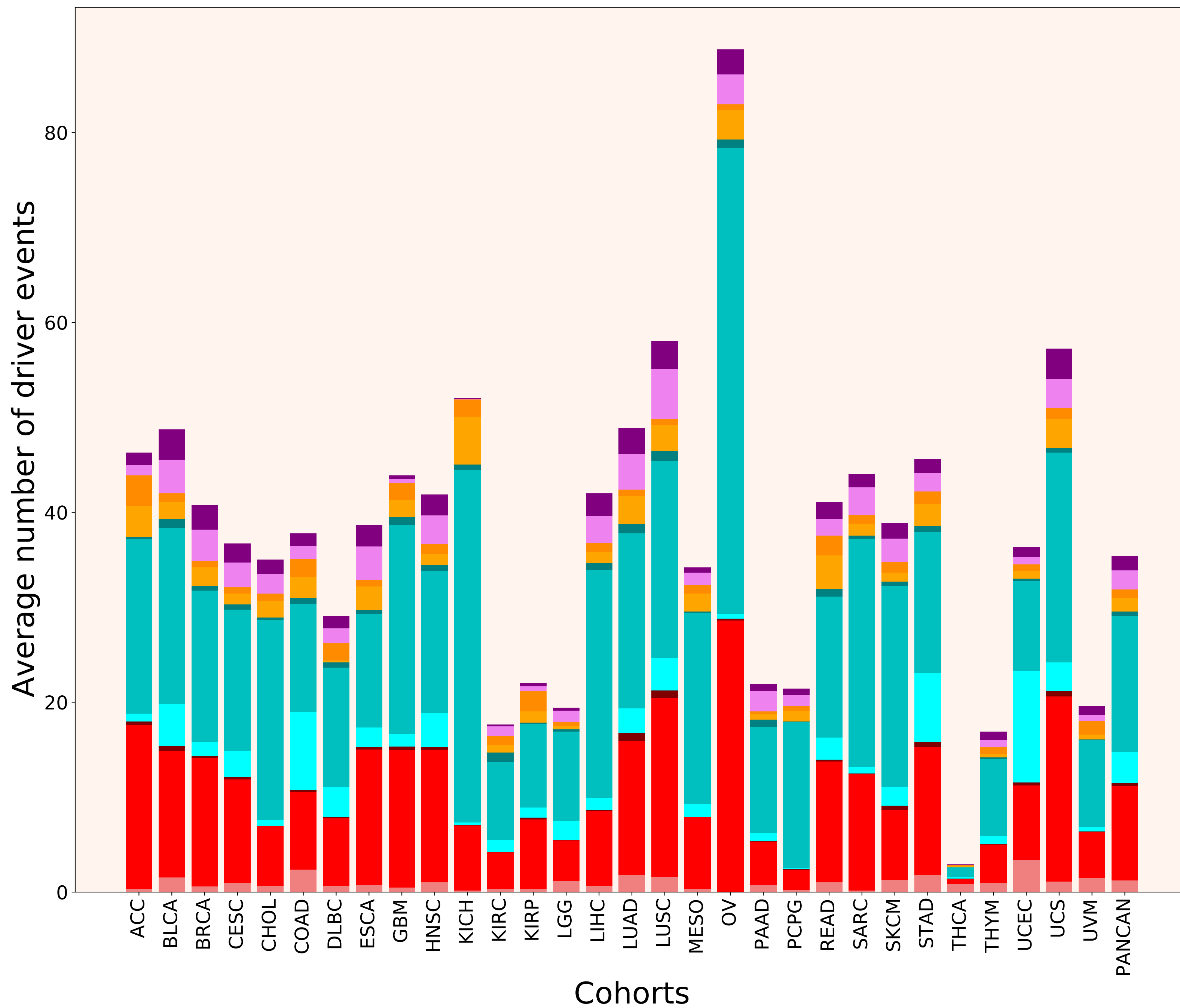

Supplement: S2 Files — (ZIP) [file pgen.1009996.s002.zip › PANCAN/cumulative histograms/2021_11_23_14_43_distribution_cohorts_females.pdf]

Driver event distribution by cancer type in males

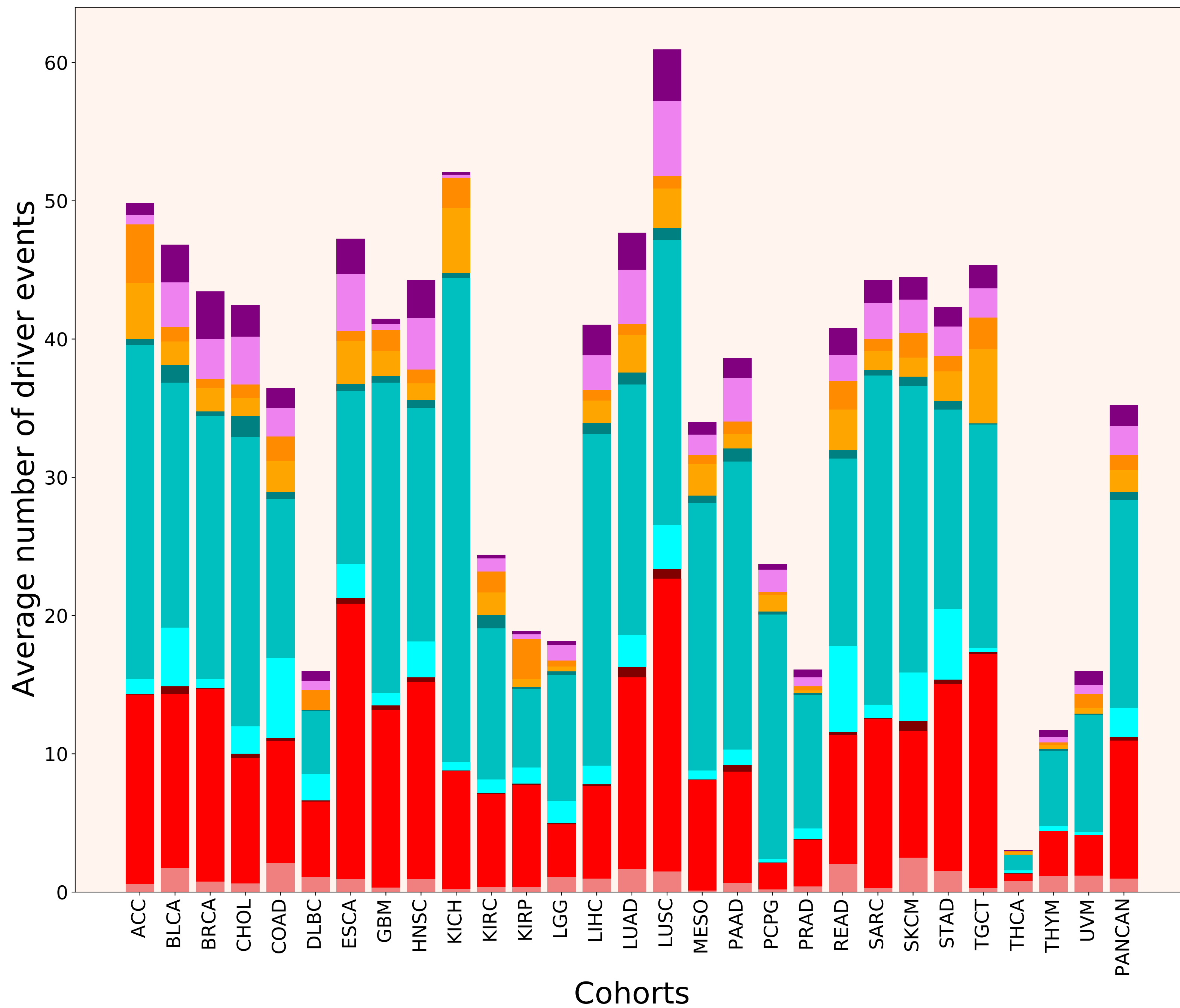

Supplement: S2 Files — (ZIP) [file pgen.1009996.s002.zip › PANCAN/cumulative histograms/2021_11_23_14_43_distribution_cohorts_males.pdf]

Driver event distribution by total number of driver events per patient in females

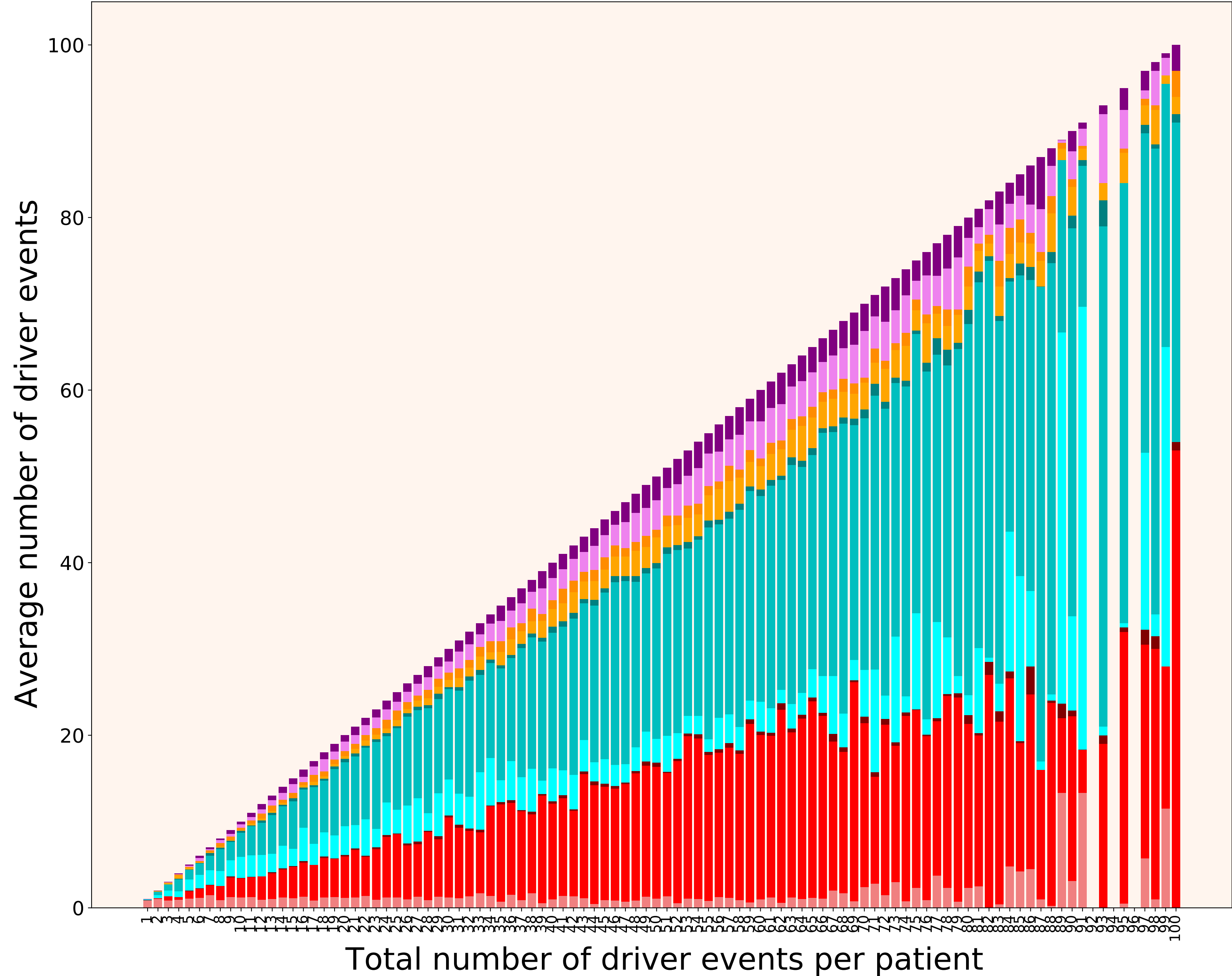

Supplement: S2 Files — (ZIP) [file pgen.1009996.s002.zip › PANCAN/cumulative histograms/2021_11_23_14_43_distribution_events_detailed_females.pdf]

Driver event distribution by age

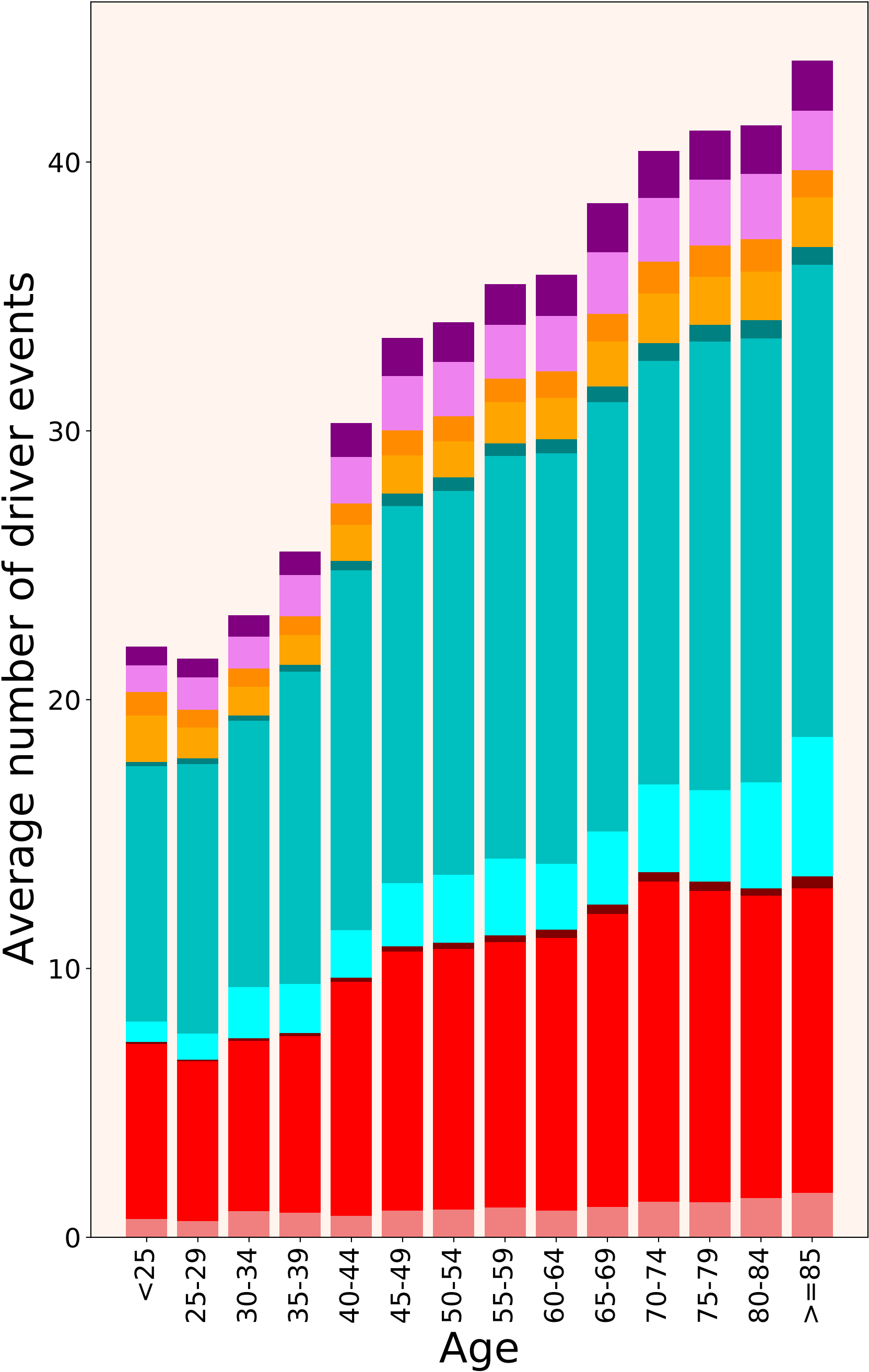

Supplement: S2 Files — (ZIP) [file pgen.1009996.s002.zip › PANCAN/cumulative histograms/2021_11_23_14_43_distribution_age.pdf]

Driver event distribution by cancer stage in males

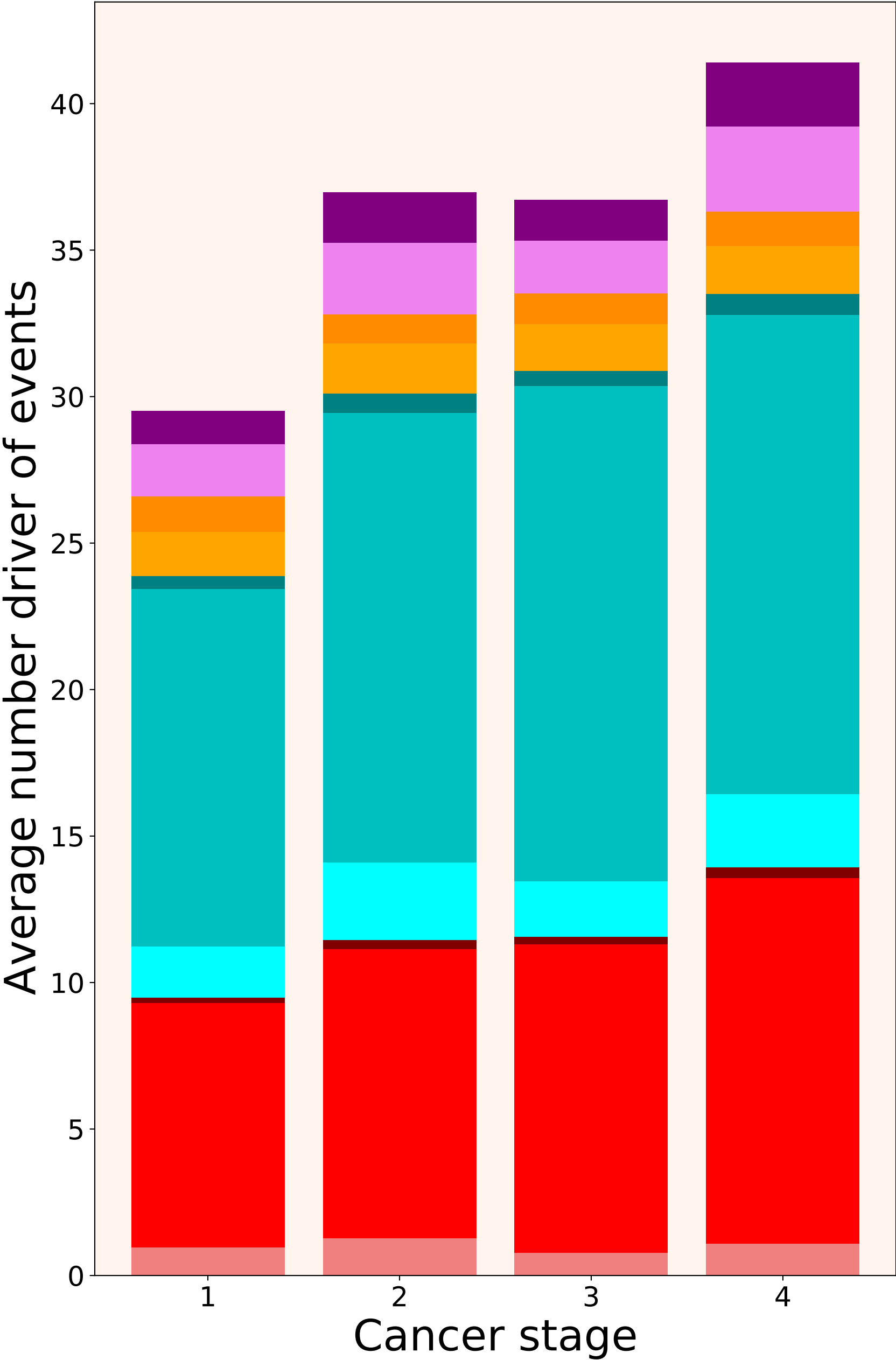

Supplement: S2 Files — (ZIP) [file pgen.1009996.s002.zip › PANCAN/cumulative histograms/2021_11_23_14_43_distribution_stages_males.pdf]

Driver event distribution by cancer type

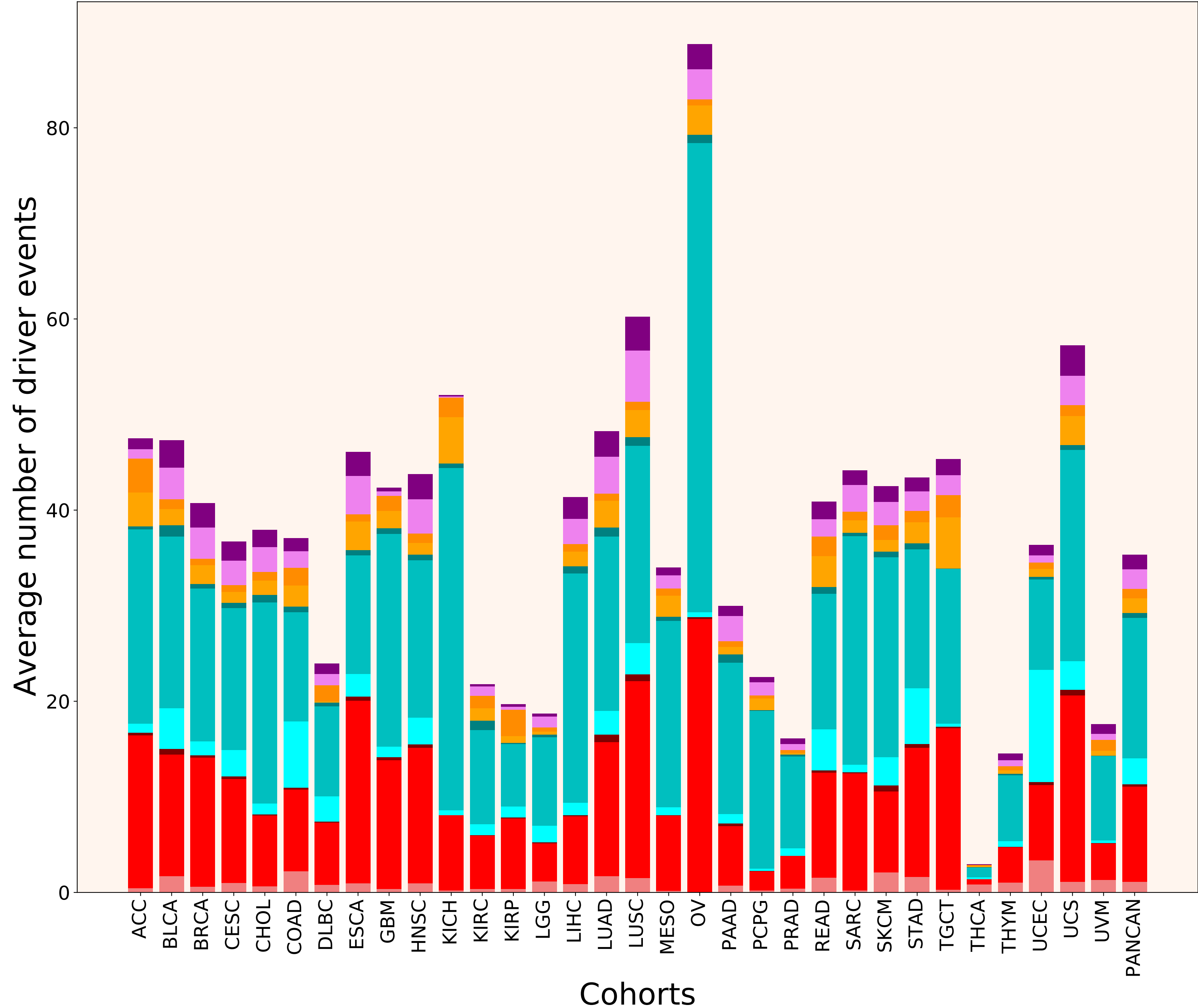

Supplement: S2 Files — (ZIP) [file pgen.1009996.s002.zip › PANCAN/cumulative histograms/2021_11_23_14_43_distribution_cohorts.pdf]

Driver event distribution by total number of driver events per patient

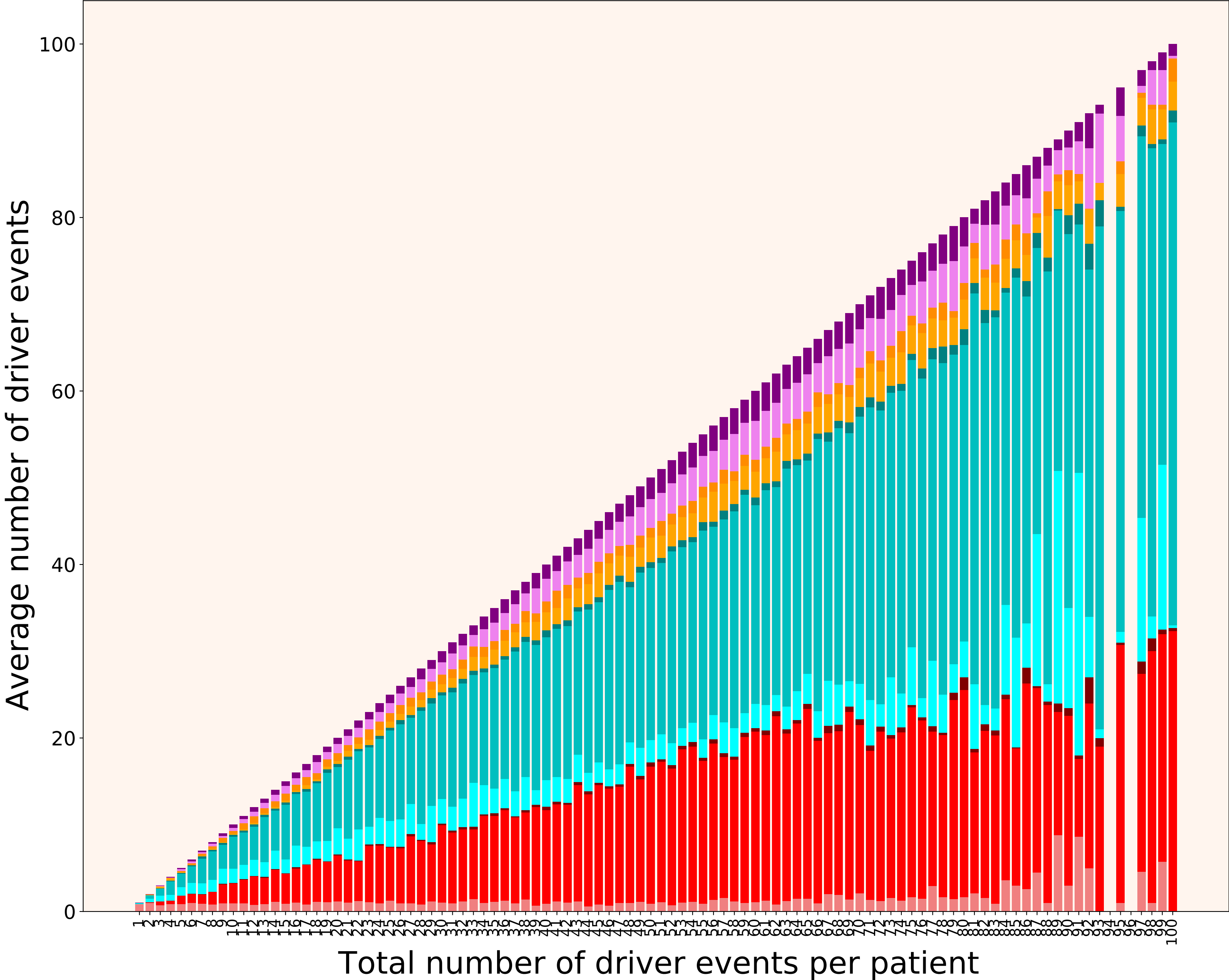

Supplement: S2 Files — (ZIP) [file pgen.1009996.s002.zip › PANCAN/cumulative histograms/2021_11_23_14_43_distribution_events_detailed.pdf]

Driver event distribution by total number of driver events per patient in males

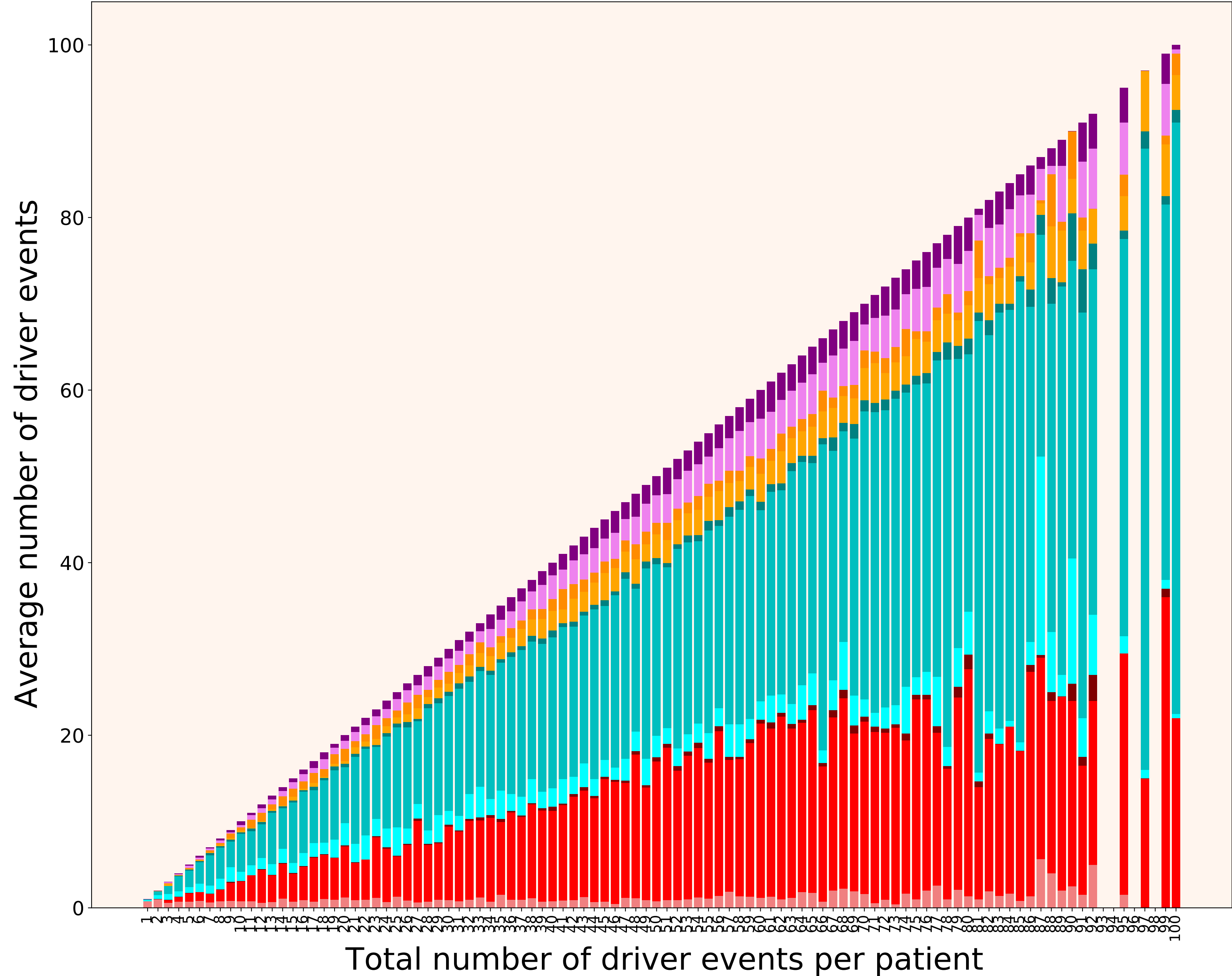

Supplement: S2 Files — (ZIP) [file pgen.1009996.s002.zip › PANCAN/cumulative histograms/2021_11_23_14_43_distribution_events_detailed_males.pdf]

Driver event distribution by age in females

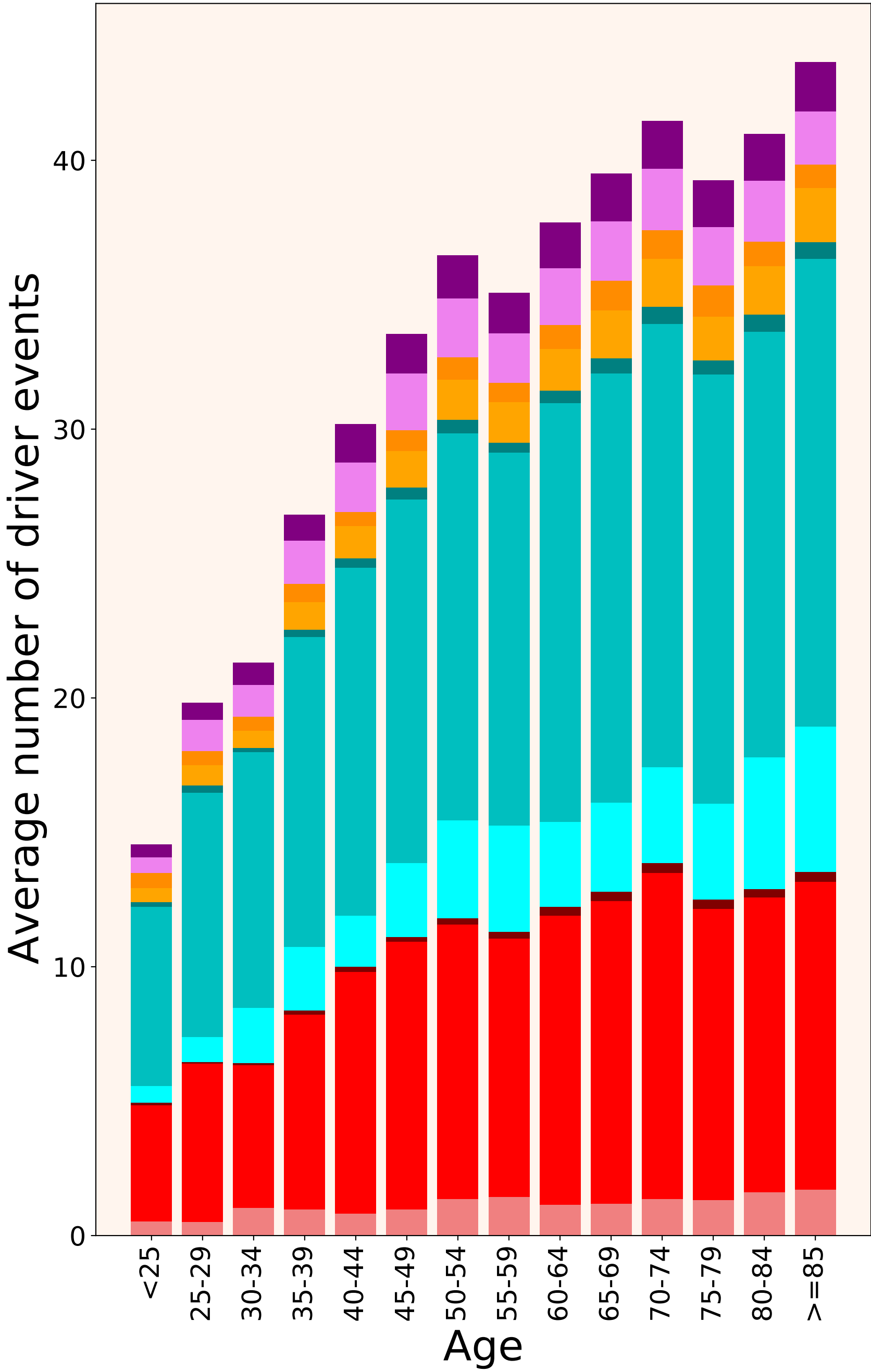

Supplement: S2 Files — (ZIP) [file pgen.1009996.s002.zip › PANCAN/cumulative histograms/2021_11_23_14_43_distribution_age_females.pdf]

# BLCA\_FEMALE

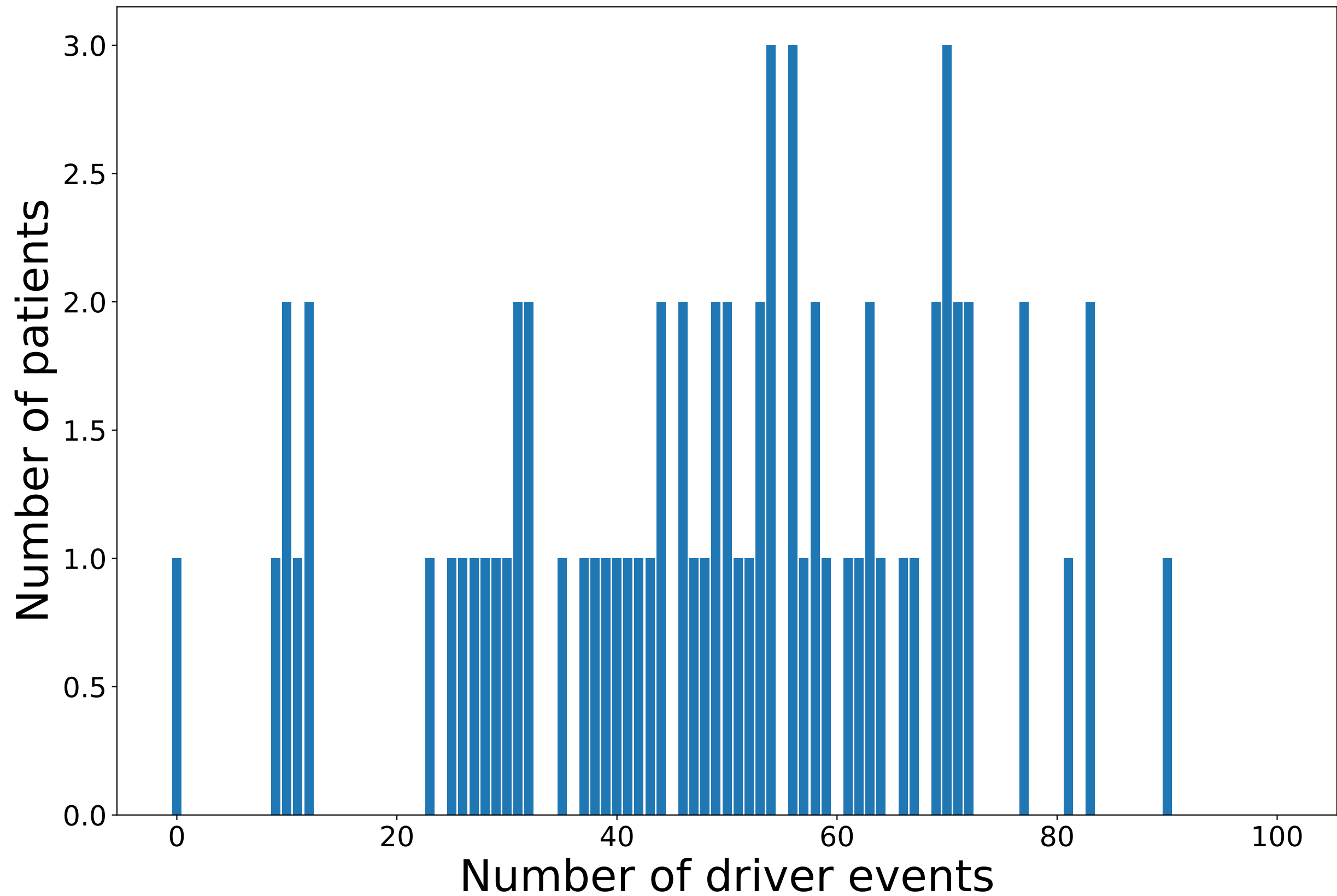

Supplement: S2 Files — (ZIP) [file pgen.1009996.s002.zip › PANCAN/patient distributions/2021_11_23_14_43_BLCA_FEMALE.pdf]

# PRAD\_MALE

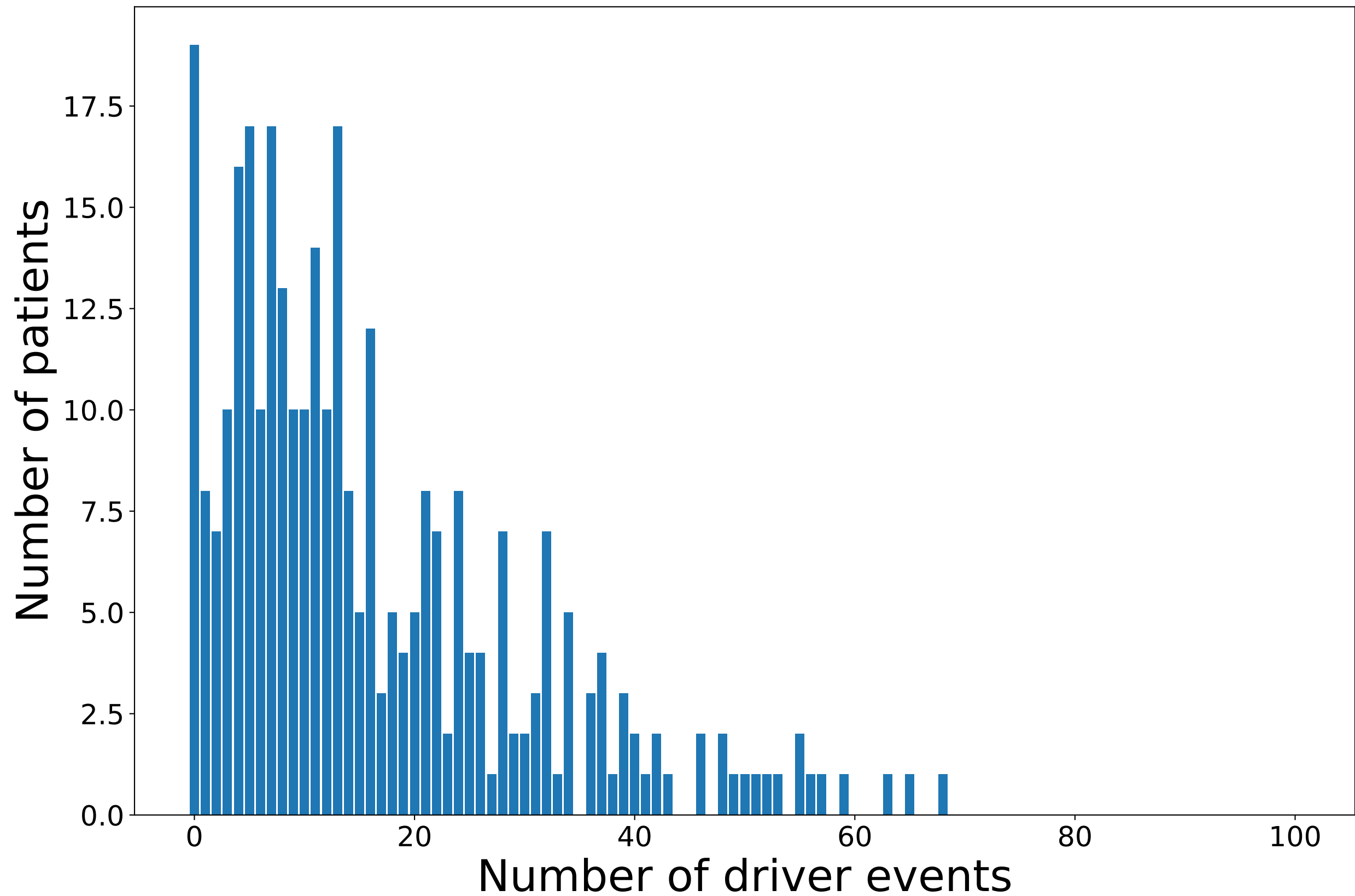

Supplement: S2 Files — (ZIP) [file pgen.1009996.s002.zip › PANCAN/patient distributions/2021_11_23_14_43_PRAD_MALE.pdf]

# KIRP

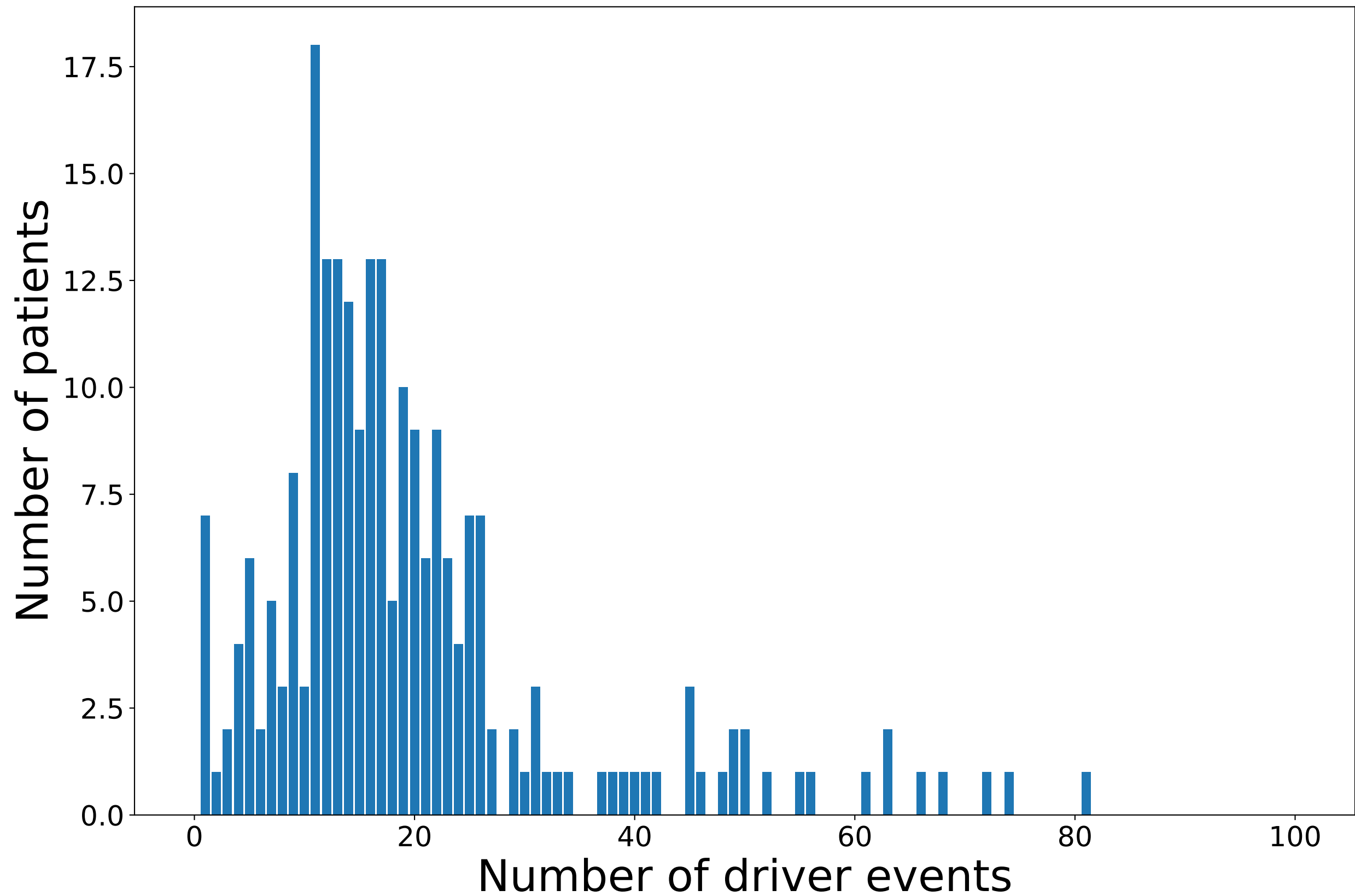

Supplement: S2 Files — (ZIP) [file pgen.1009996.s002.zip › PANCAN/patient distributions/2021_11_23_14_43_KIRP.pdf]

# COAD

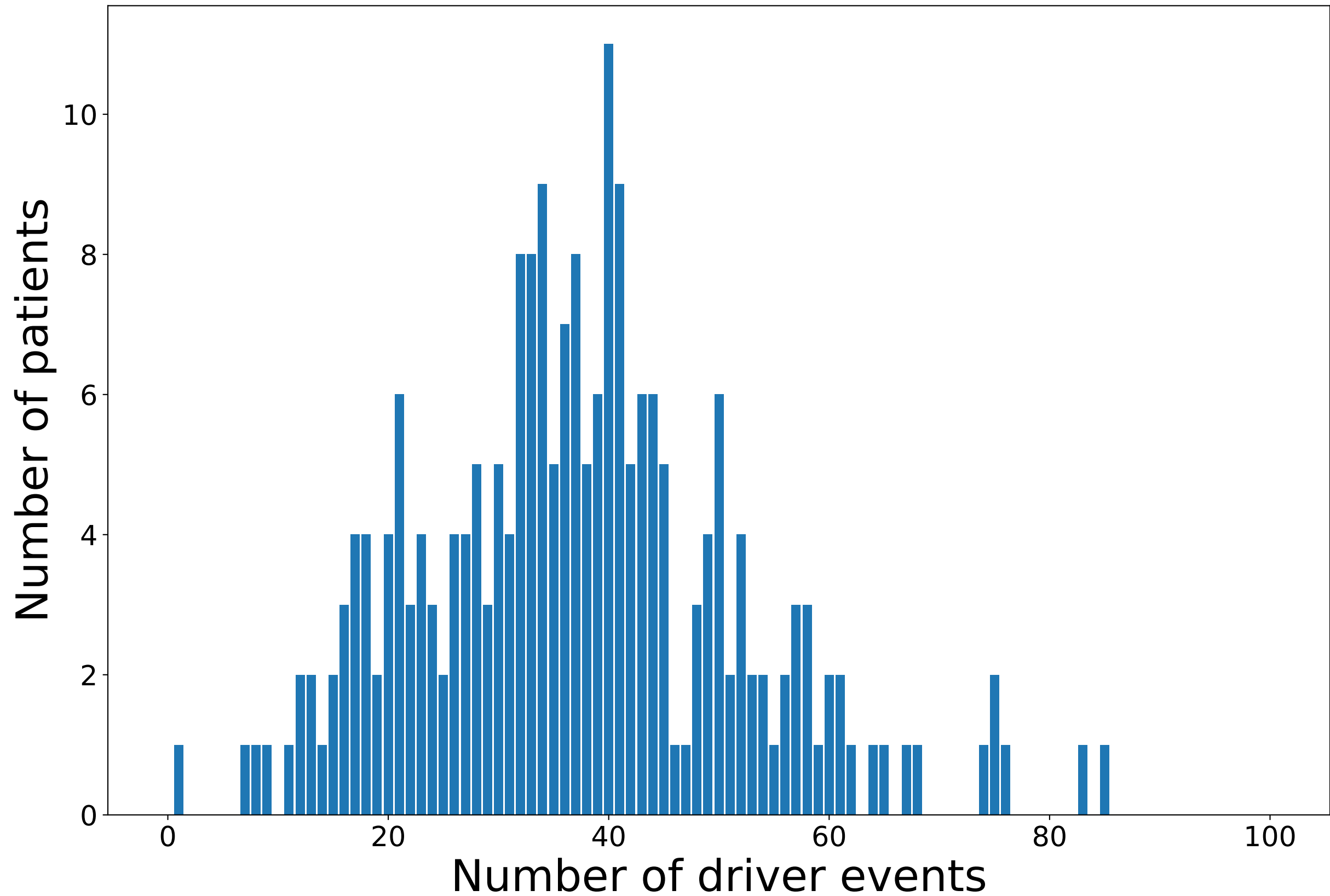

Supplement: S2 Files — (ZIP) [file pgen.1009996.s002.zip › PANCAN/patient distributions/2021_11_23_14_43_COAD.pdf]

# LIHC\_FEMALE

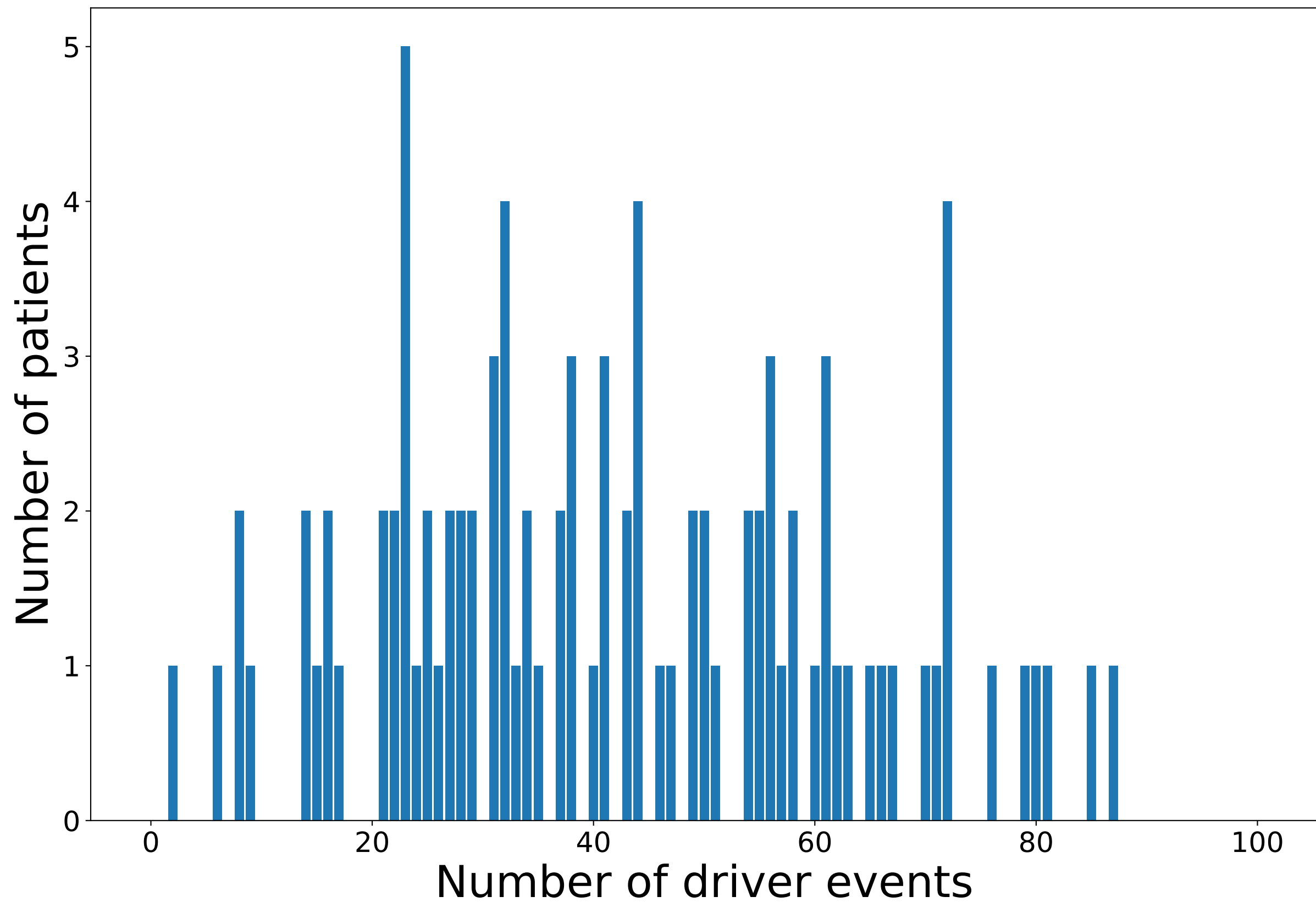

Supplement: S2 Files — (ZIP) [file pgen.1009996.s002.zip › PANCAN/patient distributions/2021_11_23_14_43_LIHC_FEMALE.pdf]

# LUAD\_MALE

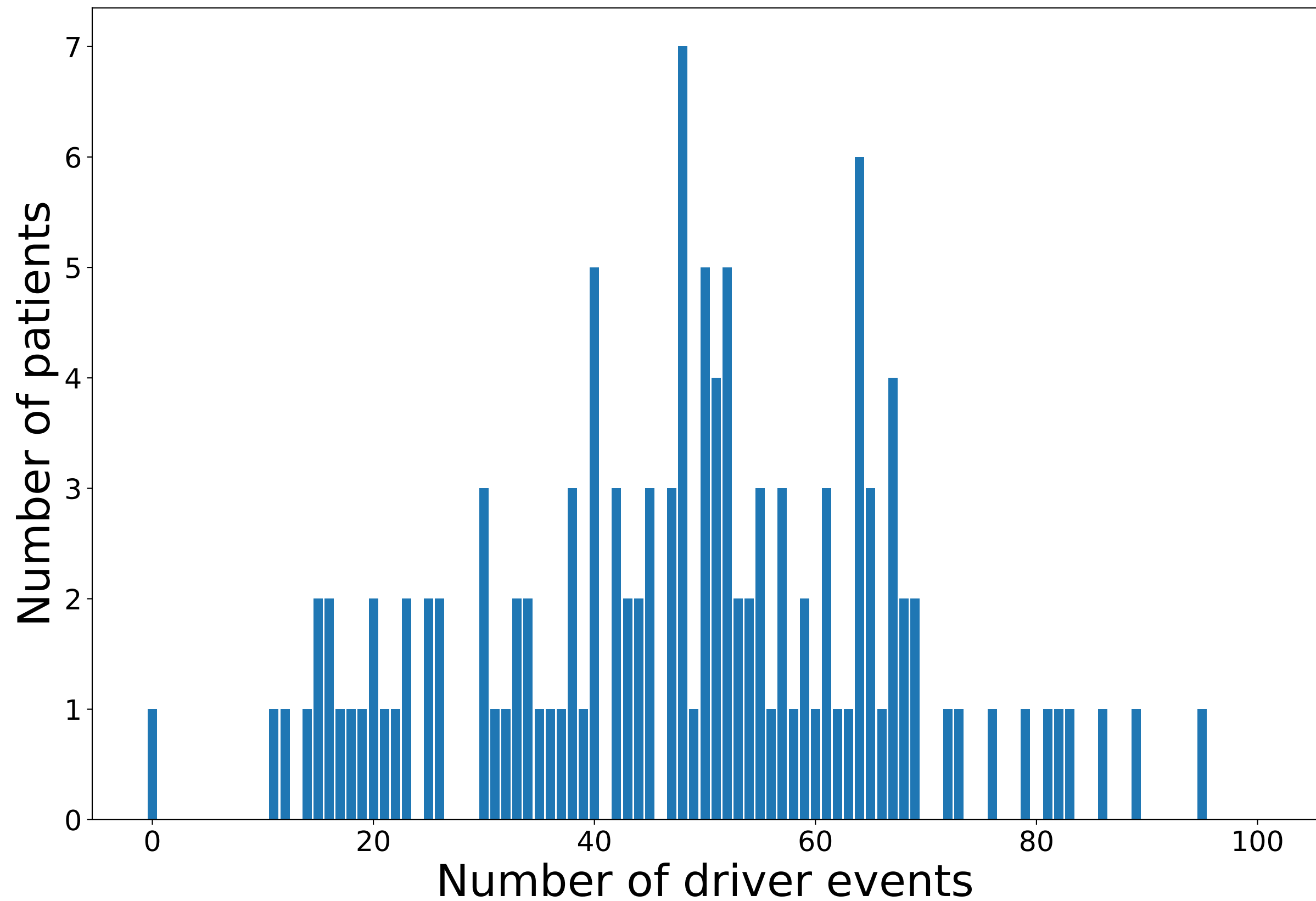

Supplement: S2 Files — (ZIP) [file pgen.1009996.s002.zip › PANCAN/patient distributions/2021_11_23_14_43_LUAD_MALE.pdf]

# LUSC\_MALE

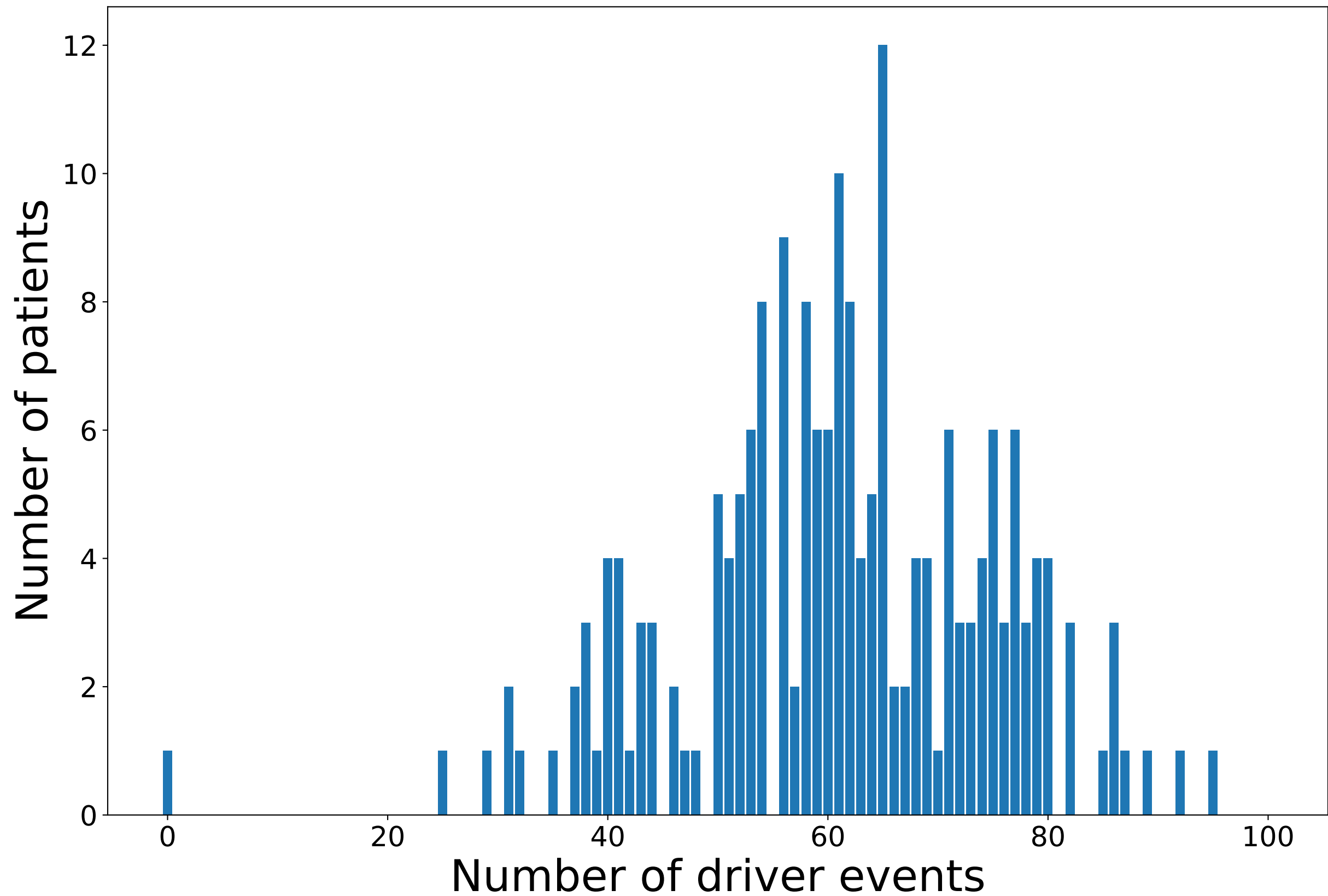

Supplement: S2 Files — (ZIP) [file pgen.1009996.s002.zip › PANCAN/patient distributions/2021_11_23_14_43_LUSC_MALE.pdf]

# KIRC\_FEMALE

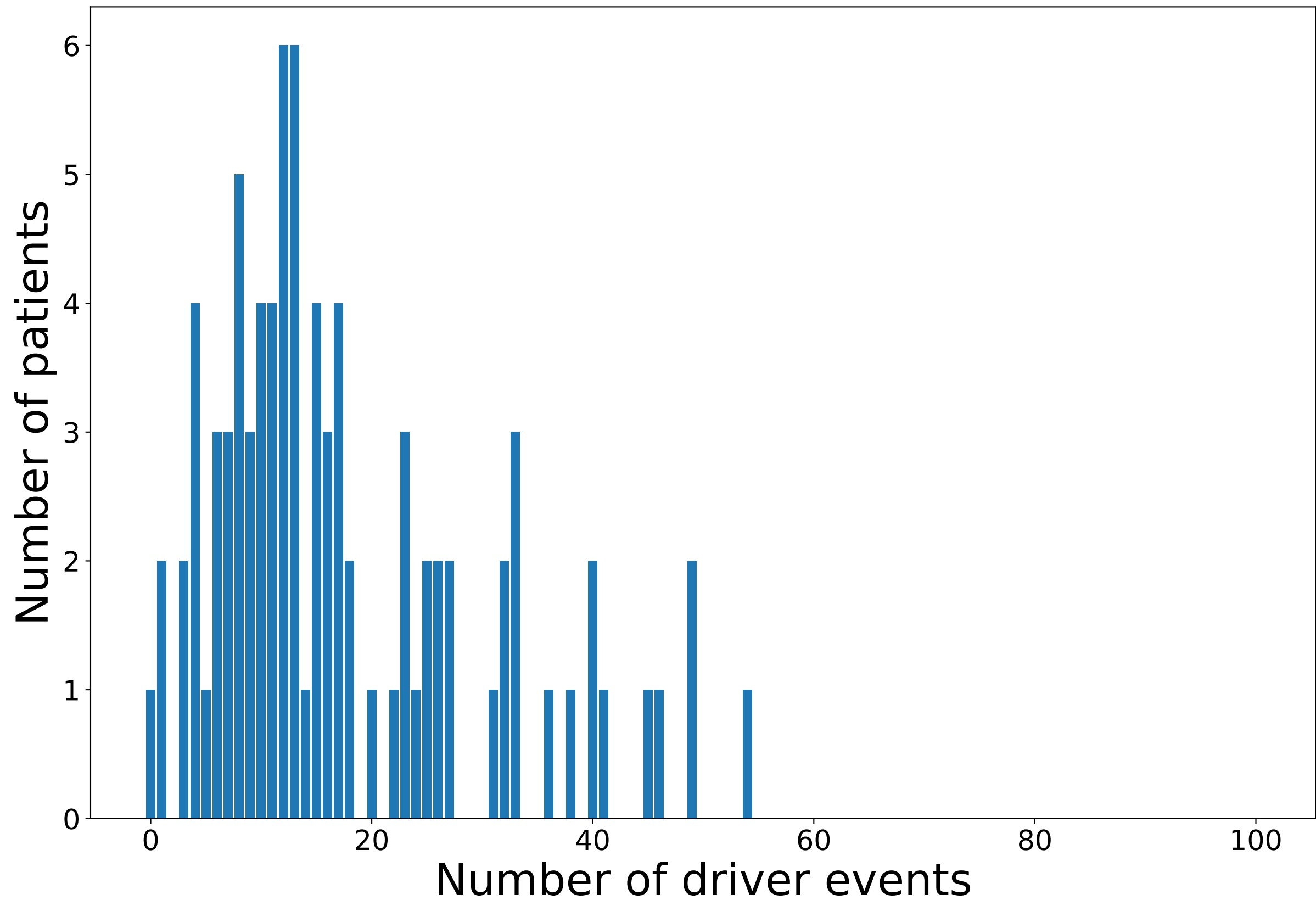

Supplement: S2 Files — (ZIP) [file pgen.1009996.s002.zip › PANCAN/patient distributions/2021_11_23_14_43_KIRC_FEMALE.pdf]

# LGG\_MALE

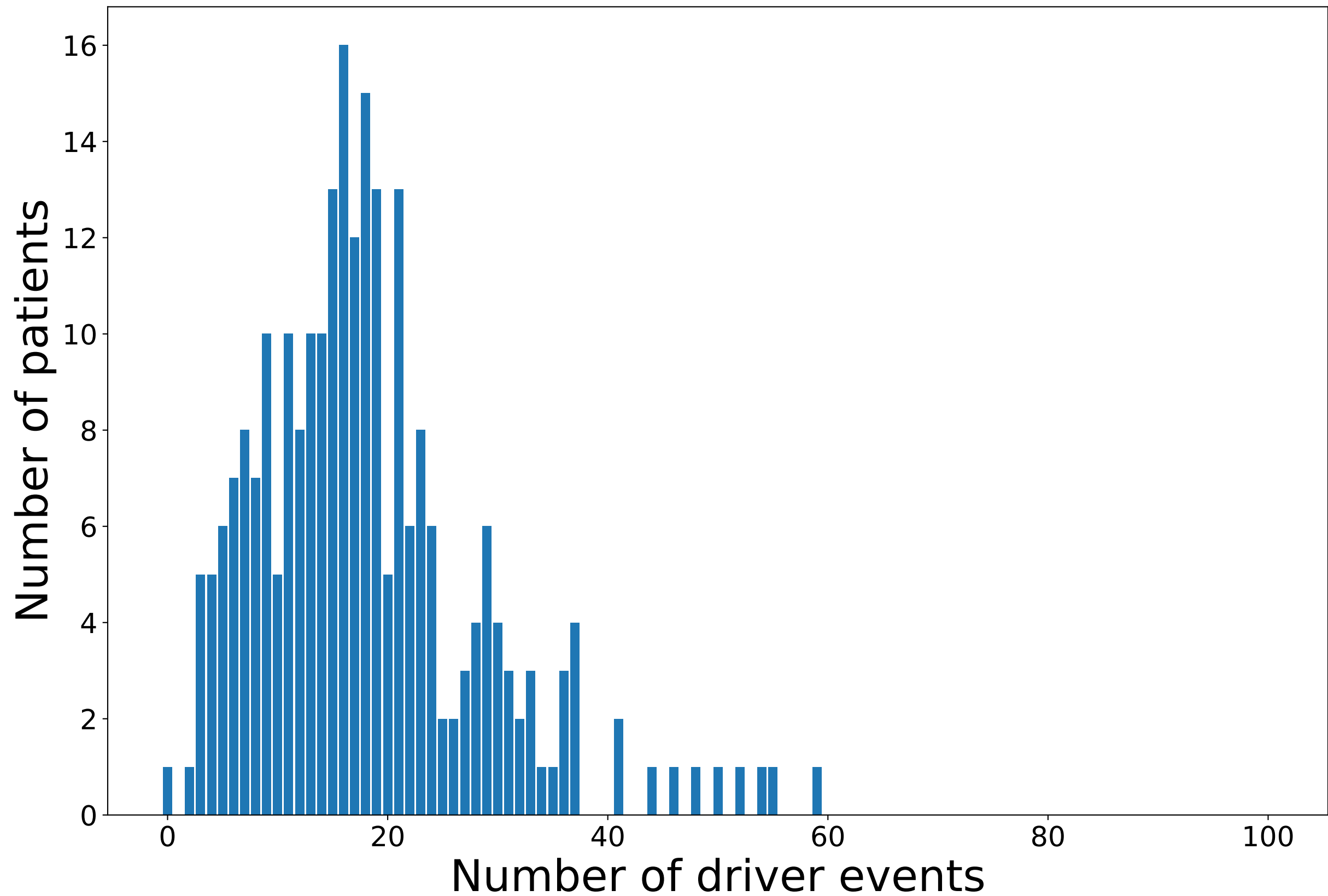

Supplement: S2 Files — (ZIP) [file pgen.1009996.s002.zip › PANCAN/patient distributions/2021_11_23_14_43_LGG_MALE.pdf]

# CHOL\_FEMALE

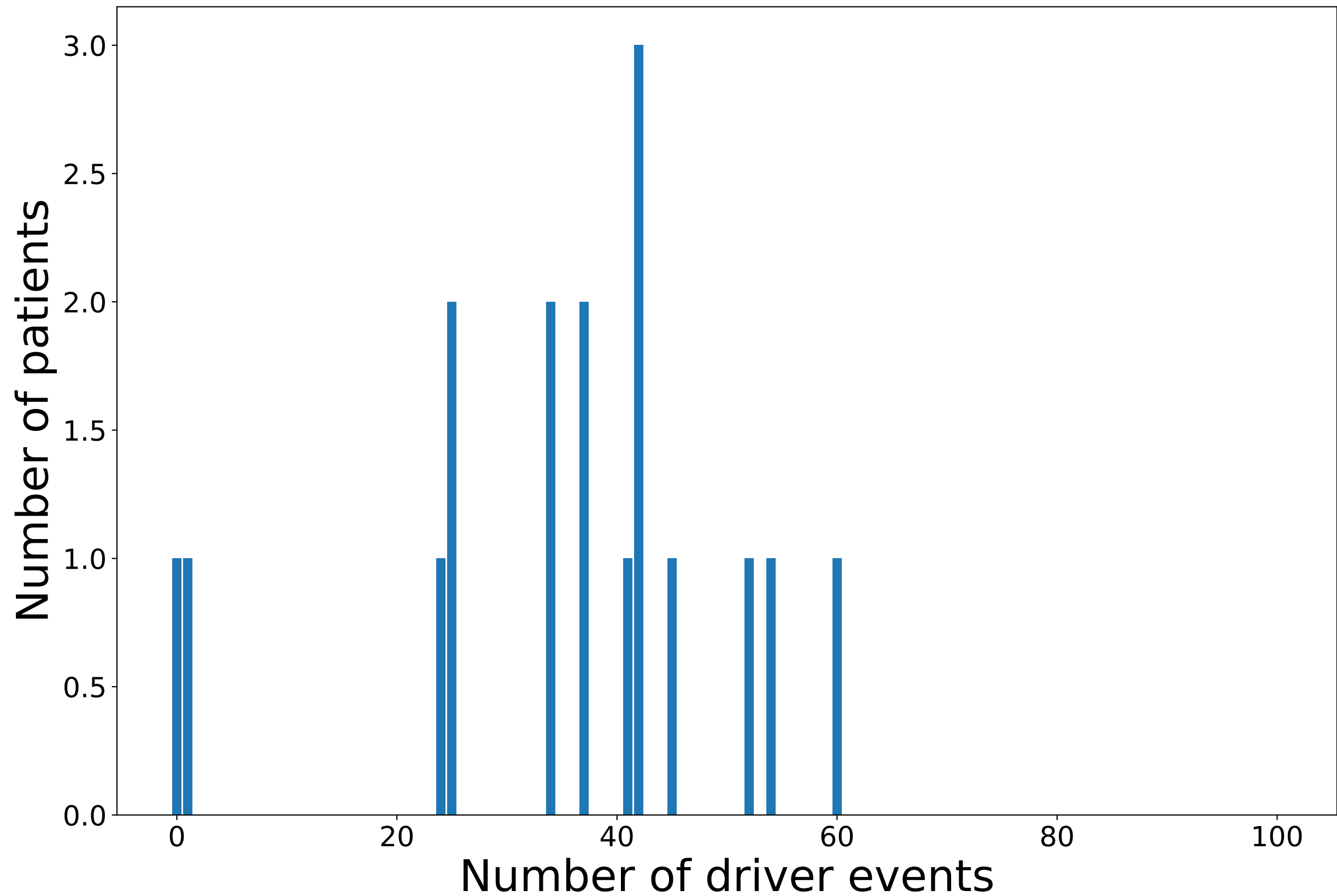

Supplement: S2 Files — (ZIP) [file pgen.1009996.s002.zip › PANCAN/patient distributions/2021_11_23_14_43_CHOL_FEMALE.pdf]

# LIHC

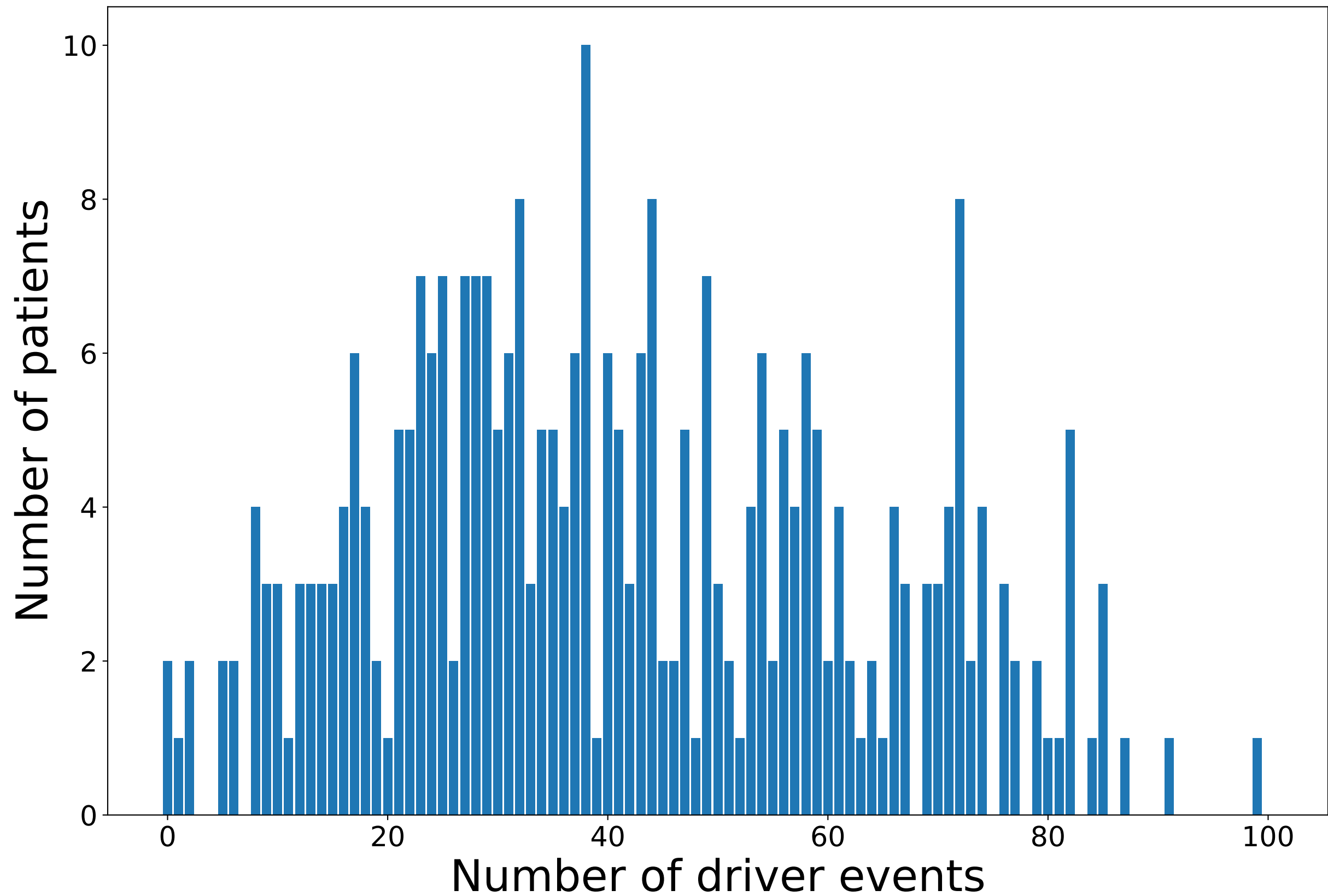

Supplement: S2 Files — (ZIP) [file pgen.1009996.s002.zip › PANCAN/patient distributions/2021_11_23_14_43_LIHC.pdf]

# KIRP\_FEMALE

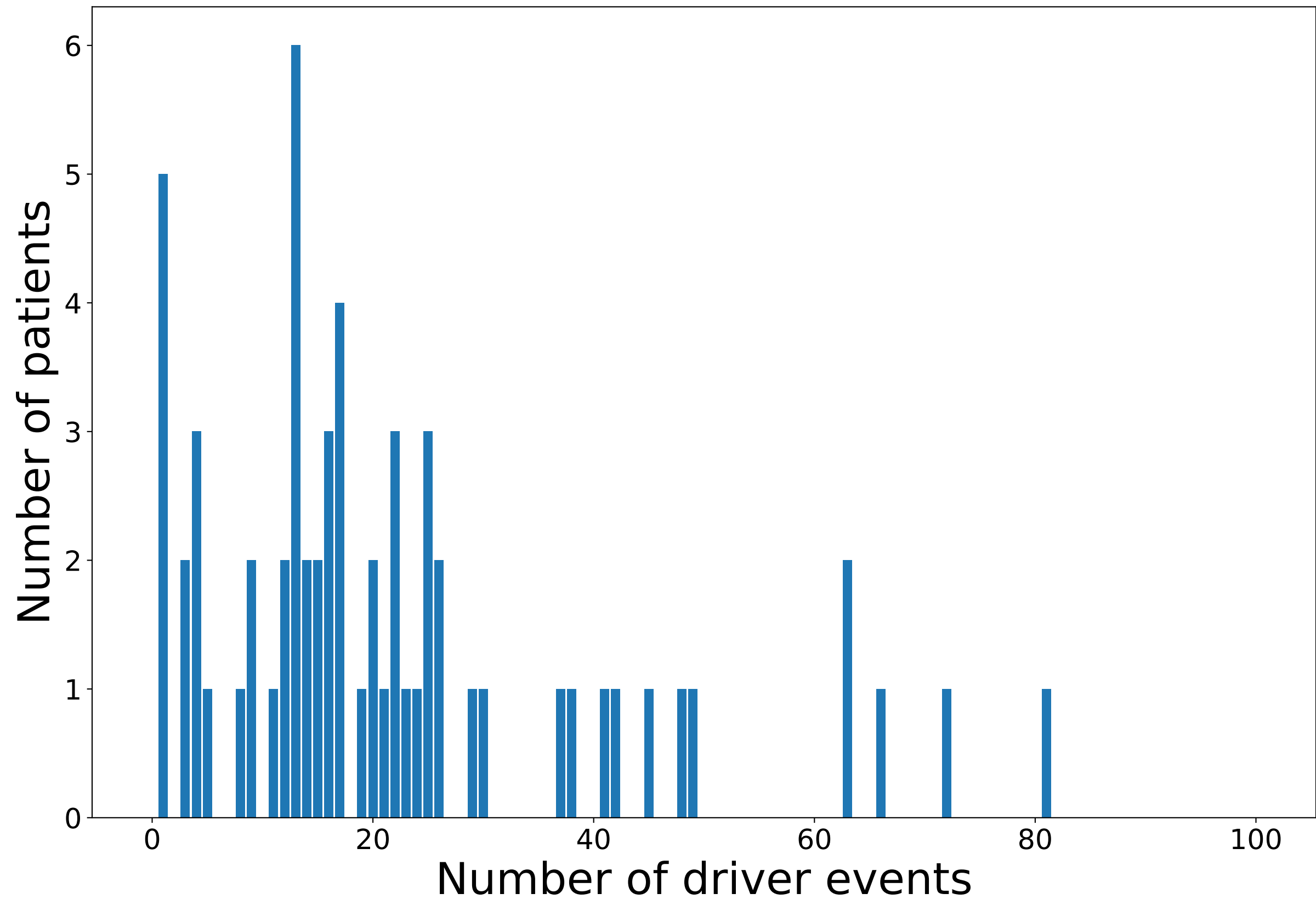

Supplement: S2 Files — (ZIP) [file pgen.1009996.s002.zip › PANCAN/patient distributions/2021_11_23_14_43_KIRP_FEMALE.pdf]

# PAAD\_MALE

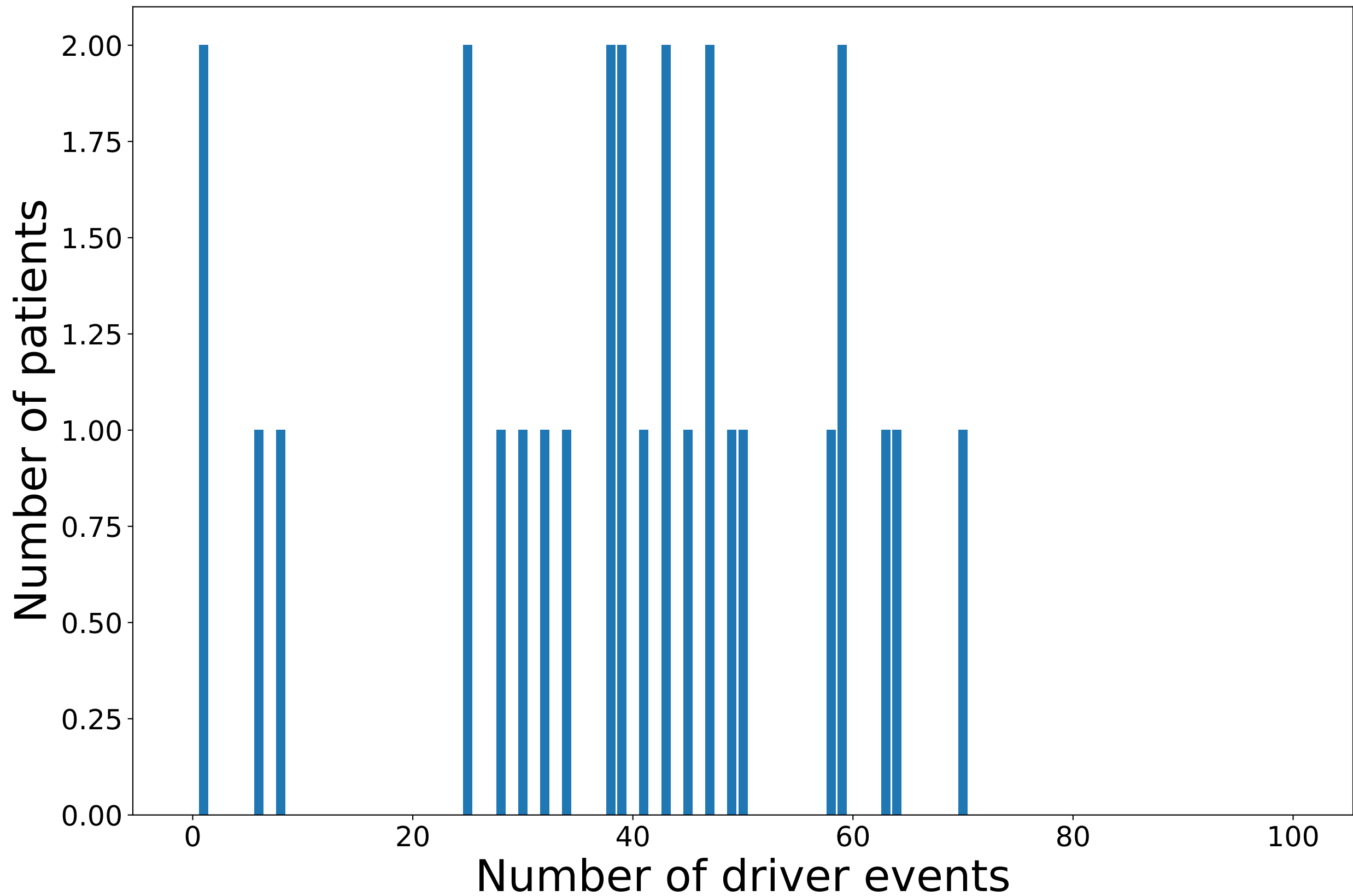

Supplement: S2 Files — (ZIP) [file pgen.1009996.s002.zip › PANCAN/patient distributions/2021_11_23_14_43_PAAD_MALE.pdf]

# SKCM\_MALE

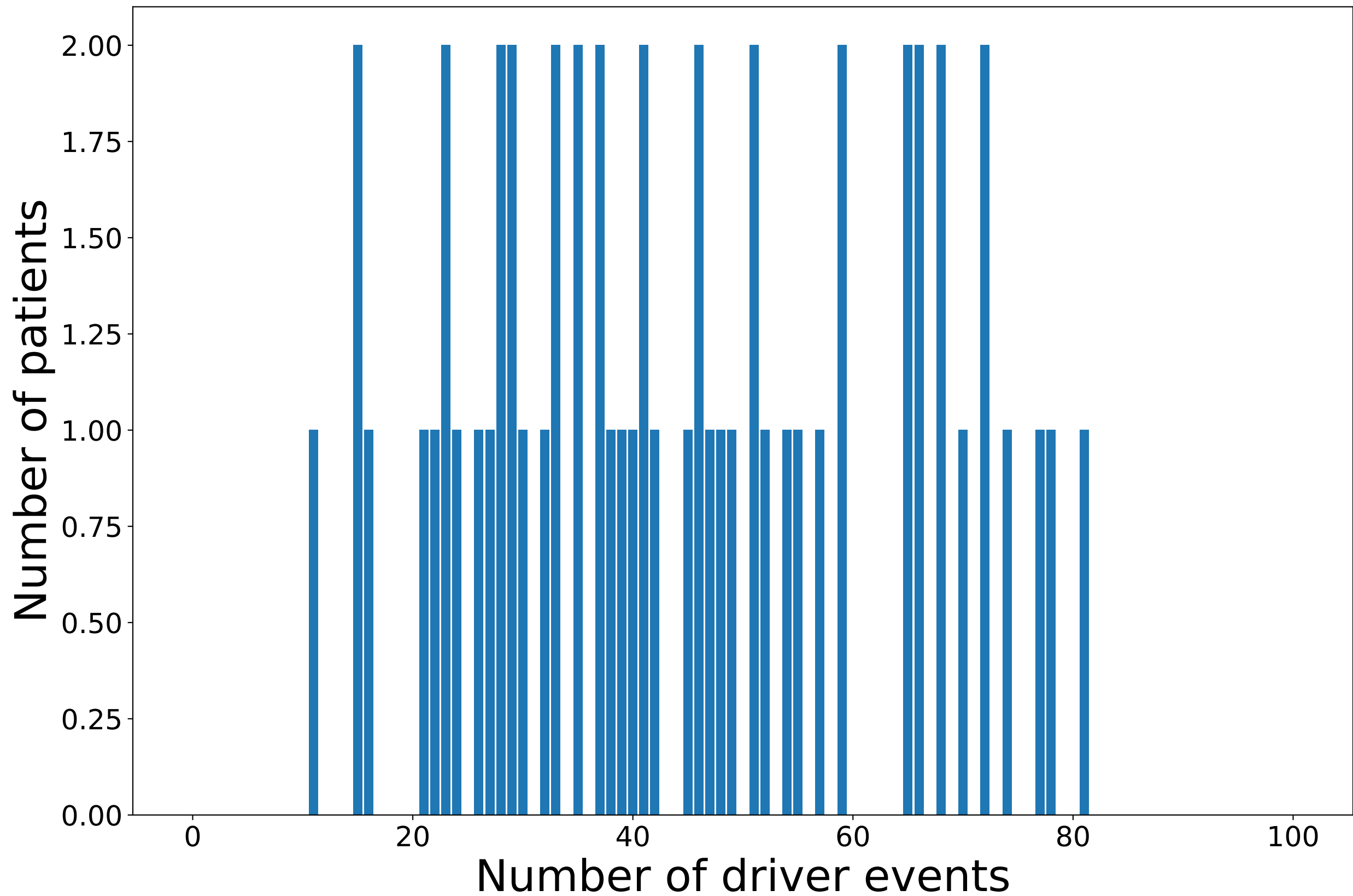

Supplement: S2 Files — (ZIP) [file pgen.1009996.s002.zip › PANCAN/patient distributions/2021_11_23_14_43_SKCM_MALE.pdf]

# READ

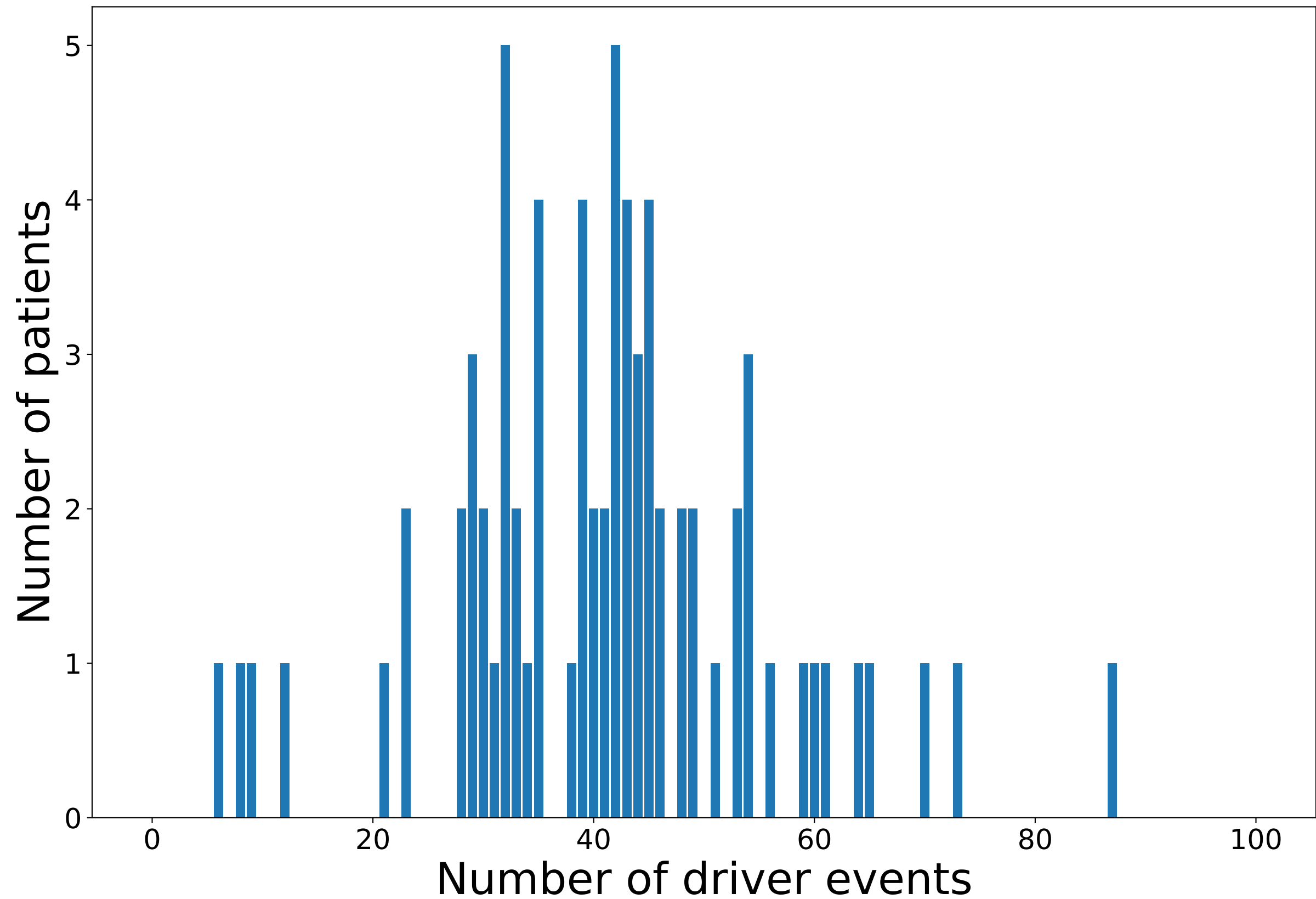

Supplement: S2 Files — (ZIP) [file pgen.1009996.s002.zip › PANCAN/patient distributions/2021_11_23_14_43_READ.pdf]

# DLBC\_FEMALE

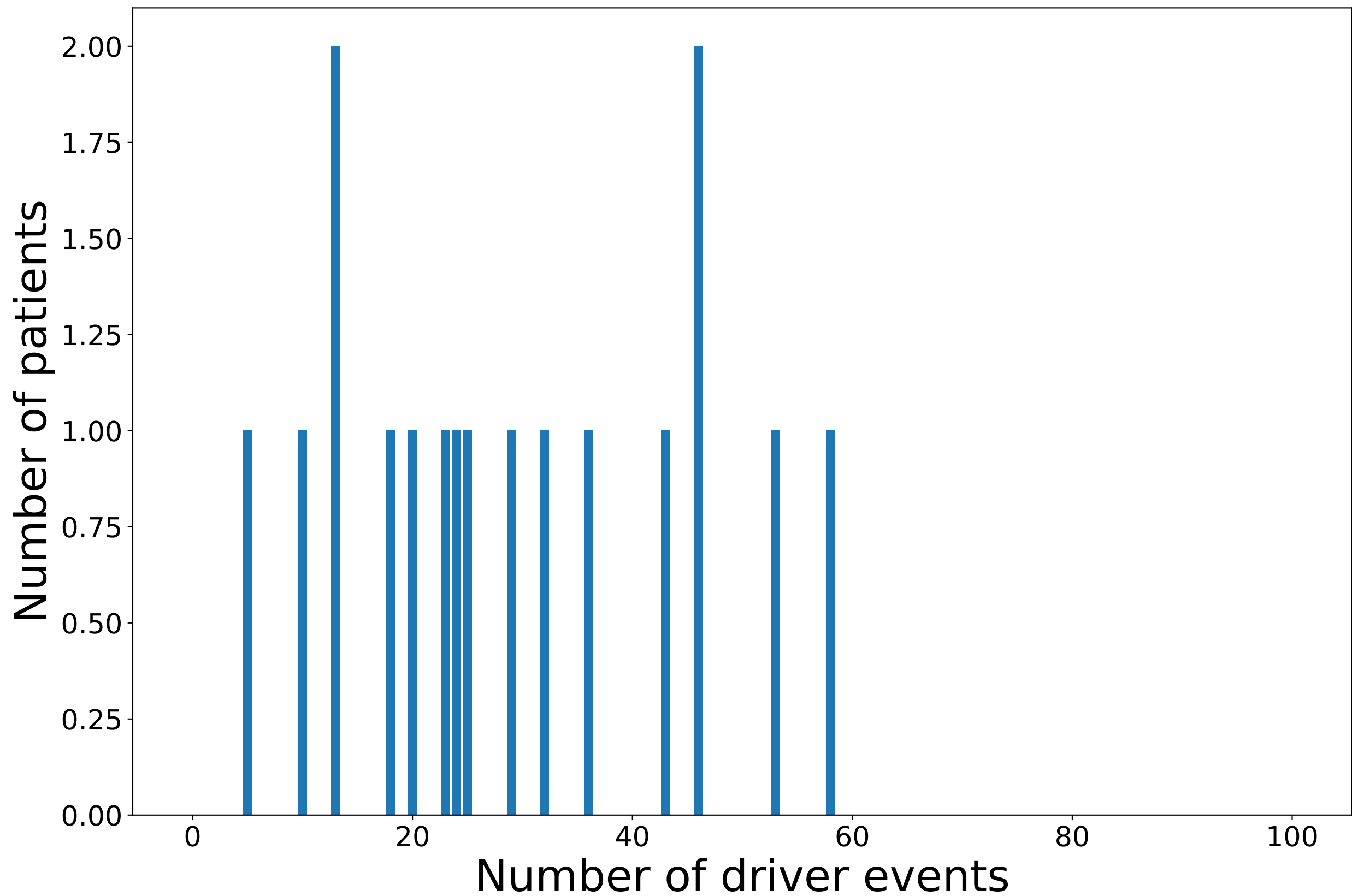

Supplement: S2 Files — (ZIP) [file pgen.1009996.s002.zip › PANCAN/patient distributions/2021_11_23_14_43_DLBC_FEMALE.pdf]

# SARC\_MALE

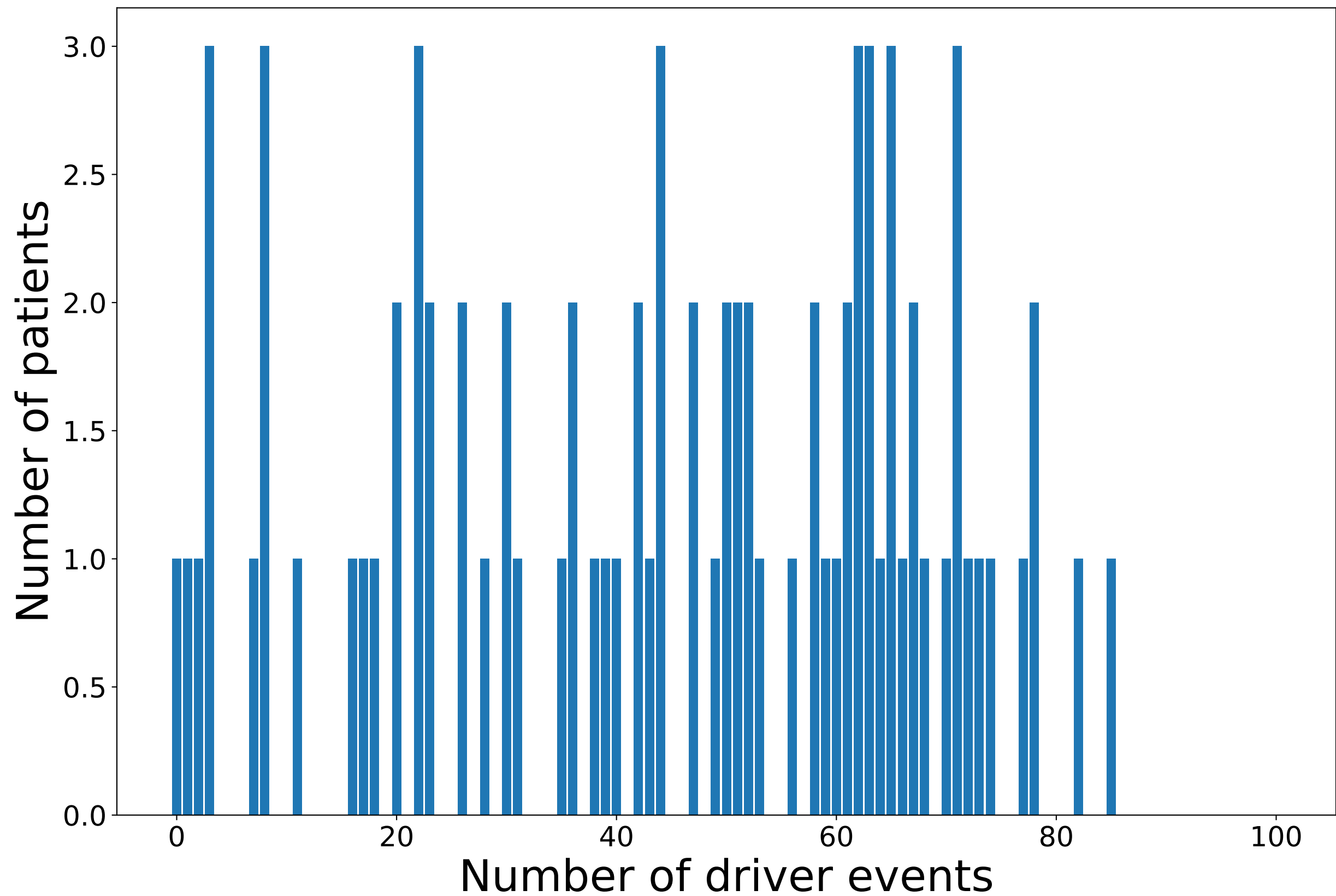

Supplement: S2 Files — (ZIP) [file pgen.1009996.s002.zip › PANCAN/patient distributions/2021_11_23_14_43_SARC_MALE.pdf]

# UVM\_FEMALE

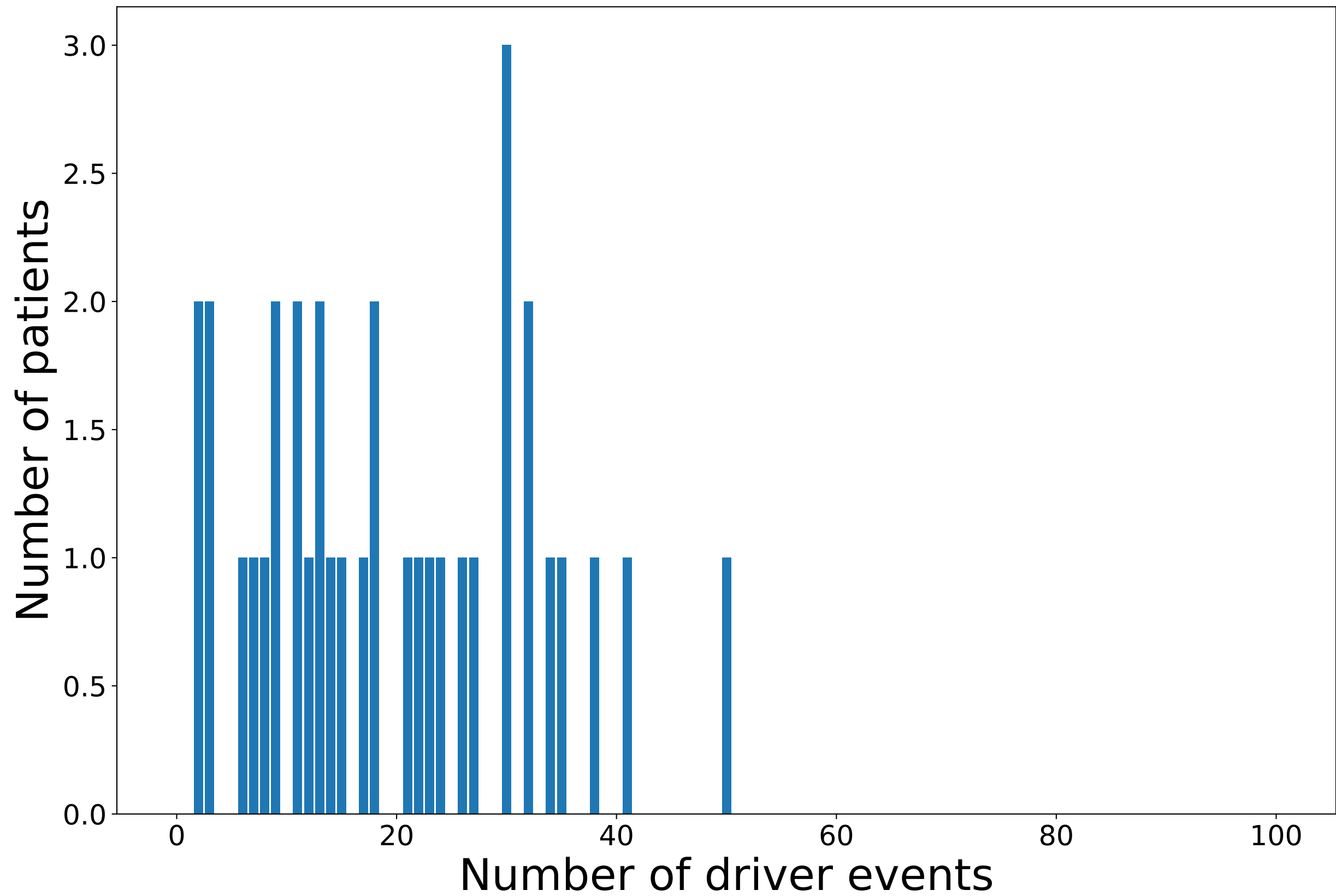

Supplement: S2 Files — (ZIP) [file pgen.1009996.s002.zip › PANCAN/patient distributions/2021_11_23_14_43_UVM_FEMALE.pdf]

# BLCA

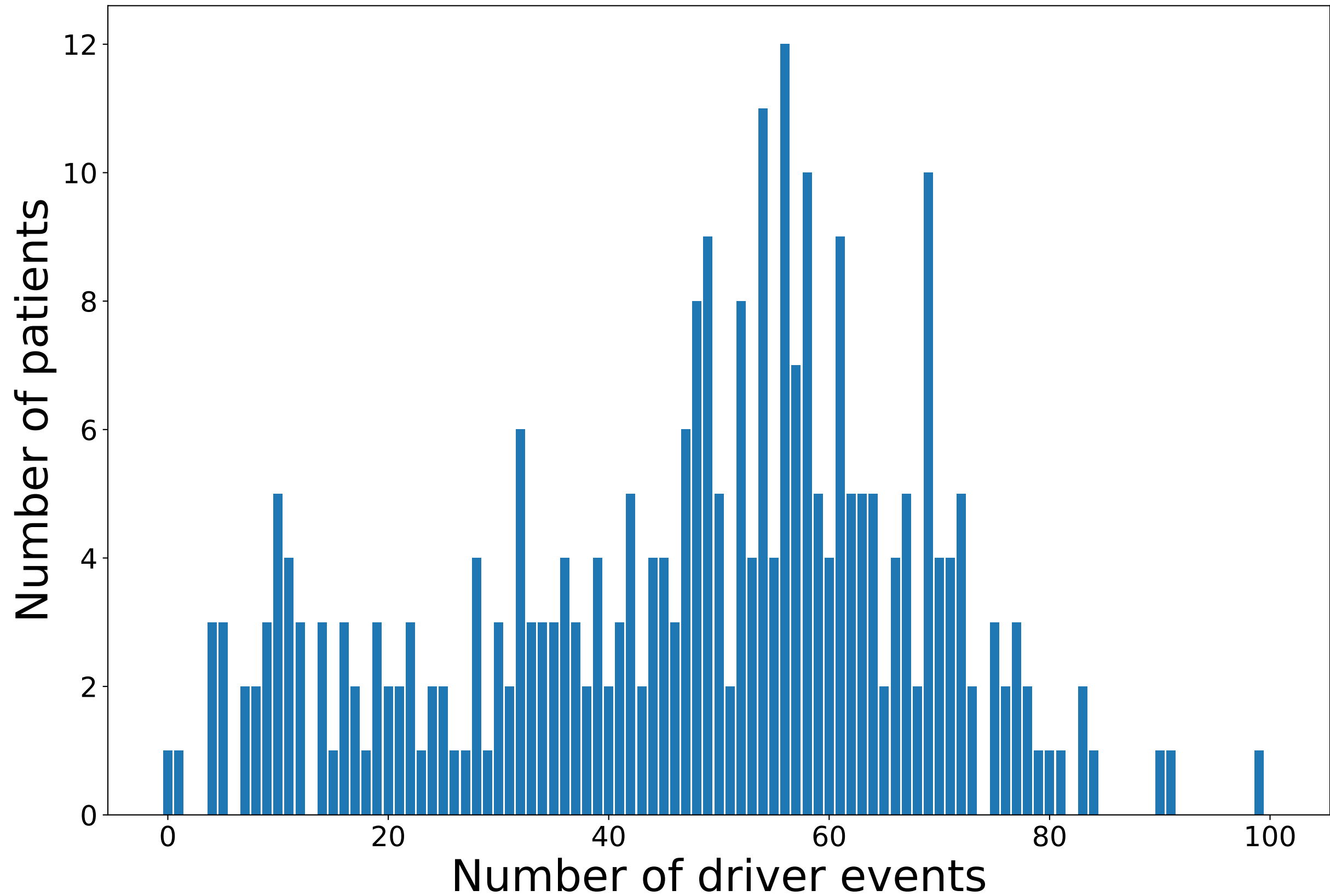

Supplement: S2 Files — (ZIP) [file pgen.1009996.s002.zip › PANCAN/patient distributions/2021_11_23_14_43_BLCA.pdf]

# KICH\_FEMALE

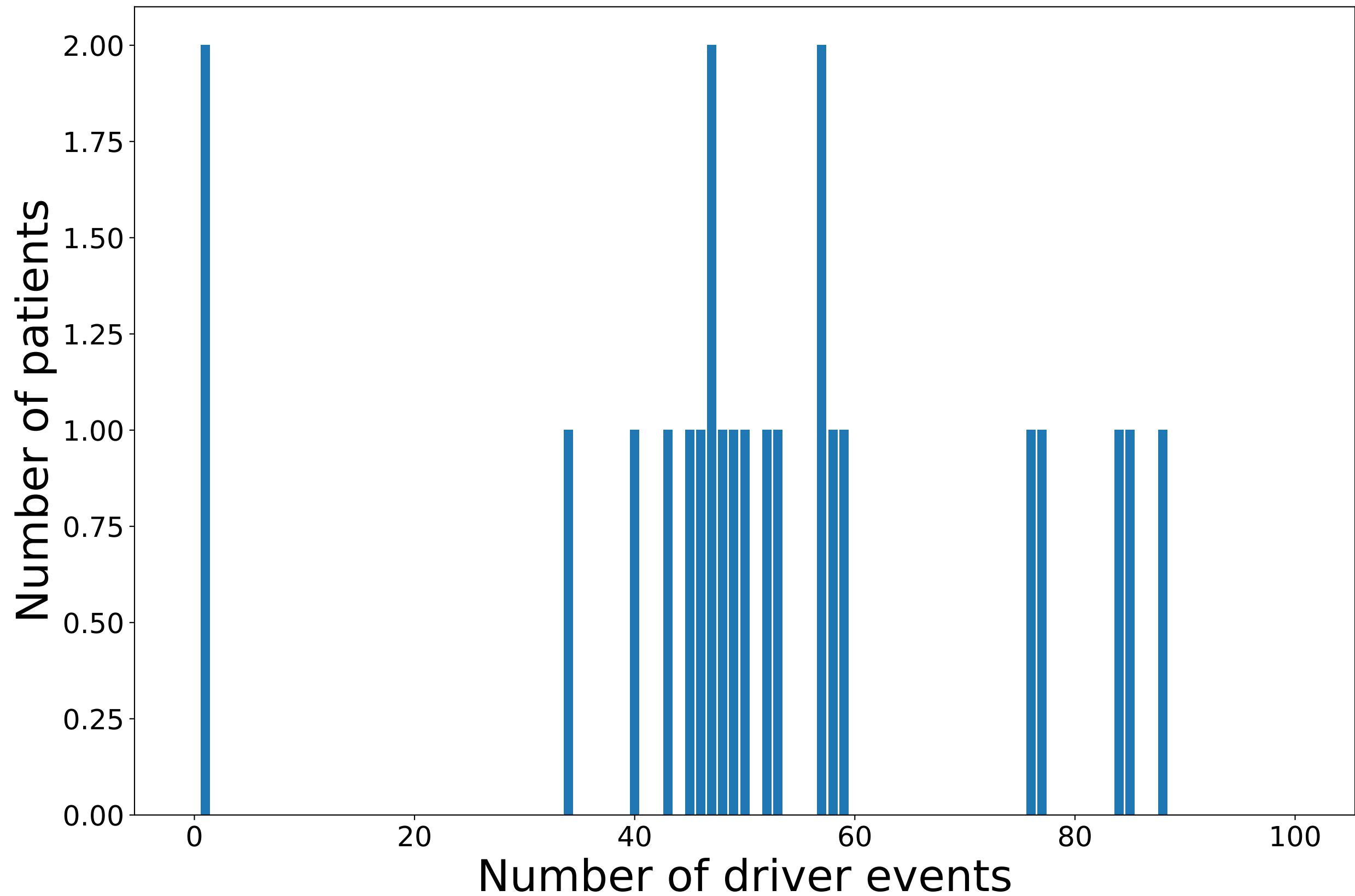

Supplement: S2 Files — (ZIP) [file pgen.1009996.s002.zip › PANCAN/patient distributions/2021_11_23_14_43_KICH_FEMALE.pdf]

# GBM\_MALE

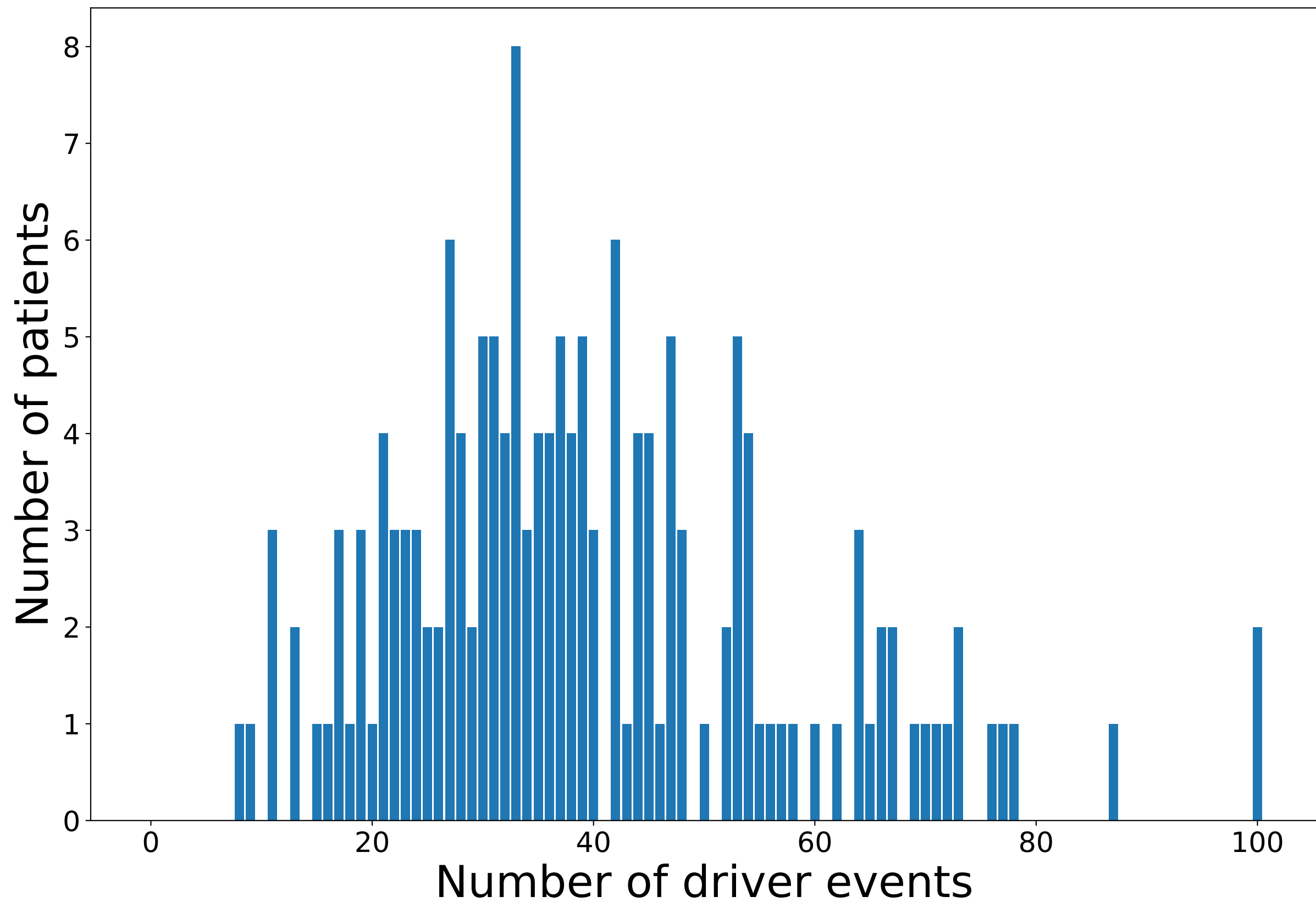

Supplement: S2 Files — (ZIP) [file pgen.1009996.s002.zip › PANCAN/patient distributions/2021_11_23_14_43_GBM_MALE.pdf]

# PAAD

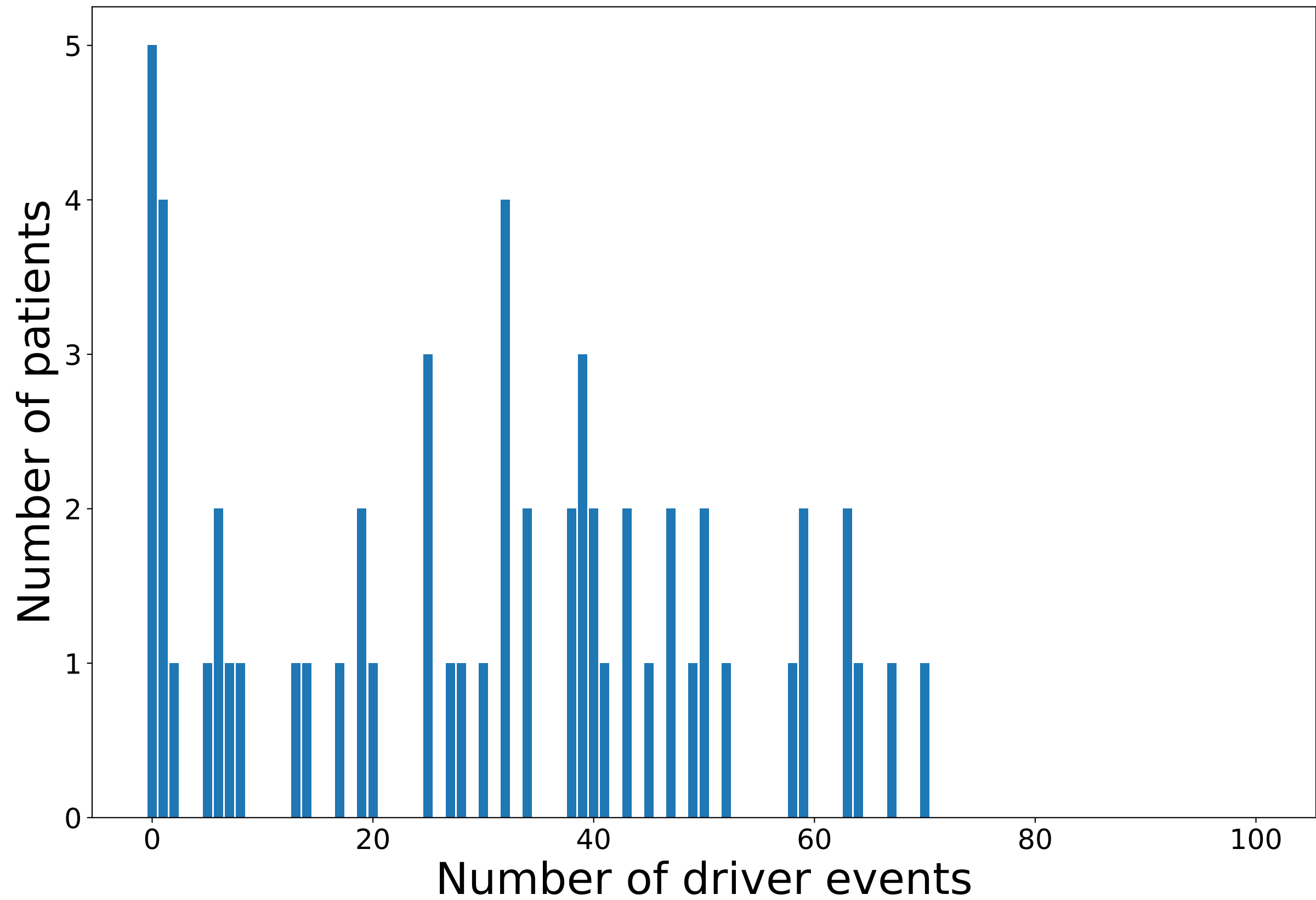

Supplement: S2 Files — (ZIP) [file pgen.1009996.s002.zip › PANCAN/patient distributions/2021_11_23_14_43_PAAD.pdf]

# THCA

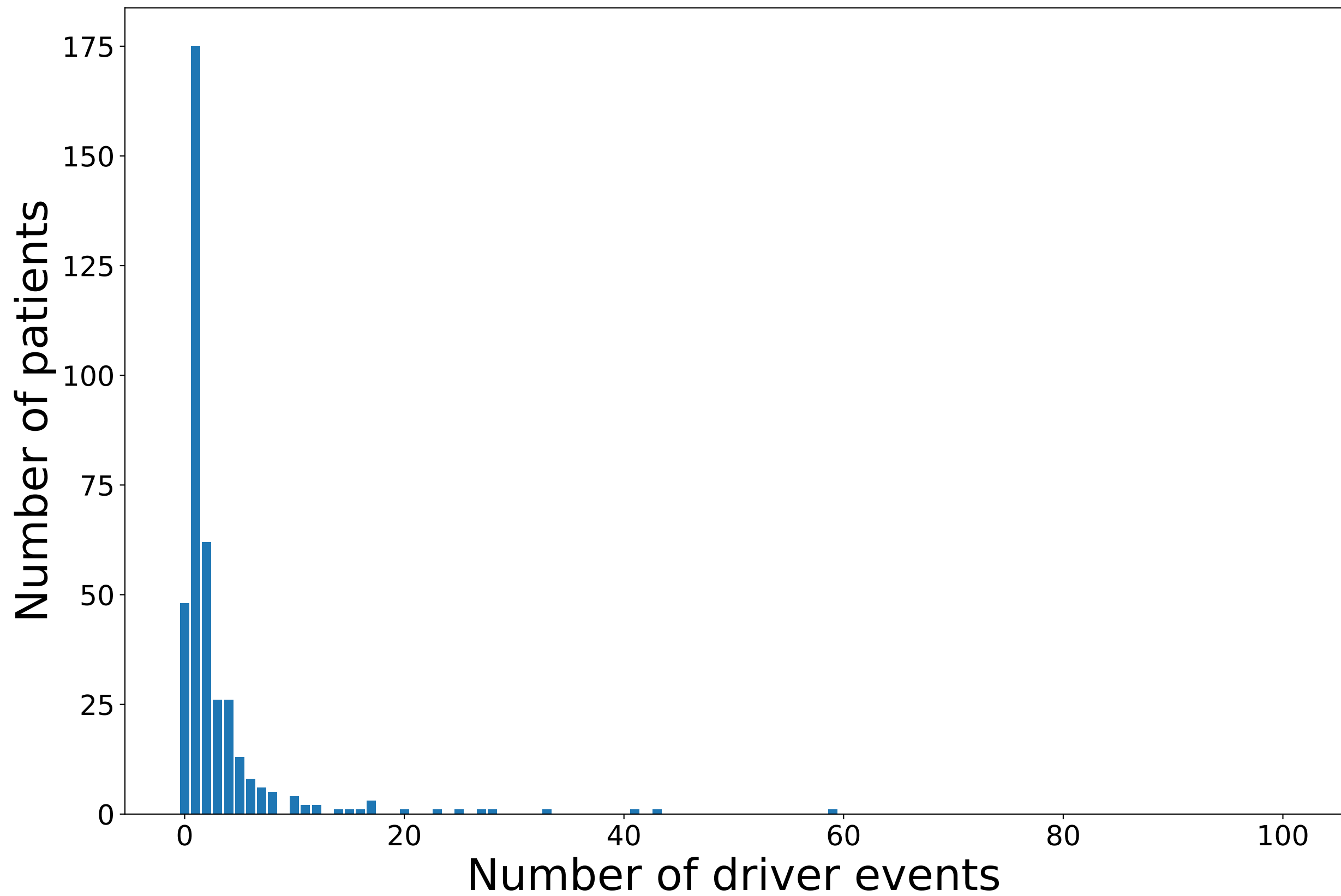

Supplement: S2 Files — (ZIP) [file pgen.1009996.s002.zip › PANCAN/patient distributions/2021_11_23_14_43_THCA.pdf]

# ESCA\_FEMALE

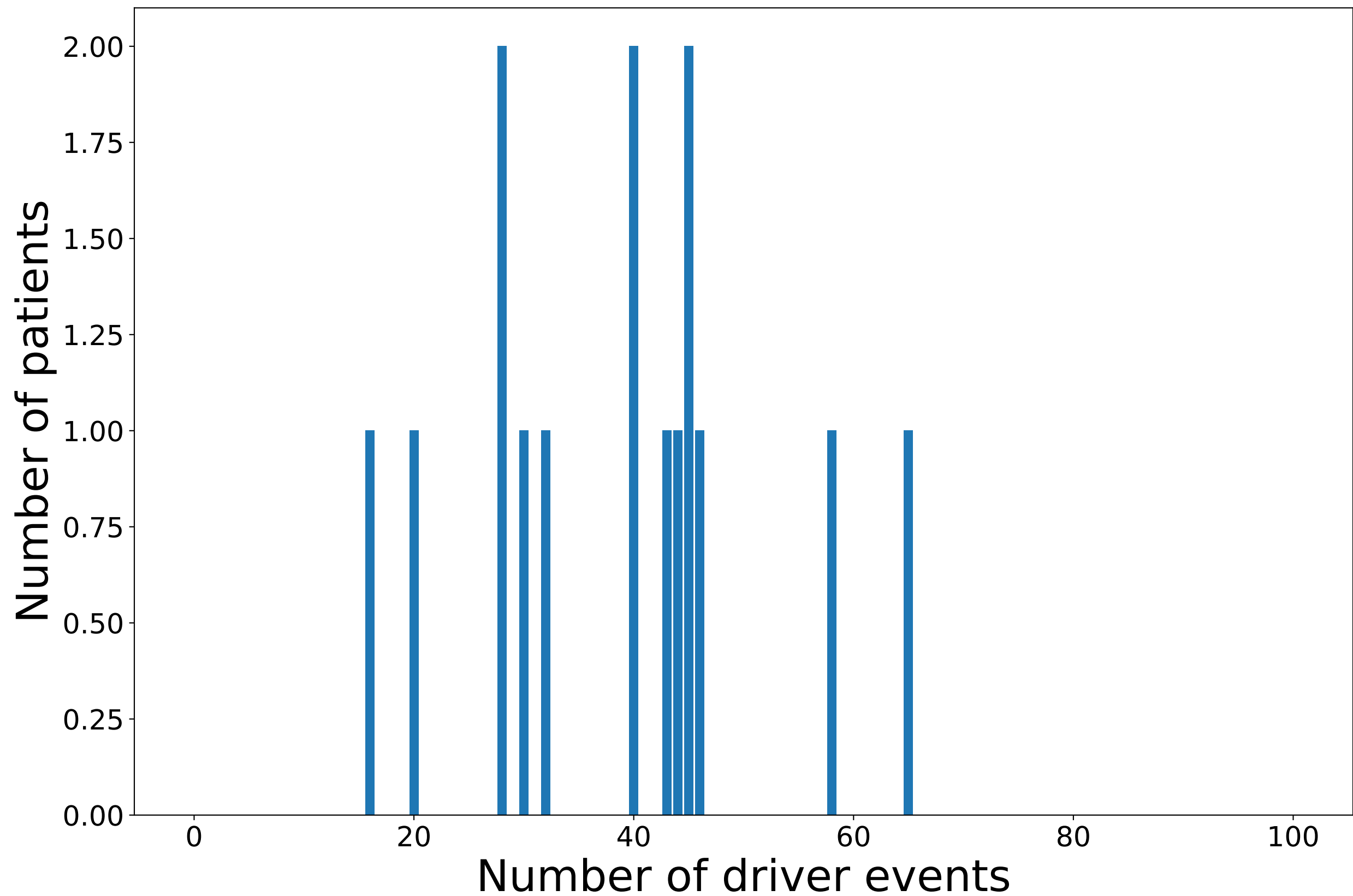

Supplement: S2 Files — (ZIP) [file pgen.1009996.s002.zip › PANCAN/patient distributions/2021_11_23_14_43_ESCA_FEMALE.pdf]

# THYM

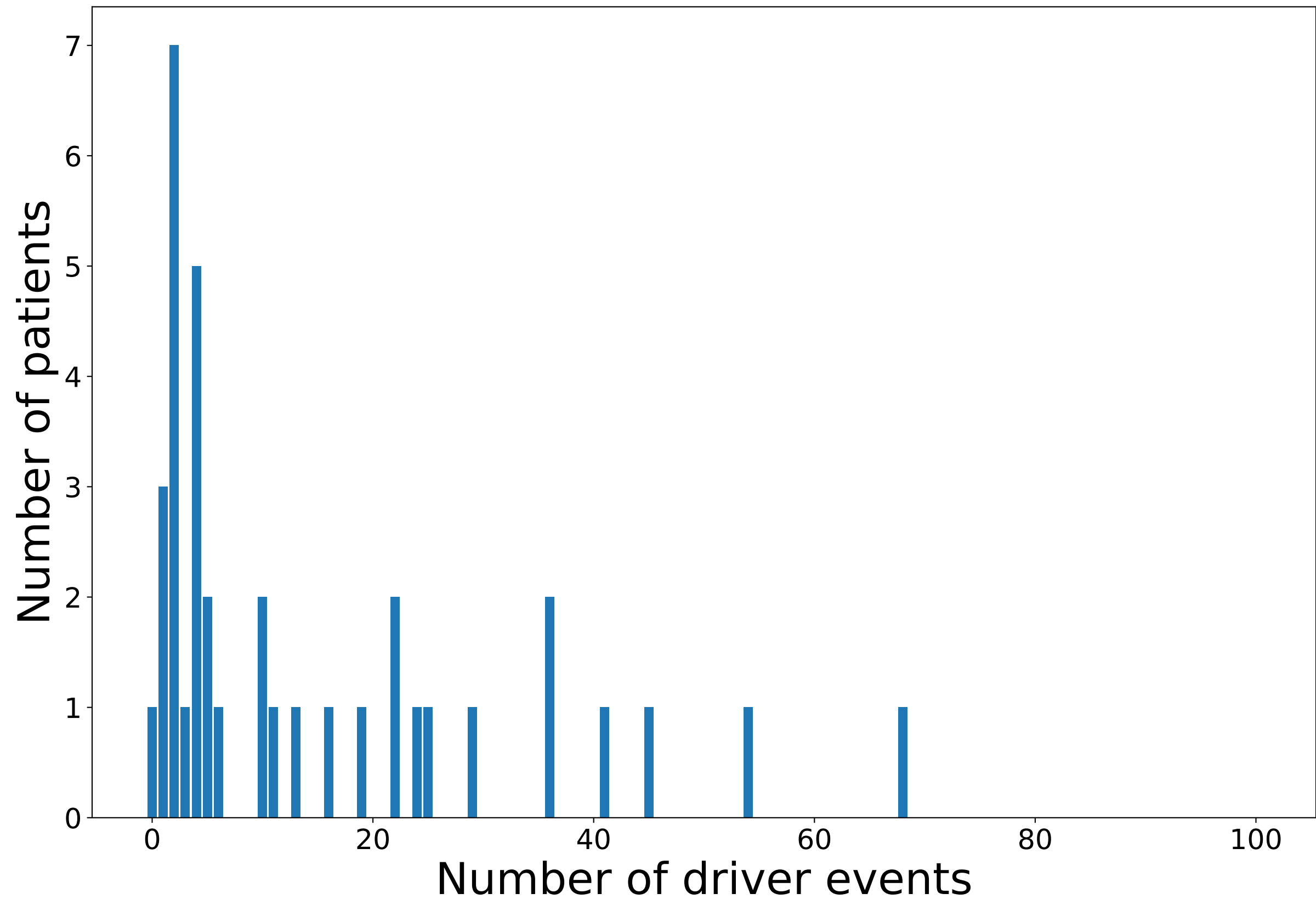

Supplement: S2 Files — (ZIP) [file pgen.1009996.s002.zip › PANCAN/patient distributions/2021_11_23_14_43_THYM.pdf]

# MESO\_MALE

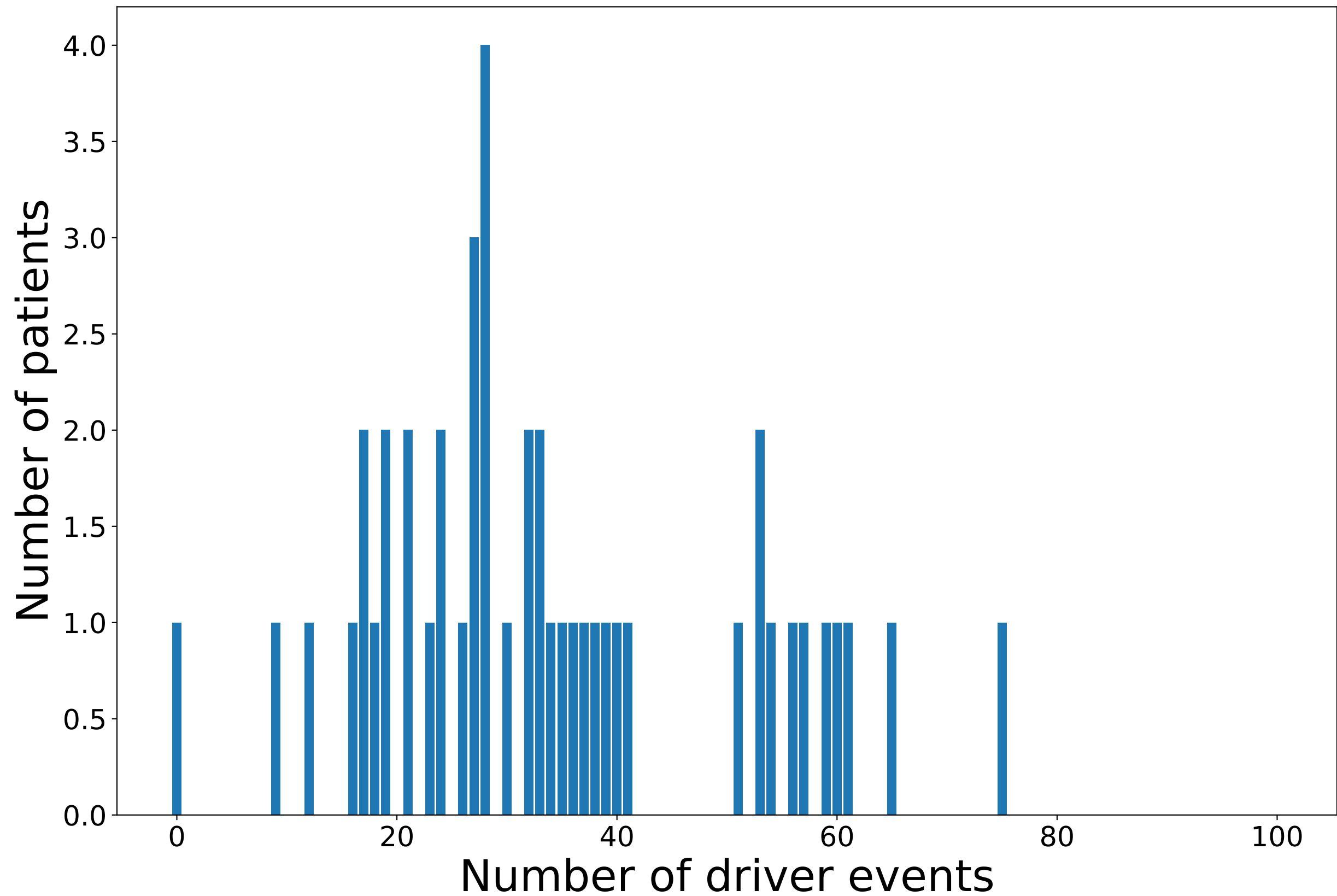

Supplement: S2 Files — (ZIP) [file pgen.1009996.s002.zip › PANCAN/patient distributions/2021_11_23_14_43_MESO_MALE.pdf]

# TGCT\_MALE

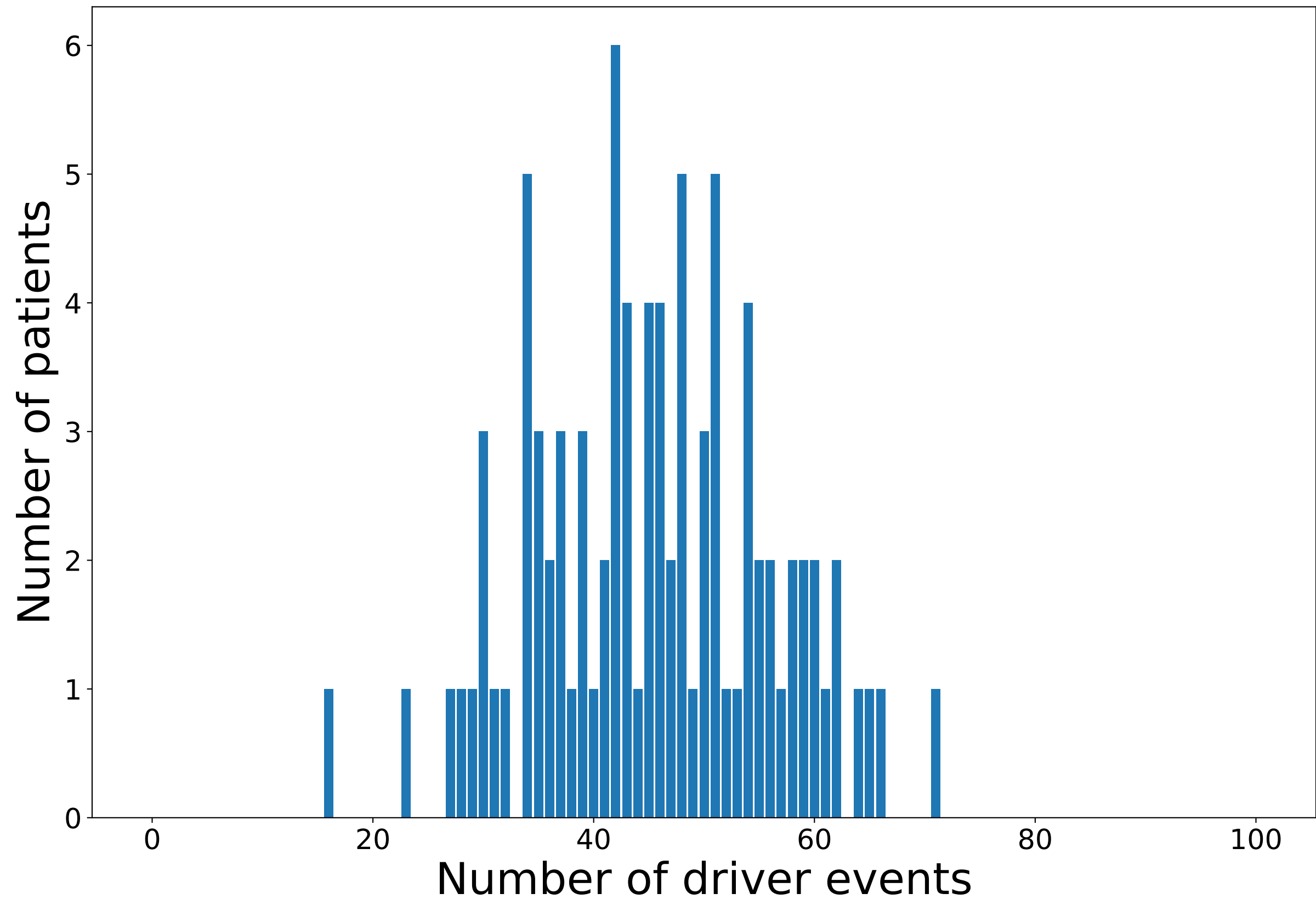

Supplement: S2 Files — (ZIP) [file pgen.1009996.s002.zip › PANCAN/patient distributions/2021_11_23_14_43_TGCT_MALE.pdf]

# KIRC

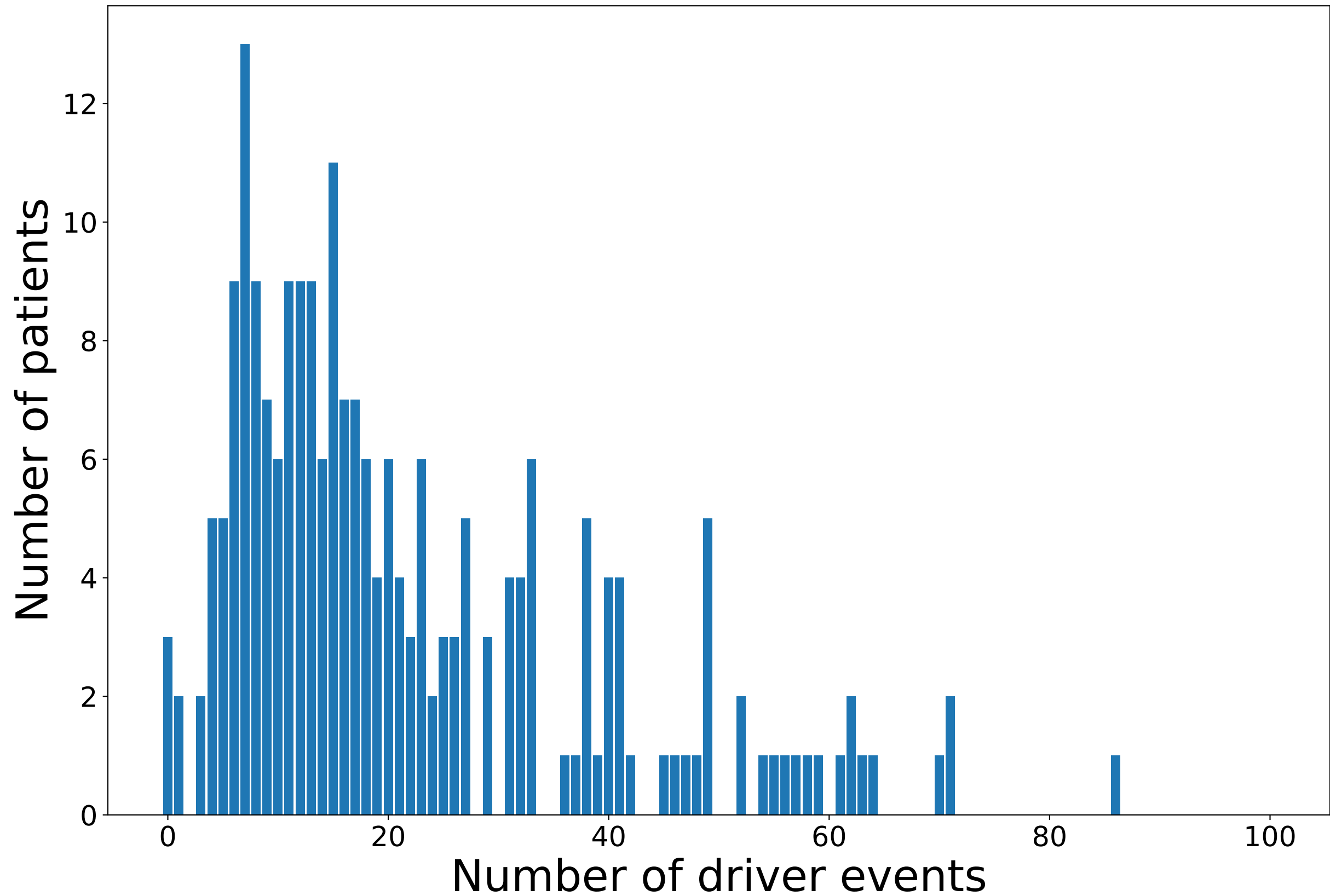

Supplement: S2 Files — (ZIP) [file pgen.1009996.s002.zip › PANCAN/patient distributions/2021_11_23_14_43_KIRC.pdf]

# BRCA\_FEMALE

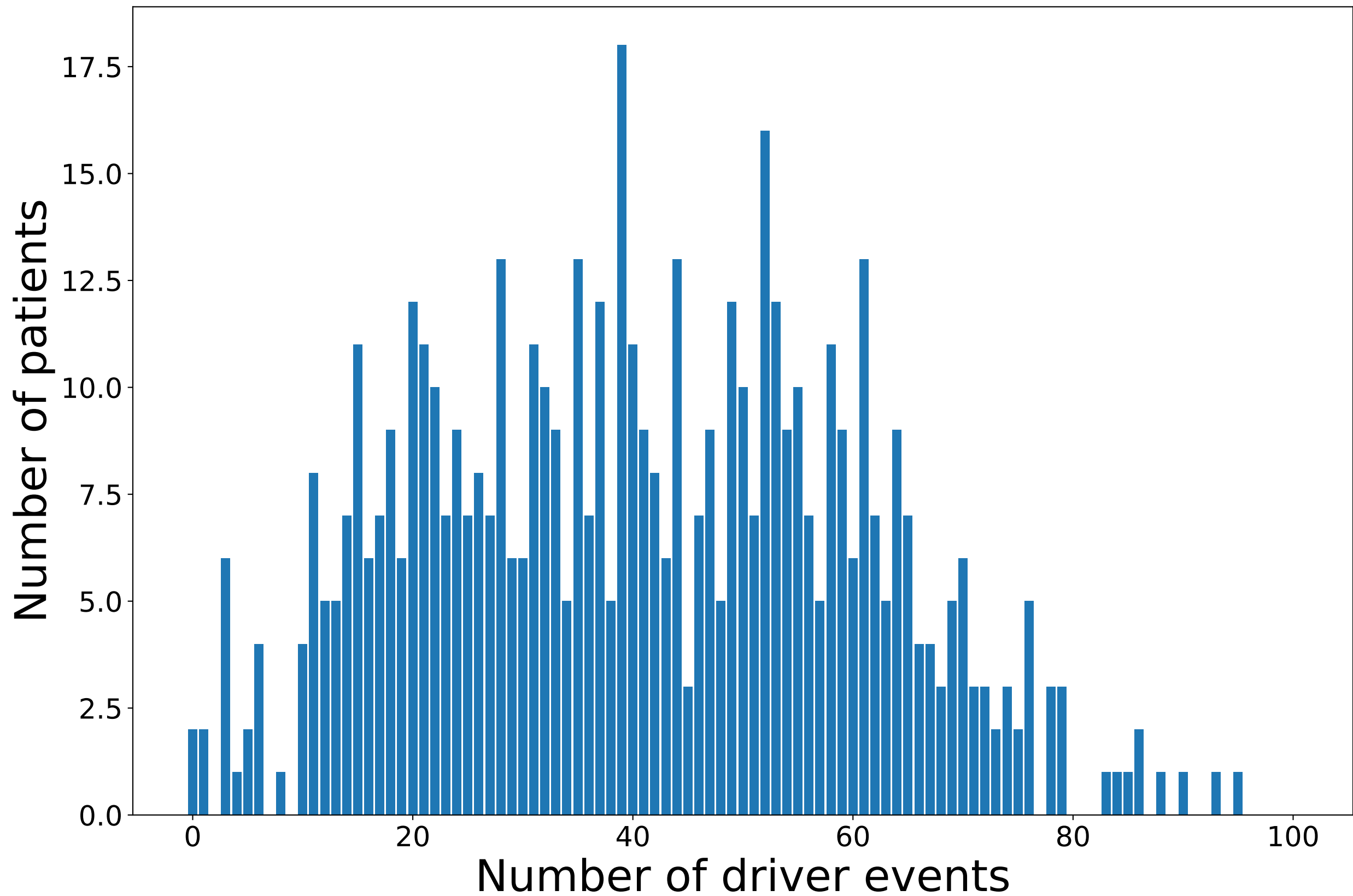

Supplement: S2 Files — (ZIP) [file pgen.1009996.s002.zip › PANCAN/patient distributions/2021_11_23_14_43_BRCA_FEMALE.pdf]

# UVM

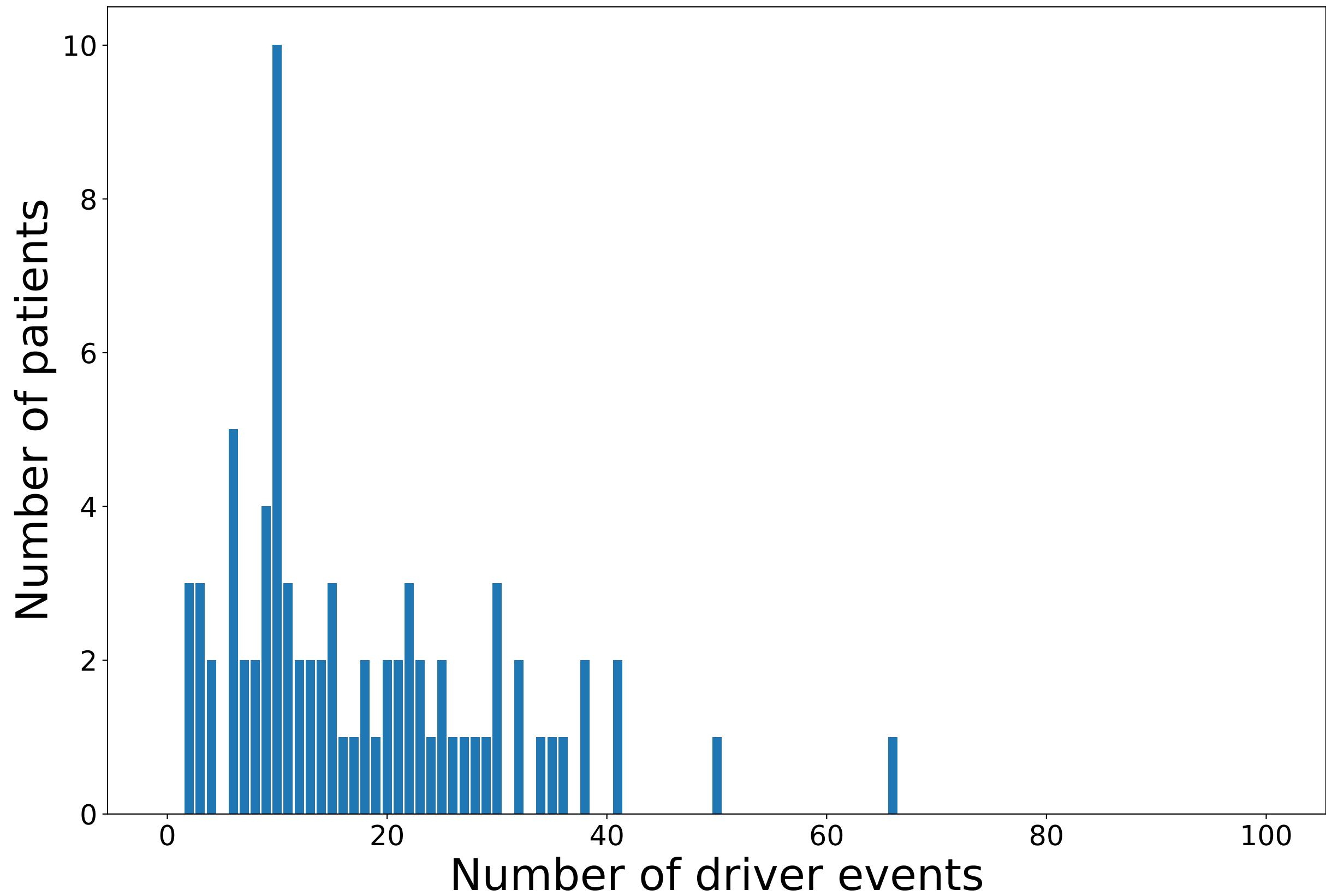

Supplement: S2 Files — (ZIP) [file pgen.1009996.s002.zip › PANCAN/patient distributions/2021_11_23_14_43_UVM.pdf]

# CESC\_FEMALE

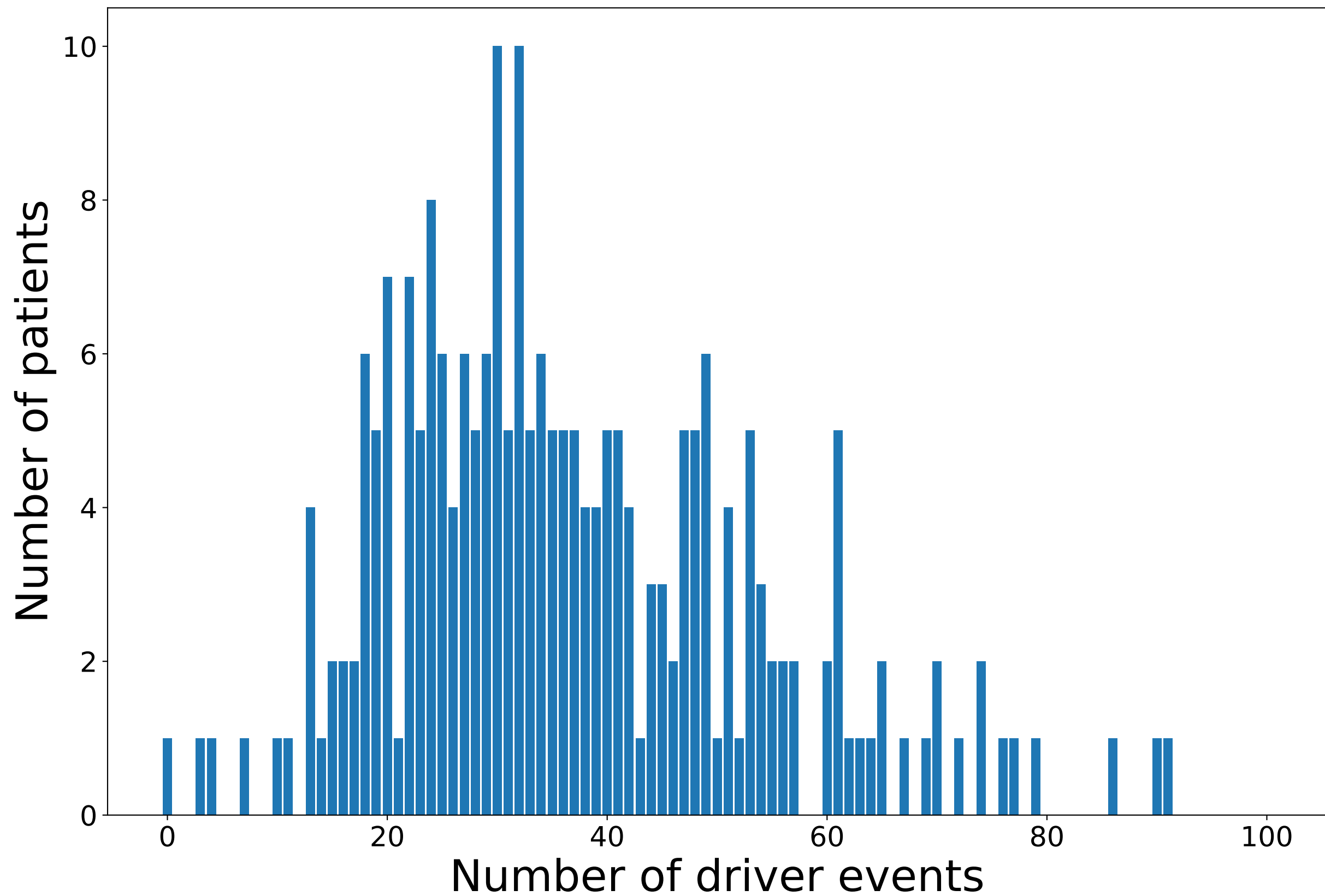

Supplement: S2 Files — (ZIP) [file pgen.1009996.s002.zip › PANCAN/patient distributions/2021_11_23_14_43_CESC_FEMALE.pdf]

# KICH\_MALE

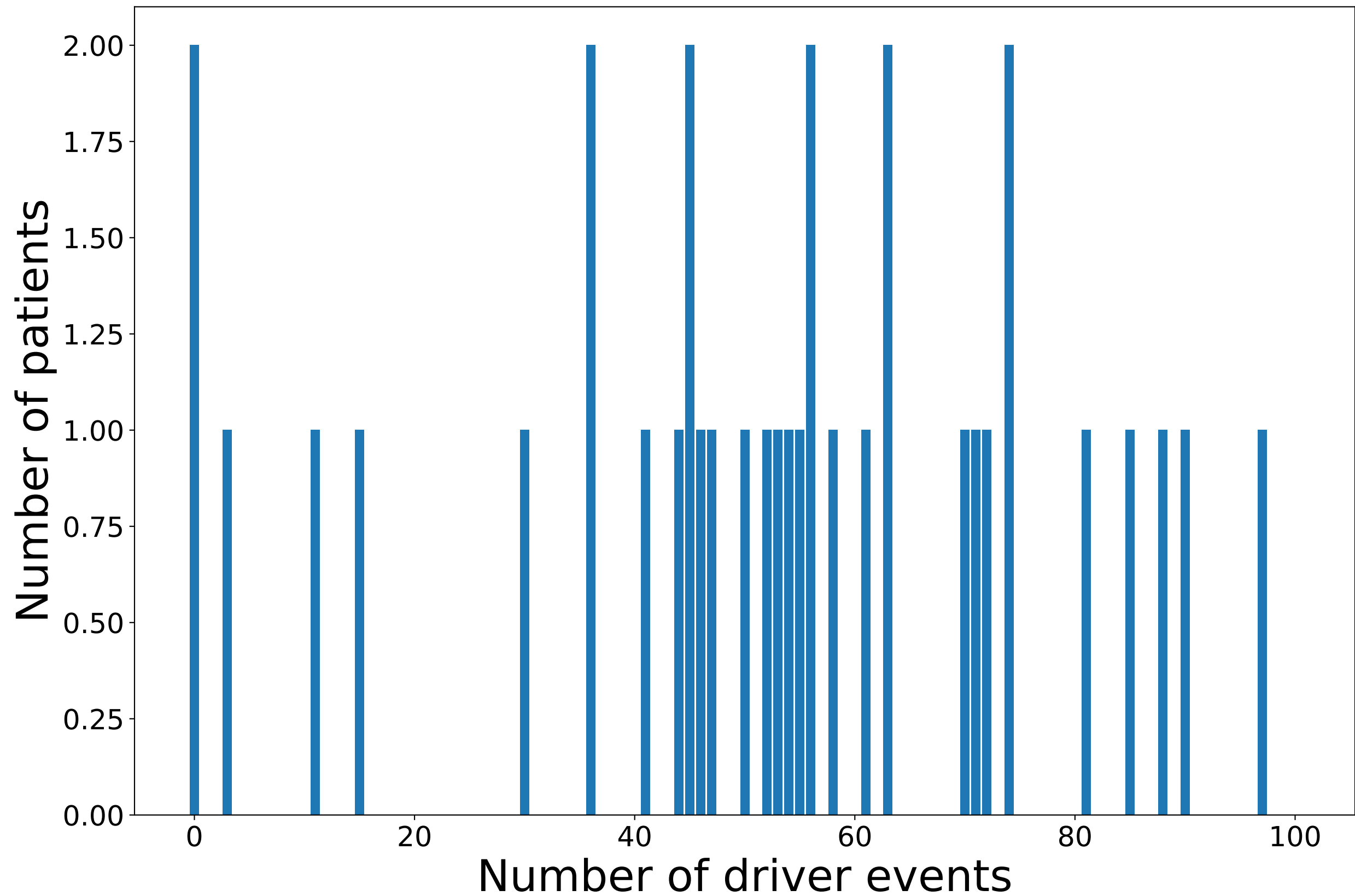

Supplement: S2 Files — (ZIP) [file pgen.1009996.s002.zip › PANCAN/patient distributions/2021_11_23_14_43_KICH_MALE.pdf]

# BRCA

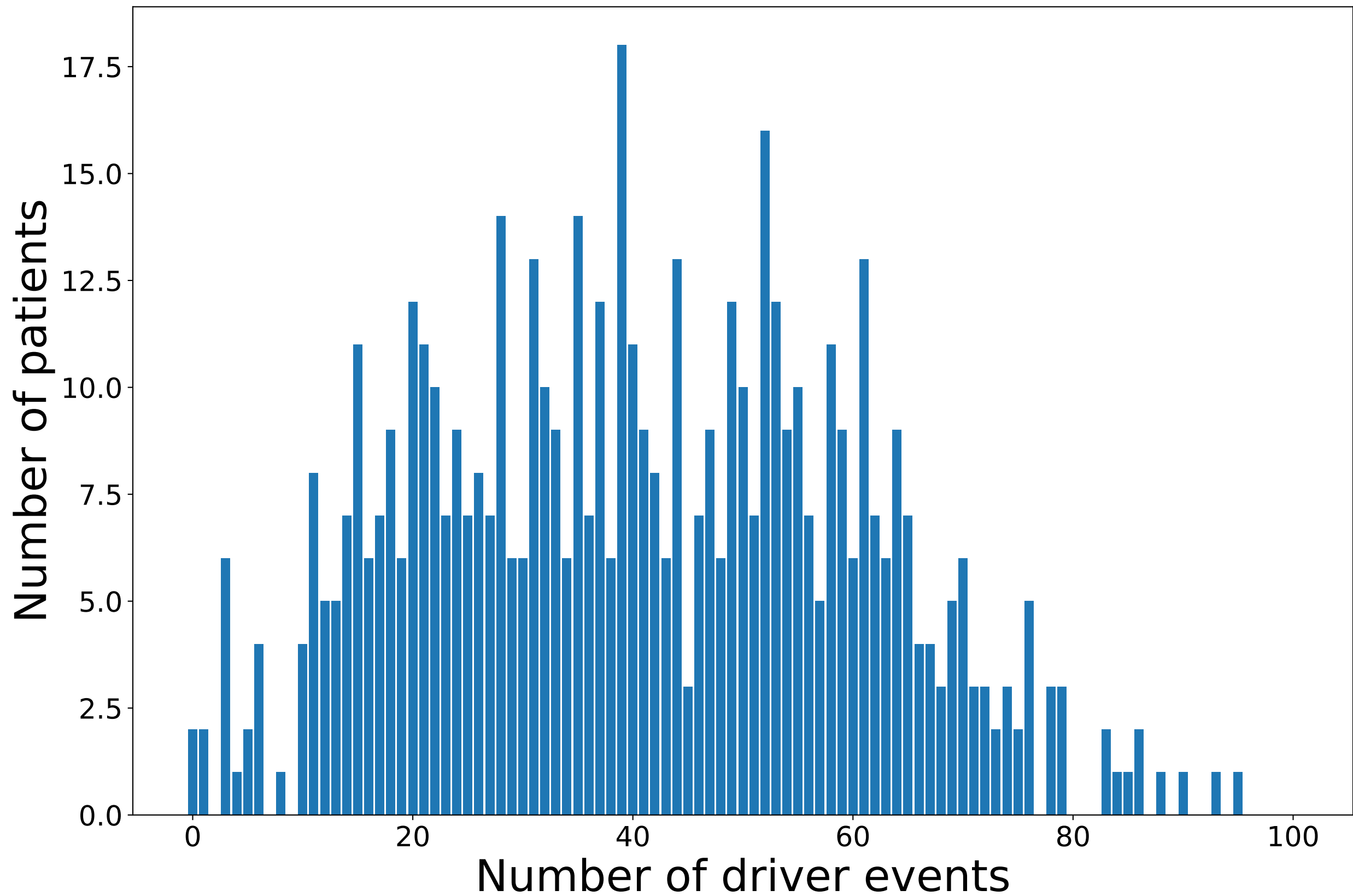

Supplement: S2 Files — (ZIP) [file pgen.1009996.s002.zip › PANCAN/patient distributions/2021_11_23_14_43_BRCA.pdf]

# THCA\_MALE

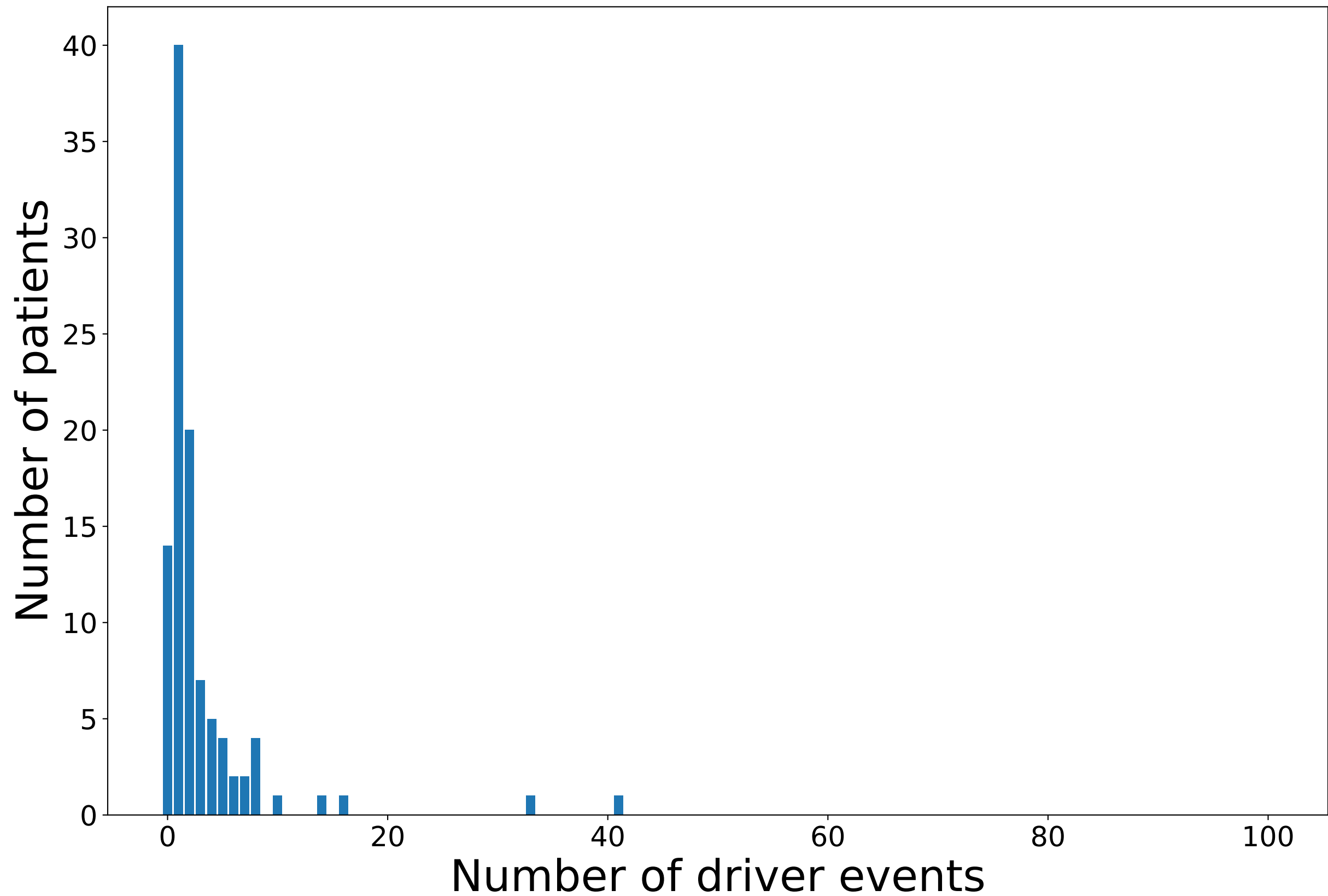

Supplement: S2 Files — (ZIP) [file pgen.1009996.s002.zip › PANCAN/patient distributions/2021_11_23_14_43_THCA_MALE.pdf]

OV

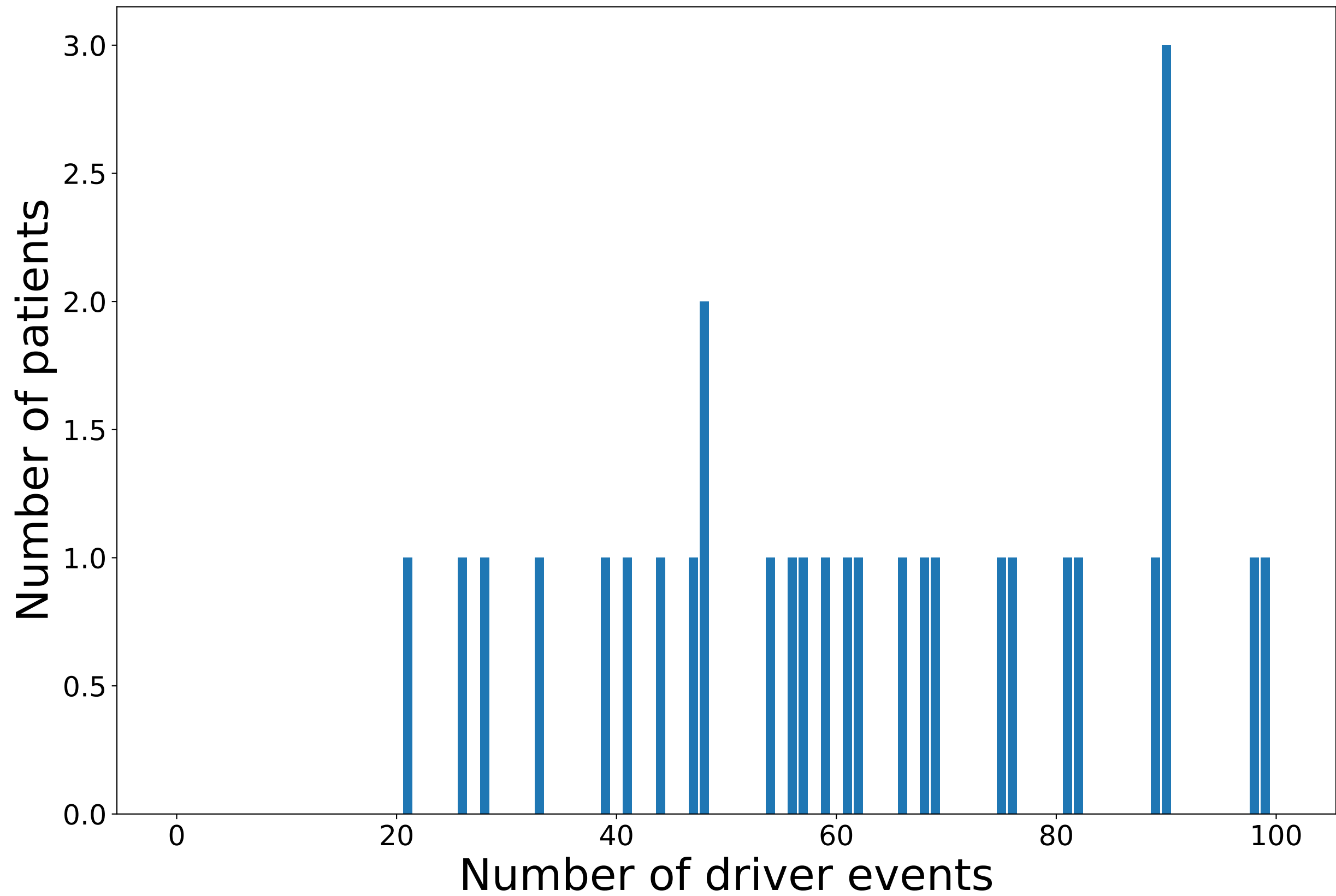

Supplement: S2 Files — (ZIP) [file pgen.1009996.s002.zip › PANCAN/patient distributions/2021_11_23_14_43_OV.pdf]

# PCPG\_MALE

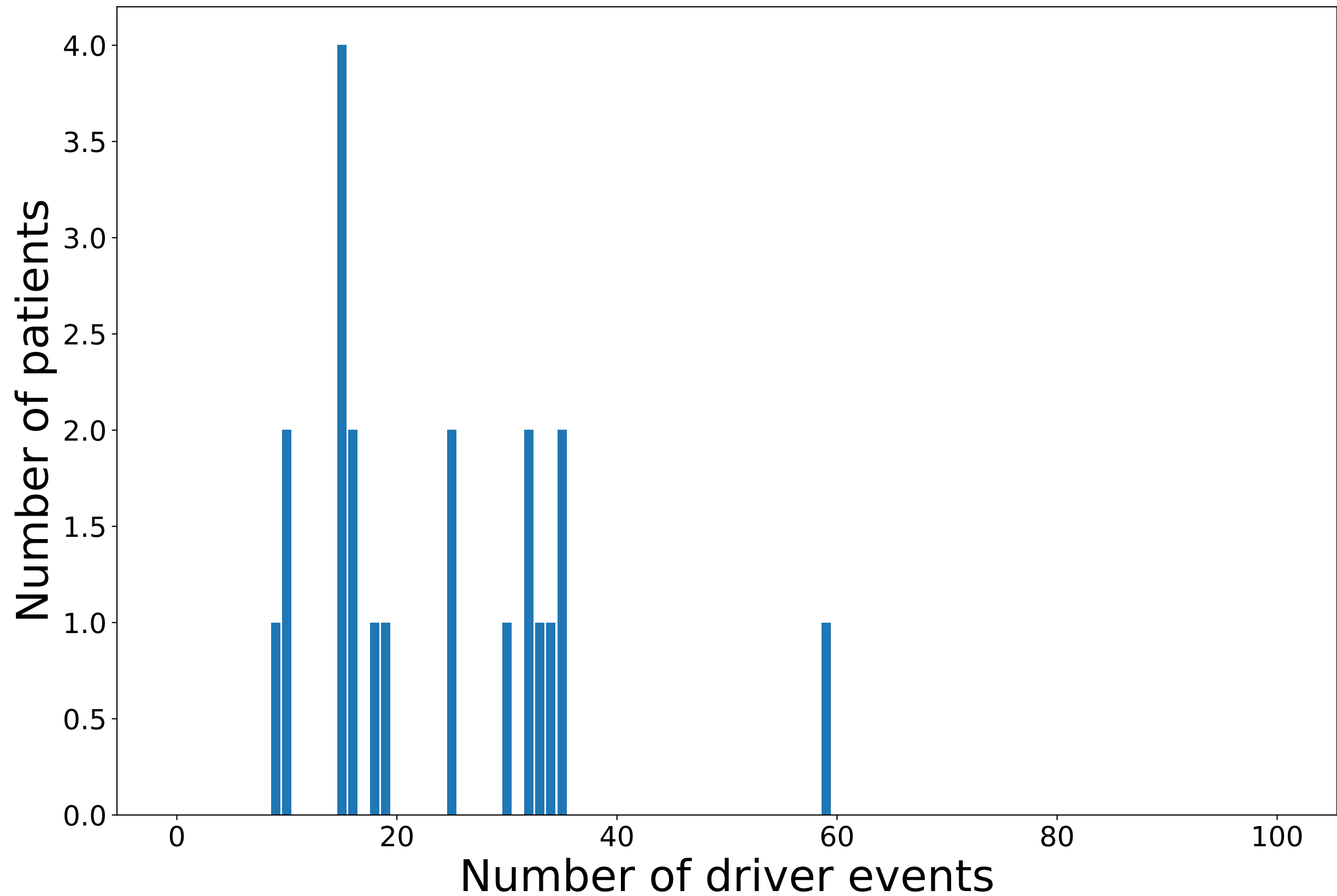

Supplement: S2 Files — (ZIP) [file pgen.1009996.s002.zip › PANCAN/patient distributions/2021_11_23_14_43_PCPG_MALE.pdf]

# PCPG\_FEMALE

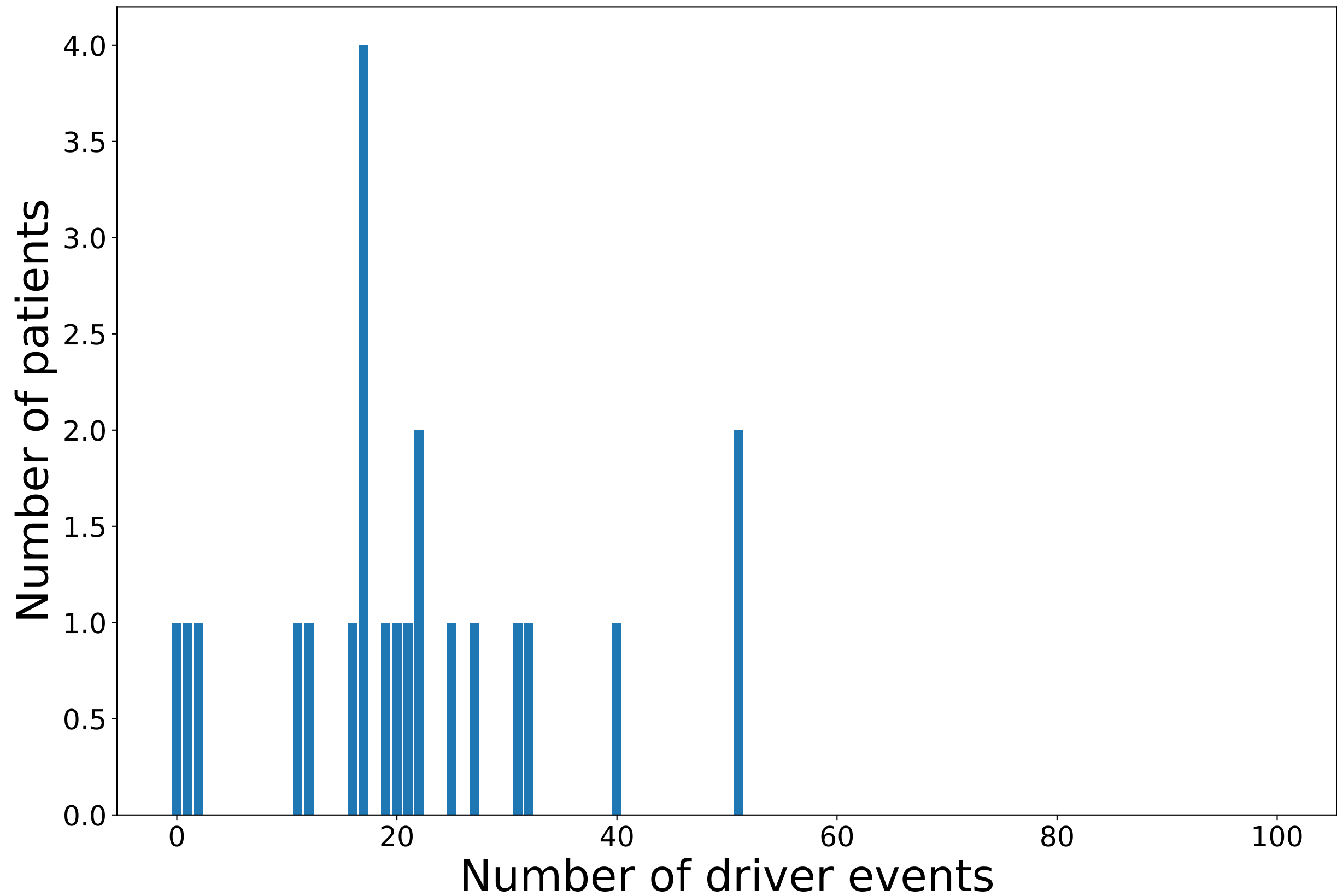

Supplement: S2 Files — (ZIP) [file pgen.1009996.s002.zip › PANCAN/patient distributions/2021_11_23_14_43_PCPG_FEMALE.pdf]

# UCS\_FEMALE

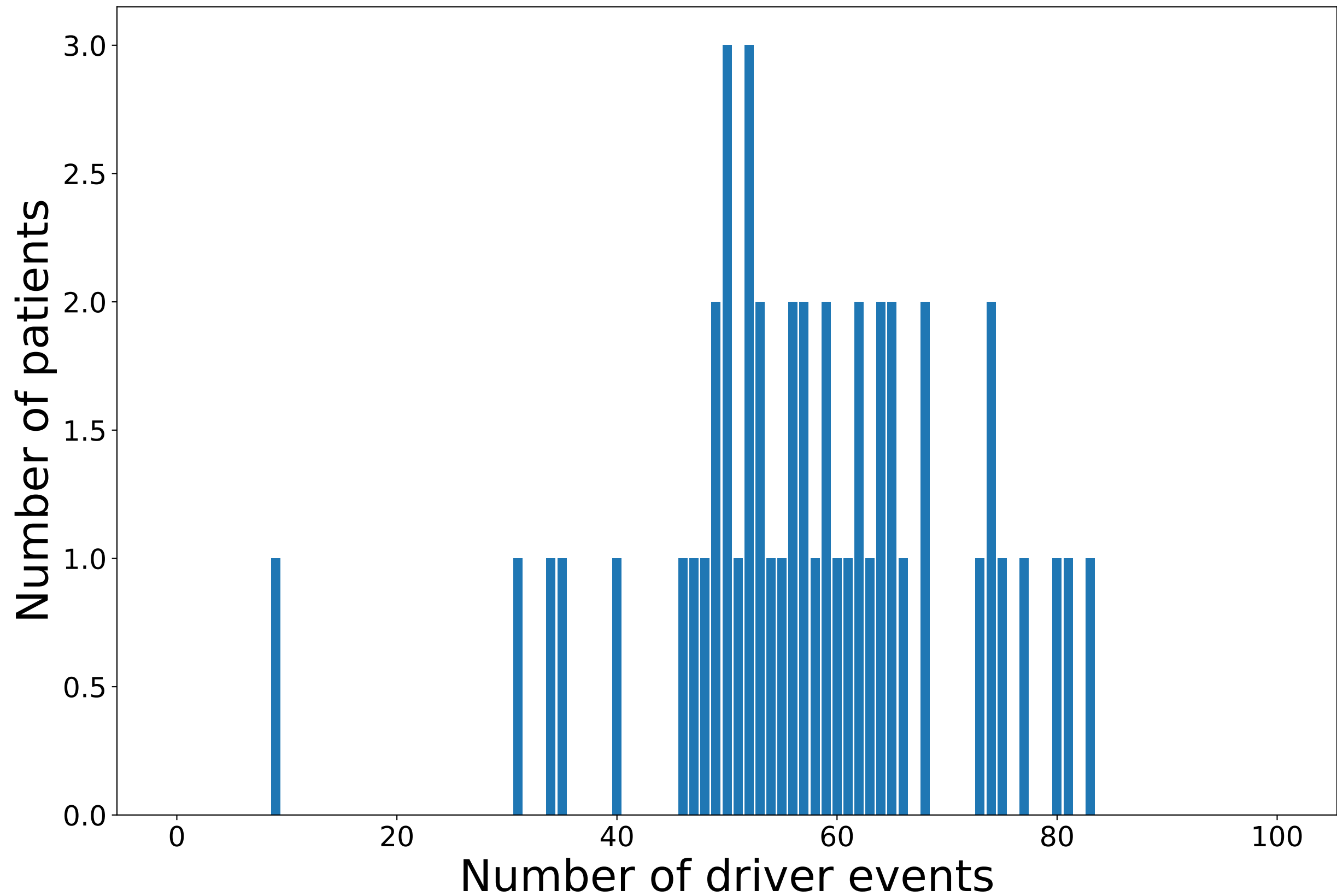

Supplement: S2 Files — (ZIP) [file pgen.1009996.s002.zip › PANCAN/patient distributions/2021_11_23_14_43_UCS_FEMALE.pdf]

# READ\_MALE

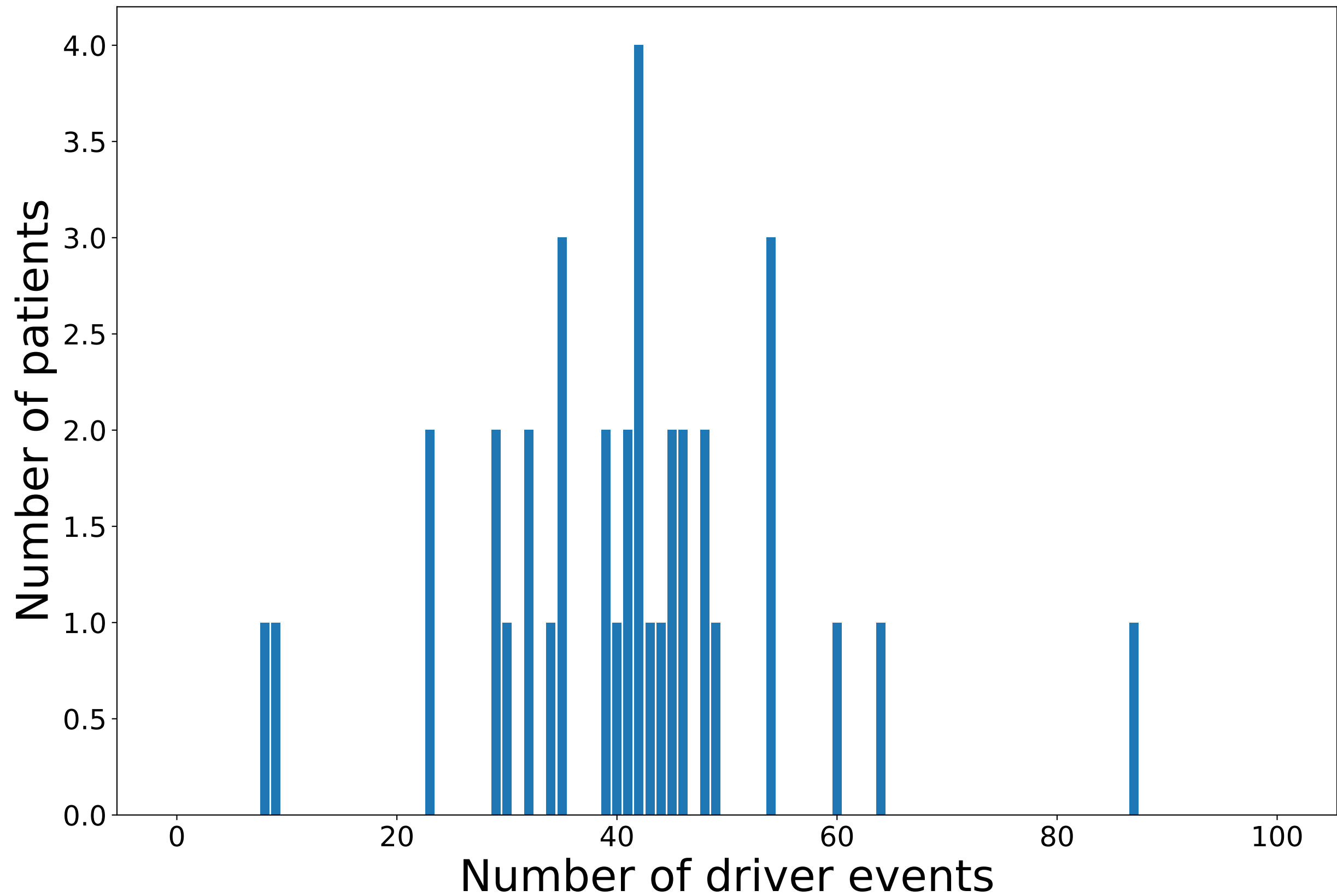

Supplement: S2 Files — (ZIP) [file pgen.1009996.s002.zip › PANCAN/patient distributions/2021_11_23_14_43_READ_MALE.pdf]

# MESO

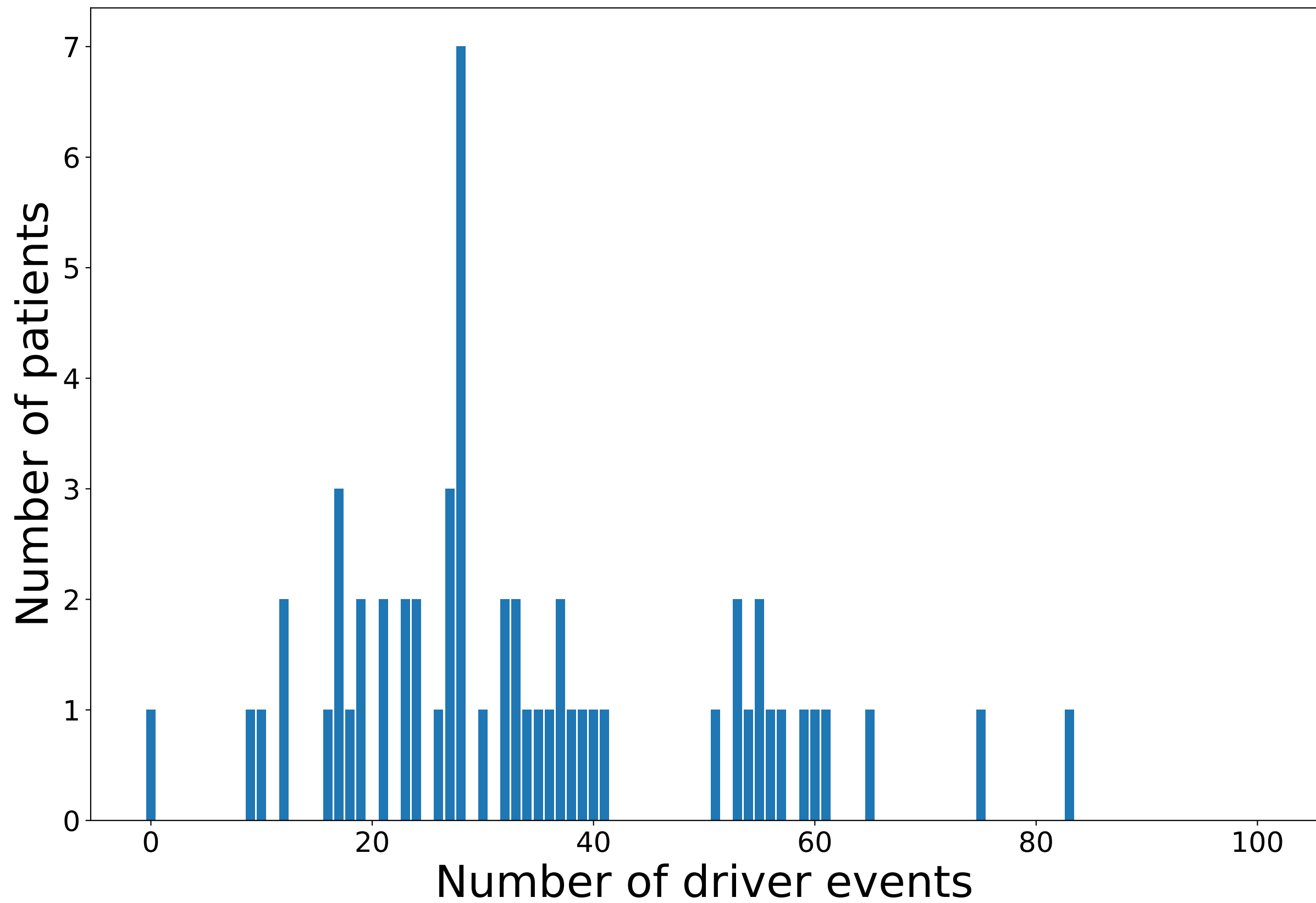

Supplement: S2 Files — (ZIP) [file pgen.1009996.s002.zip › PANCAN/patient distributions/2021_11_23_14_43_MESO.pdf]

# DLBC

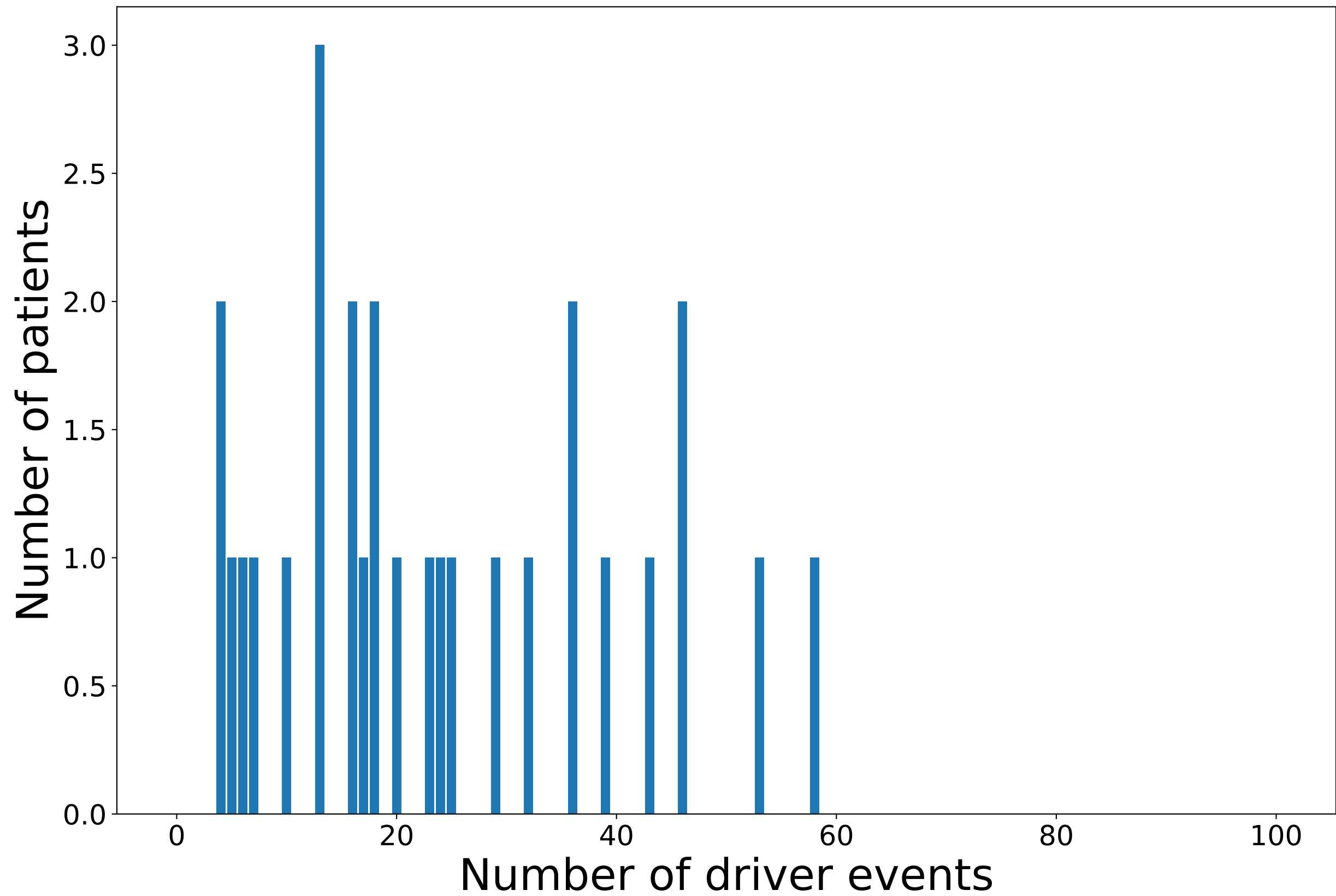

Supplement: S2 Files — (ZIP) [file pgen.1009996.s002.zip › PANCAN/patient distributions/2021_11_23_14_43_DLBC.pdf]

# SKCM

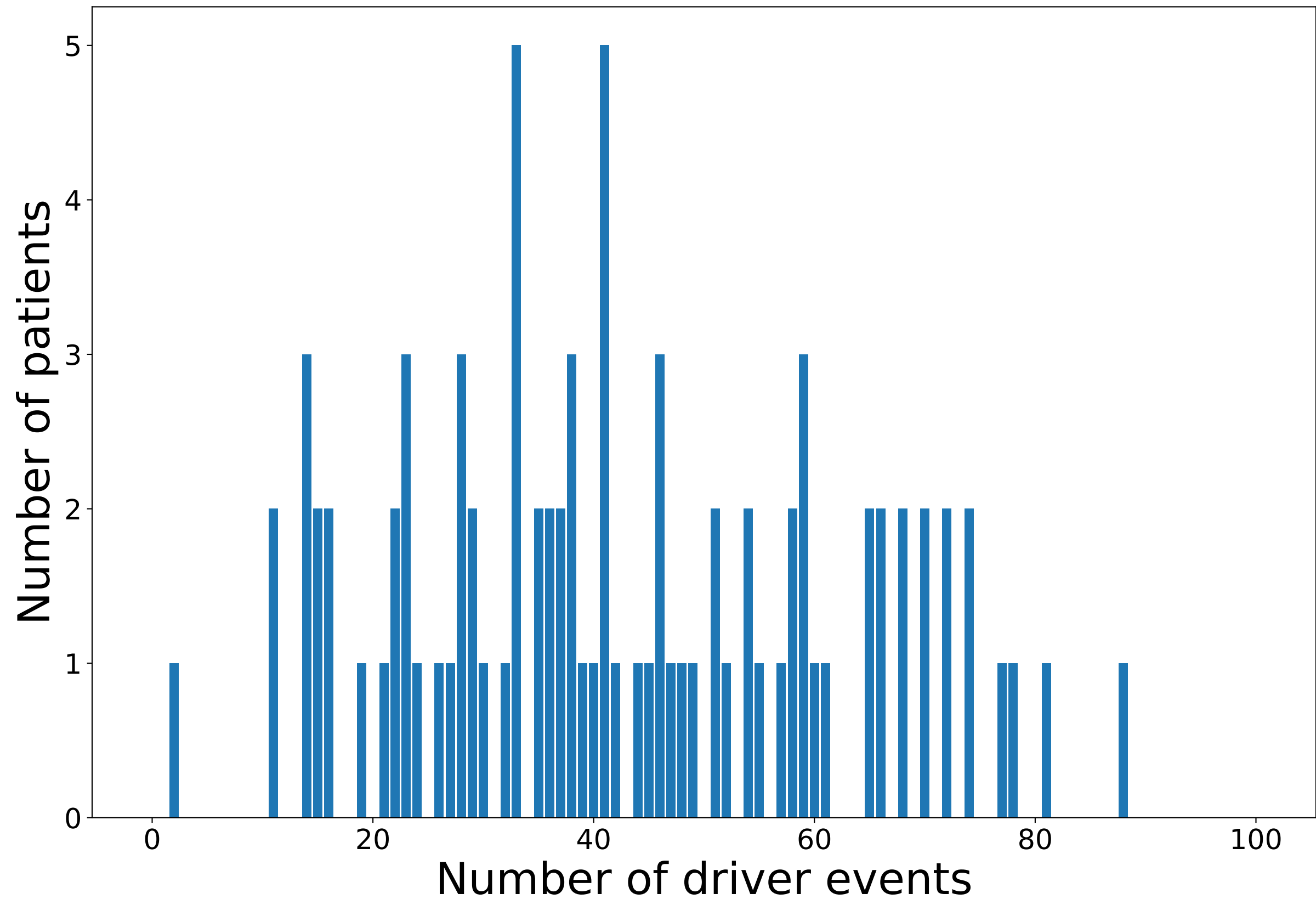

Supplement: S2 Files — (ZIP) [file pgen.1009996.s002.zip › PANCAN/patient distributions/2021_11_23_14_43_SKCM.pdf]

# LUSC\_FEMALE

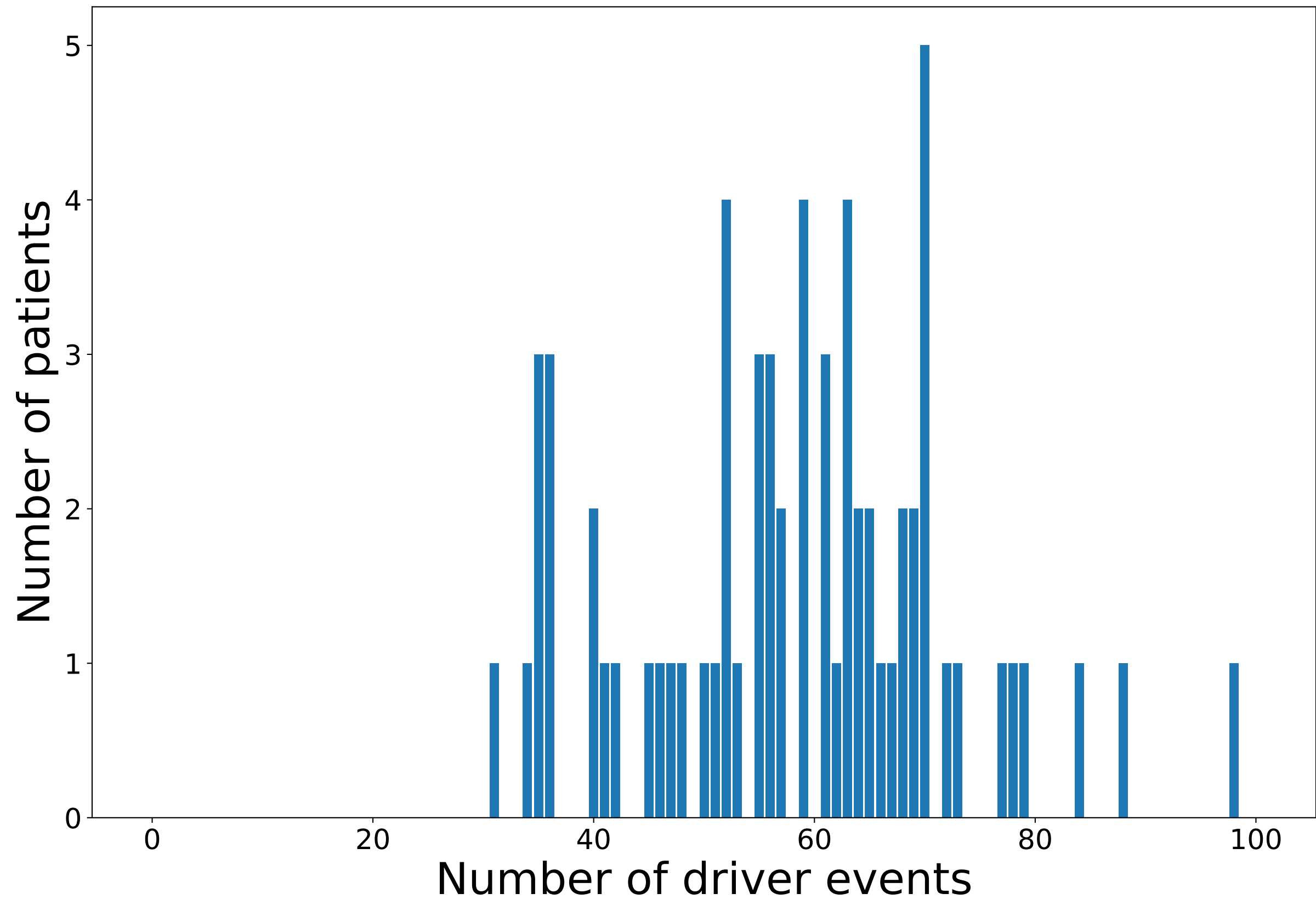

Supplement: S2 Files — (ZIP) [file pgen.1009996.s002.zip › PANCAN/patient distributions/2021_11_23_14_43_LUSC_FEMALE.pdf]

# UCEC\_FEMALE

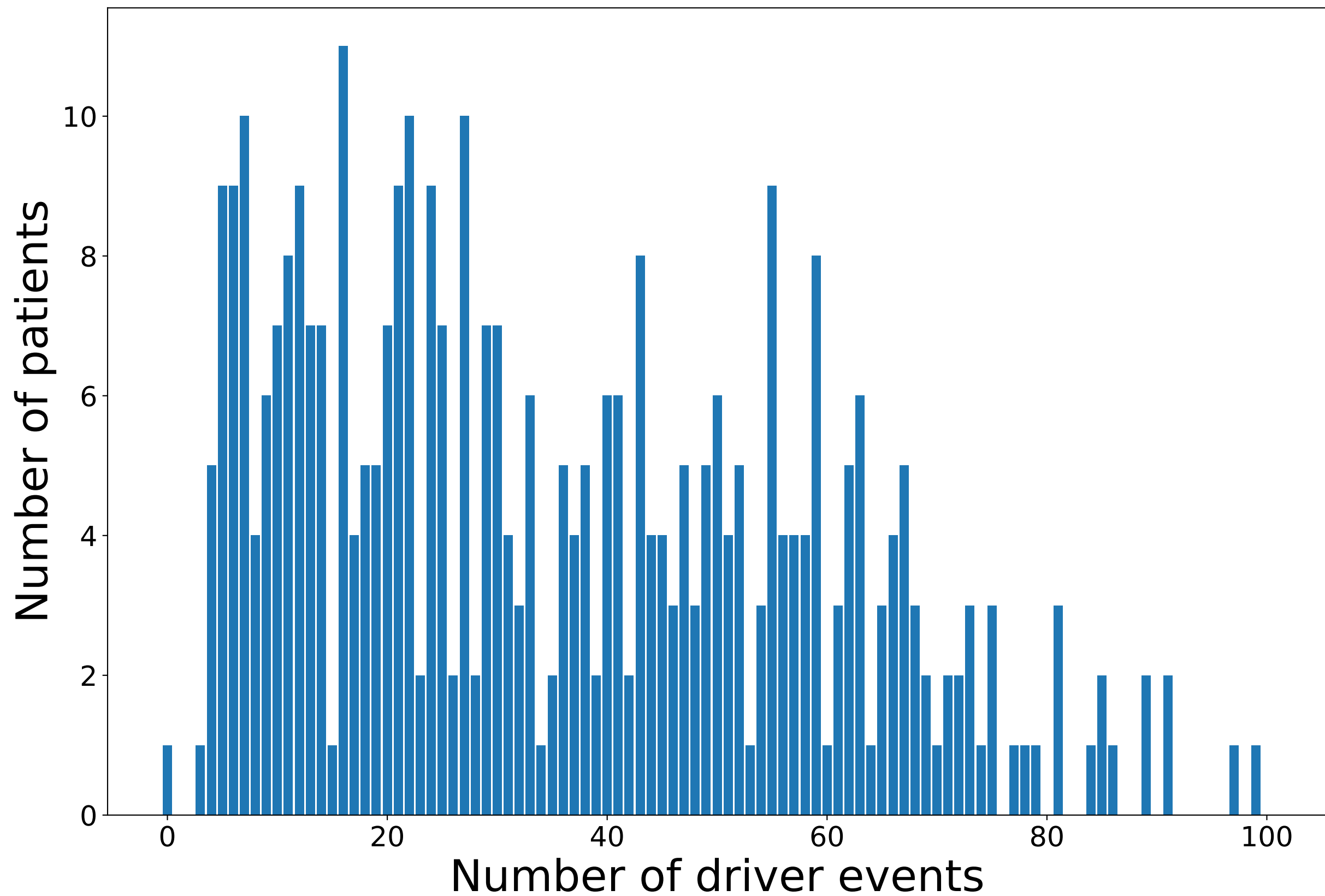

Supplement: S2 Files — (ZIP) [file pgen.1009996.s002.zip › PANCAN/patient distributions/2021_11_23_14_43_UCEC_FEMALE.pdf]

# STAD

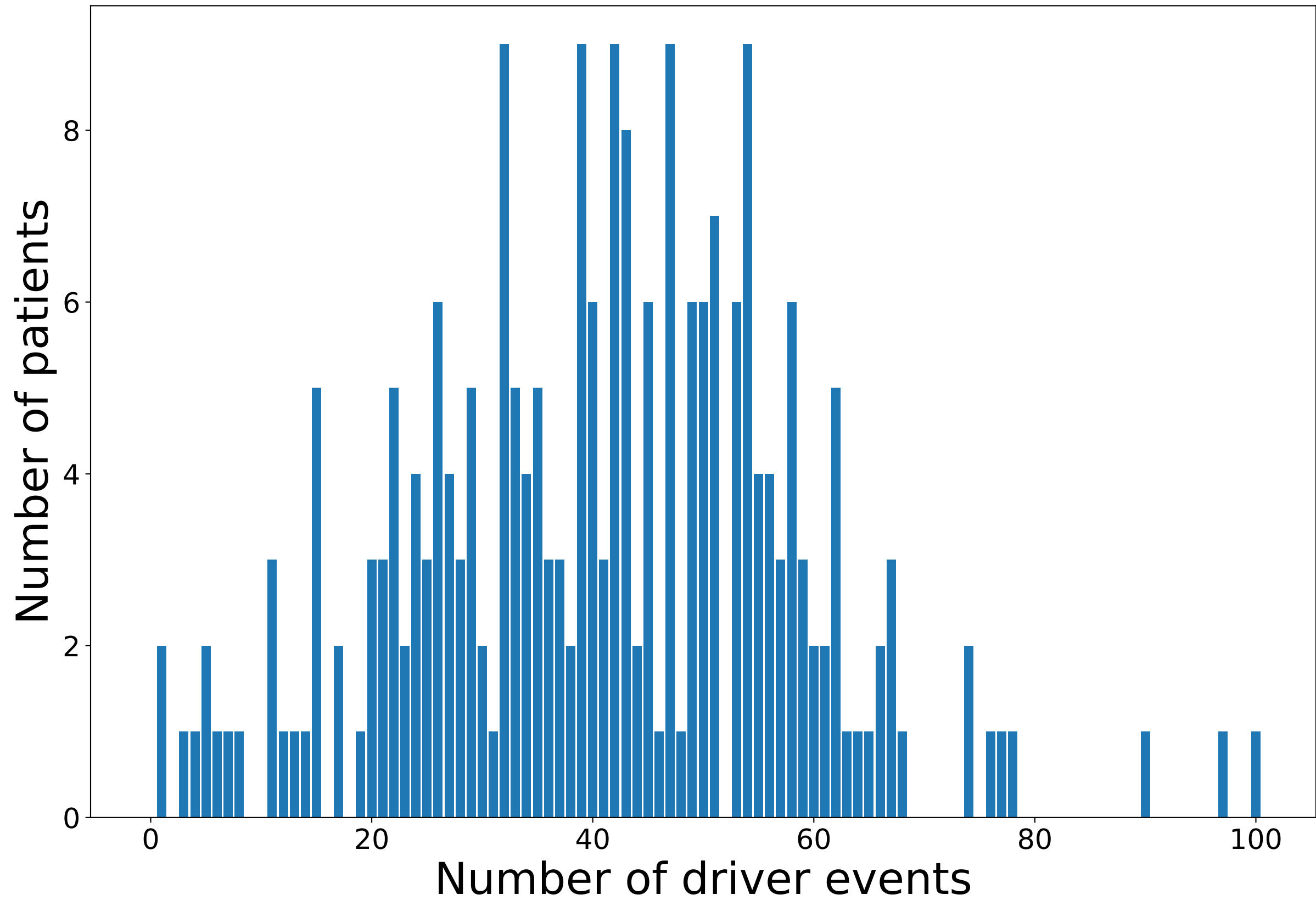

Supplement: S2 Files — (ZIP) [file pgen.1009996.s002.zip › PANCAN/patient distributions/2021_11_23_14_43_STAD.pdf]

# LGG

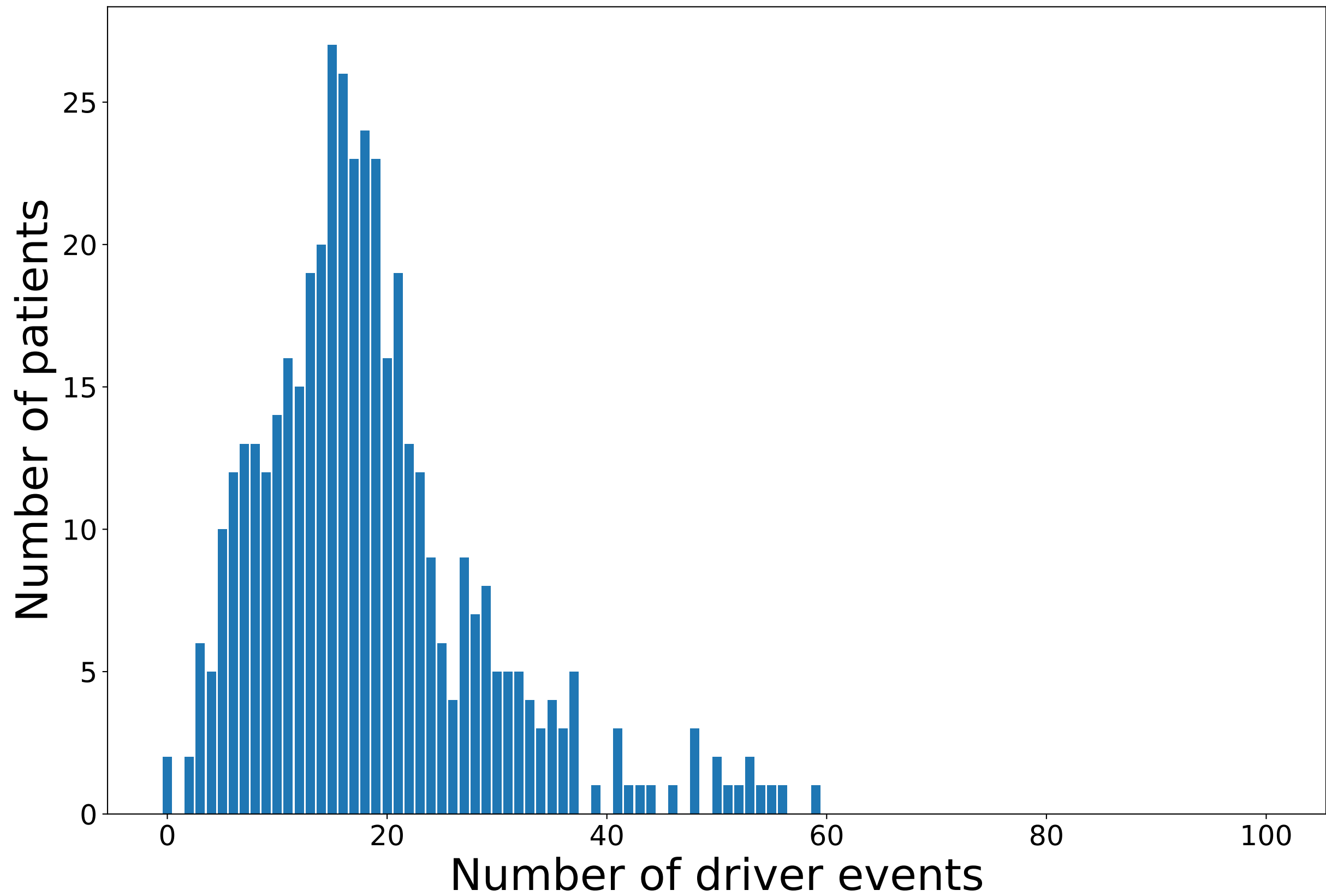

Supplement: S2 Files — (ZIP) [file pgen.1009996.s002.zip › PANCAN/patient distributions/2021_11_23_14_43_LGG.pdf]

# CHOL

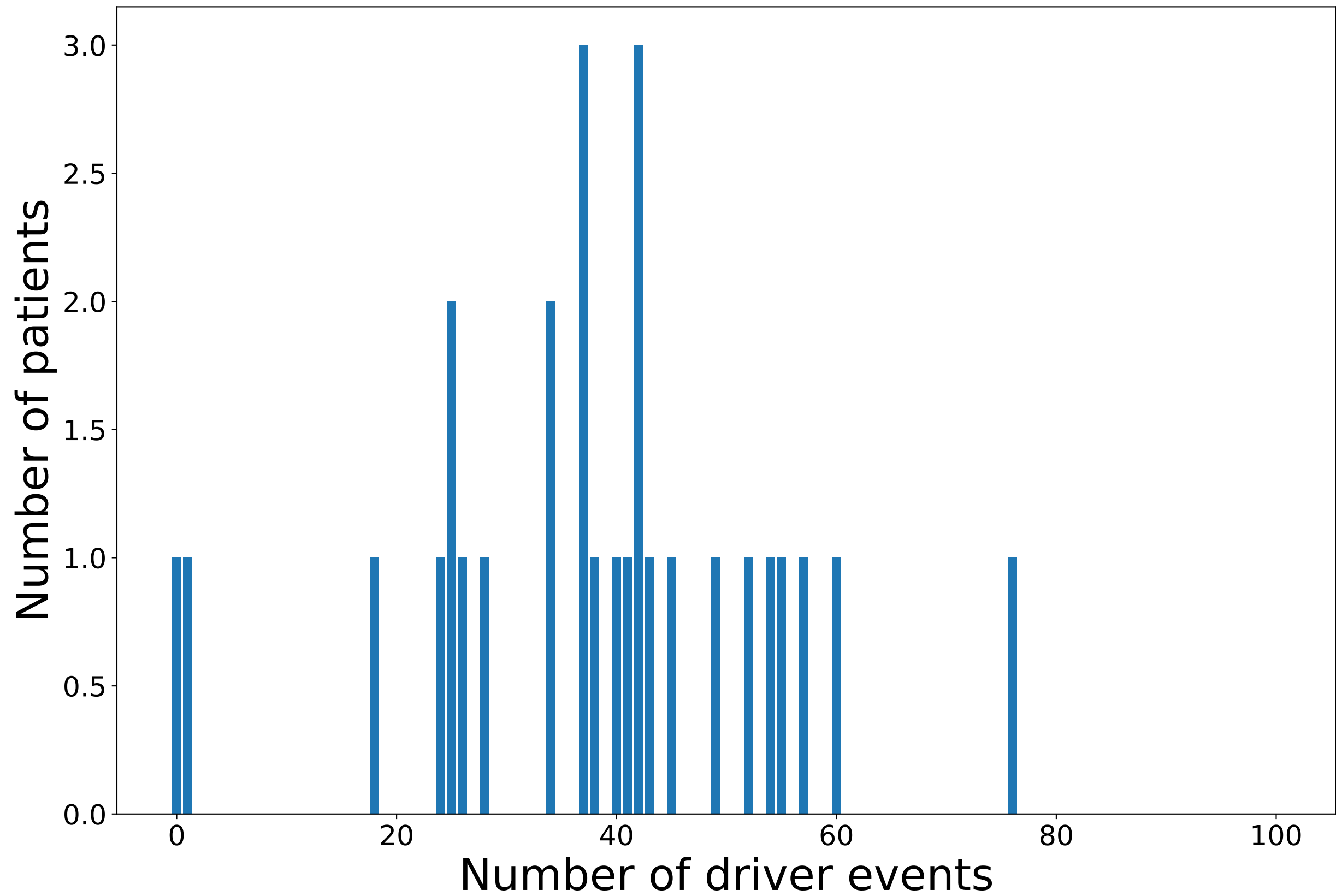

Supplement: S2 Files — (ZIP) [file pgen.1009996.s002.zip › PANCAN/patient distributions/2021_11_23_14_43_CHOL.pdf]

# ACC

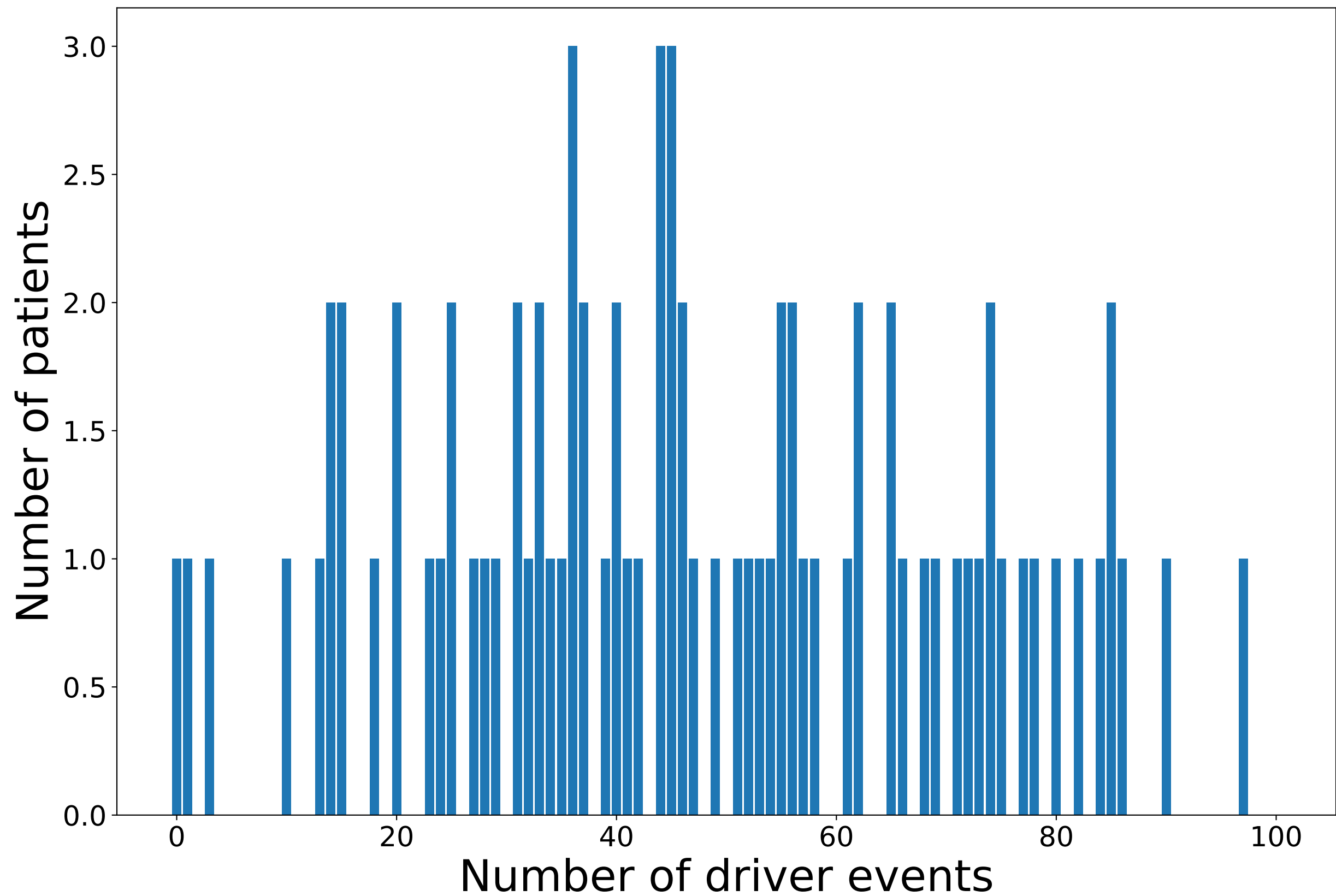

Supplement: S2 Files — (ZIP) [file pgen.1009996.s002.zip › PANCAN/patient distributions/2021_11_23_14_43_ACC.pdf]

# THYM\_FEMALE

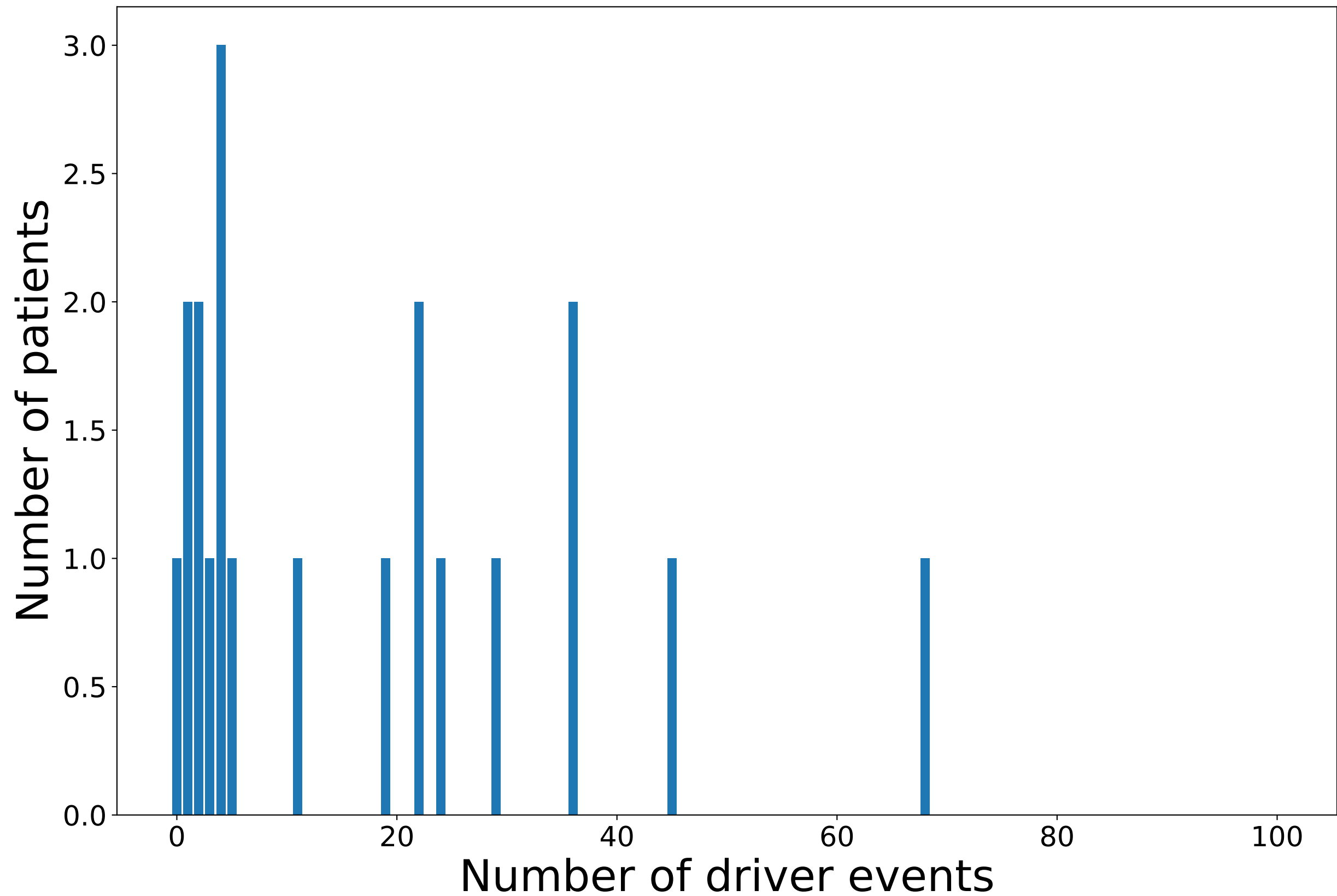

Supplement: S2 Files — (ZIP) [file pgen.1009996.s002.zip › PANCAN/patient distributions/2021_11_23_14_43_THYM_FEMALE.pdf]

# ACC\_MALE

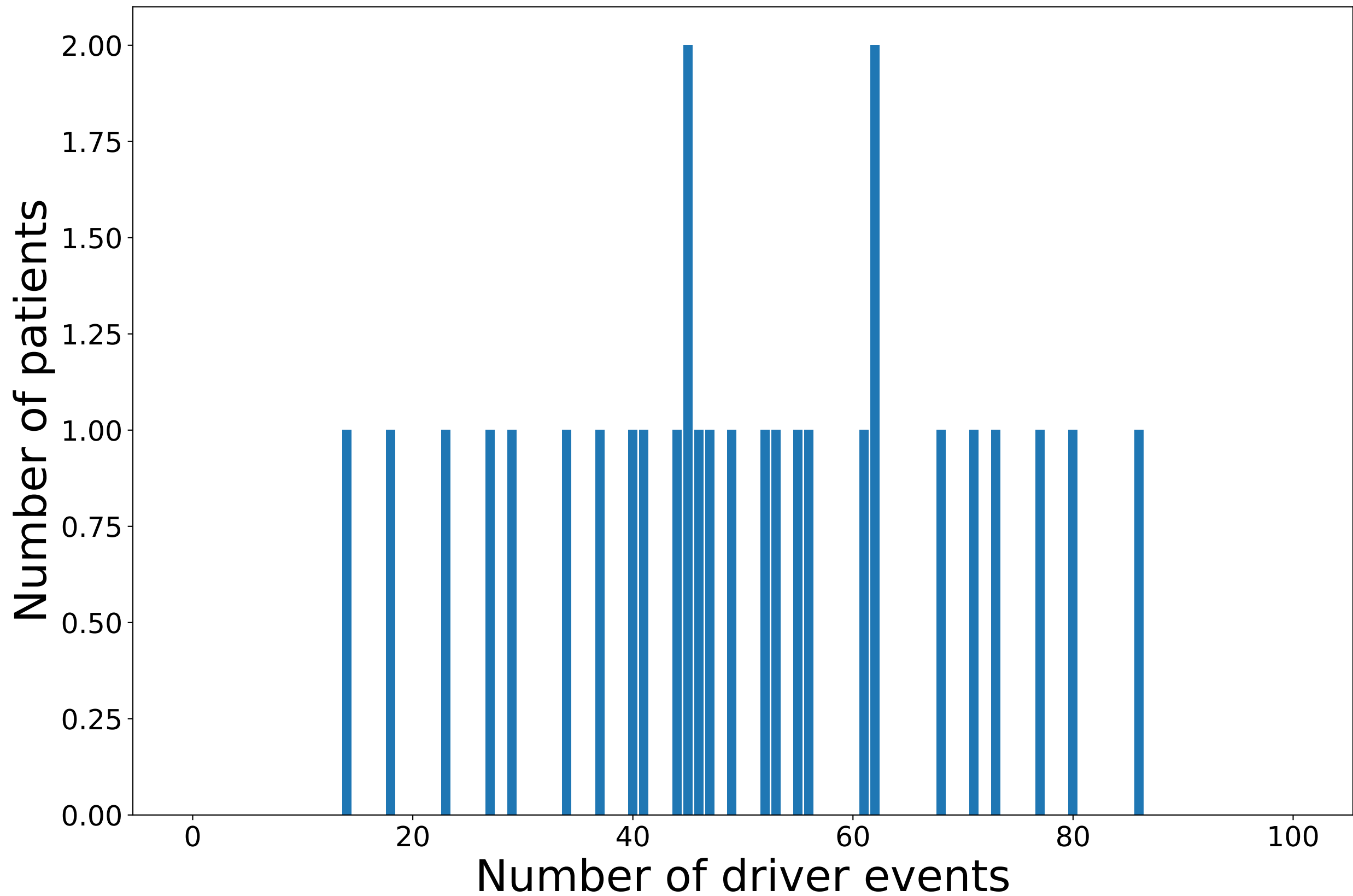

Supplement: S2 Files — (ZIP) [file pgen.1009996.s002.zip › PANCAN/patient distributions/2021_11_23_14_43_ACC_MALE.pdf]

# KICH

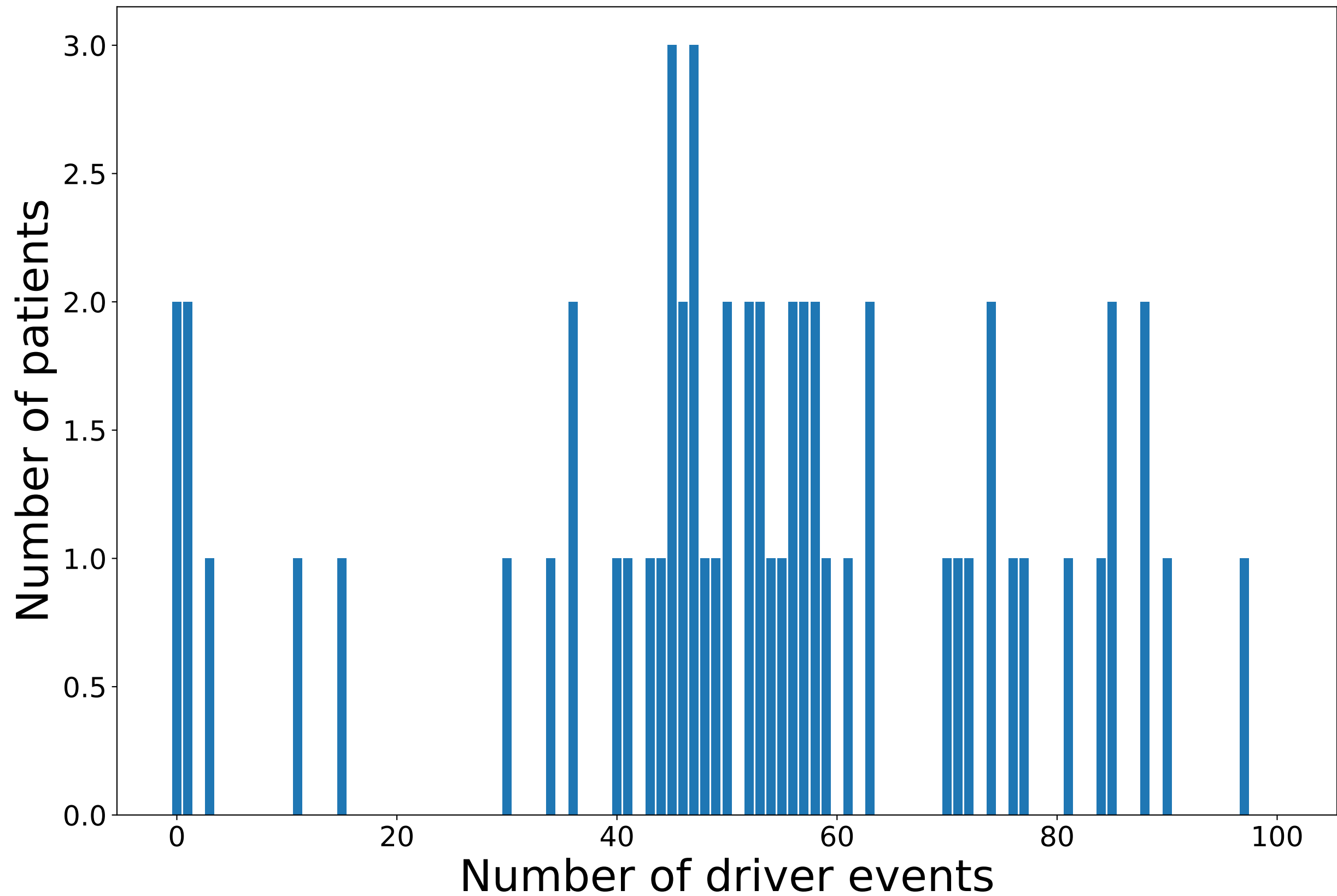

Supplement: S2 Files — (ZIP) [file pgen.1009996.s002.zip › PANCAN/patient distributions/2021_11_23_14_43_KICH.pdf]

# PANCAN

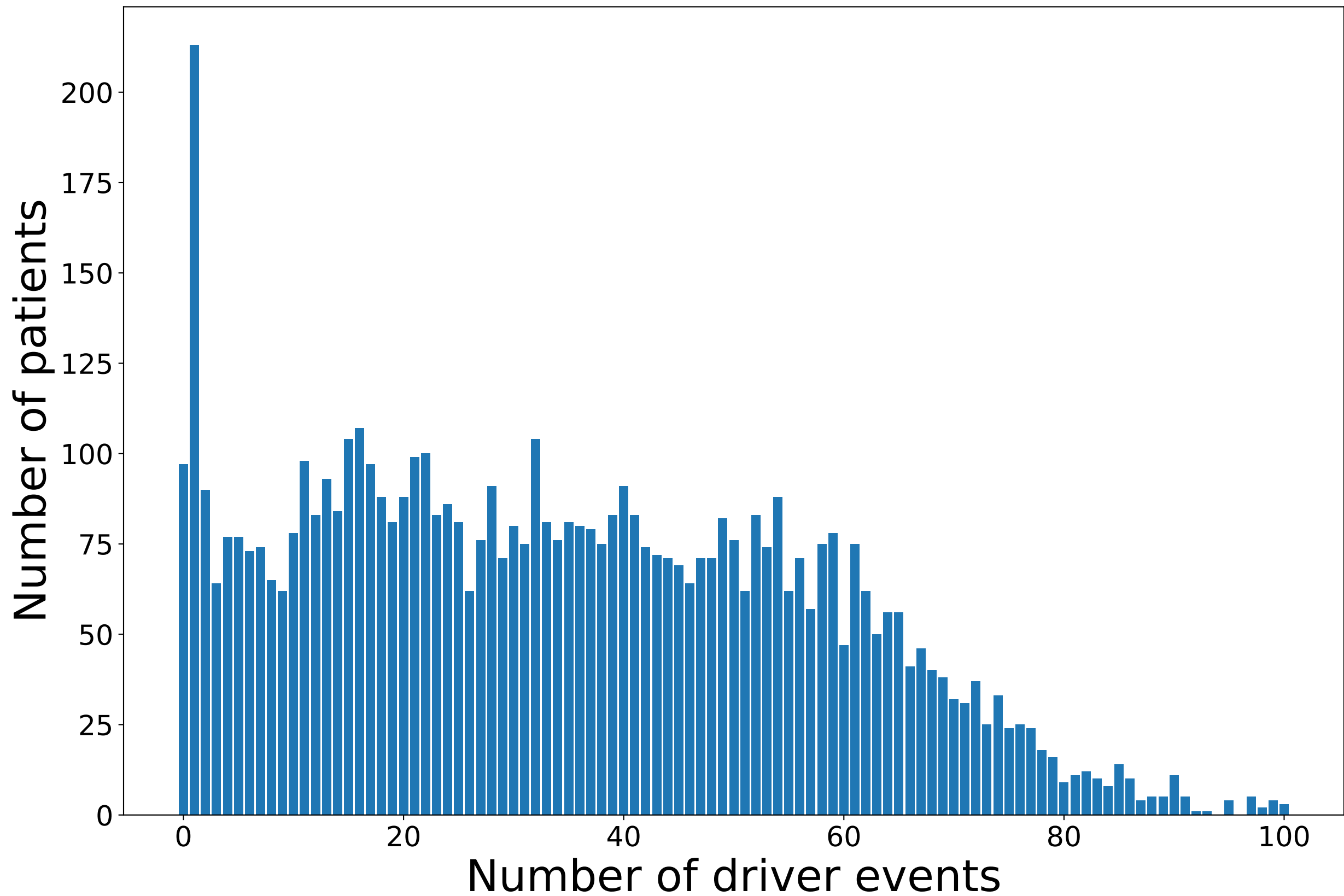

Supplement: S2 Files — (ZIP) [file pgen.1009996.s002.zip › PANCAN/patient distributions/2021_11_23_14_43_PANCAN.pdf]

# UCEC

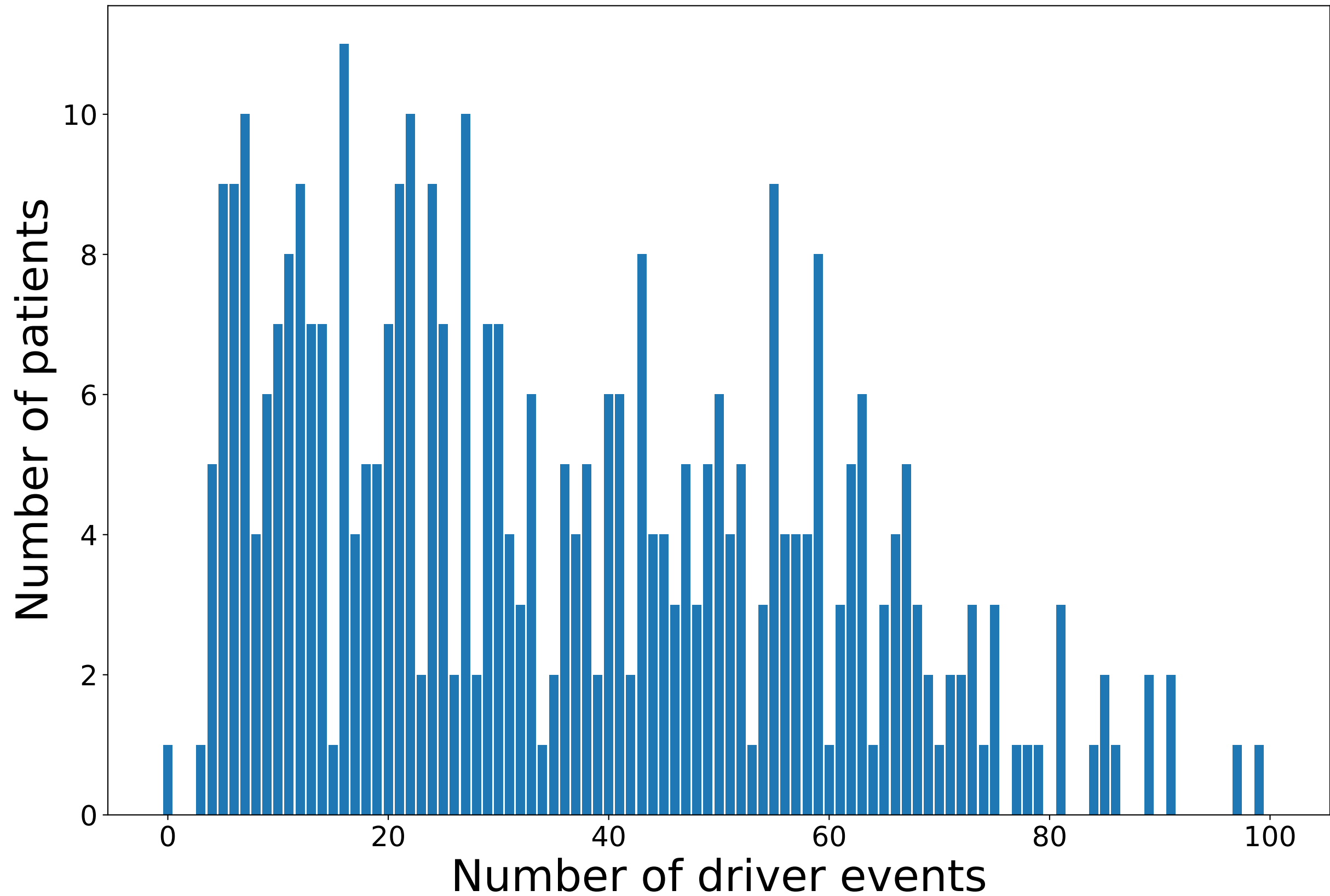

Supplement: S2 Files — (ZIP) [file pgen.1009996.s002.zip › PANCAN/patient distributions/2021_11_23_14_43_UCEC.pdf]

# DLBC\_MALE

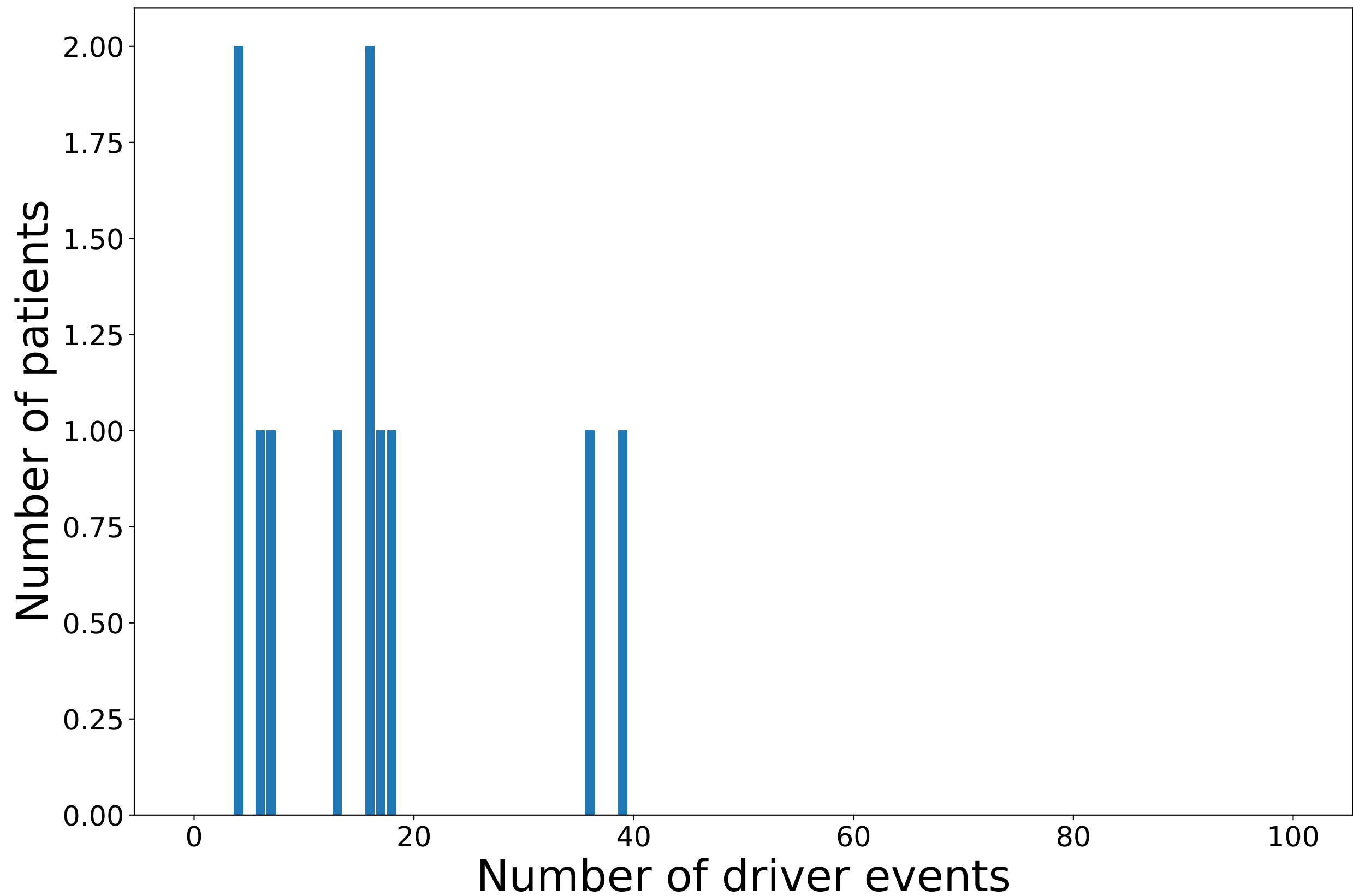

Supplement: S2 Files — (ZIP) [file pgen.1009996.s002.zip › PANCAN/patient distributions/2021_11_23_14_43_DLBC_MALE.pdf]

# MESO\_FEMALE

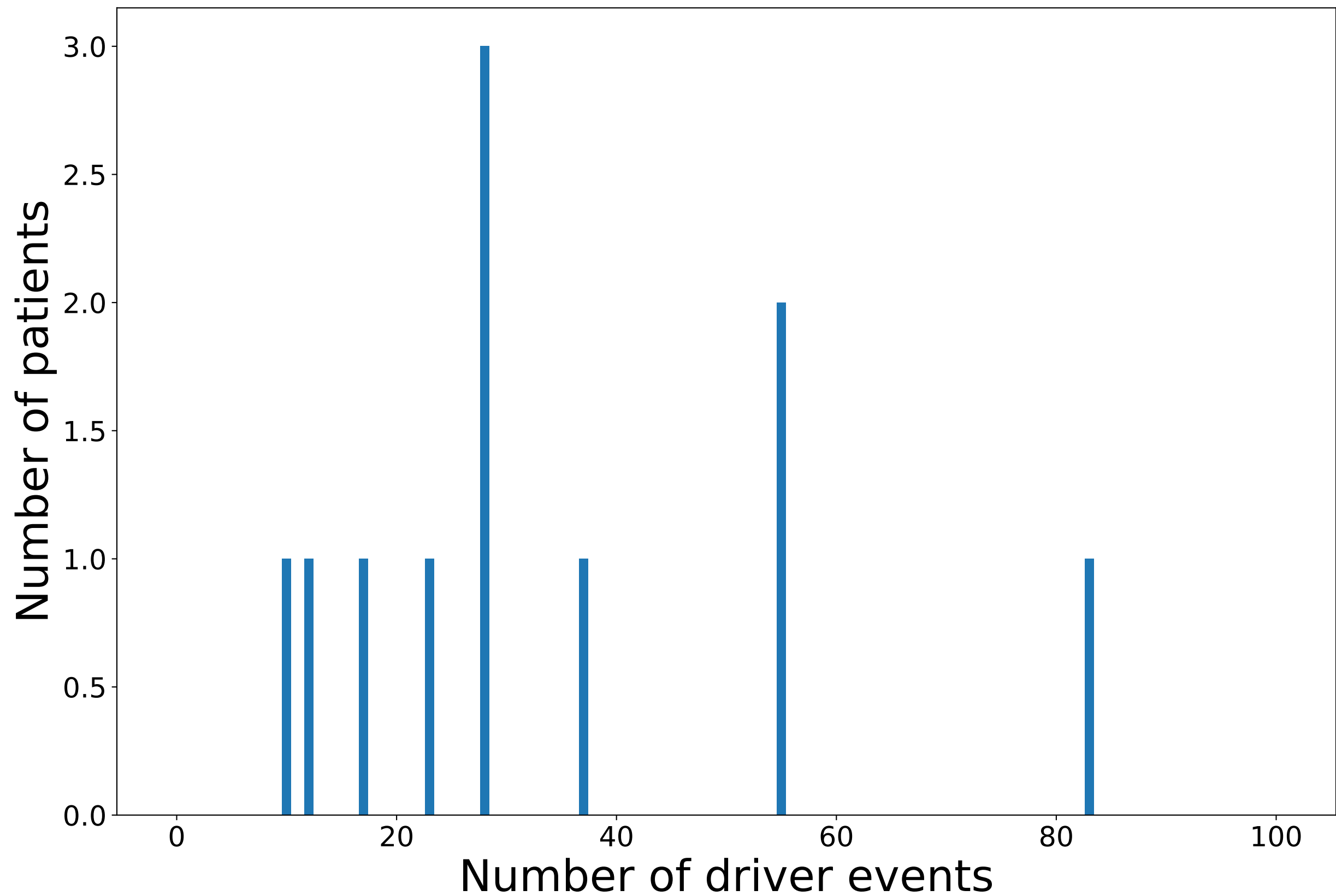

Supplement: S2 Files — (ZIP) [file pgen.1009996.s002.zip › PANCAN/patient distributions/2021_11_23_14_43_MESO_FEMALE.pdf]

# PANCAN\_MALE

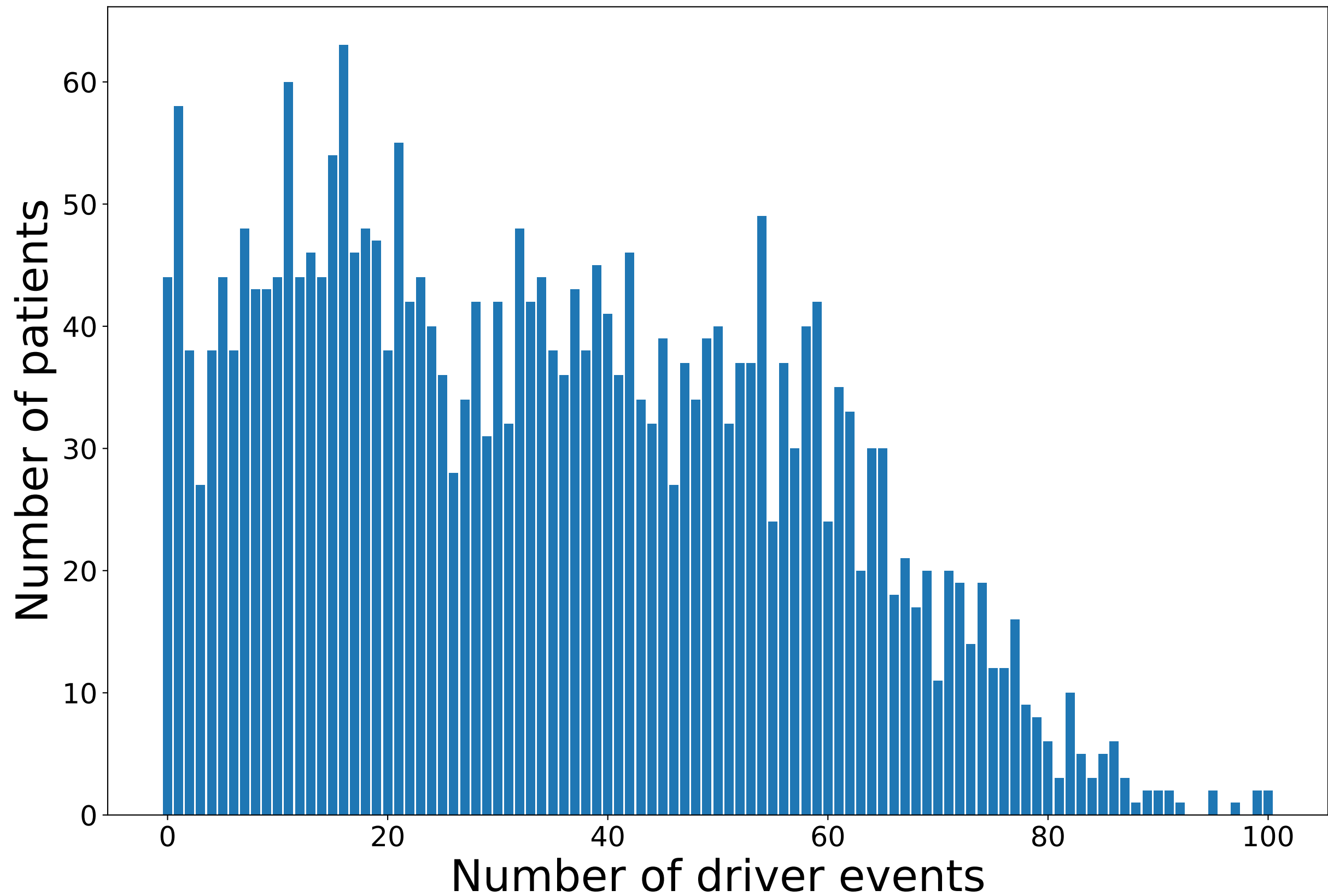

Supplement: S2 Files — (ZIP) [file pgen.1009996.s002.zip › PANCAN/patient distributions/2021_11_23_14_43_PANCAN_MALE.pdf]

# HNSC\_FEMALE

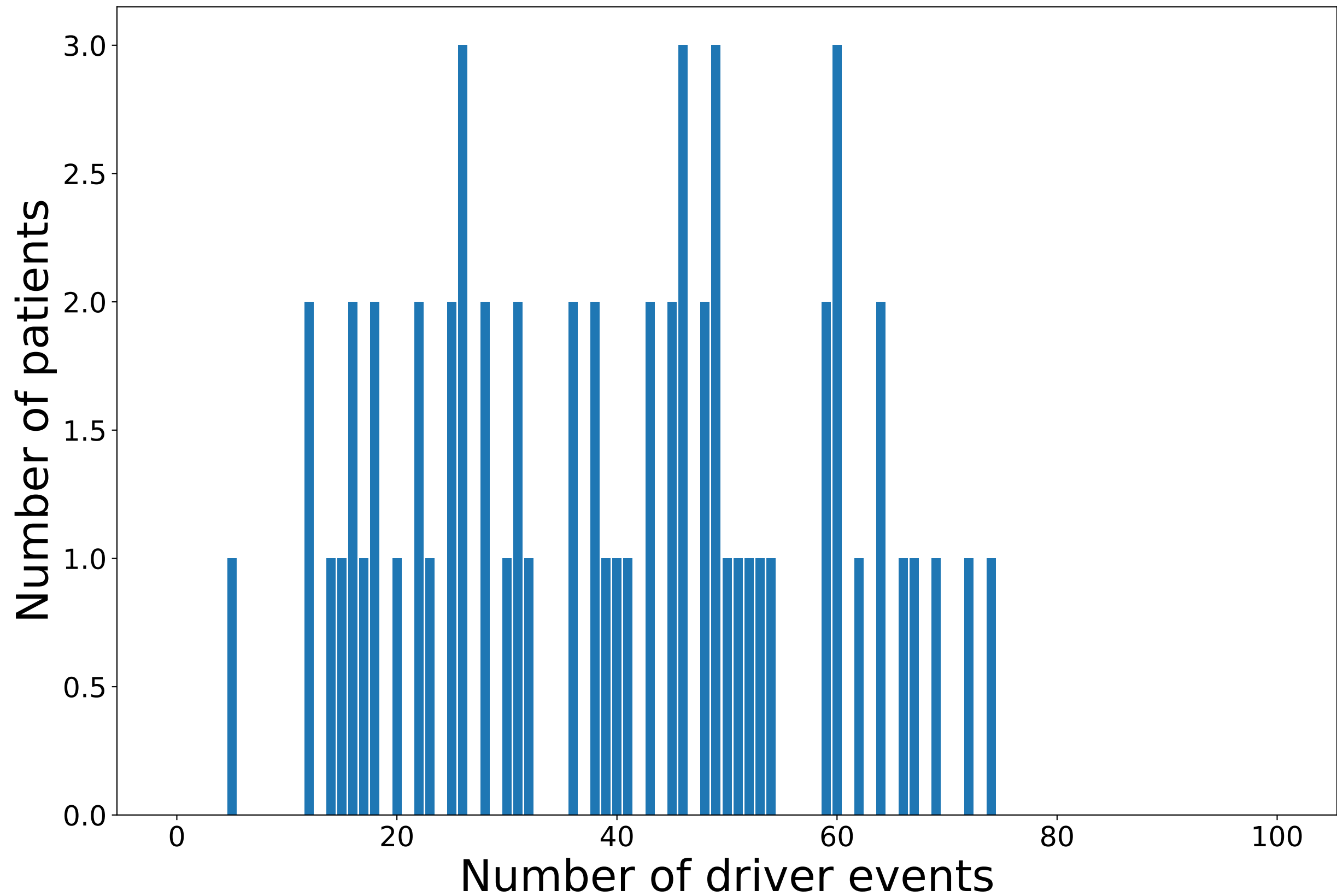

Supplement: S2 Files — (ZIP) [file pgen.1009996.s002.zip › PANCAN/patient distributions/2021_11_23_14_43_HNSC_FEMALE.pdf]

# OV\_FEMALE

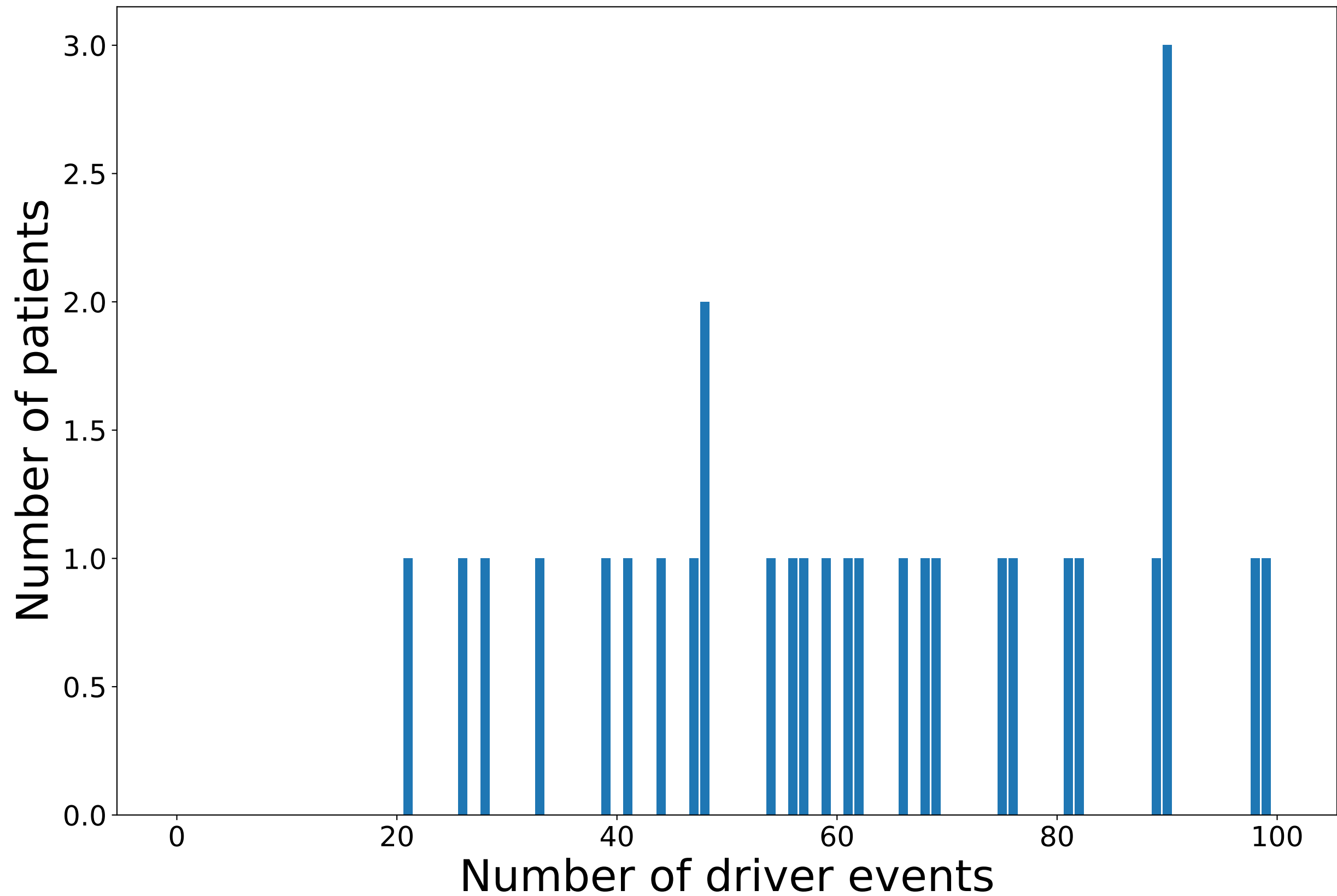

Supplement: S2 Files — (ZIP) [file pgen.1009996.s002.zip › PANCAN/patient distributions/2021_11_23_14_43_OV_FEMALE.pdf]

# TGCT

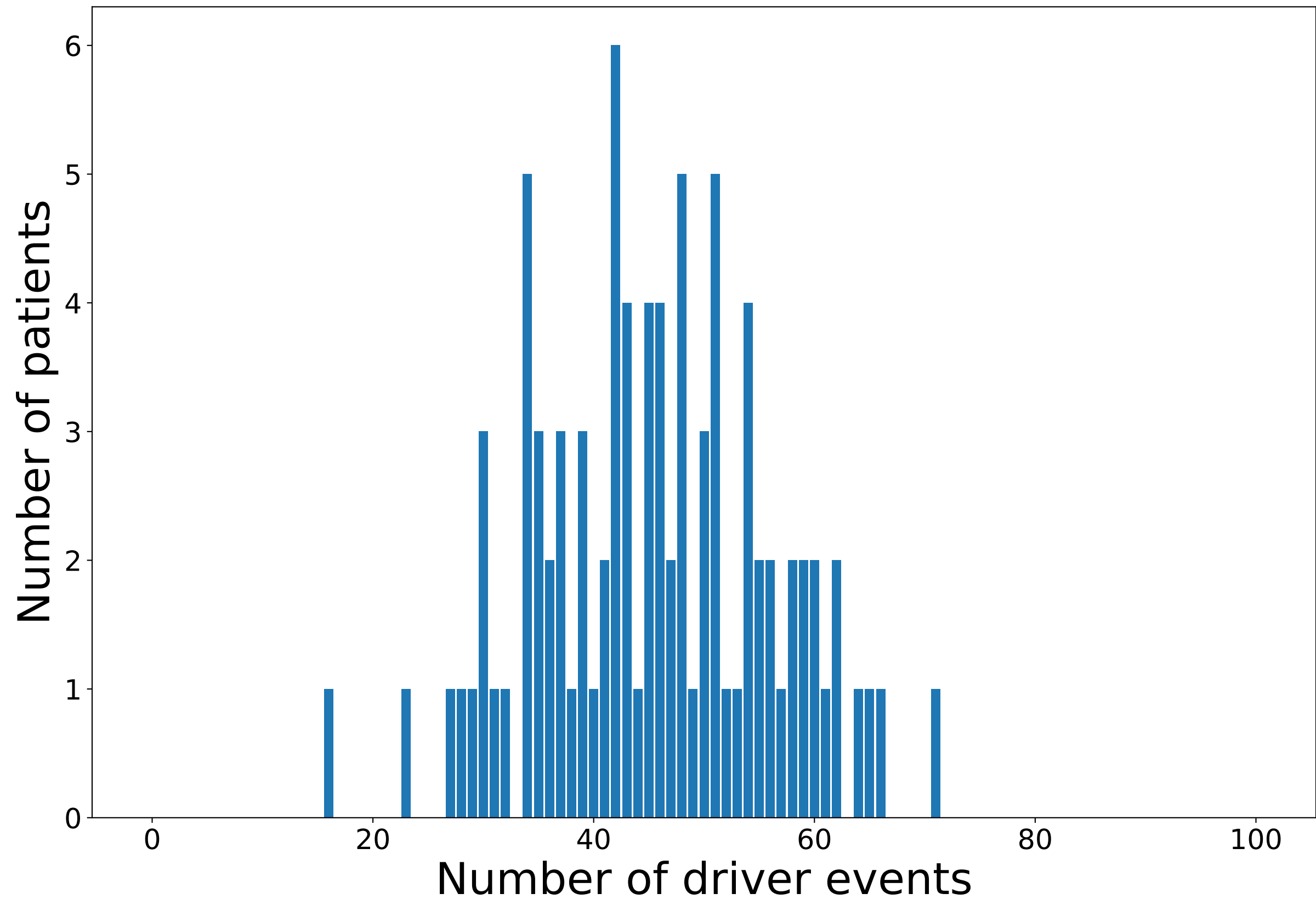

Supplement: S2 Files — (ZIP) [file pgen.1009996.s002.zip › PANCAN/patient distributions/2021_11_23_14_43_TGCT.pdf]

# CESC

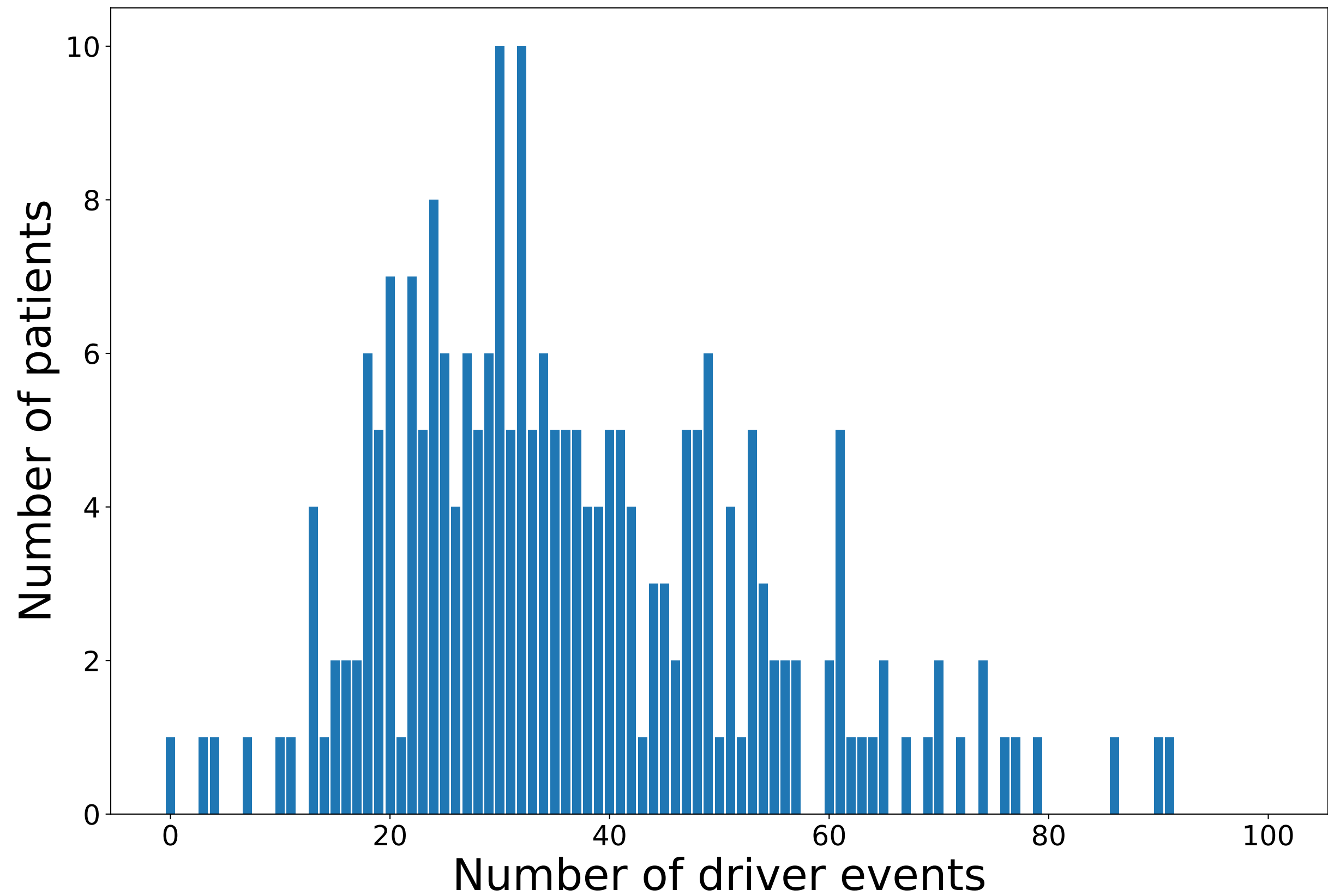

Supplement: S2 Files — (ZIP) [file pgen.1009996.s002.zip › PANCAN/patient distributions/2021_11_23_14_43_CESC.pdf]

# GBM\_FEMALE

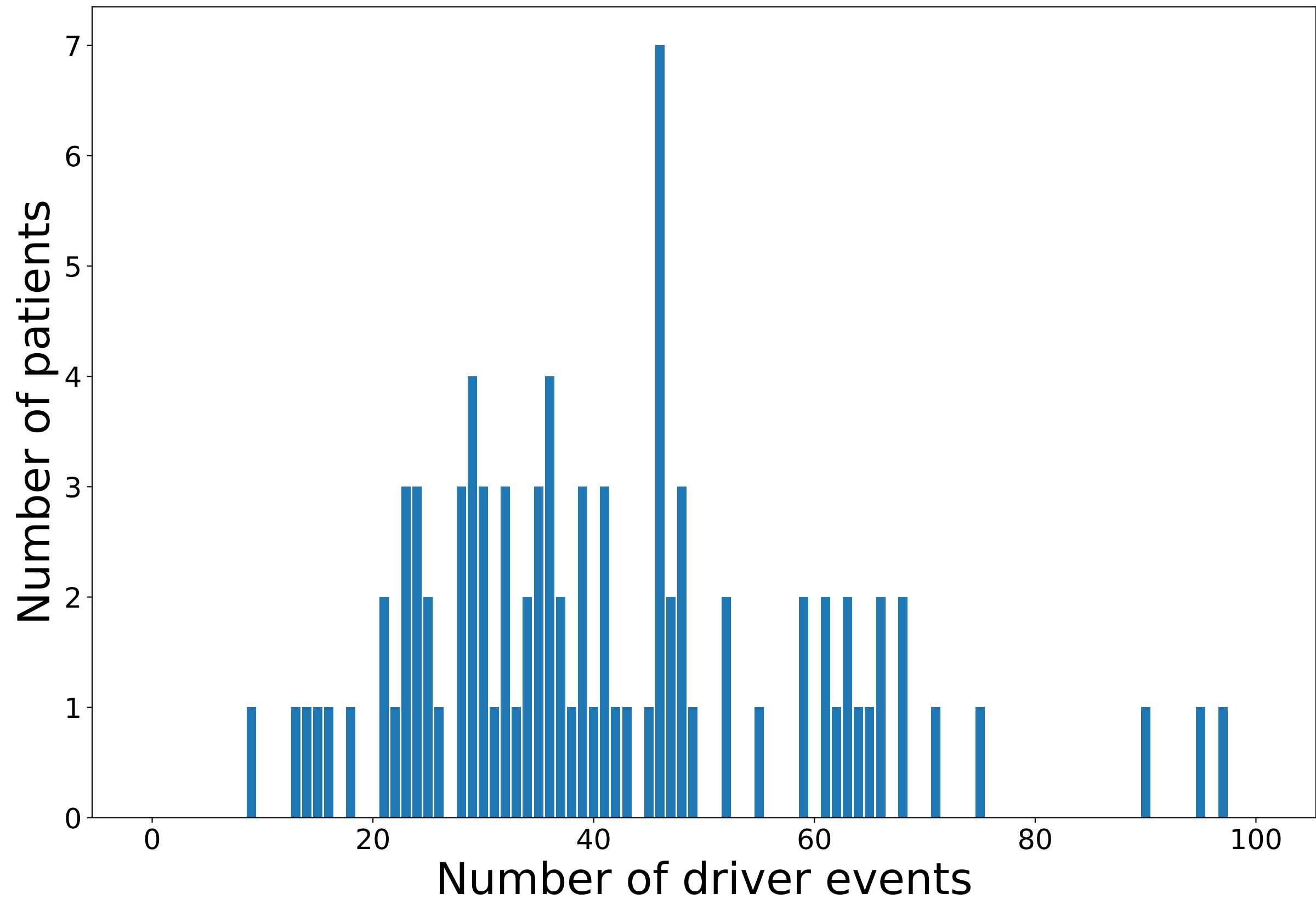

Supplement: S2 Files — (ZIP) [file pgen.1009996.s002.zip › PANCAN/patient distributions/2021_11_23_14_43_GBM_FEMALE.pdf]

# LGG\_FEMALE

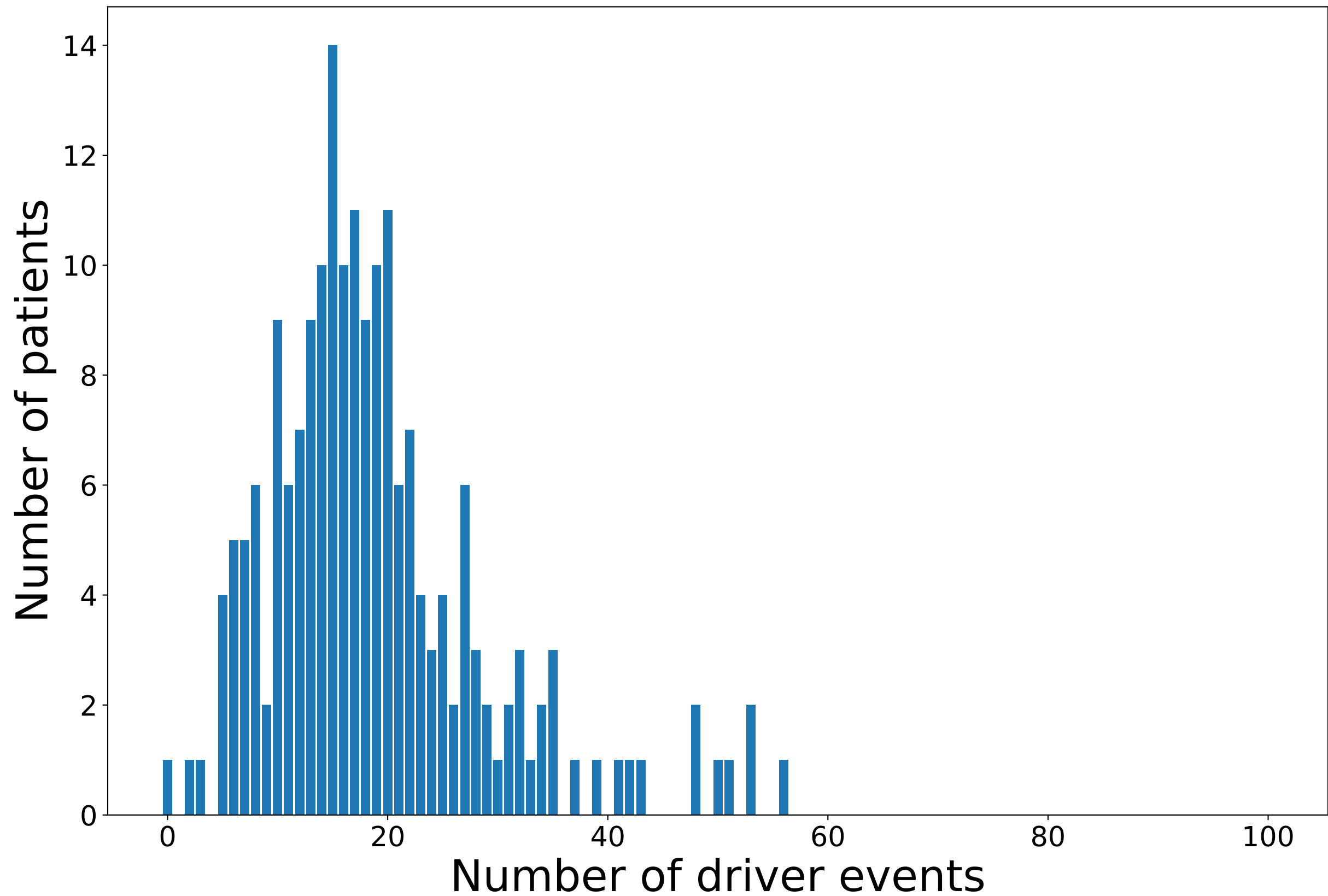

Supplement: S2 Files — (ZIP) [file pgen.1009996.s002.zip › PANCAN/patient distributions/2021_11_23_14_43_LGG_FEMALE.pdf]

# ESCA\_MALE

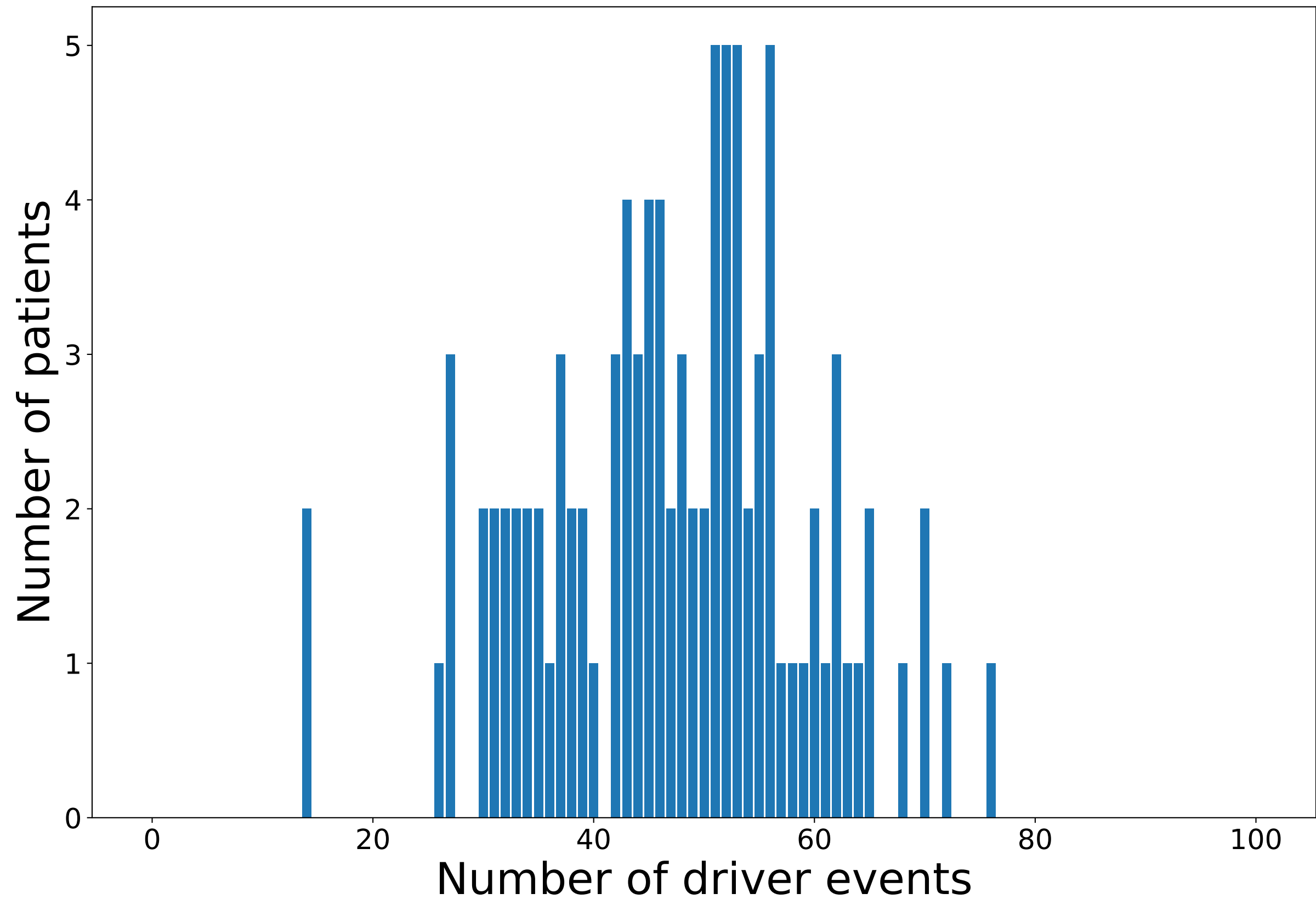

Supplement: S2 Files — (ZIP) [file pgen.1009996.s002.zip › PANCAN/patient distributions/2021_11_23_14_43_ESCA_MALE.pdf]

# GBM

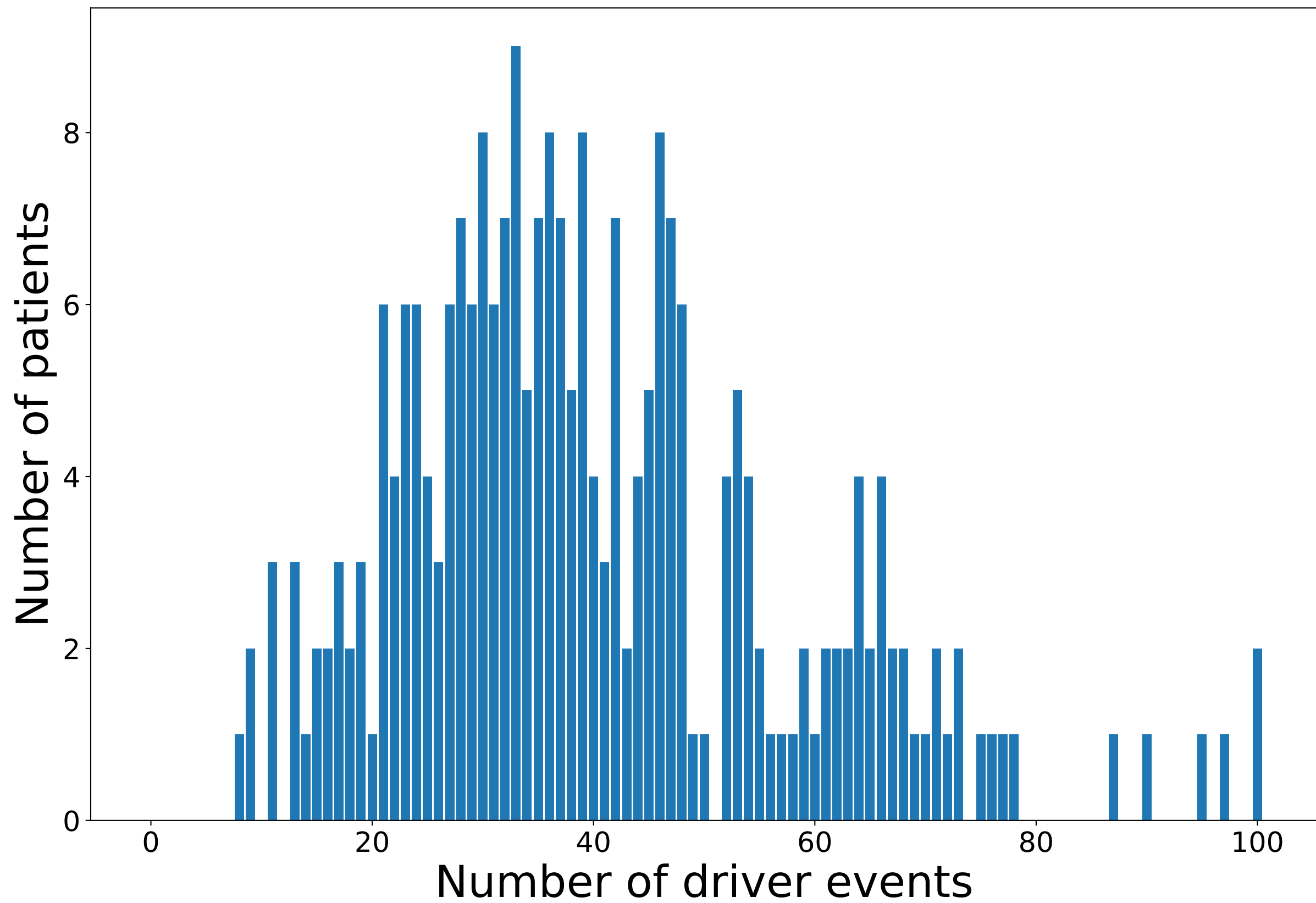

Supplement: S2 Files — (ZIP) [file pgen.1009996.s002.zip › PANCAN/patient distributions/2021_11_23_14_43_GBM.pdf]

# LIHC\_MALE

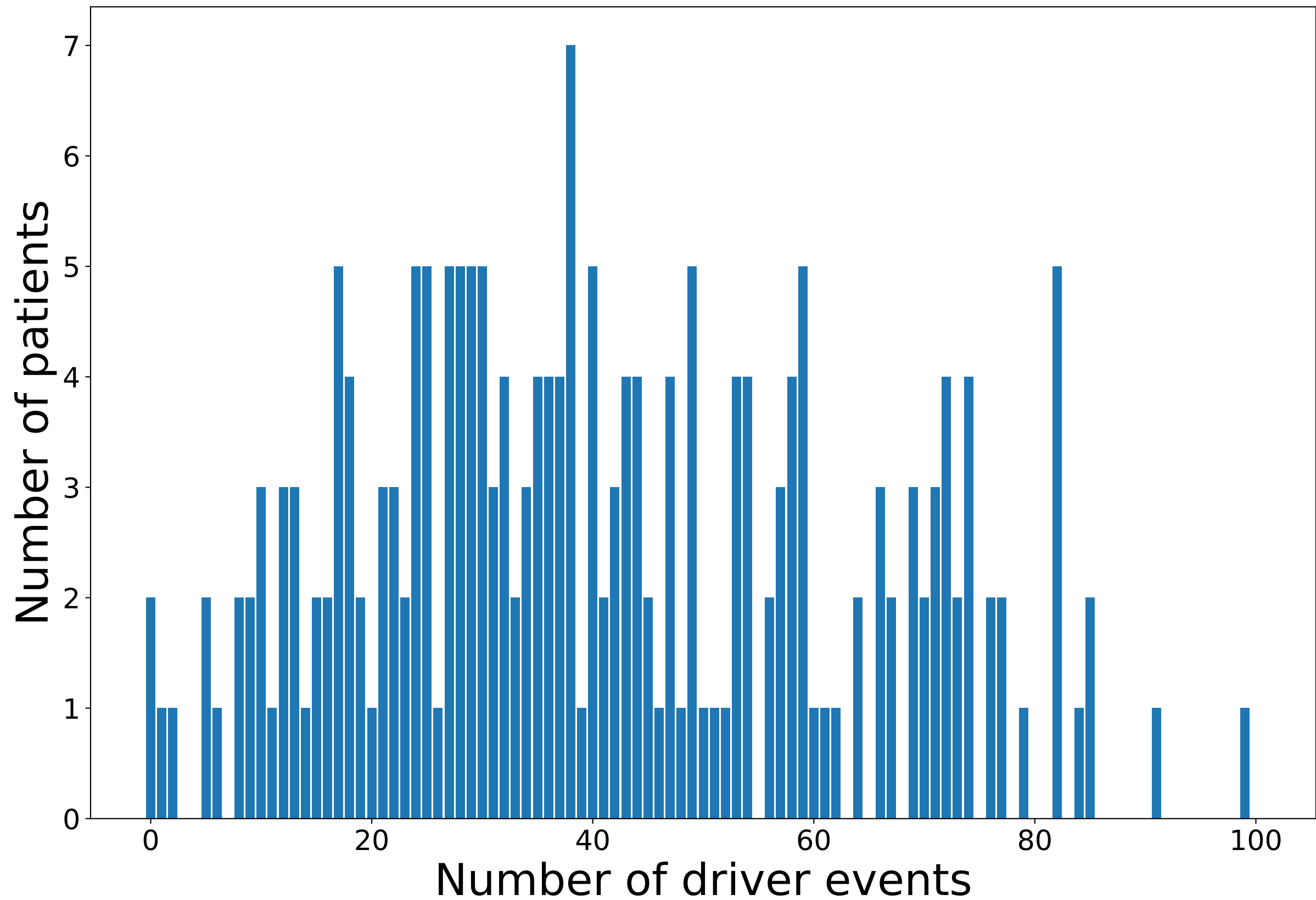

Supplement: S2 Files — (ZIP) [file pgen.1009996.s002.zip › PANCAN/patient distributions/2021_11_23_14_43_LIHC_MALE.pdf]

# SKCM\_FEMALE

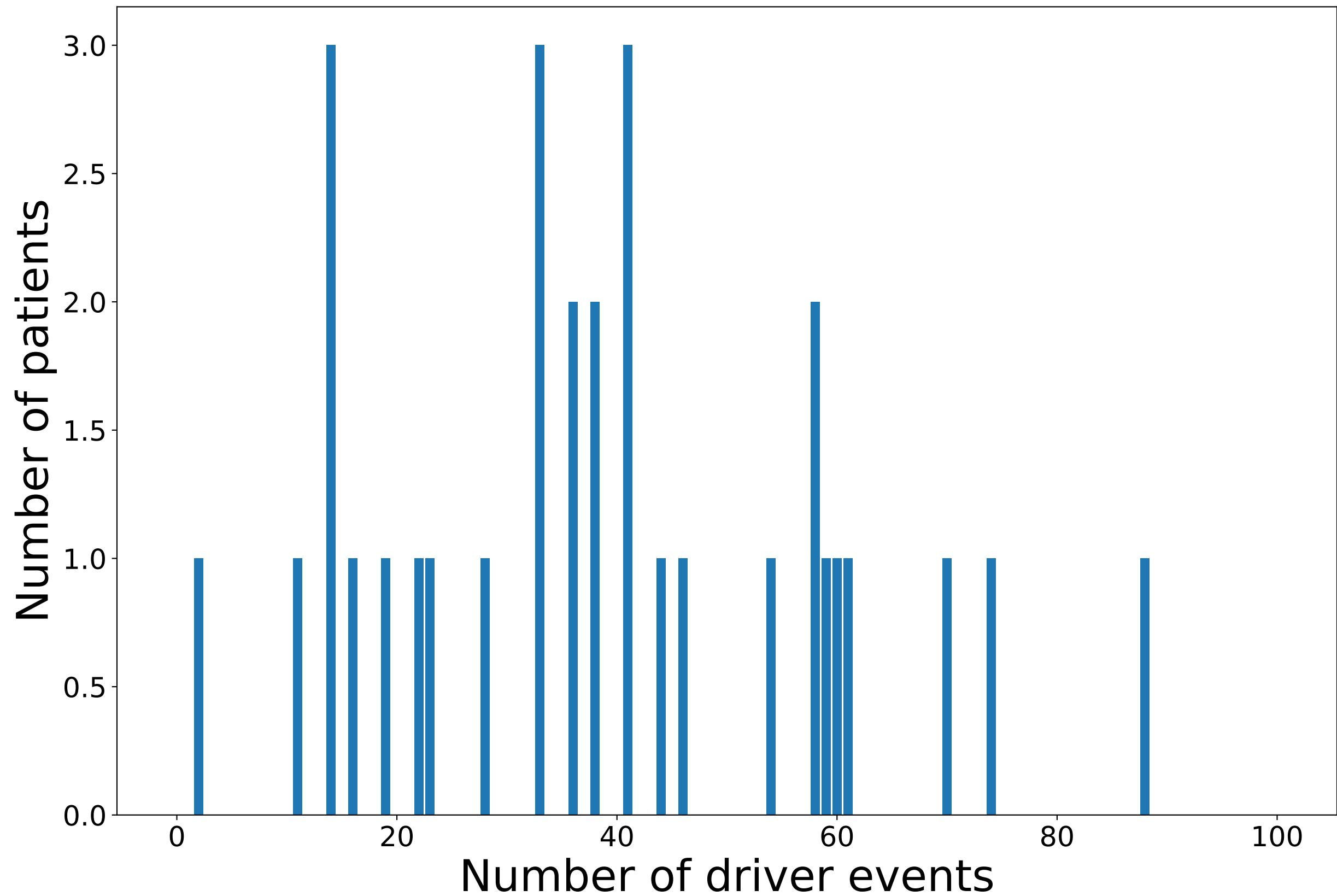

Supplement: S2 Files — (ZIP) [file pgen.1009996.s002.zip › PANCAN/patient distributions/2021_11_23_14_43_SKCM_FEMALE.pdf]

# STAD\_MALE

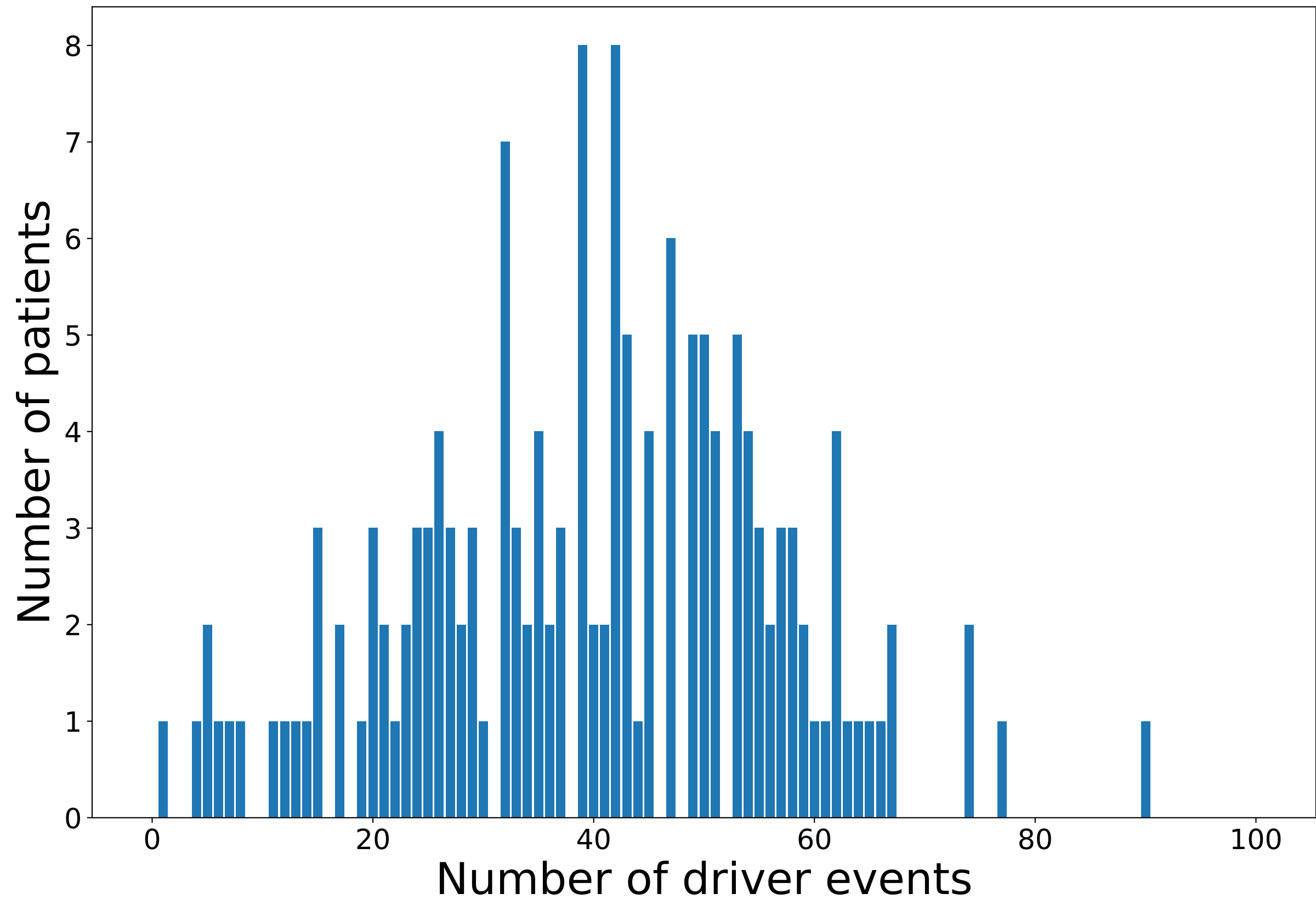

Supplement: S2 Files — (ZIP) [file pgen.1009996.s002.zip › PANCAN/patient distributions/2021_11_23_14_43_STAD_MALE.pdf]

# PAAD\_FEMALE

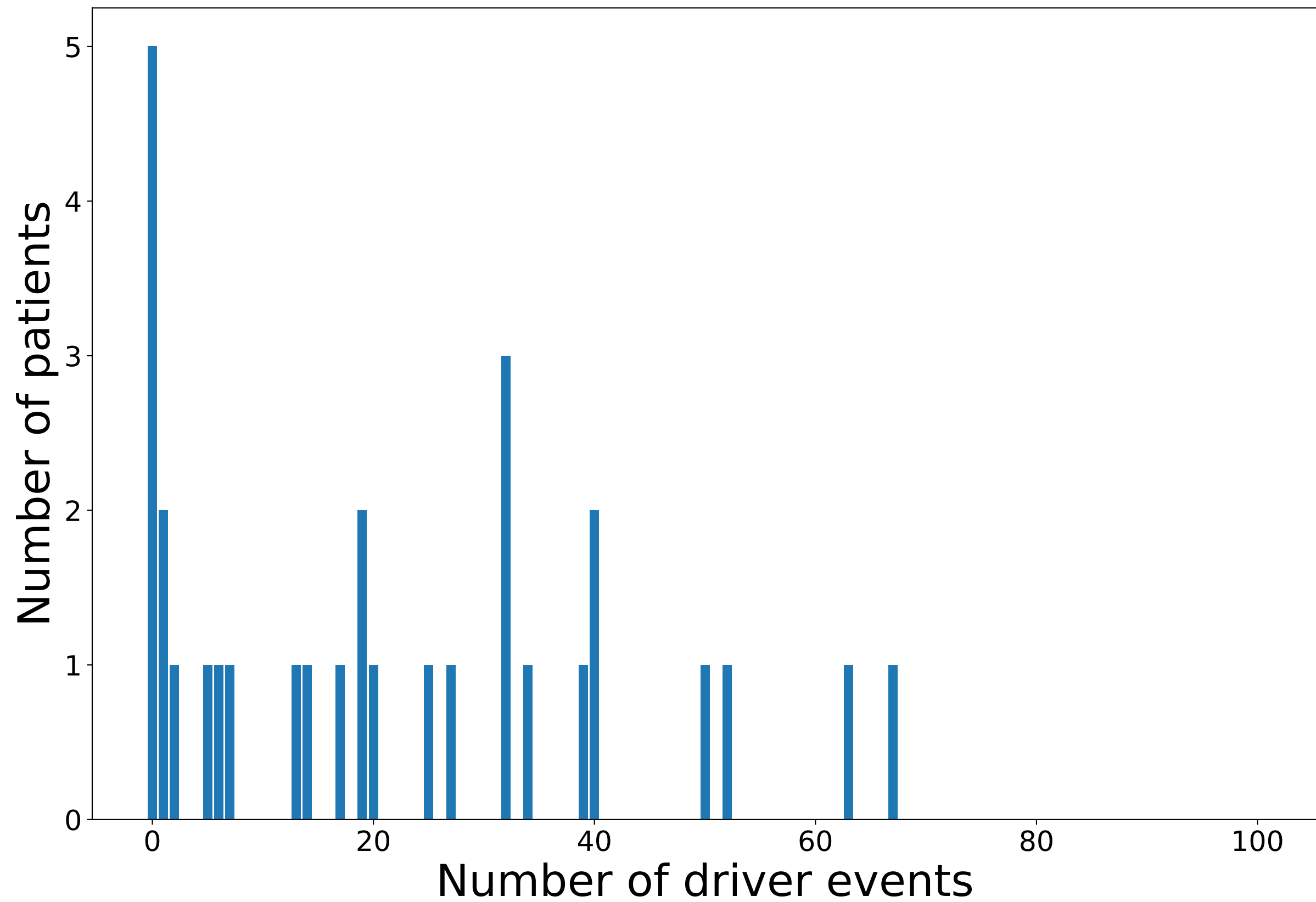

Supplement: S2 Files — (ZIP) [file pgen.1009996.s002.zip › PANCAN/patient distributions/2021_11_23_14_43_PAAD_FEMALE.pdf]

# THYM\_MALE

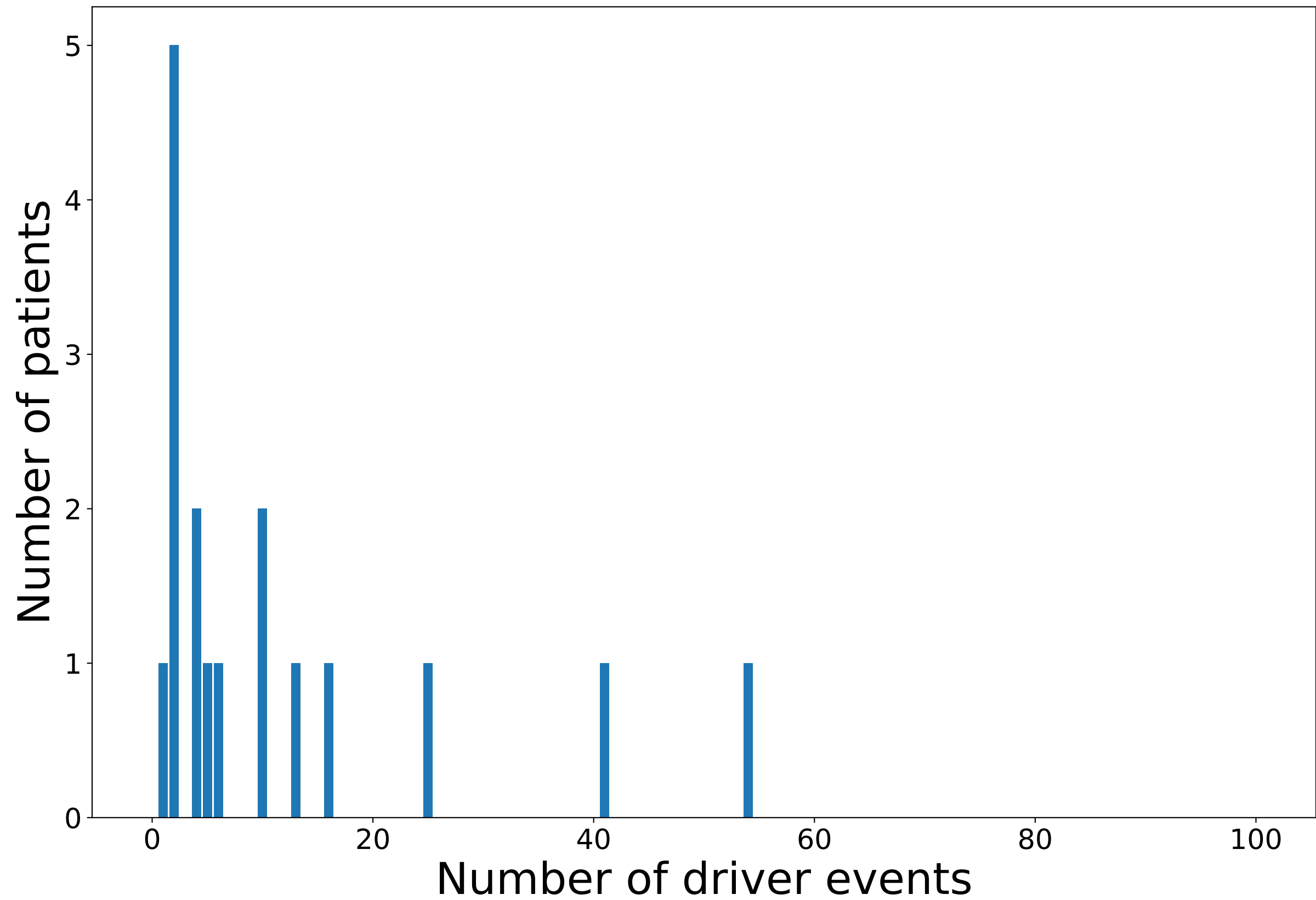

Supplement: S2 Files — (ZIP) [file pgen.1009996.s002.zip › PANCAN/patient distributions/2021_11_23_14_43_THYM_MALE.pdf]

# KIRP\_MALE

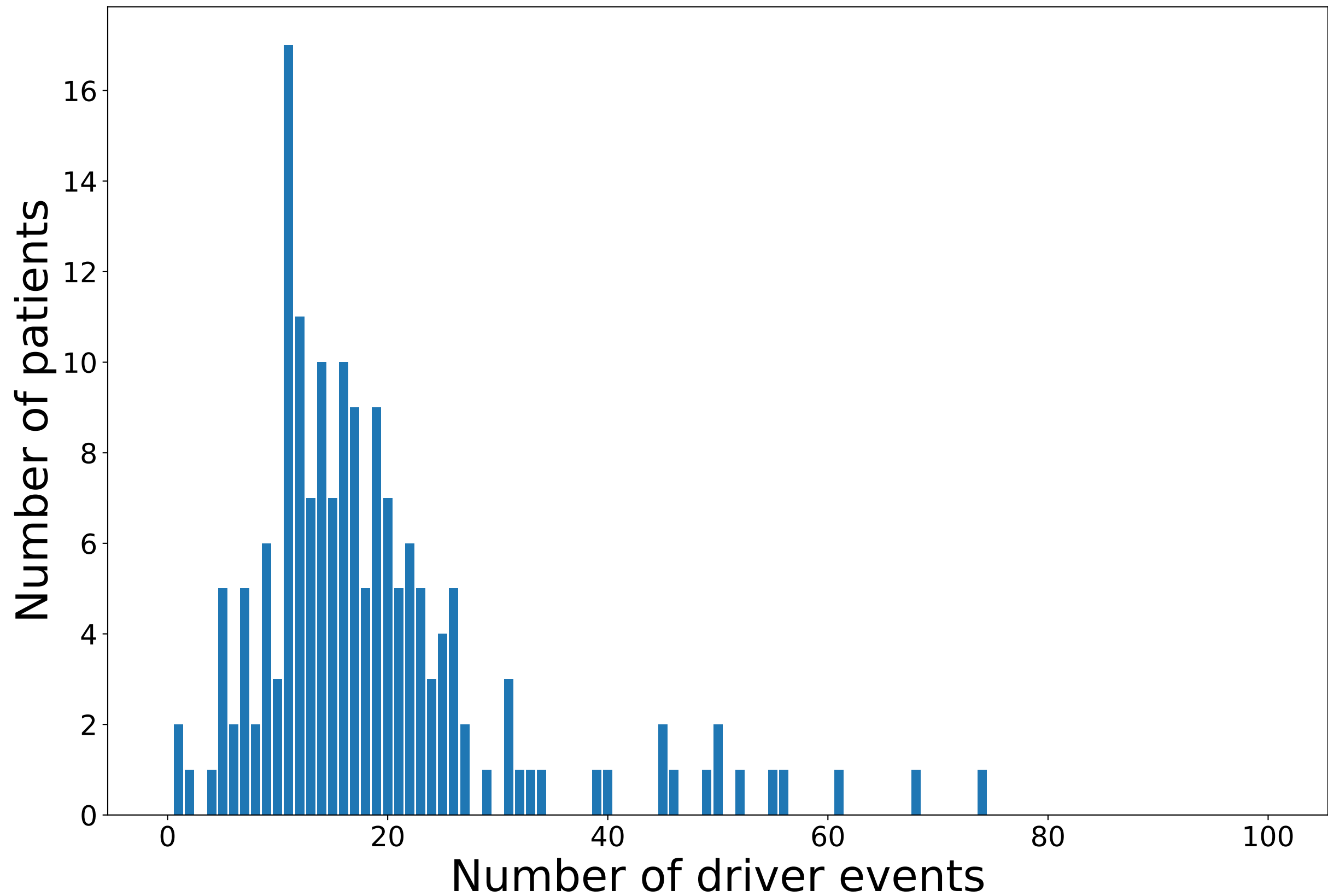

Supplement: S2 Files — (ZIP) [file pgen.1009996.s002.zip › PANCAN/patient distributions/2021_11_23_14_43_KIRP_MALE.pdf]

# PCPG

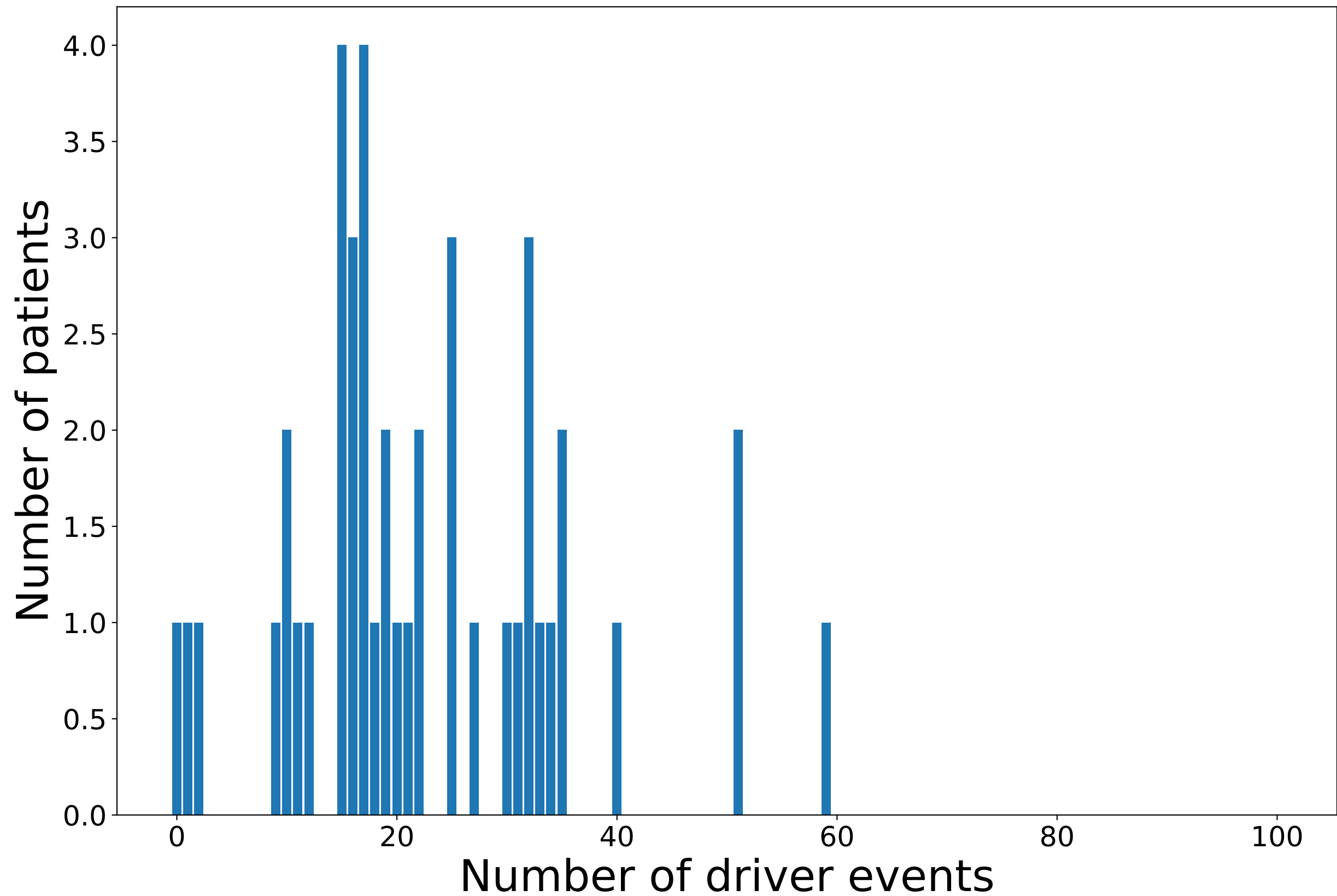

Supplement: S2 Files — (ZIP) [file pgen.1009996.s002.zip › PANCAN/patient distributions/2021_11_23_14_43_PCPG.pdf]

# LUSC

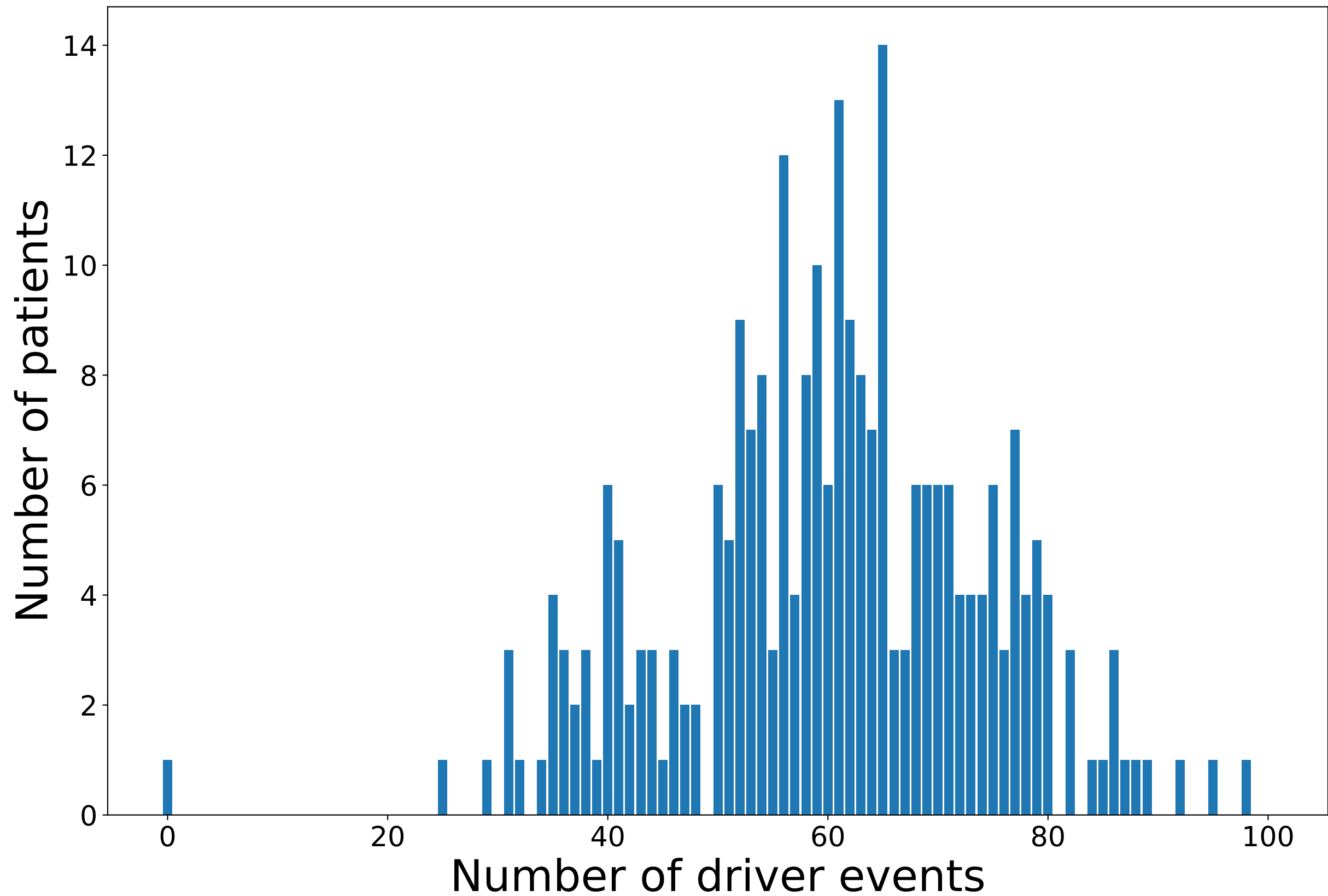

Supplement: S2 Files — (ZIP) [file pgen.1009996.s002.zip › PANCAN/patient distributions/2021_11_23_14_43_LUSC.pdf]

# HNSC\_MALE

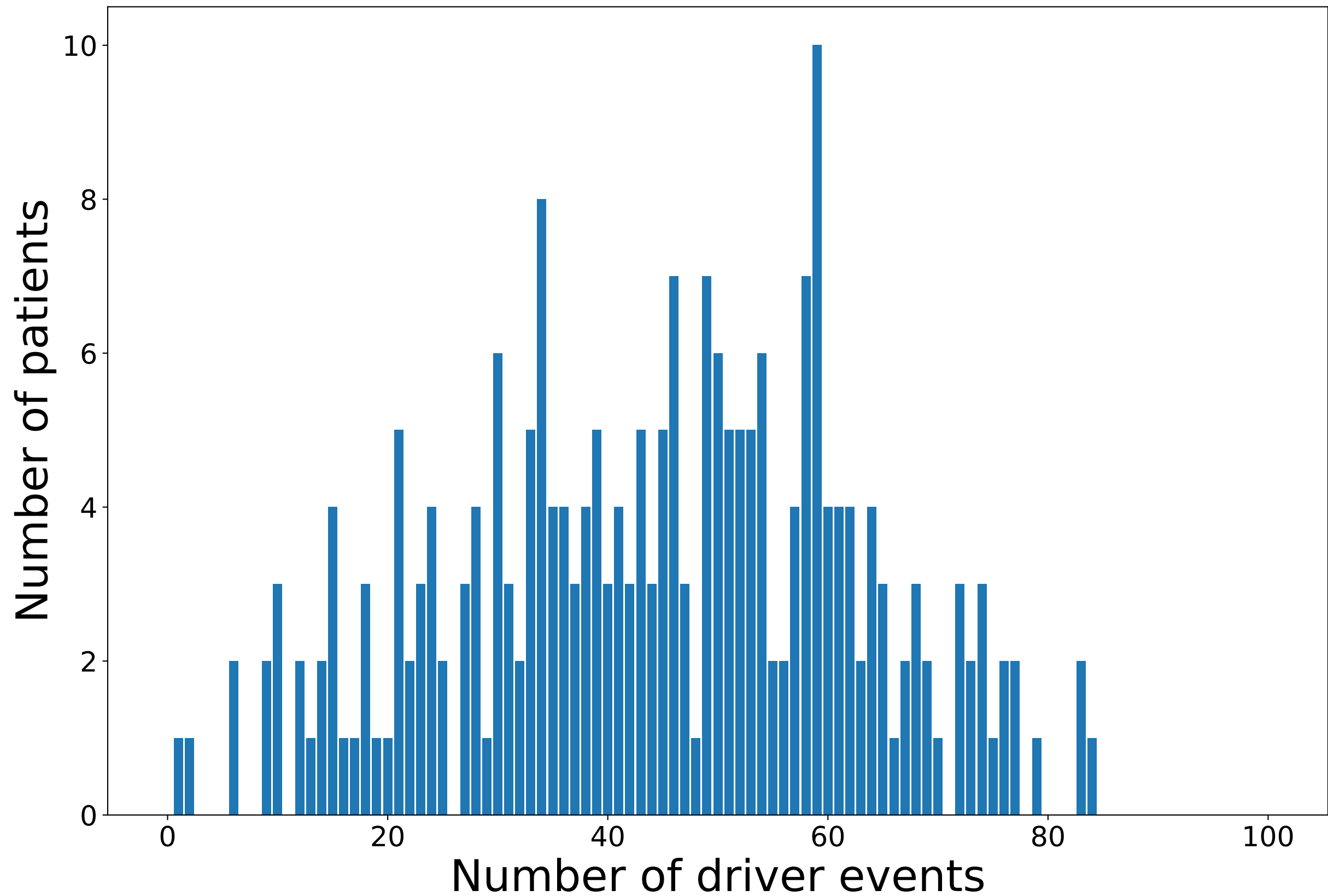

Supplement: S2 Files — (ZIP) [file pgen.1009996.s002.zip › PANCAN/patient distributions/2021_11_23_14_43_HNSC_MALE.pdf]

# READ\_FEMALE

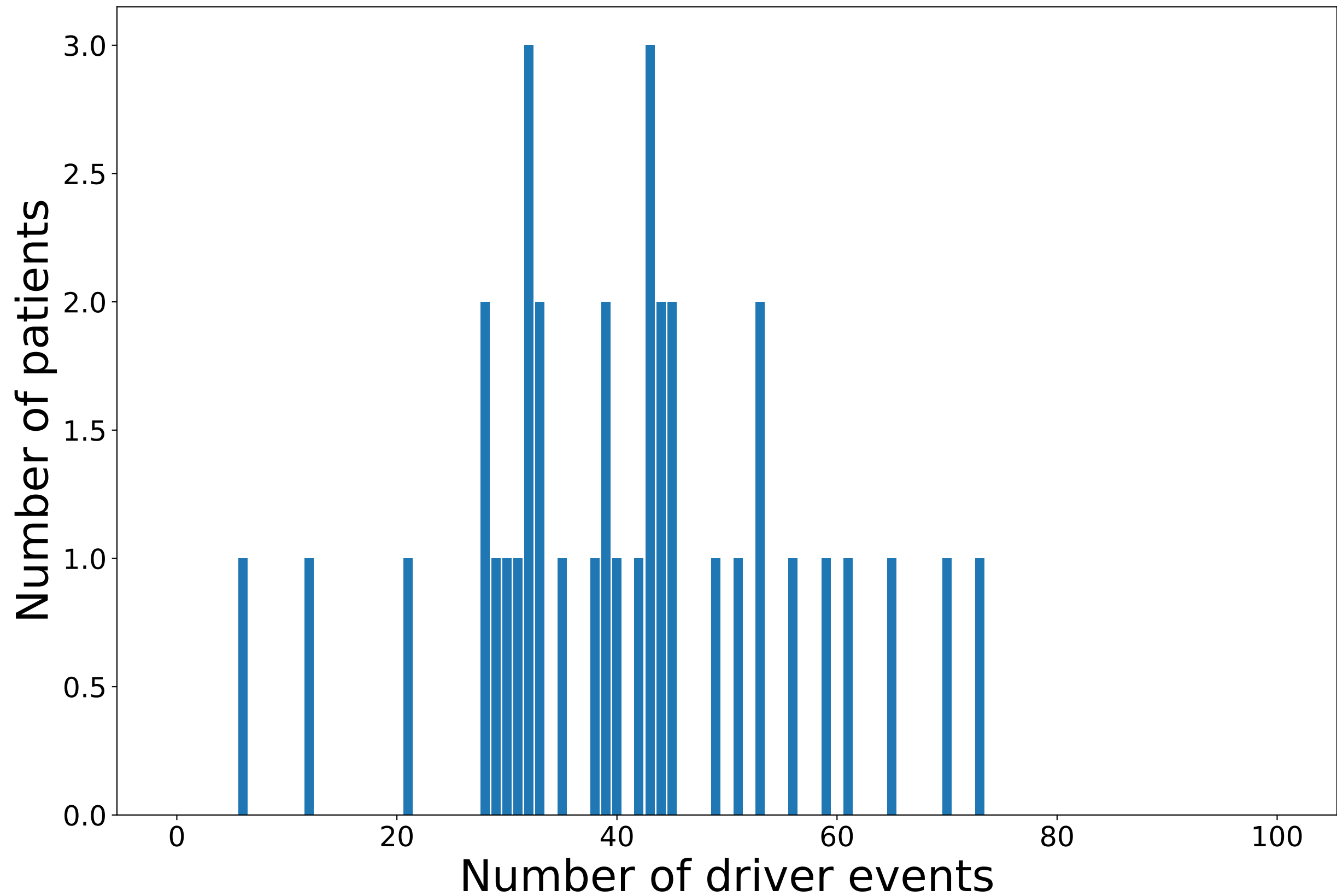

Supplement: S2 Files — (ZIP) [file pgen.1009996.s002.zip › PANCAN/patient distributions/2021_11_23_14_43_READ_FEMALE.pdf]

# LUAD\_FEMALE

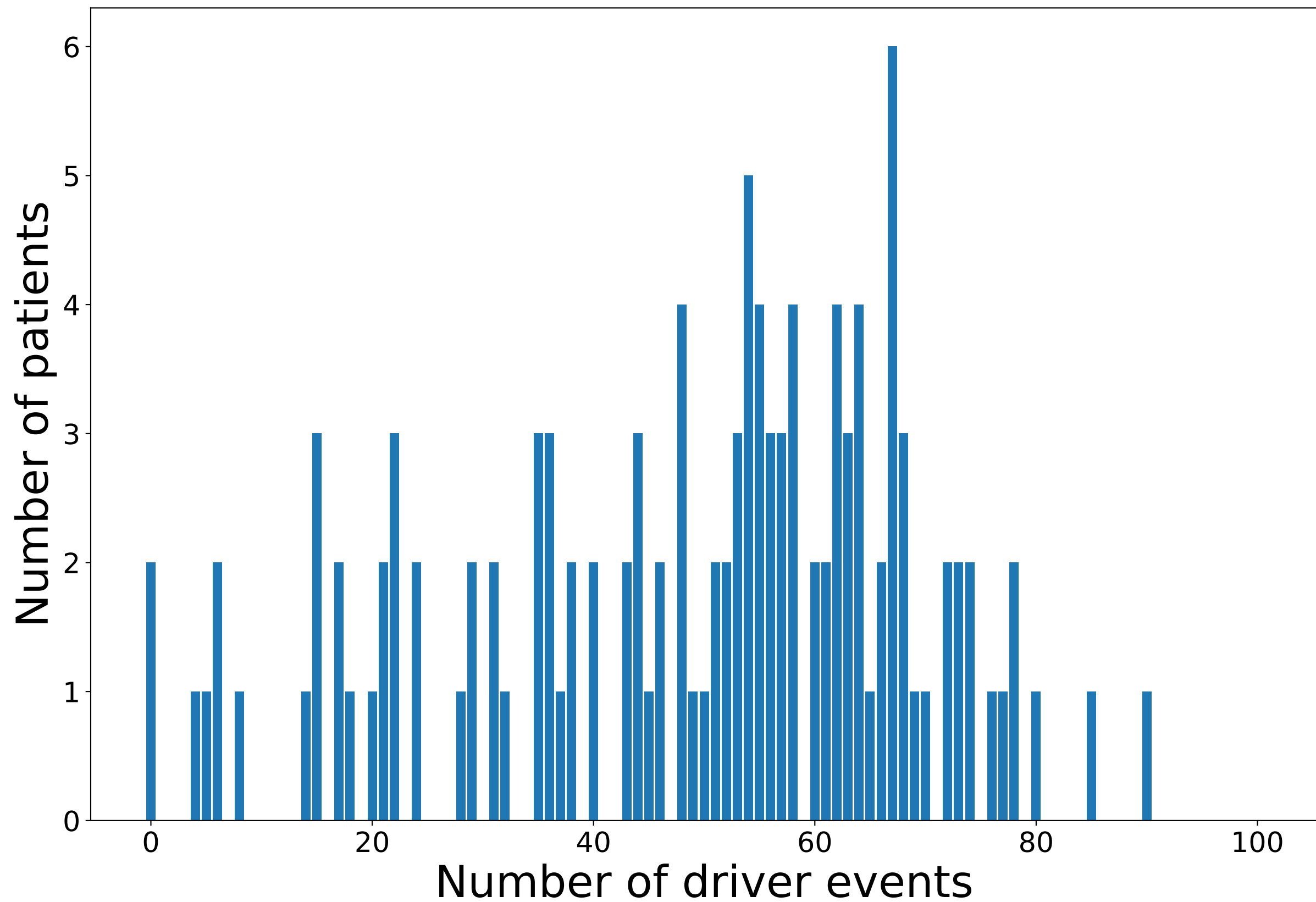

Supplement: S2 Files — (ZIP) [file pgen.1009996.s002.zip › PANCAN/patient distributions/2021_11_23_14_43_LUAD_FEMALE.pdf]

# BLCA\_MALE

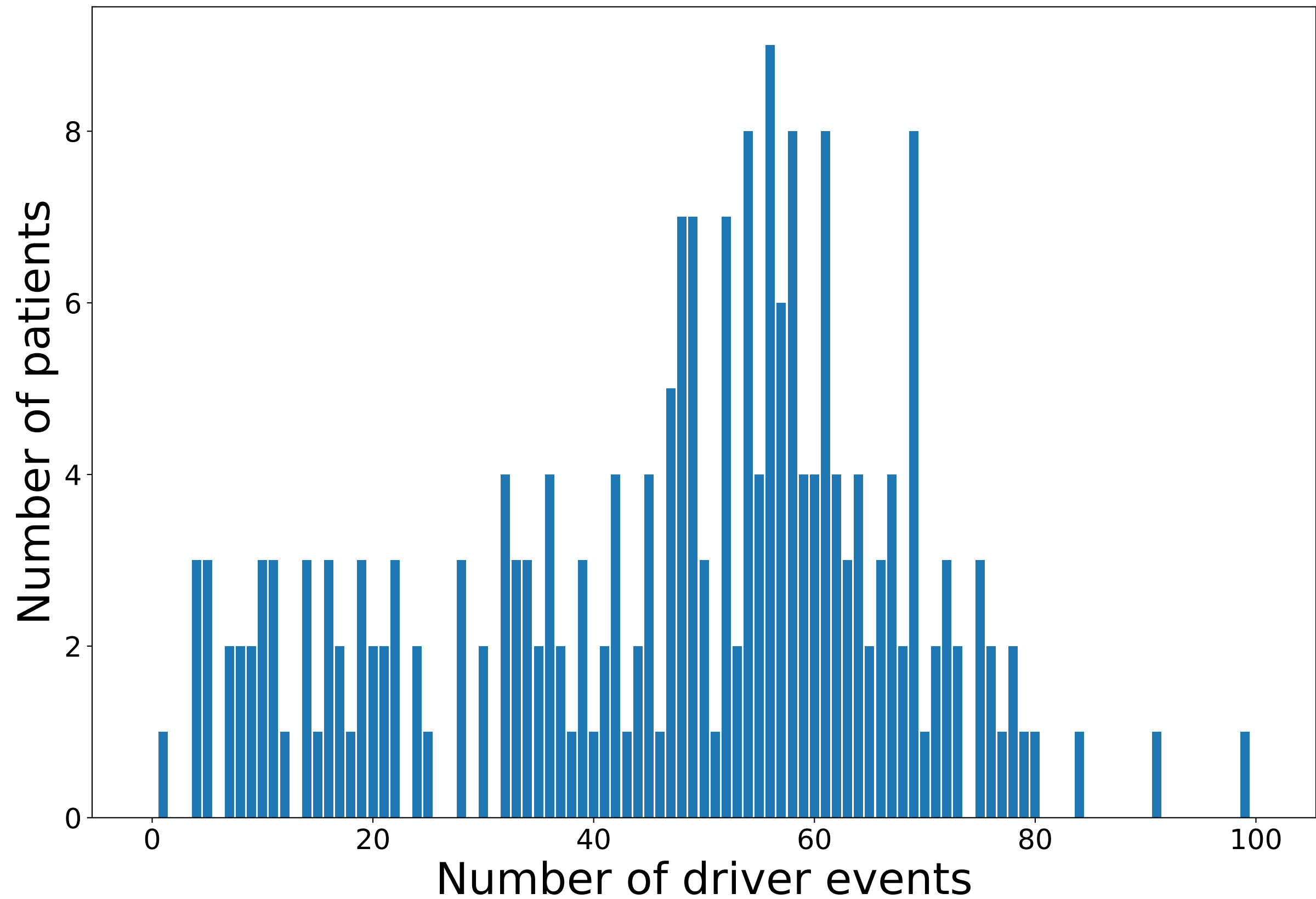

Supplement: S2 Files — (ZIP) [file pgen.1009996.s002.zip › PANCAN/patient distributions/2021_11_23_14_43_BLCA_MALE.pdf]

# SARC\_FEMALE

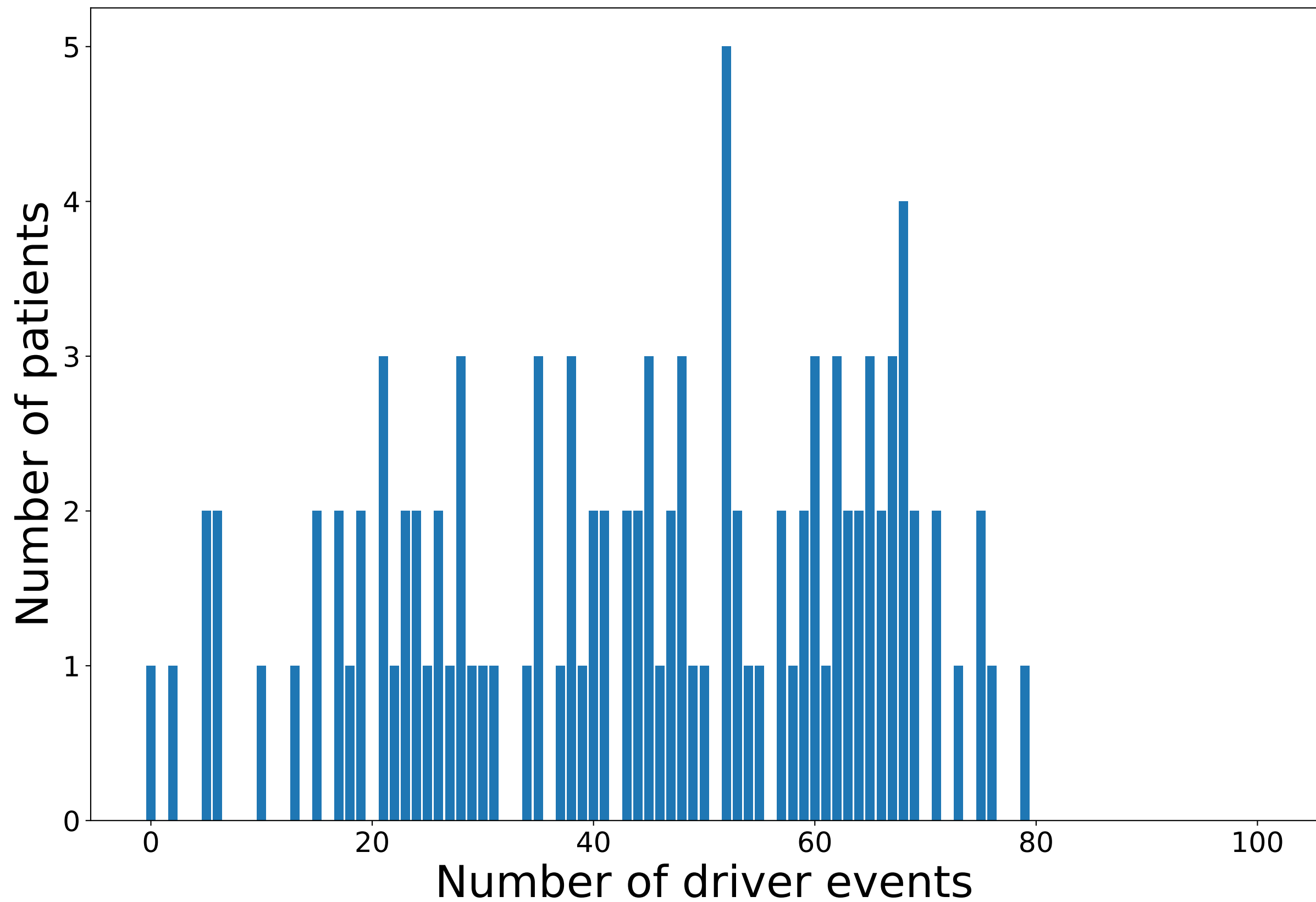

Supplement: S2 Files — (ZIP) [file pgen.1009996.s002.zip › PANCAN/patient distributions/2021_11_23_14_43_SARC_FEMALE.pdf]

# CHOL\_MALE

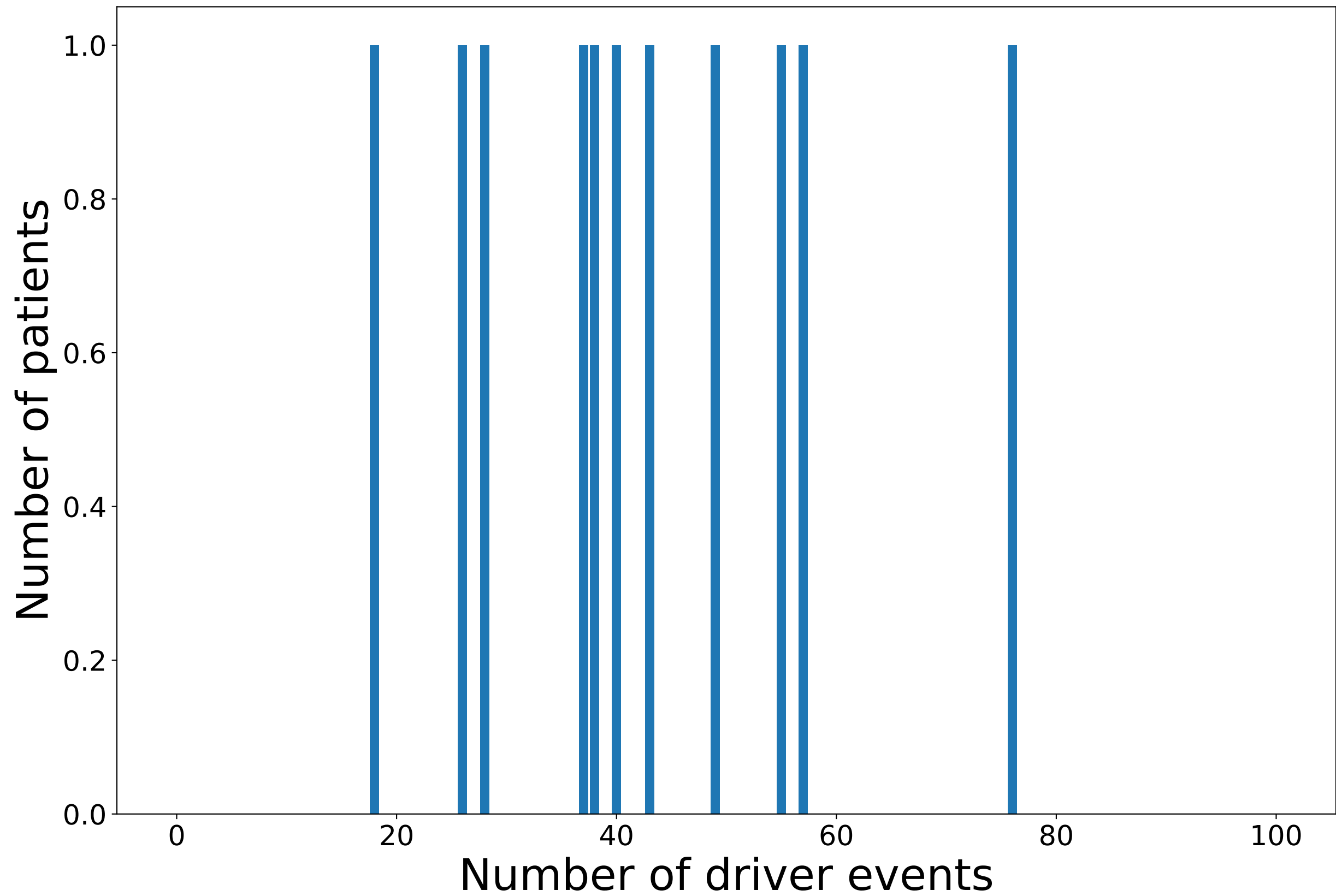

Supplement: S2 Files — (ZIP) [file pgen.1009996.s002.zip › PANCAN/patient distributions/2021_11_23_14_43_CHOL_MALE.pdf]

# COAD\_MALE

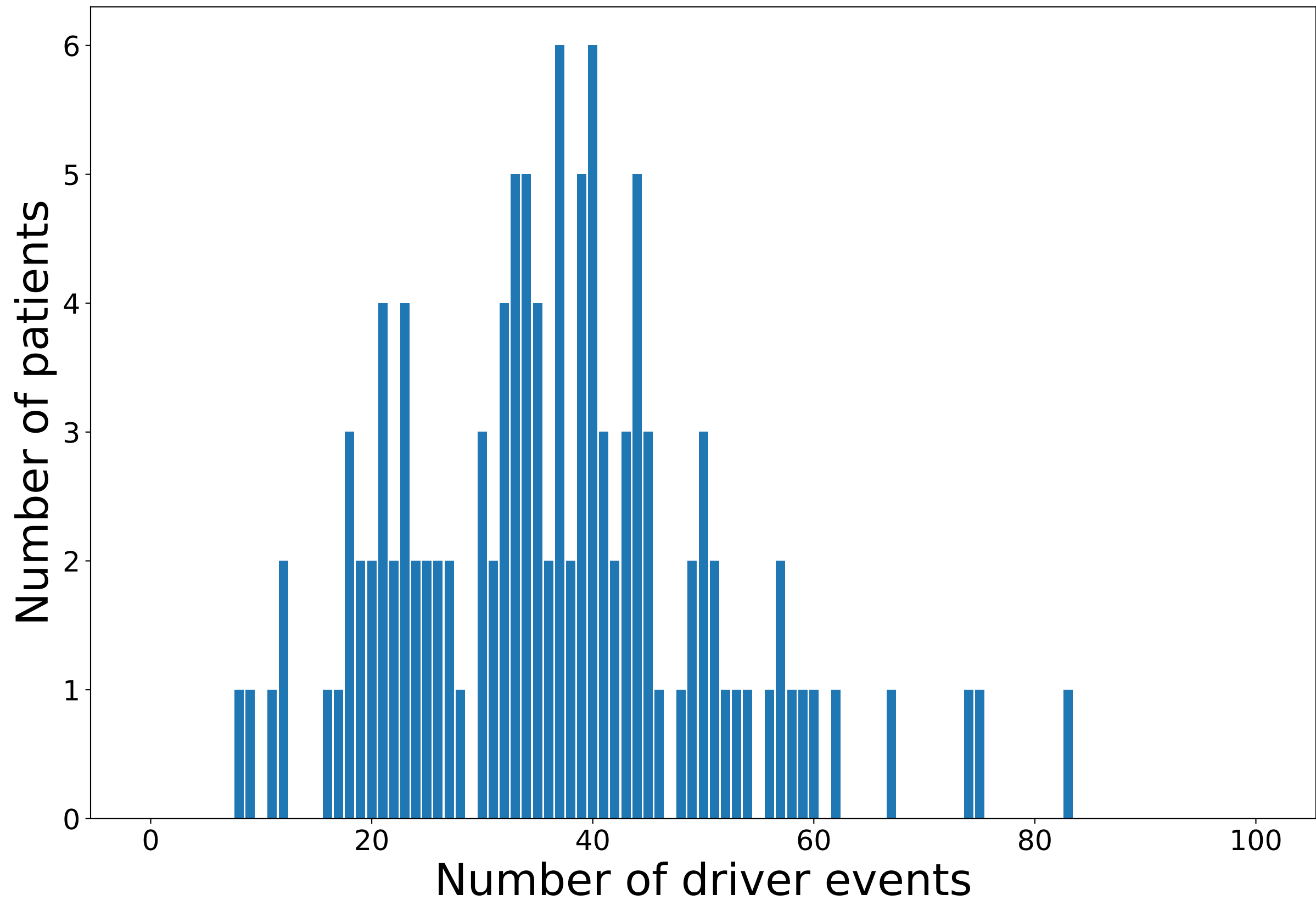

Supplement: S2 Files — (ZIP) [file pgen.1009996.s002.zip › PANCAN/patient distributions/2021_11_23_14_43_COAD_MALE.pdf]

# ESCA

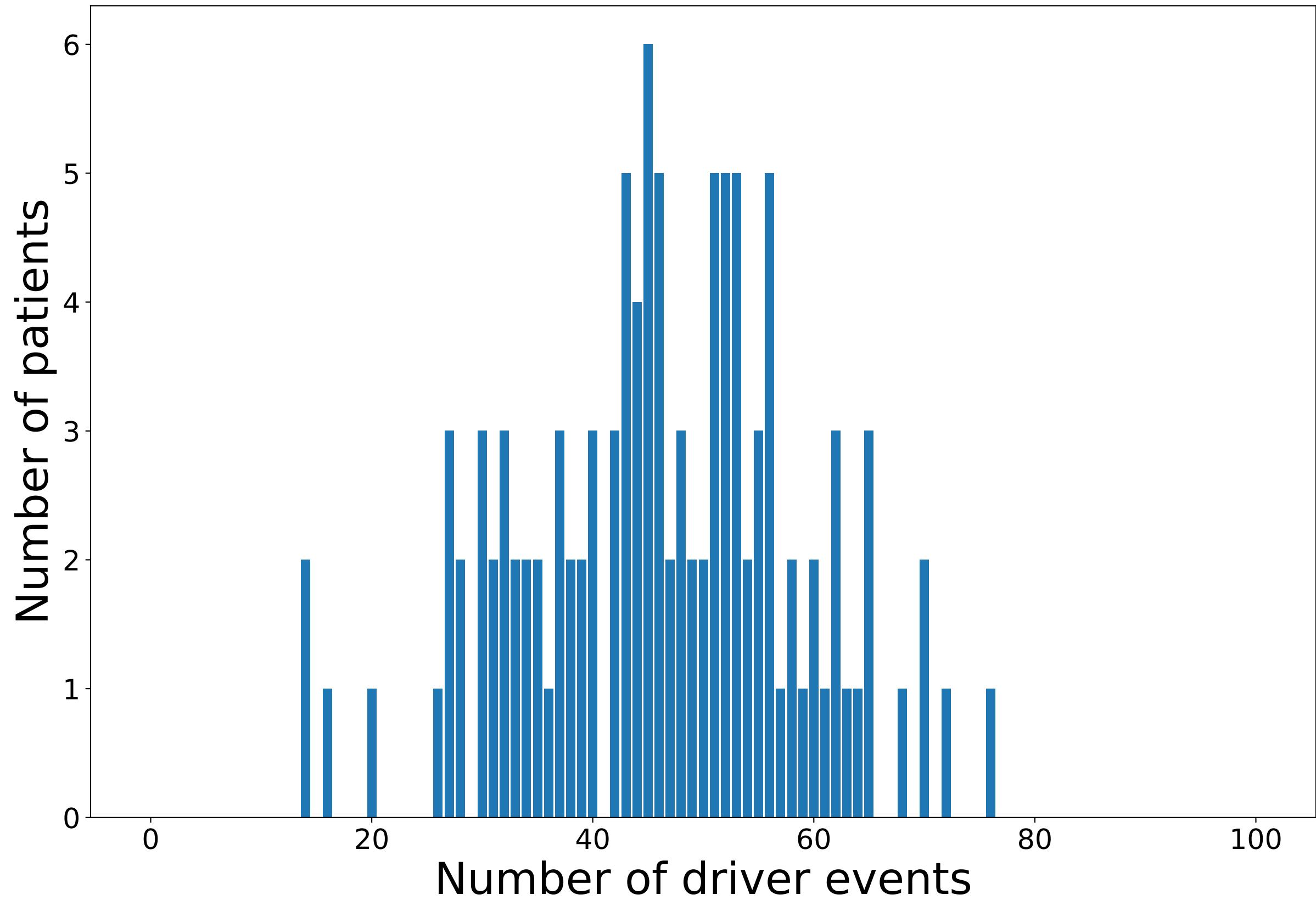

Supplement: S2 Files — (ZIP) [file pgen.1009996.s002.zip › PANCAN/patient distributions/2021_11_23_14_43_ESCA.pdf]

# COAD\_FEMALE

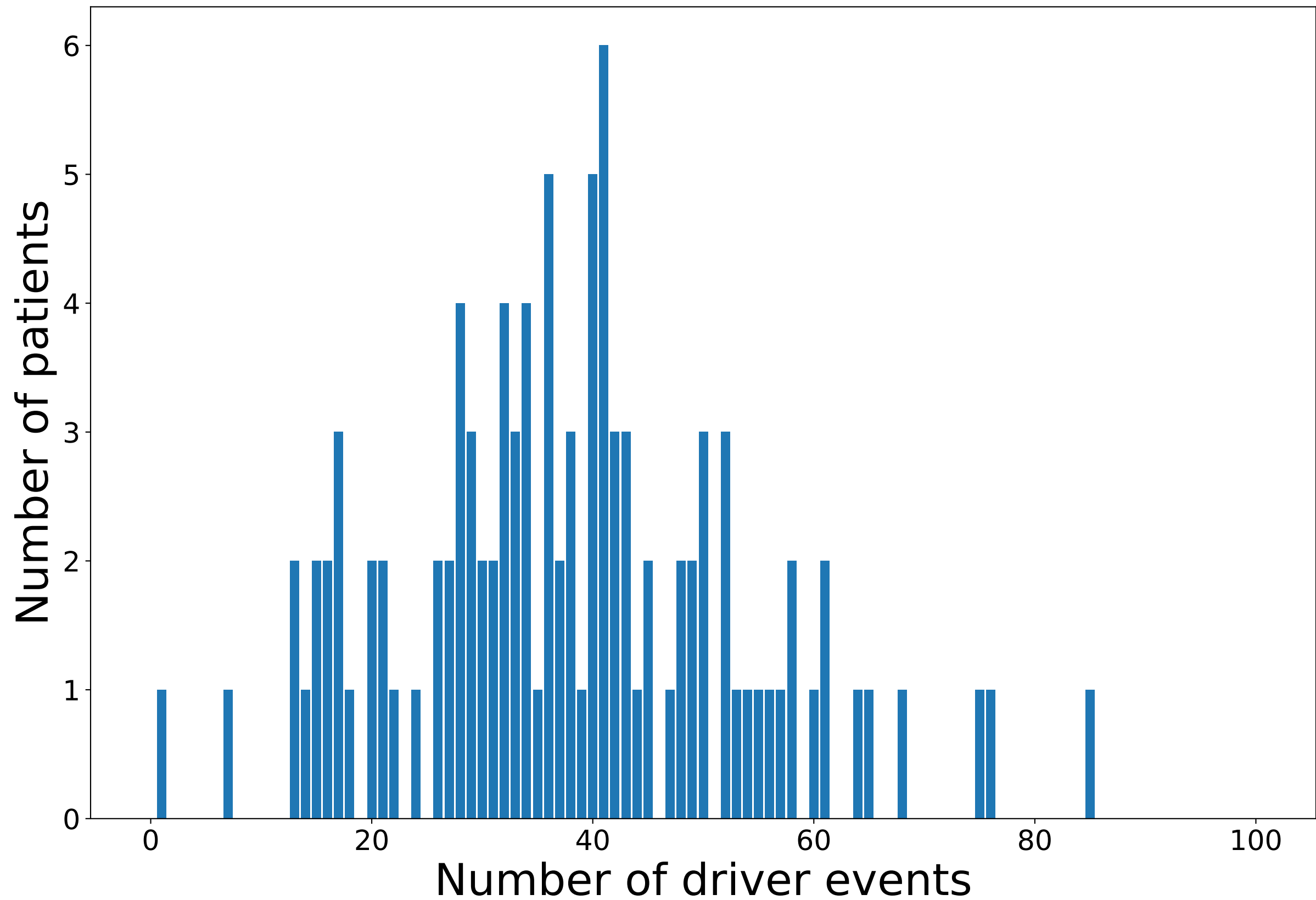

Supplement: S2 Files — (ZIP) [file pgen.1009996.s002.zip › PANCAN/patient distributions/2021_11_23_14_43_COAD_FEMALE.pdf]

# HNSC

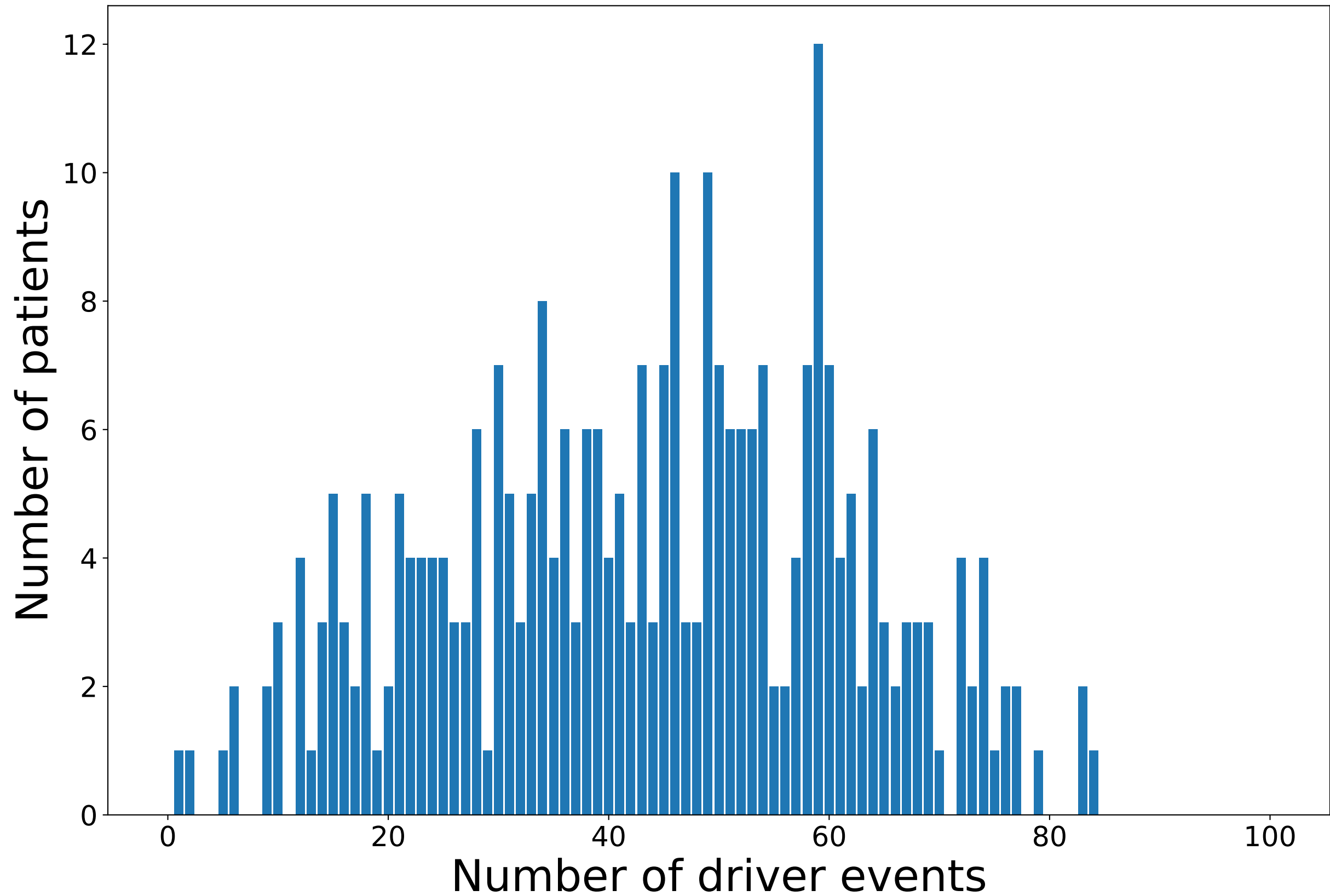

Supplement: S2 Files — (ZIP) [file pgen.1009996.s002.zip › PANCAN/patient distributions/2021_11_23_14_43_HNSC.pdf]

# UVM\_MALE

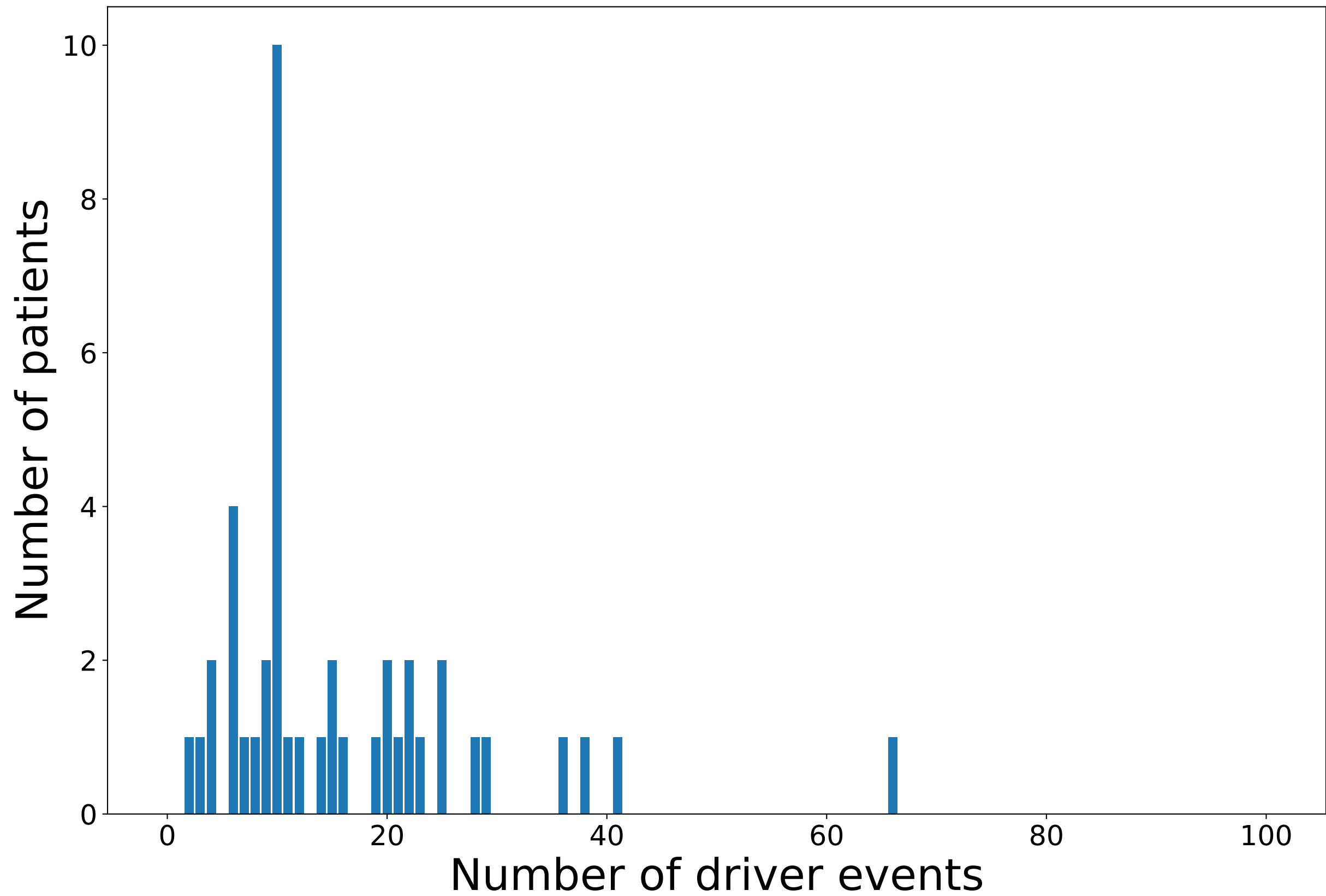

Supplement: S2 Files — (ZIP) [file pgen.1009996.s002.zip › PANCAN/patient distributions/2021_11_23_14_43_UVM_MALE.pdf]

# THCA\_FEMALE

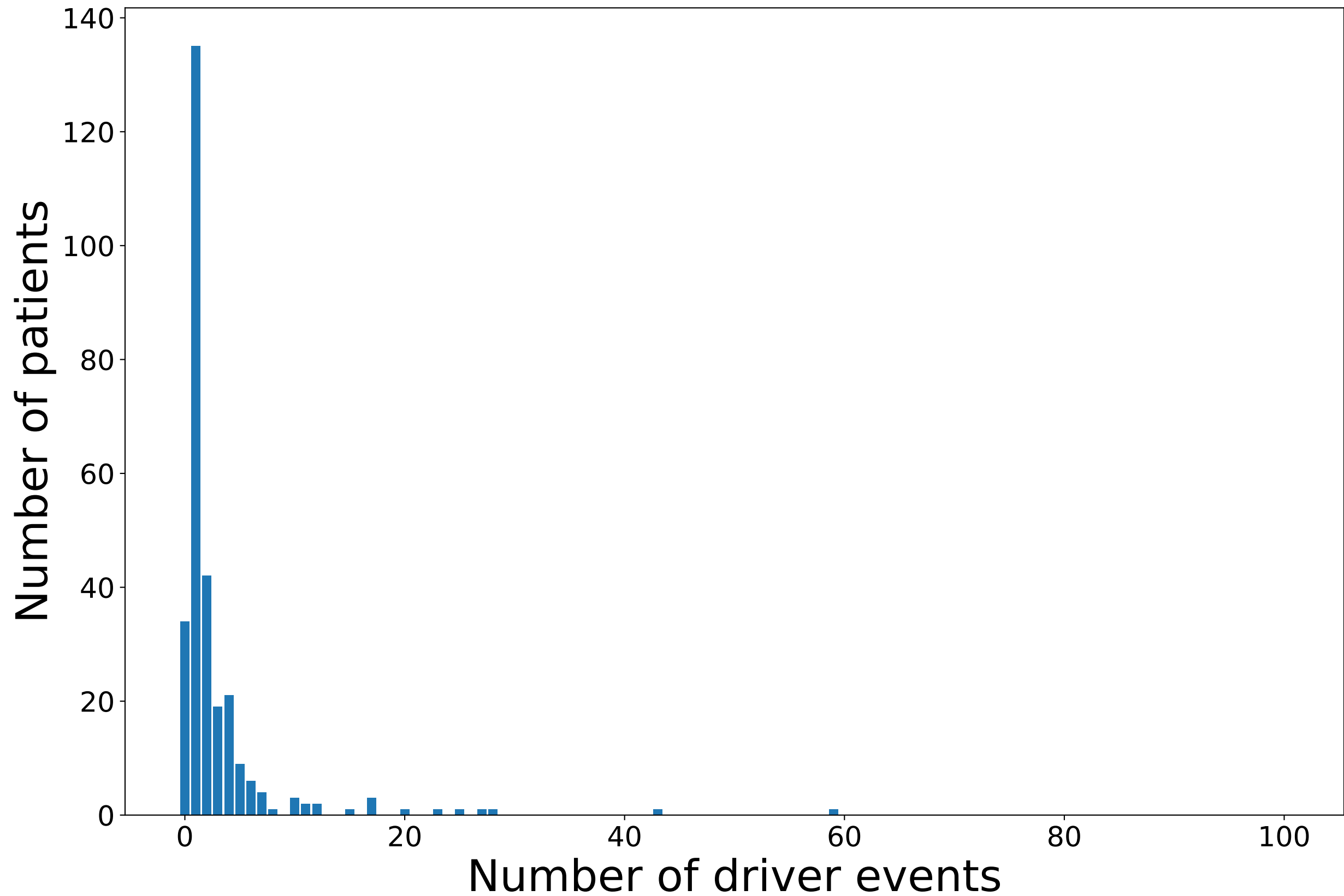

Supplement: S2 Files — (ZIP) [file pgen.1009996.s002.zip › PANCAN/patient distributions/2021_11_23_14_43_THCA_FEMALE.pdf]

# PRAD

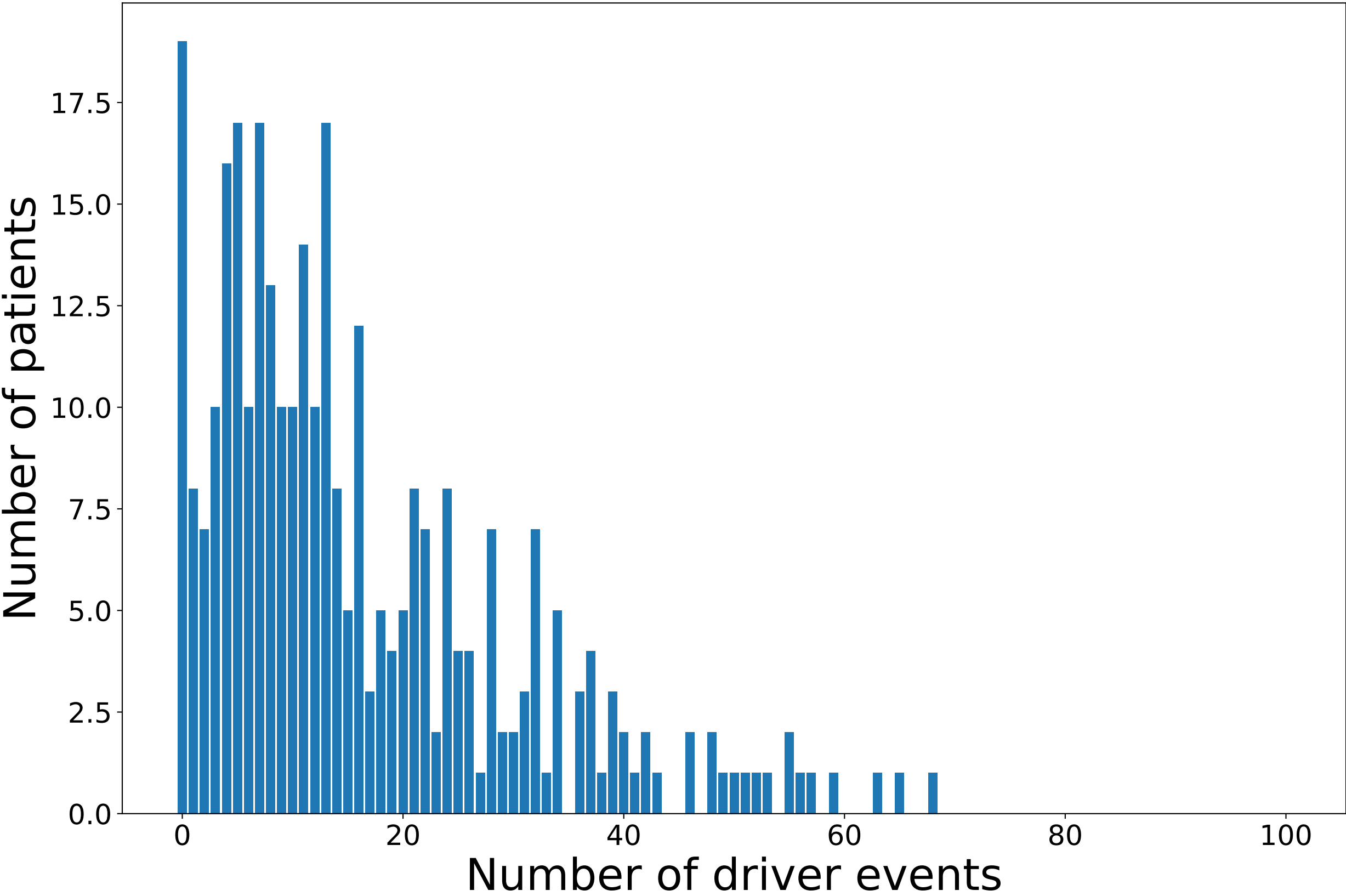

Supplement: S2 Files — (ZIP) [file pgen.1009996.s002.zip › PANCAN/patient distributions/2021_11_23_14_43_PRAD.pdf]

# PANCAN\_FEMALE

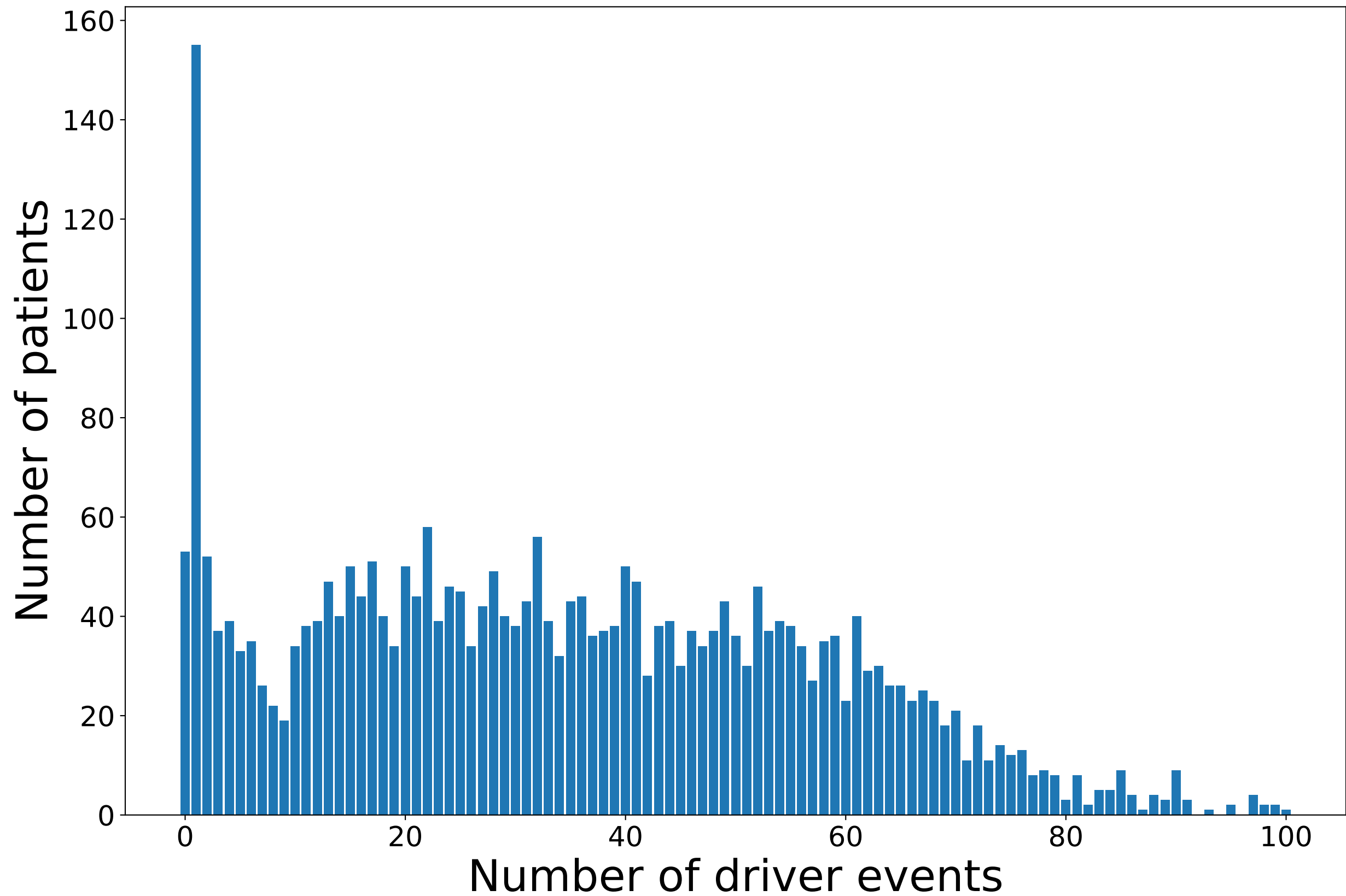

Supplement: S2 Files — (ZIP) [file pgen.1009996.s002.zip › PANCAN/patient distributions/2021_11_23_14_43_PANCAN_FEMALE.pdf]
