# Supplementary figures and images for: Comprehensive patient-level classification and quantification of driver events in TCGA PanCanAtlas cohorts (part 3 of 6)
Source: PLoS Genet. 2022 Jan 14;18(1):e1009996. doi: 10.1371/journal.pgen.1009996 (PMC8759692; doi:10.1371/journal.pgen.1009996)

# LUSC\_MALE

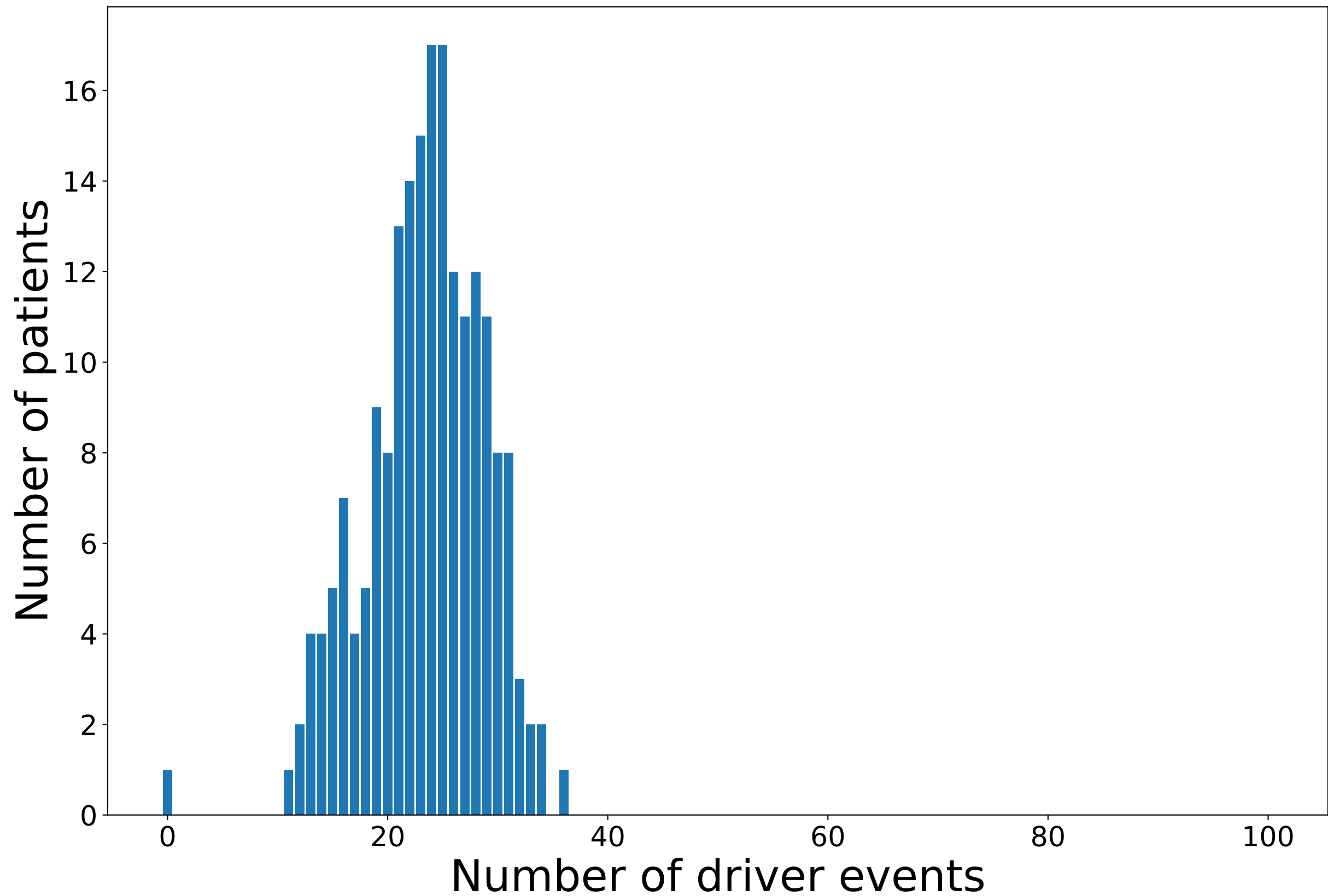

Supplement: S3 Files — (ZIP) [file pgen.1009996.s003.zip › COHORTS/patient distributions/2021_11_23_14_20_LUSC_MALE.pdf]

# PRAD\_MALE

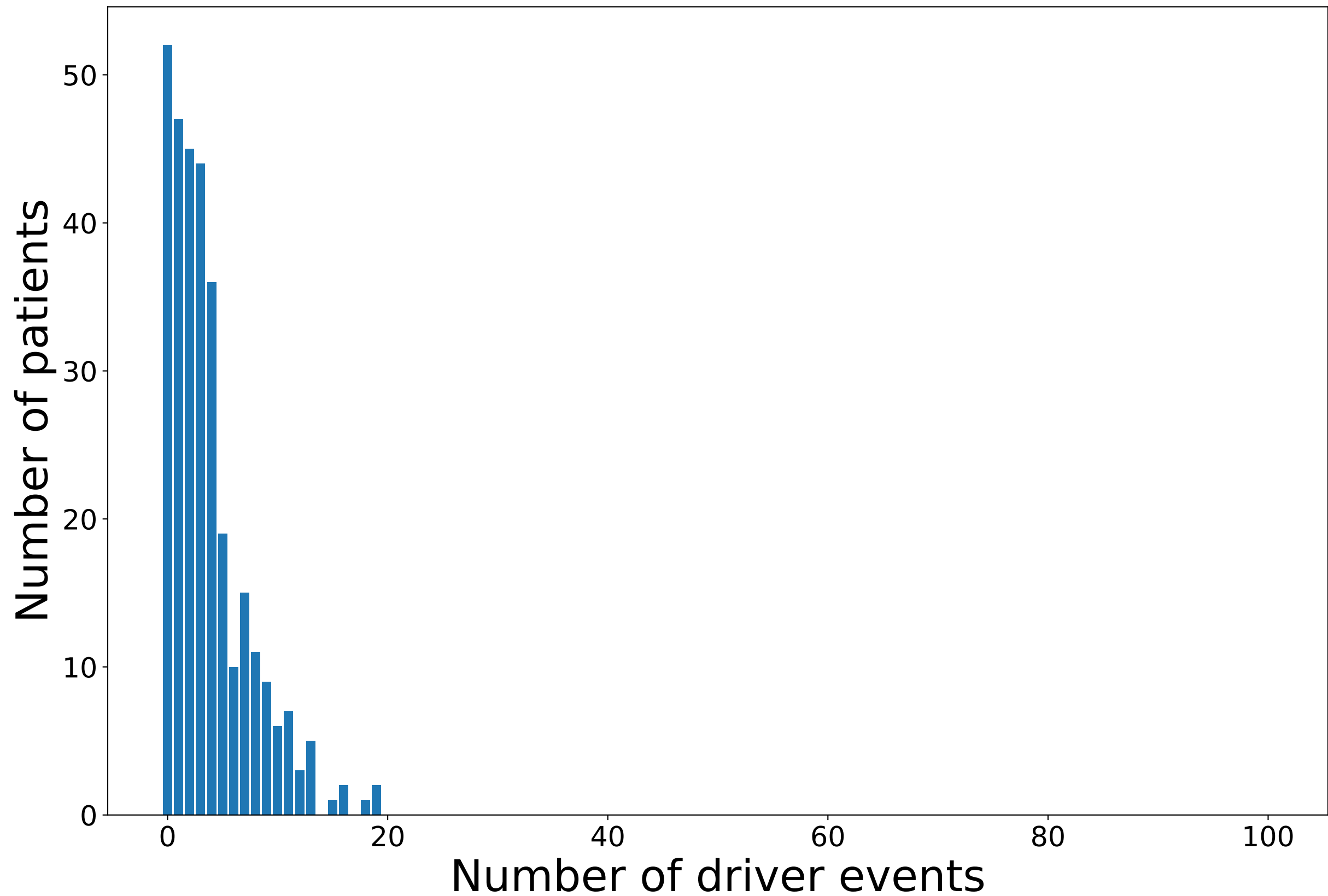

Supplement: S3 Files — (ZIP) [file pgen.1009996.s003.zip › COHORTS/patient distributions/2021_11_23_14_20_PRAD_MALE.pdf]

# CHOL\_FEMALE

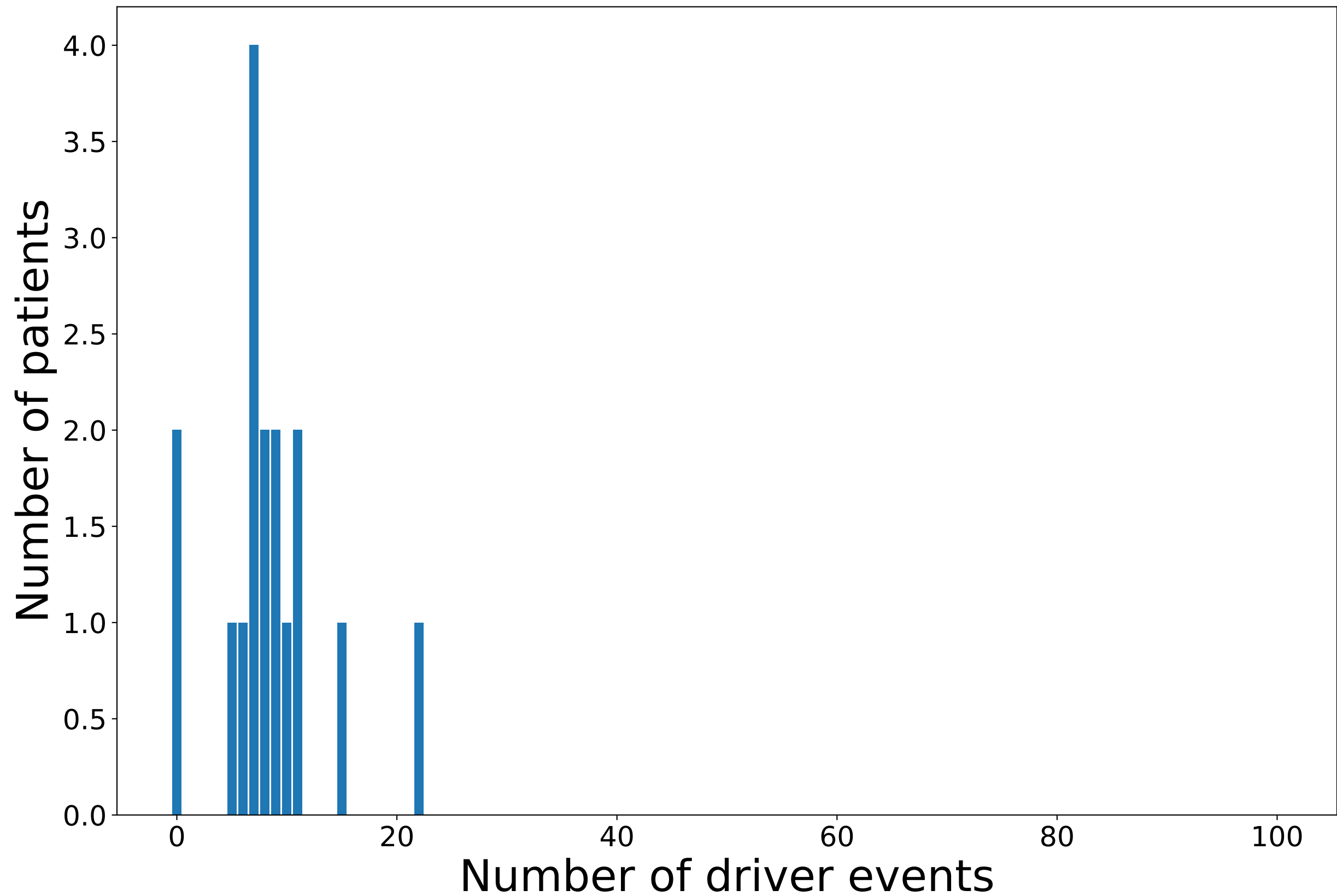

Supplement: S3 Files — (ZIP) [file pgen.1009996.s003.zip › COHORTS/patient distributions/2021_11_23_14_20_CHOL_FEMALE.pdf]

# TGCT

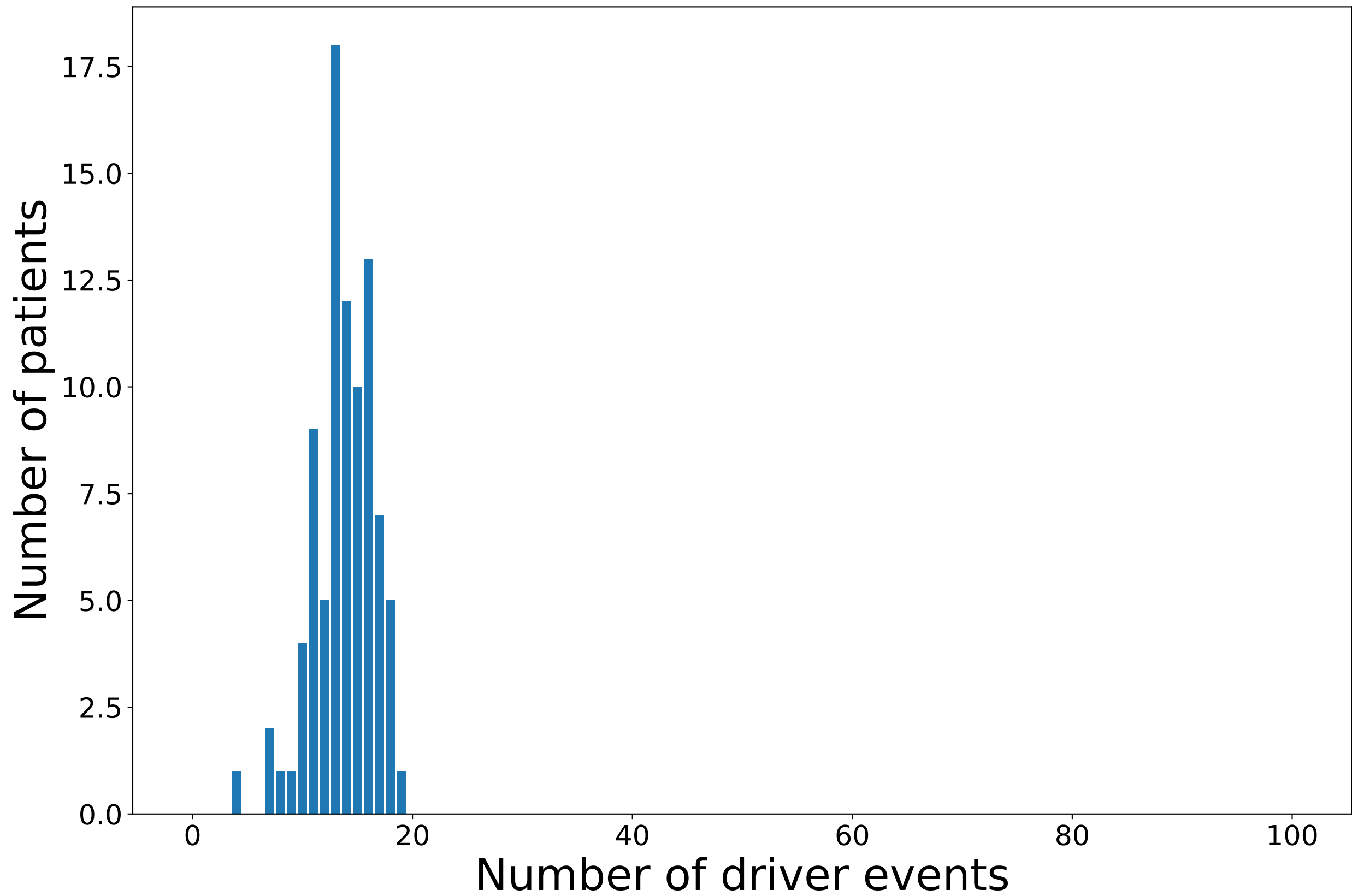

Supplement: S3 Files — (ZIP) [file pgen.1009996.s003.zip › COHORTS/patient distributions/2021_11_23_14_20_TGCT.pdf]

# KIRP\_FEMALE

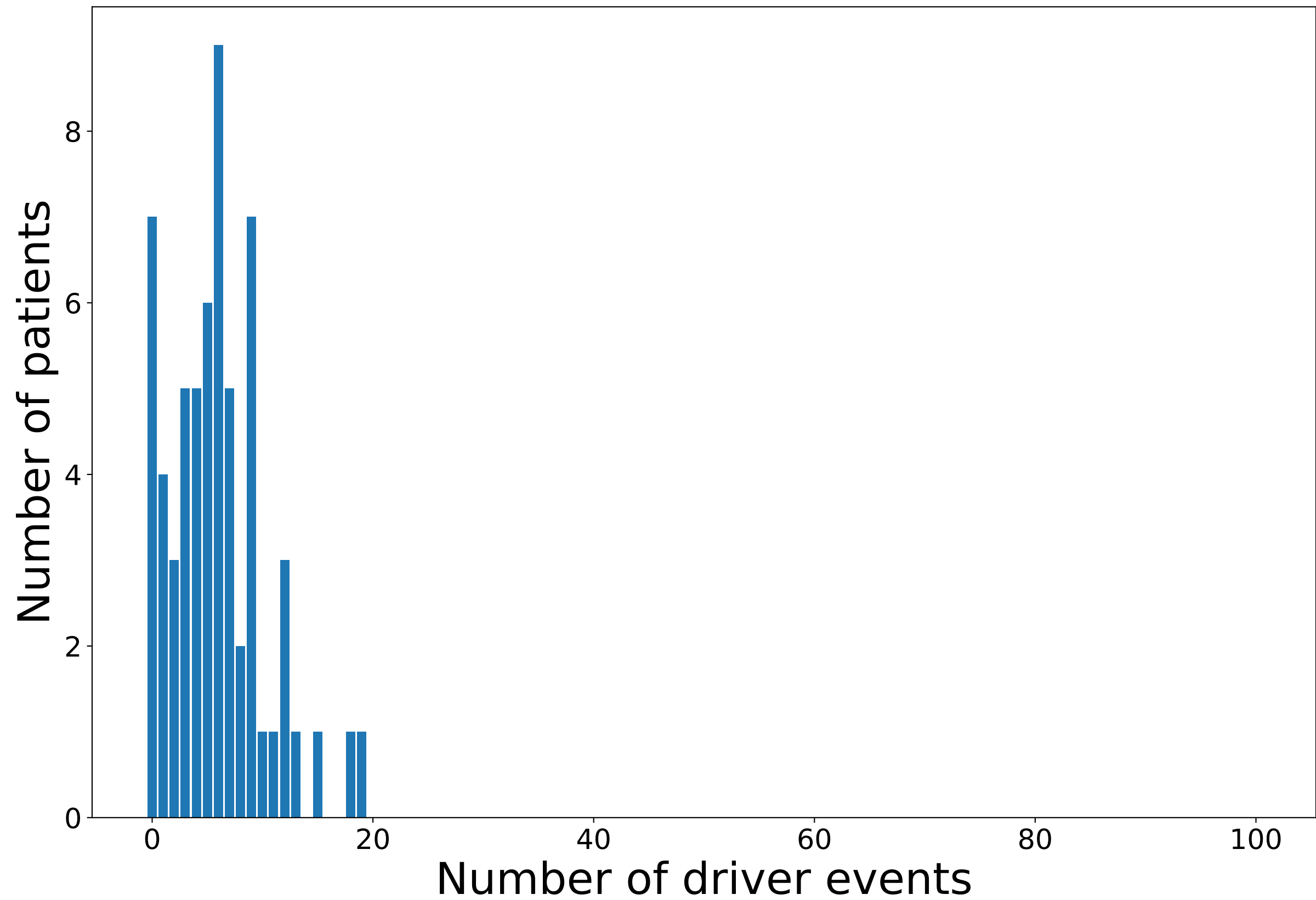

Supplement: S3 Files — (ZIP) [file pgen.1009996.s003.zip › COHORTS/patient distributions/2021_11_23_14_20_KIRP_FEMALE.pdf]

# KICH\_FEMALE

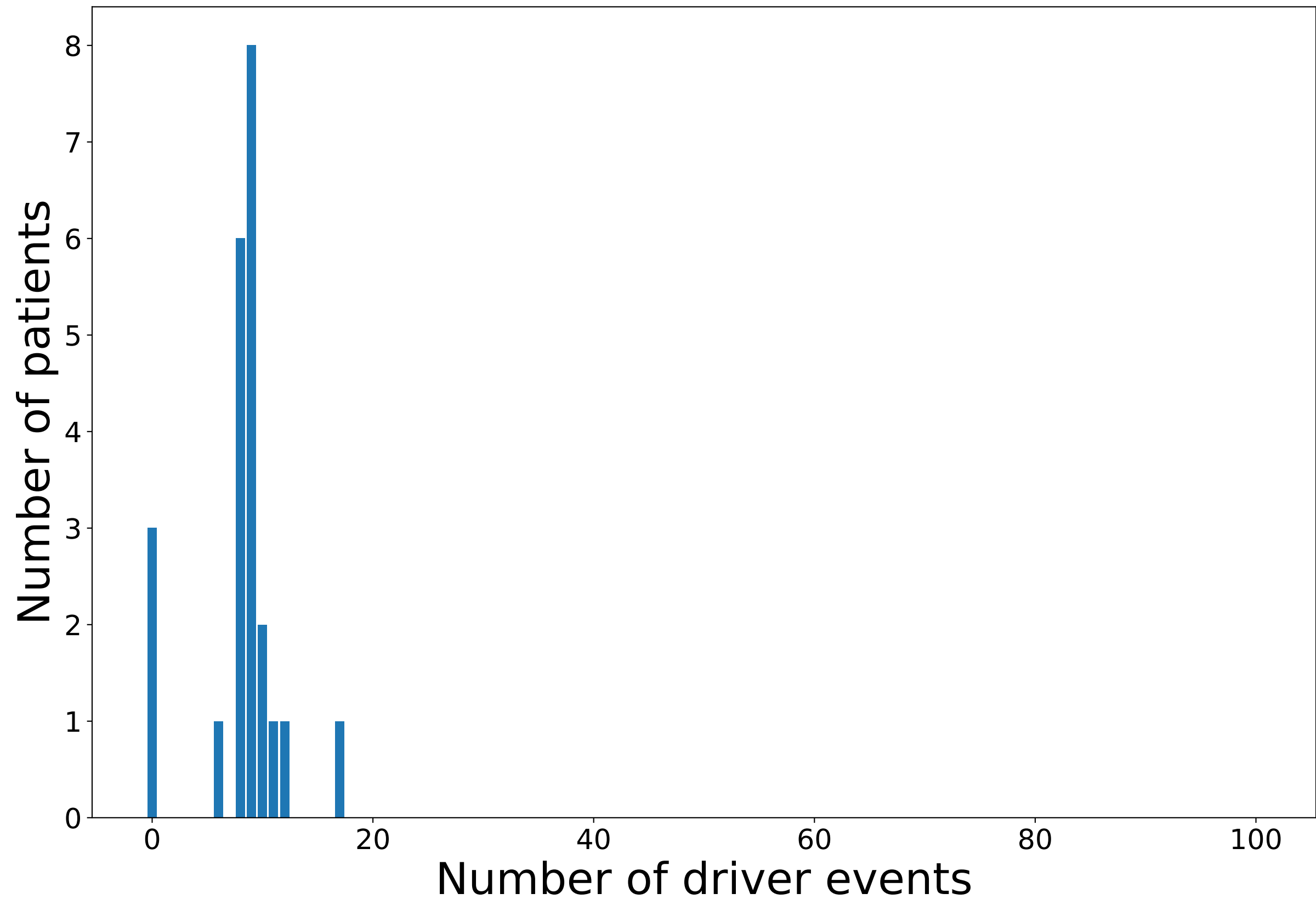

Supplement: S3 Files — (ZIP) [file pgen.1009996.s003.zip › COHORTS/patient distributions/2021_11_23_14_20_KICH_FEMALE.pdf]

# TGCT\_MALE

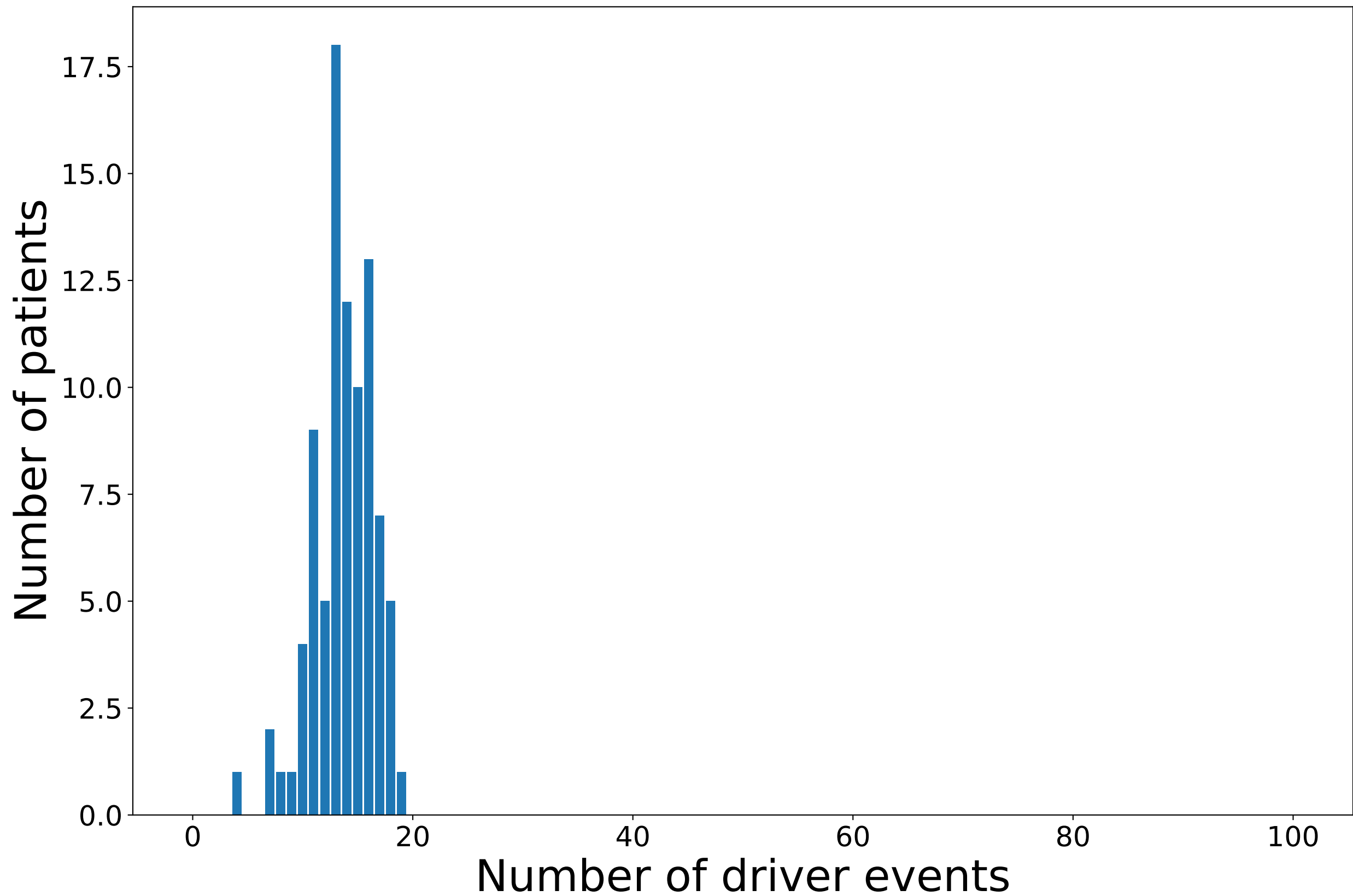

Supplement: S3 Files — (ZIP) [file pgen.1009996.s003.zip › COHORTS/patient distributions/2021_11_23_14_20_TGCT_MALE.pdf]

# ESCA\_FEMALE

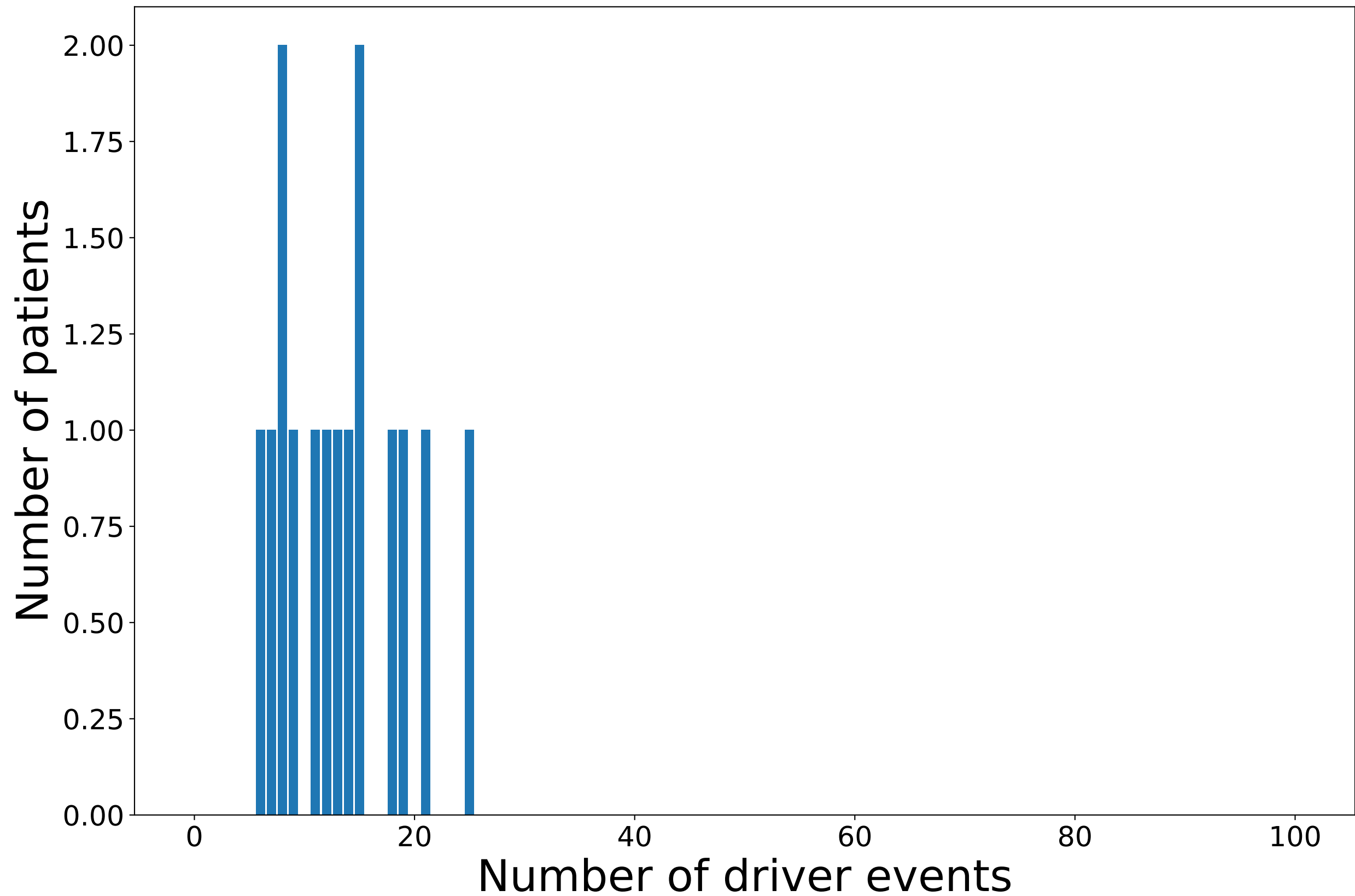

Supplement: S3 Files — (ZIP) [file pgen.1009996.s003.zip › COHORTS/patient distributions/2021_11_23_14_20_ESCA_FEMALE.pdf]

# PCPG

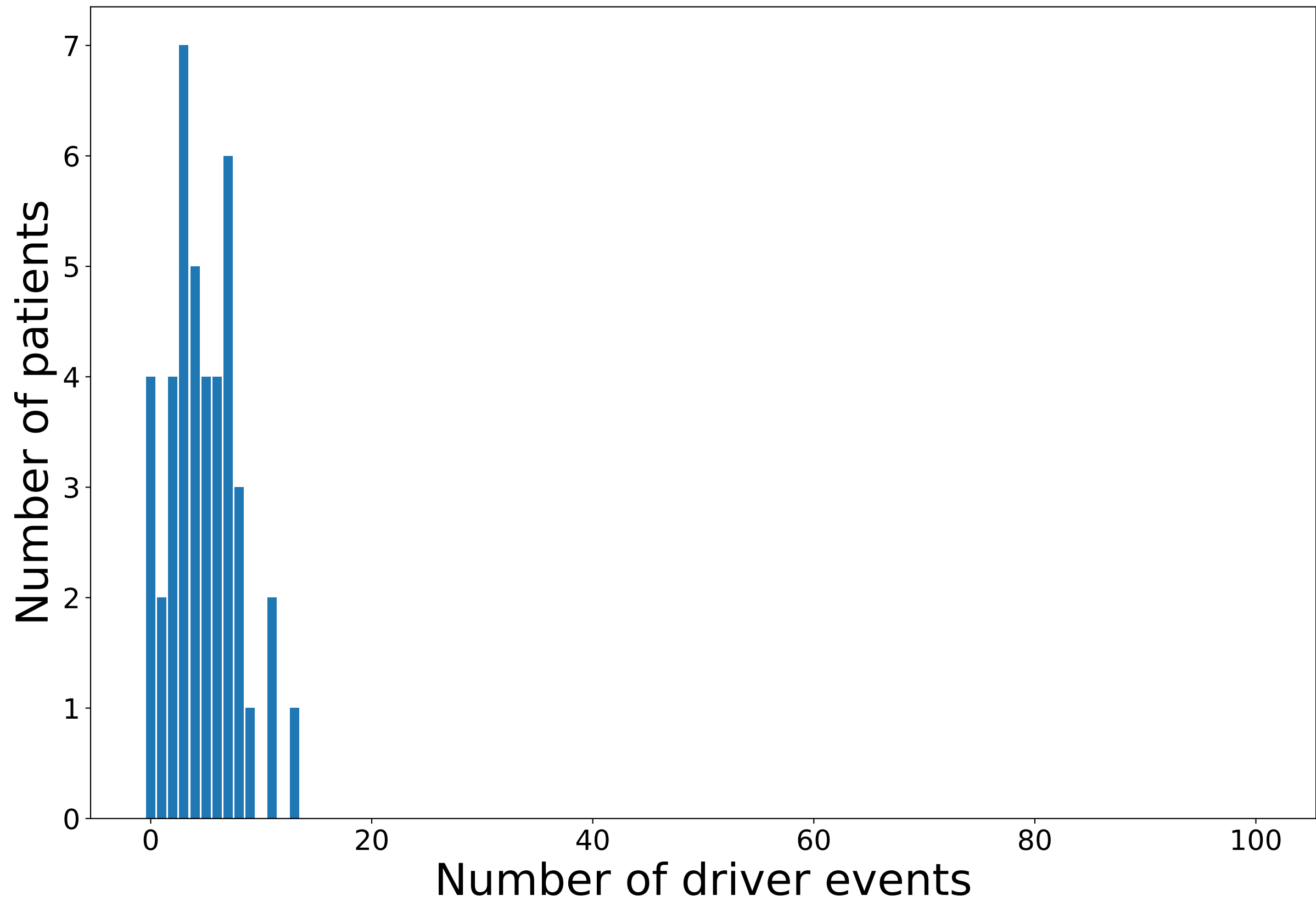

Supplement: S3 Files — (ZIP) [file pgen.1009996.s003.zip › COHORTS/patient distributions/2021_11_23_14_20_PCPG.pdf]

# LUSC

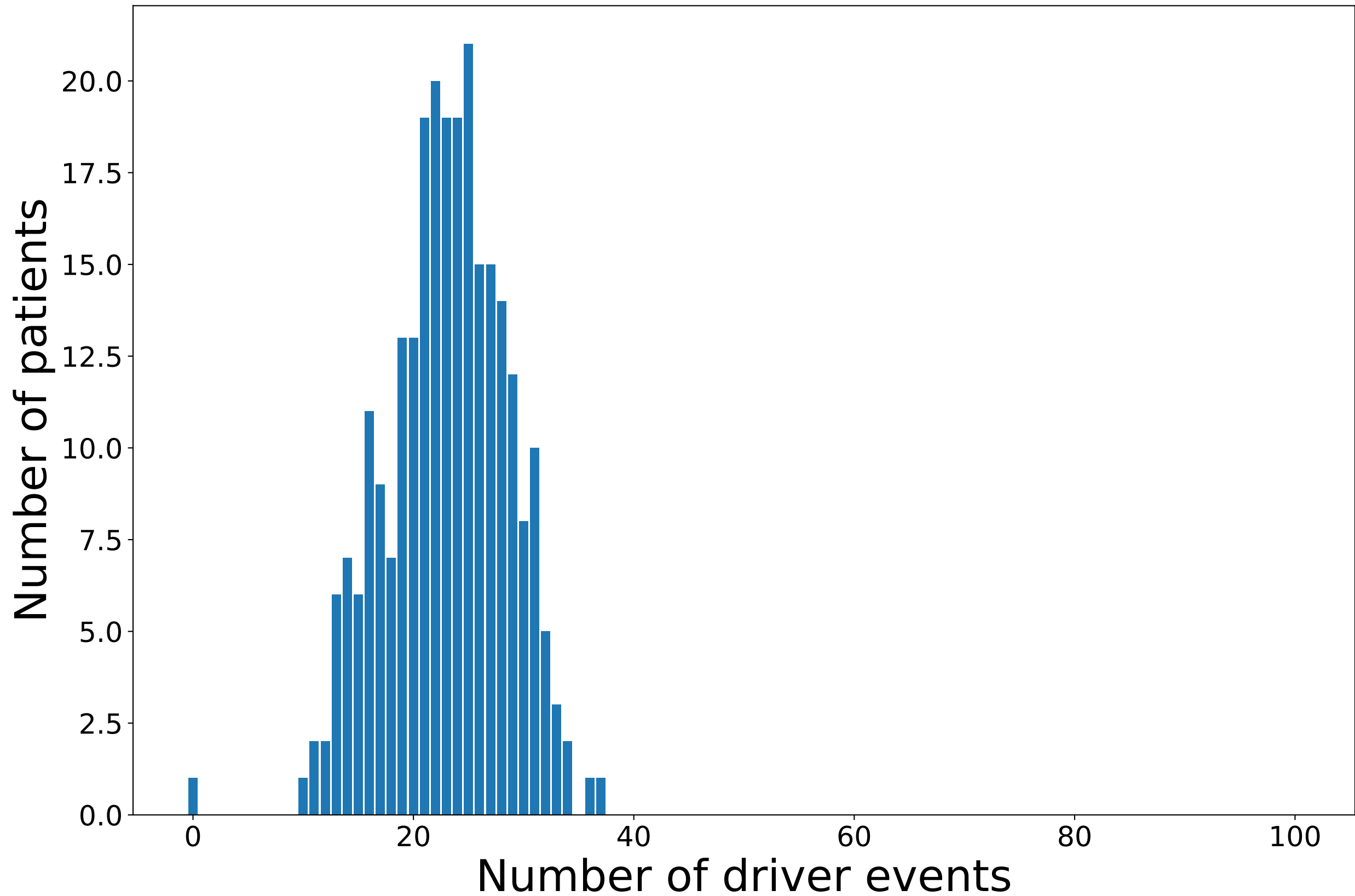

Supplement: S3 Files — (ZIP) [file pgen.1009996.s003.zip › COHORTS/patient distributions/2021_11_23_14_20_LUSC.pdf]

# MESO\_MALE

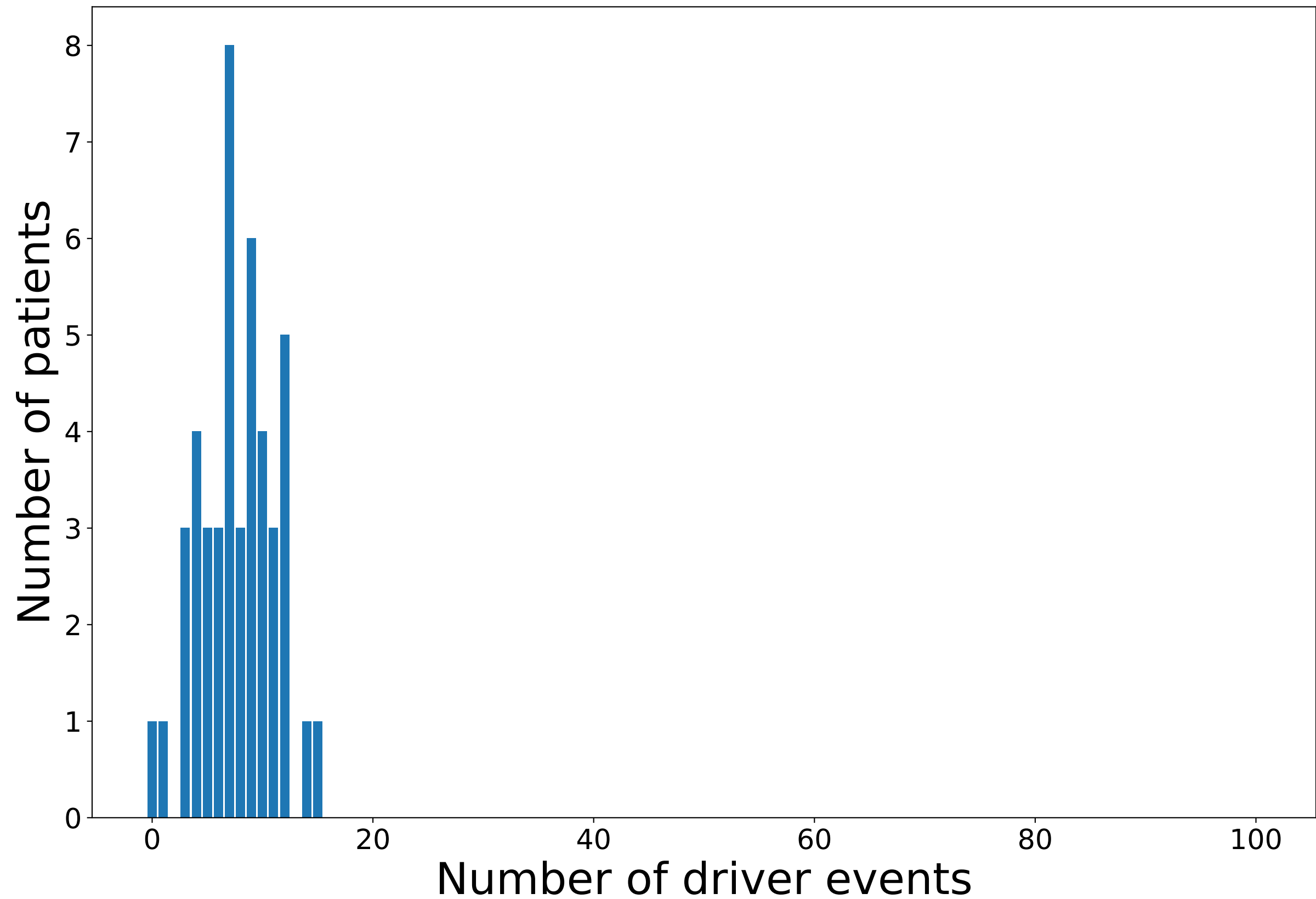

Supplement: S3 Files — (ZIP) [file pgen.1009996.s003.zip › COHORTS/patient distributions/2021_11_23_14_20_MESO_MALE.pdf]

# DLBC\_FEMALE

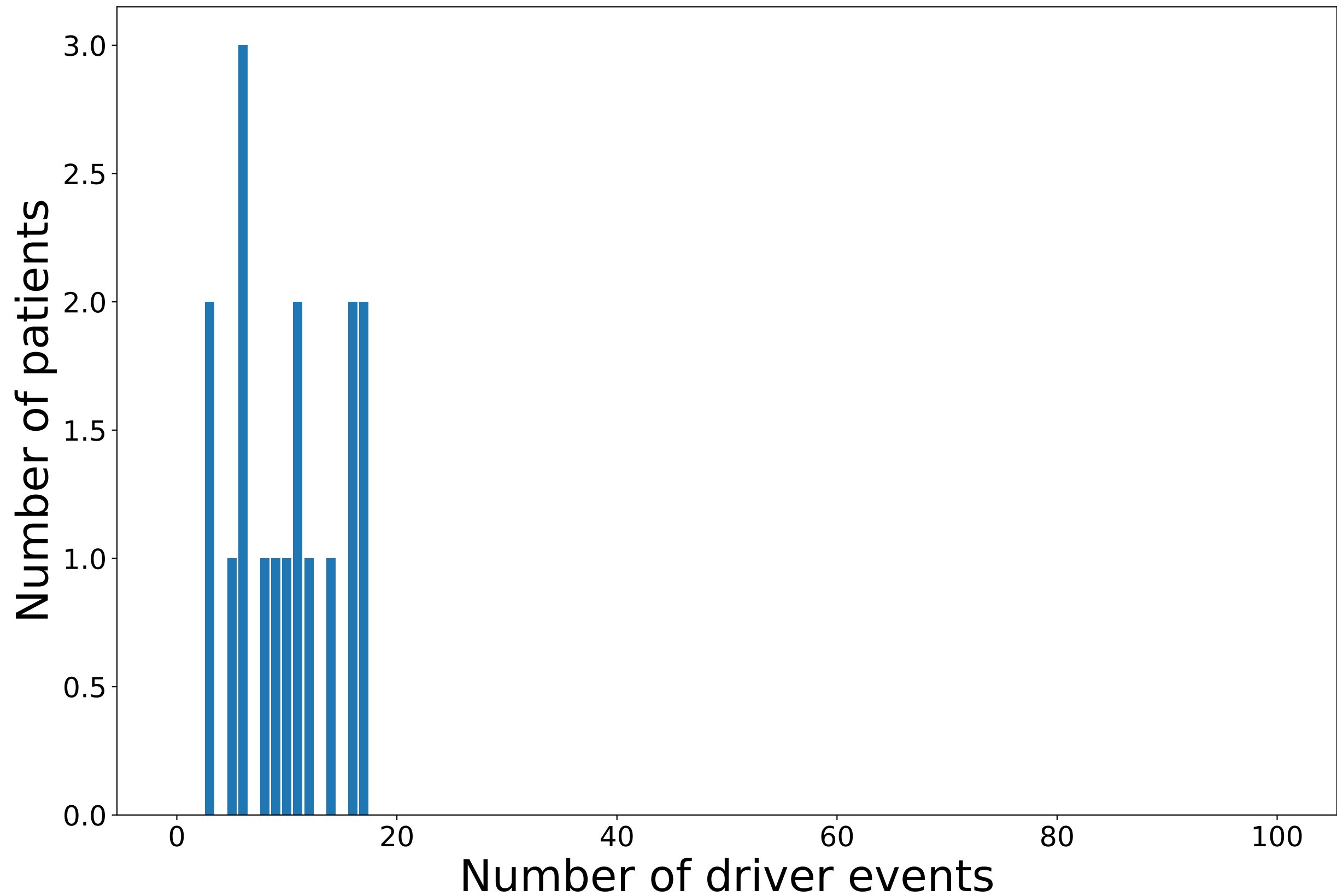

Supplement: S3 Files — (ZIP) [file pgen.1009996.s003.zip › COHORTS/patient distributions/2021_11_23_14_20_DLBC_FEMALE.pdf]

# UVM\_FEMALE

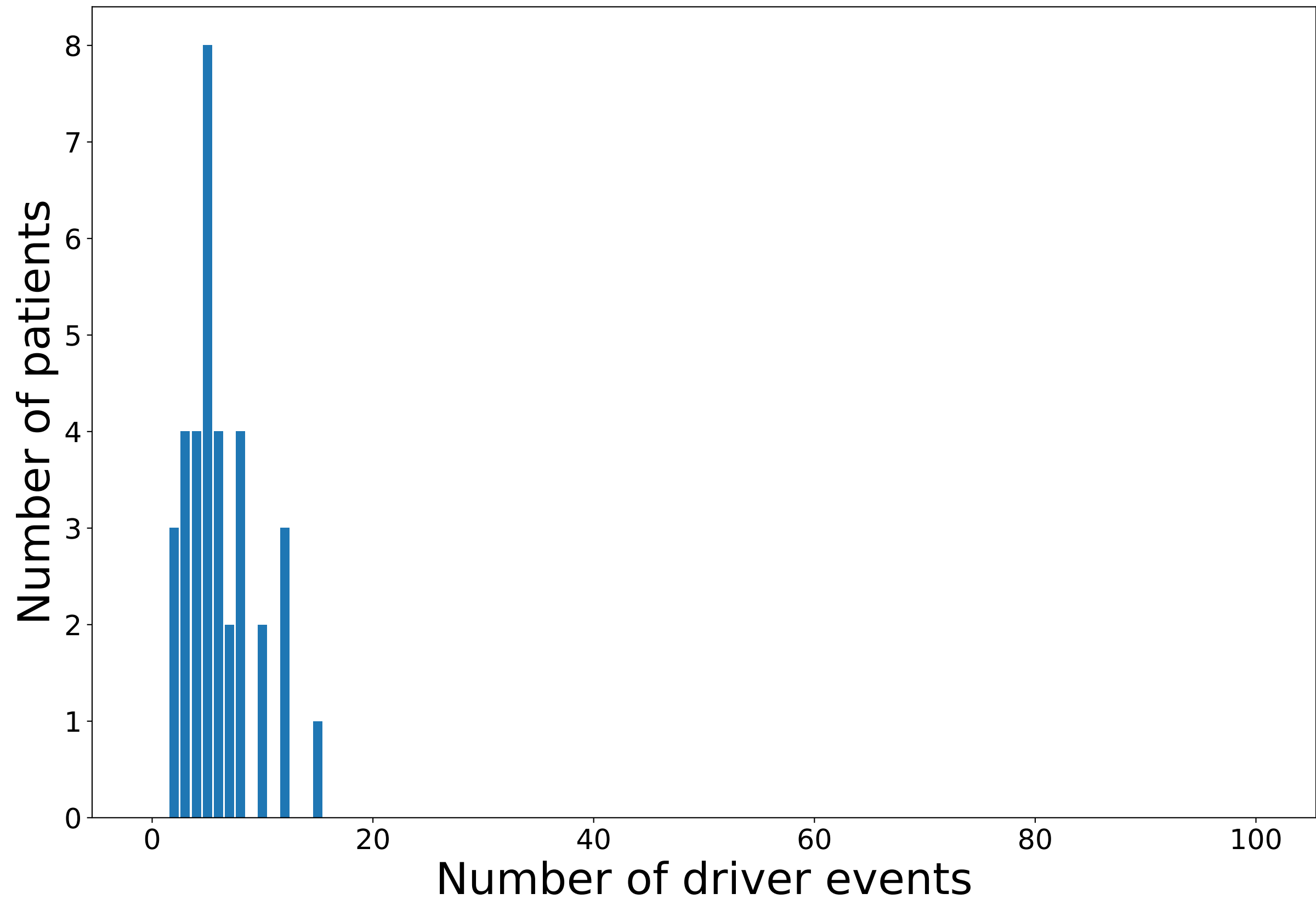

Supplement: S3 Files — (ZIP) [file pgen.1009996.s003.zip › COHORTS/patient distributions/2021_11_23_14_20_UVM_FEMALE.pdf]

# BRCA\_FEMALE

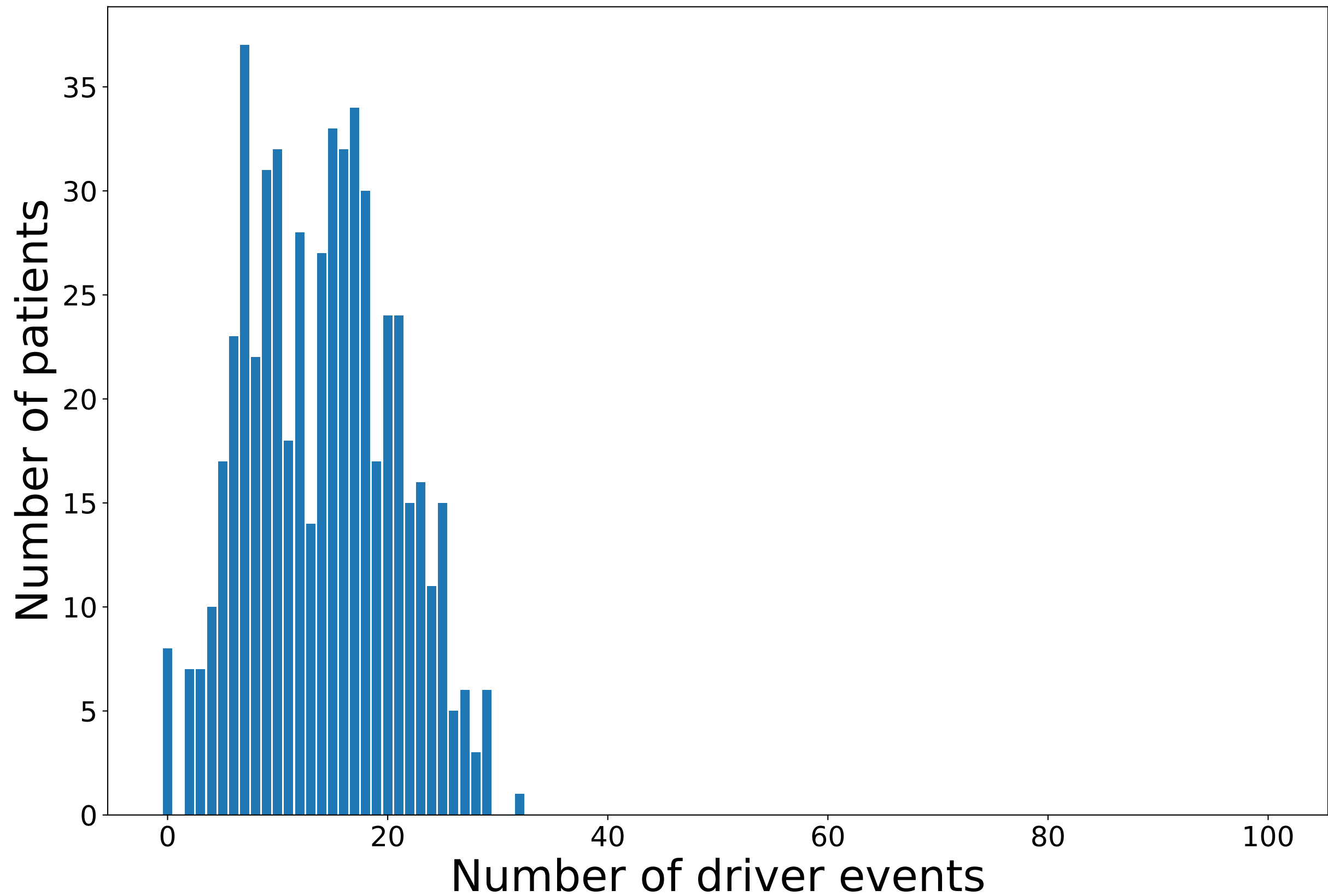

Supplement: S3 Files — (ZIP) [file pgen.1009996.s003.zip › COHORTS/patient distributions/2021_11_23_14_20_BRCA_FEMALE.pdf]

# GBM\_MALE

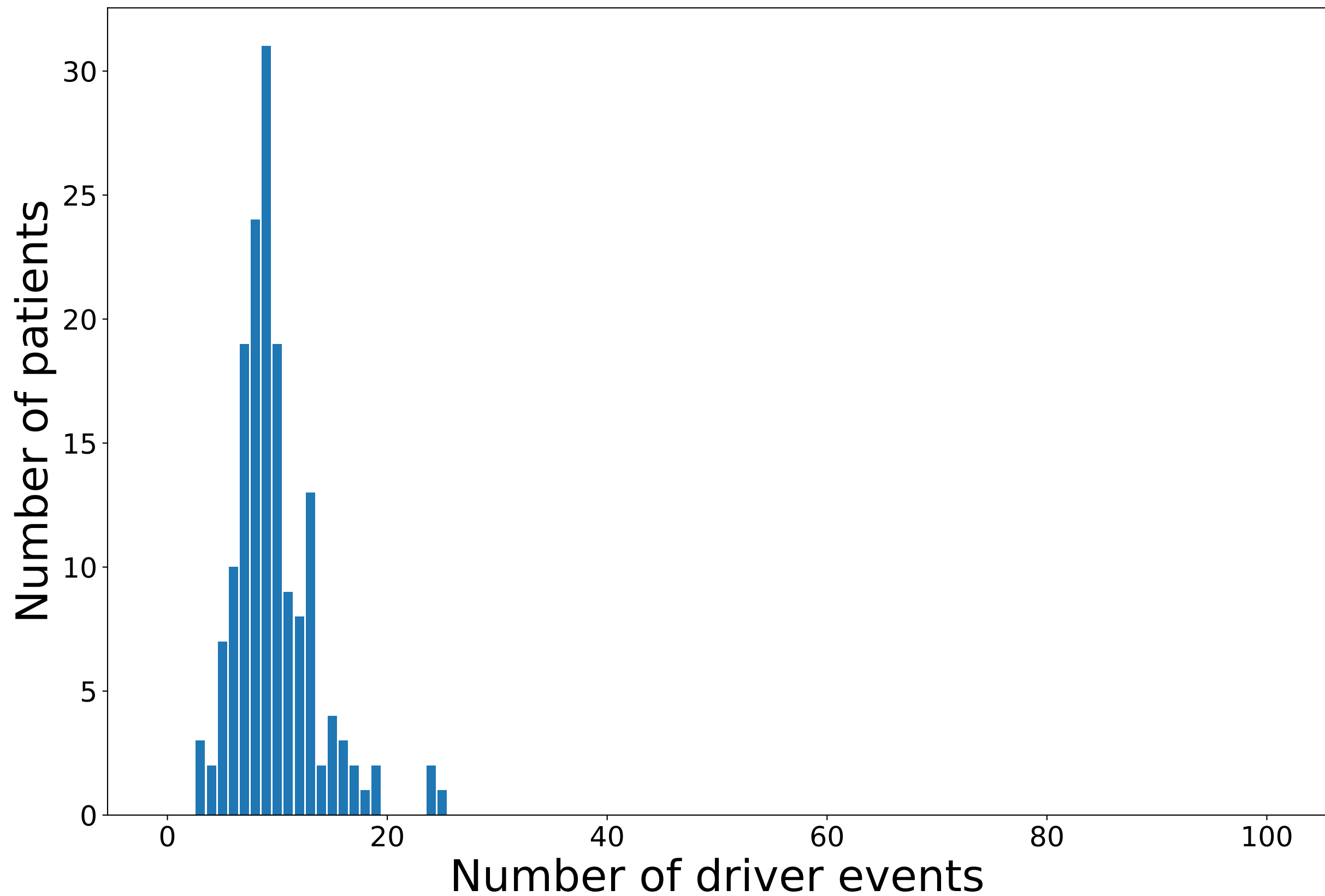

Supplement: S3 Files — (ZIP) [file pgen.1009996.s003.zip › COHORTS/patient distributions/2021_11_23_14_20_GBM_MALE.pdf]

# SKCM\_MALE

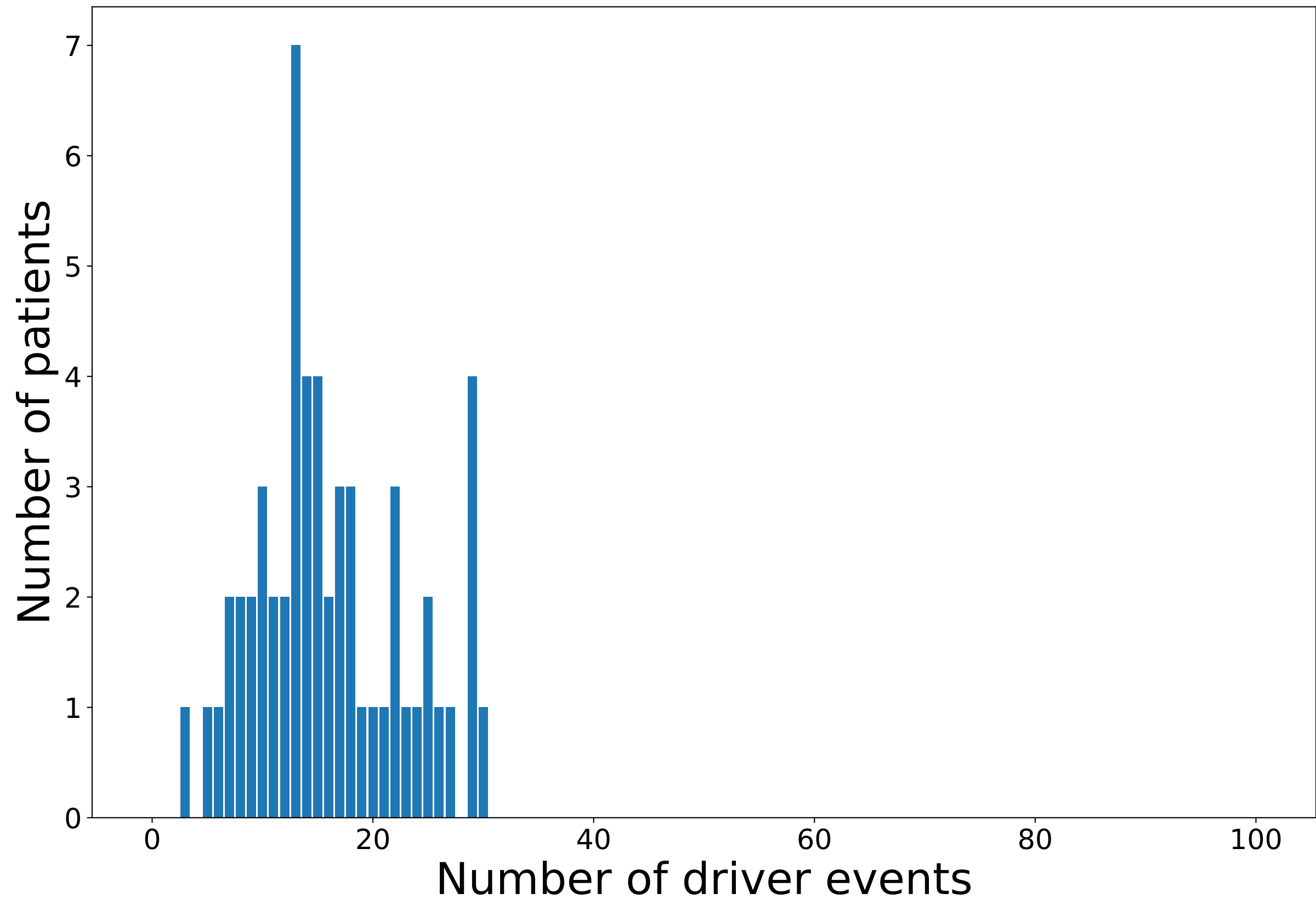

Supplement: S3 Files — (ZIP) [file pgen.1009996.s003.zip › COHORTS/patient distributions/2021_11_23_14_20_SKCM_MALE.pdf]

# SARC\_MALE

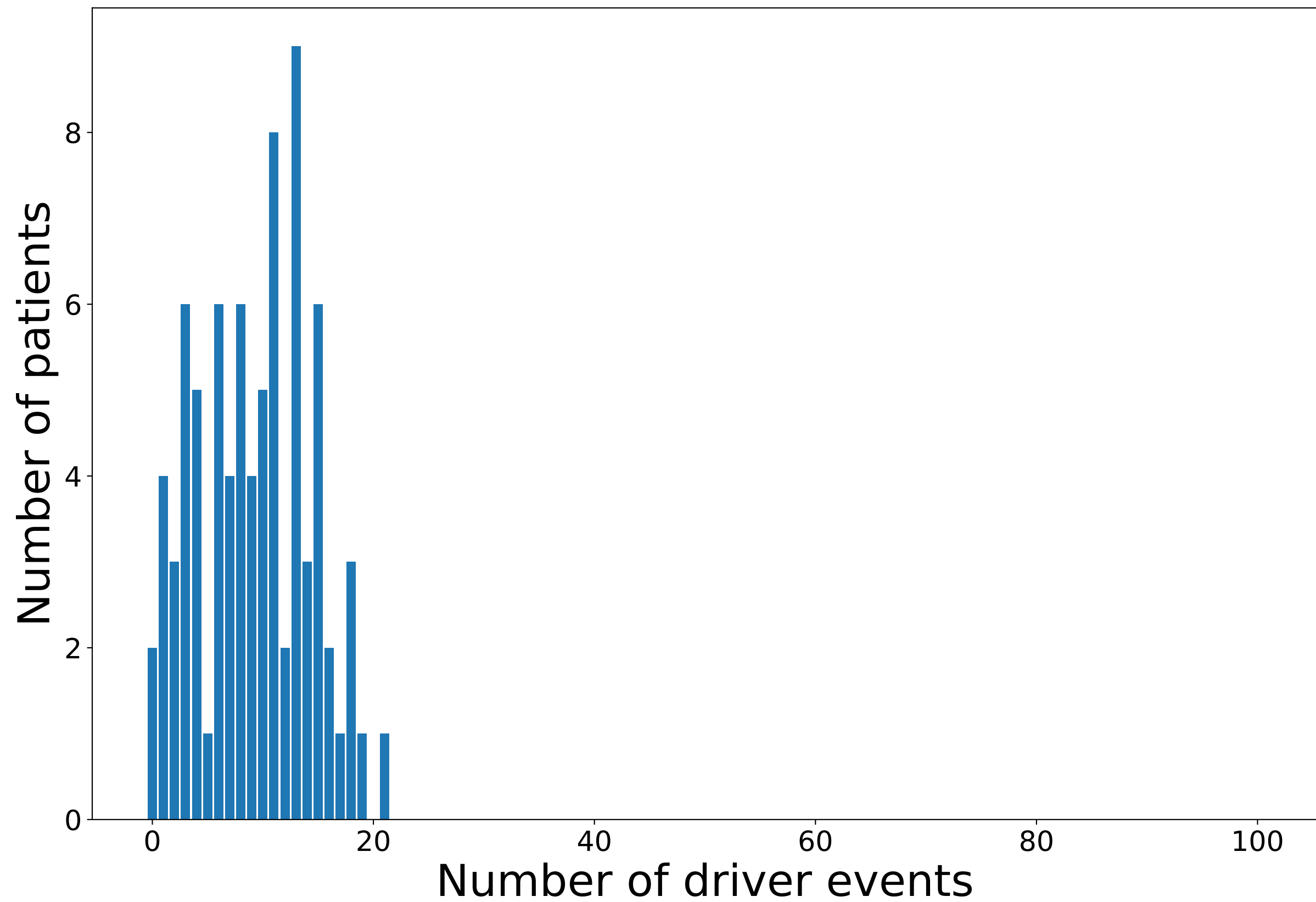

Supplement: S3 Files — (ZIP) [file pgen.1009996.s003.zip › COHORTS/patient distributions/2021_11_23_14_20_SARC_MALE.pdf]

# HNSC

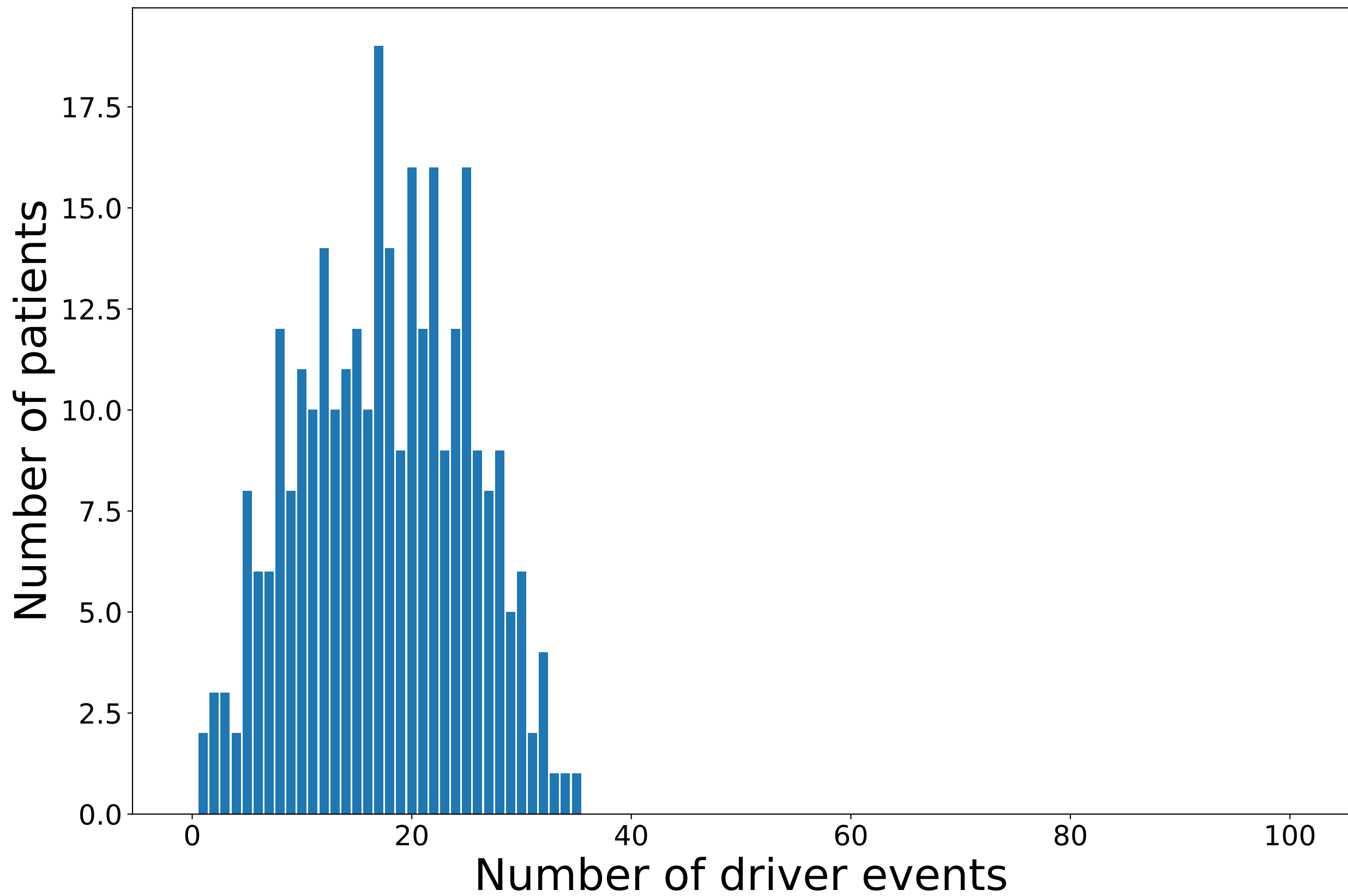

Supplement: S3 Files — (ZIP) [file pgen.1009996.s003.zip › COHORTS/patient distributions/2021_11_23_14_20_HNSC.pdf]

# PCPG\_FEMALE

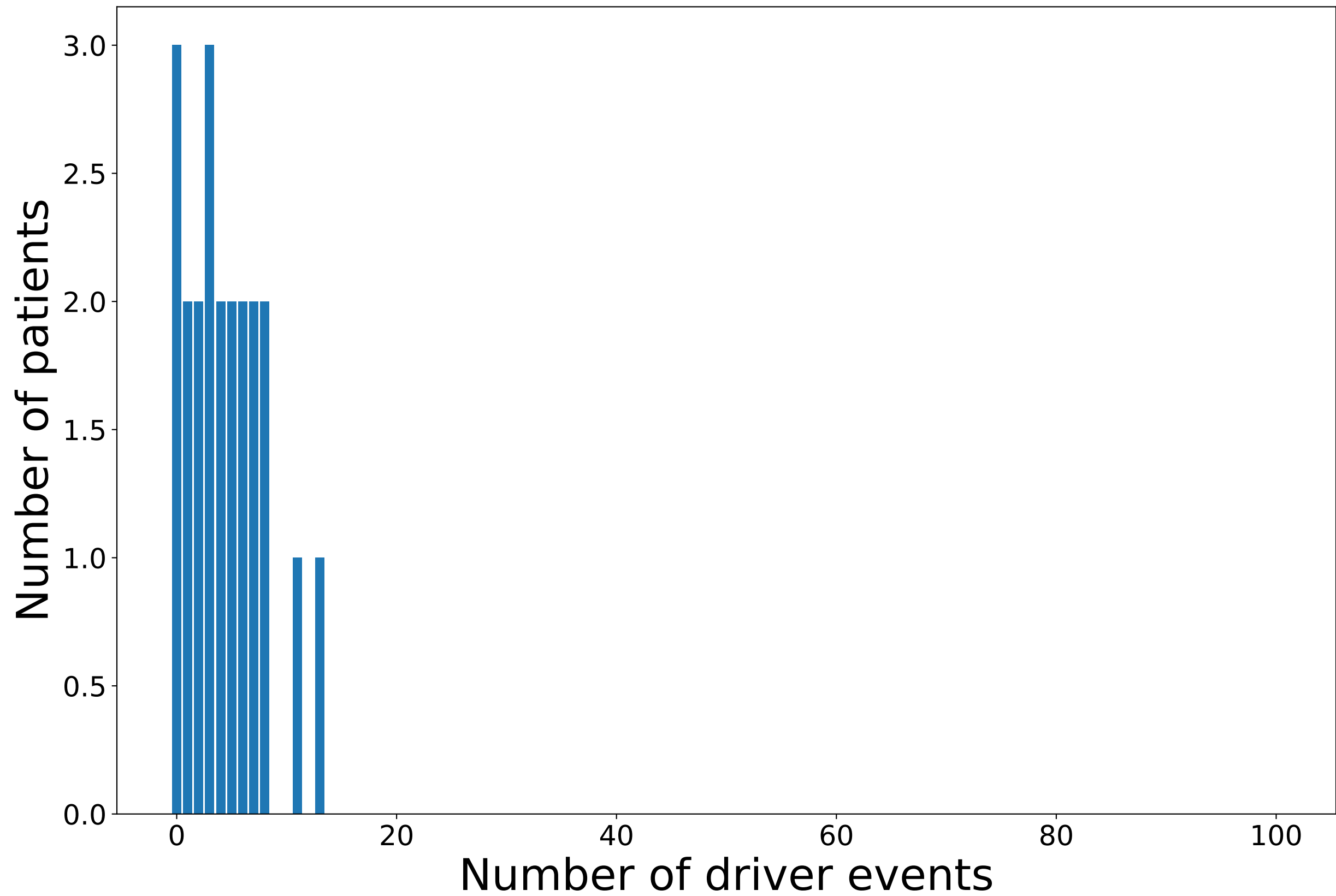

Supplement: S3 Files — (ZIP) [file pgen.1009996.s003.zip › COHORTS/patient distributions/2021_11_23_14_20_PCPG_FEMALE.pdf]

OV

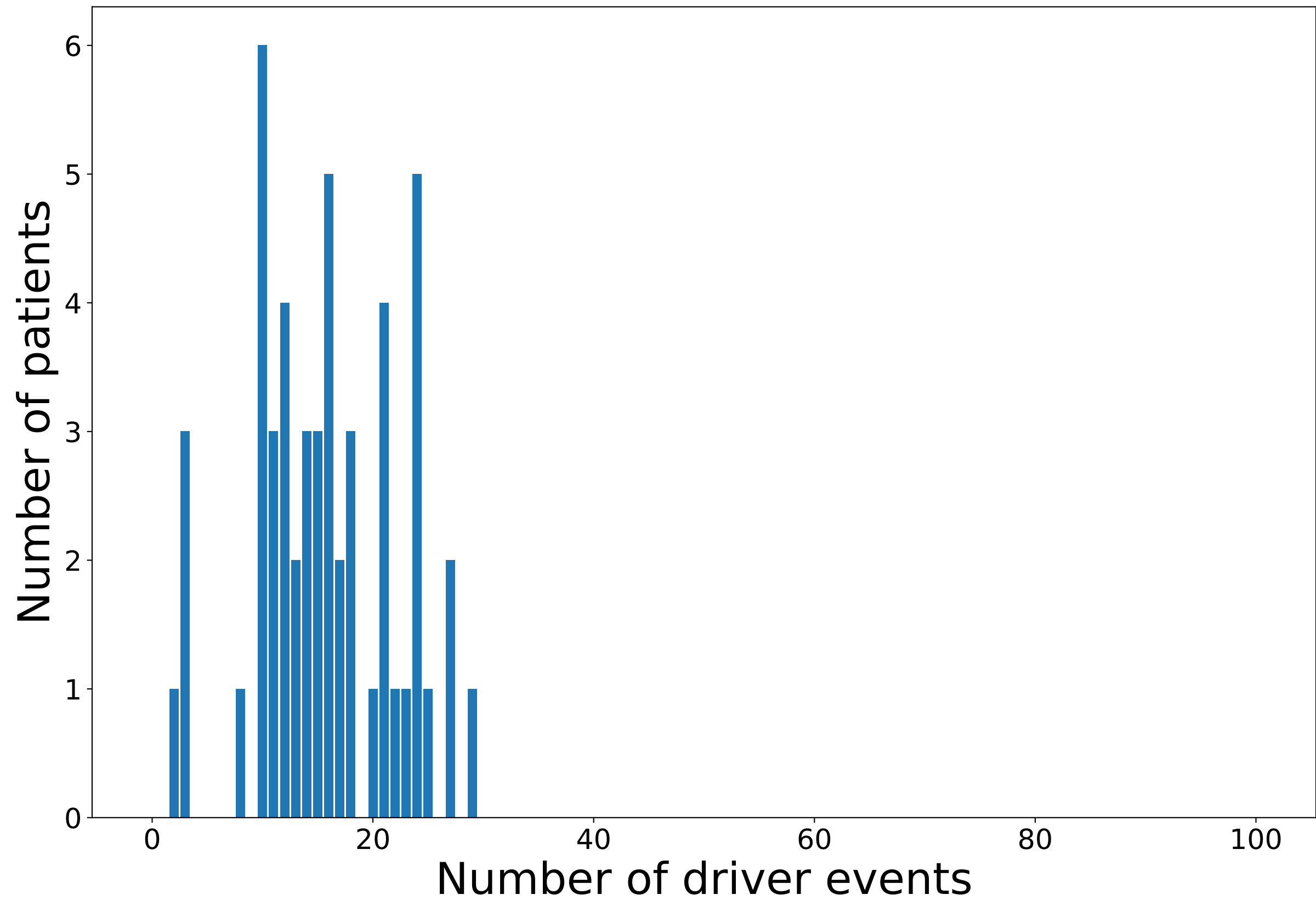

Supplement: S3 Files — (ZIP) [file pgen.1009996.s003.zip › COHORTS/patient distributions/2021_11_23_14_20_OV.pdf]

# CESC\_FEMALE

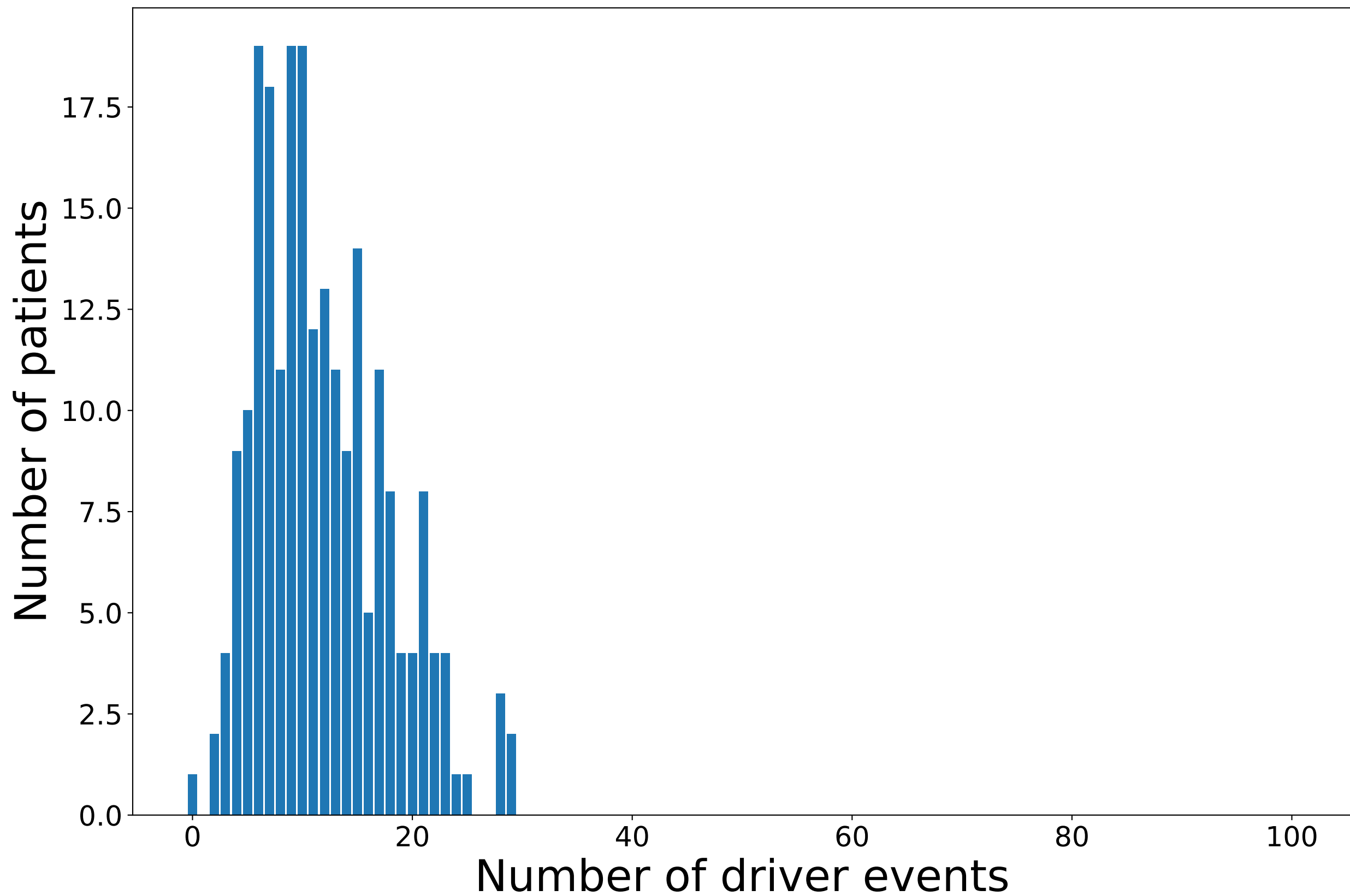

Supplement: S3 Files — (ZIP) [file pgen.1009996.s003.zip › COHORTS/patient distributions/2021_11_23_14_20_CESC_FEMALE.pdf]

# ESCA

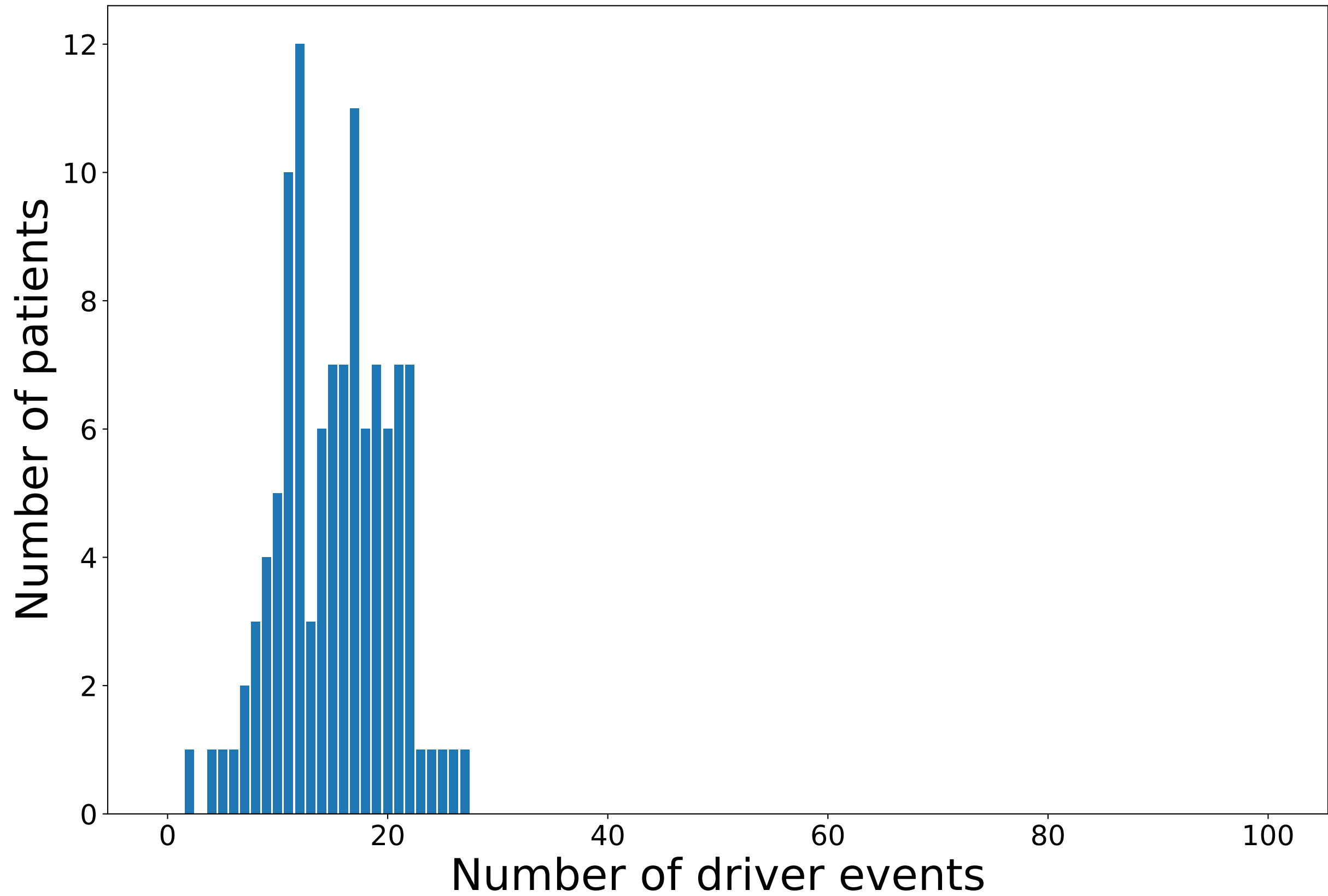

Supplement: S3 Files — (ZIP) [file pgen.1009996.s003.zip › COHORTS/patient distributions/2021_11_23_14_20_ESCA.pdf]

# UCEC\_FEMALE

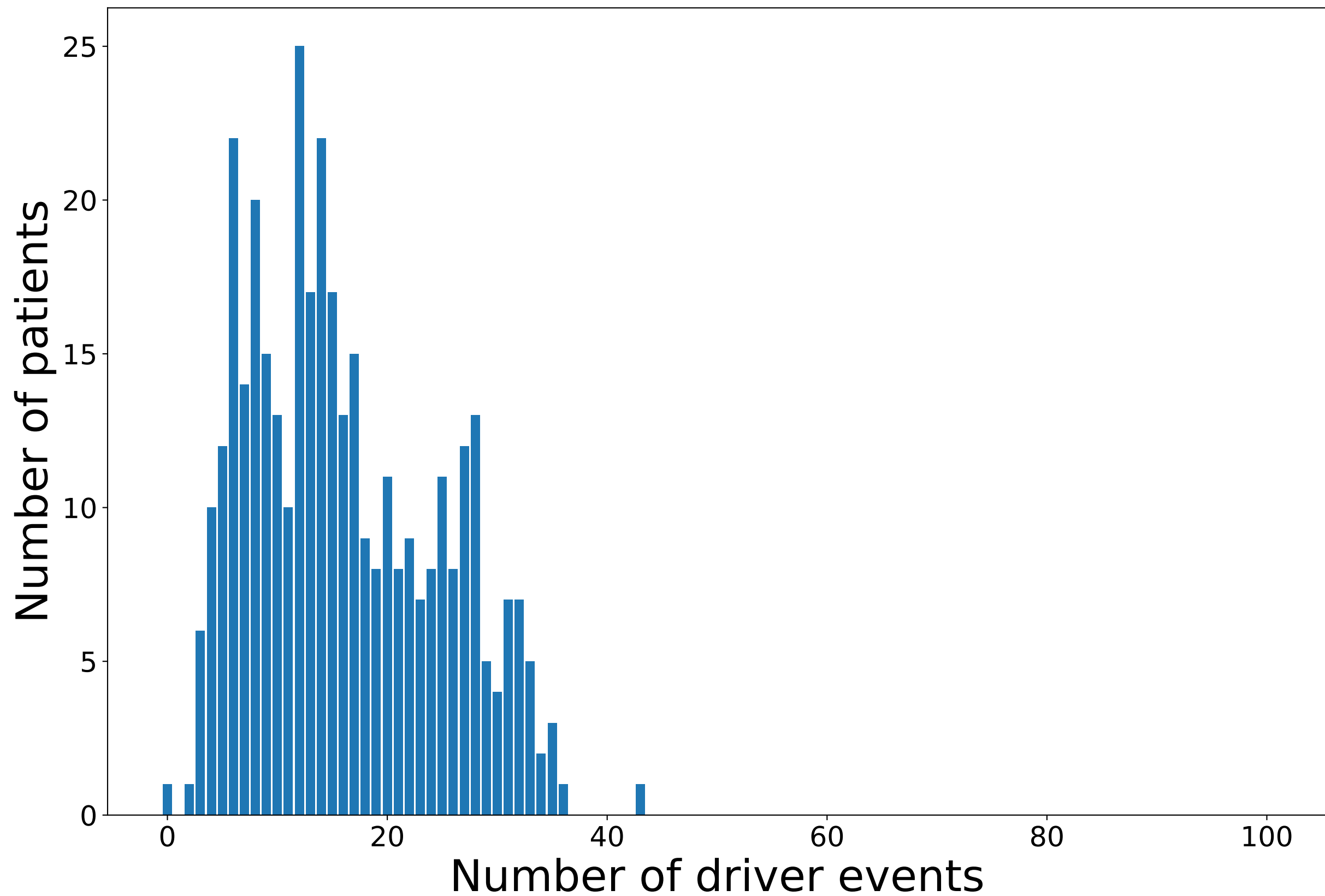

Supplement: S3 Files — (ZIP) [file pgen.1009996.s003.zip › COHORTS/patient distributions/2021_11_23_14_20_UCEC_FEMALE.pdf]

# PCPG\_MALE

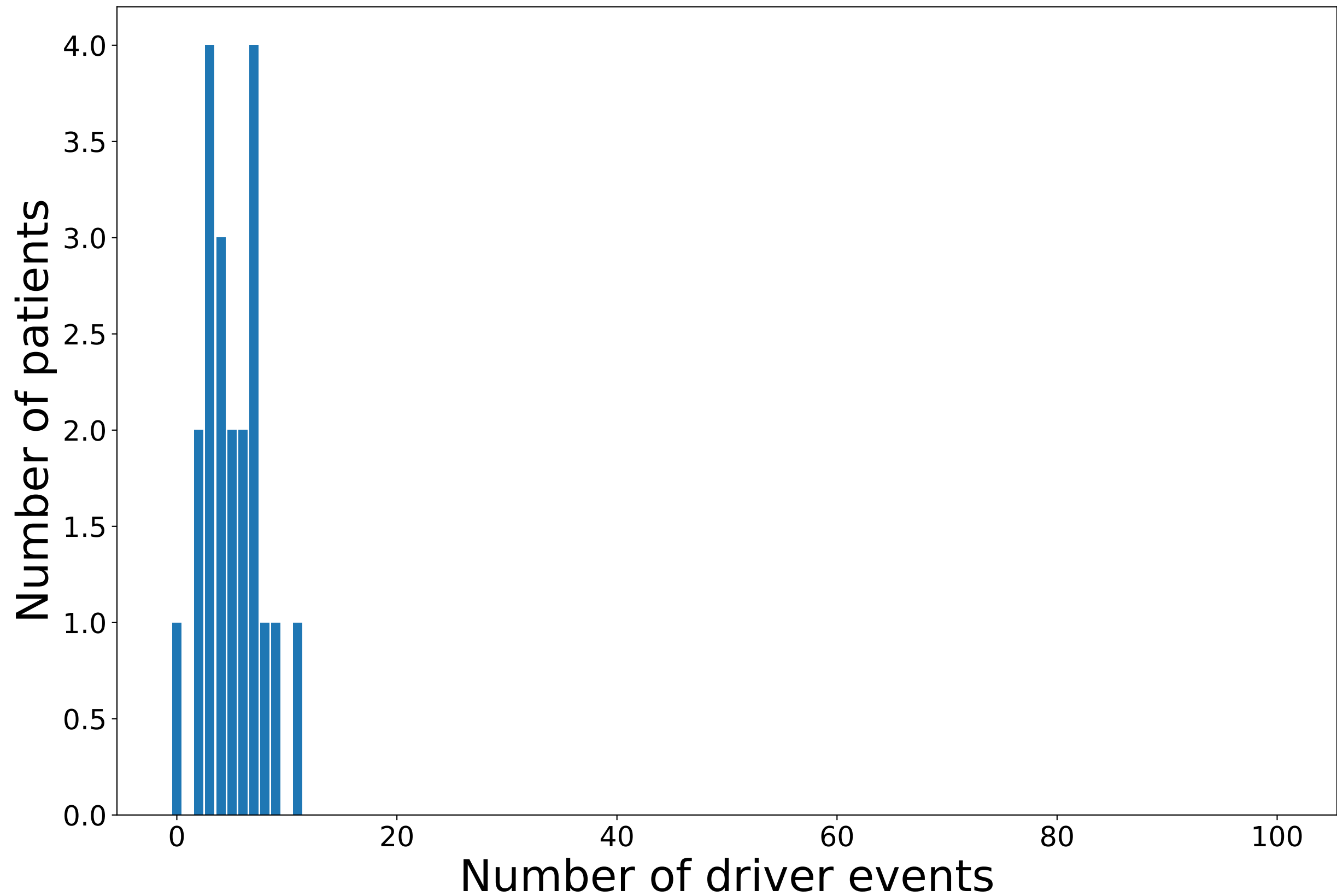

Supplement: S3 Files — (ZIP) [file pgen.1009996.s003.zip › COHORTS/patient distributions/2021_11_23_14_20_PCPG_MALE.pdf]

# THCA\_MALE

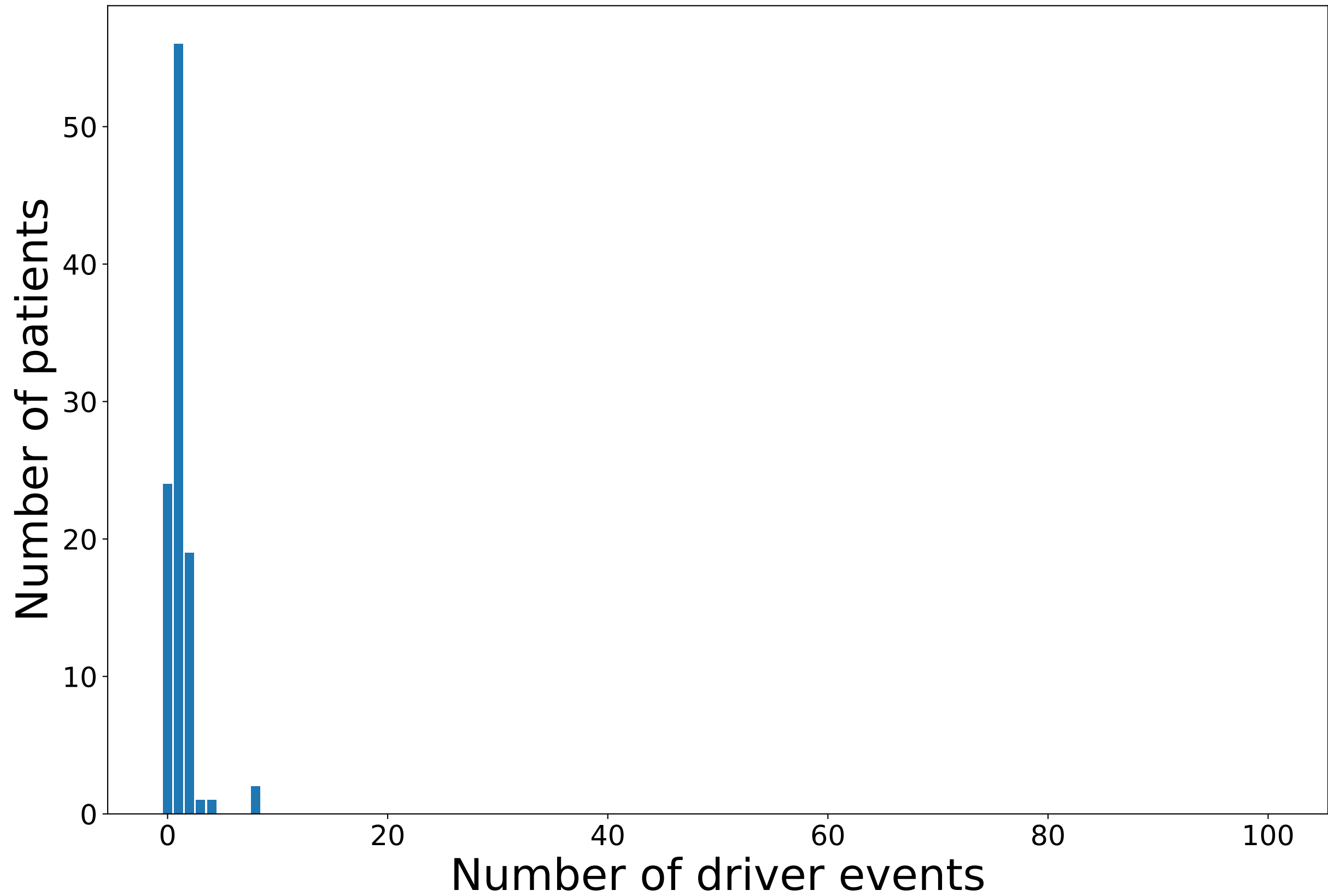

Supplement: S3 Files — (ZIP) [file pgen.1009996.s003.zip › COHORTS/patient distributions/2021_11_23_14_20_THCA_MALE.pdf]

# READ\_MALE

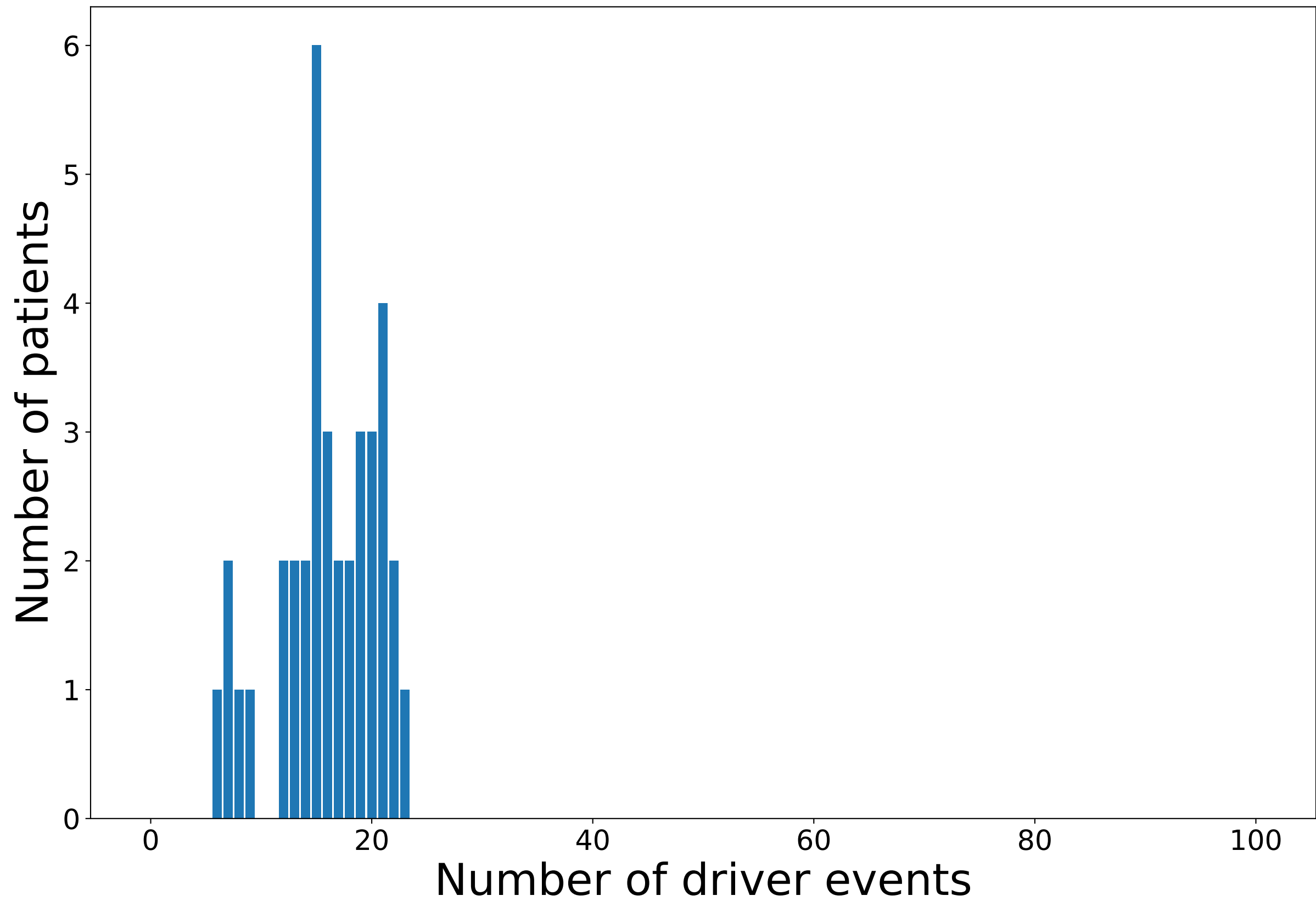

Supplement: S3 Files — (ZIP) [file pgen.1009996.s003.zip › COHORTS/patient distributions/2021_11_23_14_20_READ_MALE.pdf]

# LGG

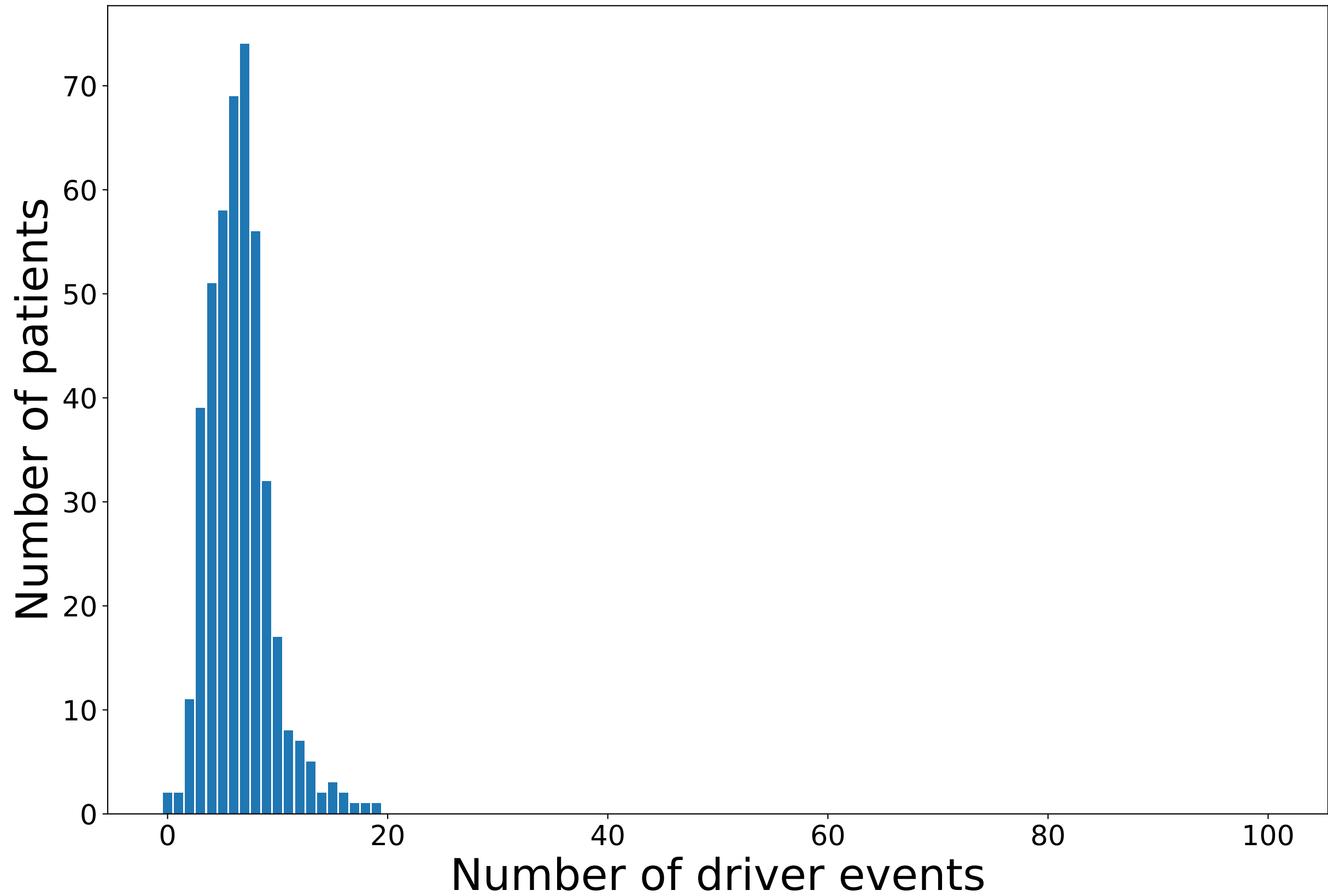

Supplement: S3 Files — (ZIP) [file pgen.1009996.s003.zip › COHORTS/patient distributions/2021_11_23_14_20_LGG.pdf]

# KICH\_MALE

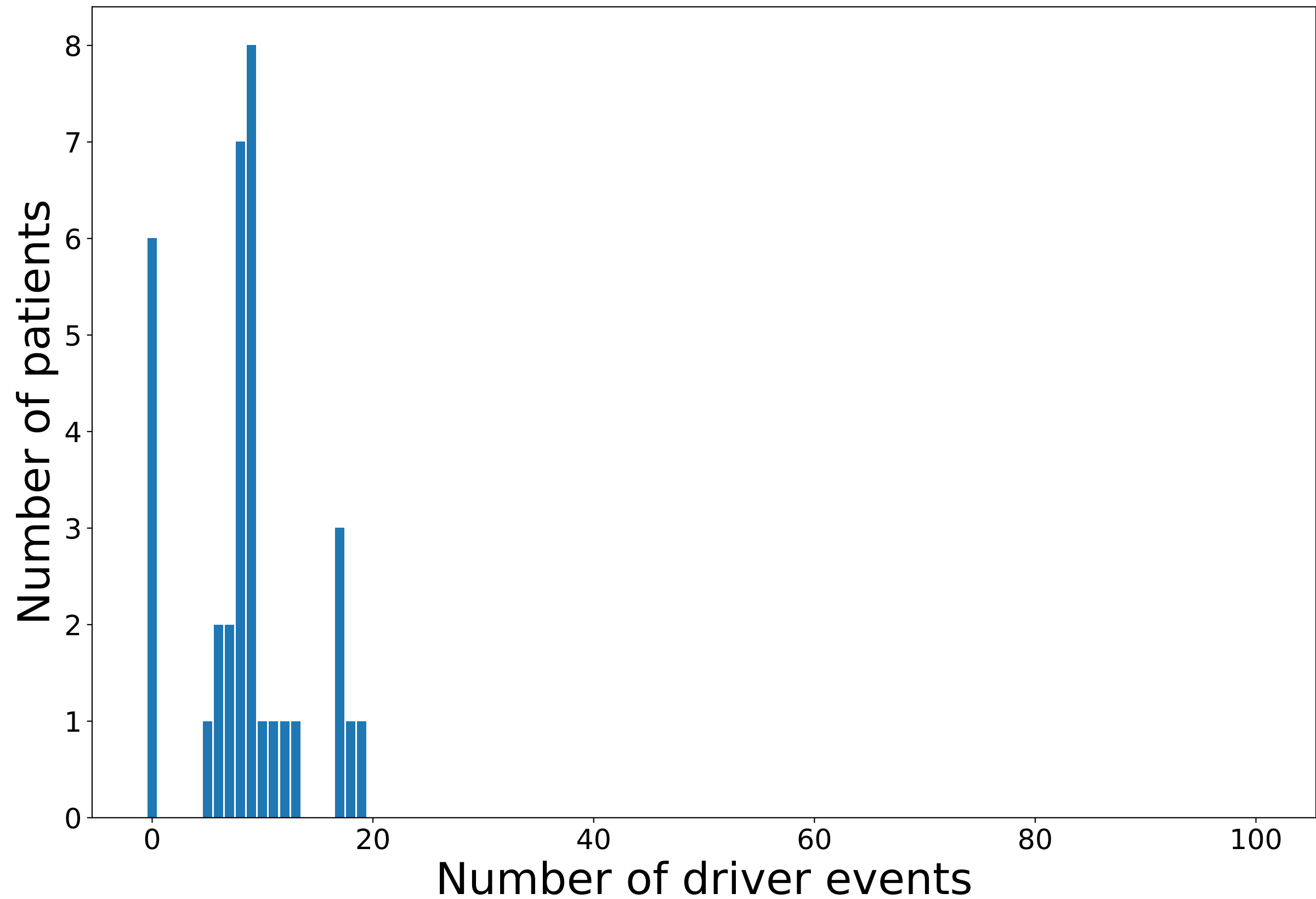

Supplement: S3 Files — (ZIP) [file pgen.1009996.s003.zip › COHORTS/patient distributions/2021_11_23_14_20_KICH_MALE.pdf]

# UCS\_FEMALE

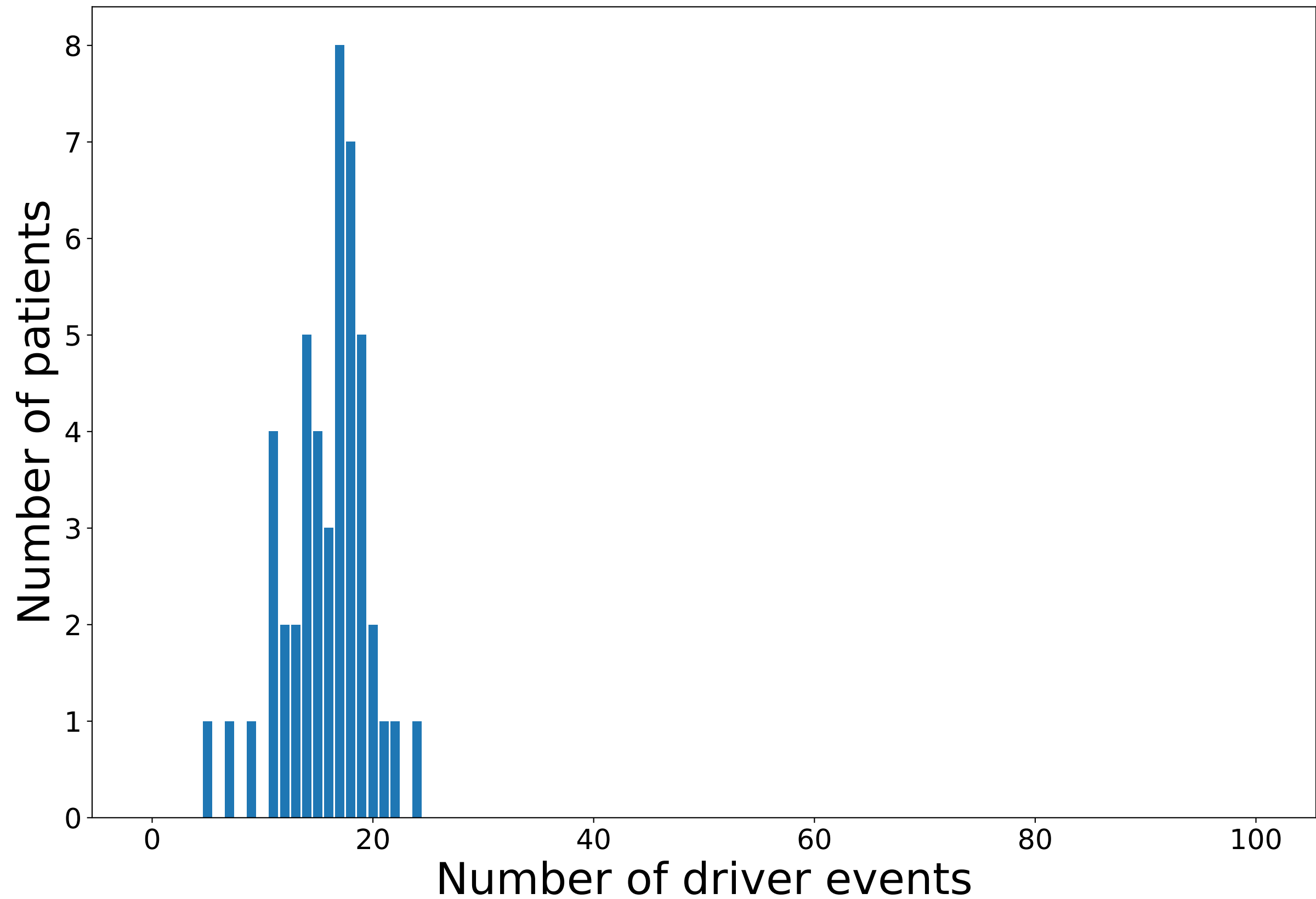

Supplement: S3 Files — (ZIP) [file pgen.1009996.s003.zip › COHORTS/patient distributions/2021_11_23_14_20_UCS_FEMALE.pdf]

ACC

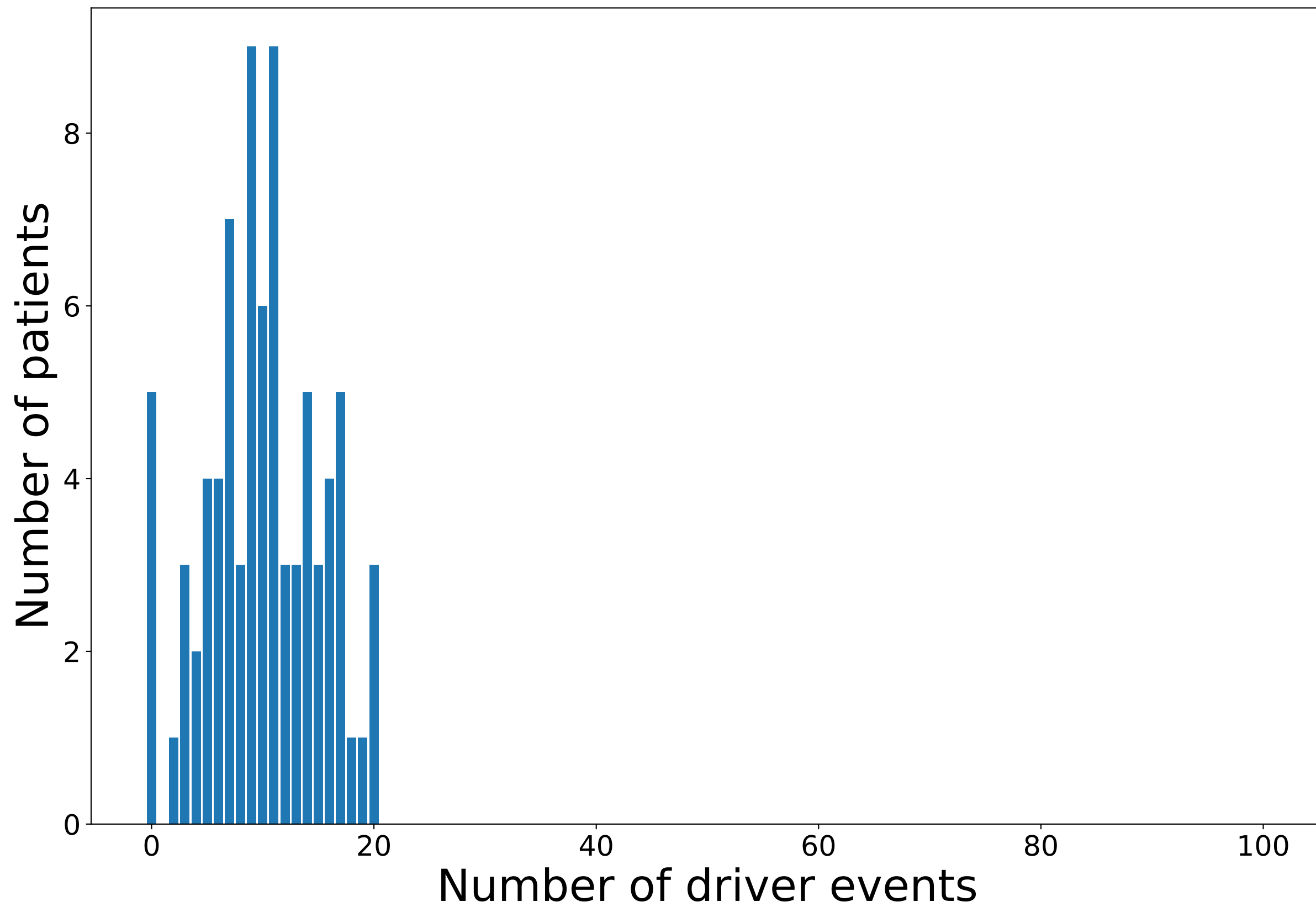

Supplement: S3 Files — (ZIP) [file pgen.1009996.s003.zip › COHORTS/patient distributions/2021_11_23_14_20_ACC.pdf]

# LUSC\_FEMALE

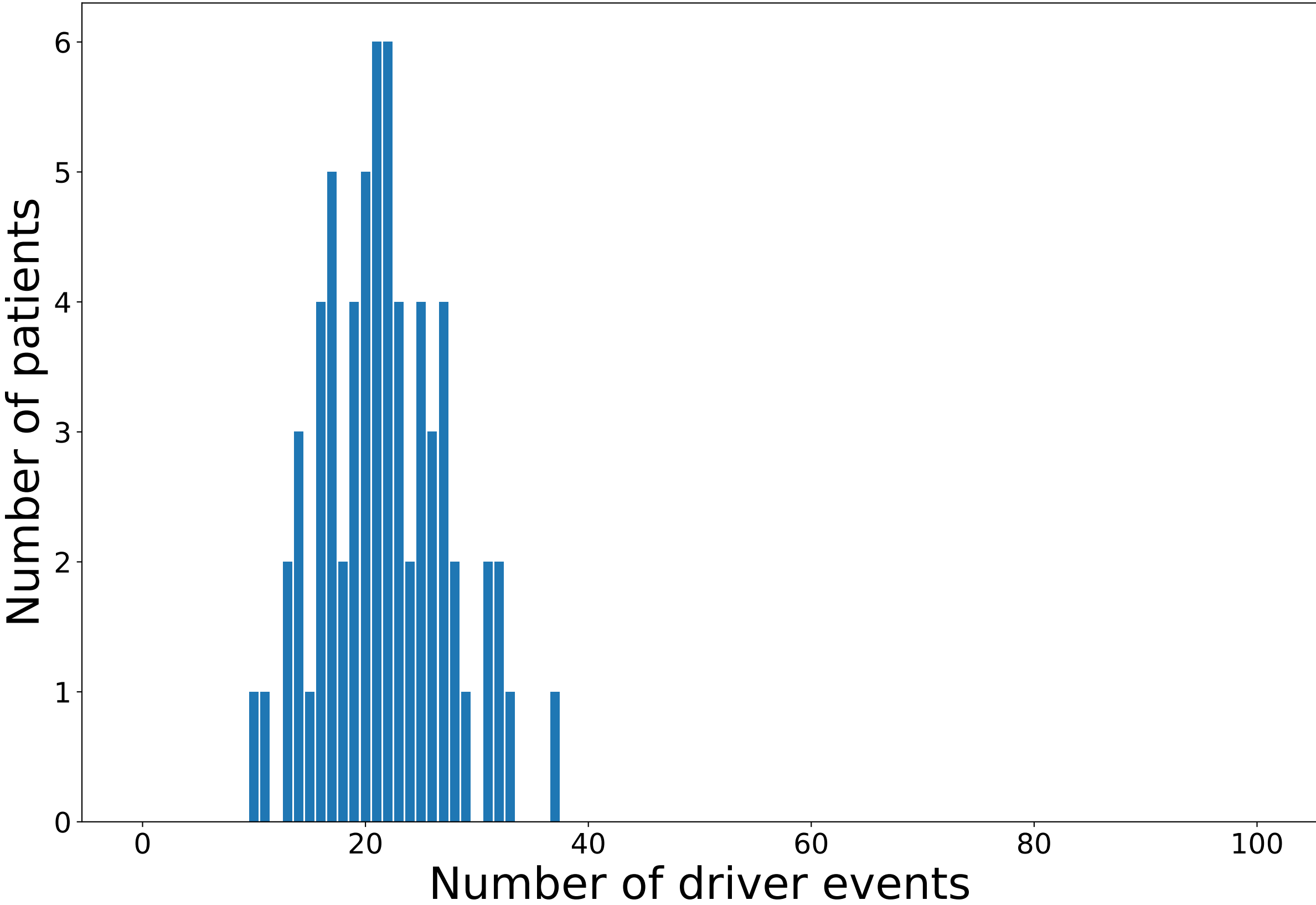

Supplement: S3 Files — (ZIP) [file pgen.1009996.s003.zip › COHORTS/patient distributions/2021_11_23_14_20_LUSC_FEMALE.pdf]

# THYM\_FEMALE

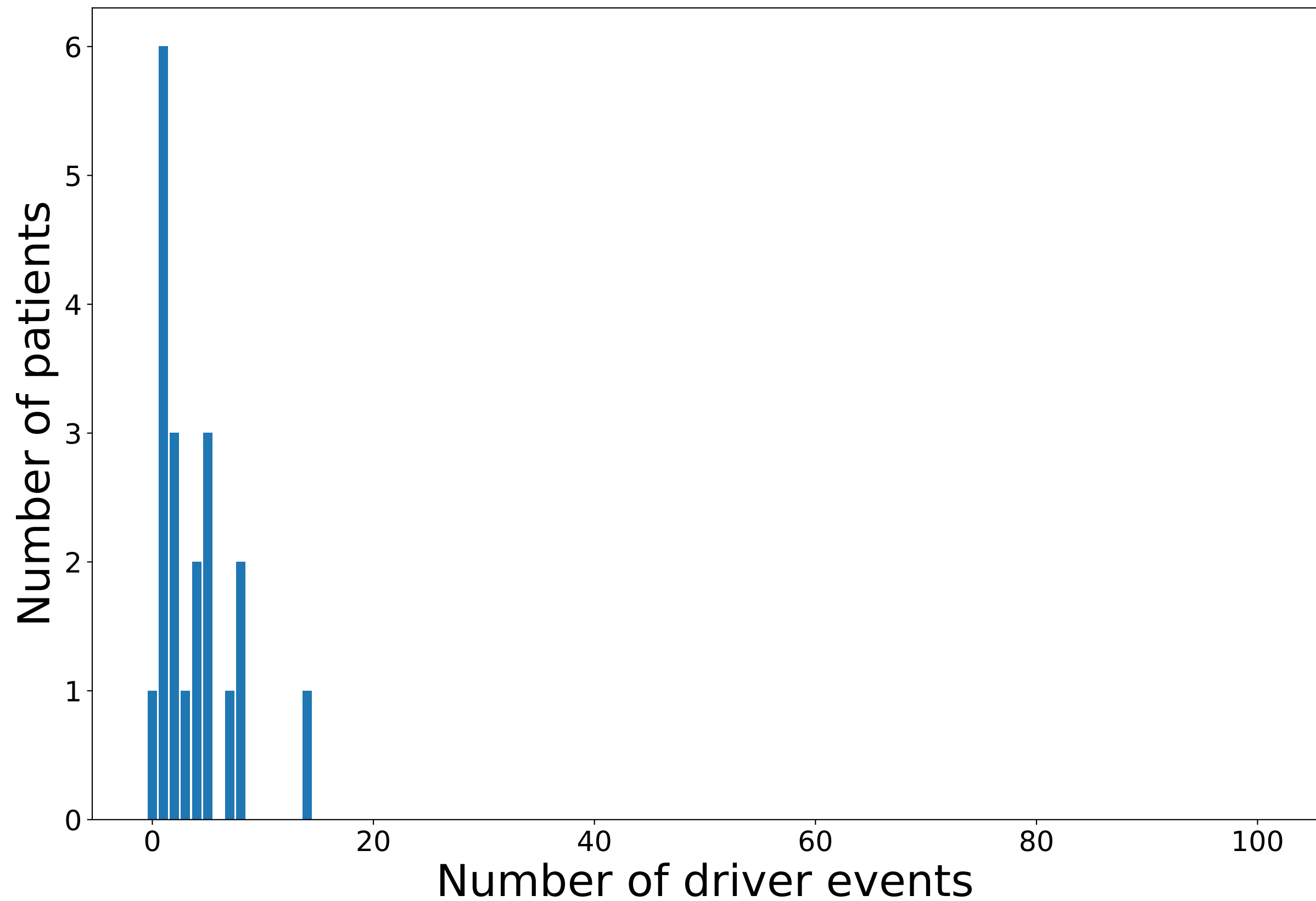

Supplement: S3 Files — (ZIP) [file pgen.1009996.s003.zip › COHORTS/patient distributions/2021_11_23_14_20_THYM_FEMALE.pdf]

# LUAD

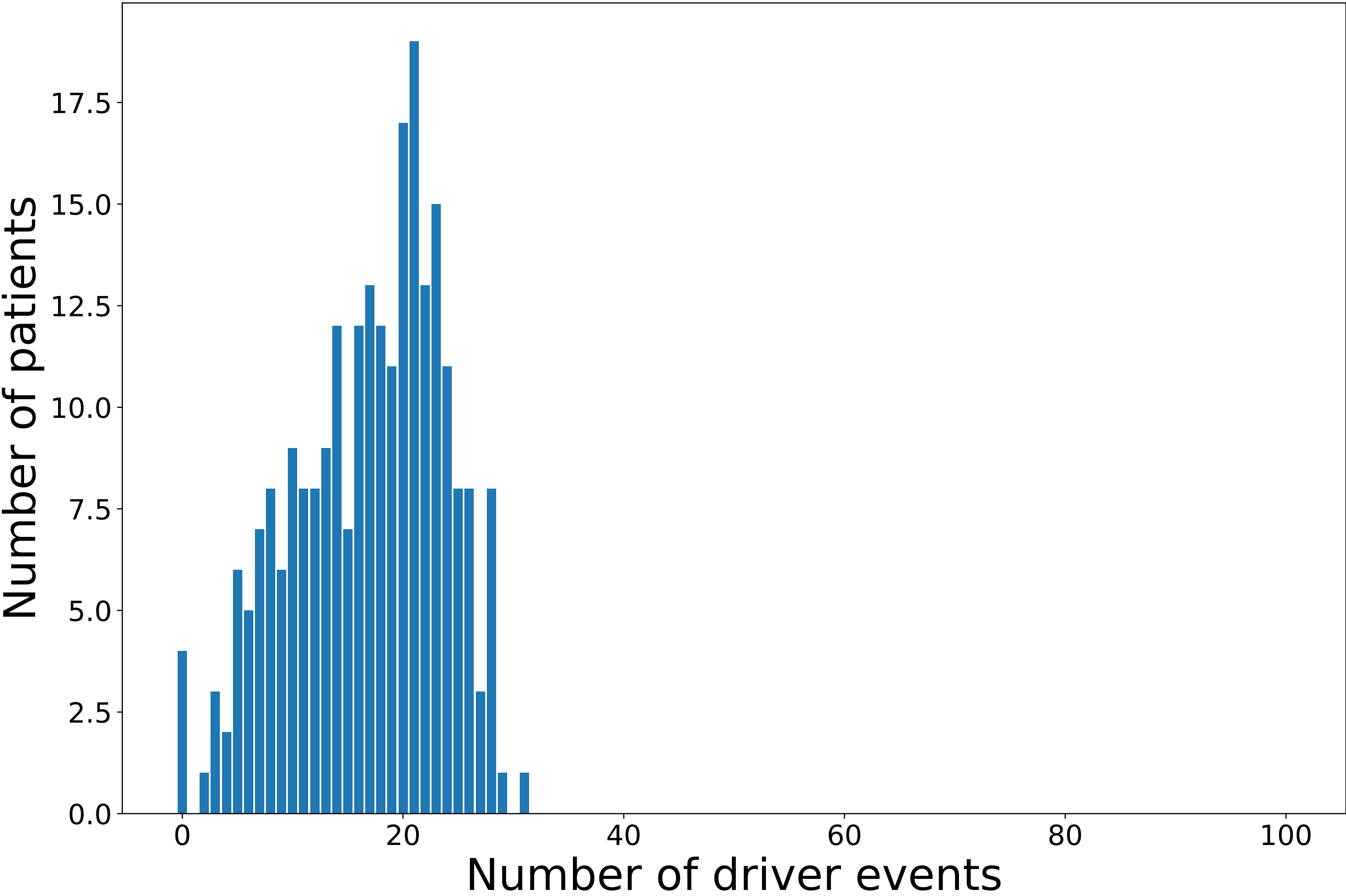

Supplement: S3 Files — (ZIP) [file pgen.1009996.s003.zip › COHORTS/patient distributions/2021_11_23_14_20_LUAD.pdf]

# DLBC\_MALE

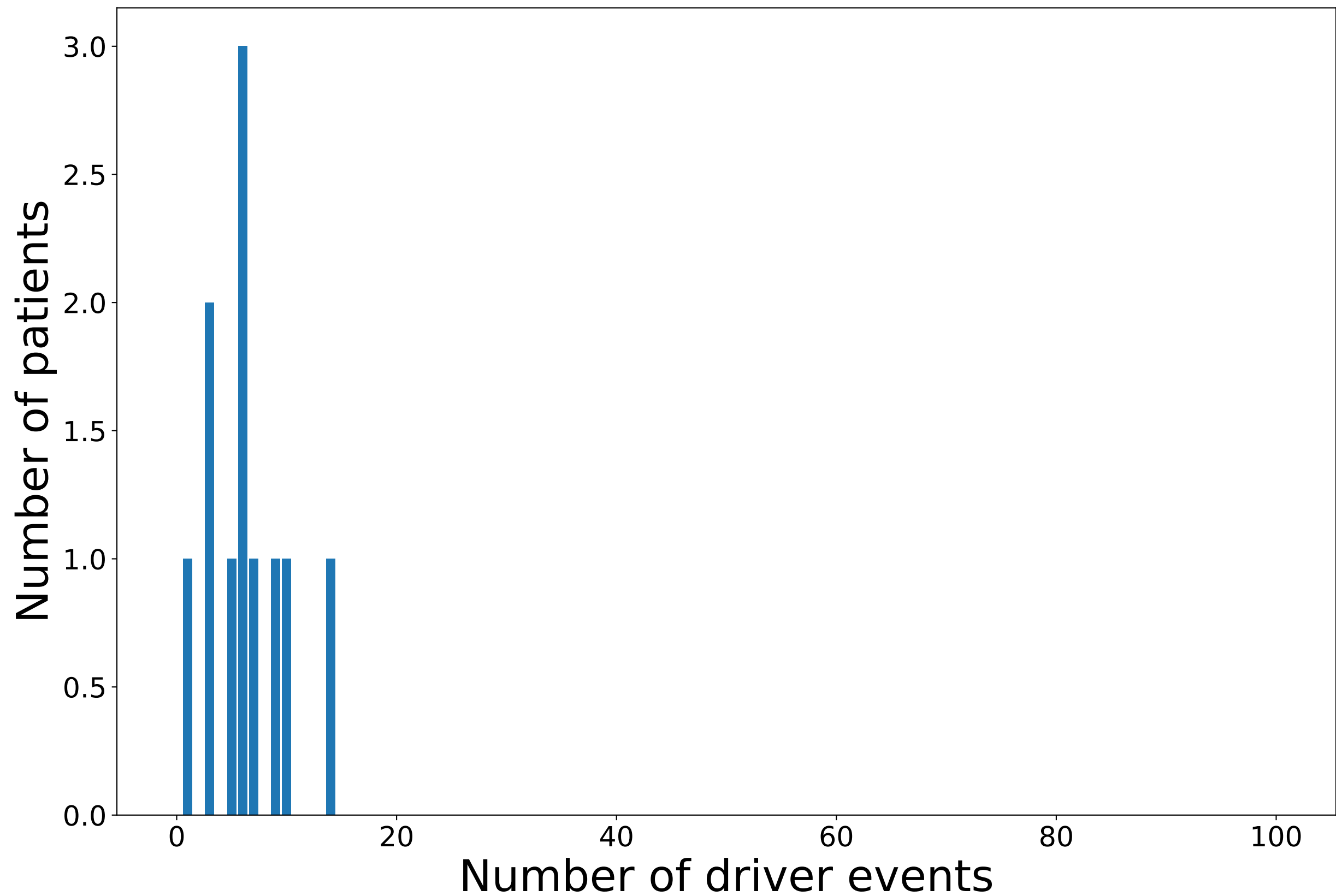

Supplement: S3 Files — (ZIP) [file pgen.1009996.s003.zip › COHORTS/patient distributions/2021_11_23_14_20_DLBC_MALE.pdf]

# SARC

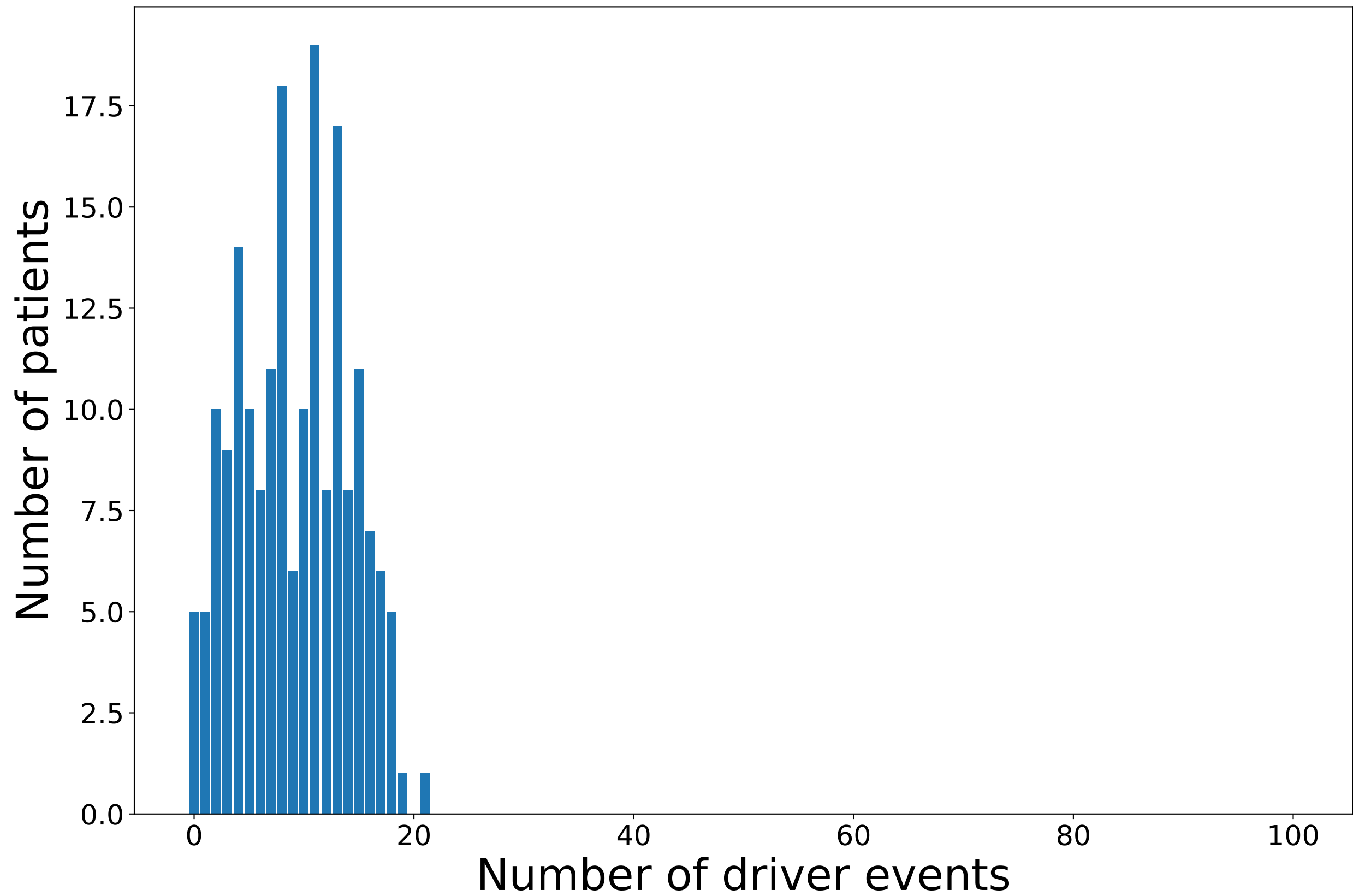

Supplement: S3 Files — (ZIP) [file pgen.1009996.s003.zip › COHORTS/patient distributions/2021_11_23_14_20_SARC.pdf]

# ACC\_MALE

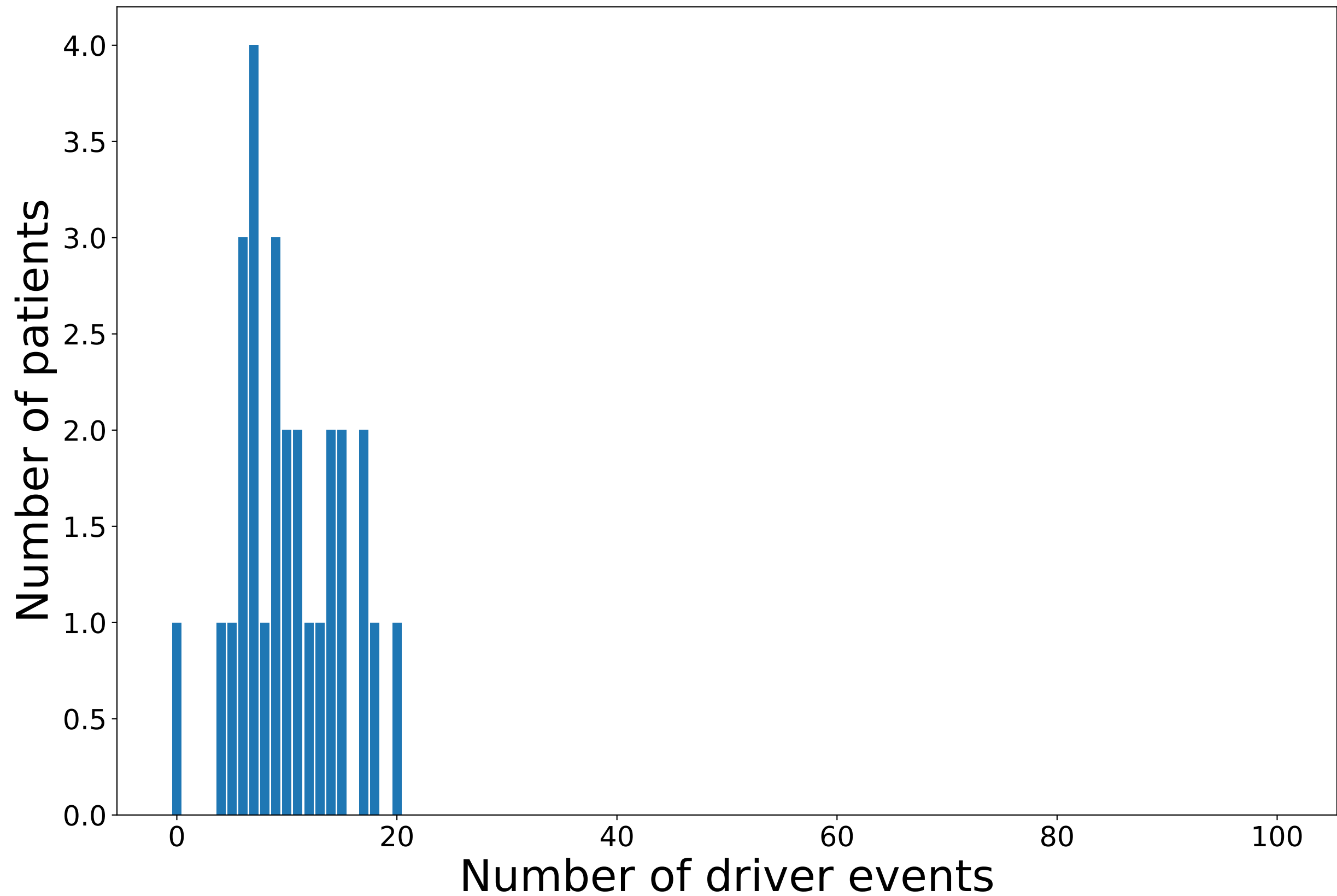

Supplement: S3 Files — (ZIP) [file pgen.1009996.s003.zip › COHORTS/patient distributions/2021_11_23_14_20_ACC_MALE.pdf]

PRAD

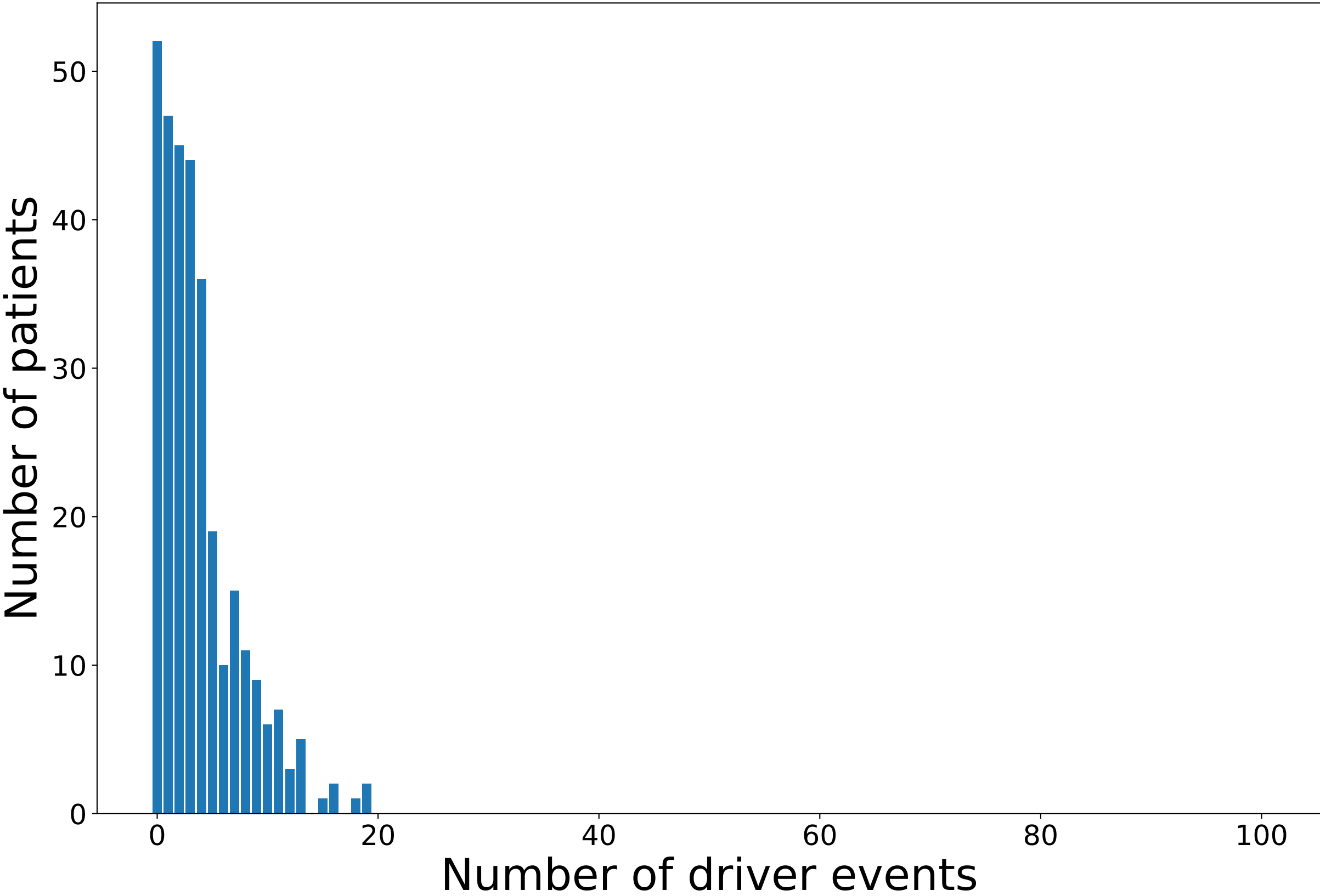

Supplement: S3 Files — (ZIP) [file pgen.1009996.s003.zip › COHORTS/patient distributions/2021_11_23_14_20_PRAD.pdf]

# ESCA\_MALE

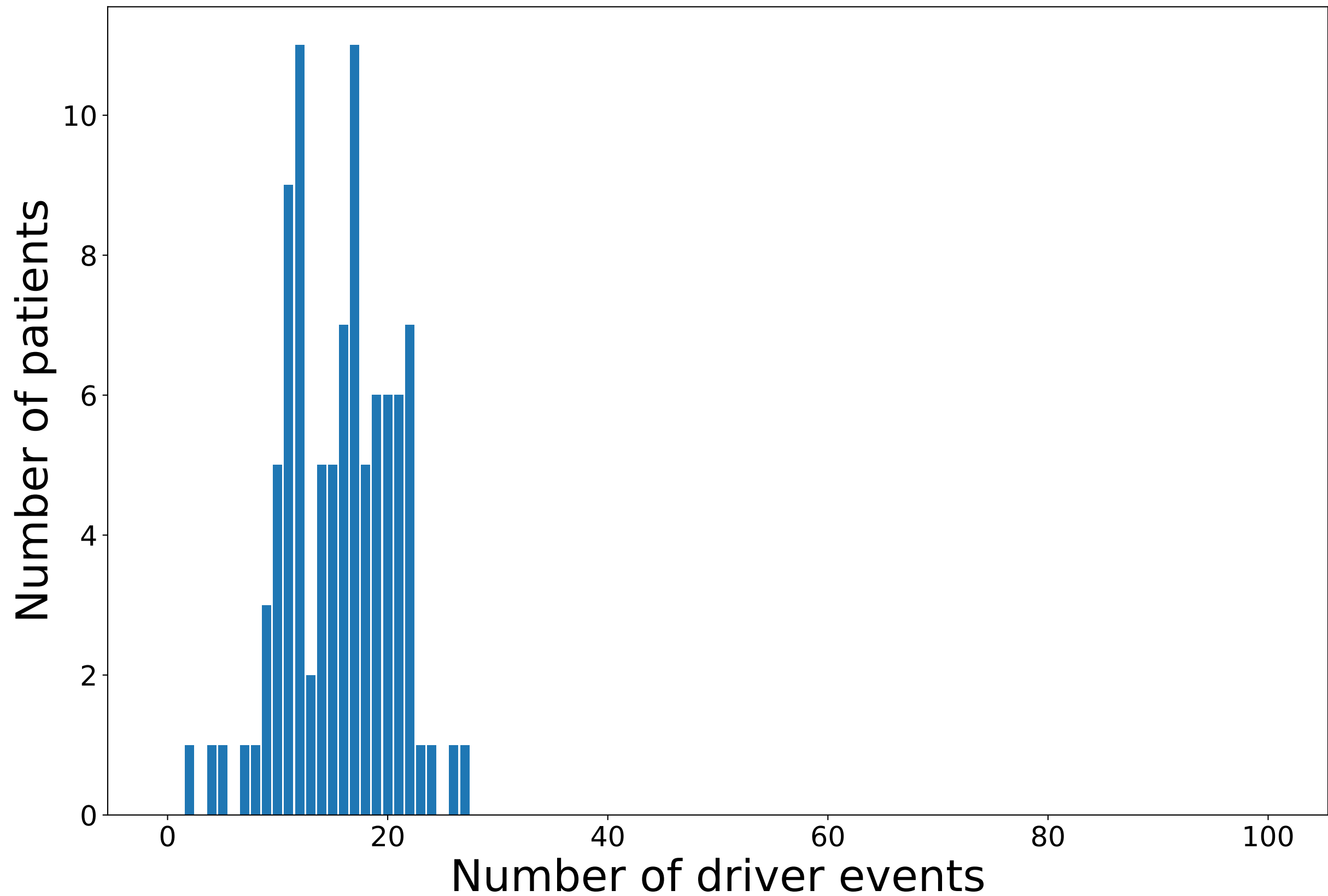

Supplement: S3 Files — (ZIP) [file pgen.1009996.s003.zip › COHORTS/patient distributions/2021_11_23_14_20_ESCA_MALE.pdf]

# GBM\_FEMALE

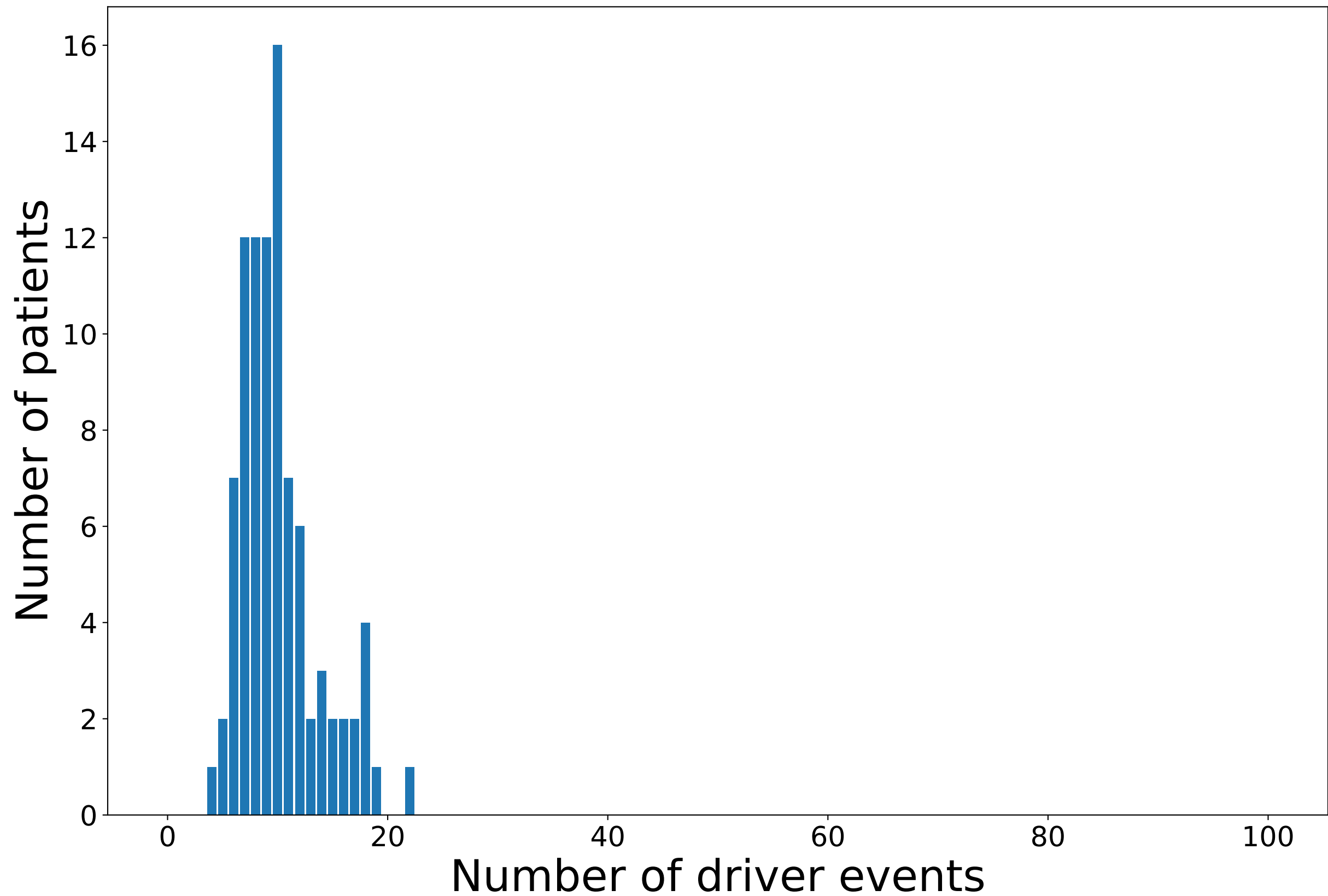

Supplement: S3 Files — (ZIP) [file pgen.1009996.s003.zip › COHORTS/patient distributions/2021_11_23_14_20_GBM_FEMALE.pdf]

# LIHC\_MALE

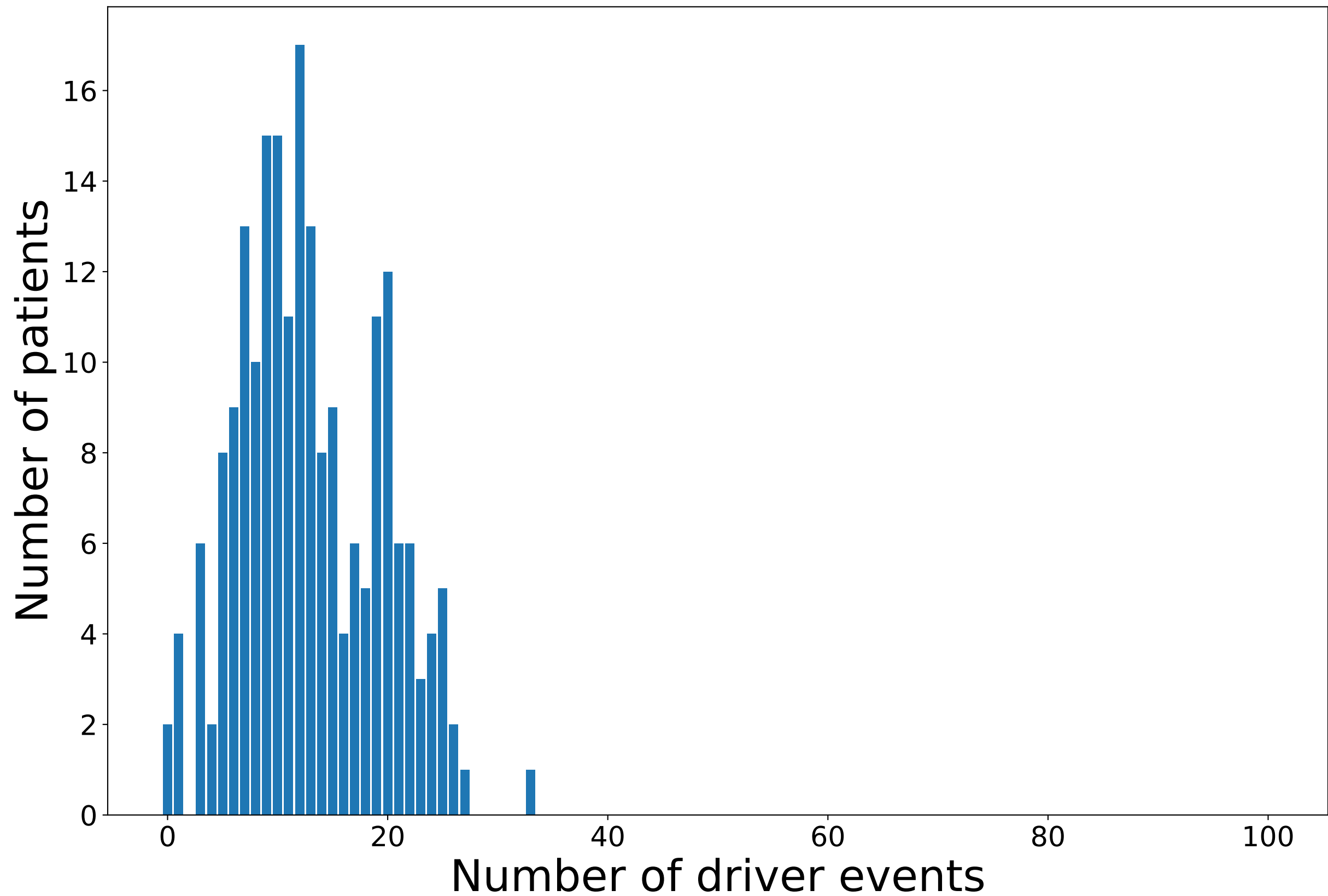

Supplement: S3 Files — (ZIP) [file pgen.1009996.s003.zip › COHORTS/patient distributions/2021_11_23_14_20_LIHC_MALE.pdf]

# MESO\_FEMALE

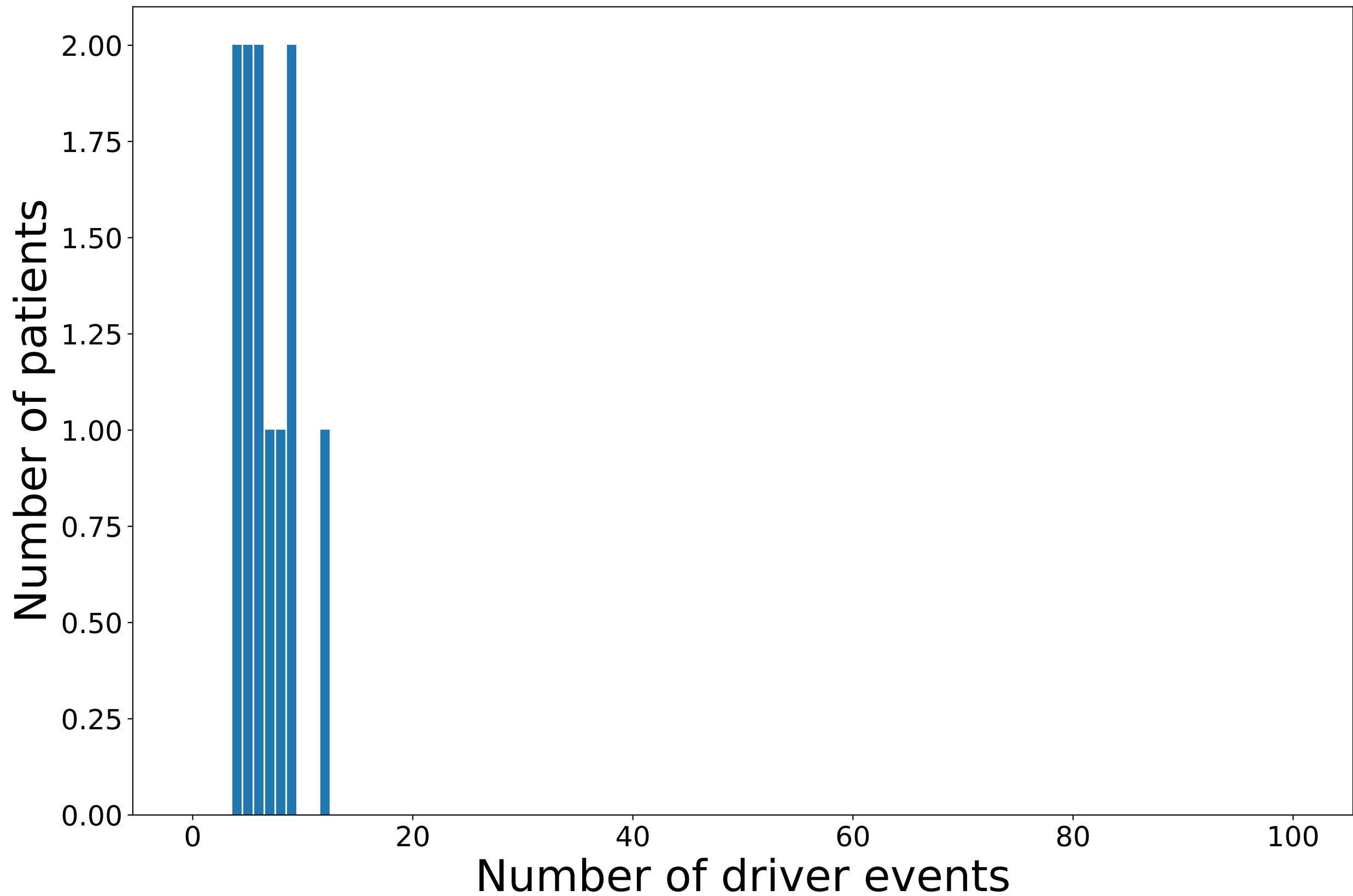

Supplement: S3 Files — (ZIP) [file pgen.1009996.s003.zip › COHORTS/patient distributions/2021_11_23_14_20_MESO_FEMALE.pdf]

# LGG\_FEMALE

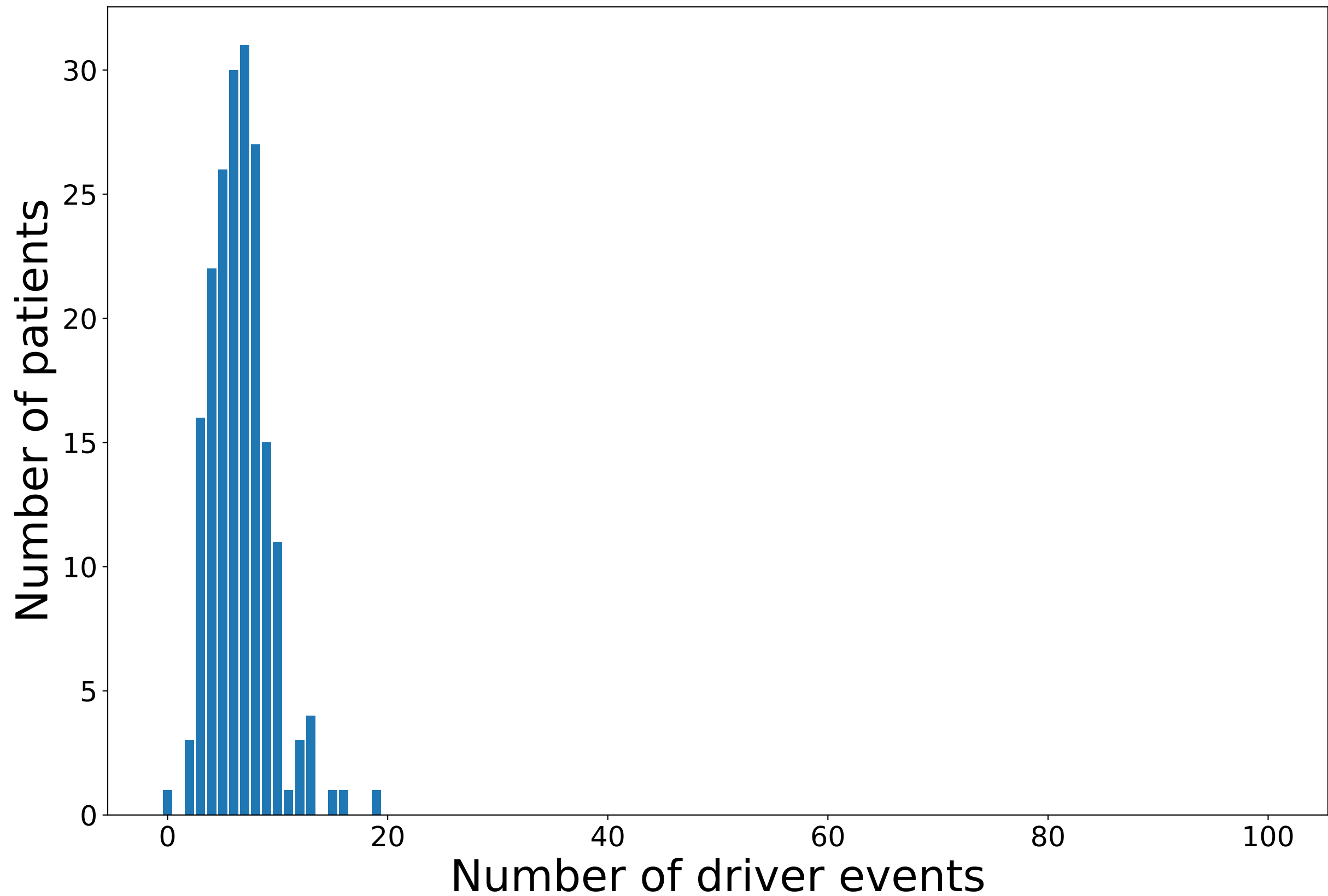

Supplement: S3 Files — (ZIP) [file pgen.1009996.s003.zip › COHORTS/patient distributions/2021_11_23_14_20_LGG_FEMALE.pdf]

# HNSC\_FEMALE

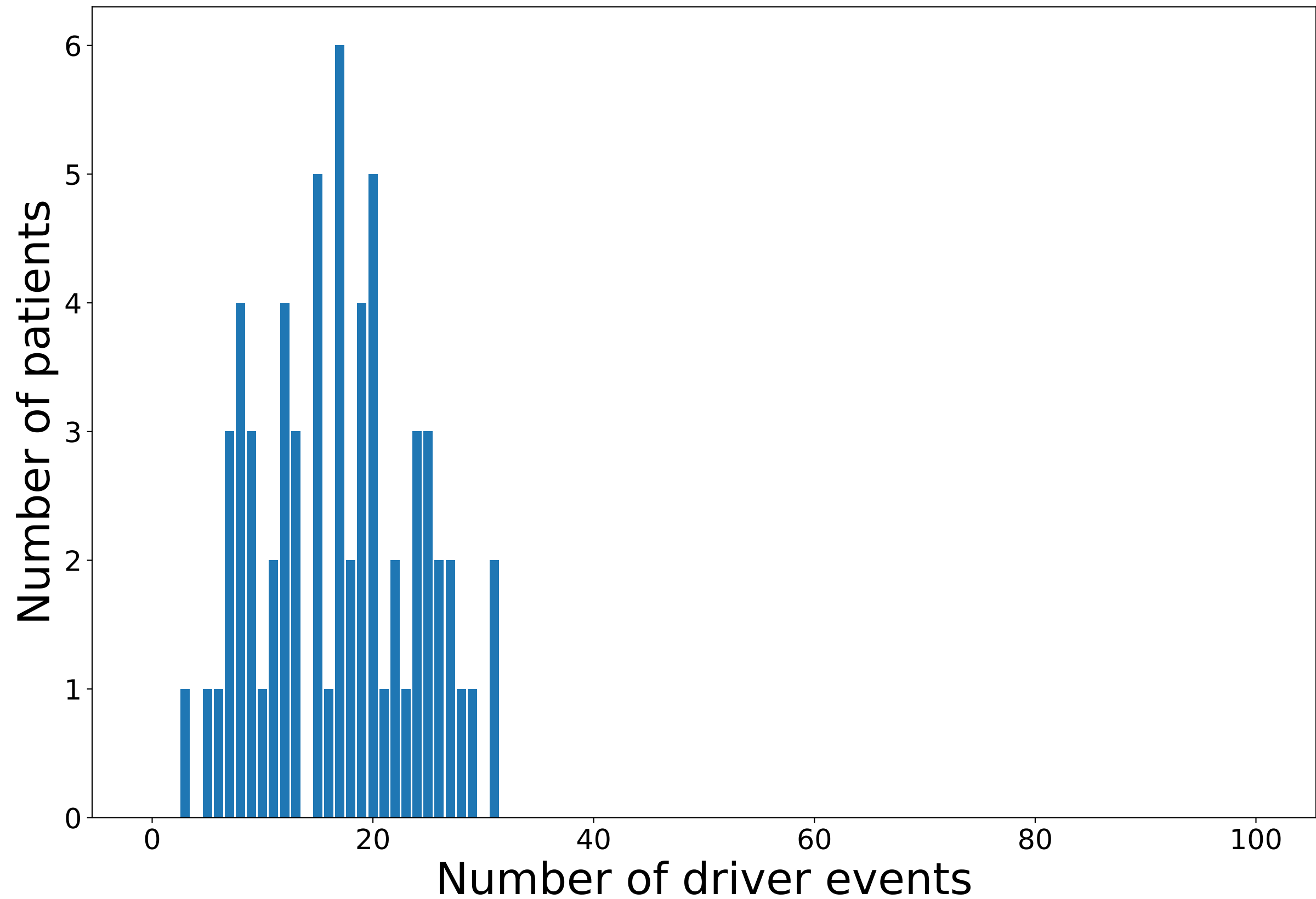

Supplement: S3 Files — (ZIP) [file pgen.1009996.s003.zip › COHORTS/patient distributions/2021_11_23_14_20_HNSC_FEMALE.pdf]

# LIHC

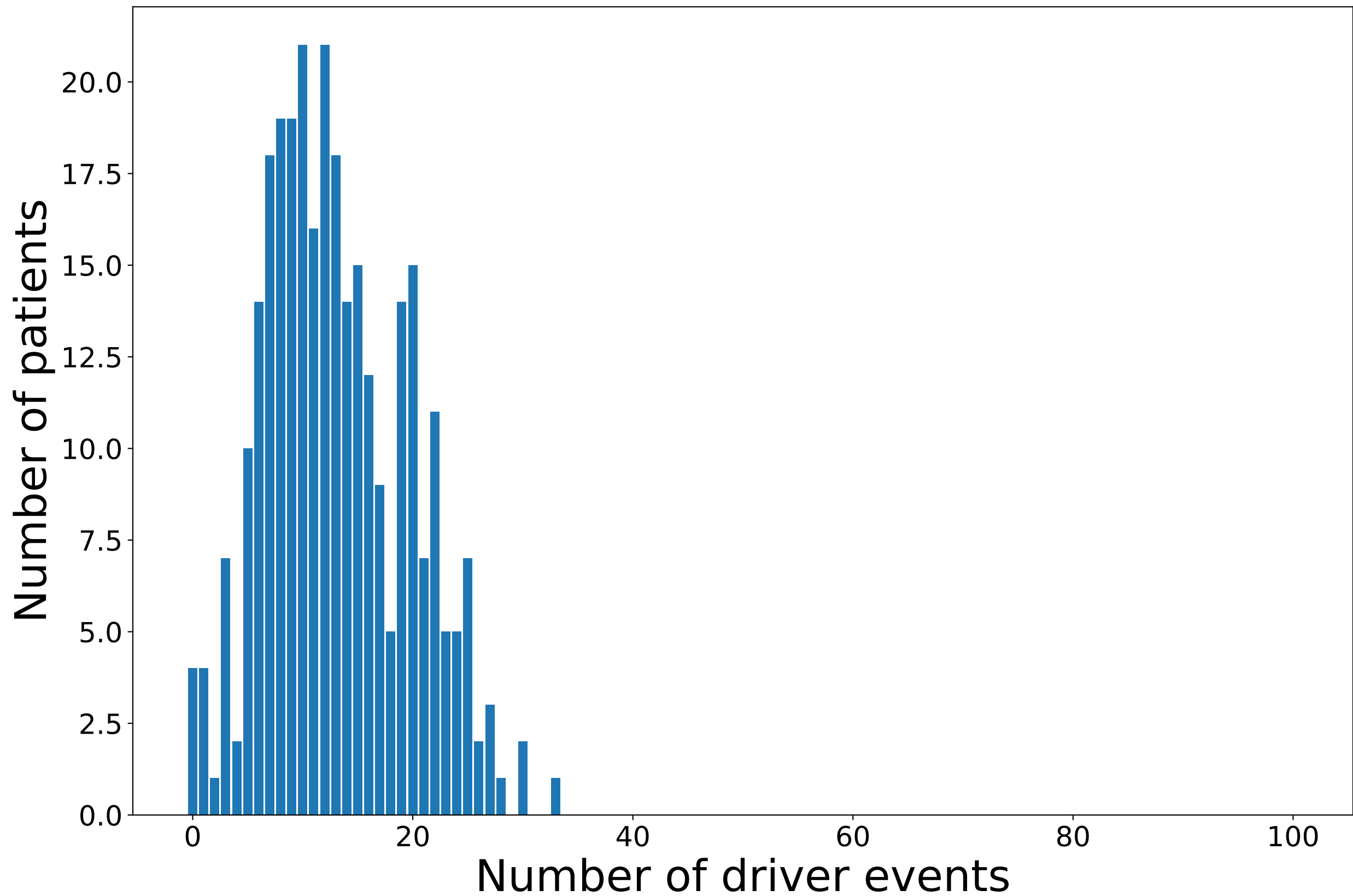

Supplement: S3 Files — (ZIP) [file pgen.1009996.s003.zip › COHORTS/patient distributions/2021_11_23_14_20_LIHC.pdf]

# PANCAN\_MALE

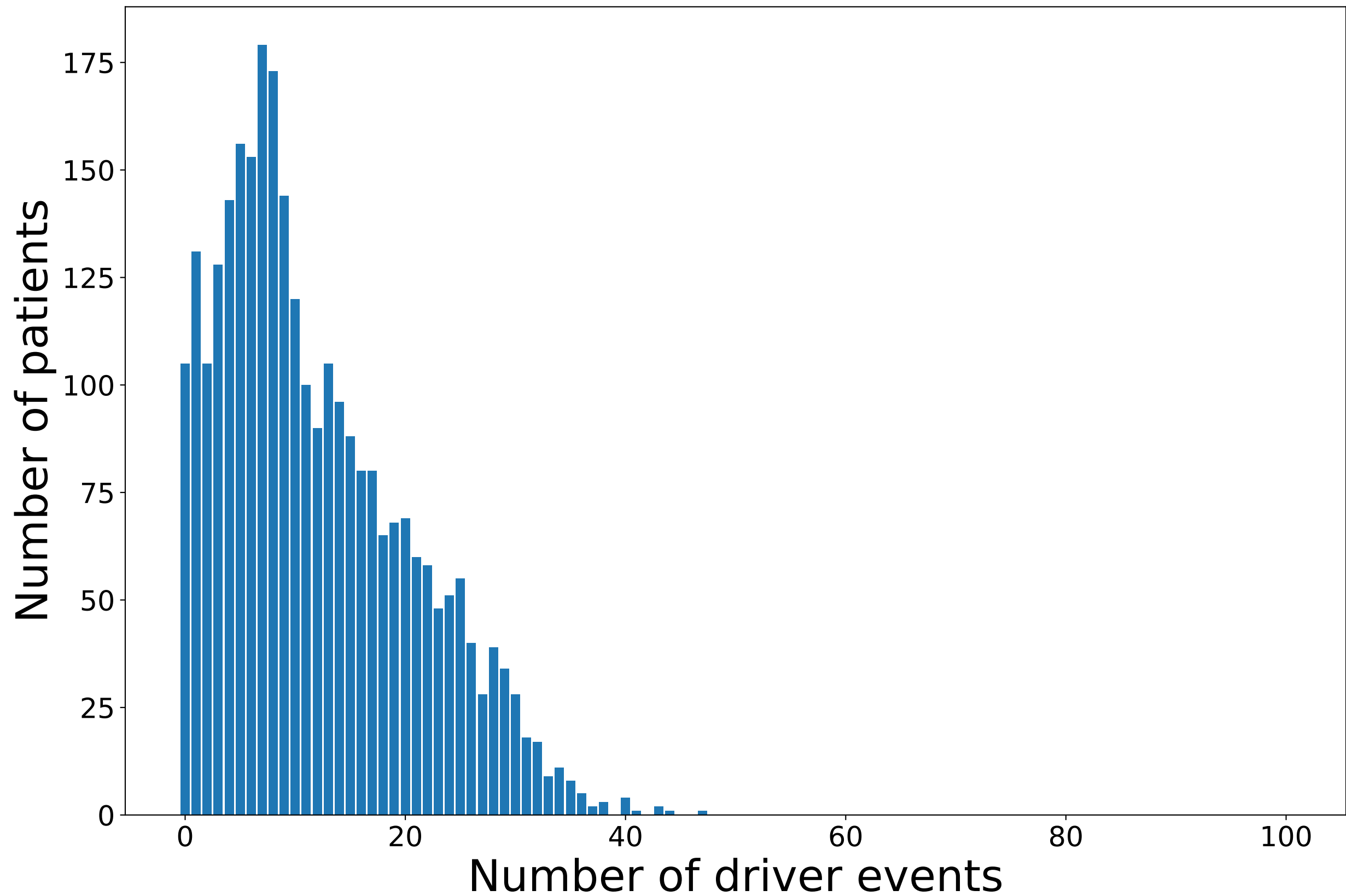

Supplement: S3 Files — (ZIP) [file pgen.1009996.s003.zip › COHORTS/patient distributions/2021_11_23_14_20_PANCAN_MALE.pdf]

# COAD

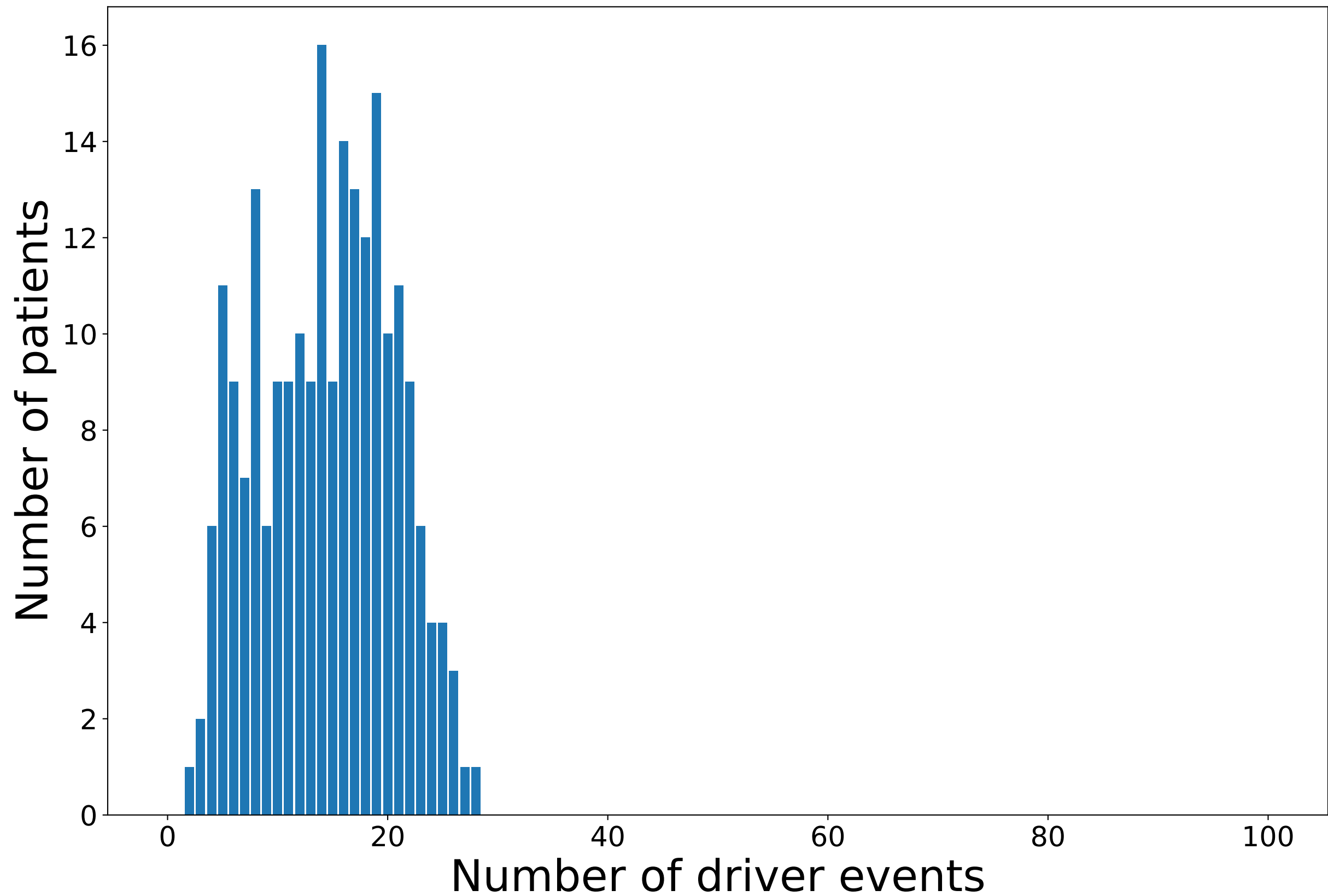

Supplement: S3 Files — (ZIP) [file pgen.1009996.s003.zip › COHORTS/patient distributions/2021_11_23_14_20_COAD.pdf]

# KIRP

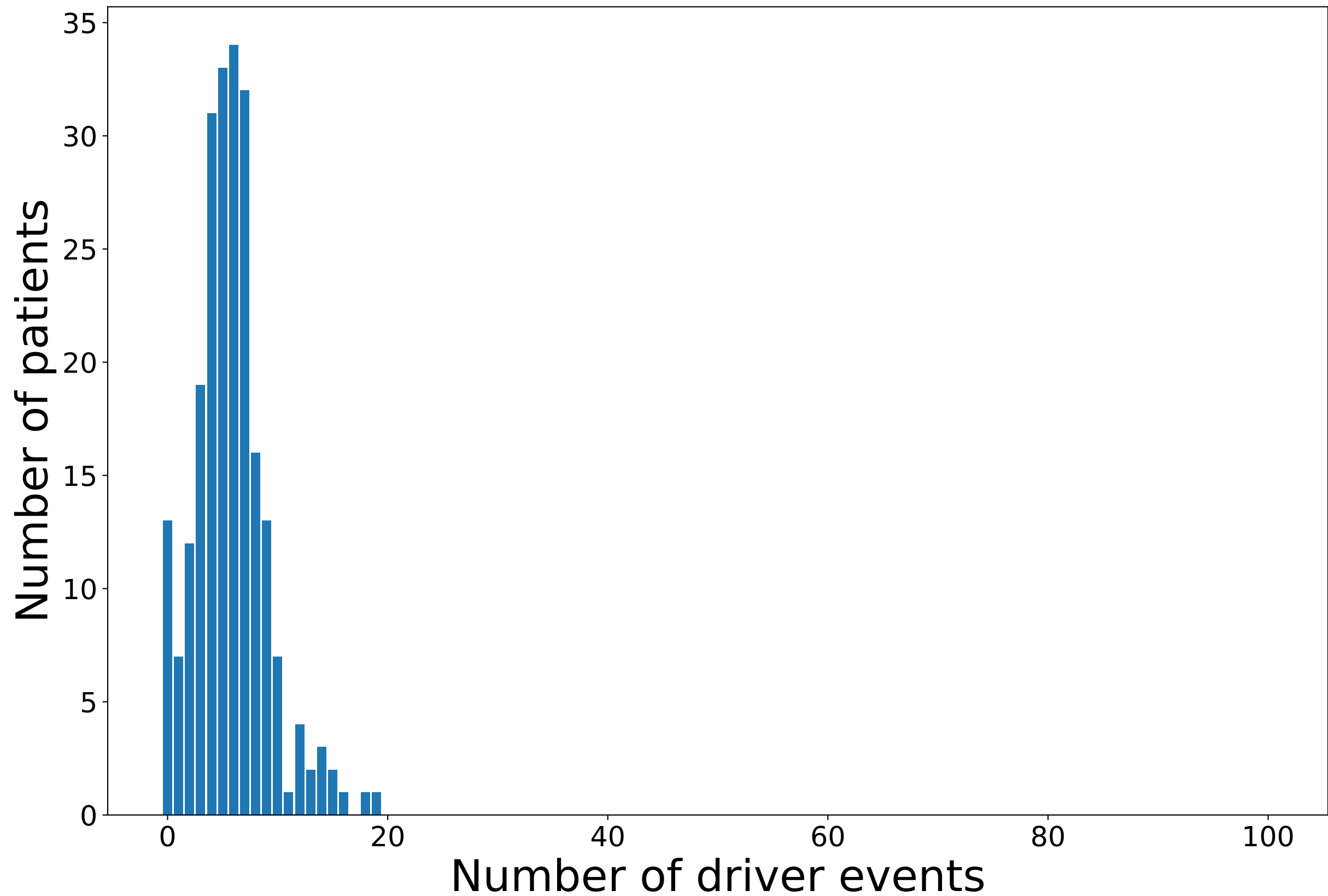

Supplement: S3 Files — (ZIP) [file pgen.1009996.s003.zip › COHORTS/patient distributions/2021_11_23_14_20_KIRP.pdf]

# OV\_FEMALE

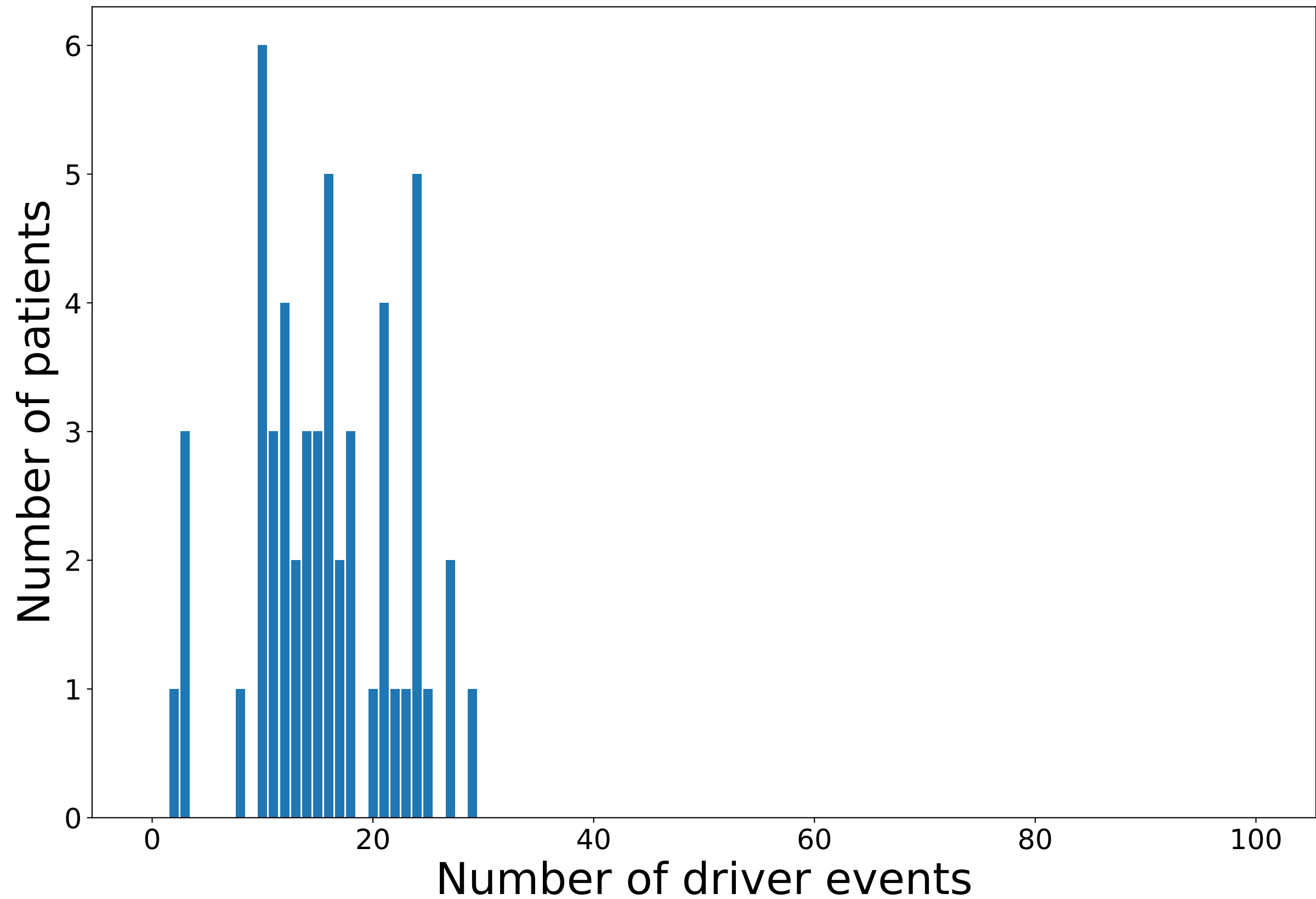

Supplement: S3 Files — (ZIP) [file pgen.1009996.s003.zip › COHORTS/patient distributions/2021_11_23_14_20_OV_FEMALE.pdf]

# PAAD\_FEMALE

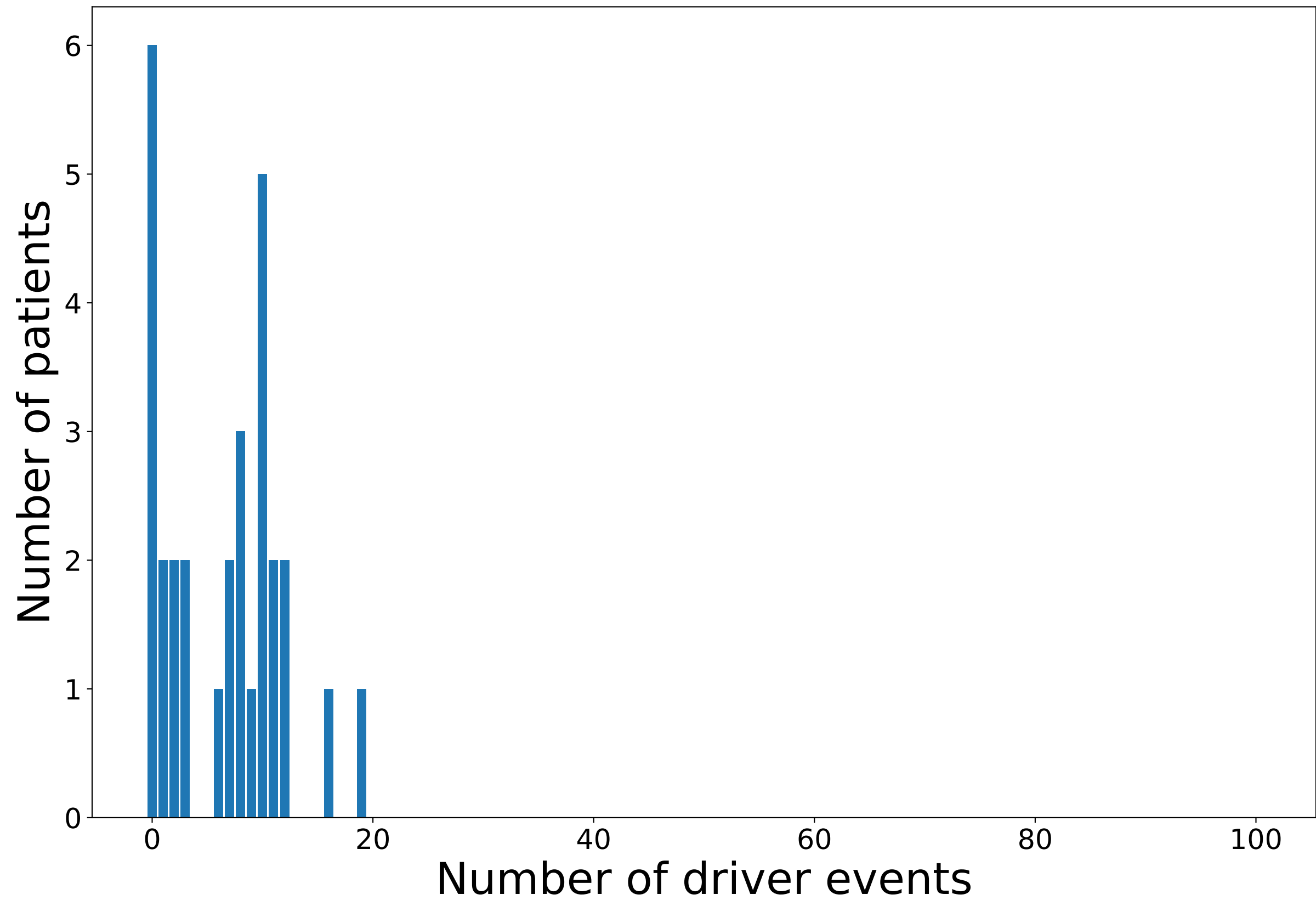

Supplement: S3 Files — (ZIP) [file pgen.1009996.s003.zip › COHORTS/patient distributions/2021_11_23_14_20_PAAD_FEMALE.pdf]

# HNSC\_MALE

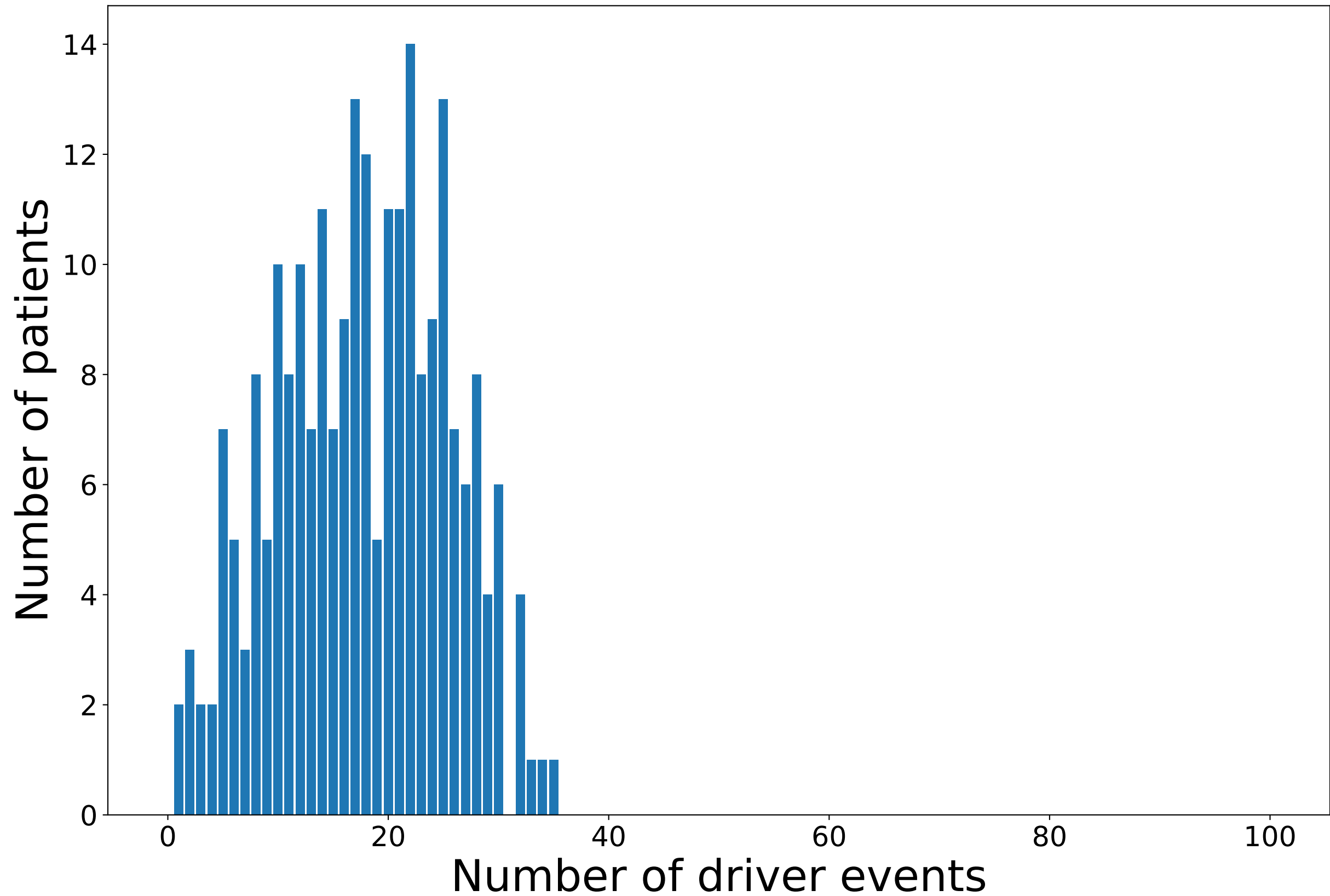

Supplement: S3 Files — (ZIP) [file pgen.1009996.s003.zip › COHORTS/patient distributions/2021_11_23_14_20_HNSC_MALE.pdf]

# KIRC

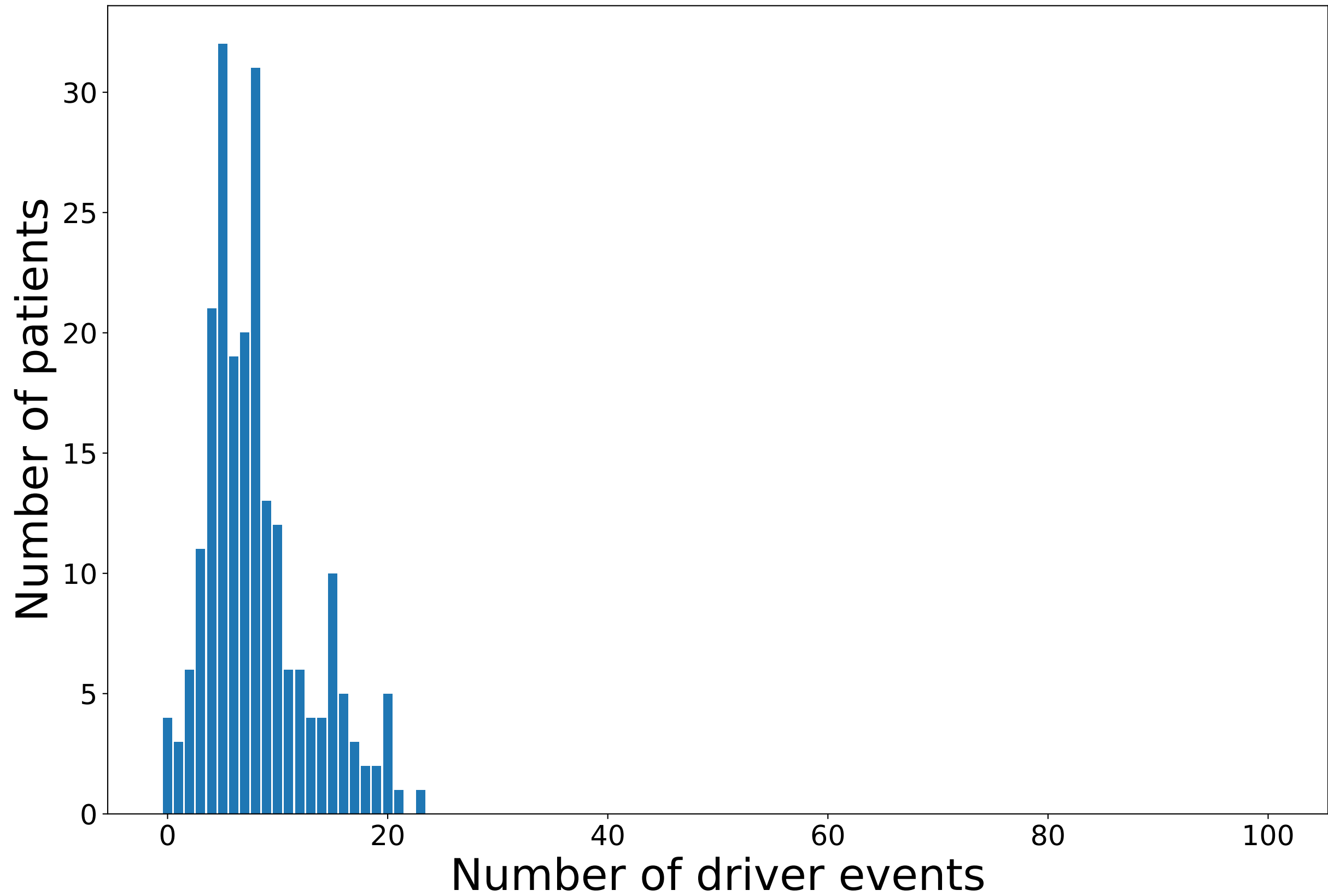

Supplement: S3 Files — (ZIP) [file pgen.1009996.s003.zip › COHORTS/patient distributions/2021_11_23_14_20_KIRC.pdf]

# SKCM\_FEMALE

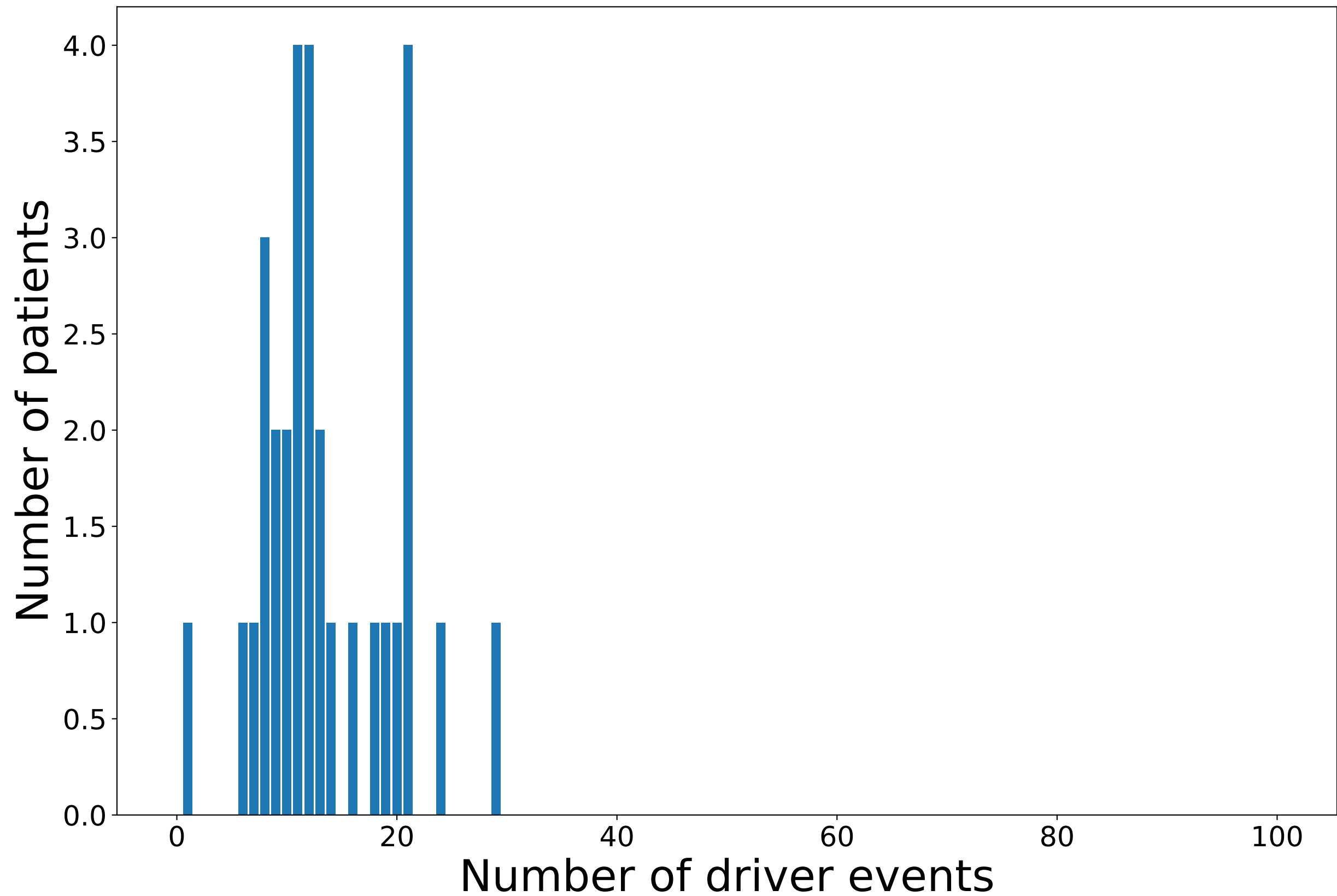

Supplement: S3 Files — (ZIP) [file pgen.1009996.s003.zip › COHORTS/patient distributions/2021_11_23_14_20_SKCM_FEMALE.pdf]

# THYM

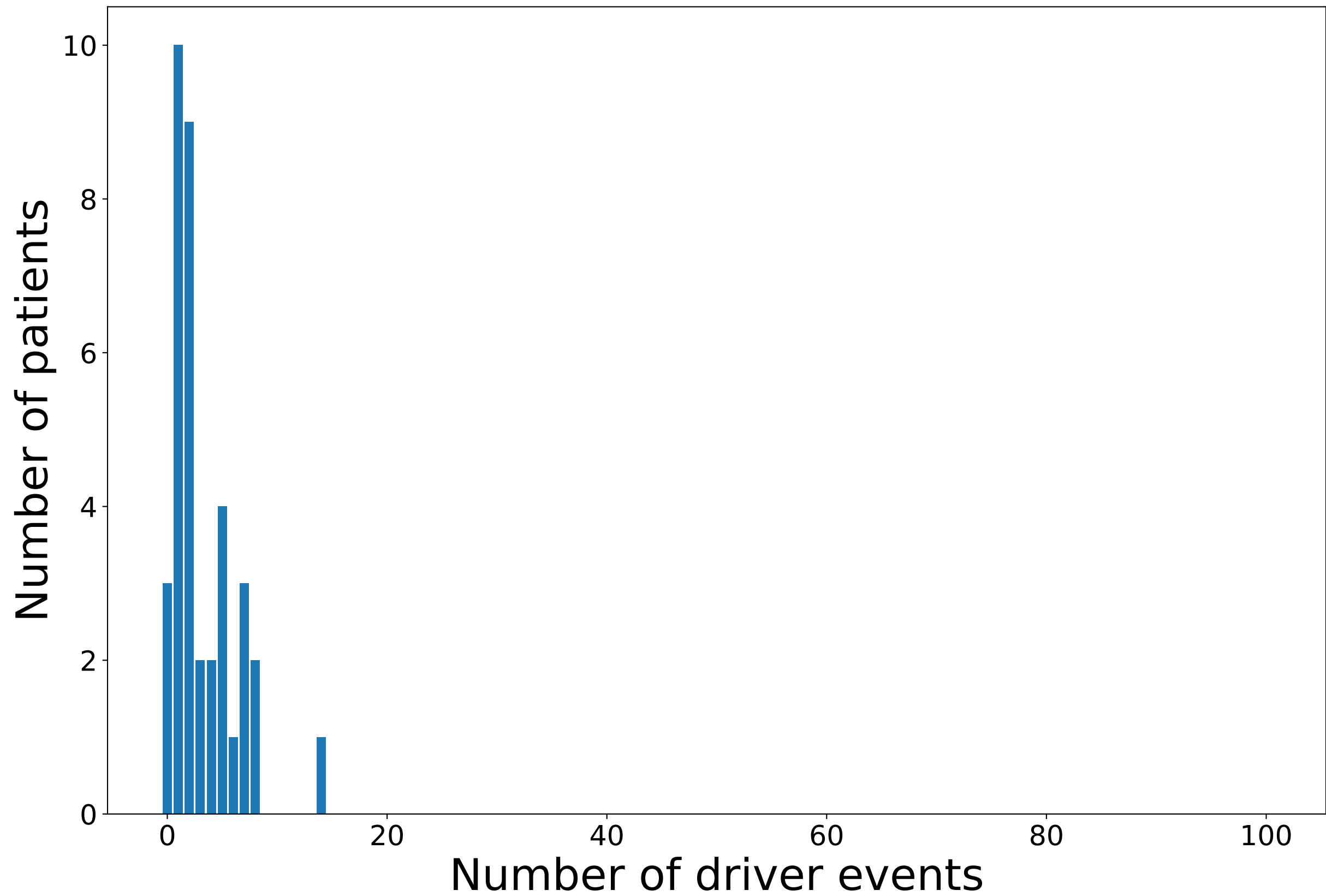

Supplement: S3 Files — (ZIP) [file pgen.1009996.s003.zip › COHORTS/patient distributions/2021_11_23_14_20_THYM.pdf]

# KIRP\_MALE

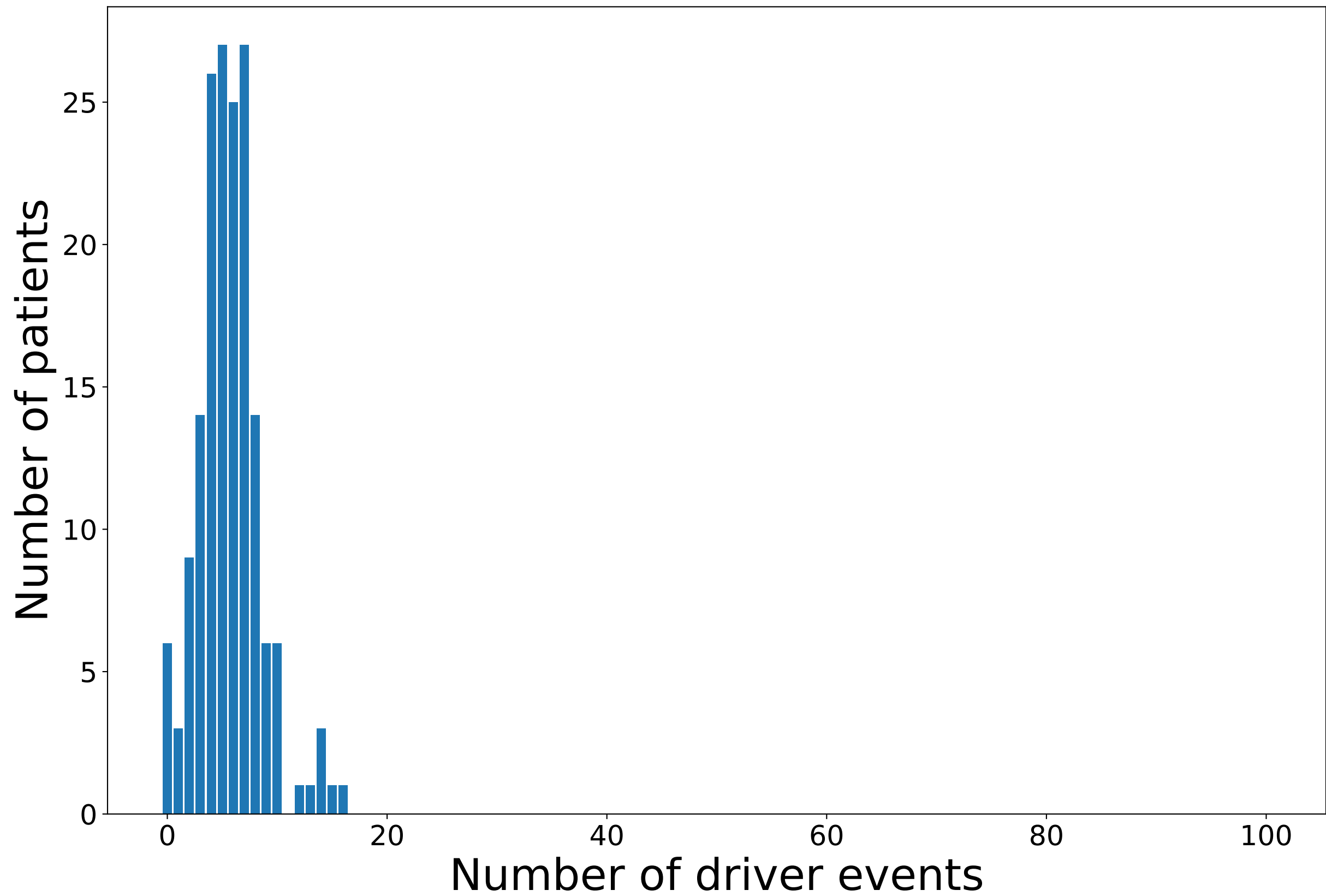

Supplement: S3 Files — (ZIP) [file pgen.1009996.s003.zip › COHORTS/patient distributions/2021_11_23_14_20_KIRP_MALE.pdf]

# GBM

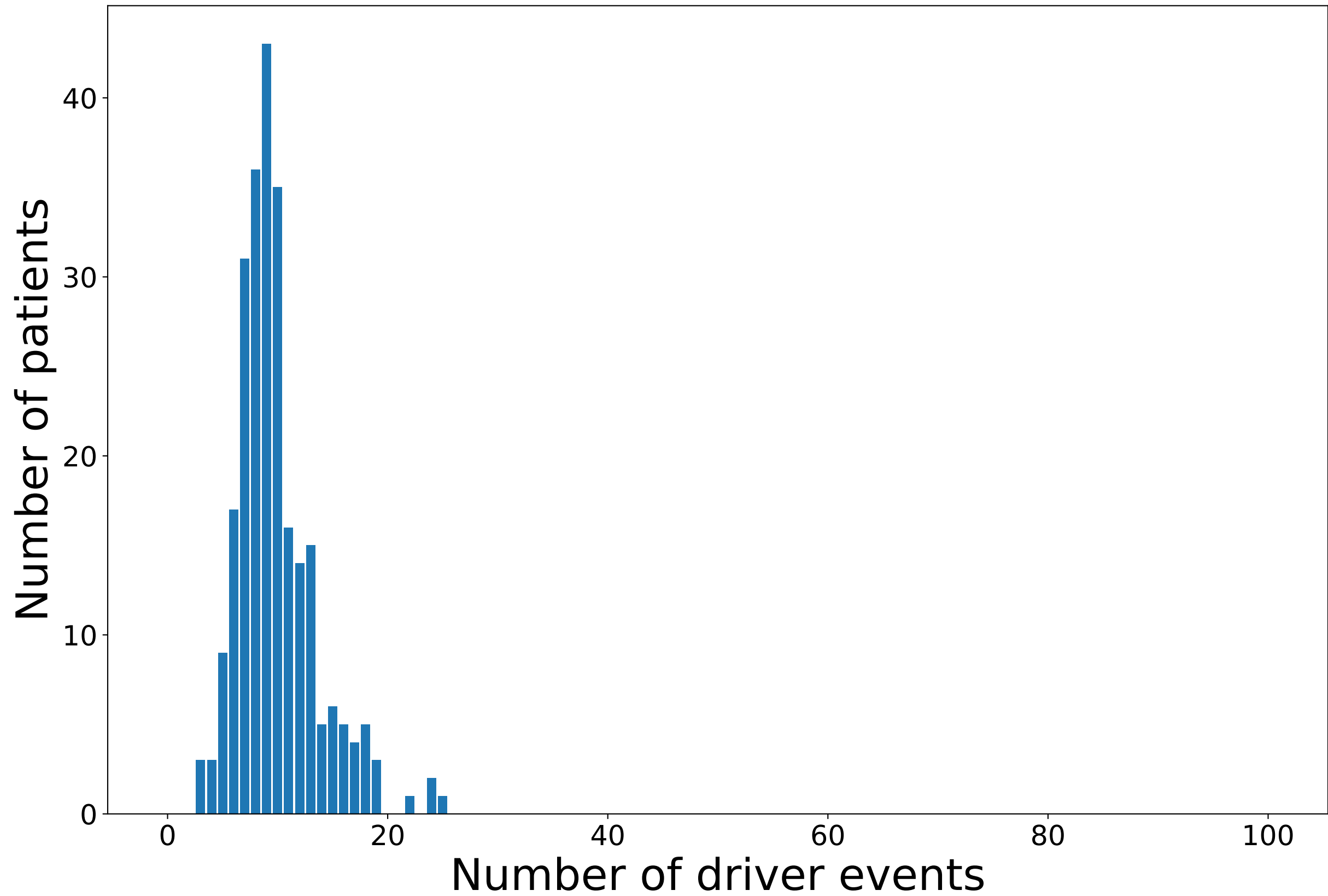

Supplement: S3 Files — (ZIP) [file pgen.1009996.s003.zip › COHORTS/patient distributions/2021_11_23_14_20_GBM.pdf]

# READ\_FEMALE

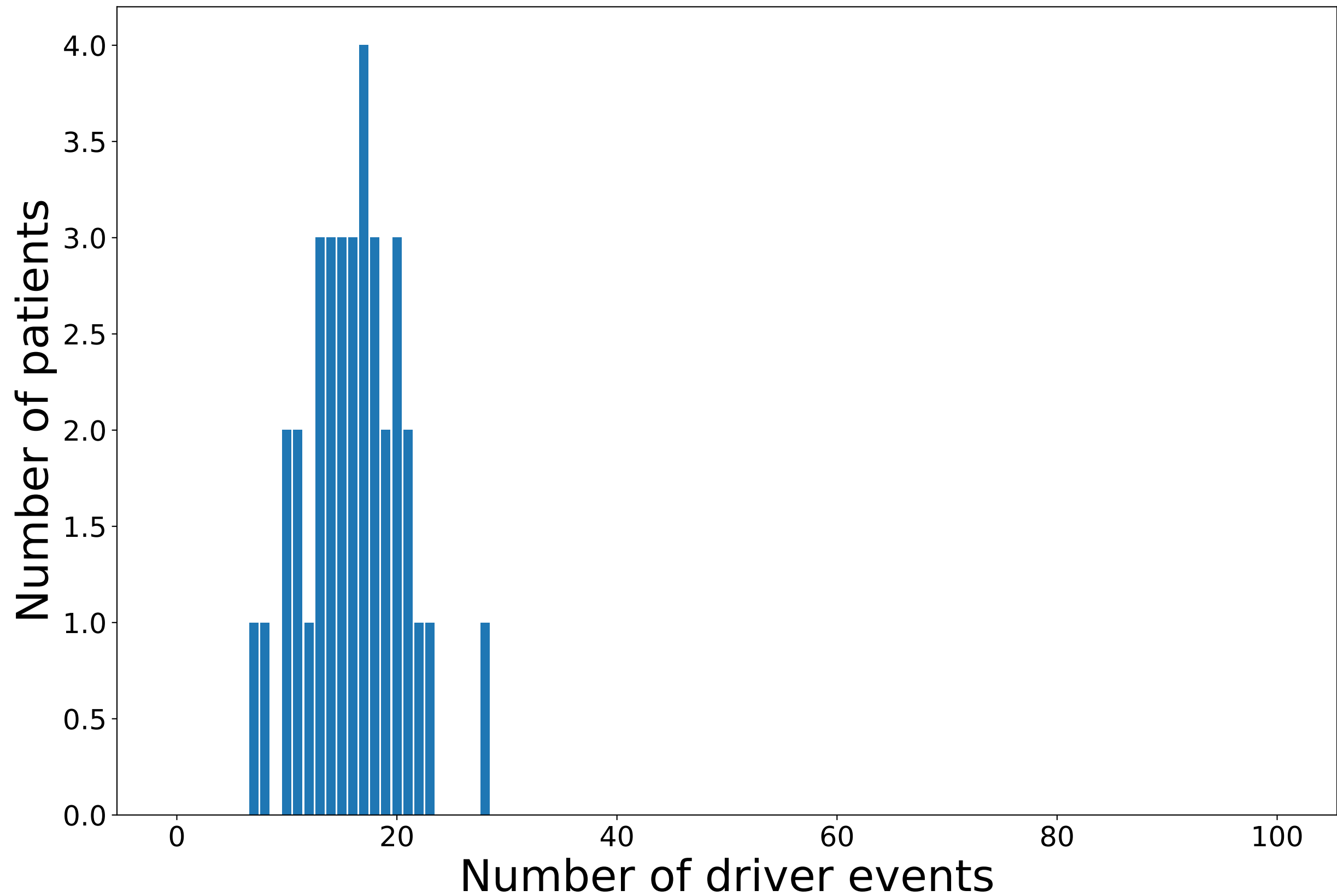

Supplement: S3 Files — (ZIP) [file pgen.1009996.s003.zip › COHORTS/patient distributions/2021_11_23_14_20_READ_FEMALE.pdf]

# BLCA

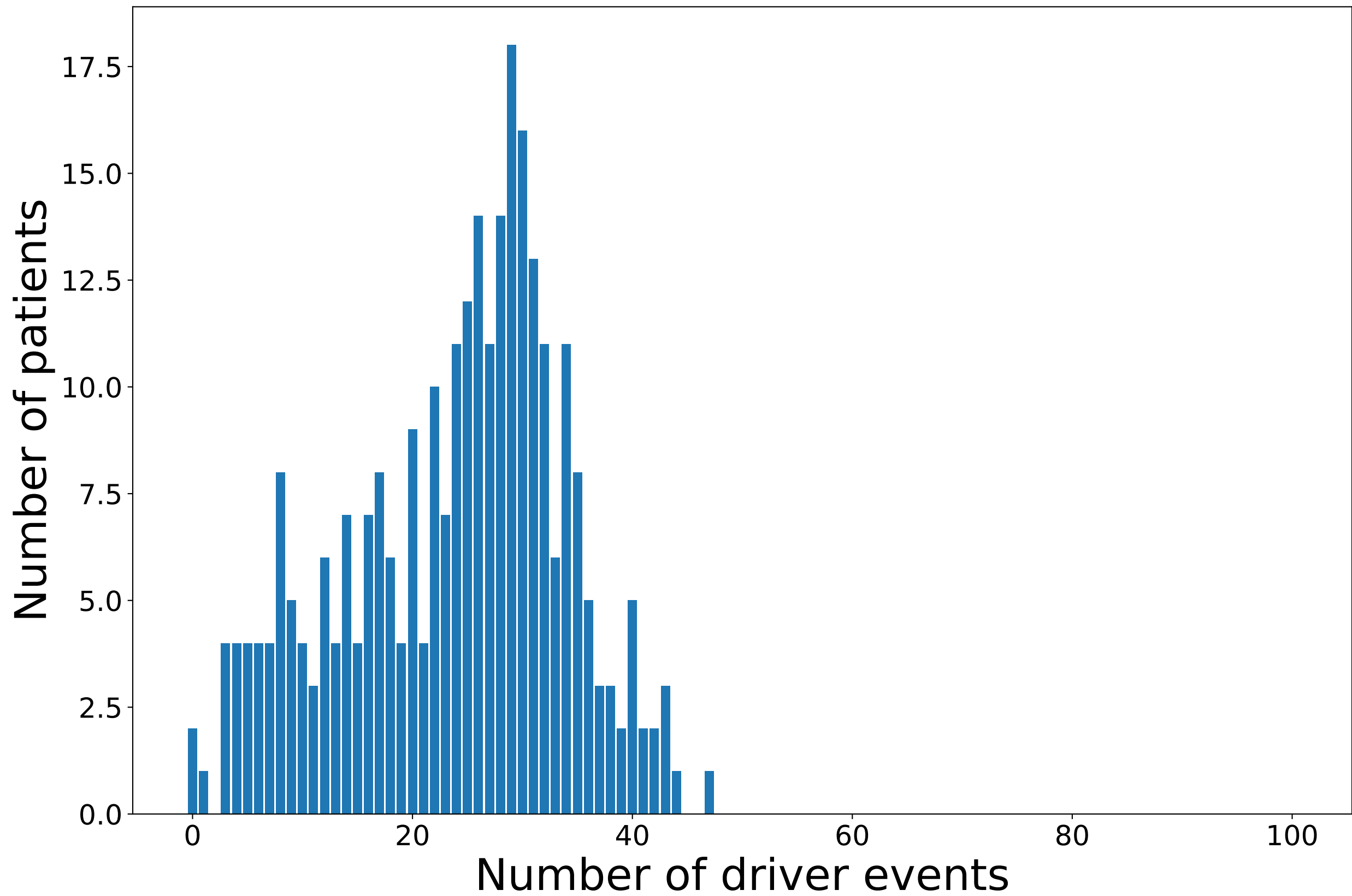

Supplement: S3 Files — (ZIP) [file pgen.1009996.s003.zip › COHORTS/patient distributions/2021_11_23_14_20_BLCA.pdf]

# THYM\_MALE

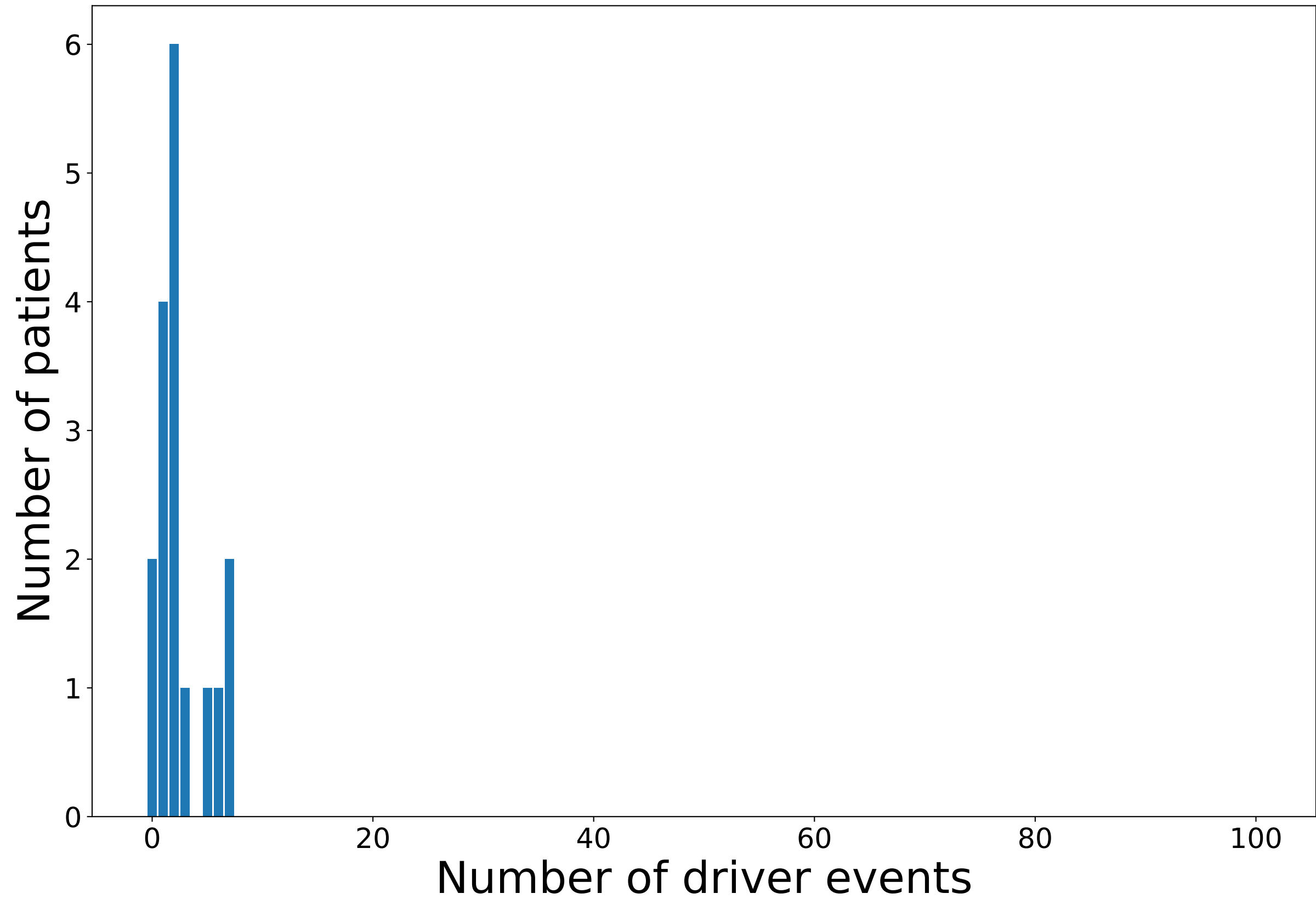

Supplement: S3 Files — (ZIP) [file pgen.1009996.s003.zip › COHORTS/patient distributions/2021_11_23_14_20_THYM_MALE.pdf]

# THCA

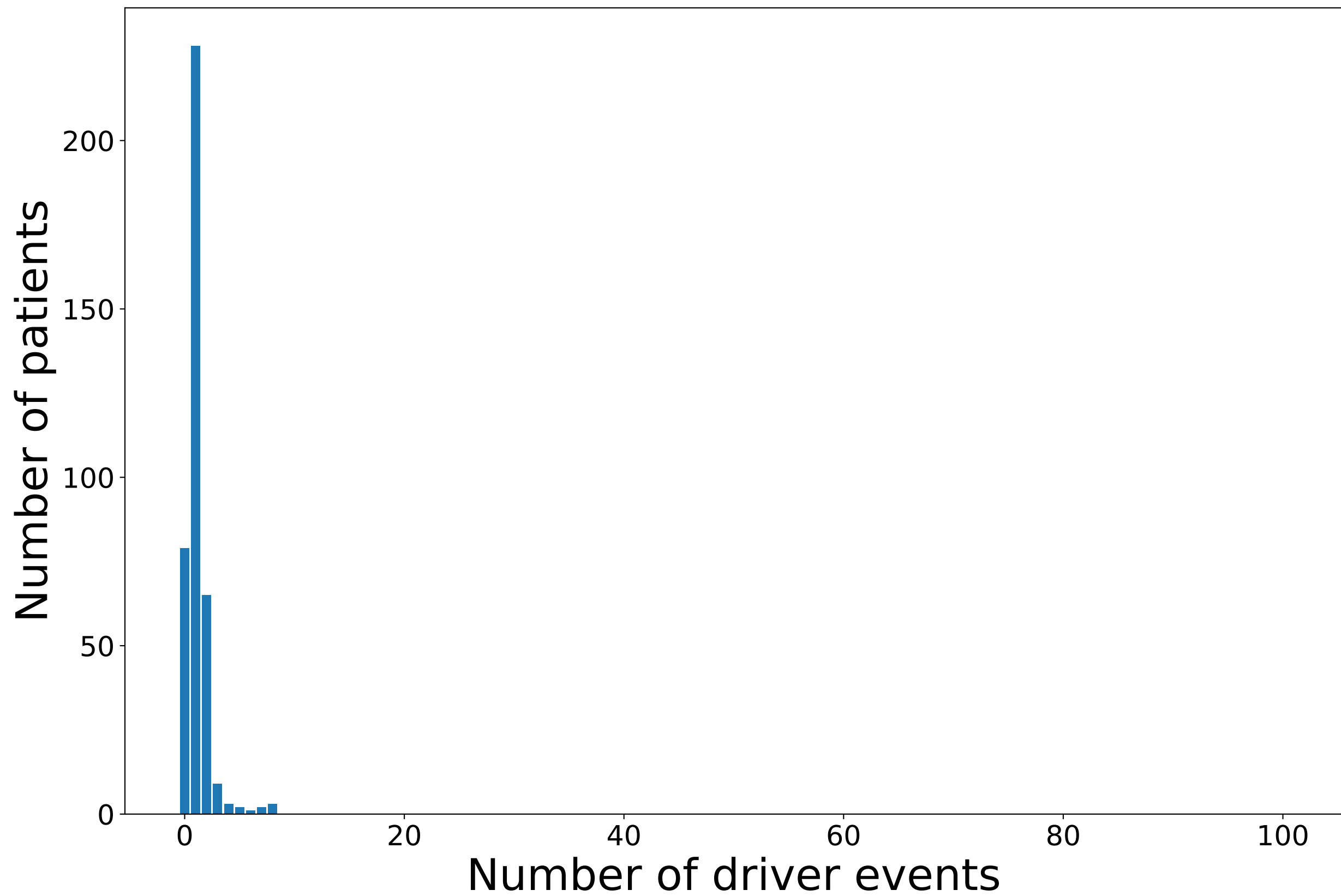

Supplement: S3 Files — (ZIP) [file pgen.1009996.s003.zip › COHORTS/patient distributions/2021_11_23_14_20_THCA.pdf]

# LUAD\_FEMALE

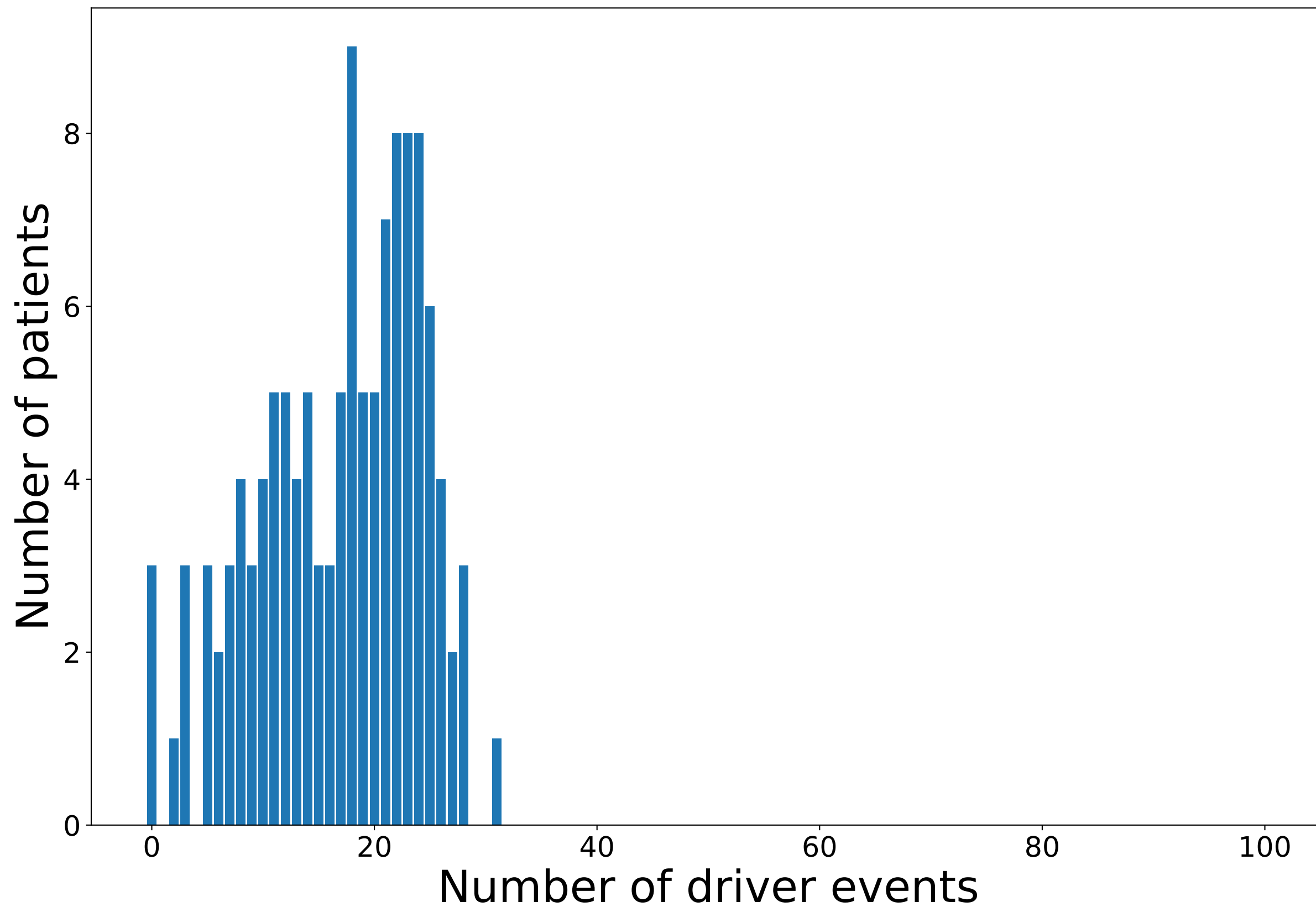

Supplement: S3 Files — (ZIP) [file pgen.1009996.s003.zip › COHORTS/patient distributions/2021_11_23_14_20_LUAD_FEMALE.pdf]

# PAAD

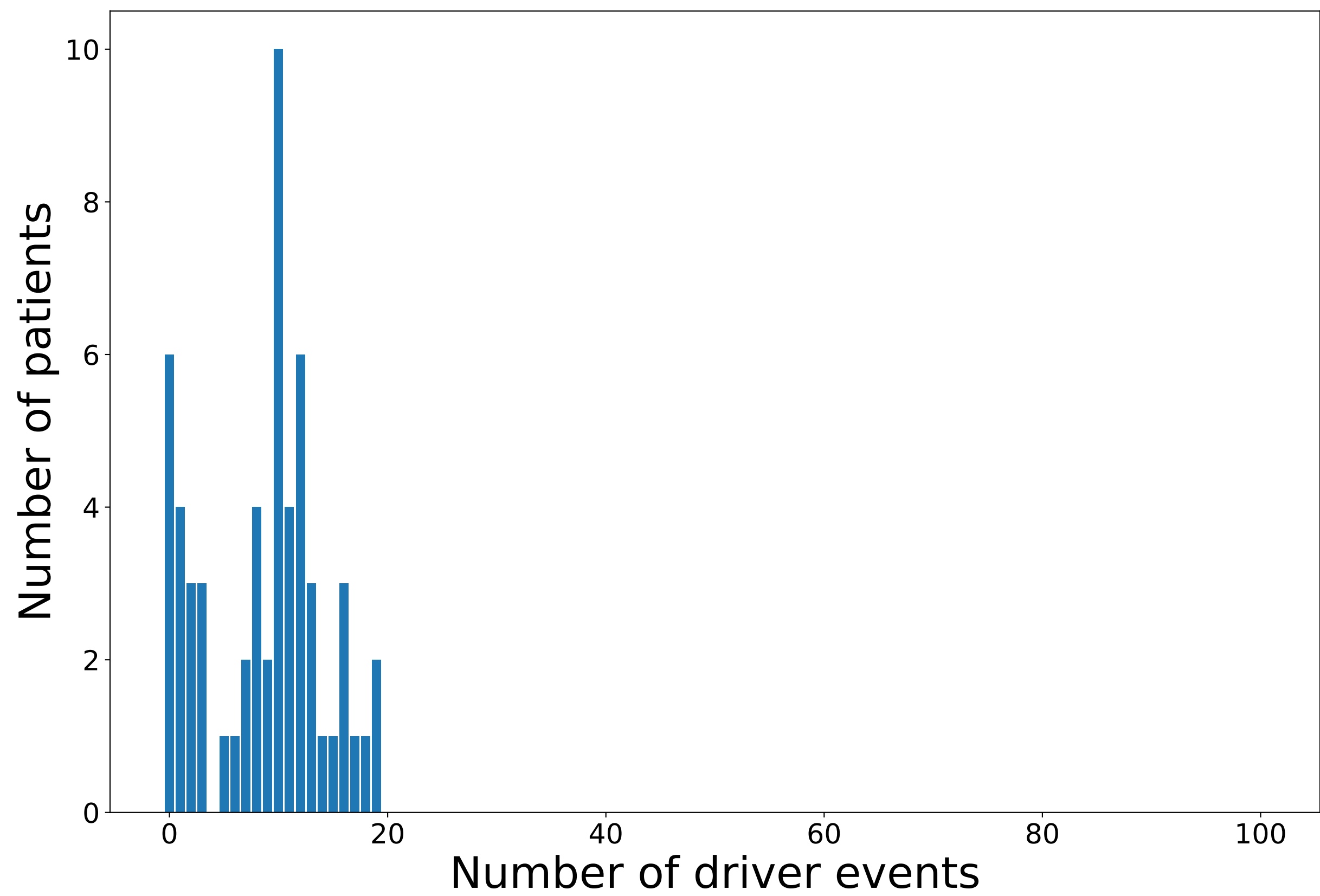

Supplement: S3 Files — (ZIP) [file pgen.1009996.s003.zip › COHORTS/patient distributions/2021_11_23_14_20_PAAD.pdf]

# READ

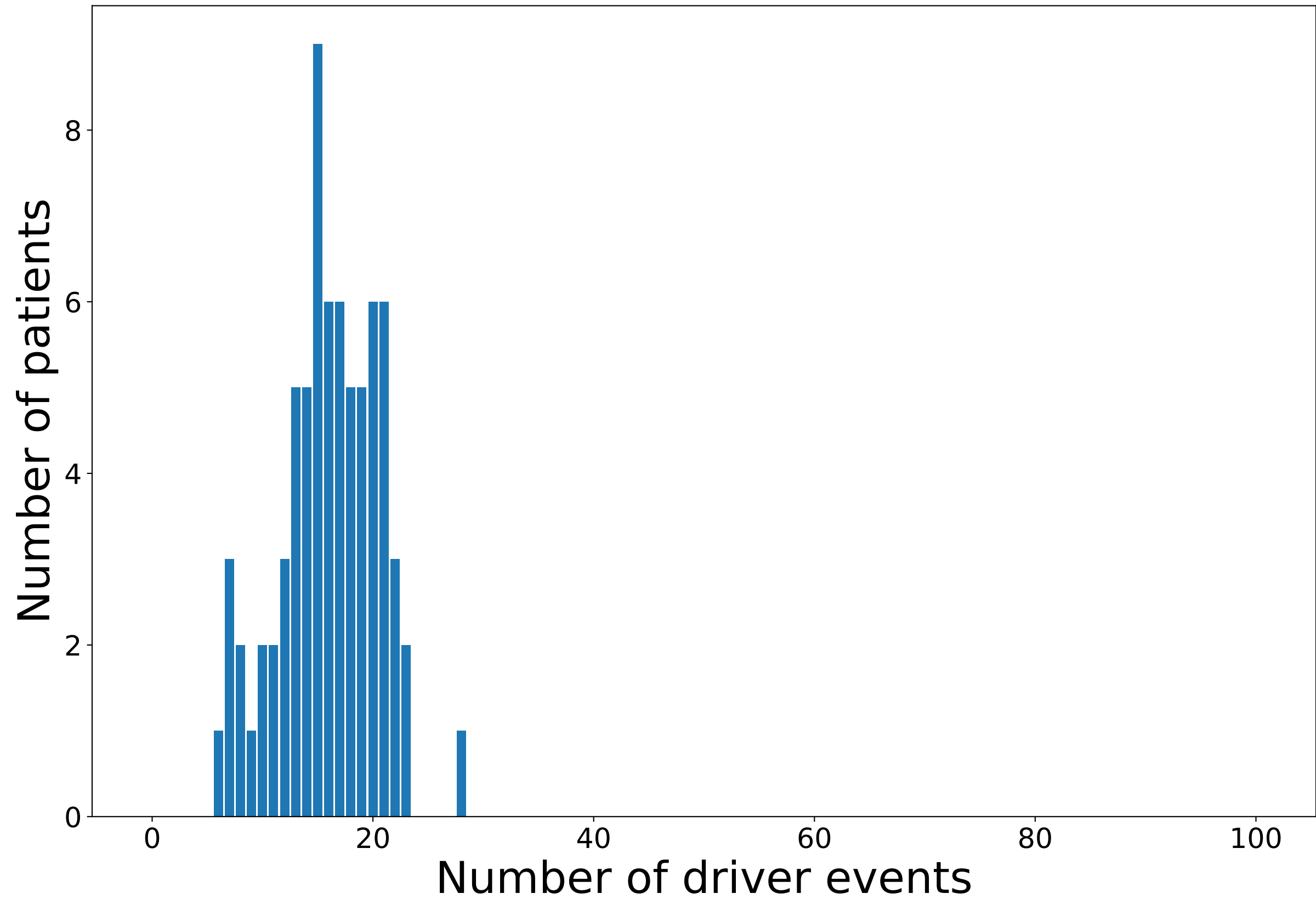

Supplement: S3 Files — (ZIP) [file pgen.1009996.s003.zip › COHORTS/patient distributions/2021_11_23_14_20_READ.pdf]

# STAD\_MALE

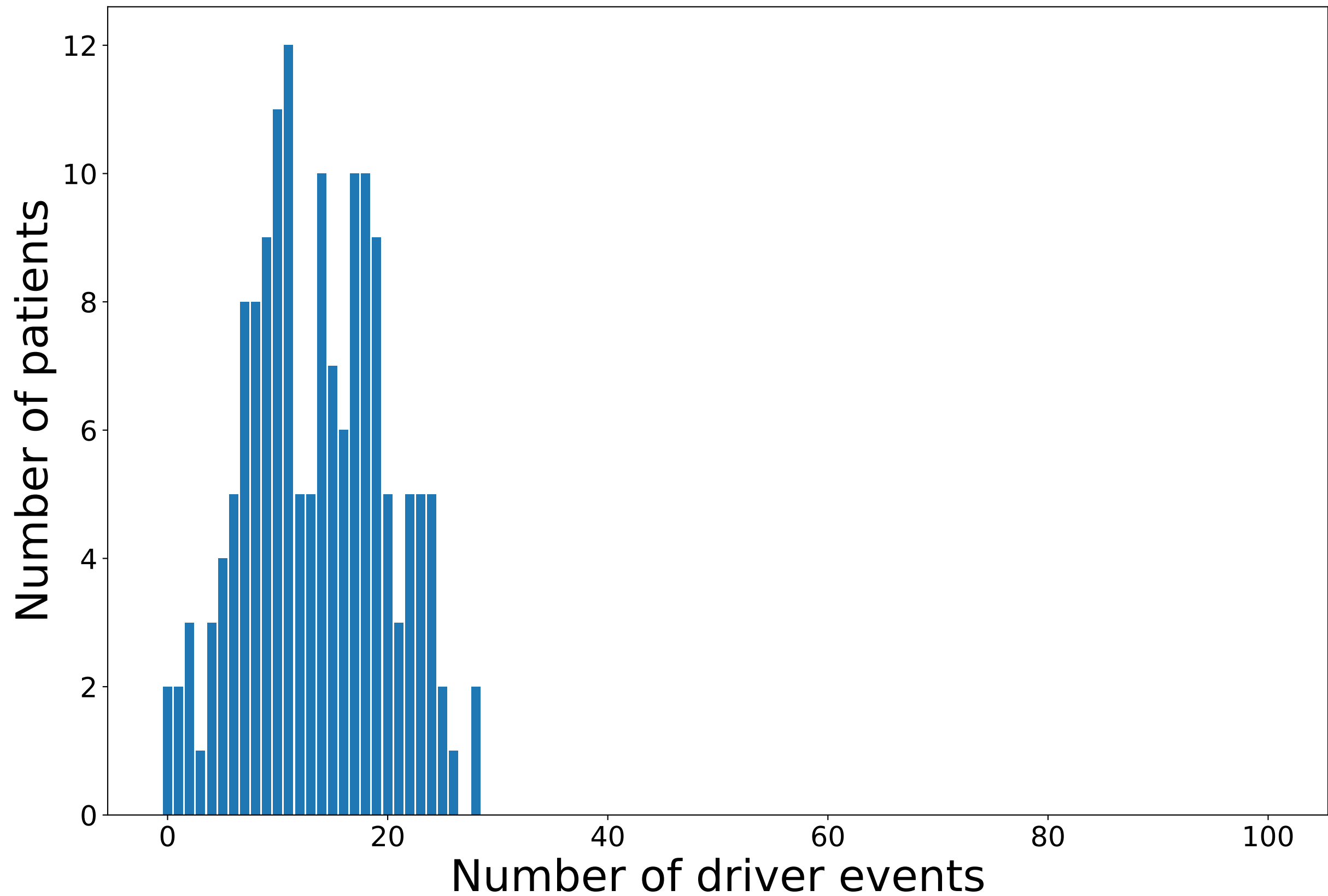

Supplement: S3 Files — (ZIP) [file pgen.1009996.s003.zip › COHORTS/patient distributions/2021_11_23_14_20_STAD_MALE.pdf]

# SARC\_FEMALE

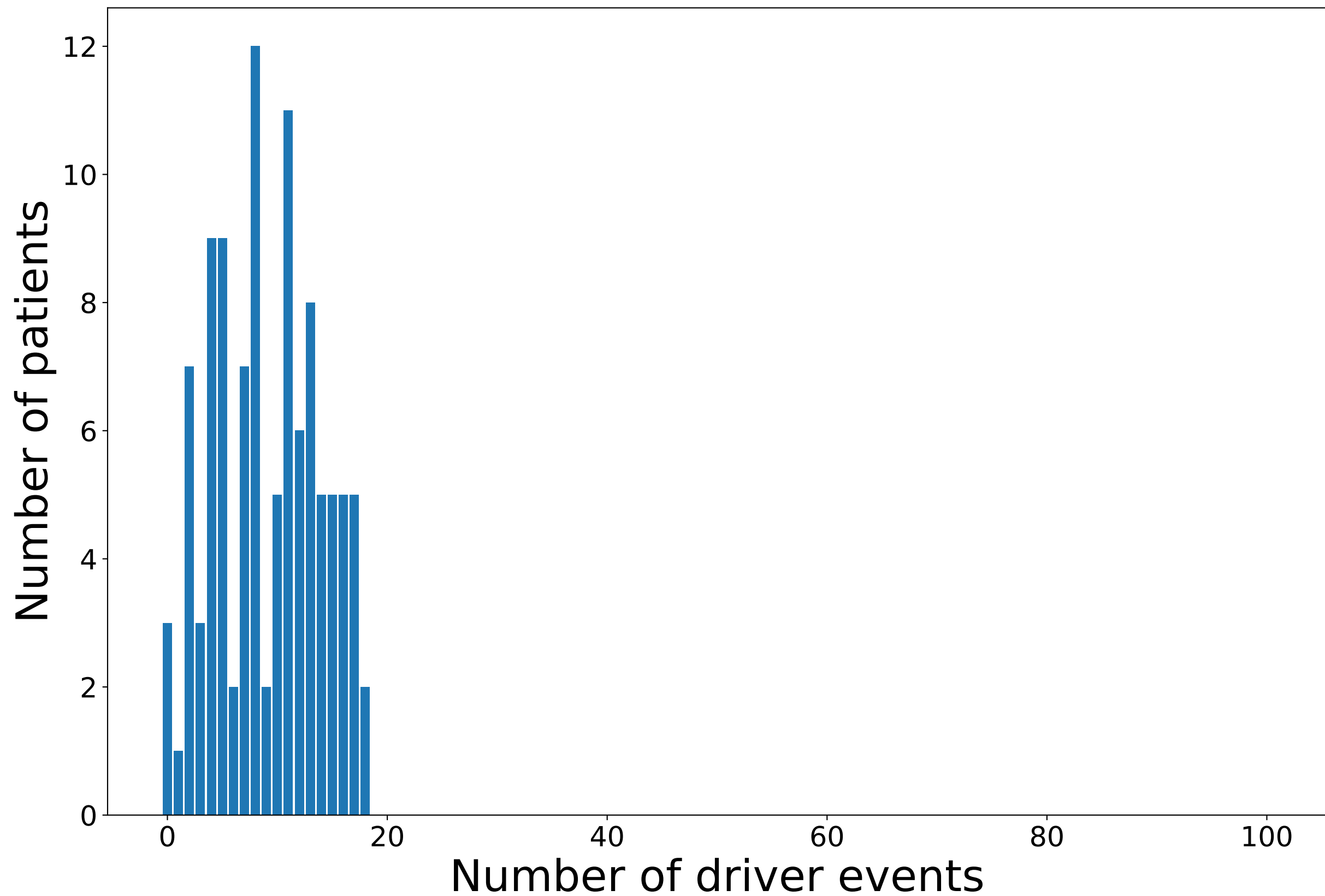

Supplement: S3 Files — (ZIP) [file pgen.1009996.s003.zip › COHORTS/patient distributions/2021_11_23_14_20_SARC_FEMALE.pdf]

# UCS

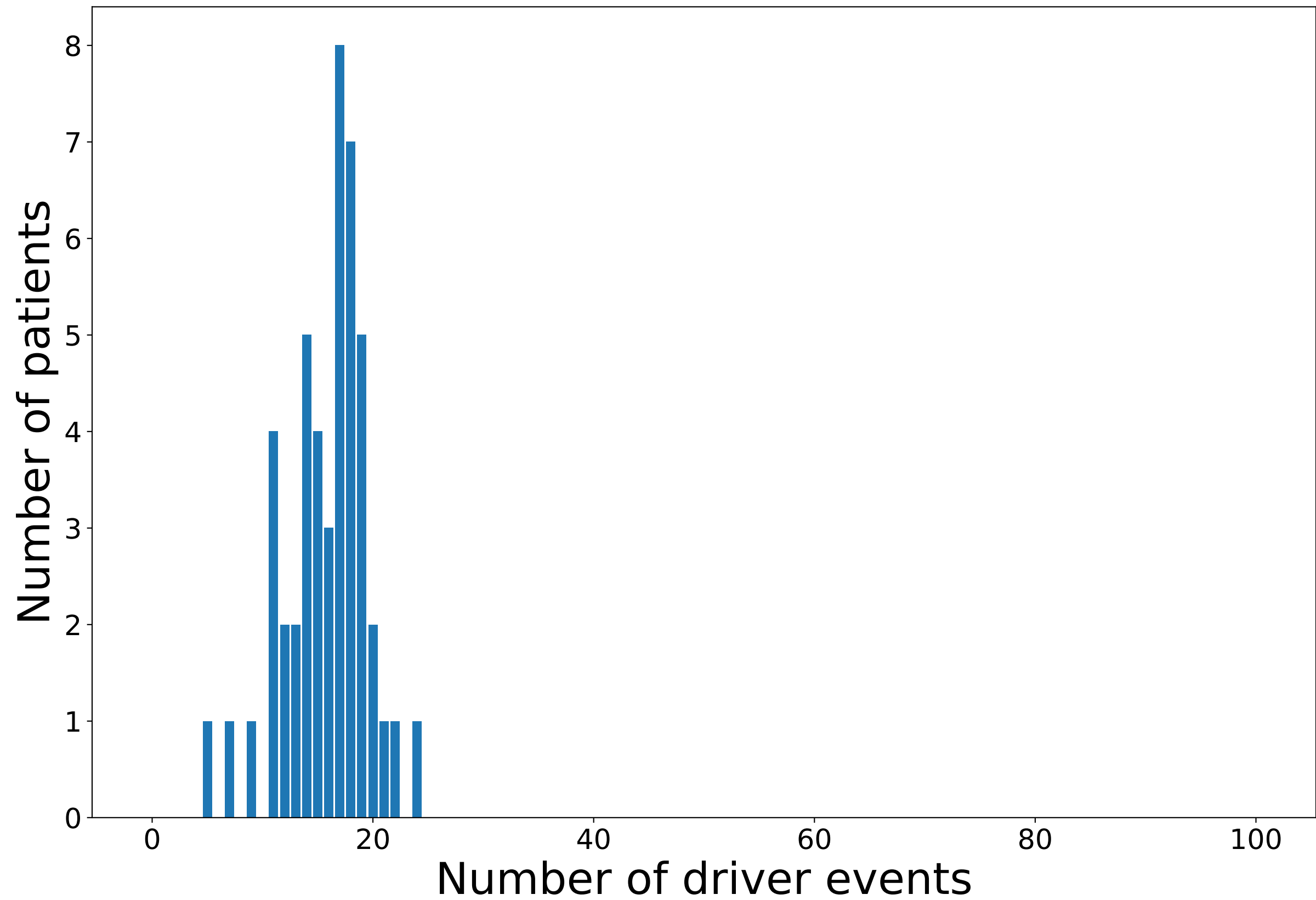

Supplement: S3 Files — (ZIP) [file pgen.1009996.s003.zip › COHORTS/patient distributions/2021_11_23_14_20_UCS.pdf]

# UVM\_MALE

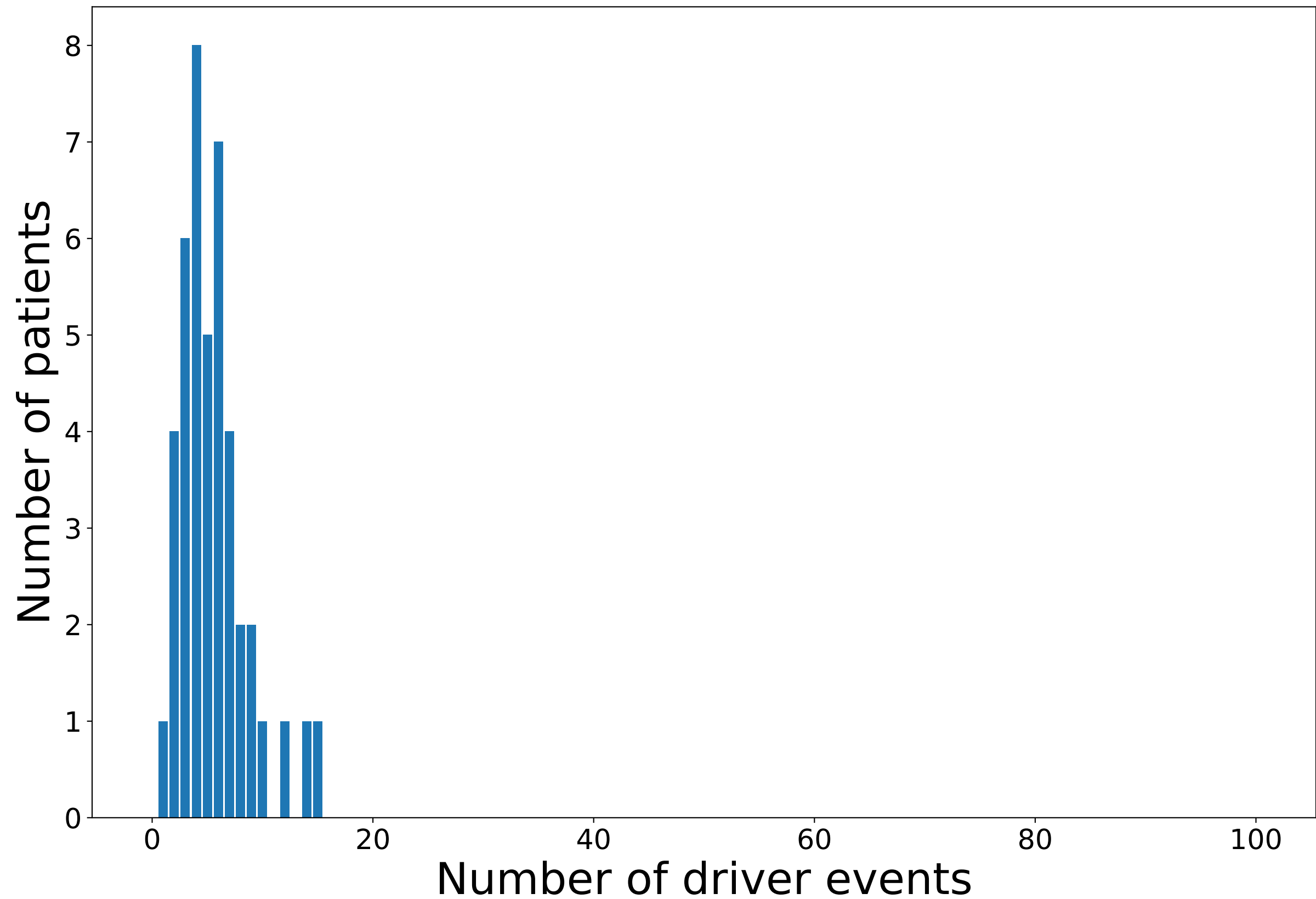

Supplement: S3 Files — (ZIP) [file pgen.1009996.s003.zip › COHORTS/patient distributions/2021_11_23_14_20_UVM_MALE.pdf]

# DLBC

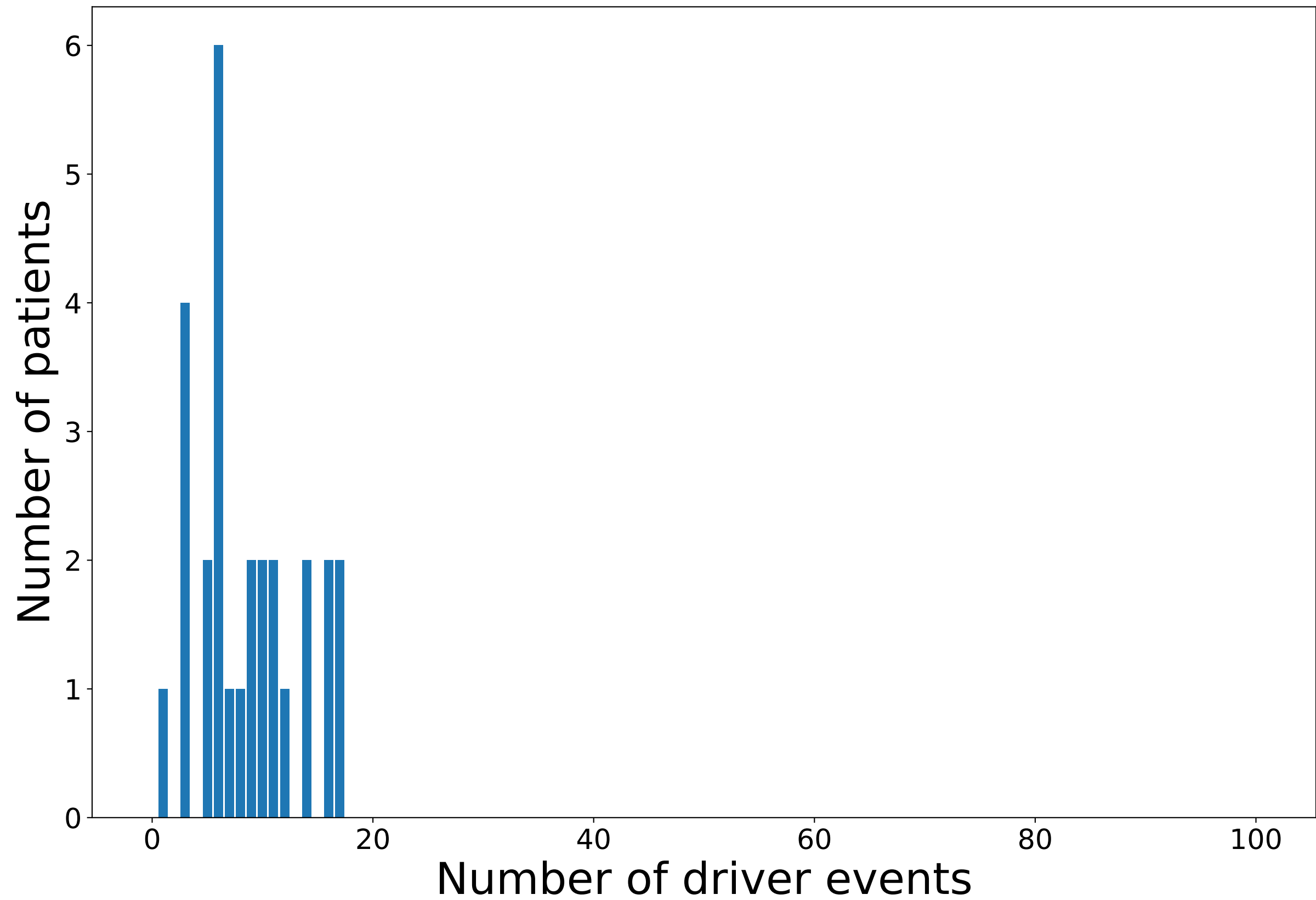

Supplement: S3 Files — (ZIP) [file pgen.1009996.s003.zip › COHORTS/patient distributions/2021_11_23_14_20_DLBC.pdf]

# MESO

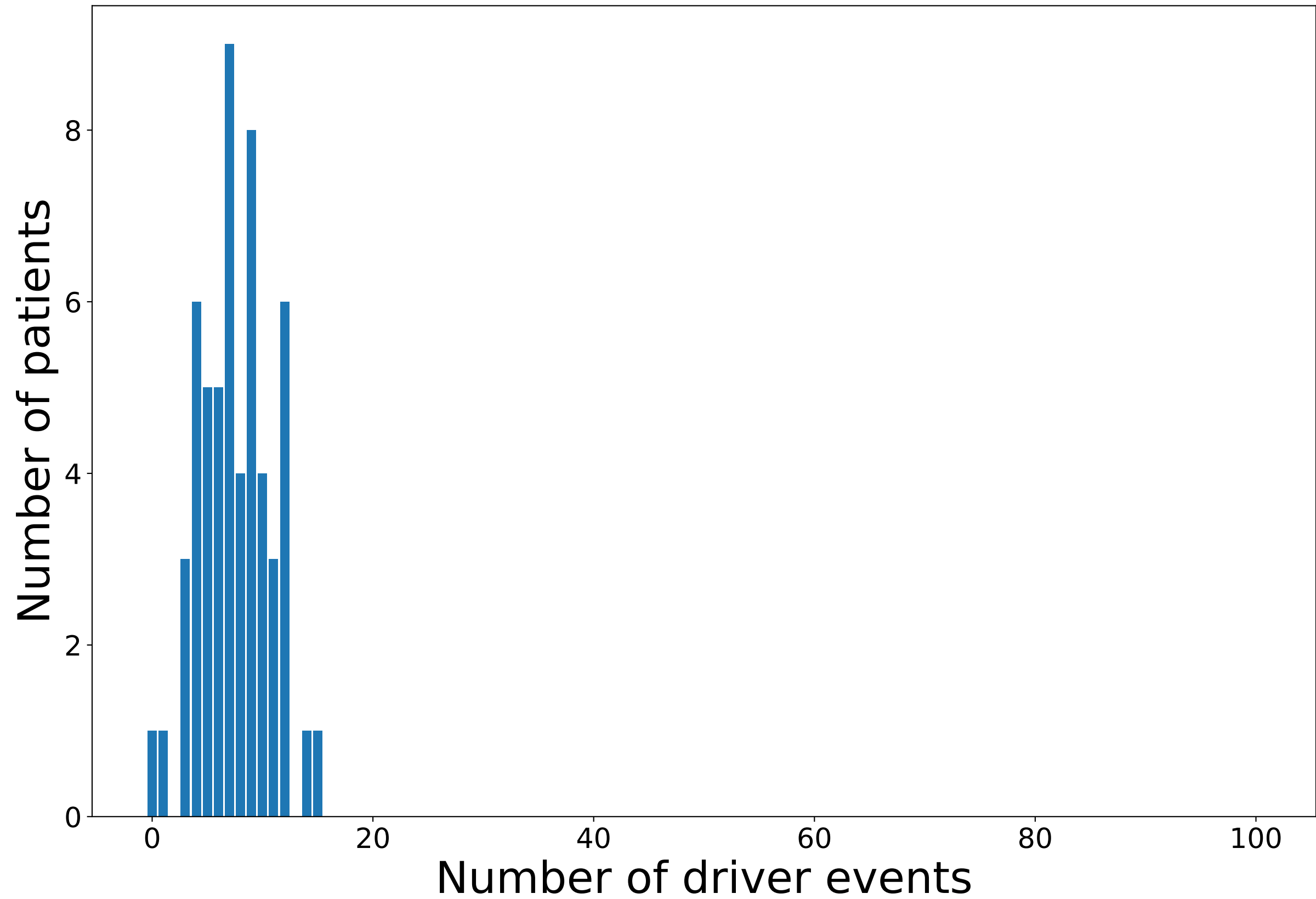

Supplement: S3 Files — (ZIP) [file pgen.1009996.s003.zip › COHORTS/patient distributions/2021_11_23_14_20_MESO.pdf]

# SKCM

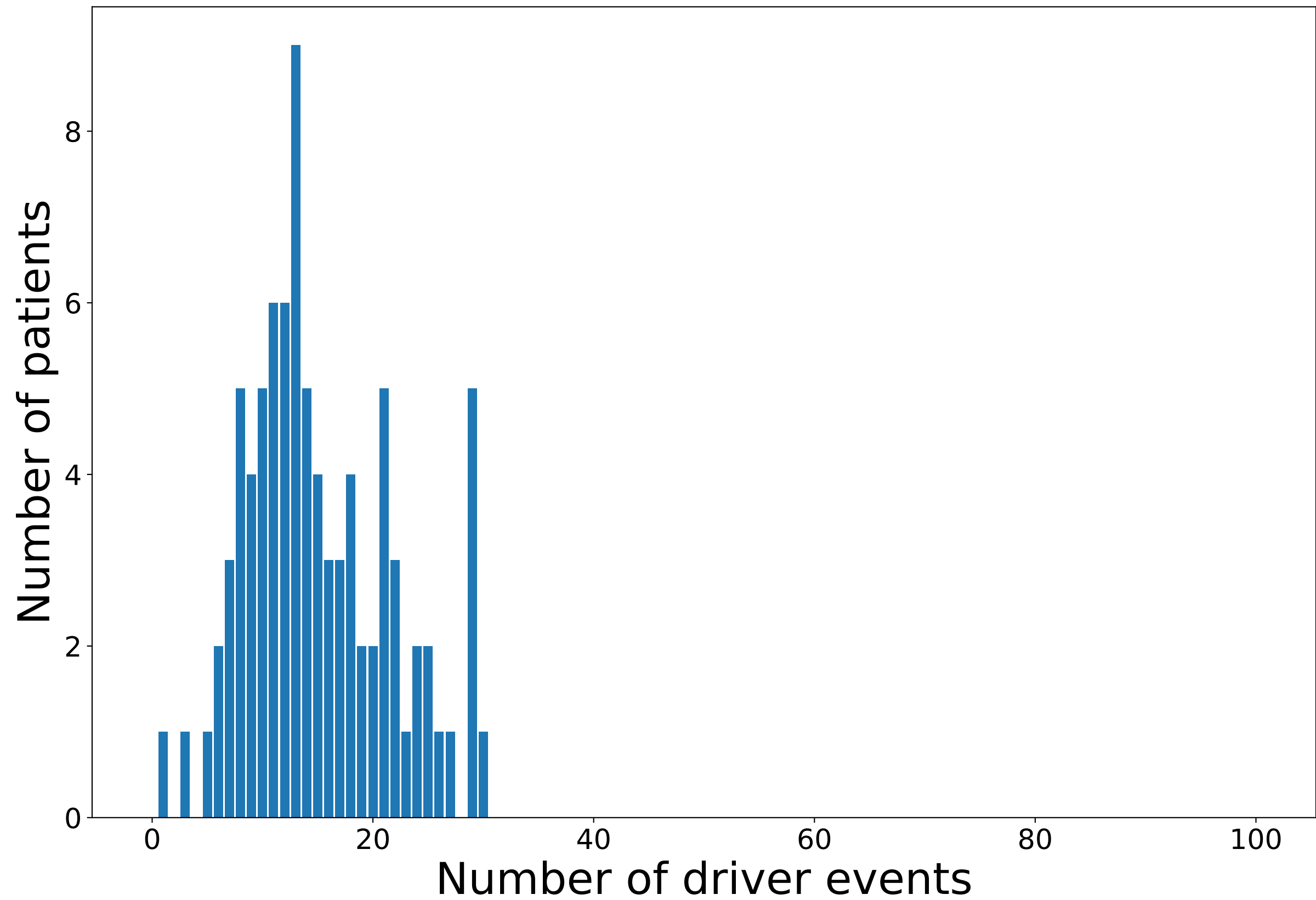

Supplement: S3 Files — (ZIP) [file pgen.1009996.s003.zip › COHORTS/patient distributions/2021_11_23_14_20_SKCM.pdf]

# PANCAN

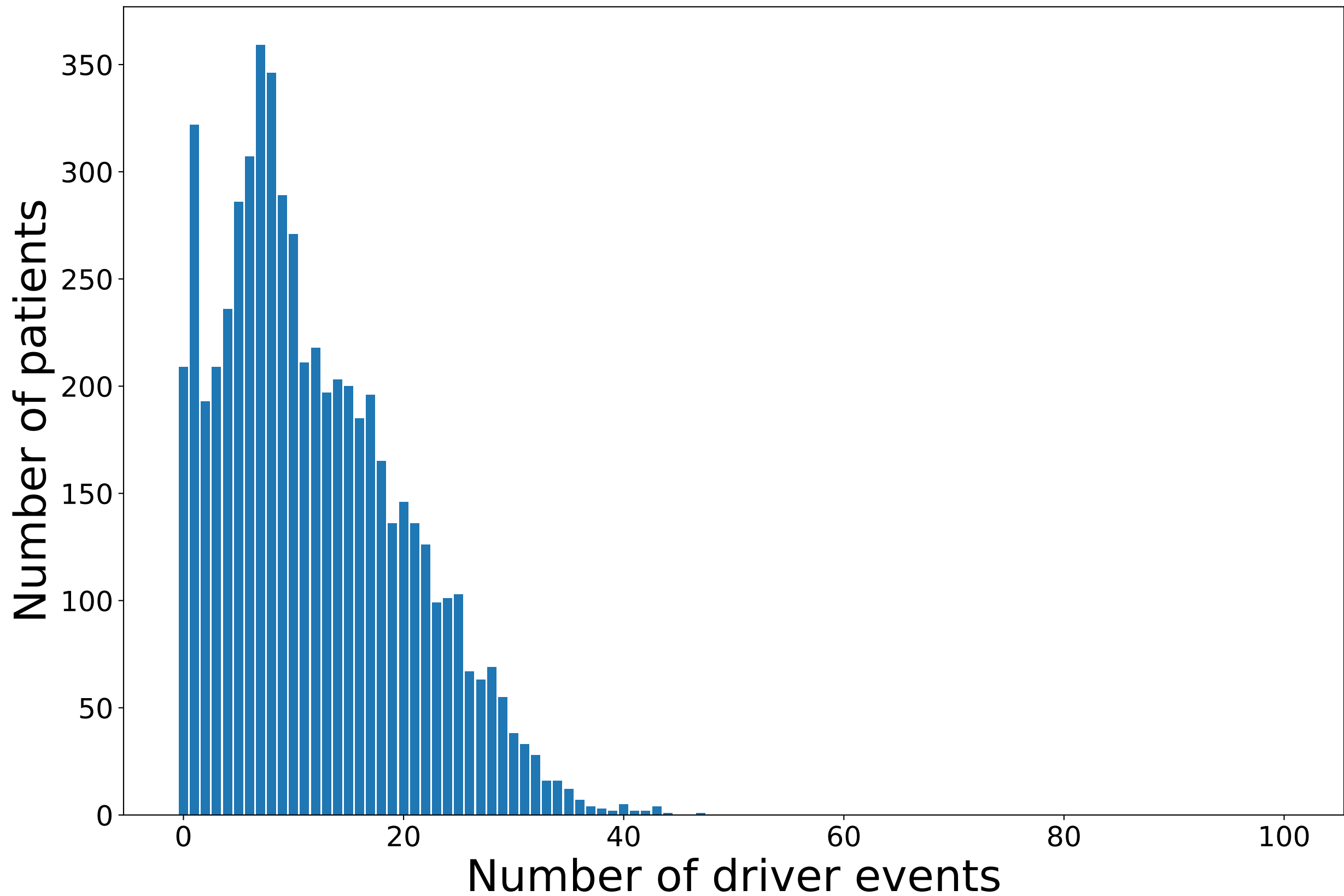

Supplement: S3 Files — (ZIP) [file pgen.1009996.s003.zip › COHORTS/patient distributions/2021_11_23_14_20_PANCAN.pdf]

# CHOL\_MALE

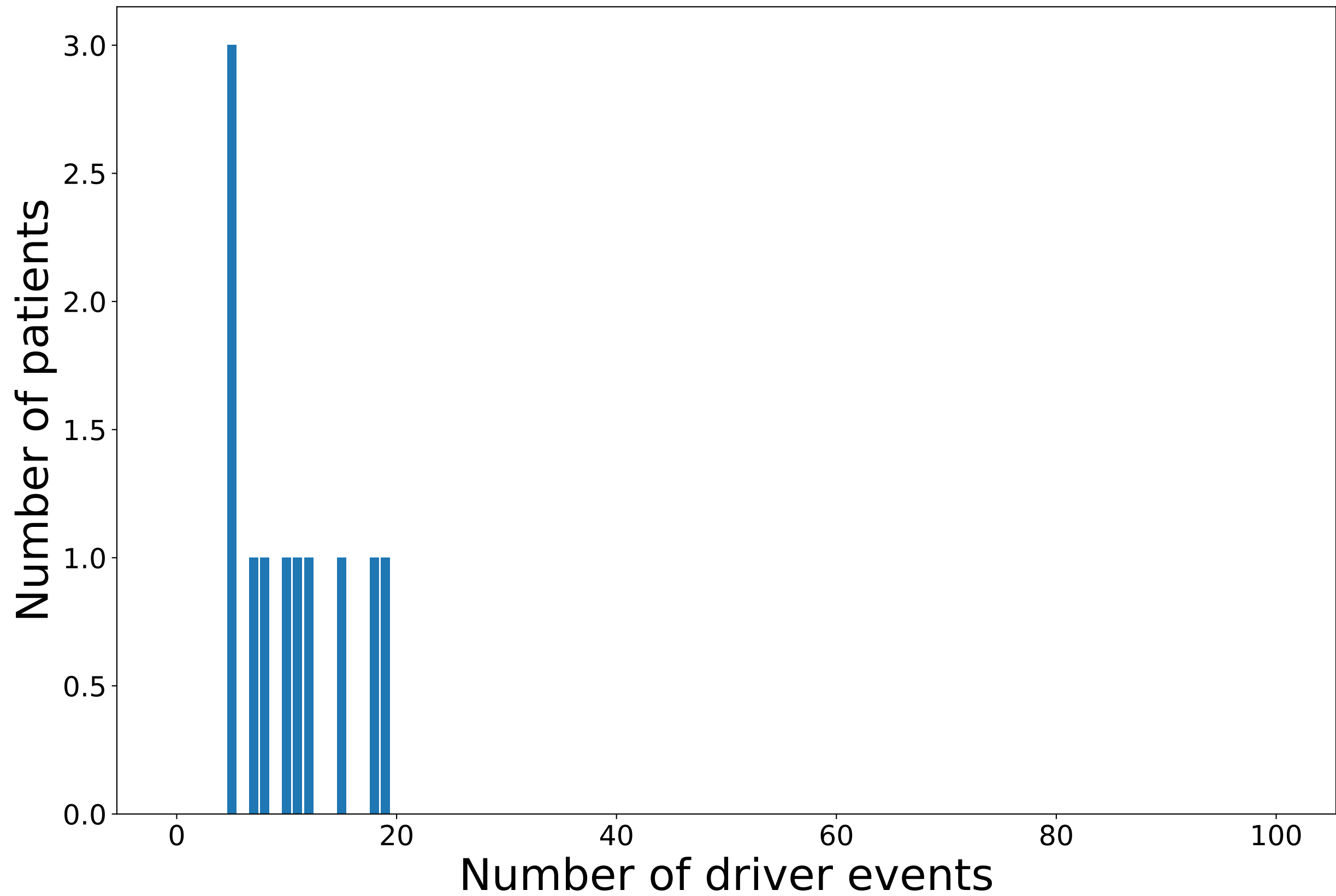

Supplement: S3 Files — (ZIP) [file pgen.1009996.s003.zip › COHORTS/patient distributions/2021_11_23_14_20_CHOL_MALE.pdf]

# COAD\_MALE

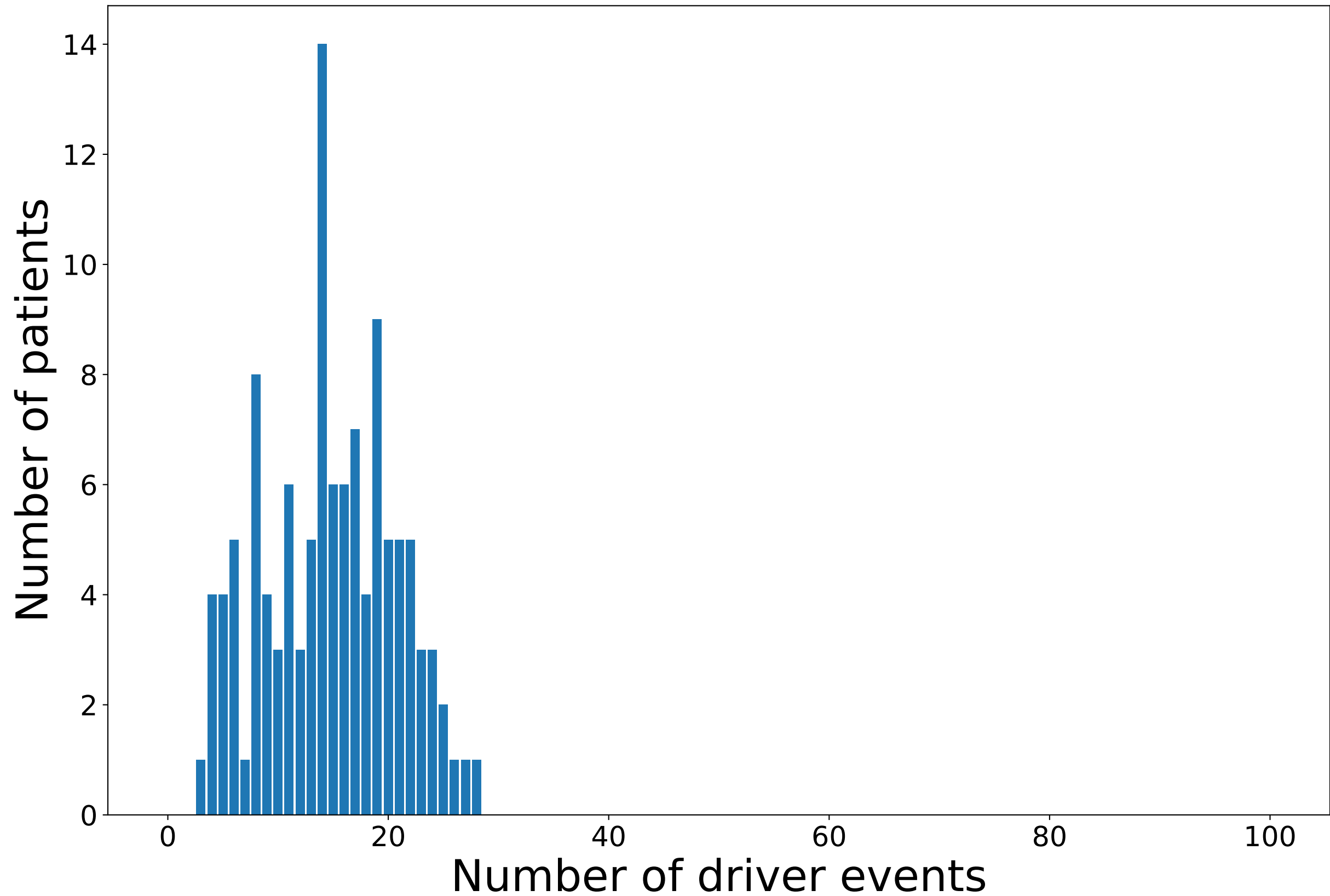

Supplement: S3 Files — (ZIP) [file pgen.1009996.s003.zip › COHORTS/patient distributions/2021_11_23_14_20_COAD_MALE.pdf]

# BLCA\_MALE

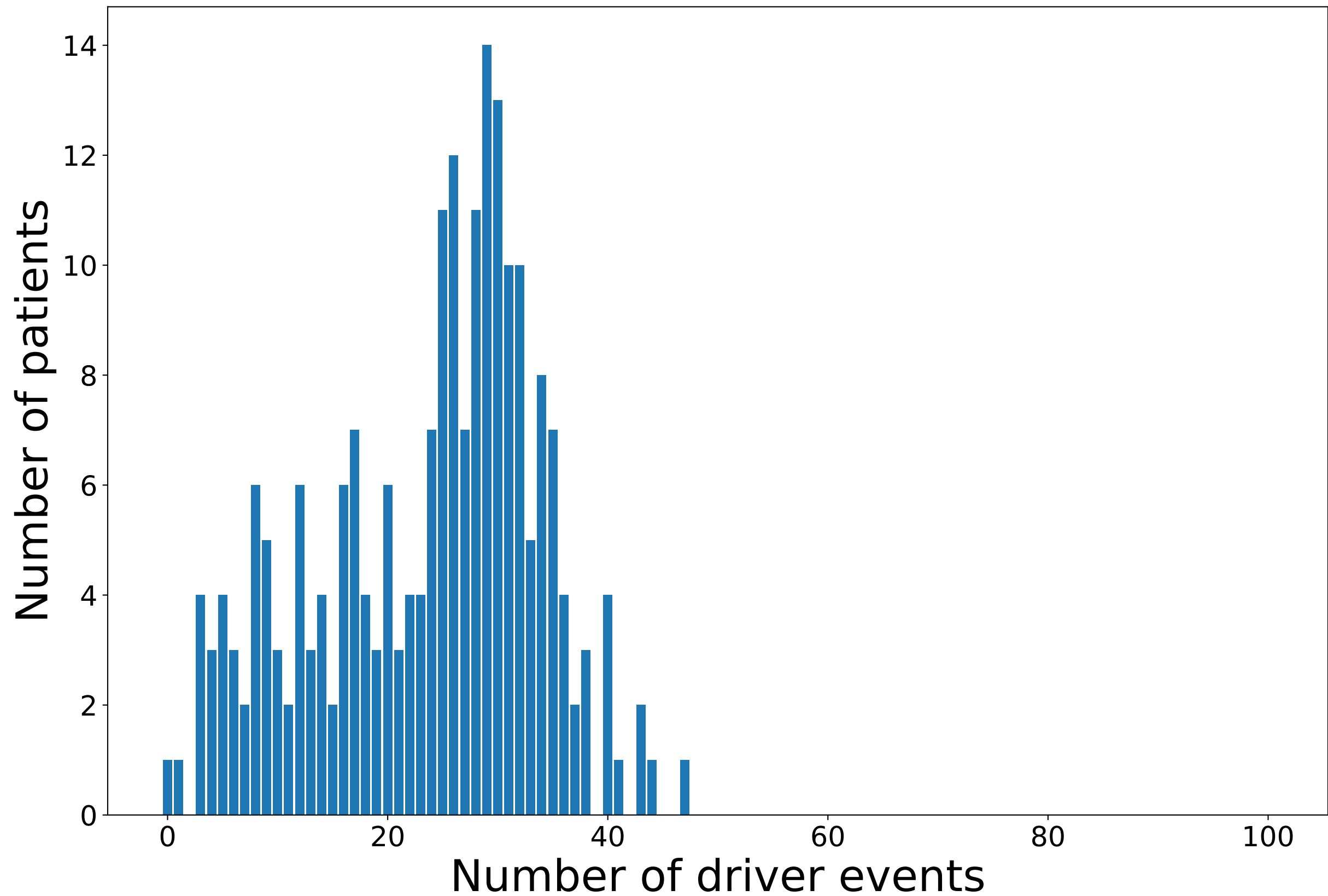

Supplement: S3 Files — (ZIP) [file pgen.1009996.s003.zip › COHORTS/patient distributions/2021_11_23_14_20_BLCA_MALE.pdf]

# COAD\_FEMALE

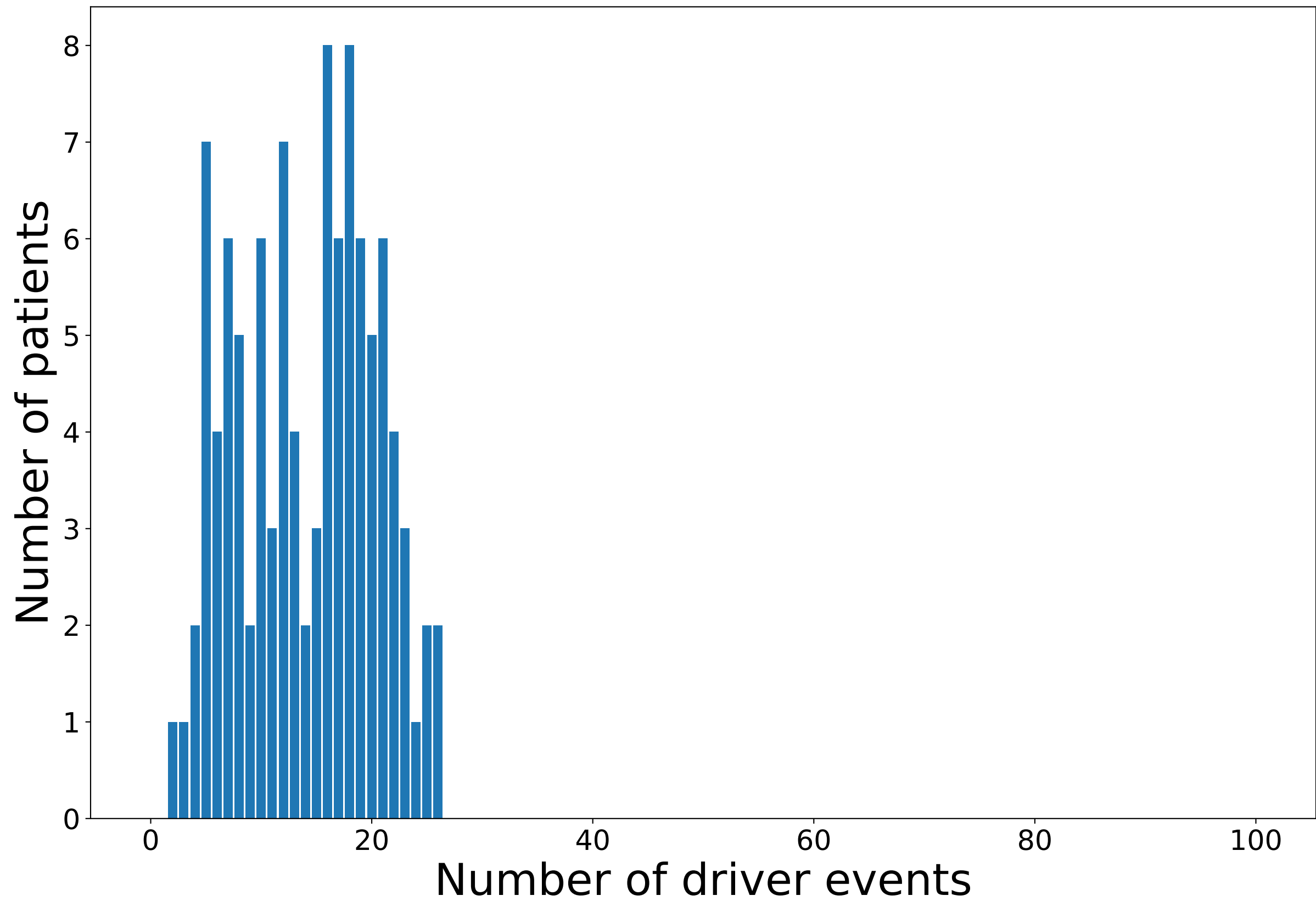

Supplement: S3 Files — (ZIP) [file pgen.1009996.s003.zip › COHORTS/patient distributions/2021_11_23_14_20_COAD_FEMALE.pdf]

# BRCA

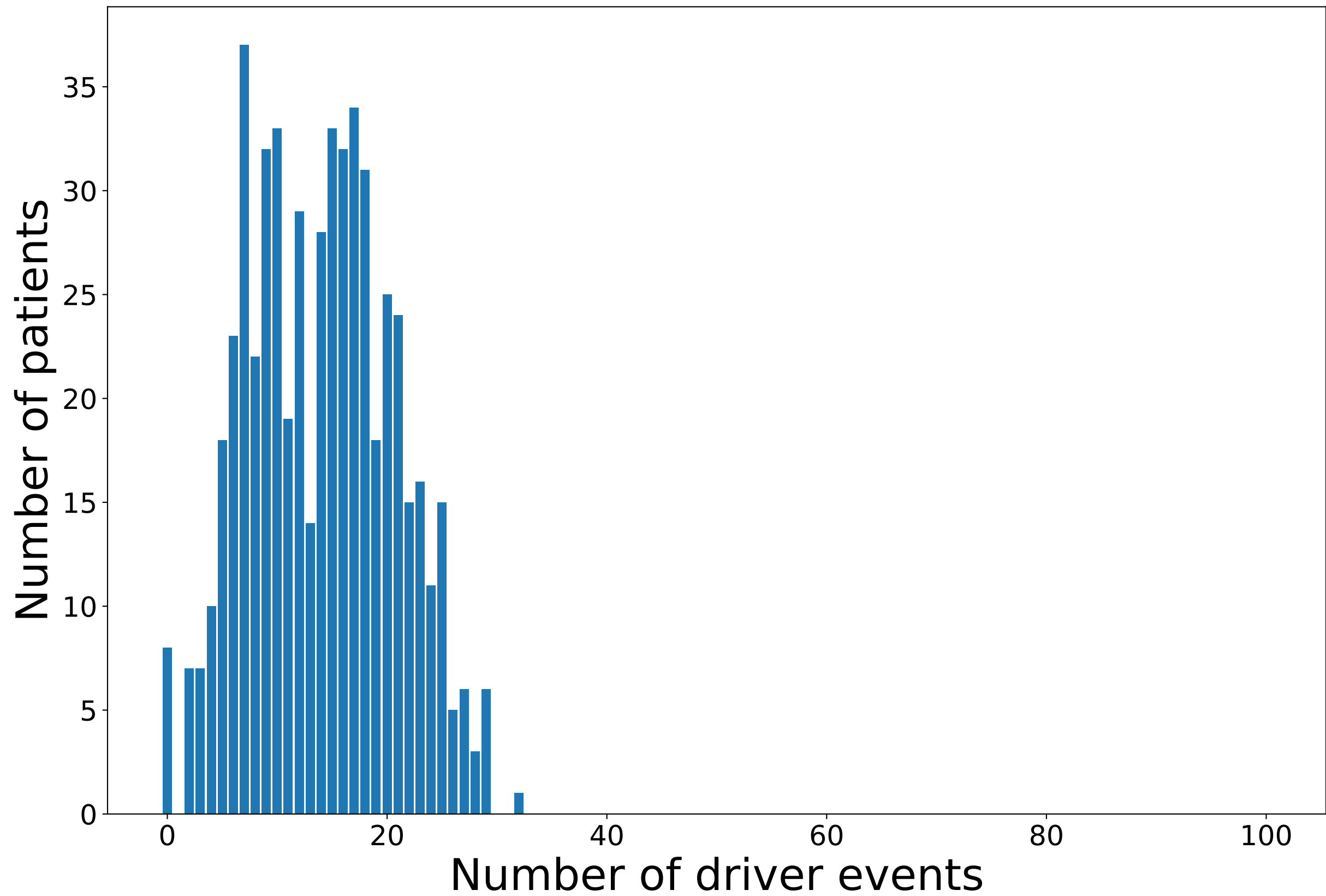

Supplement: S3 Files — (ZIP) [file pgen.1009996.s003.zip › COHORTS/patient distributions/2021_11_23_14_20_BRCA.pdf]

# KIRC\_MALE

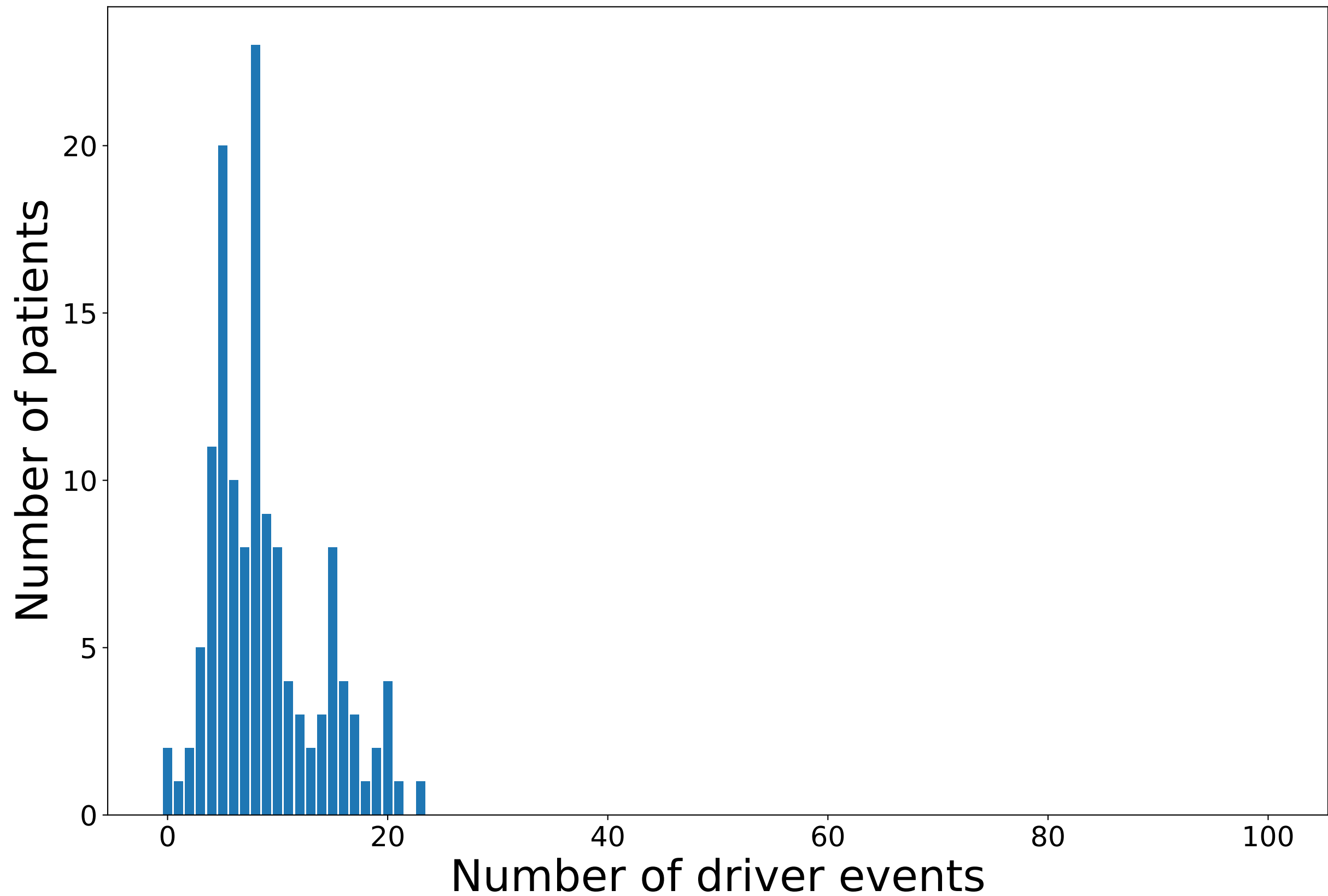

Supplement: S3 Files — (ZIP) [file pgen.1009996.s003.zip › COHORTS/patient distributions/2021_11_23_14_20_KIRC_MALE.pdf]

# ACC\_FEMALE

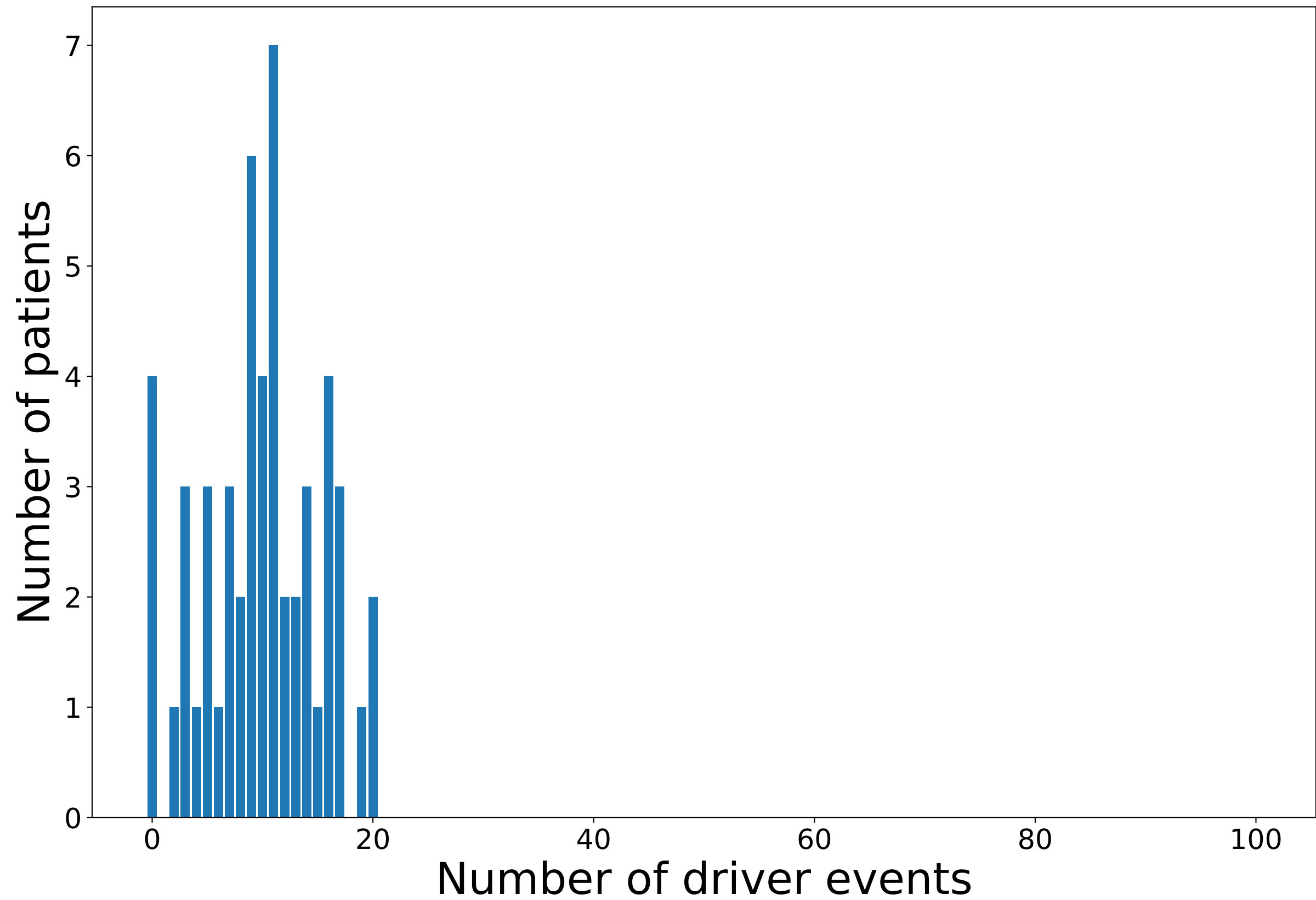

Supplement: S3 Files — (ZIP) [file pgen.1009996.s003.zip › COHORTS/patient distributions/2021_11_23_14_20_ACC_FEMALE.pdf]

# UCEC

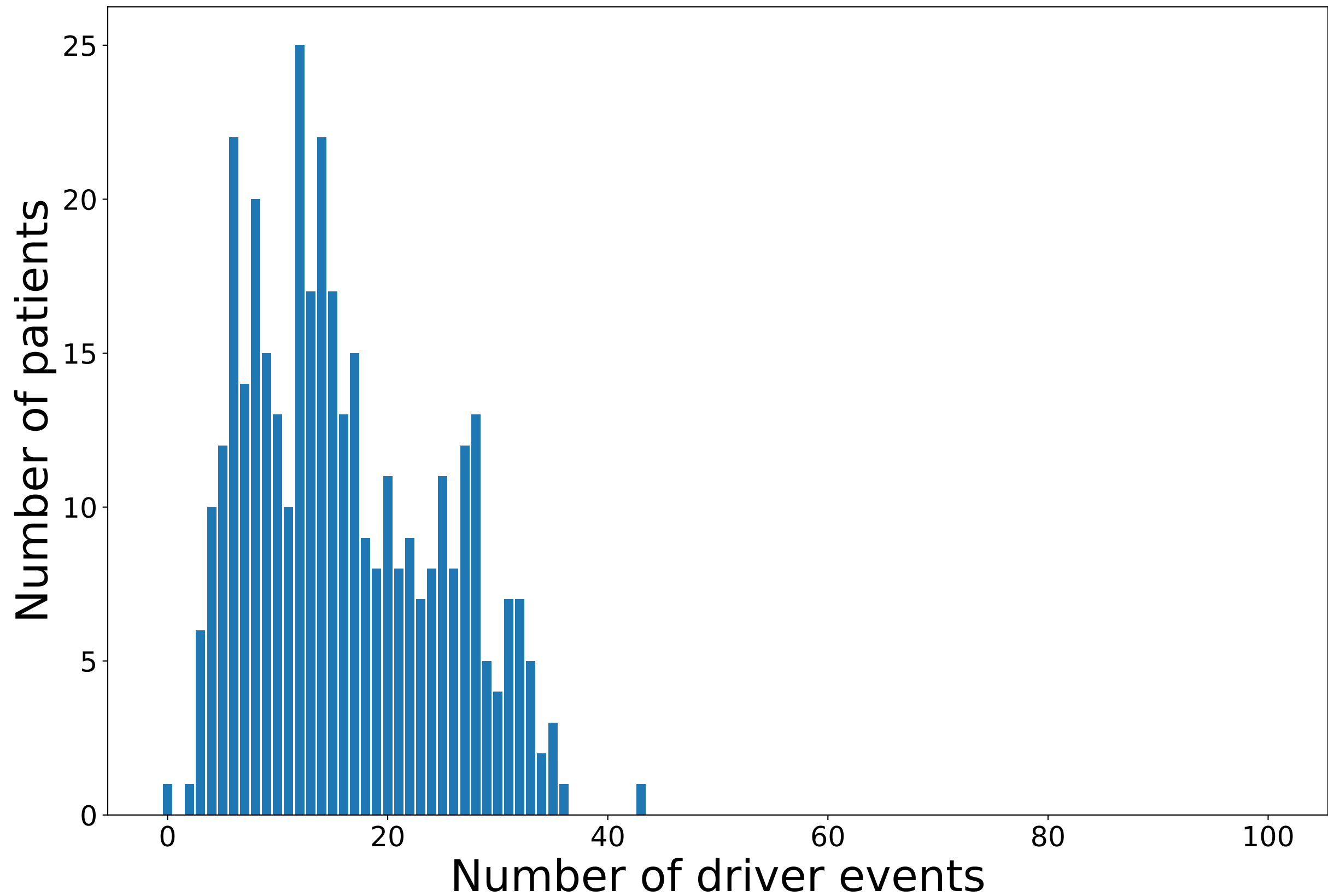

Supplement: S3 Files — (ZIP) [file pgen.1009996.s003.zip › COHORTS/patient distributions/2021_11_23_14_20_UCEC.pdf]

# THCA\_FEMALE

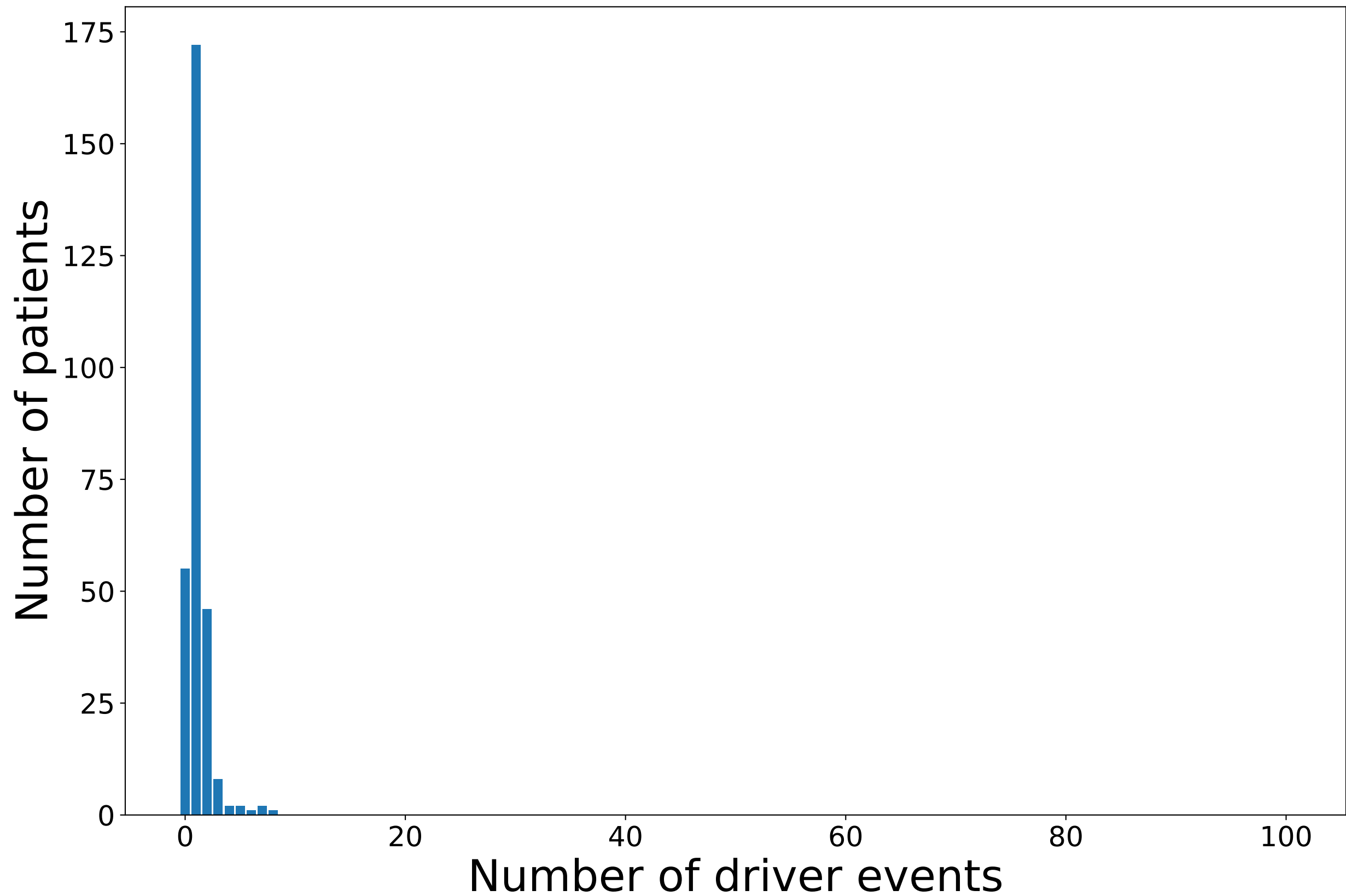

Supplement: S3 Files — (ZIP) [file pgen.1009996.s003.zip › COHORTS/patient distributions/2021_11_23_14_20_THCA_FEMALE.pdf]

# KICH

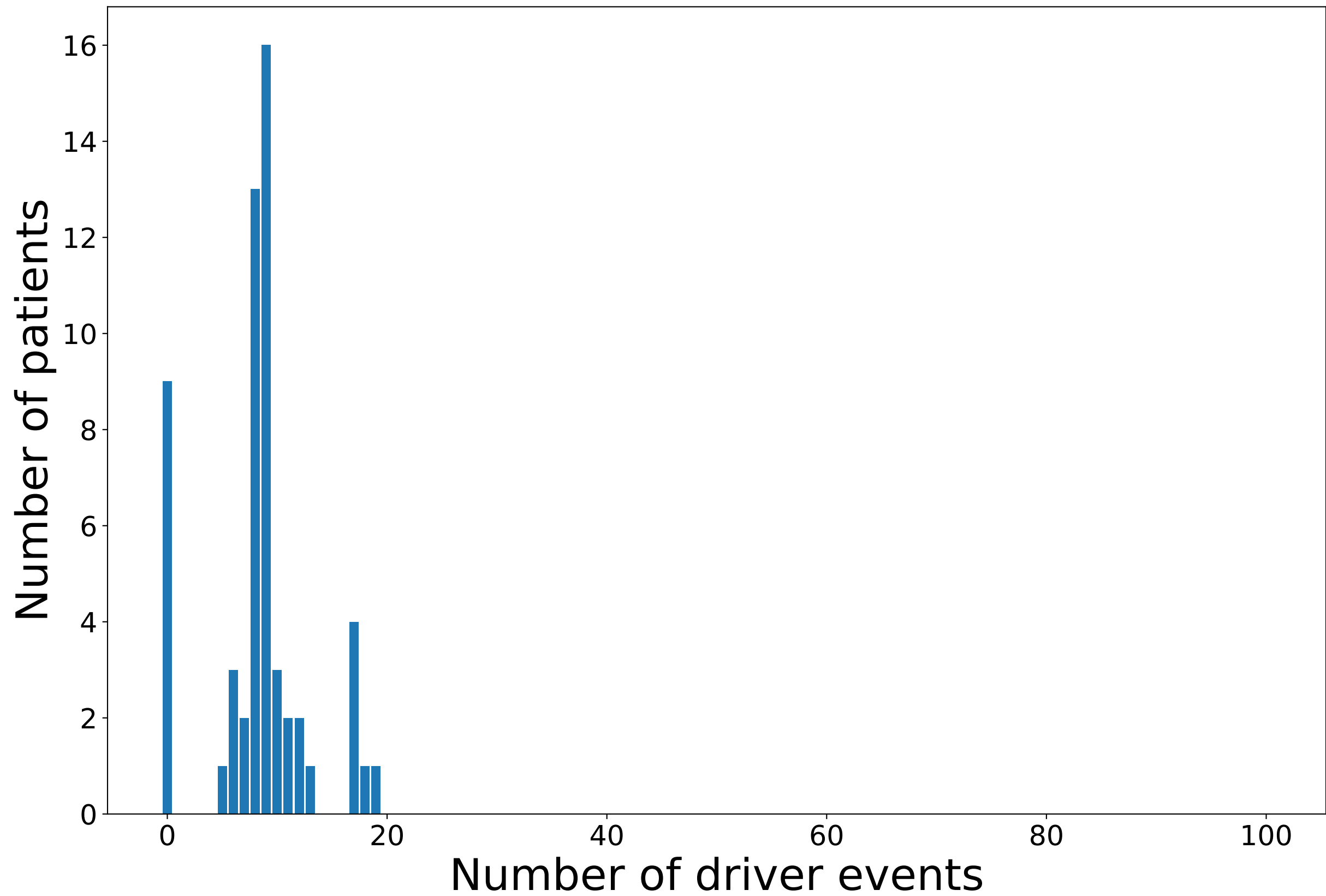

Supplement: S3 Files — (ZIP) [file pgen.1009996.s003.zip › COHORTS/patient distributions/2021_11_23_14_20_KICH.pdf]

# PANCAN\_FEMALE

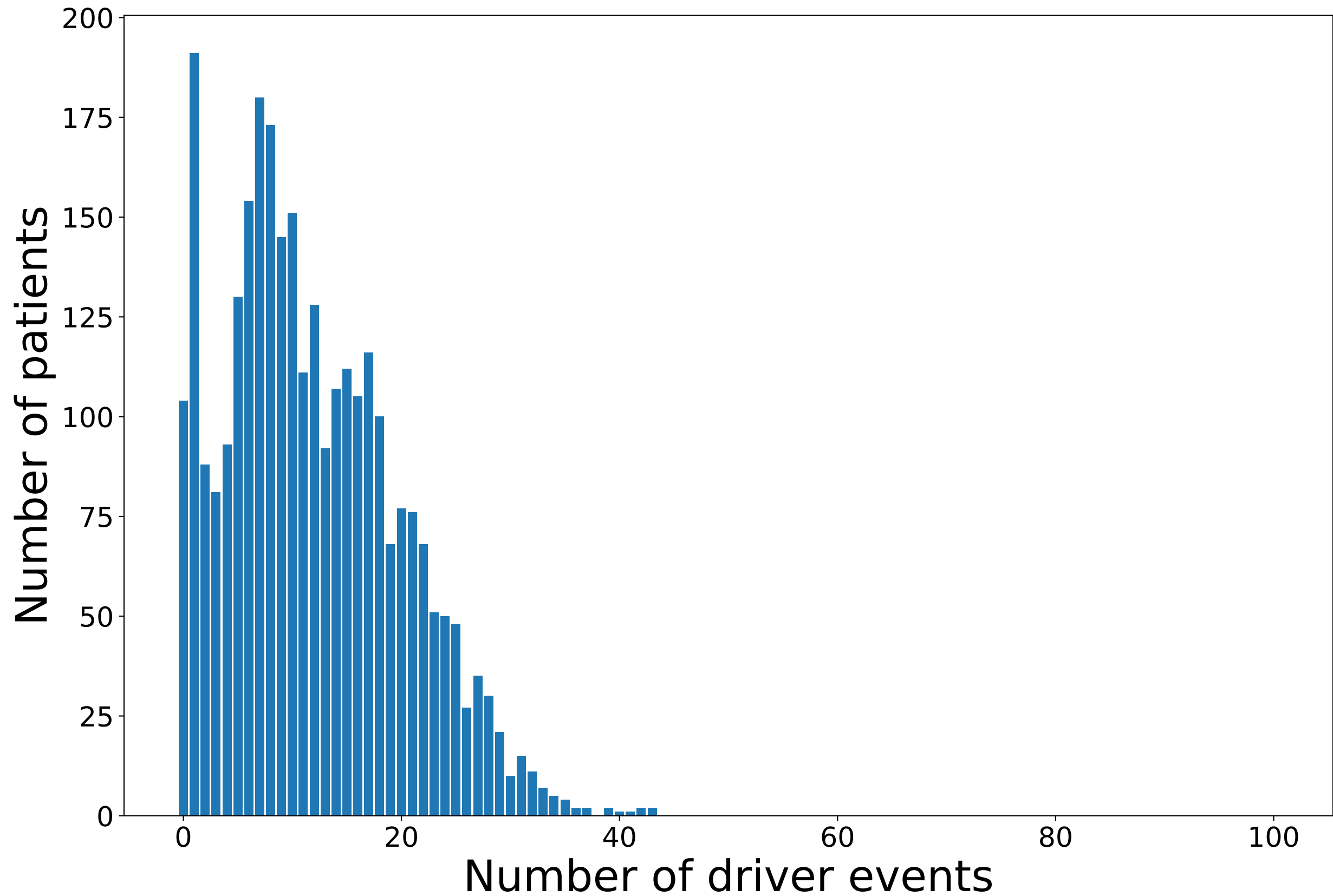

Supplement: S3 Files — (ZIP) [file pgen.1009996.s003.zip › COHORTS/patient distributions/2021_11_23_14_20_PANCAN_FEMALE.pdf]

# STAD

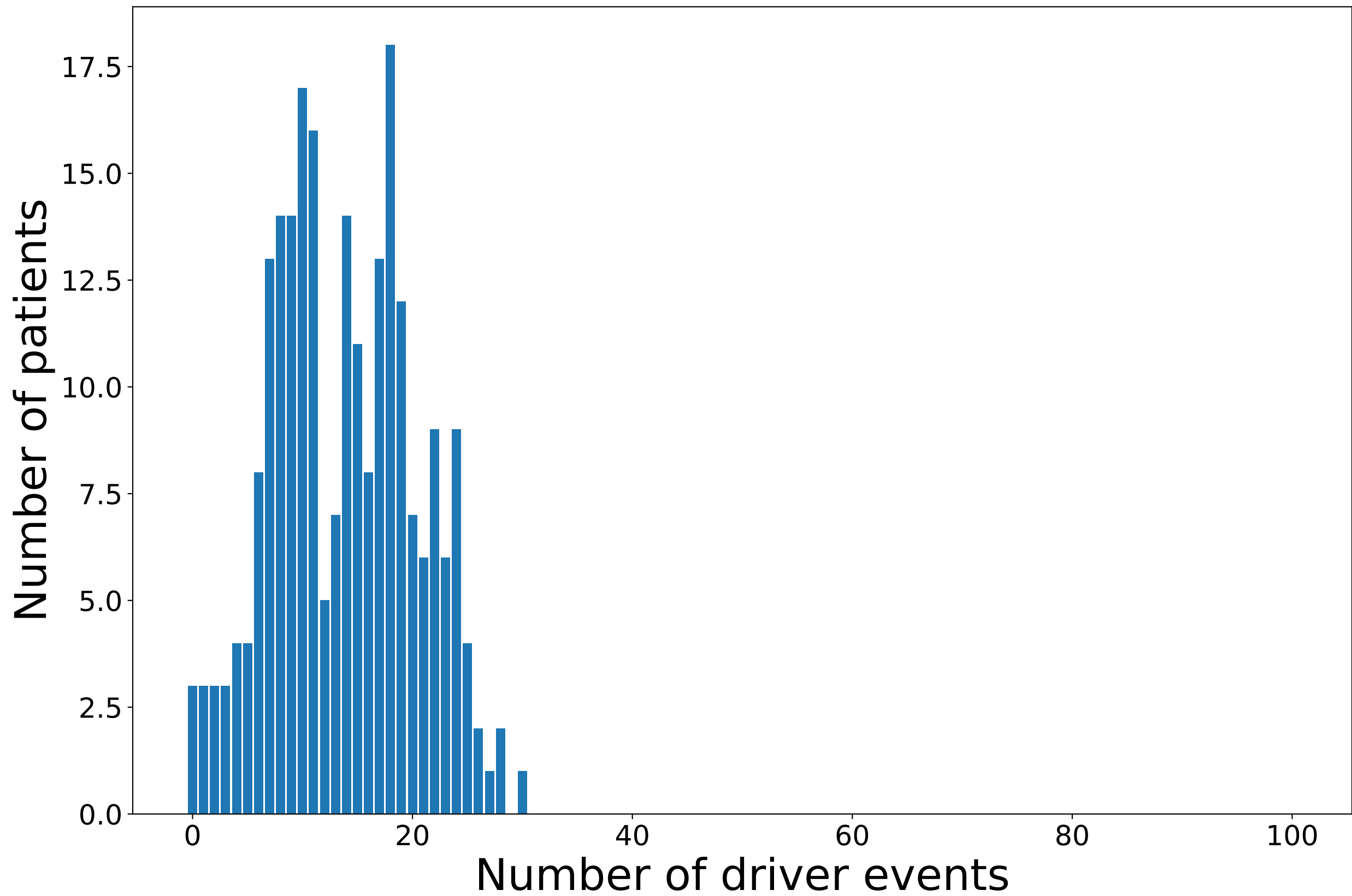

Supplement: S3 Files — (ZIP) [file pgen.1009996.s003.zip › COHORTS/patient distributions/2021_11_23_14_20_STAD.pdf]

# CHOL

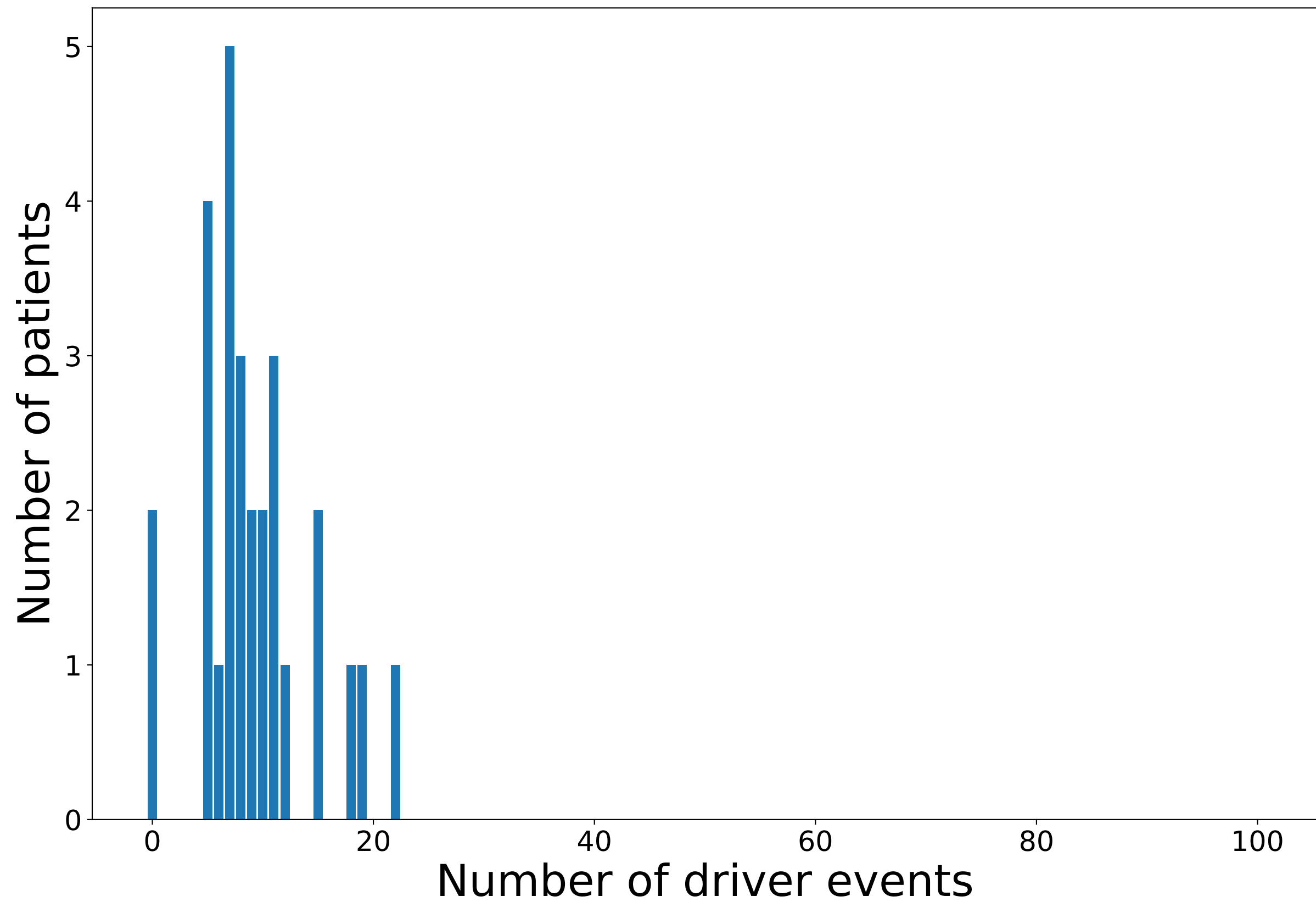

Supplement: S3 Files — (ZIP) [file pgen.1009996.s003.zip › COHORTS/patient distributions/2021_11_23_14_20_CHOL.pdf]

# STAD\_FEMALE

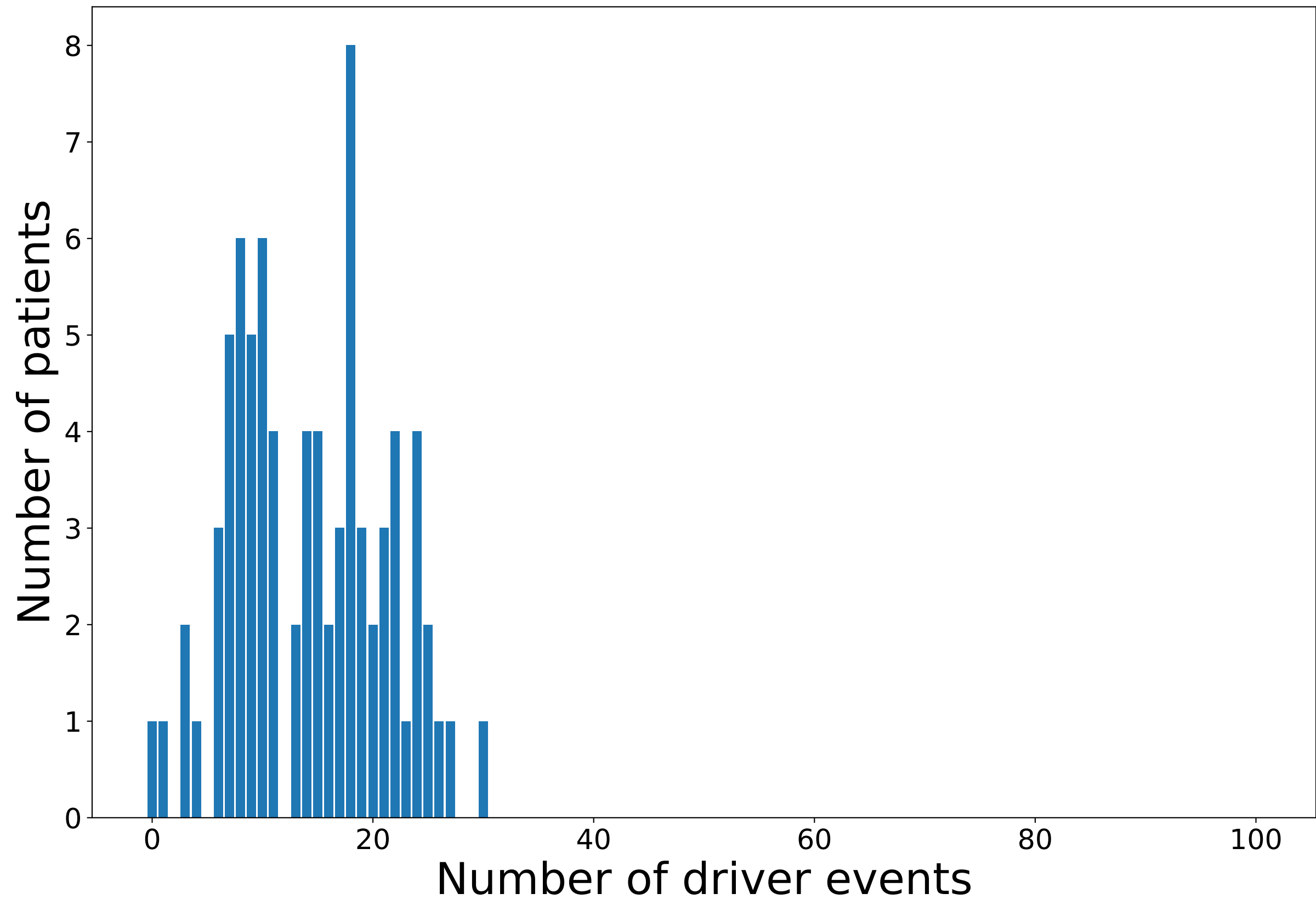

Supplement: S3 Files — (ZIP) [file pgen.1009996.s003.zip › COHORTS/patient distributions/2021_11_23_14_20_STAD_FEMALE.pdf]

Driver event distribution by cancer stage in males READ

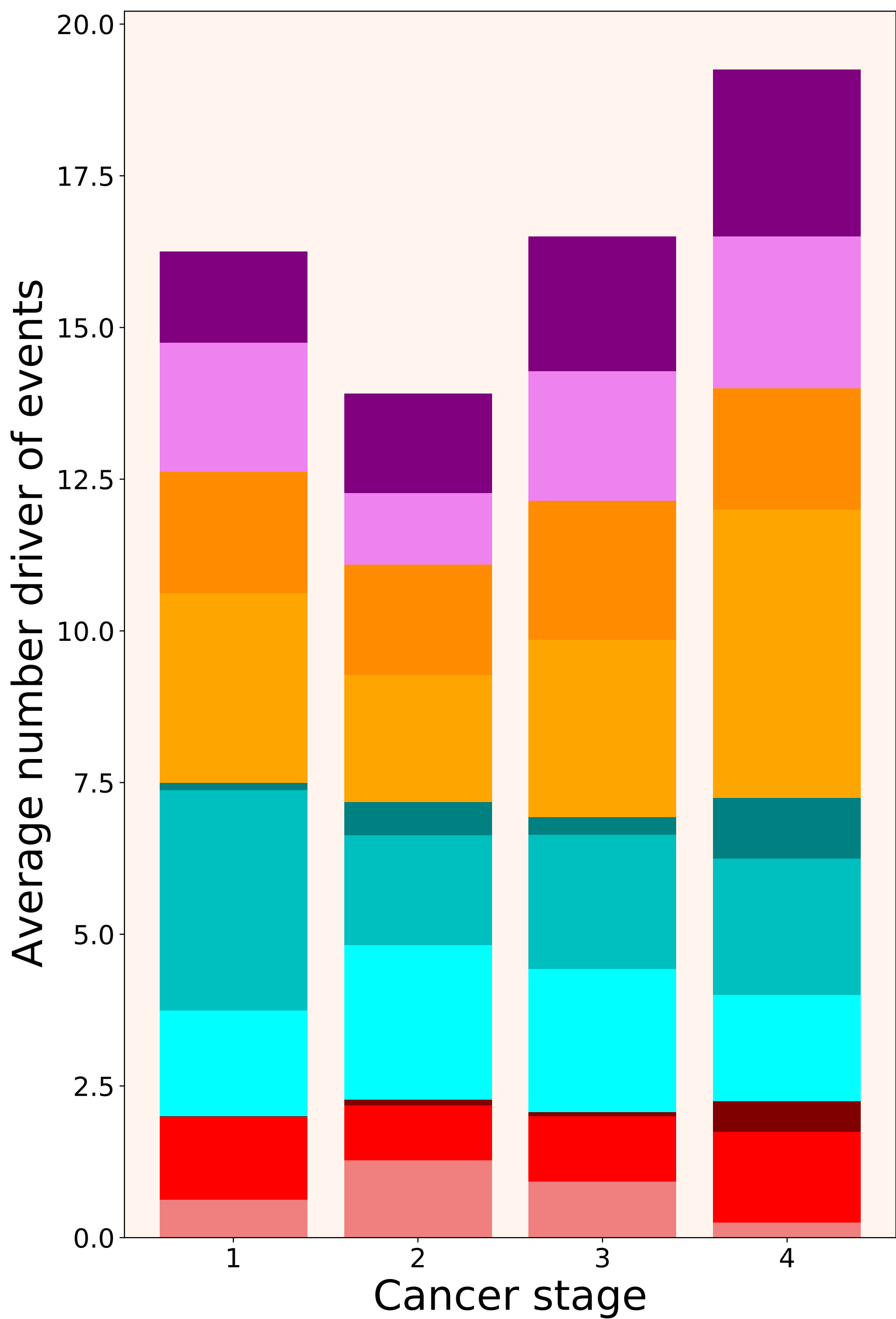

Supplement: S3 Files — (ZIP) [file pgen.1009996.s003.zip › COHORTS/cumulative histograms/Distribution_stages_cohorts/2021_11_23_14_20_distribution_stages_males_READ.pdf]

Driver event distribution by cancer stage in males PAAD

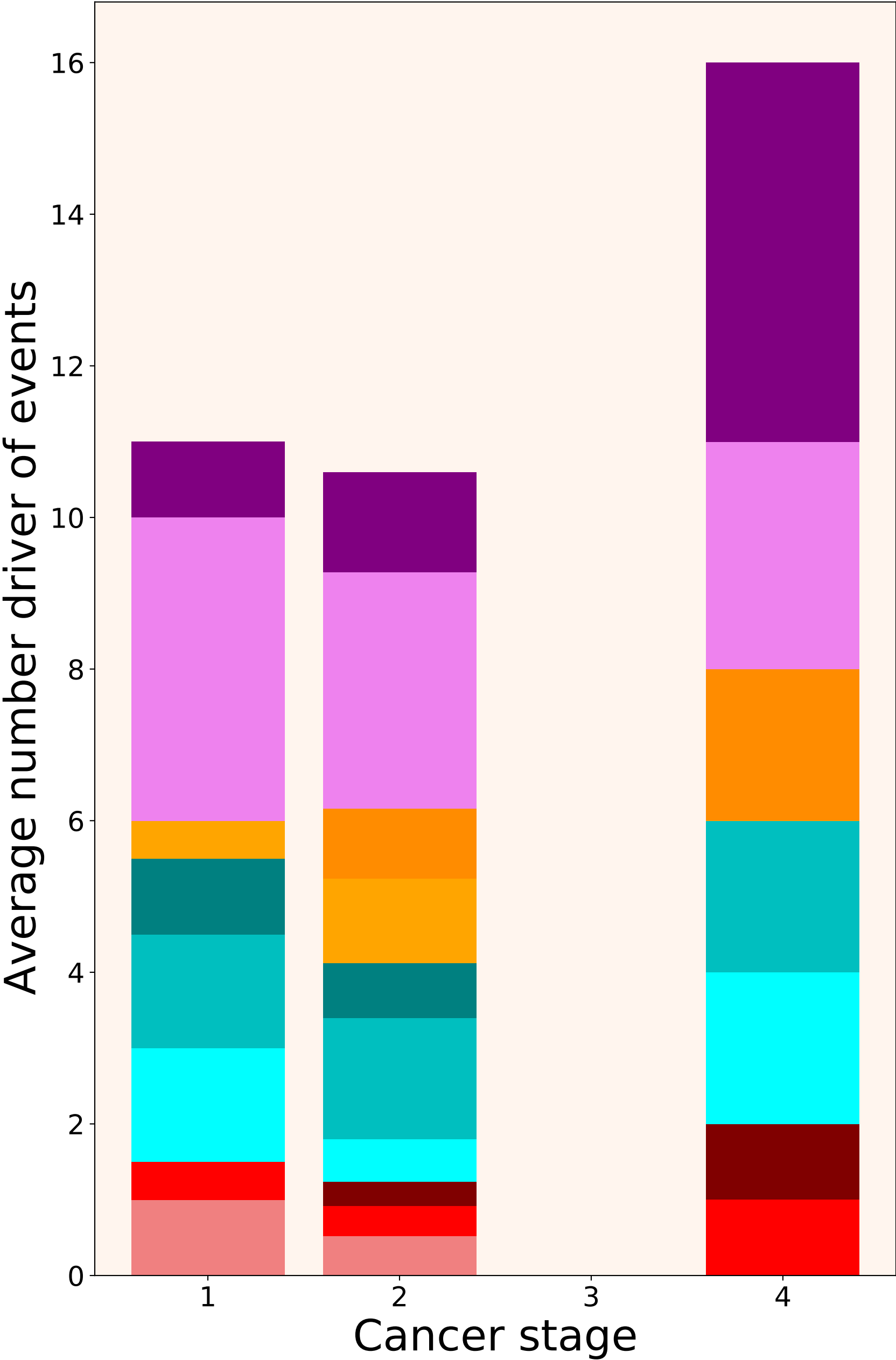

Supplement: S3 Files — (ZIP) [file pgen.1009996.s003.zip › COHORTS/cumulative histograms/Distribution_stages_cohorts/2021_11_23_14_20_distribution_stages_males_PAAD.pdf]

Driver event distribution by cancer stage COAD

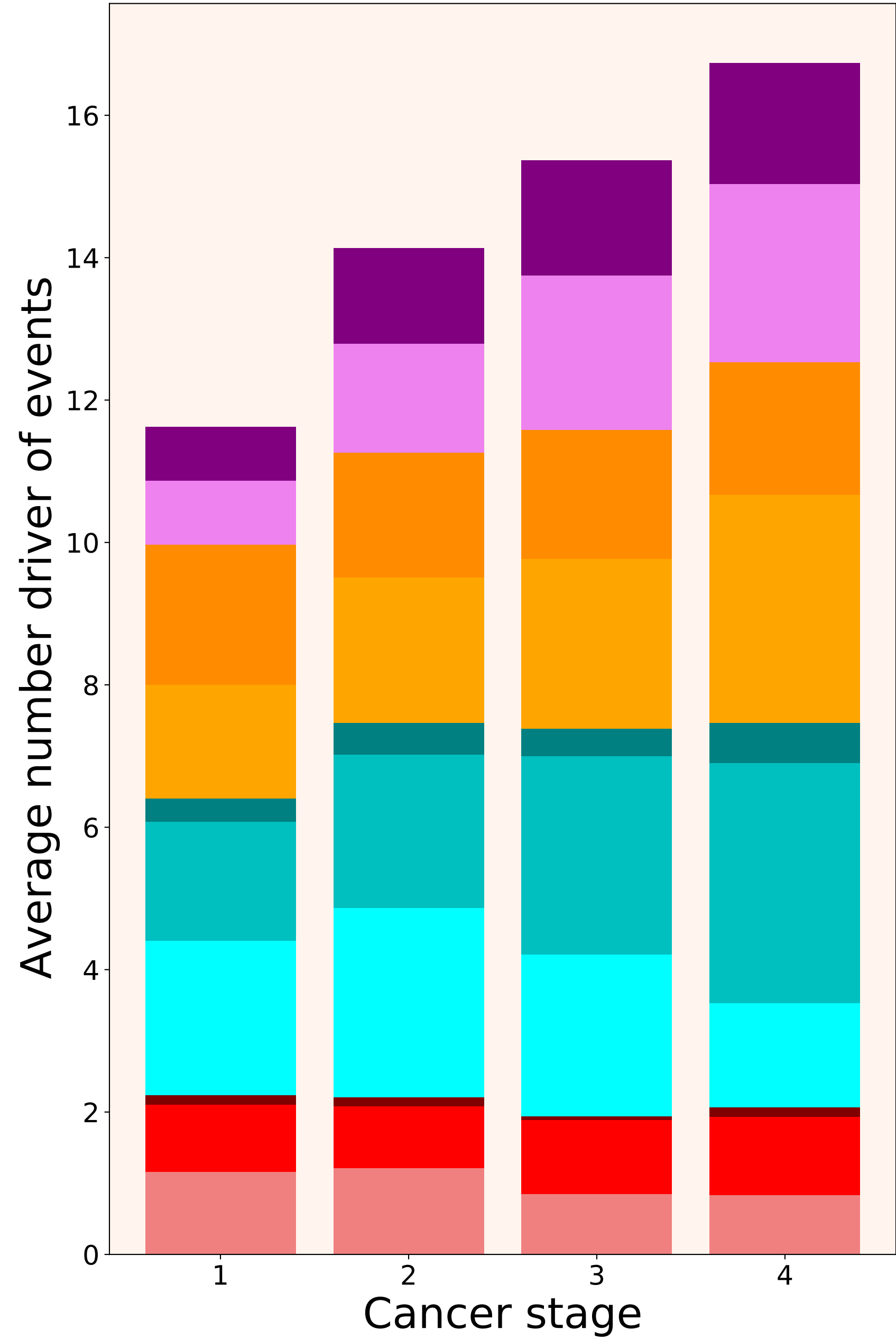

Supplement: S3 Files — (ZIP) [file pgen.1009996.s003.zip › COHORTS/cumulative histograms/Distribution_stages_cohorts/2021_11_23_14_20_distribution_stages_COAD.pdf]

Driver event distribution by cancer stage KIRP

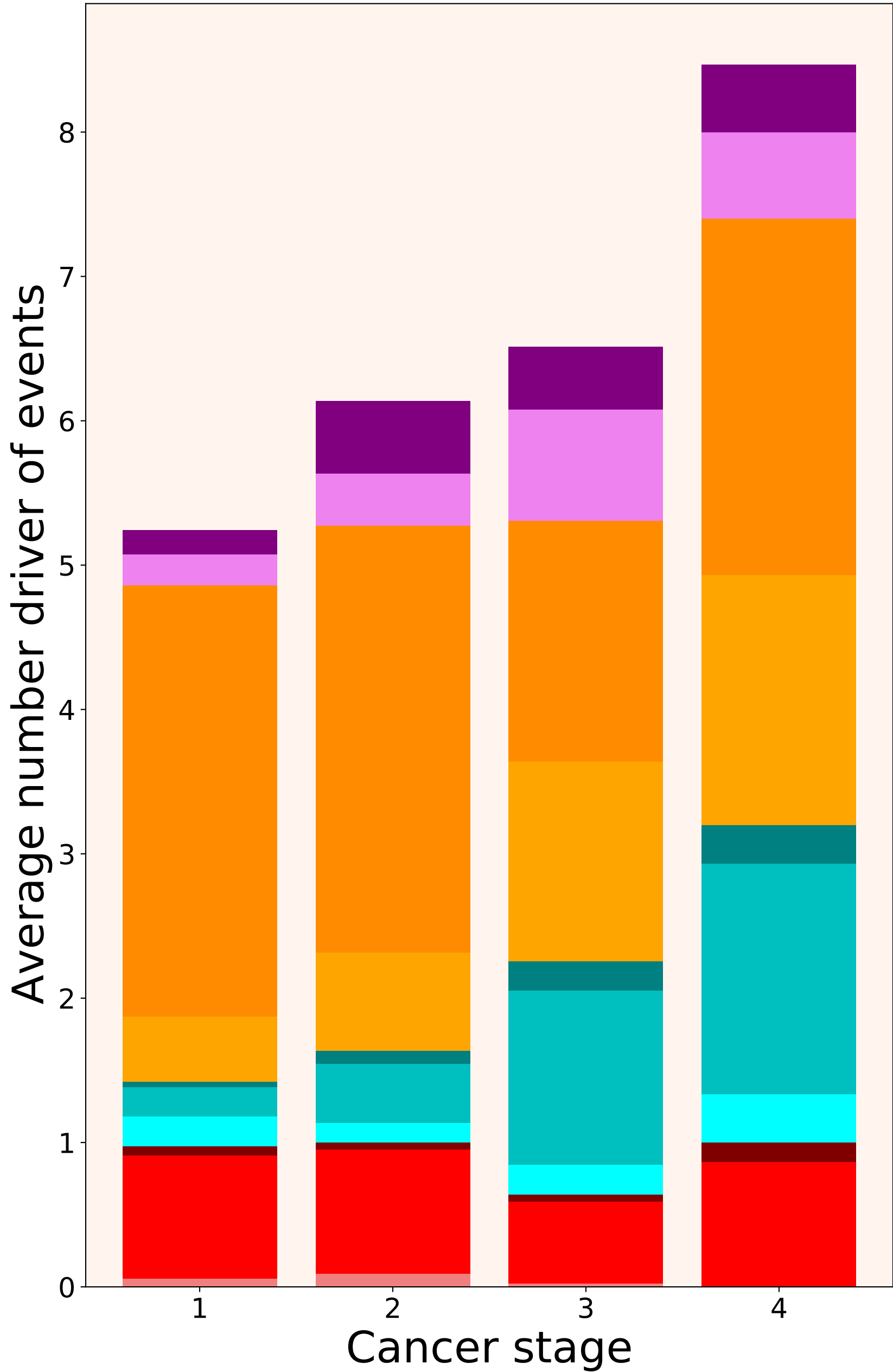

Supplement: S3 Files — (ZIP) [file pgen.1009996.s003.zip › COHORTS/cumulative histograms/Distribution_stages_cohorts/2021_11_23_14_20_distribution_stages_KIRP.pdf]

Driver event distribution by cancer stage in males THCA

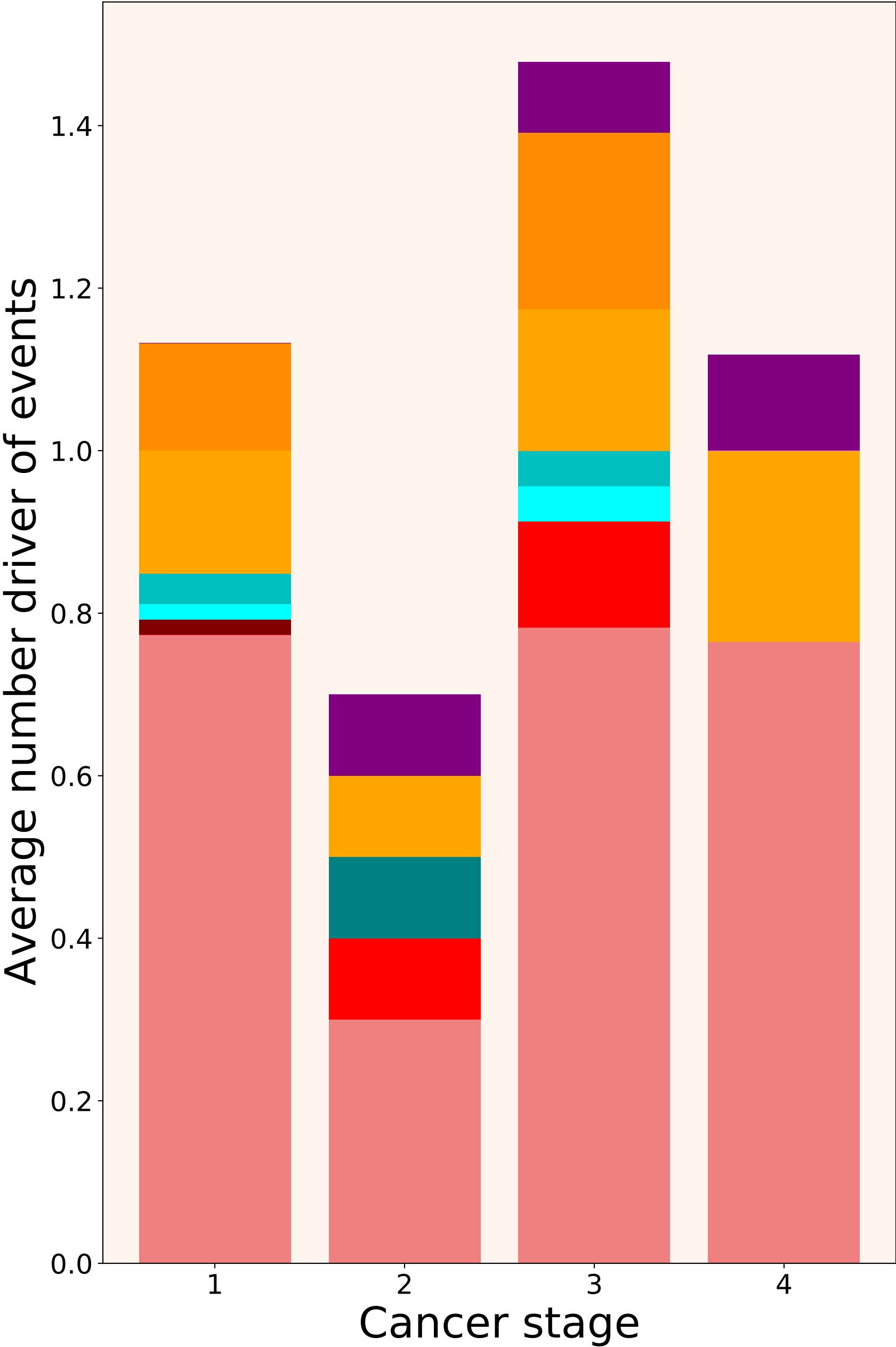

Supplement: S3 Files — (ZIP) [file pgen.1009996.s003.zip › COHORTS/cumulative histograms/Distribution_stages_cohorts/2021_11_23_14_20_distribution_stages_males_THCA.pdf]

Driver event distribution by cancer stage in males BLCA

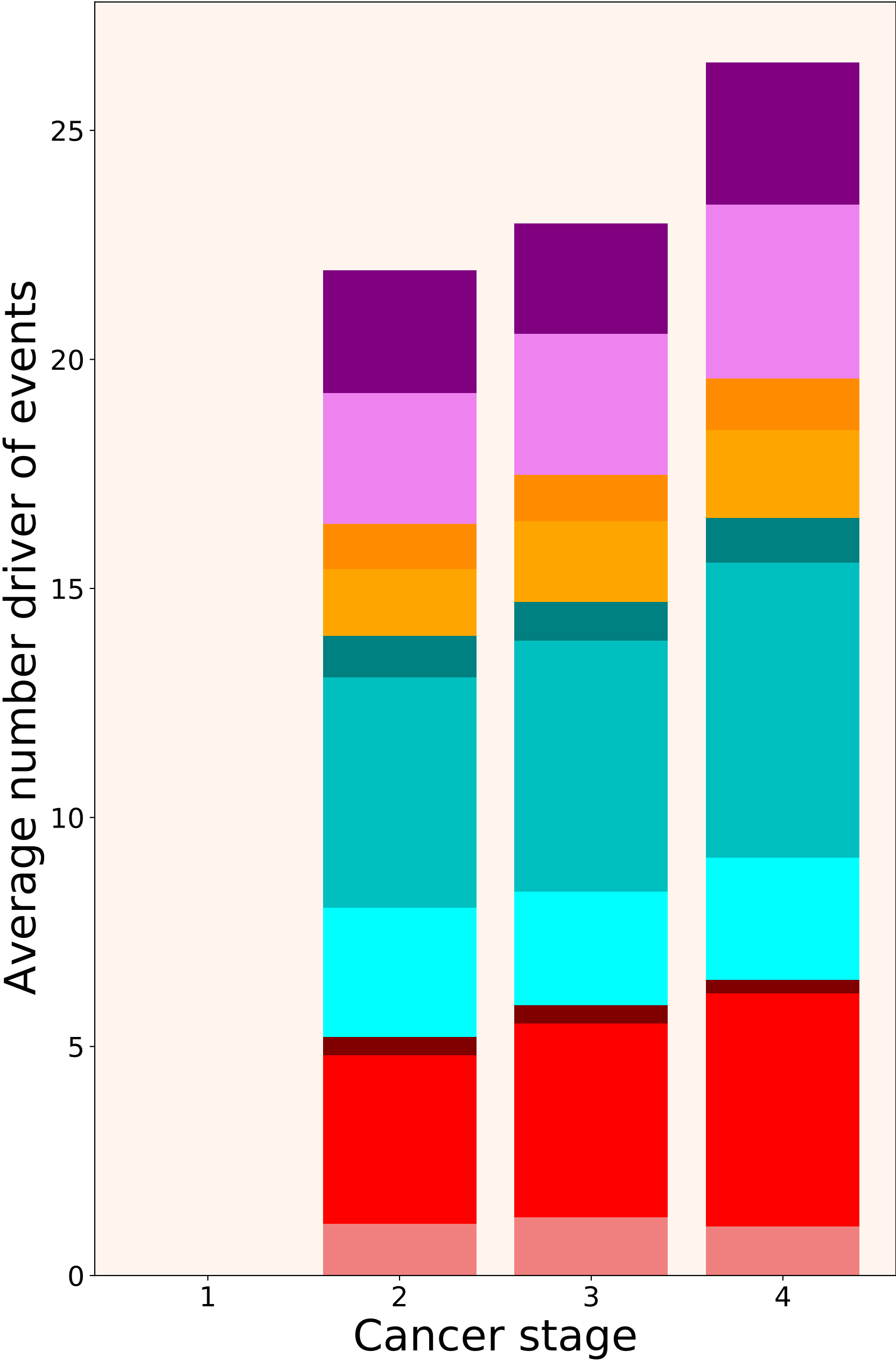

Supplement: S3 Files — (ZIP) [file pgen.1009996.s003.zip › COHORTS/cumulative histograms/Distribution_stages_cohorts/2021_11_23_14_20_distribution_stages_males_BLCA.pdf]

Driver event distribution by cancer stage UVM

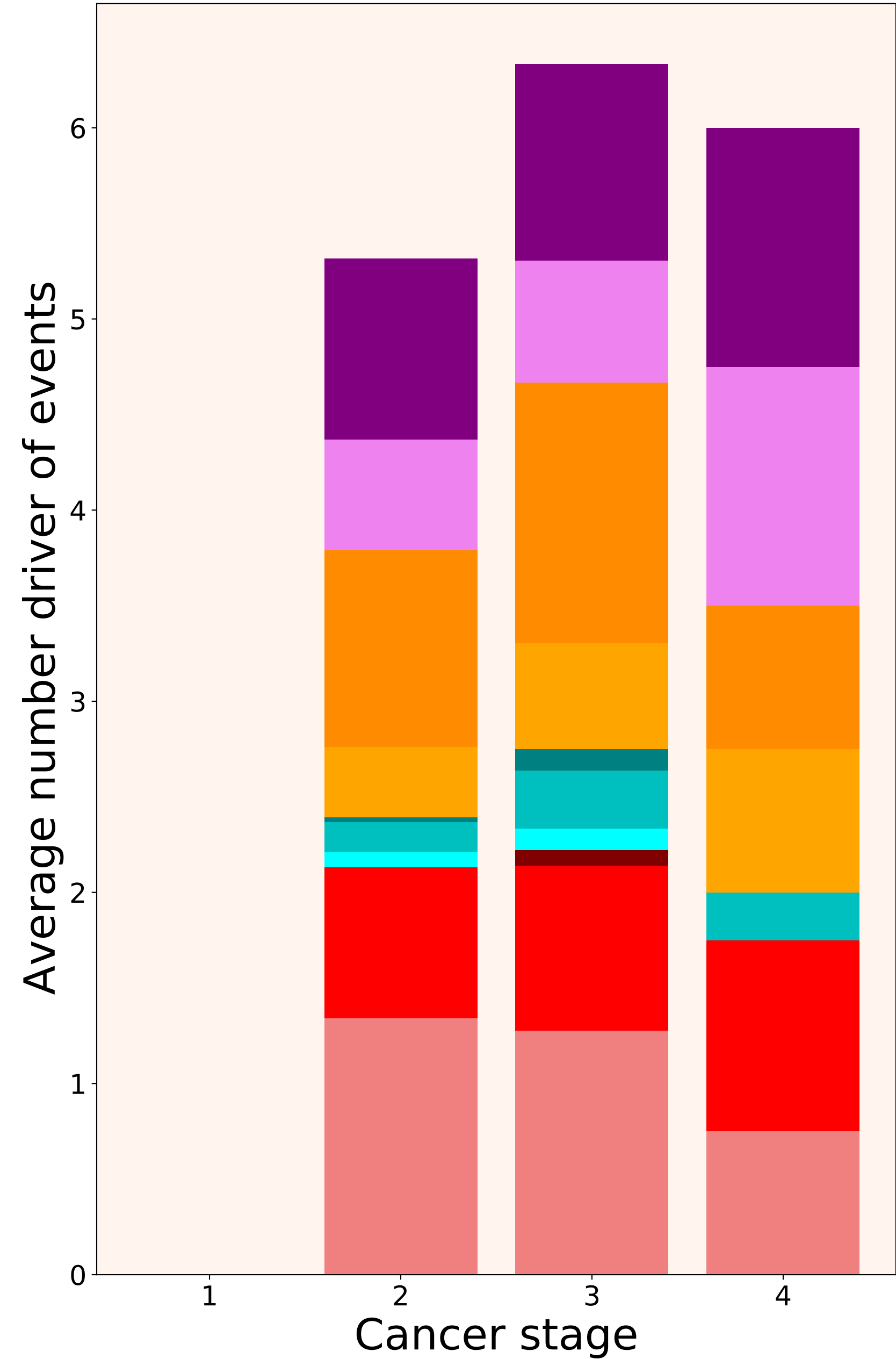

Supplement: S3 Files — (ZIP) [file pgen.1009996.s003.zip › COHORTS/cumulative histograms/Distribution_stages_cohorts/2021_11_23_14_20_distribution_stages_UVM.pdf]

Driver event distribution by cancer stage in females CESC

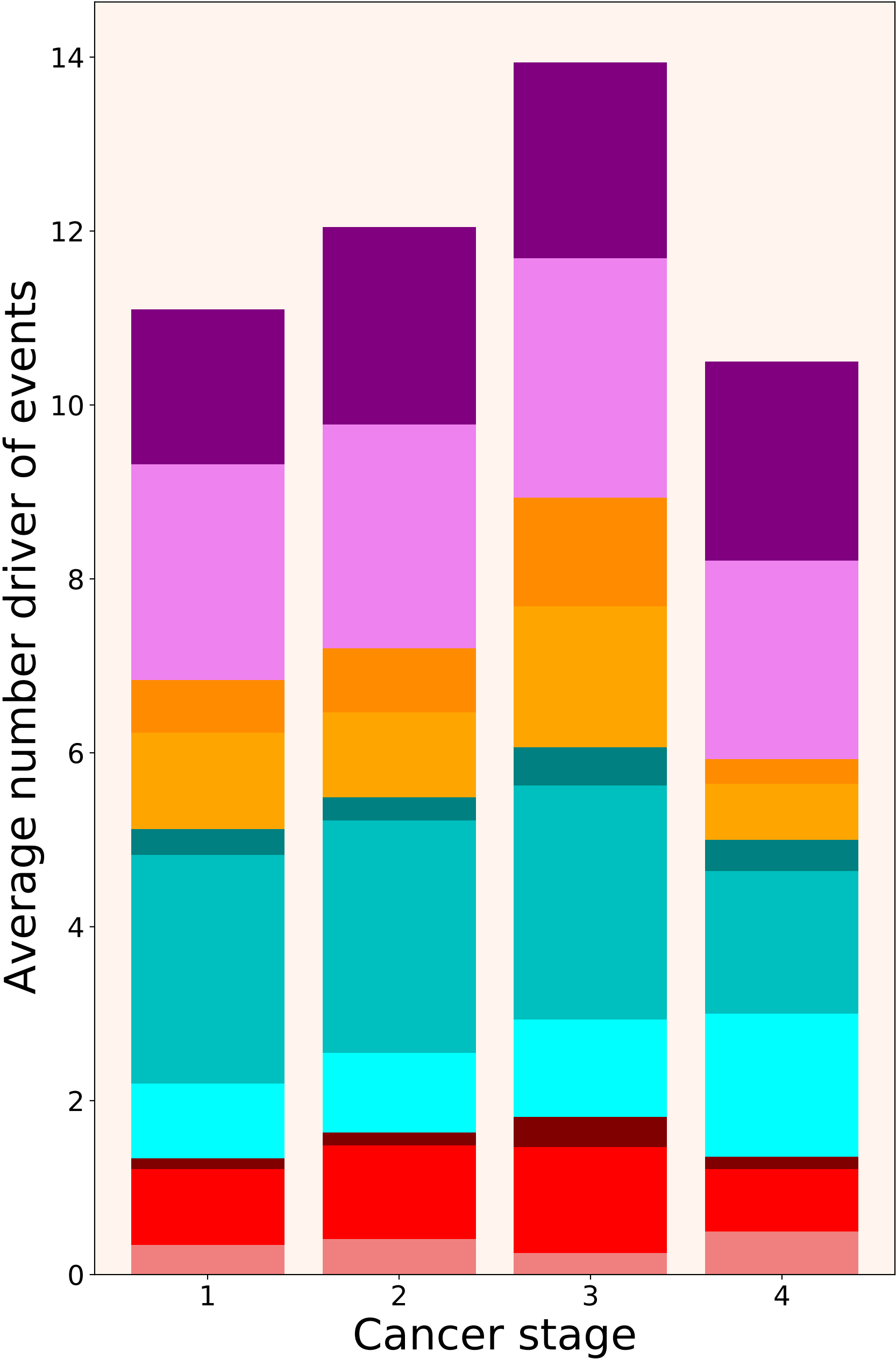

Supplement: S3 Files — (ZIP) [file pgen.1009996.s003.zip › COHORTS/cumulative histograms/Distribution_stages_cohorts/2021_11_23_14_20_distribution_stages_females_CESC.pdf]

Driver event distribution by cancer stage LIHC

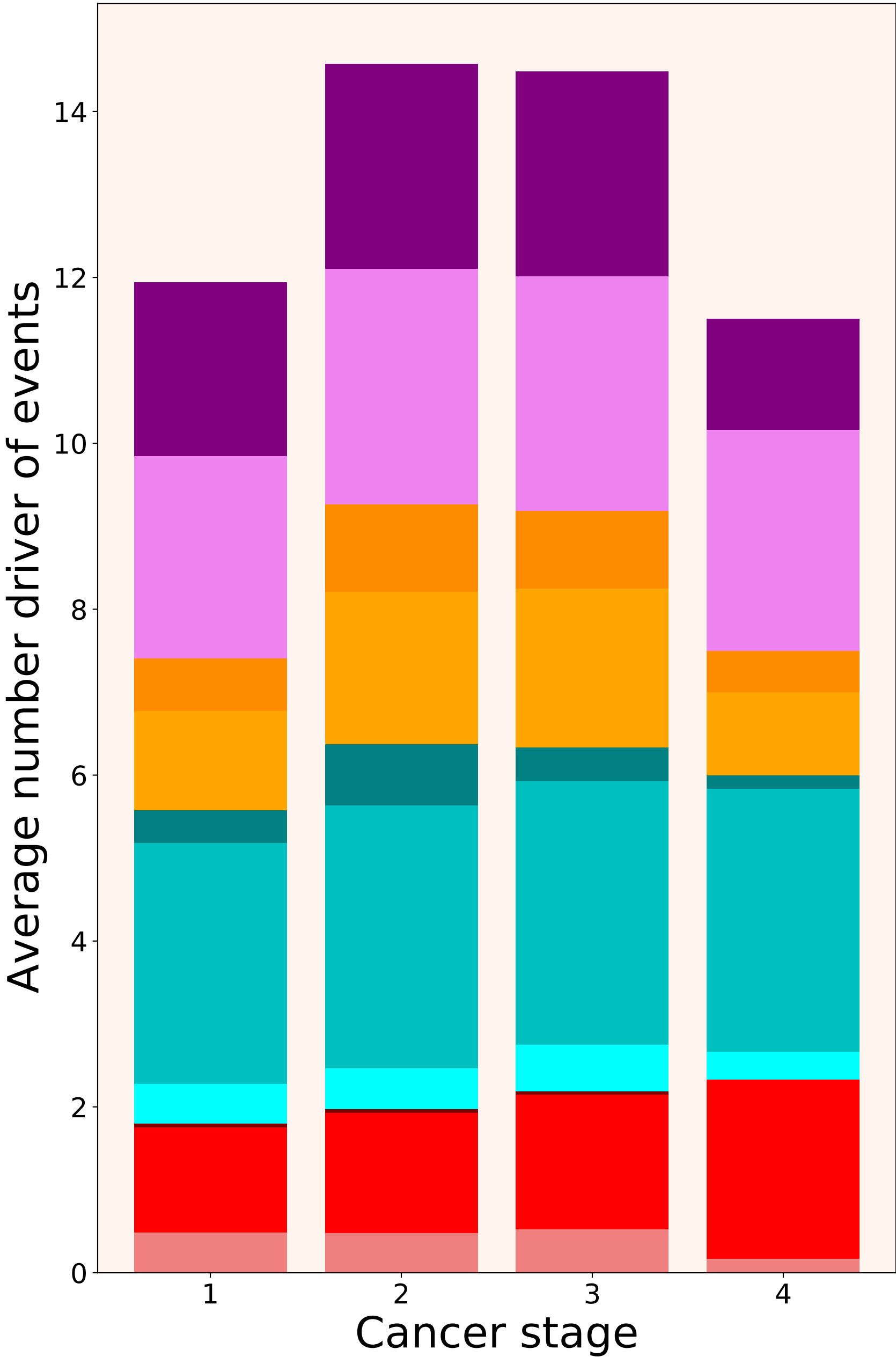

Supplement: S3 Files — (ZIP) [file pgen.1009996.s003.zip › COHORTS/cumulative histograms/Distribution_stages_cohorts/2021_11_23_14_20_distribution_stages_LIHC.pdf]

Driver event distribution by cancer stage in males KIRC

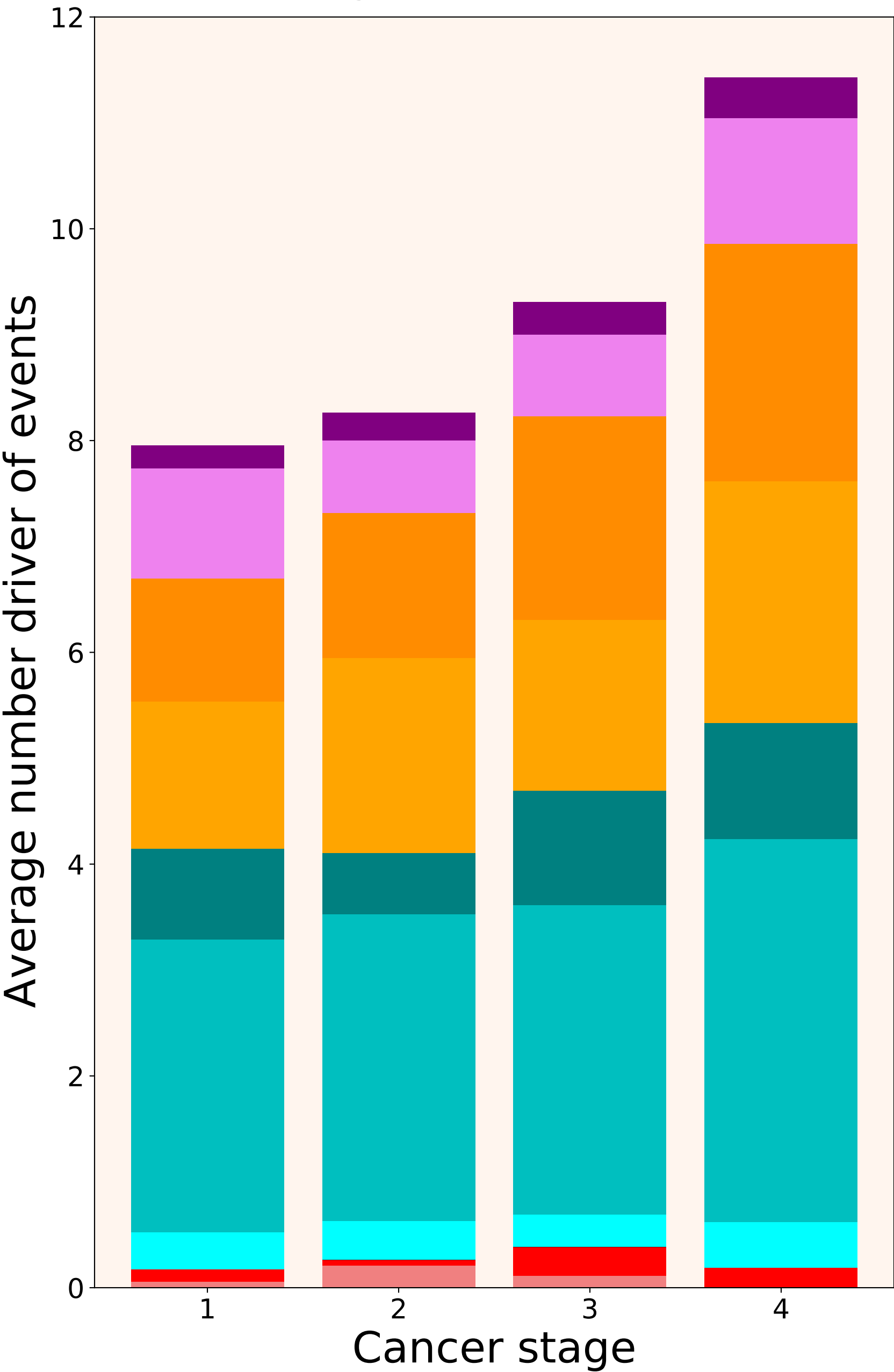

Supplement: S3 Files — (ZIP) [file pgen.1009996.s003.zip › COHORTS/cumulative histograms/Distribution_stages_cohorts/2021_11_23_14_20_distribution_stages_males_KIRC.pdf]

Driver event distribution by cancer stage THCA

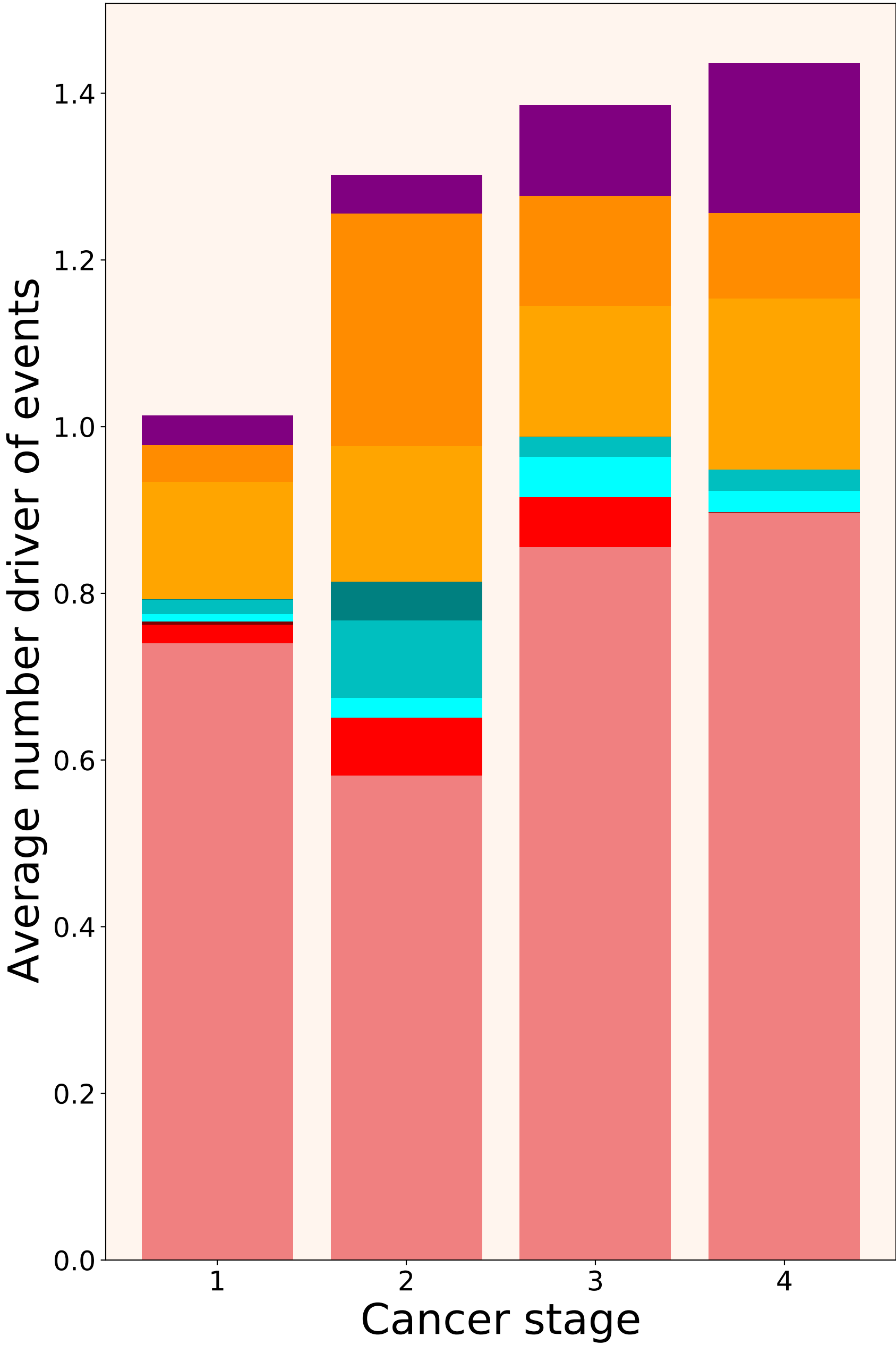

Supplement: S3 Files — (ZIP) [file pgen.1009996.s003.zip › COHORTS/cumulative histograms/Distribution_stages_cohorts/2021_11_23_14_20_distribution_stages_THCA.pdf]

Driver event distribution by cancer stage PAAD

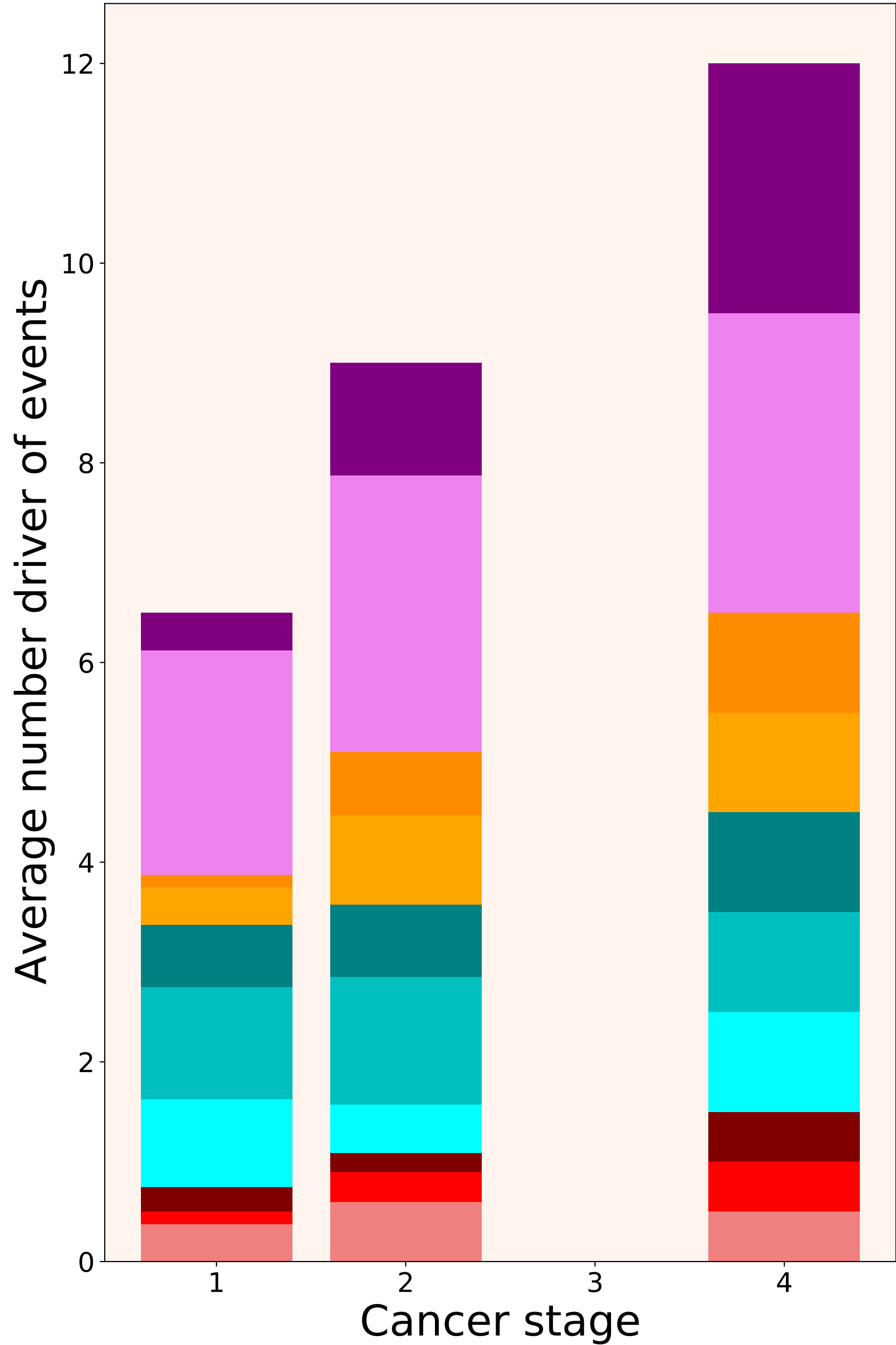

Supplement: S3 Files — (ZIP) [file pgen.1009996.s003.zip › COHORTS/cumulative histograms/Distribution_stages_cohorts/2021_11_23_14_20_distribution_stages_PAAD.pdf]

Driver event distribution by cancer stage in males COAD

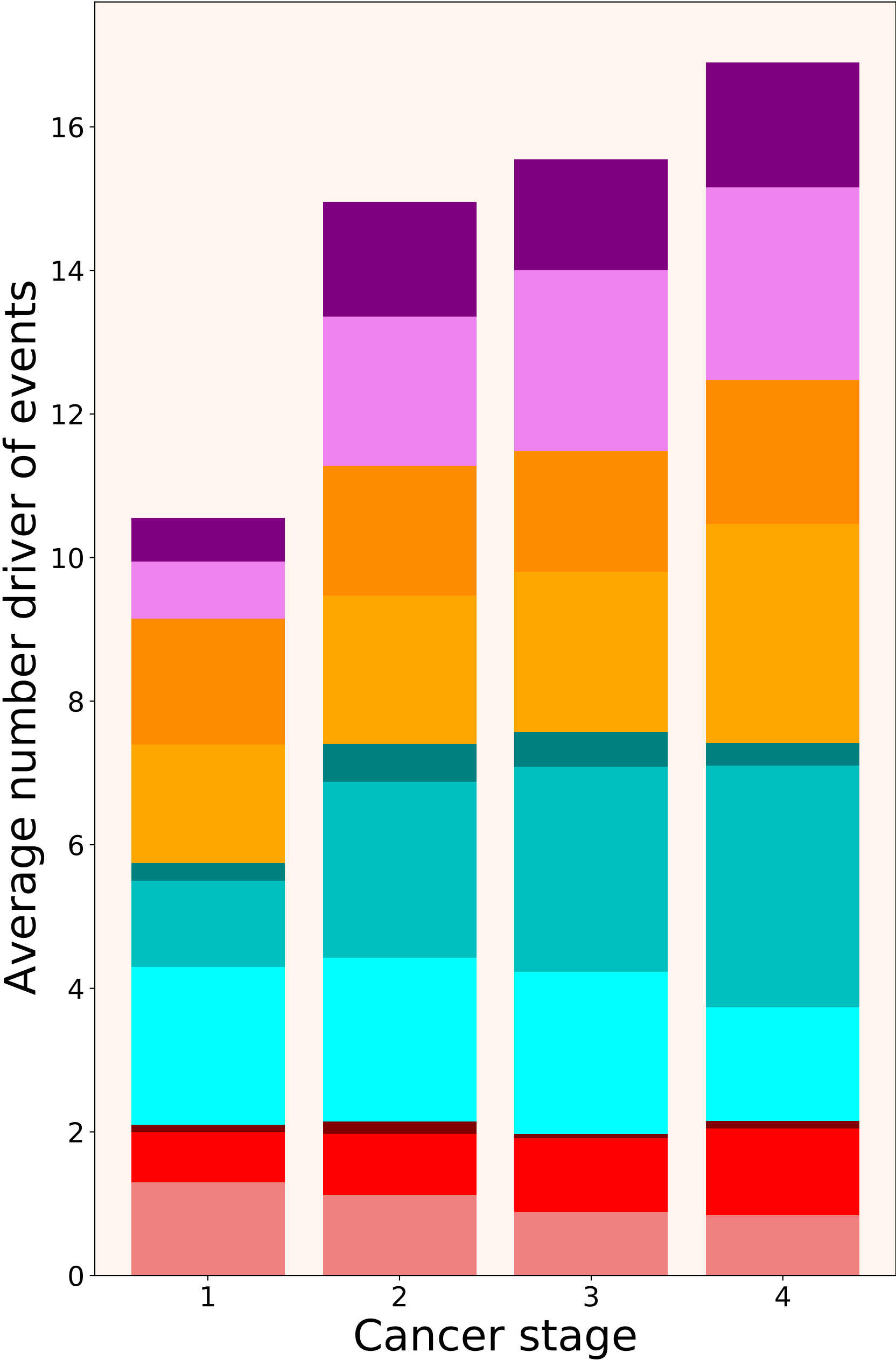

Supplement: S3 Files — (ZIP) [file pgen.1009996.s003.zip › COHORTS/cumulative histograms/Distribution_stages_cohorts/2021_11_23_14_20_distribution_stages_males_COAD.pdf]

Driver event distribution by cancer stage in males KIRP

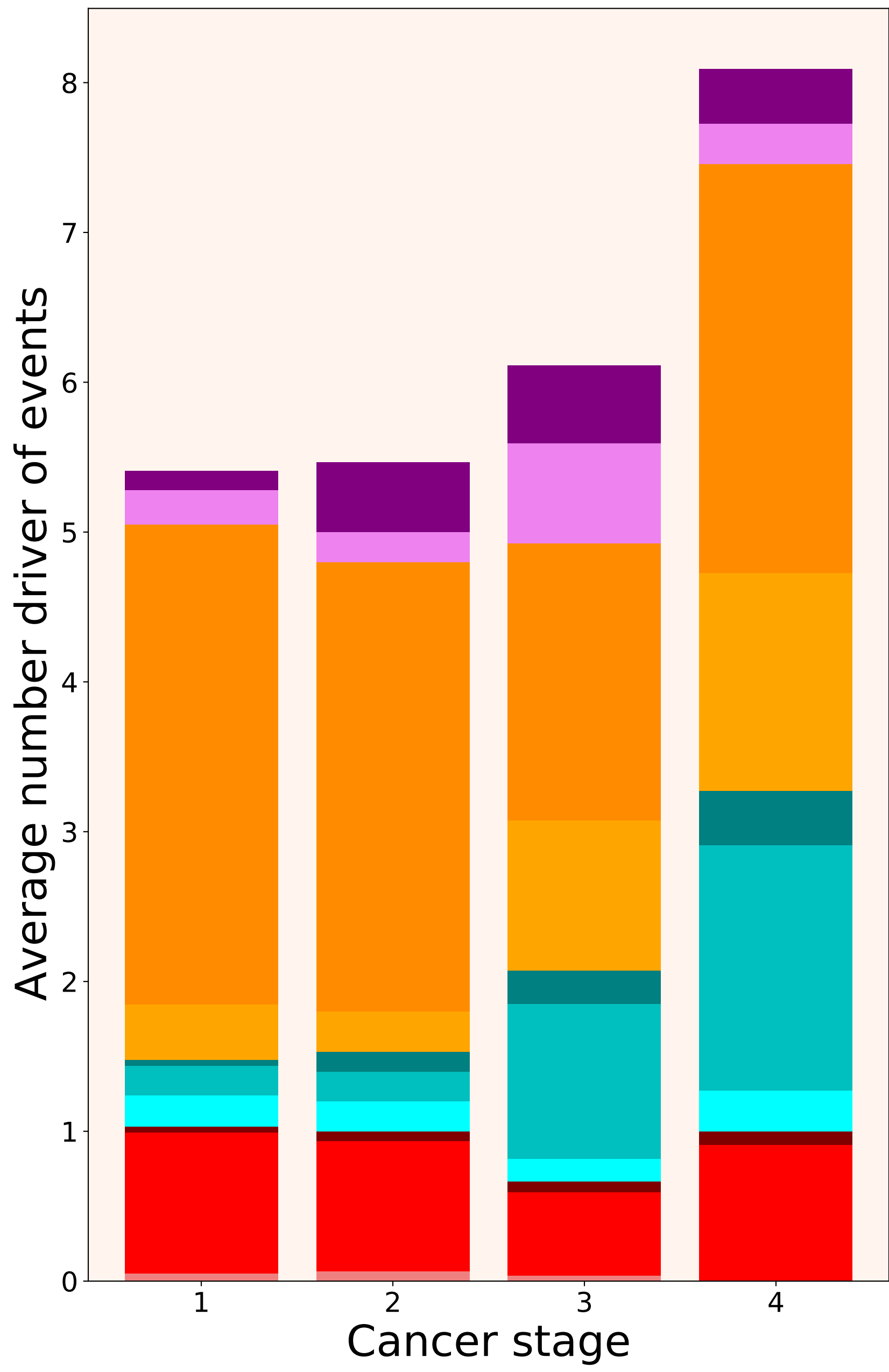

Supplement: S3 Files — (ZIP) [file pgen.1009996.s003.zip › COHORTS/cumulative histograms/Distribution_stages_cohorts/2021_11_23_14_20_distribution_stages_males_KIRP.pdf]

Driver event distribution by cancer stage BLCA

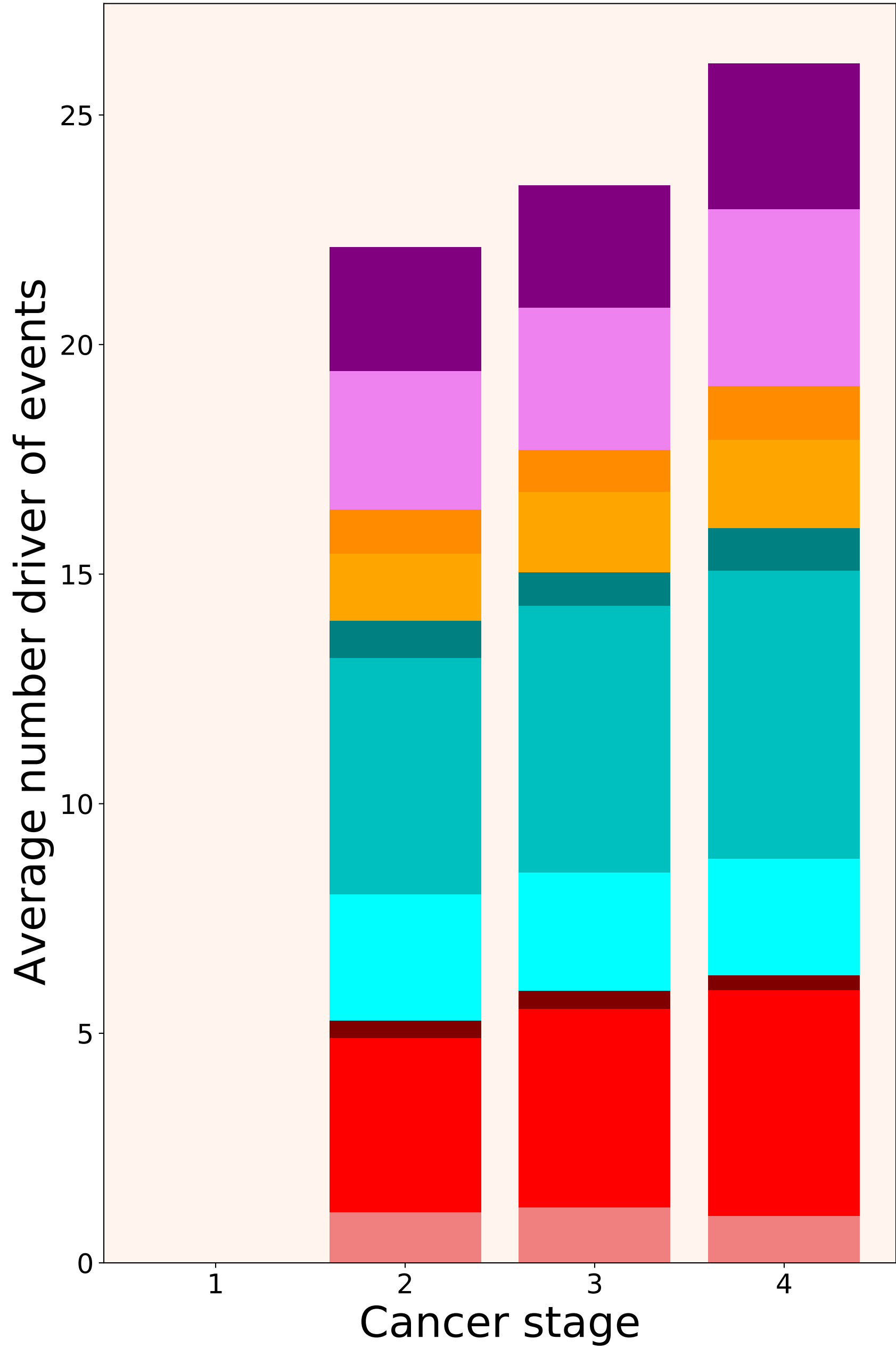

Supplement: S3 Files — (ZIP) [file pgen.1009996.s003.zip › COHORTS/cumulative histograms/Distribution_stages_cohorts/2021_11_23_14_20_distribution_stages_BLCA.pdf]

Driver event distribution by cancer stage READ

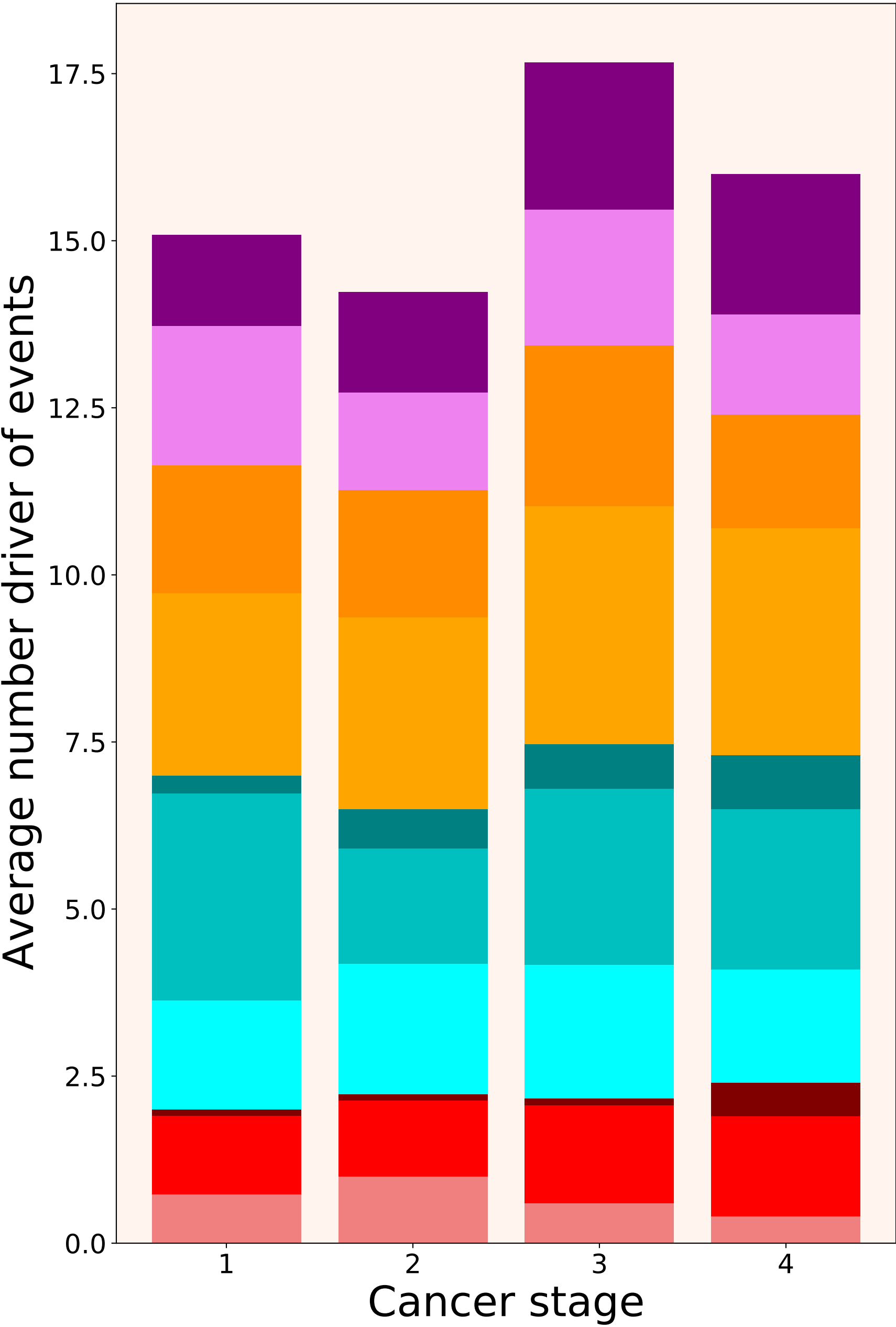

Supplement: S3 Files — (ZIP) [file pgen.1009996.s003.zip › COHORTS/cumulative histograms/Distribution_stages_cohorts/2021_11_23_14_20_distribution_stages_READ.pdf]
