# Supplementary figures and images for: Comprehensive patient-level classification and quantification of driver events in TCGA PanCanAtlas cohorts (part 6 of 6)
Source: PLoS Genet. 2022 Jan 14;18(1):e1009996. doi: 10.1371/journal.pgen.1009996 (PMC8759692; doi:10.1371/journal.pgen.1009996)

# BLCA\_FEMALE

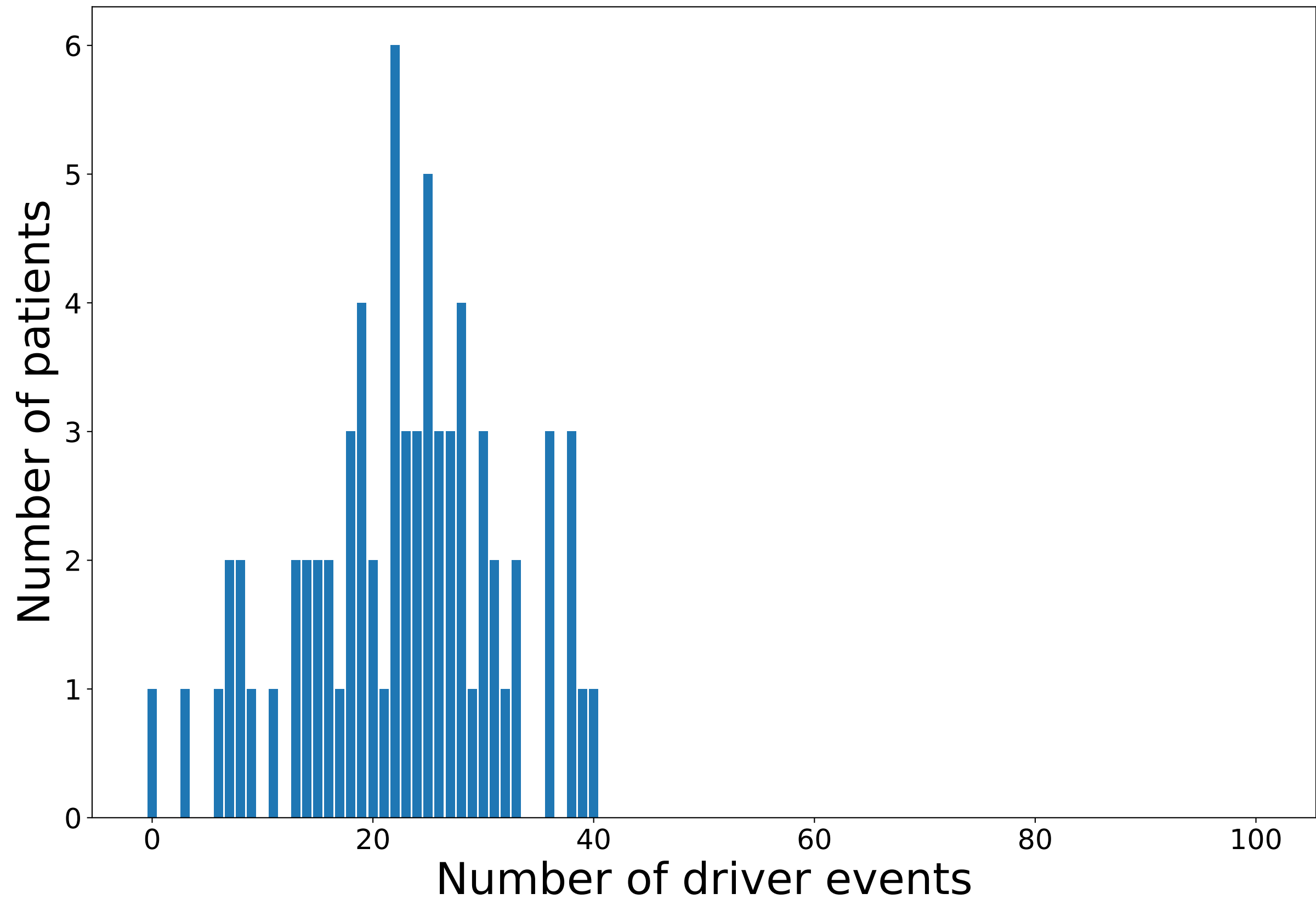

Supplement: S4 Files — (ZIP) [file pgen.1009996.s004.zip › Aneuploidy/COHORTS GISTIC2/patient distributions/2021_11_23_15_0_BLCA_FEMALE.pdf]

# UCS\_FEMALE

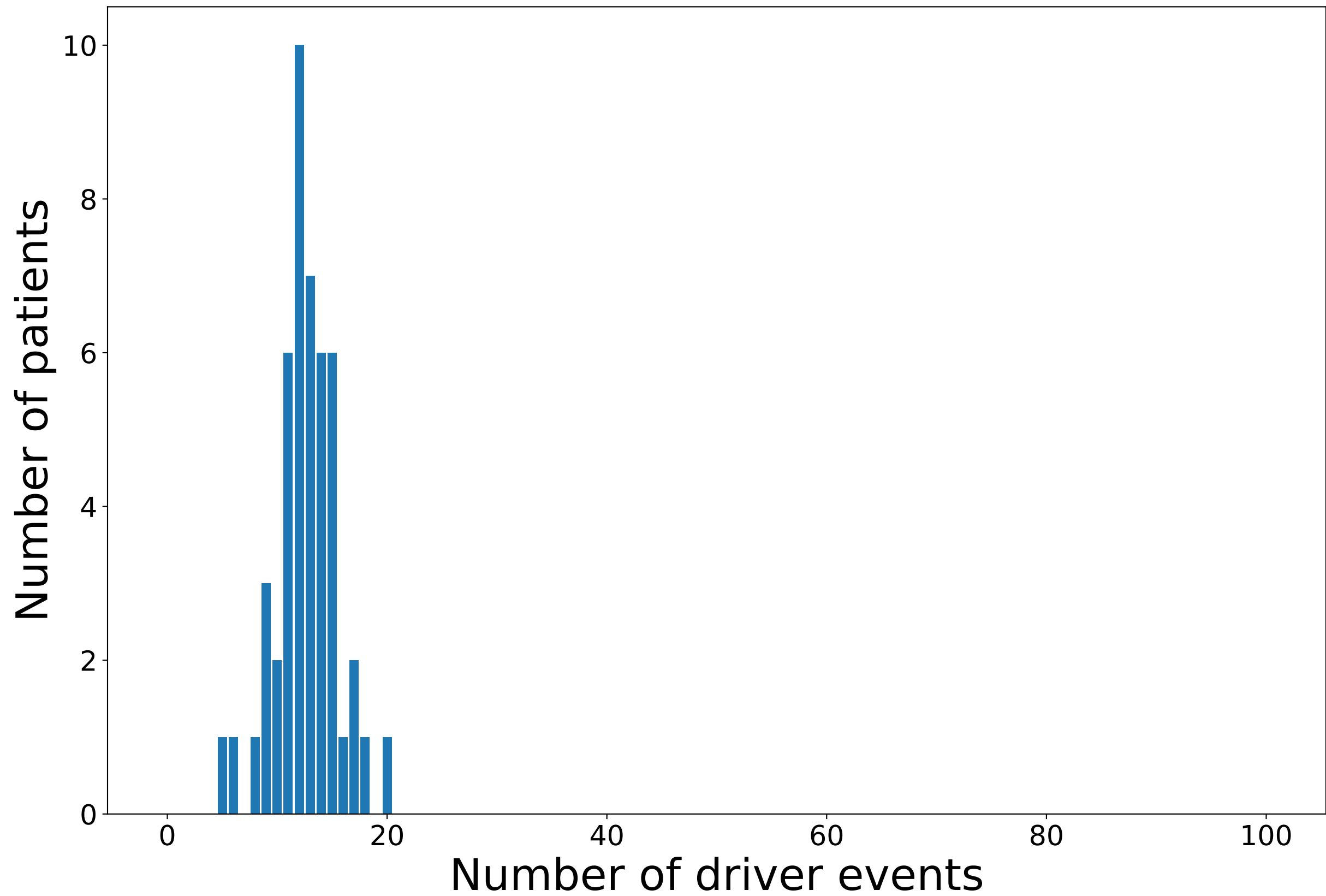

Supplement: S4 Files — (ZIP) [file pgen.1009996.s004.zip › Aneuploidy/COHORTS GISTIC2/patient distributions/2021_11_23_15_0_UCS_FEMALE.pdf]

# UVM\_MALE

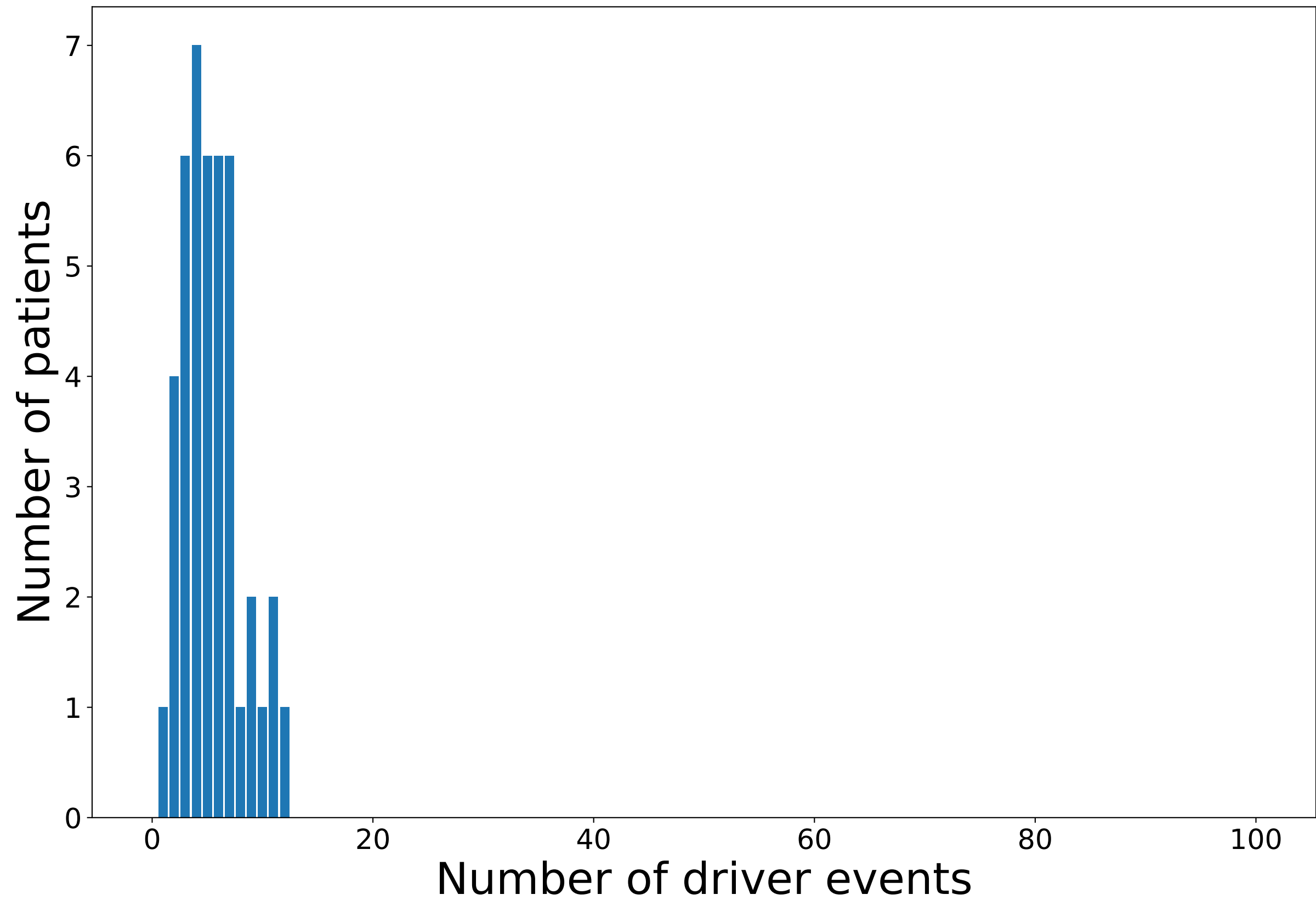

Supplement: S4 Files — (ZIP) [file pgen.1009996.s004.zip › Aneuploidy/COHORTS GISTIC2/patient distributions/2021_11_23_15_0_UVM_MALE.pdf]

# PCPG

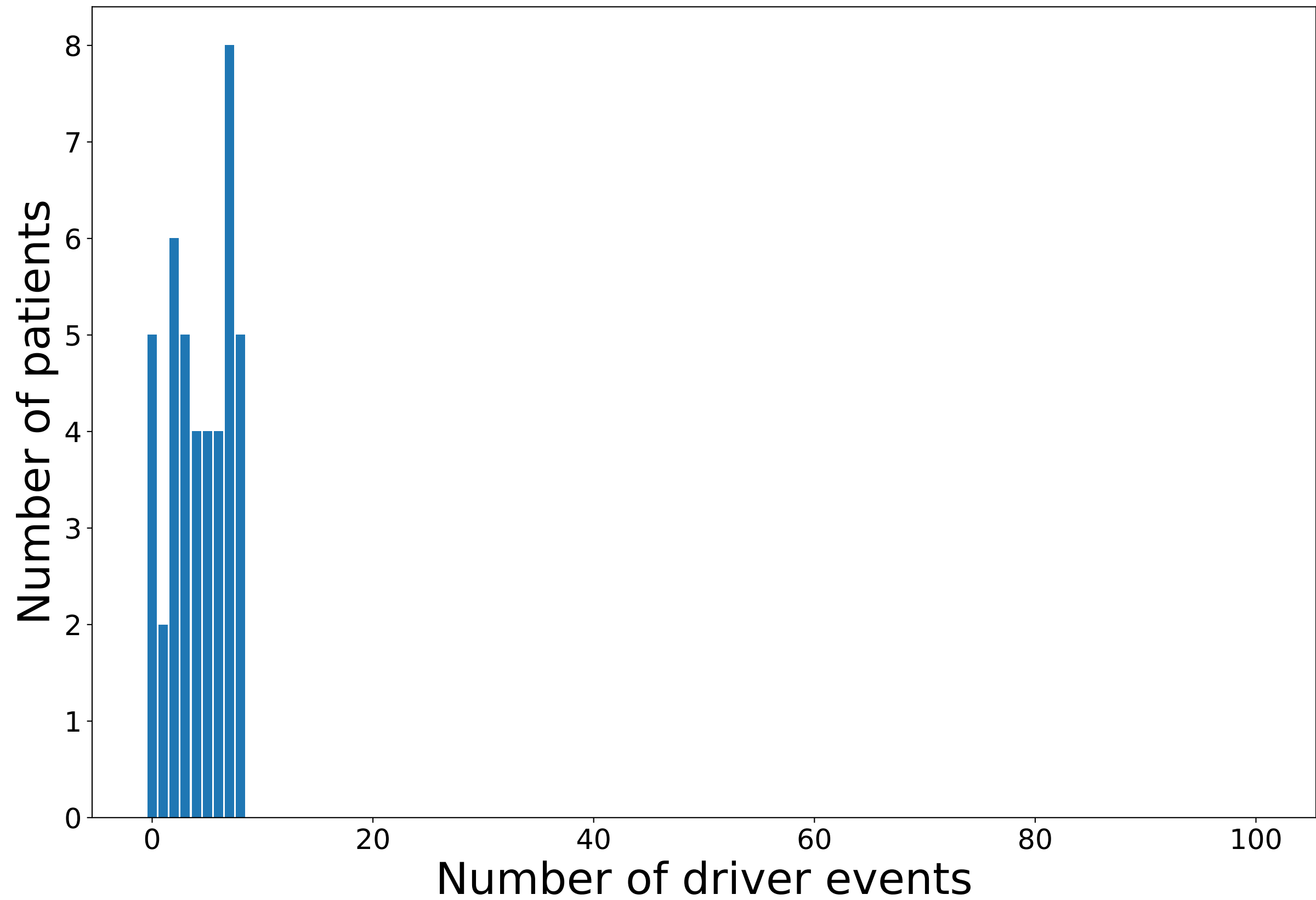

Supplement: S4 Files — (ZIP) [file pgen.1009996.s004.zip › Aneuploidy/COHORTS GISTIC2/patient distributions/2021_11_23_15_0_PCPG.pdf]

# KICH\_MALE

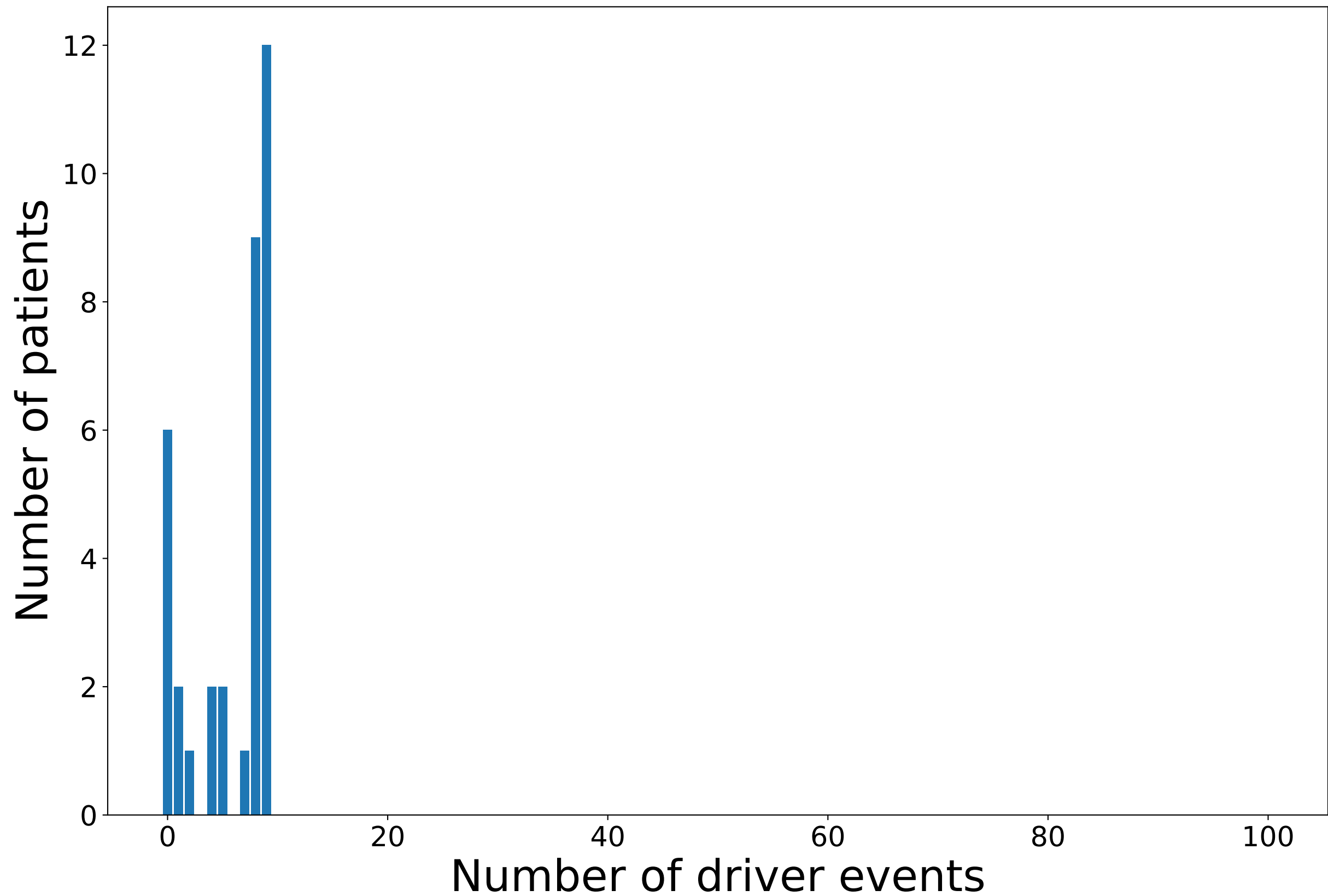

Supplement: S4 Files — (ZIP) [file pgen.1009996.s004.zip › Aneuploidy/COHORTS GISTIC2/patient distributions/2021_11_23_15_0_KICH_MALE.pdf]

# THYM\_FEMALE

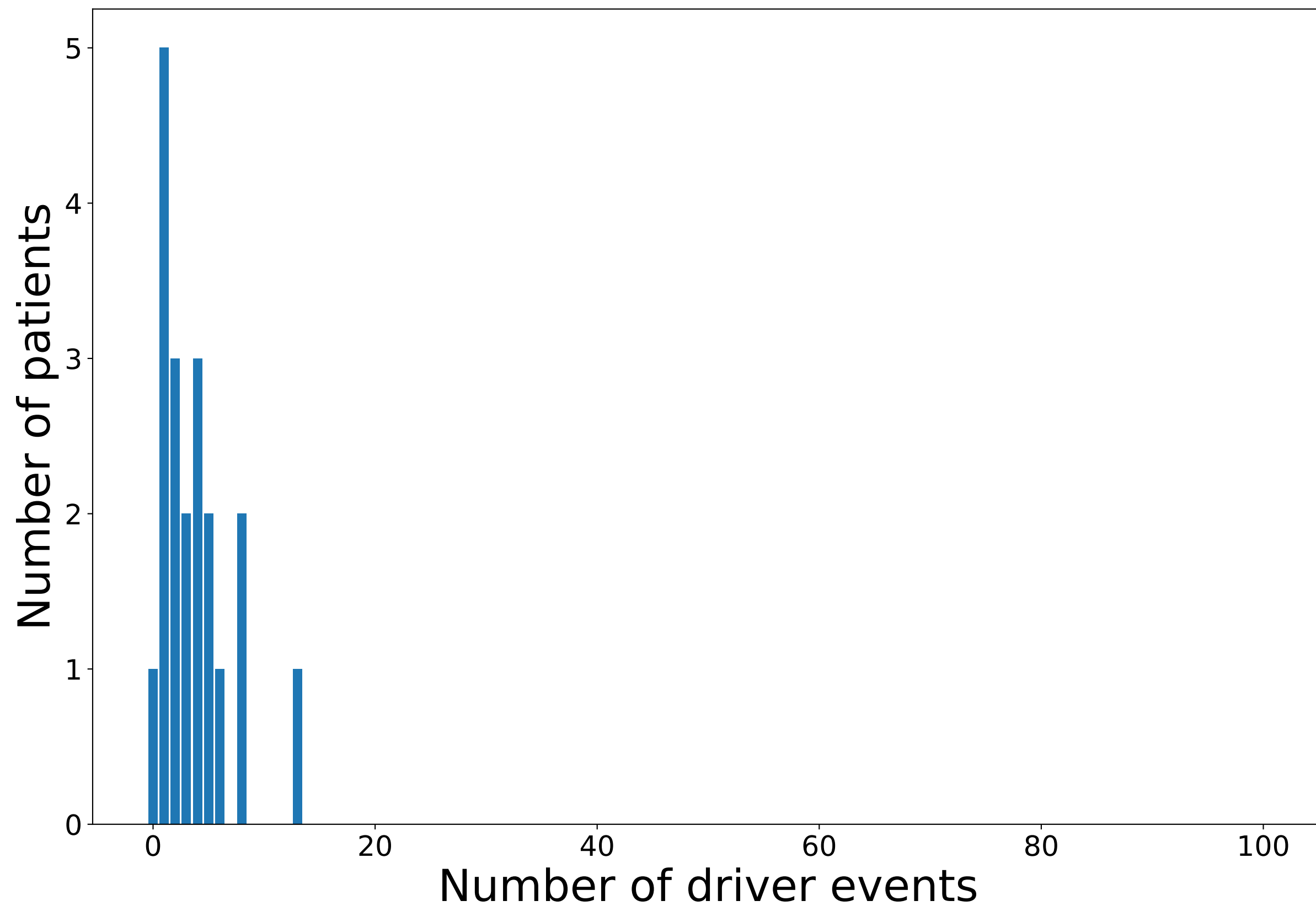

Supplement: S4 Files — (ZIP) [file pgen.1009996.s004.zip › Aneuploidy/COHORTS GISTIC2/patient distributions/2021_11_23_15_0_THYM_FEMALE.pdf]

# LUSC

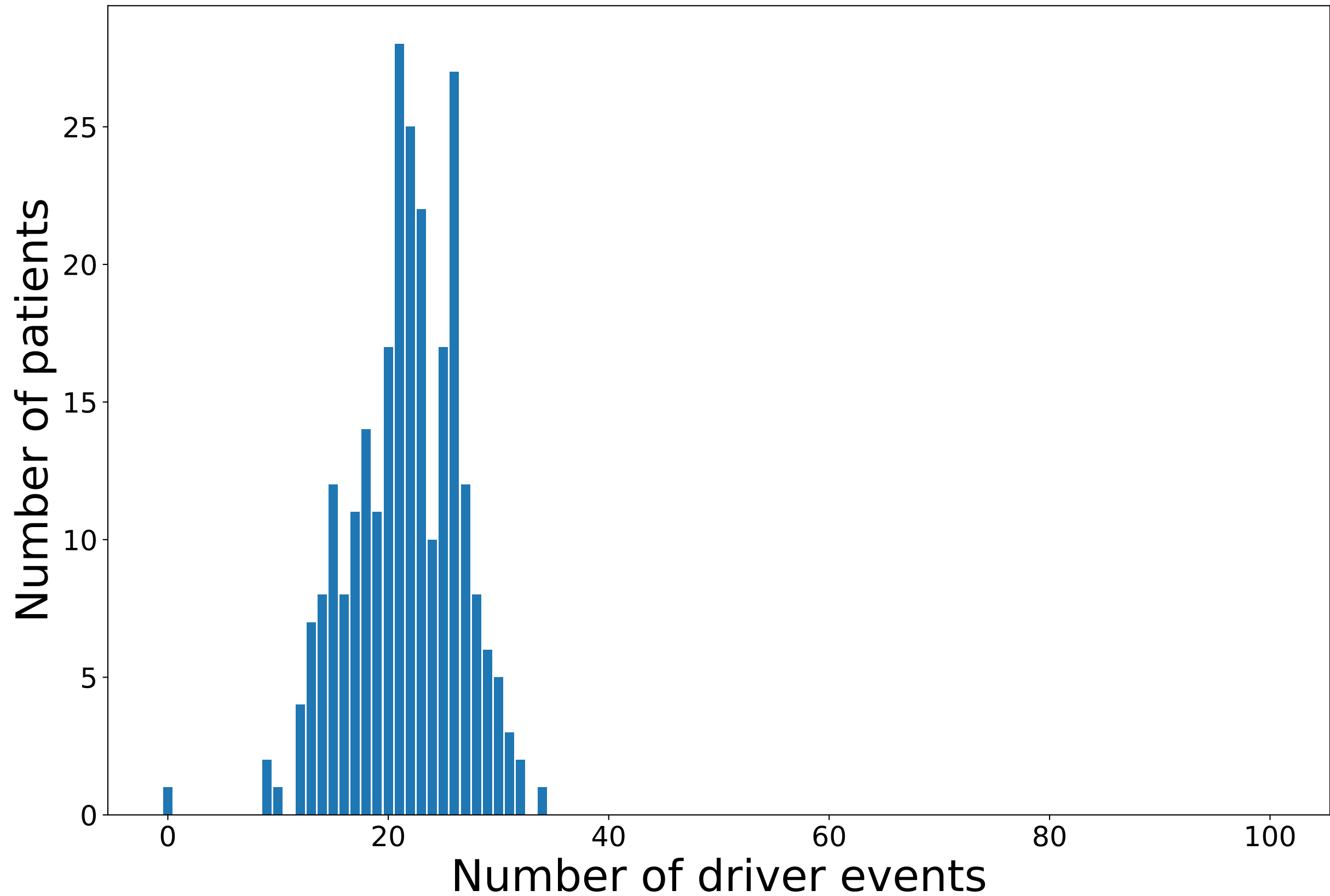

Supplement: S4 Files — (ZIP) [file pgen.1009996.s004.zip › Aneuploidy/COHORTS GISTIC2/patient distributions/2021_11_23_15_0_LUSC.pdf]

# THCA\_MALE

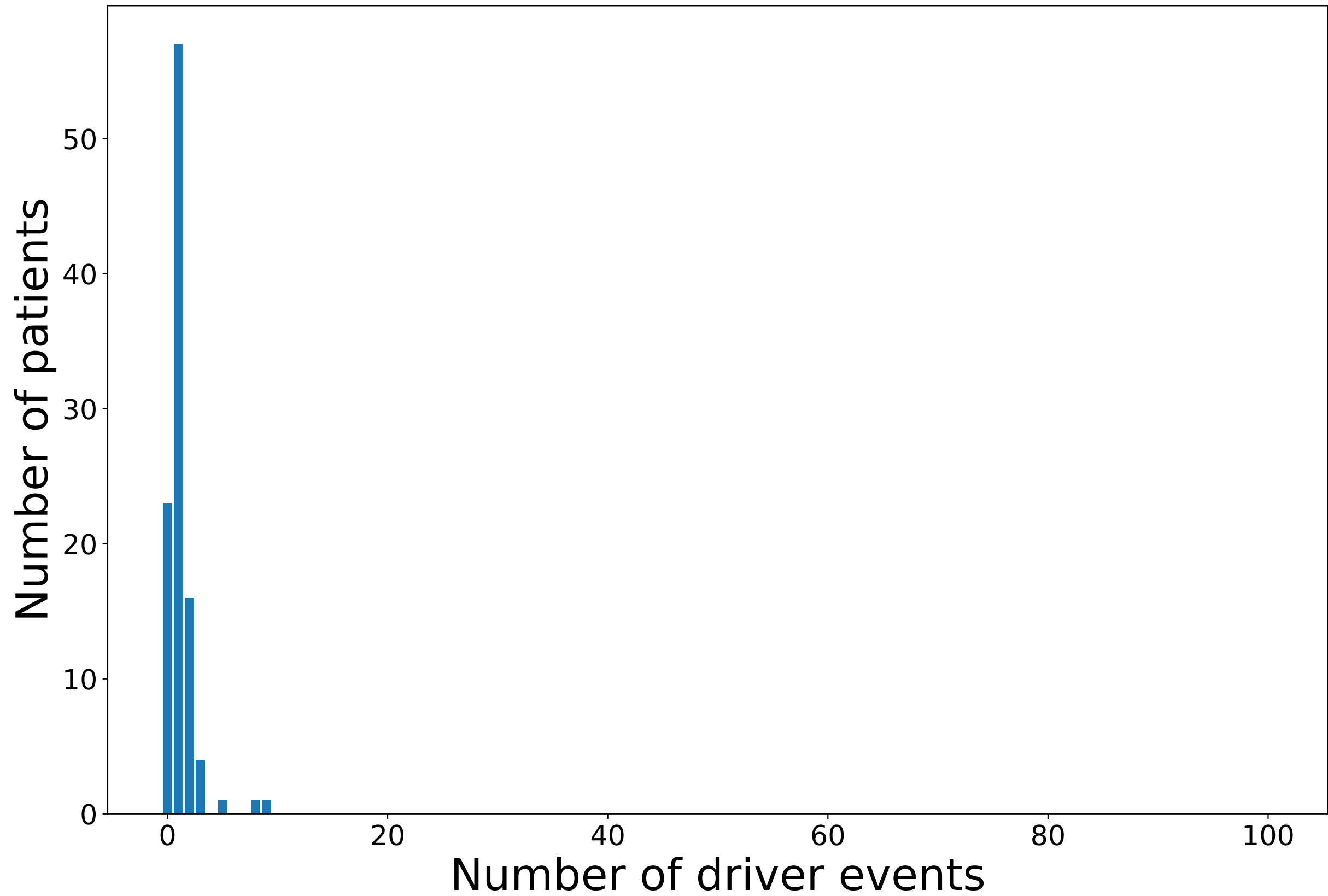

Supplement: S4 Files — (ZIP) [file pgen.1009996.s004.zip › Aneuploidy/COHORTS GISTIC2/patient distributions/2021_11_23_15_0_THCA_MALE.pdf]

# PCPG\_MALE

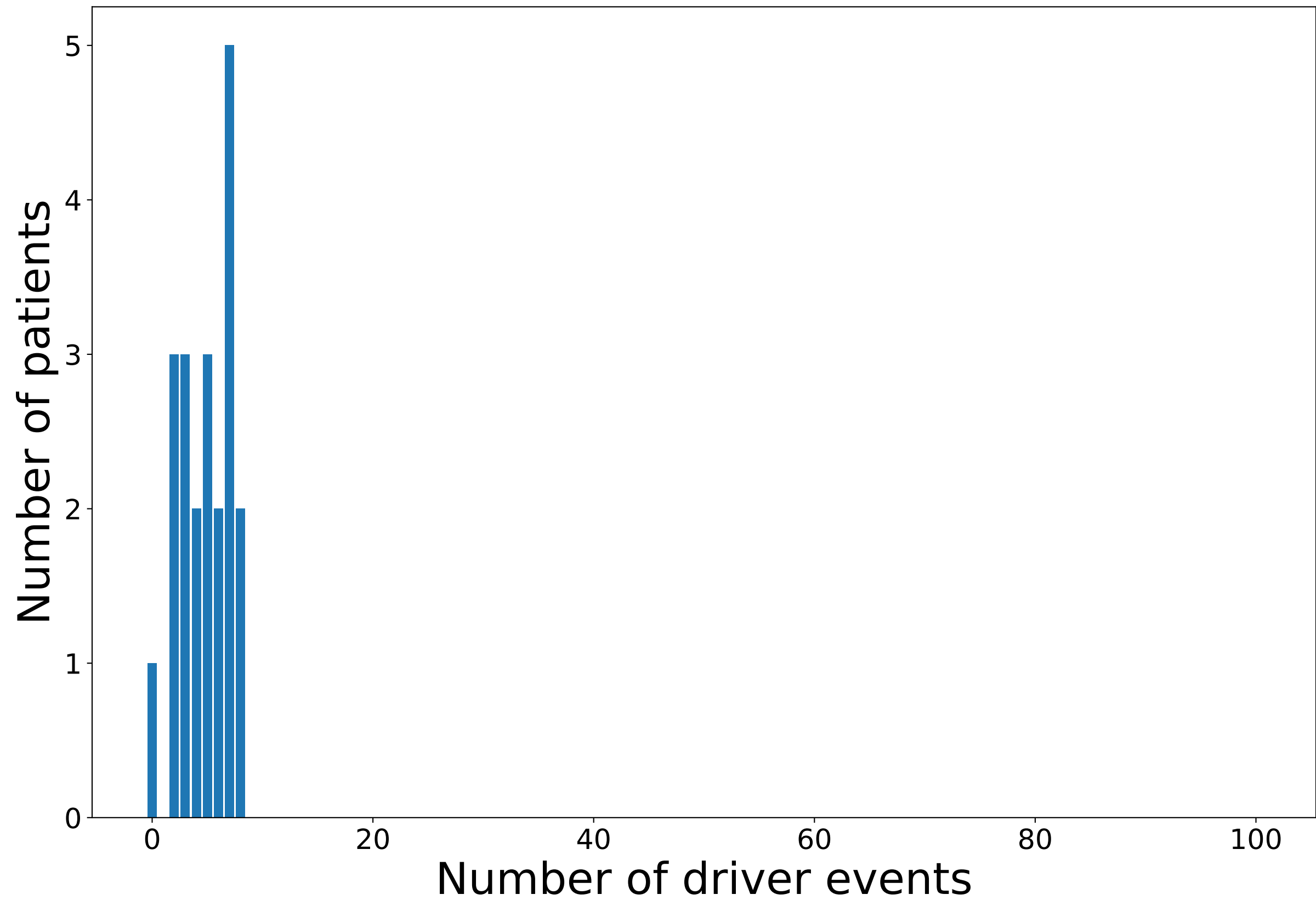

Supplement: S4 Files — (ZIP) [file pgen.1009996.s004.zip › Aneuploidy/COHORTS GISTIC2/patient distributions/2021_11_23_15_0_PCPG_MALE.pdf]

# READ\_MALE

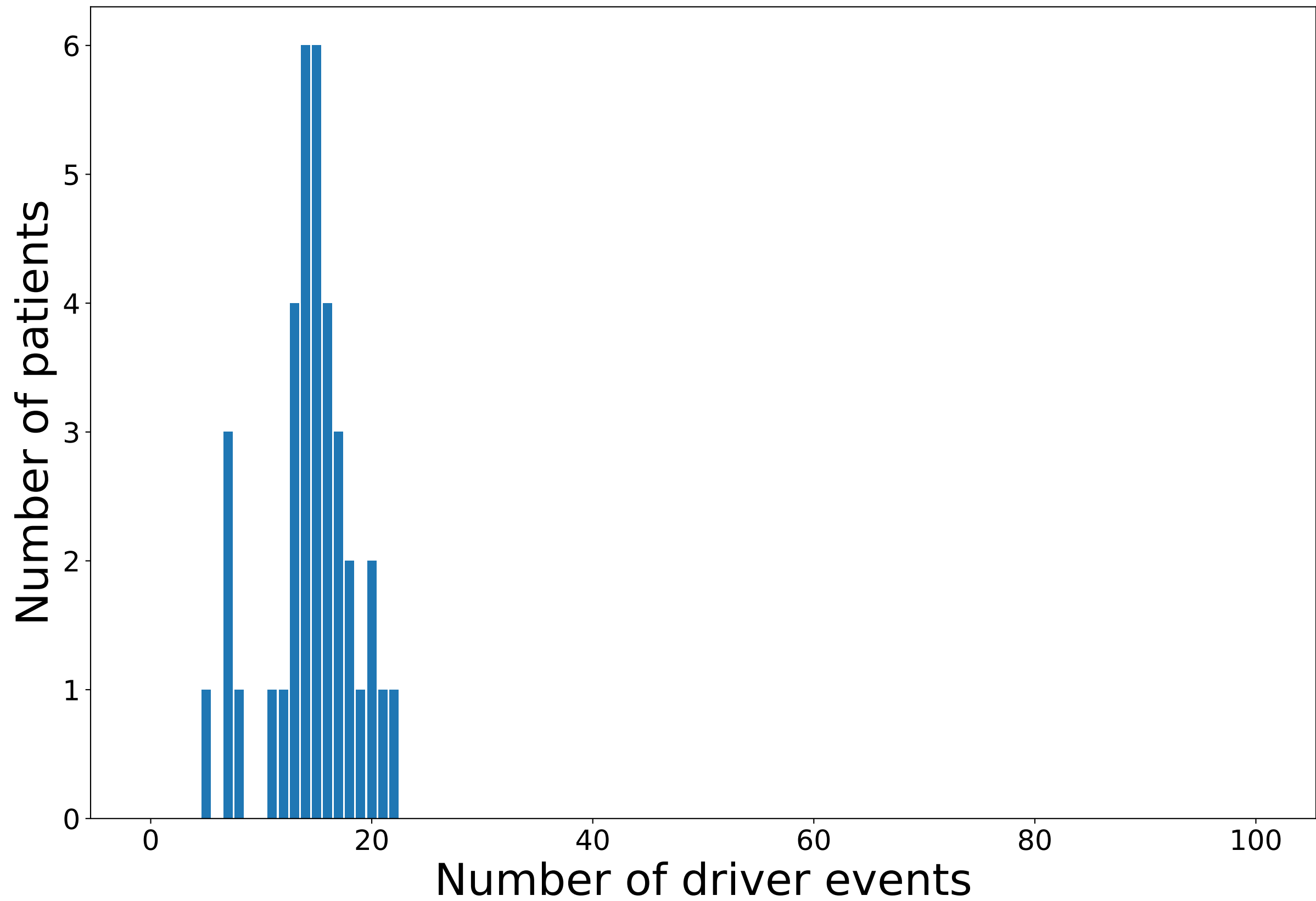

Supplement: S4 Files — (ZIP) [file pgen.1009996.s004.zip › Aneuploidy/COHORTS GISTIC2/patient distributions/2021_11_23_15_0_READ_MALE.pdf]
