# Supplementary figures and images for: Comprehensive patient-level classification and quantification of driver events in TCGA PanCanAtlas cohorts (part 5 of 6)
Source: PLoS Genet. 2022 Jan 14;18(1):e1009996. doi: 10.1371/journal.pgen.1009996 (PMC8759692; doi:10.1371/journal.pgen.1009996)

# COAD

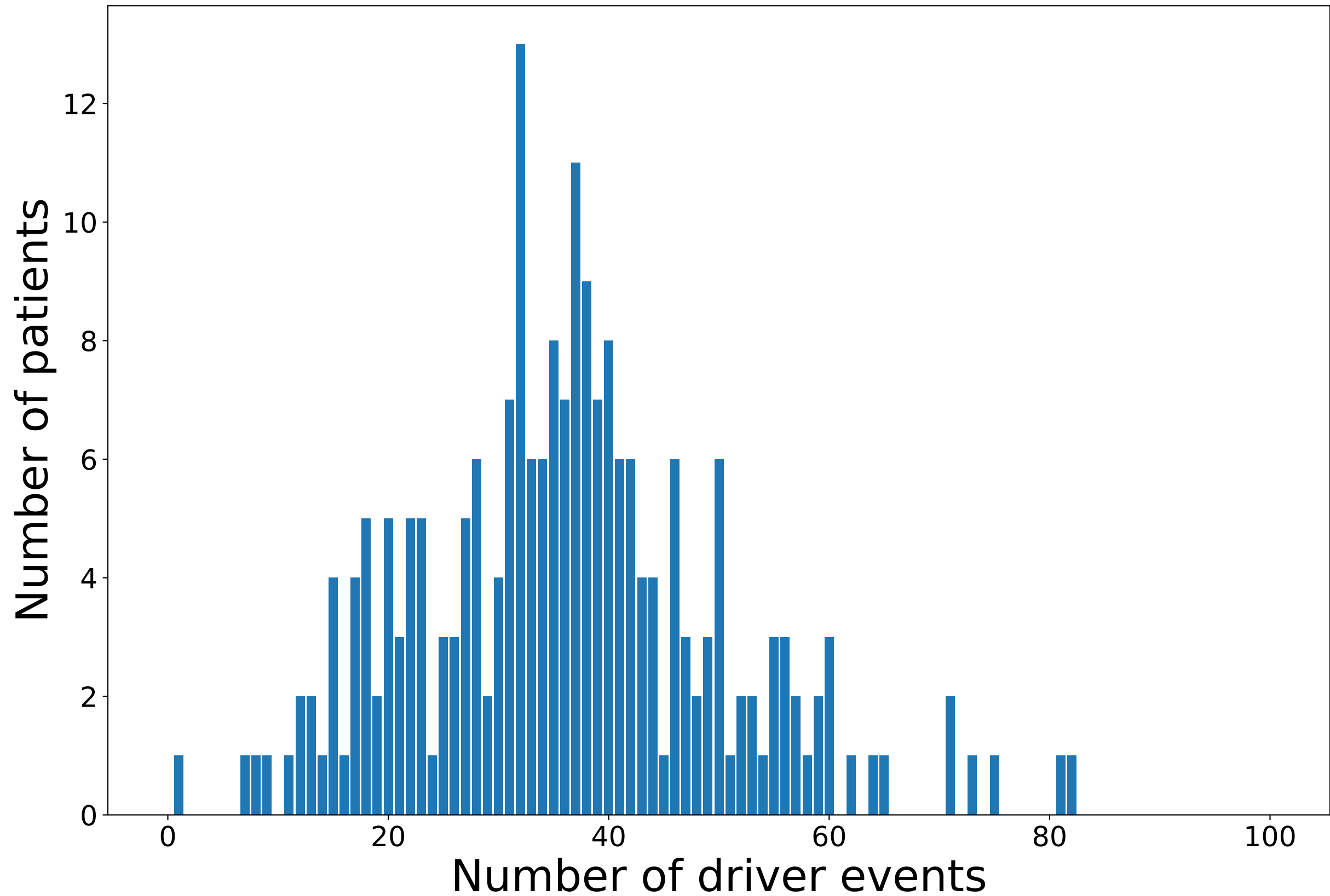

Supplement: S4 Files — (ZIP) [file pgen.1009996.s004.zip › Aneuploidy/PANCAN GISTIC2/patient distributions/2021_11_23_15_3_COAD.pdf]

# KIRP

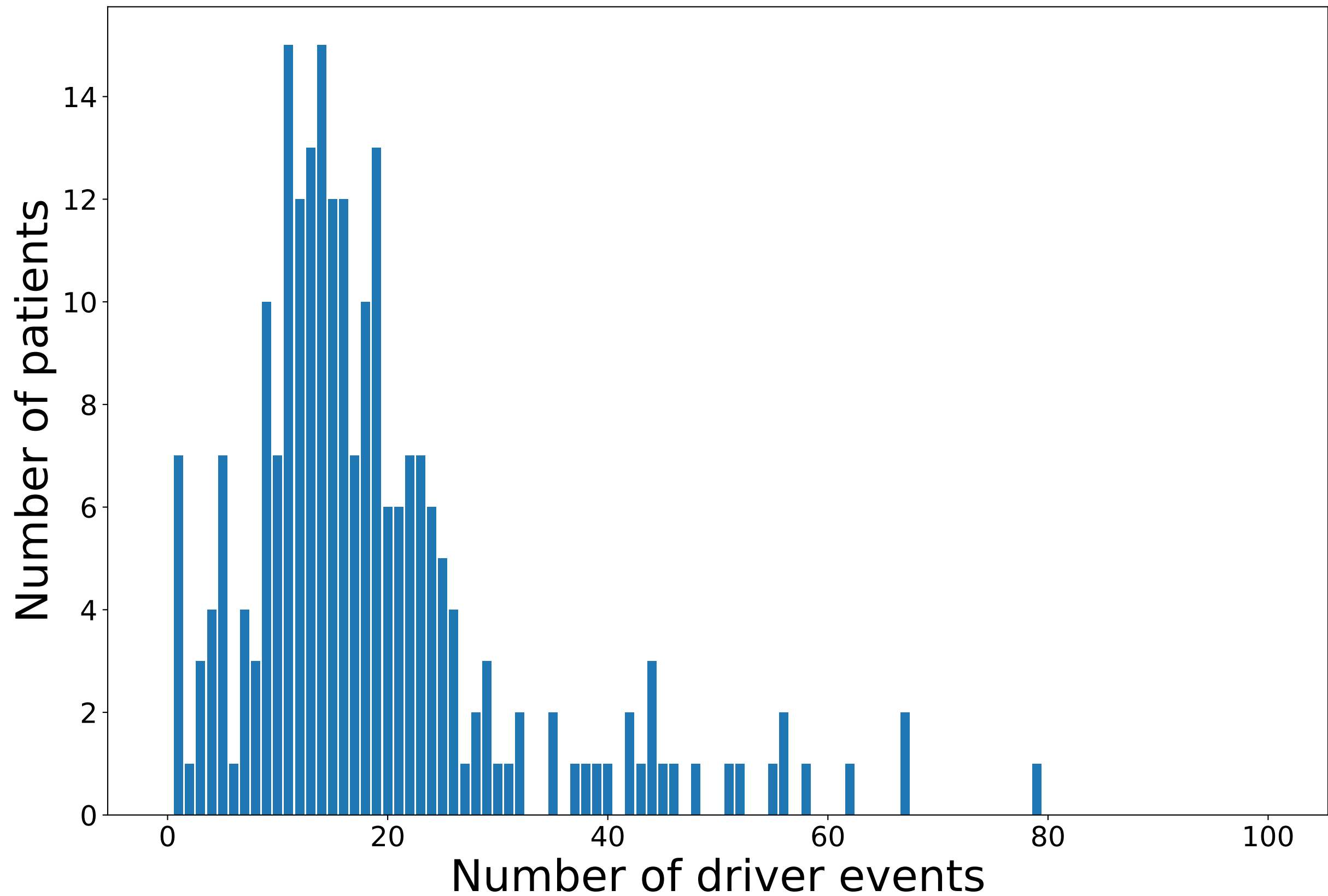

Supplement: S4 Files — (ZIP) [file pgen.1009996.s004.zip › Aneuploidy/PANCAN GISTIC2/patient distributions/2021_11_23_15_3_KIRP.pdf]

# SARC\_FEMALE

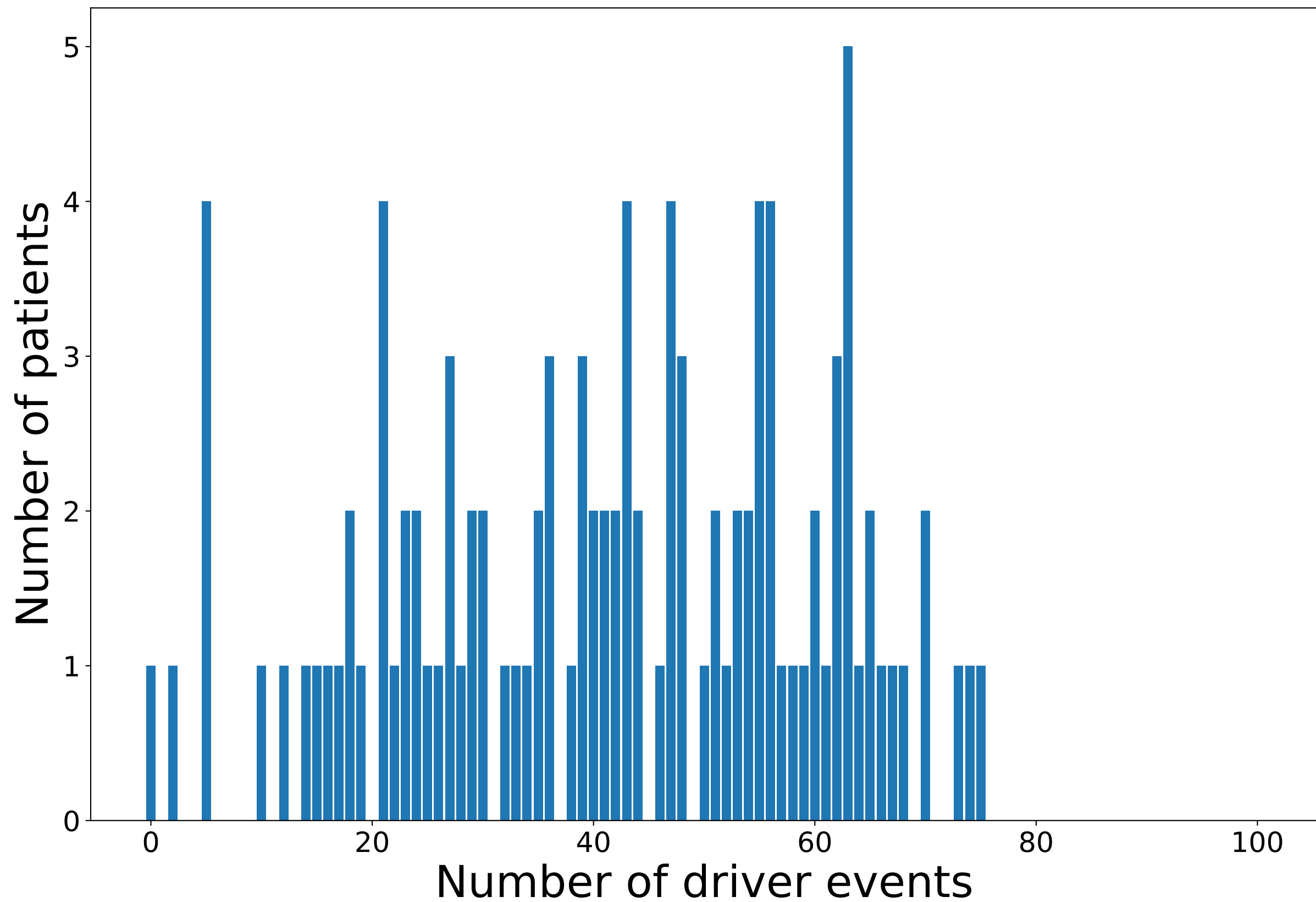

Supplement: S4 Files — (ZIP) [file pgen.1009996.s004.zip › Aneuploidy/PANCAN GISTIC2/patient distributions/2021_11_23_15_3_SARC_FEMALE.pdf]

# DLBC\_MALE

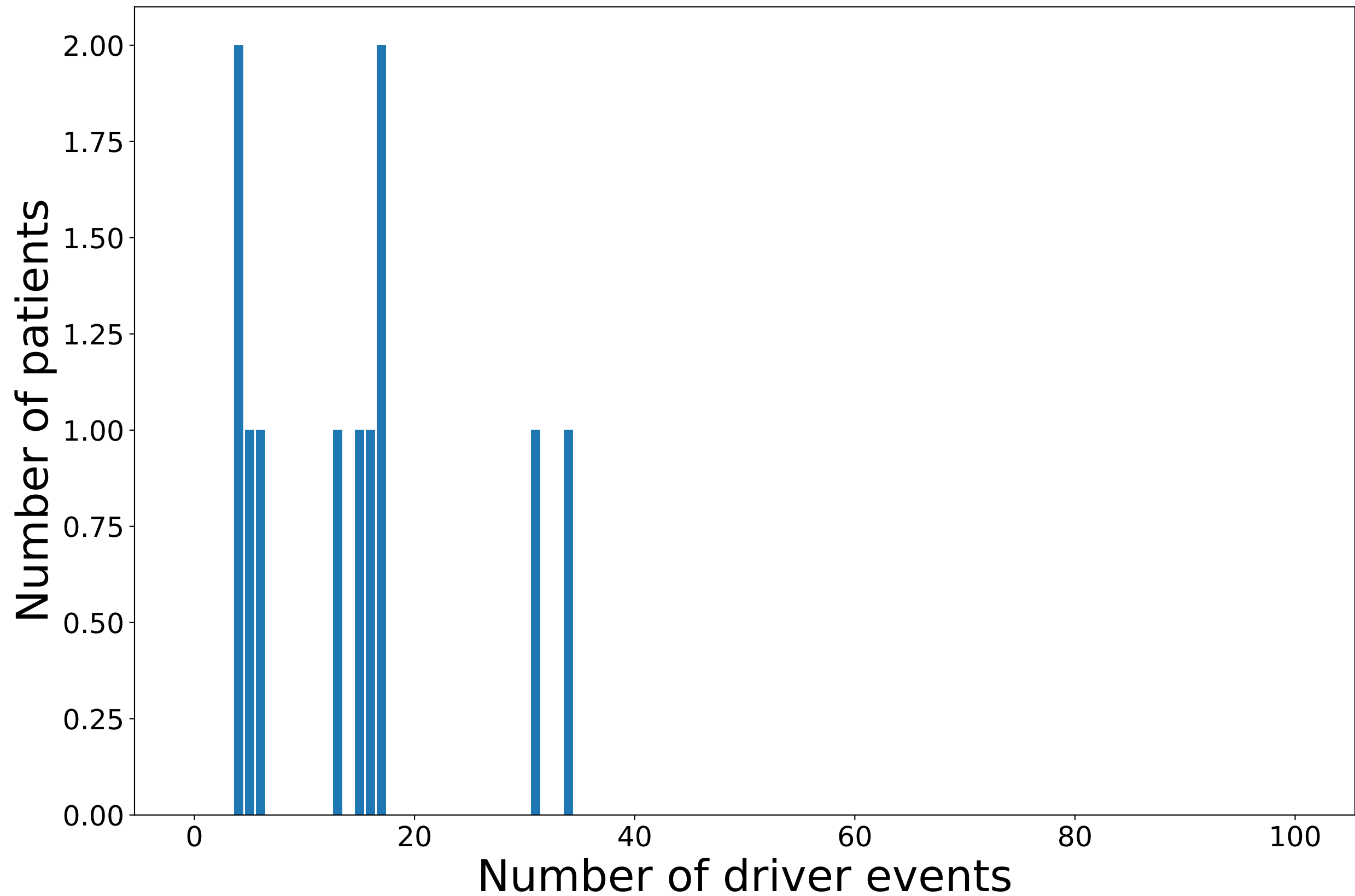

Supplement: S4 Files — (ZIP) [file pgen.1009996.s004.zip › Aneuploidy/PANCAN GISTIC2/patient distributions/2021_11_23_15_3_DLBC_MALE.pdf]

# THYM

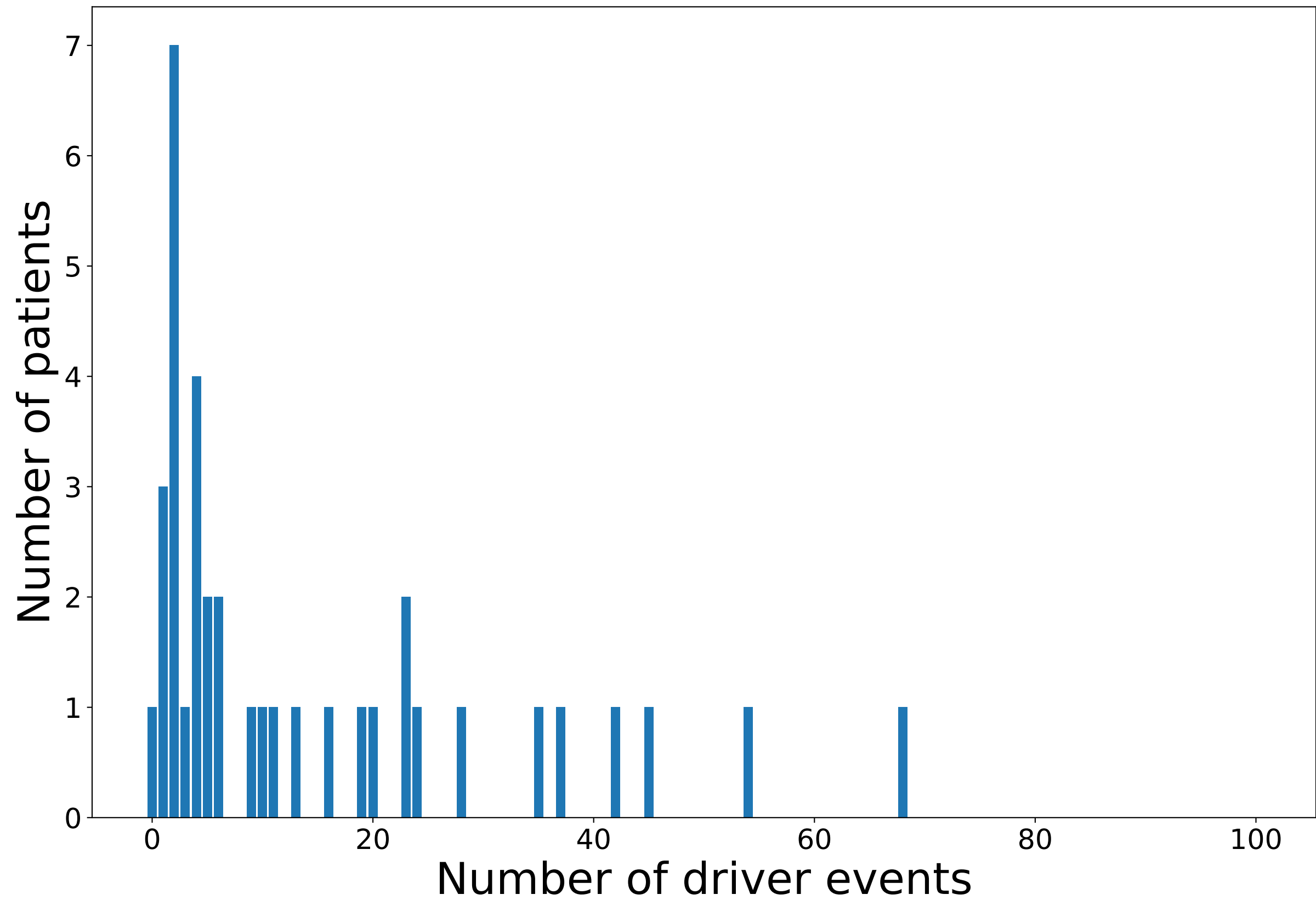

Supplement: S4 Files — (ZIP) [file pgen.1009996.s004.zip › Aneuploidy/PANCAN GISTIC2/patient distributions/2021_11_23_15_3_THYM.pdf]

# KICH\_MALE

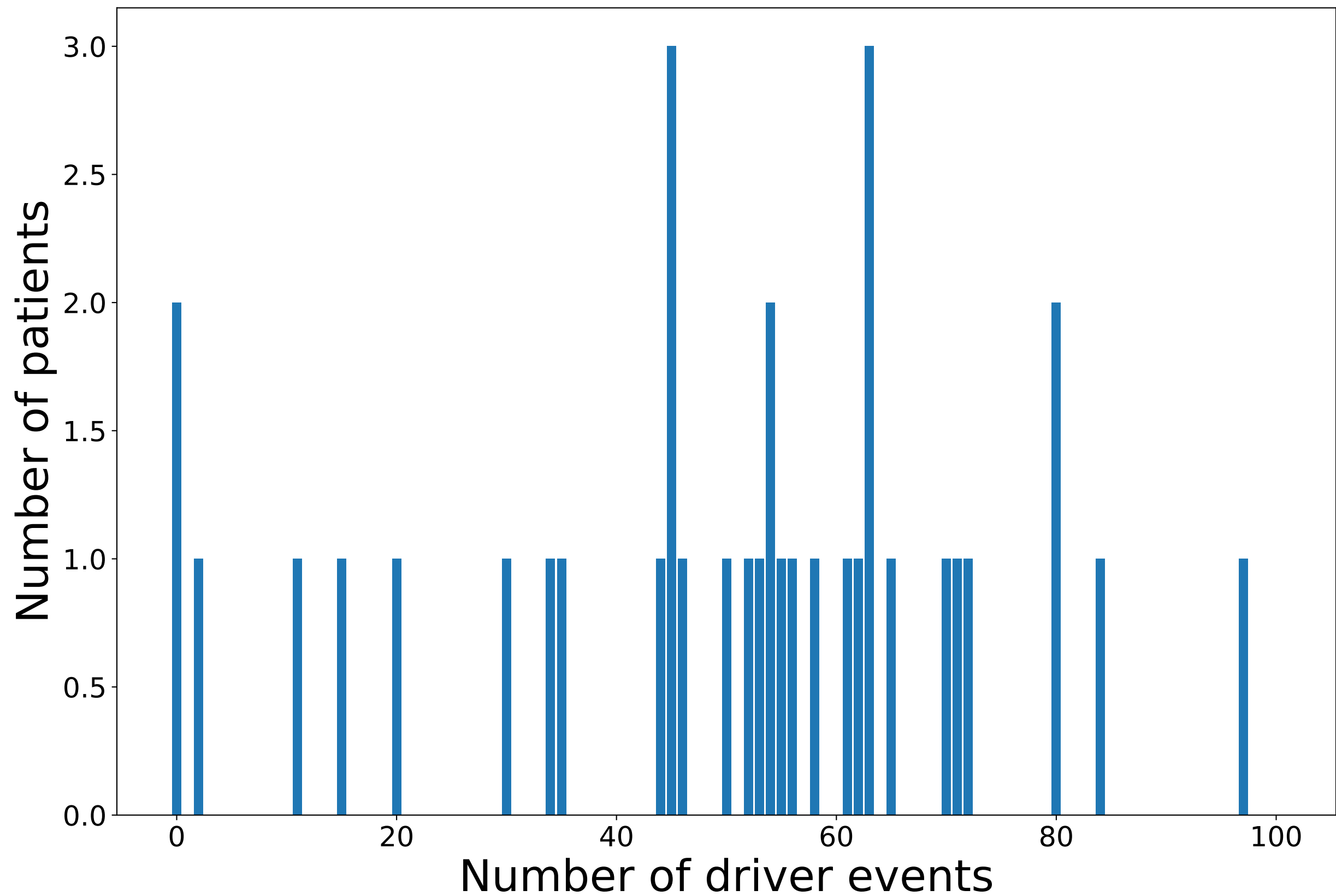

Supplement: S4 Files — (ZIP) [file pgen.1009996.s004.zip › Aneuploidy/PANCAN GISTIC2/patient distributions/2021_11_23_15_3_KICH_MALE.pdf]

# STAD\_FEMALE

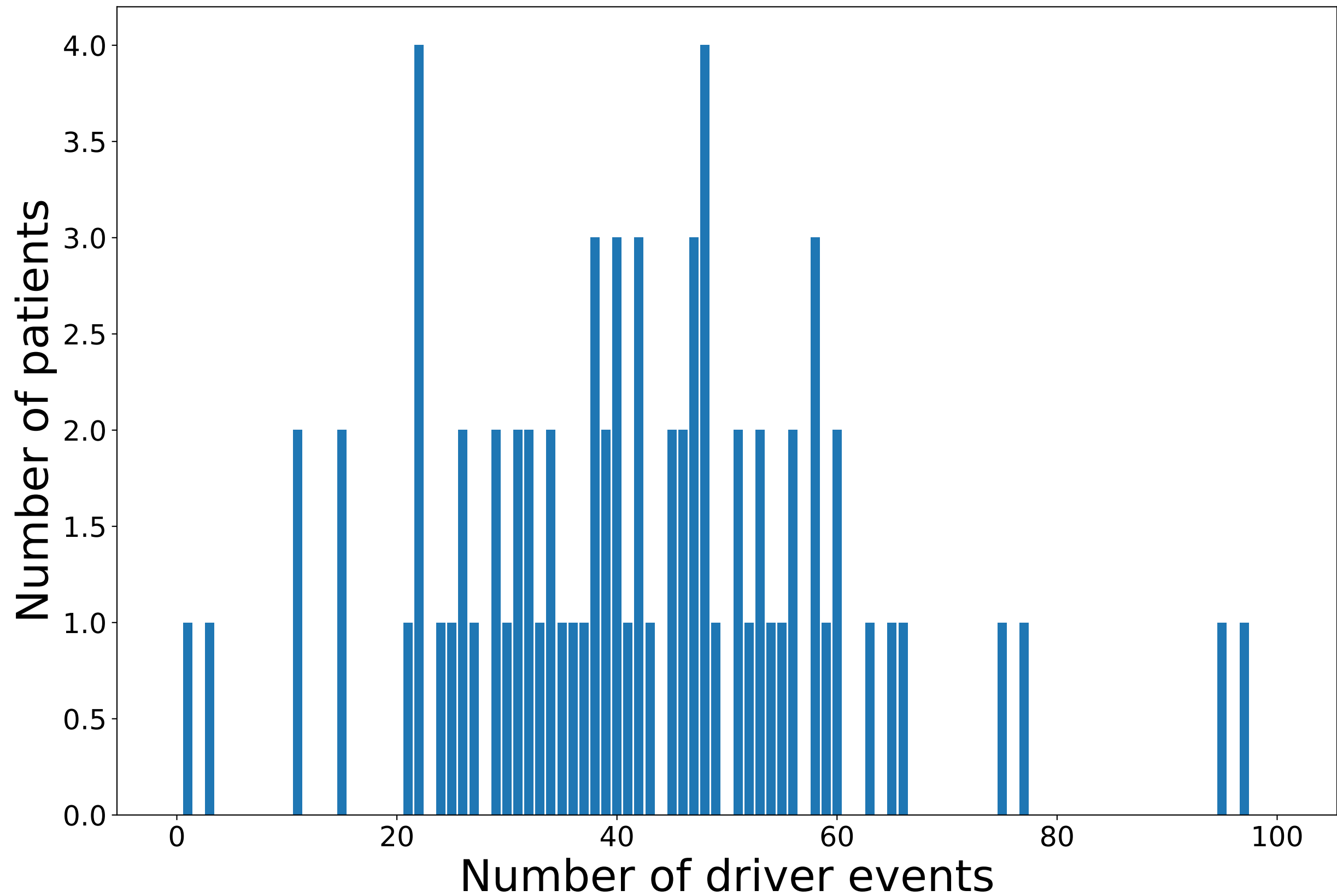

Supplement: S4 Files — (ZIP) [file pgen.1009996.s004.zip › Aneuploidy/PANCAN GISTIC2/patient distributions/2021_11_23_15_3_STAD_FEMALE.pdf]

# PANCAN\_FEMALE

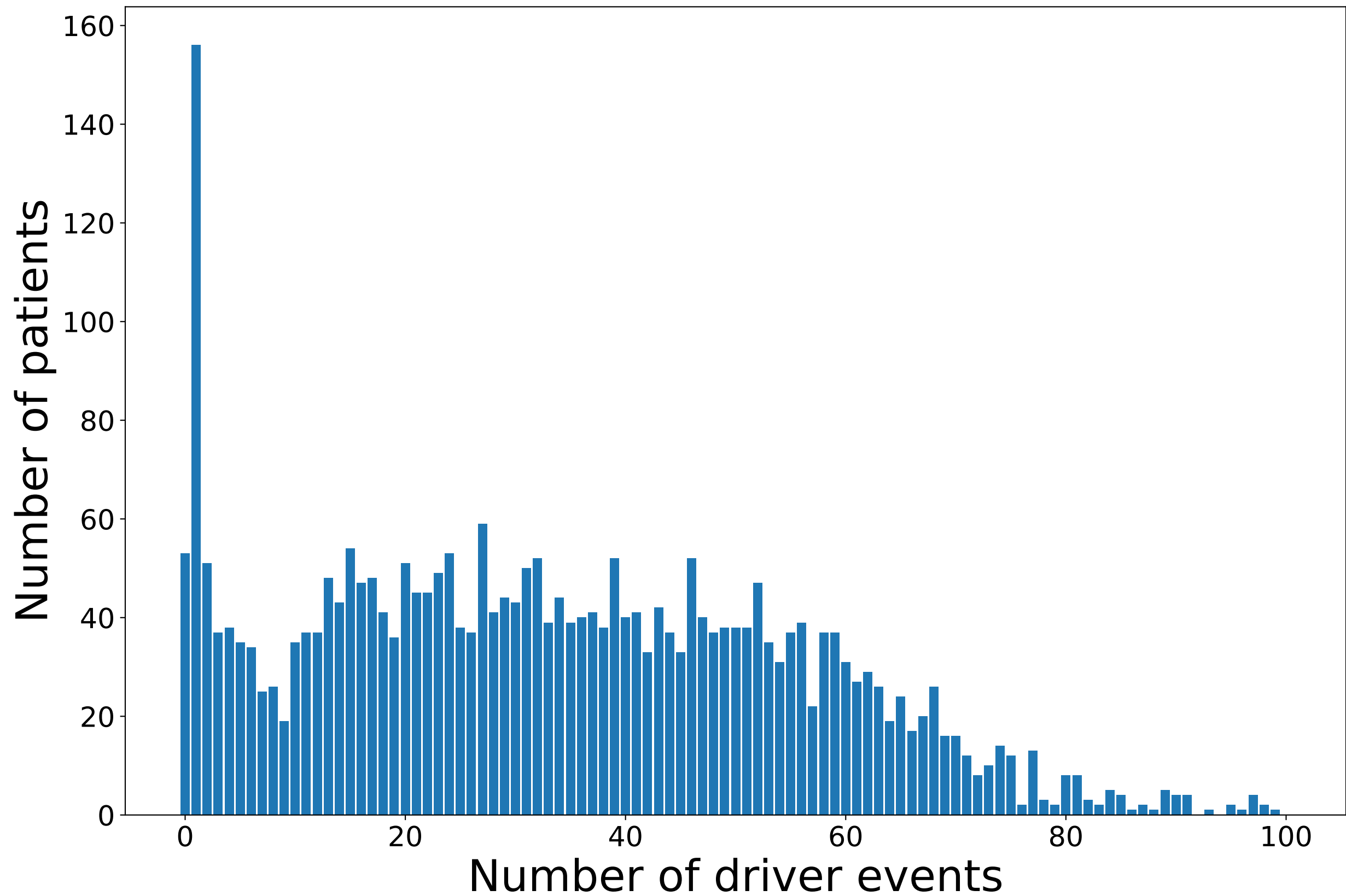

Supplement: S4 Files — (ZIP) [file pgen.1009996.s004.zip › Aneuploidy/PANCAN GISTIC2/patient distributions/2021_11_23_15_3_PANCAN_FEMALE.pdf]

# PCPG\_MALE

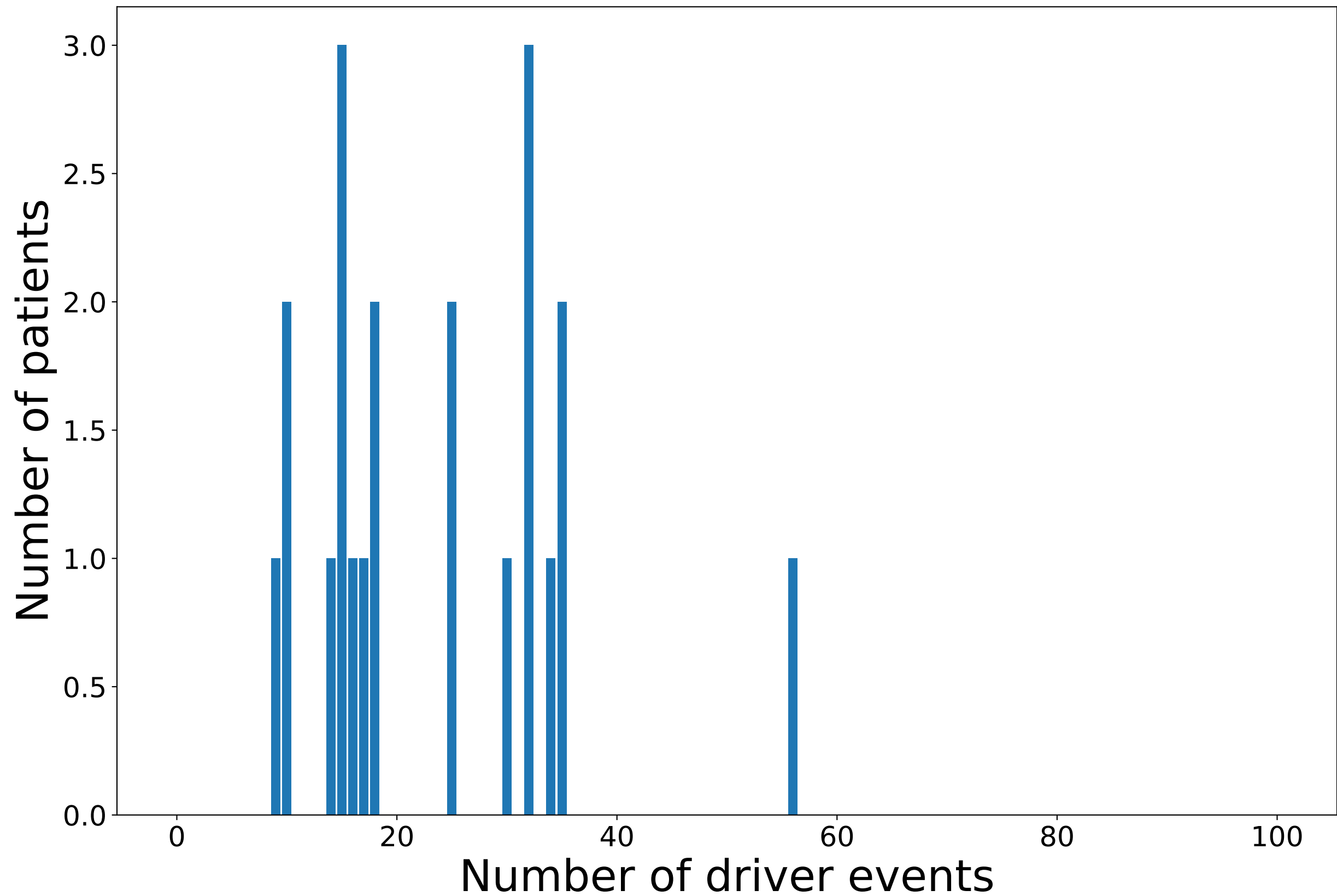

Supplement: S4 Files — (ZIP) [file pgen.1009996.s004.zip › Aneuploidy/PANCAN GISTIC2/patient distributions/2021_11_23_15_3_PCPG_MALE.pdf]

# KIRC

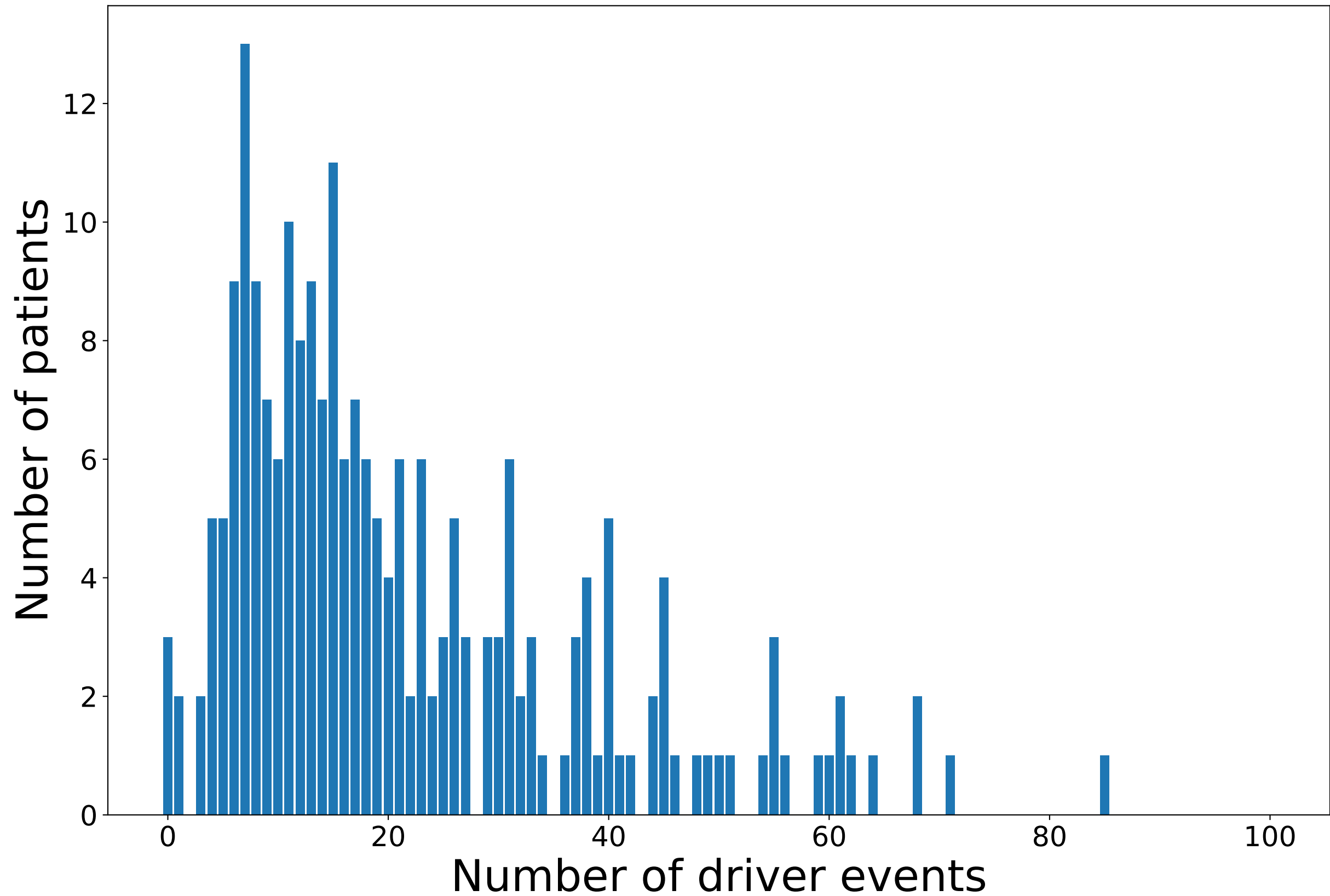

Supplement: S4 Files — (ZIP) [file pgen.1009996.s004.zip › Aneuploidy/PANCAN GISTIC2/patient distributions/2021_11_23_15_3_KIRC.pdf]

# THCA\_MALE

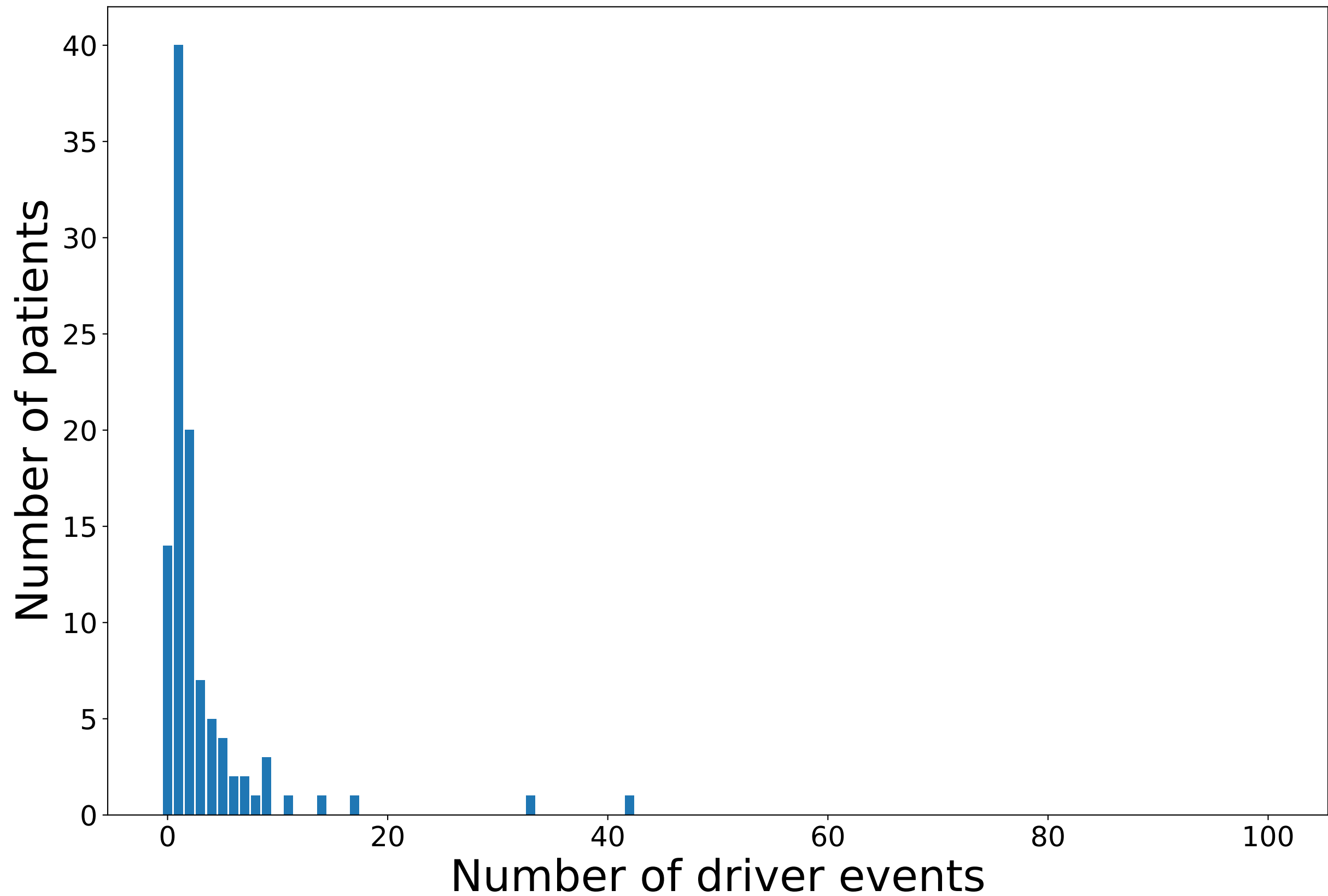

Supplement: S4 Files — (ZIP) [file pgen.1009996.s004.zip › Aneuploidy/PANCAN GISTIC2/patient distributions/2021_11_23_15_3_THCA_MALE.pdf]

# READ\_MALE

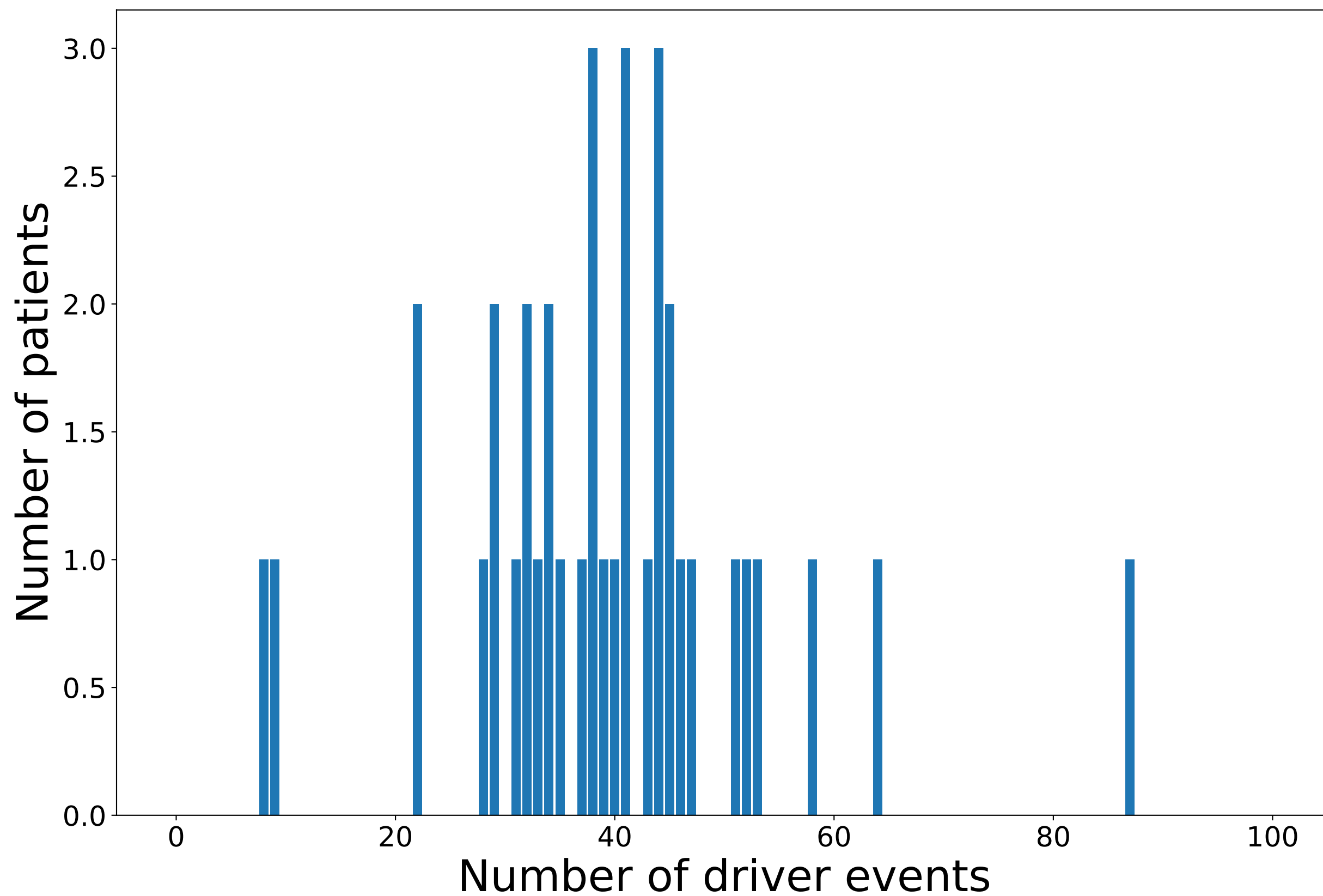

Supplement: S4 Files — (ZIP) [file pgen.1009996.s004.zip › Aneuploidy/PANCAN GISTIC2/patient distributions/2021_11_23_15_3_READ_MALE.pdf]

# UVM\_FEMALE

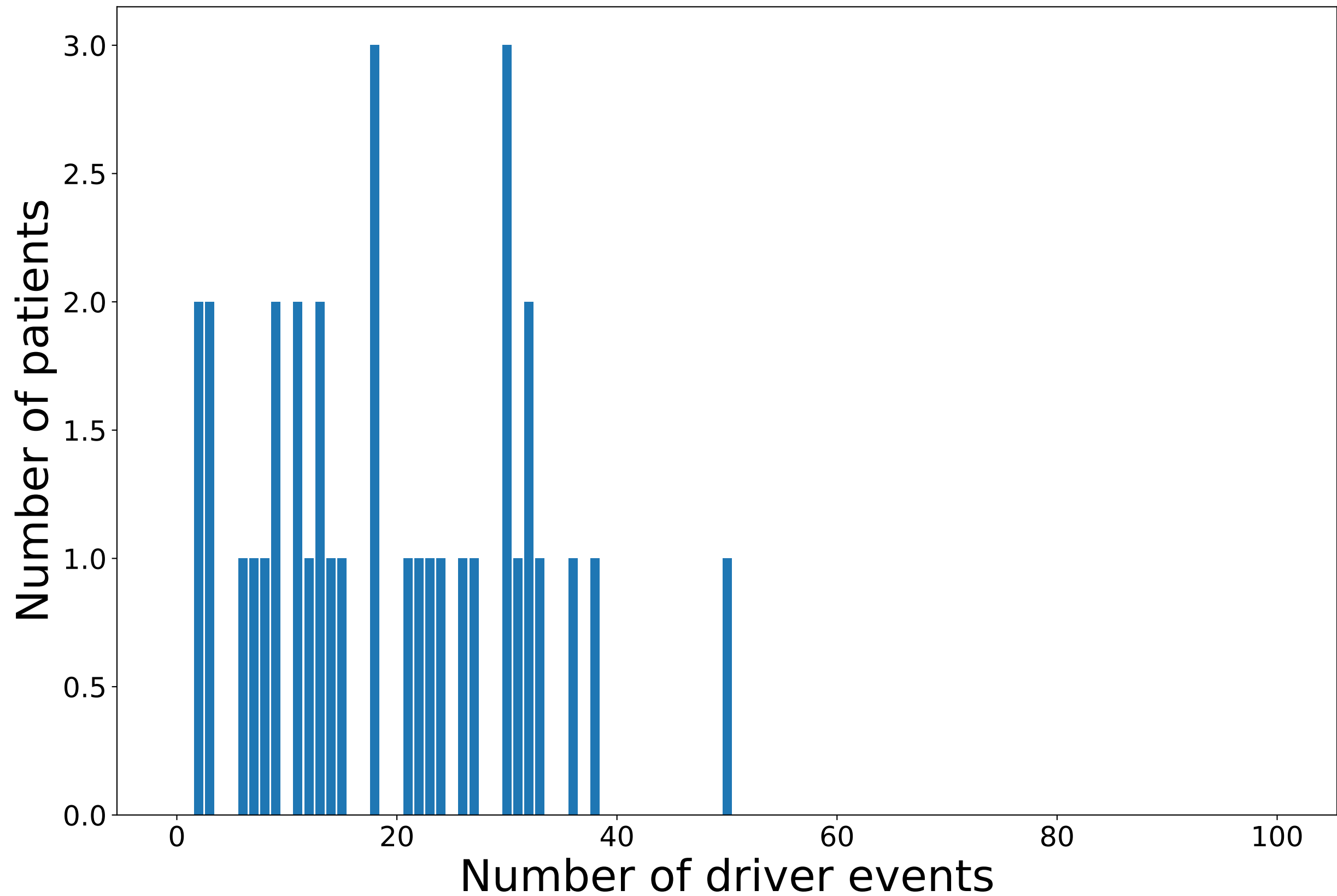

Supplement: S4 Files — (ZIP) [file pgen.1009996.s004.zip › Aneuploidy/PANCAN GISTIC2/patient distributions/2021_11_23_15_3_UVM_FEMALE.pdf]

# GBM\_MALE

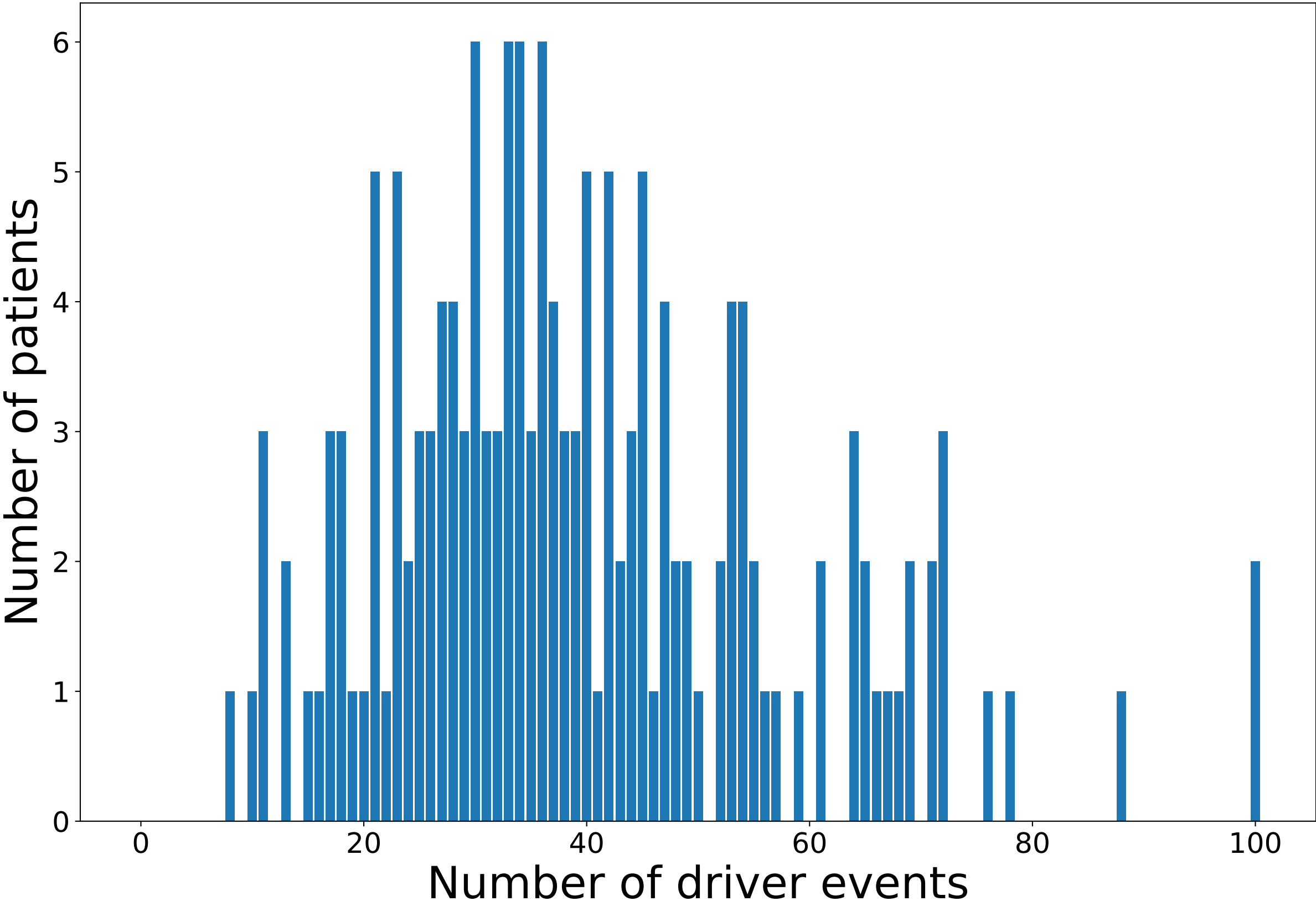

Supplement: S4 Files — (ZIP) [file pgen.1009996.s004.zip › Aneuploidy/PANCAN GISTIC2/patient distributions/2021_11_23_15_3_GBM_MALE.pdf]

# READ

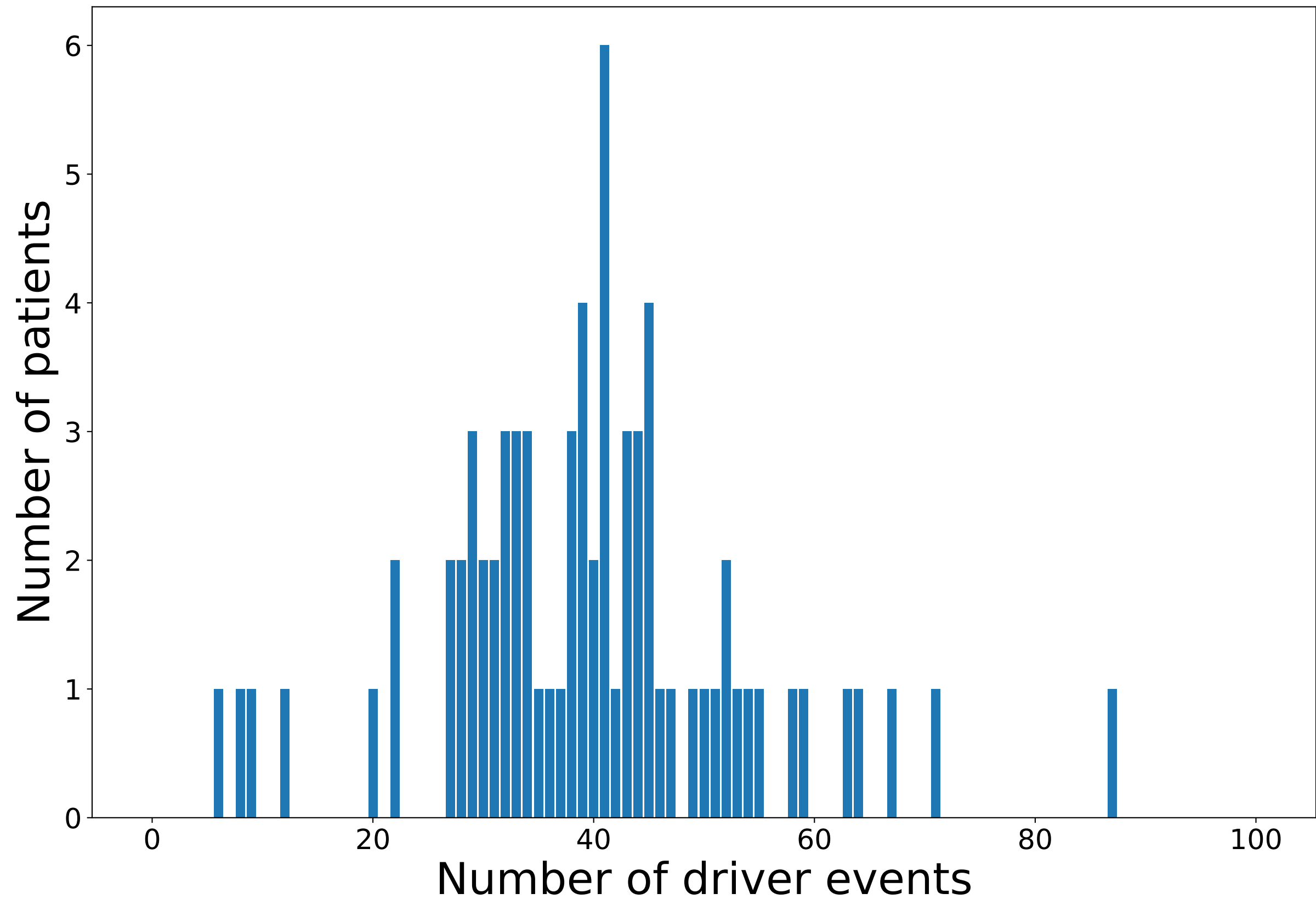

Supplement: S4 Files — (ZIP) [file pgen.1009996.s004.zip › Aneuploidy/PANCAN GISTIC2/patient distributions/2021_11_23_15_3_READ.pdf]

# THCA\_FEMALE

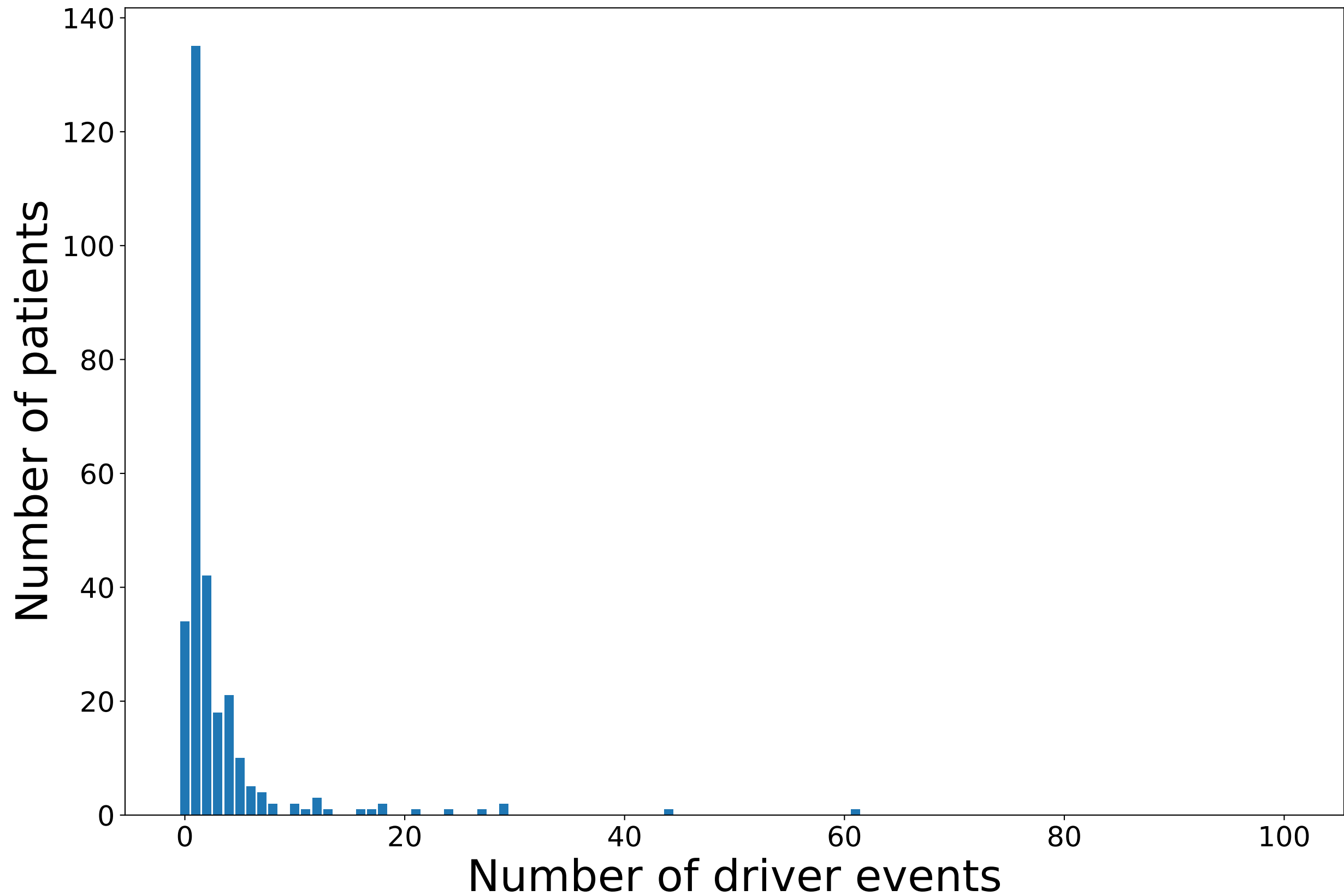

Supplement: S4 Files — (ZIP) [file pgen.1009996.s004.zip › Aneuploidy/PANCAN GISTIC2/patient distributions/2021_11_23_15_3_THCA_FEMALE.pdf]

# BLCA

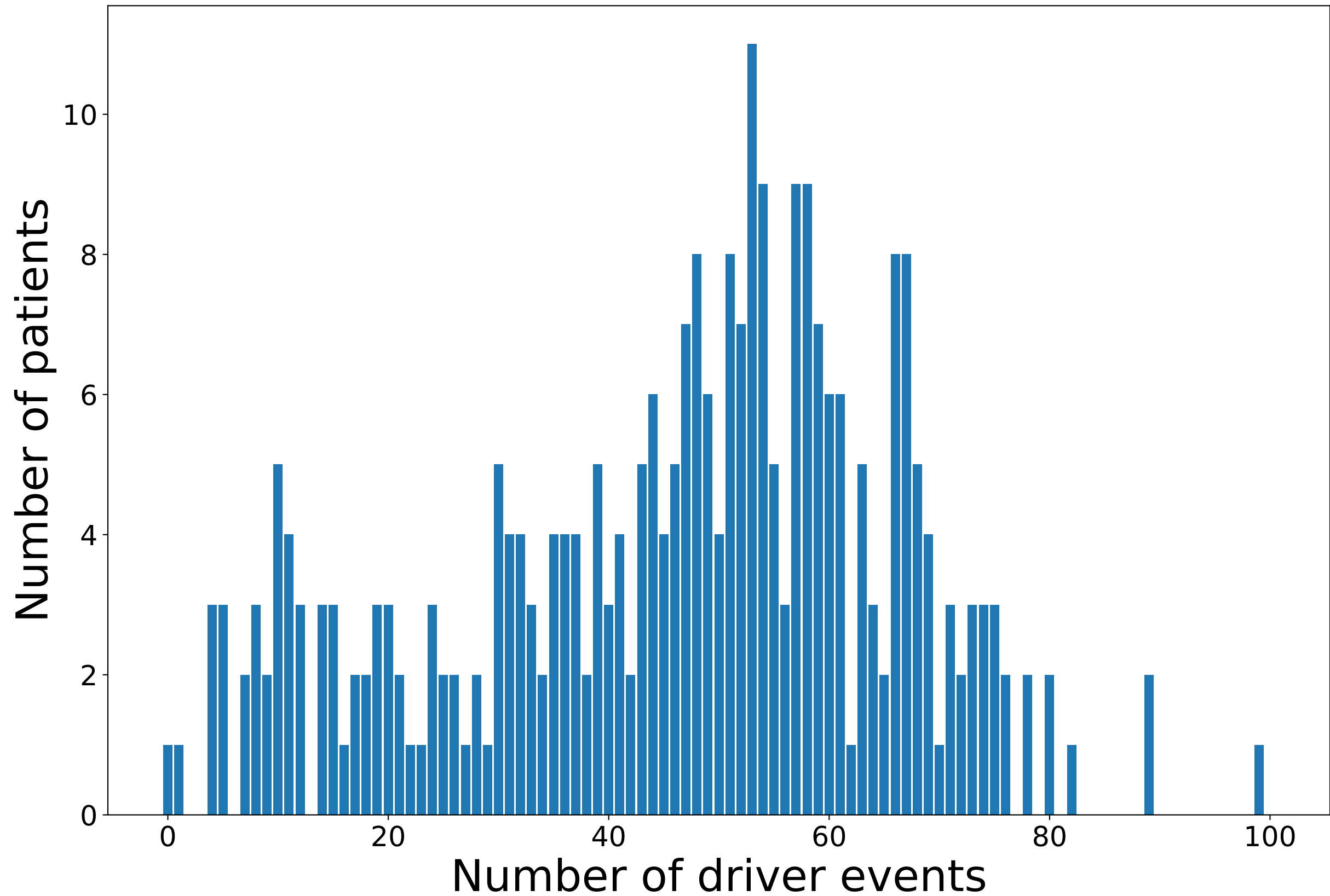

Supplement: S4 Files — (ZIP) [file pgen.1009996.s004.zip › Aneuploidy/PANCAN GISTIC2/patient distributions/2021_11_23_15_3_BLCA.pdf]

# THCA

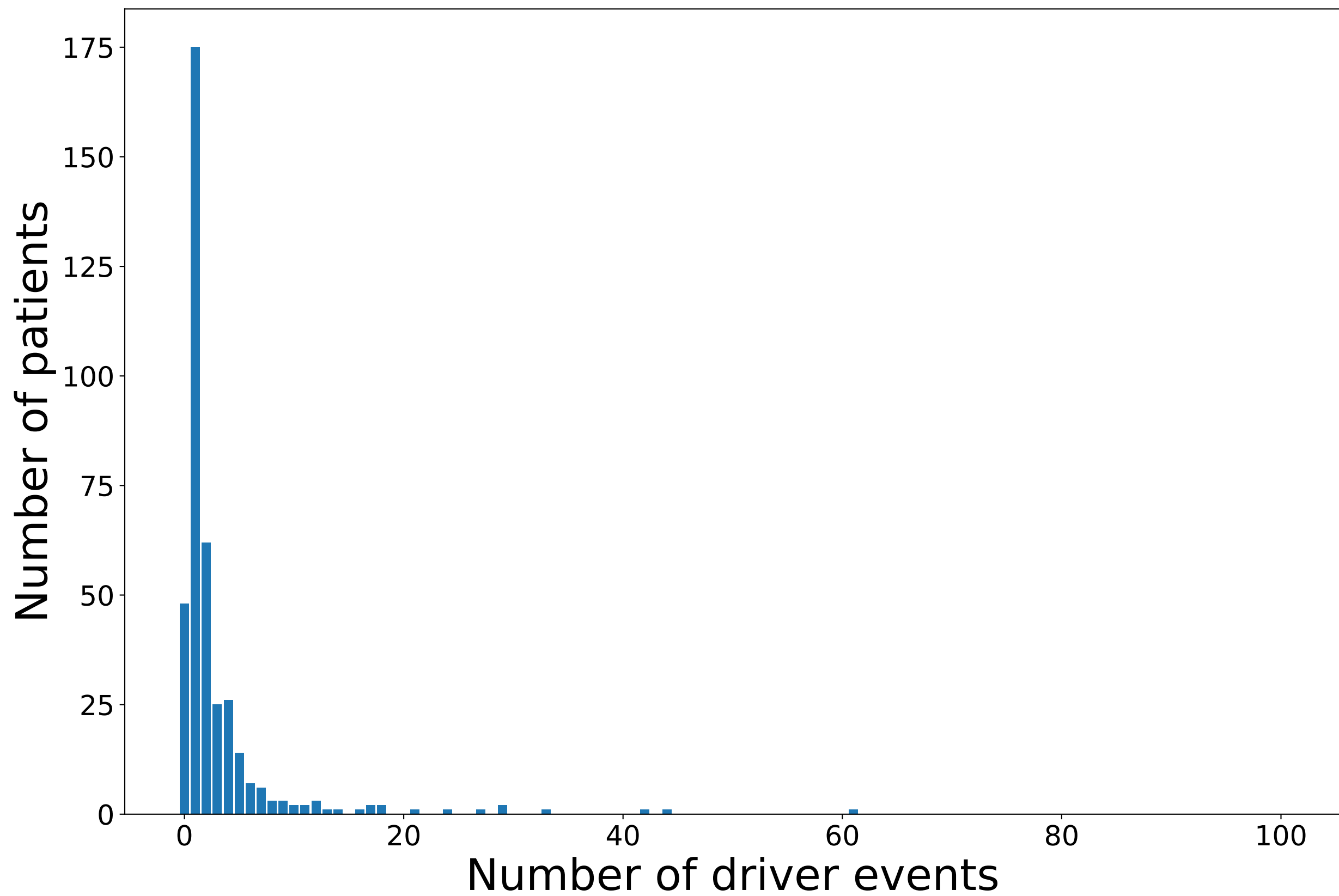

Supplement: S4 Files — (ZIP) [file pgen.1009996.s004.zip › Aneuploidy/PANCAN GISTIC2/patient distributions/2021_11_23_15_3_THCA.pdf]

# PAAD

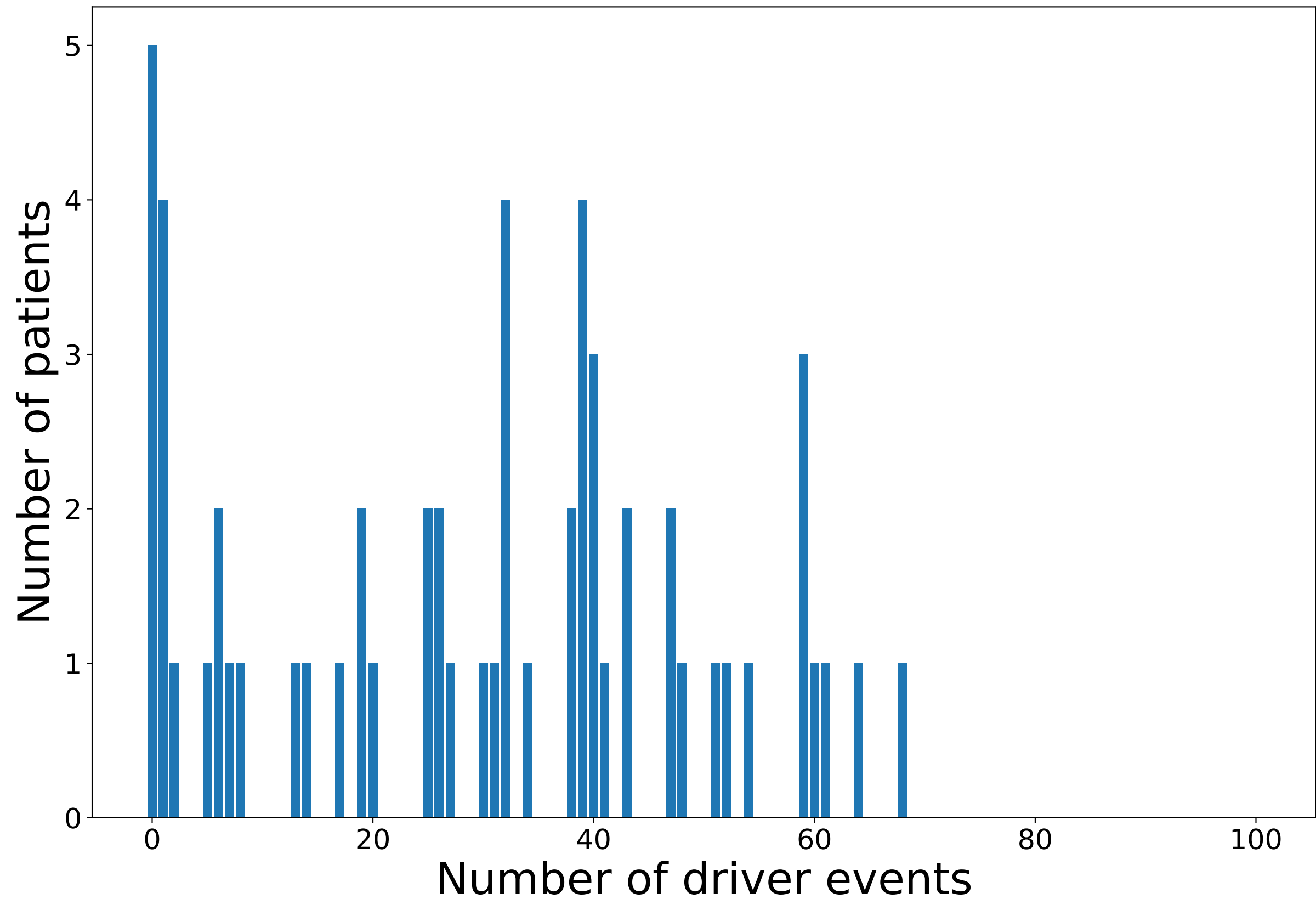

Supplement: S4 Files — (ZIP) [file pgen.1009996.s004.zip › Aneuploidy/PANCAN GISTIC2/patient distributions/2021_11_23_15_3_PAAD.pdf]

# CHOL\_FEMALE

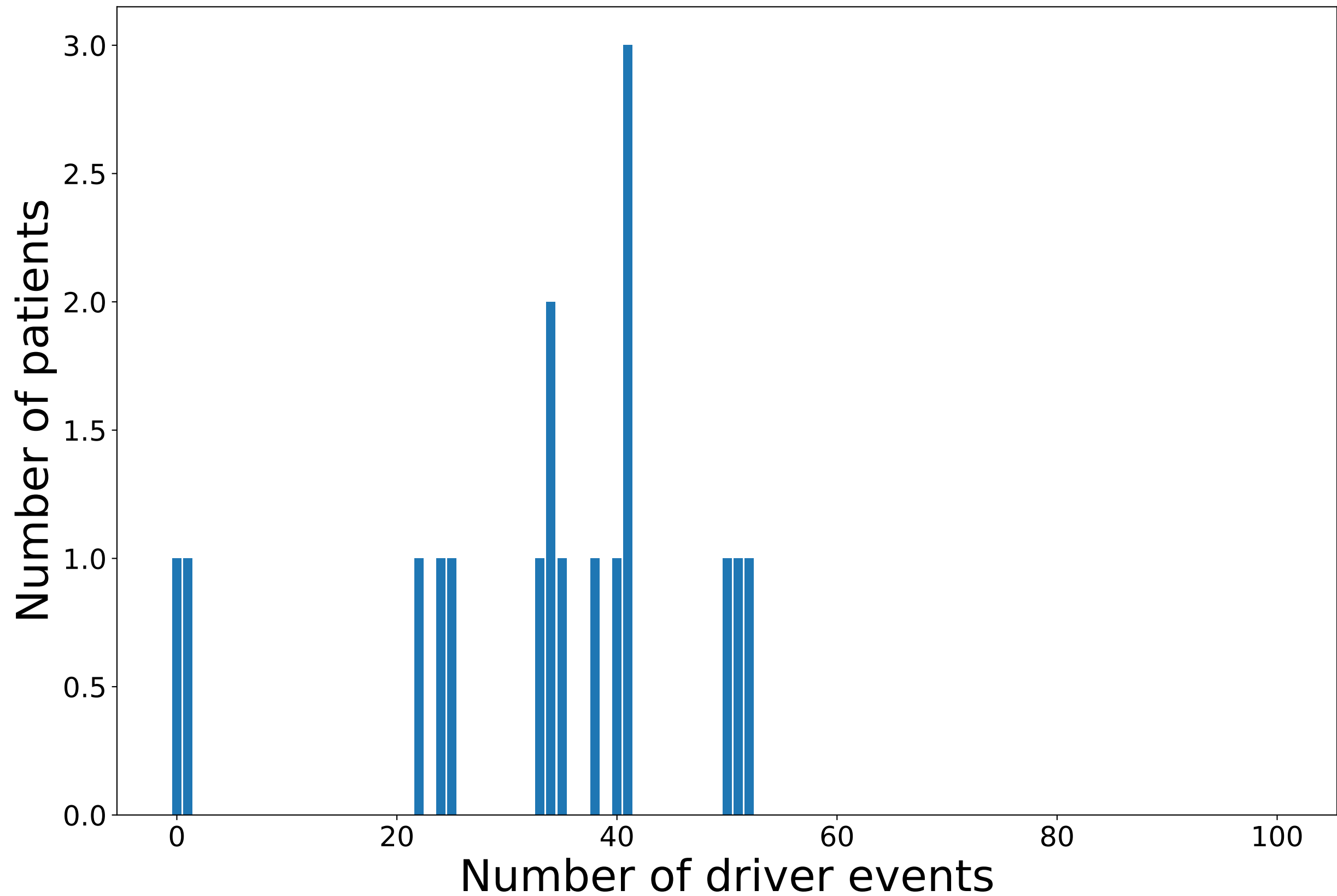

Supplement: S4 Files — (ZIP) [file pgen.1009996.s004.zip › Aneuploidy/PANCAN GISTIC2/patient distributions/2021_11_23_15_3_CHOL_FEMALE.pdf]

# UVM\_MALE

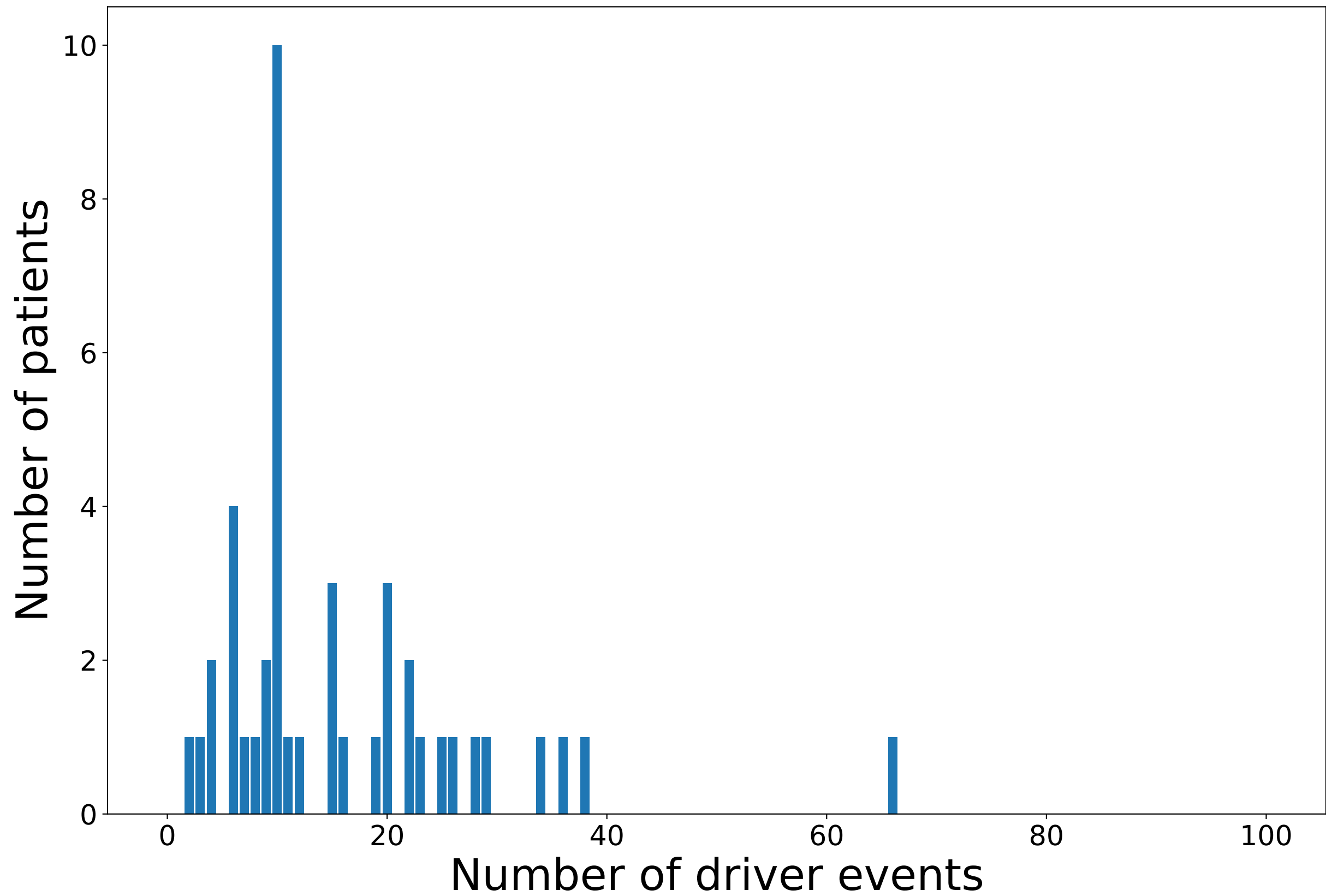

Supplement: S4 Files — (ZIP) [file pgen.1009996.s004.zip › Aneuploidy/PANCAN GISTIC2/patient distributions/2021_11_23_15_3_UVM_MALE.pdf]

# ESCA

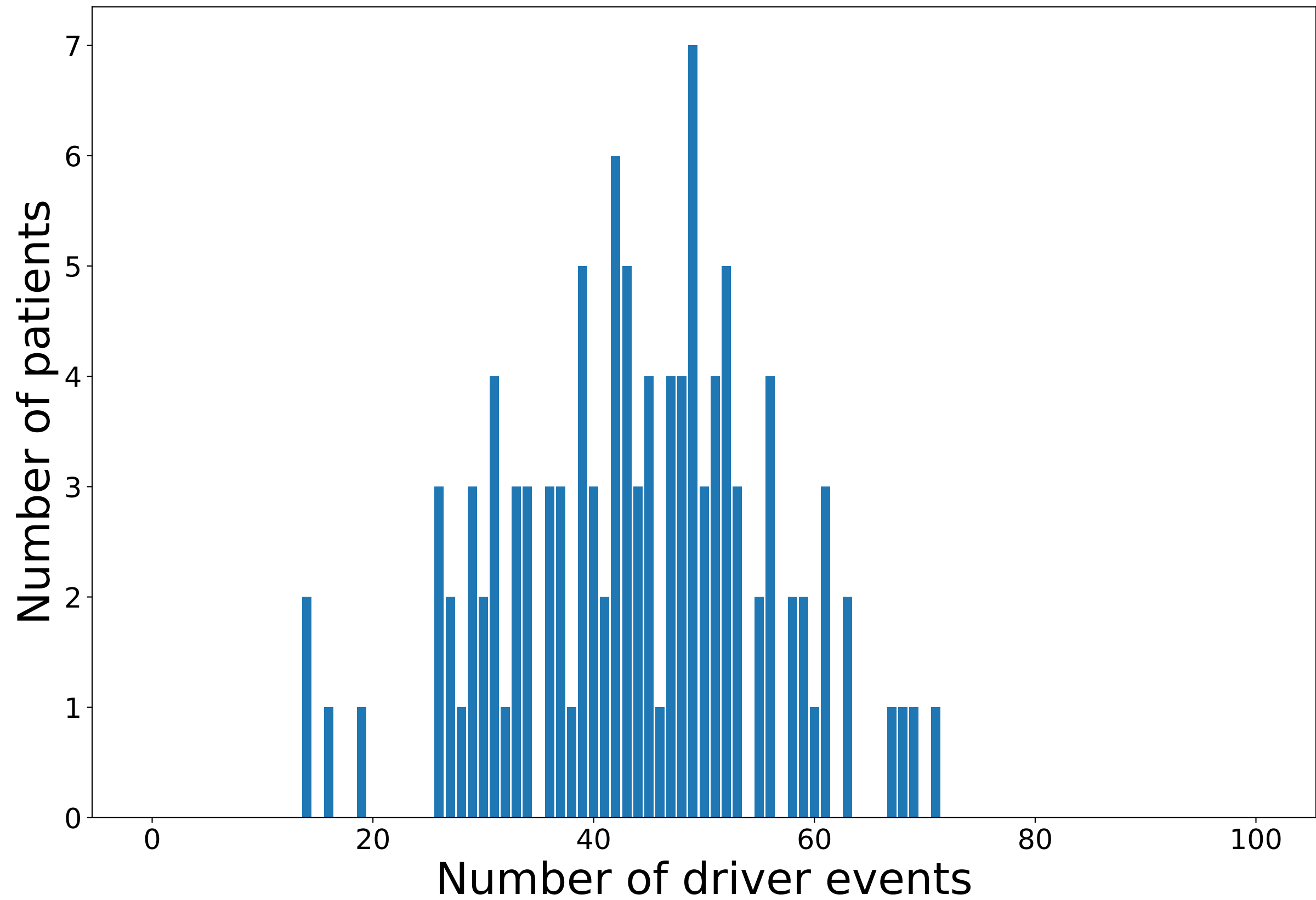

Supplement: S4 Files — (ZIP) [file pgen.1009996.s004.zip › Aneuploidy/PANCAN GISTIC2/patient distributions/2021_11_23_15_3_ESCA.pdf]

# STAD\_MALE

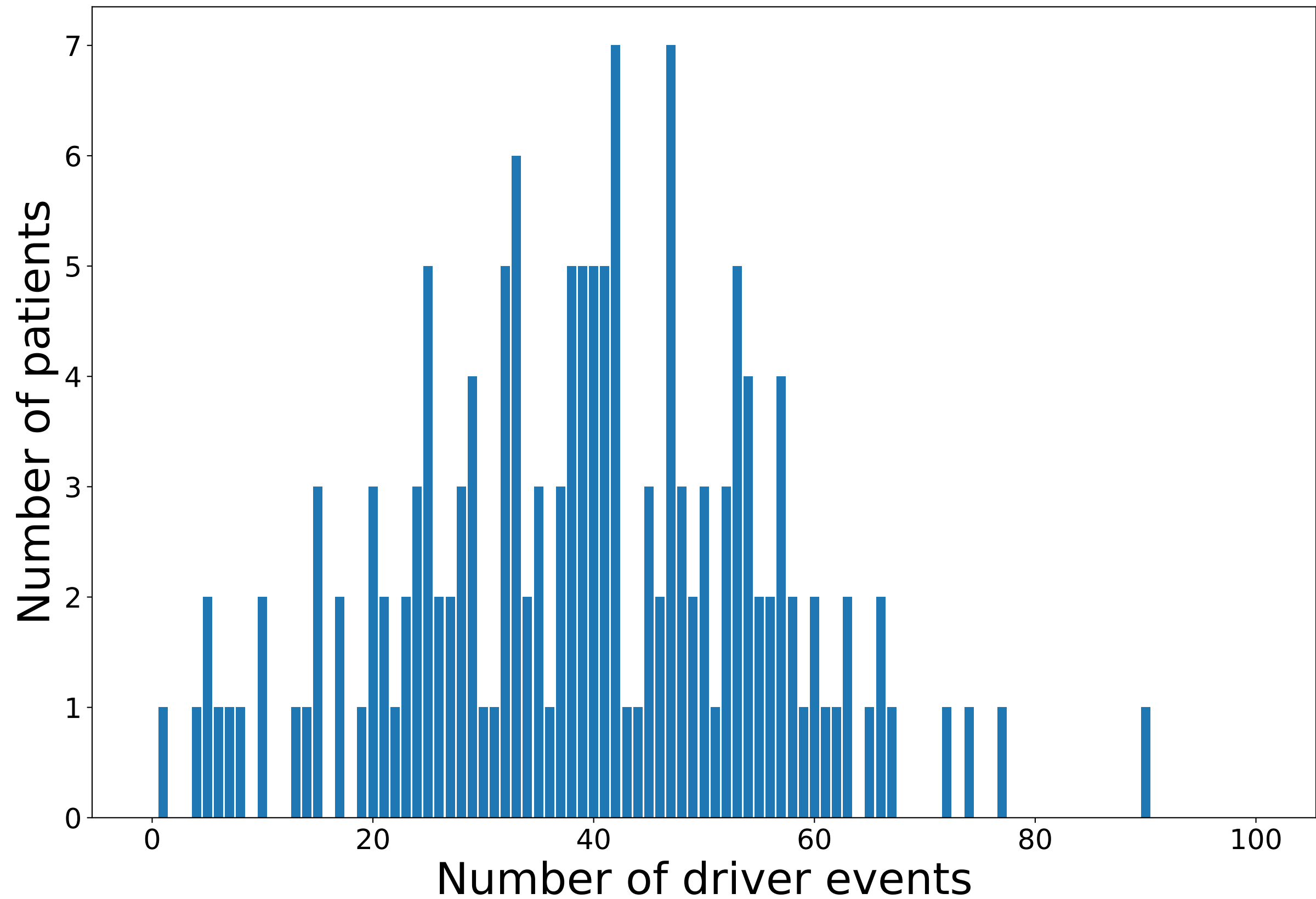

Supplement: S4 Files — (ZIP) [file pgen.1009996.s004.zip › Aneuploidy/PANCAN GISTIC2/patient distributions/2021_11_23_15_3_STAD_MALE.pdf]

# KIRP\_FEMALE

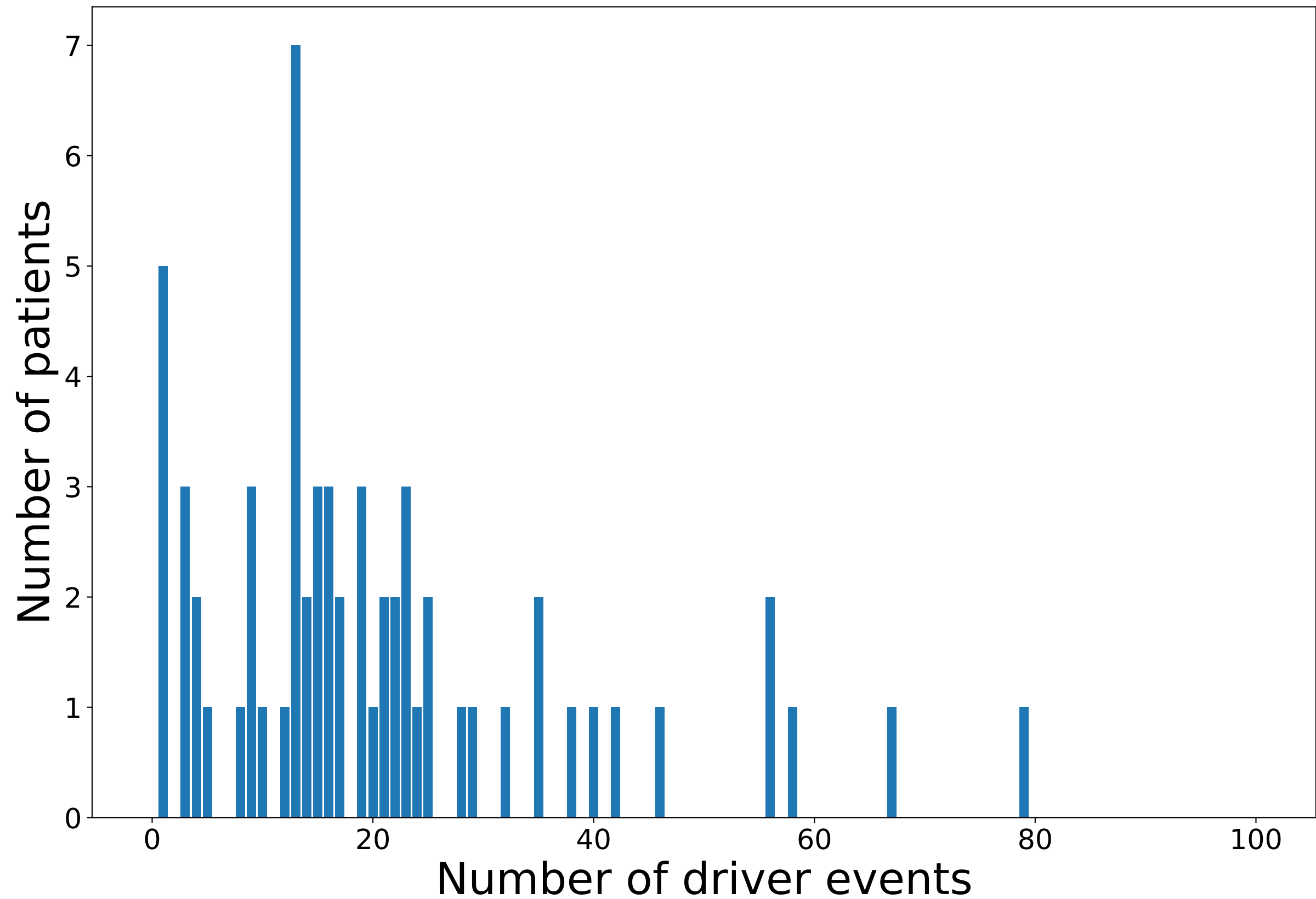

Supplement: S4 Files — (ZIP) [file pgen.1009996.s004.zip › Aneuploidy/PANCAN GISTIC2/patient distributions/2021_11_23_15_3_KIRP_FEMALE.pdf]

# HNSC

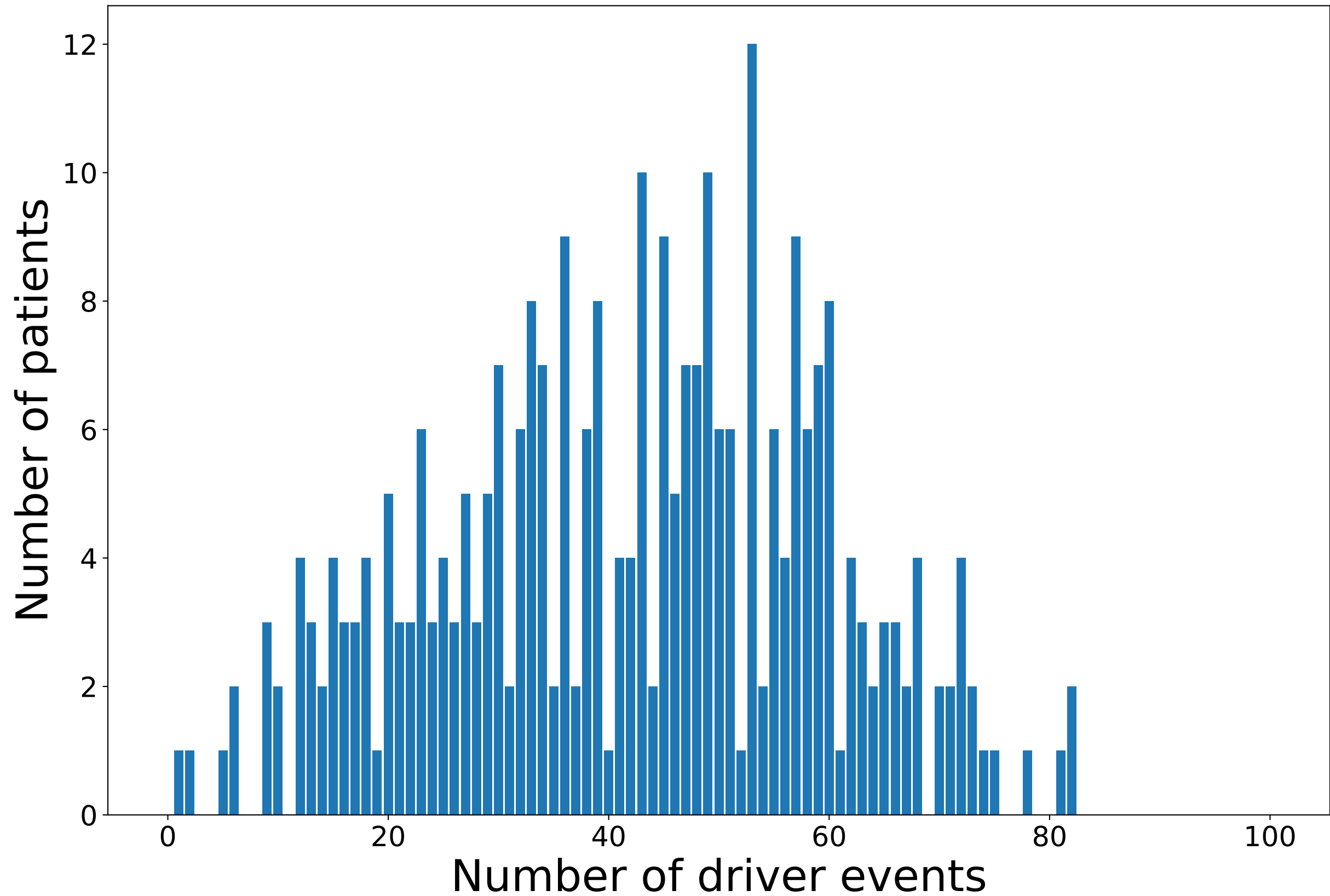

Supplement: S4 Files — (ZIP) [file pgen.1009996.s004.zip › Aneuploidy/PANCAN GISTIC2/patient distributions/2021_11_23_15_3_HNSC.pdf]

# UVM

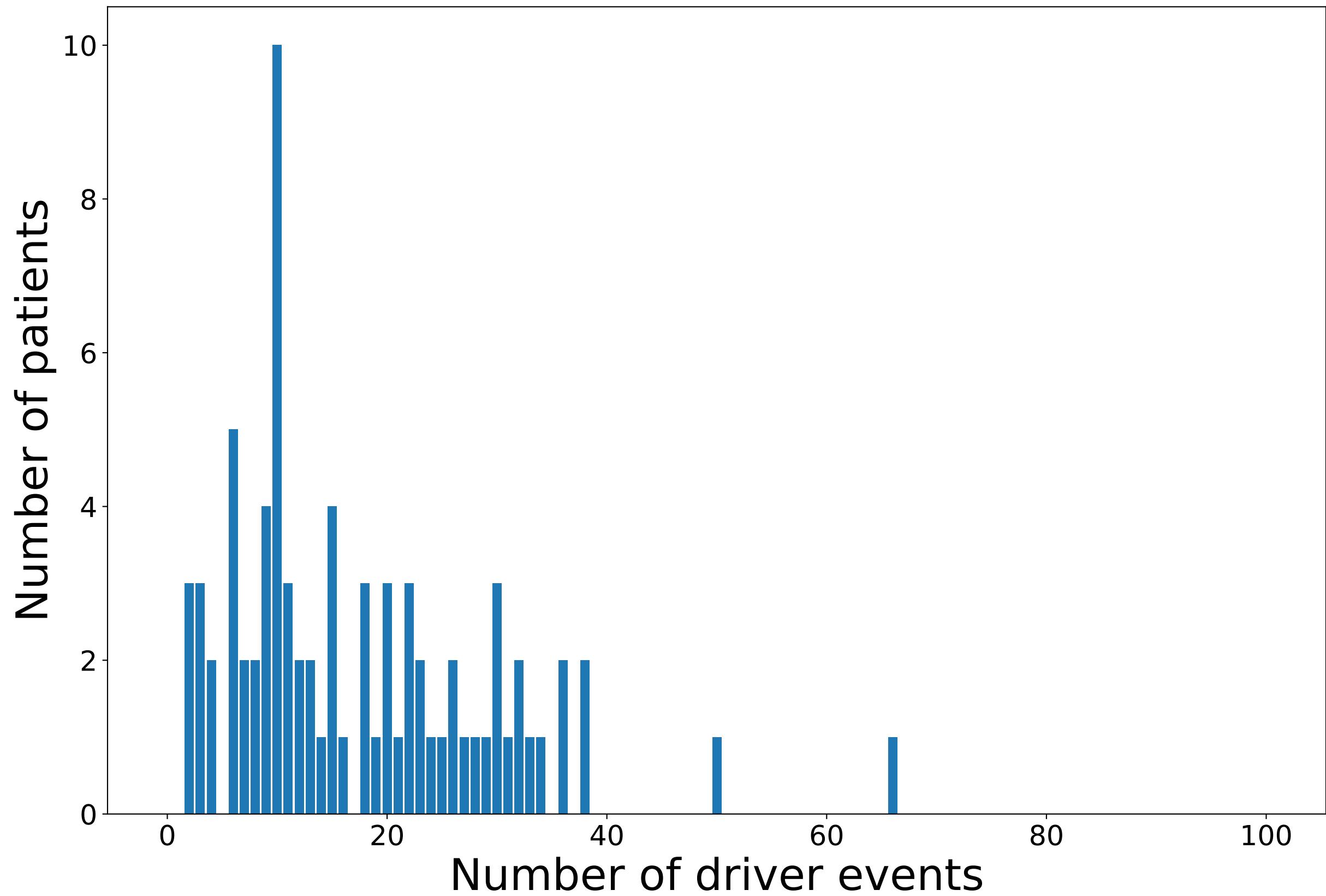

Supplement: S4 Files — (ZIP) [file pgen.1009996.s004.zip › Aneuploidy/PANCAN GISTIC2/patient distributions/2021_11_23_15_3_UVM.pdf]

# THYM\_MALE

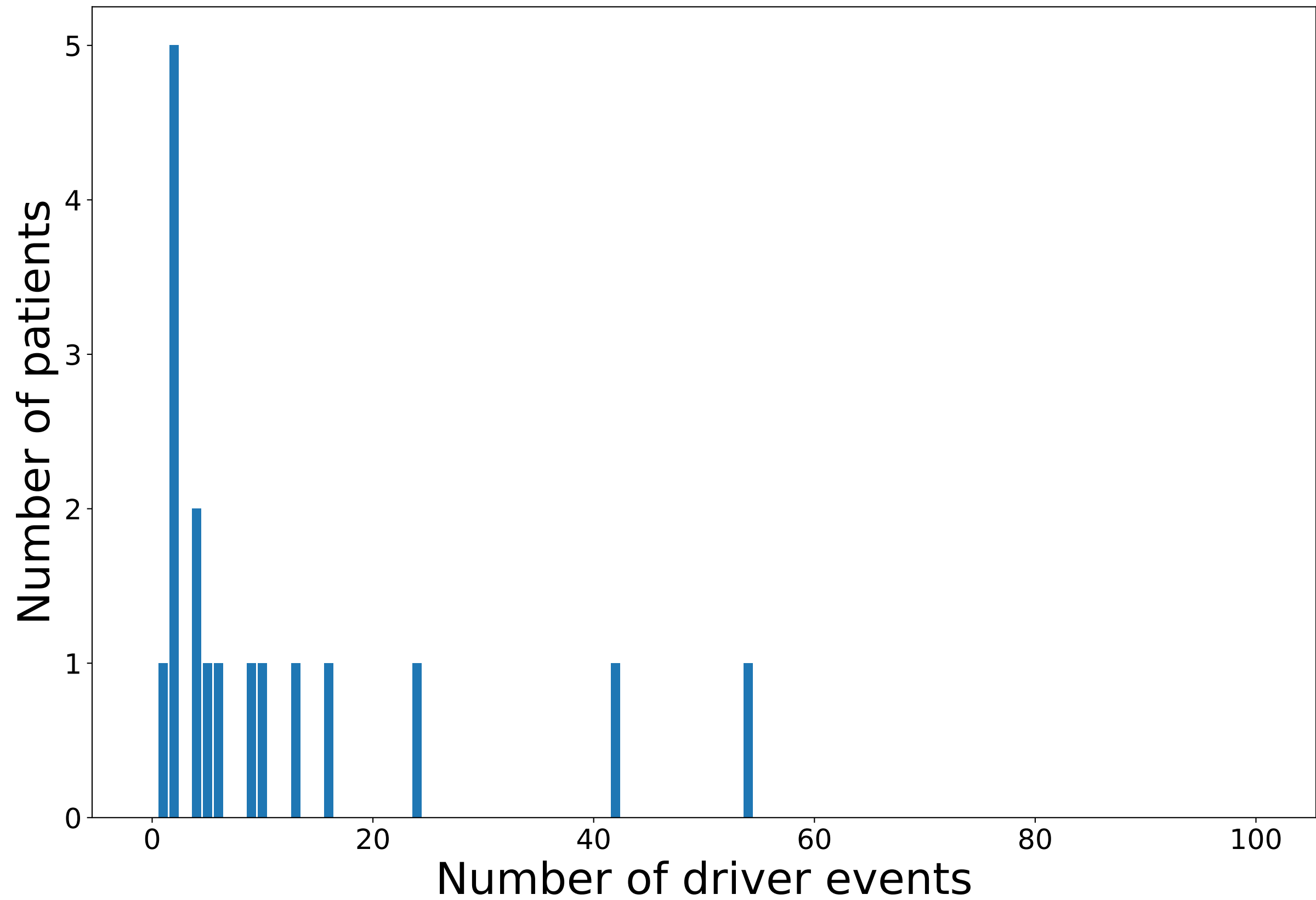

Supplement: S4 Files — (ZIP) [file pgen.1009996.s004.zip › Aneuploidy/PANCAN GISTIC2/patient distributions/2021_11_23_15_3_THYM_MALE.pdf]

# KIRP\_MALE

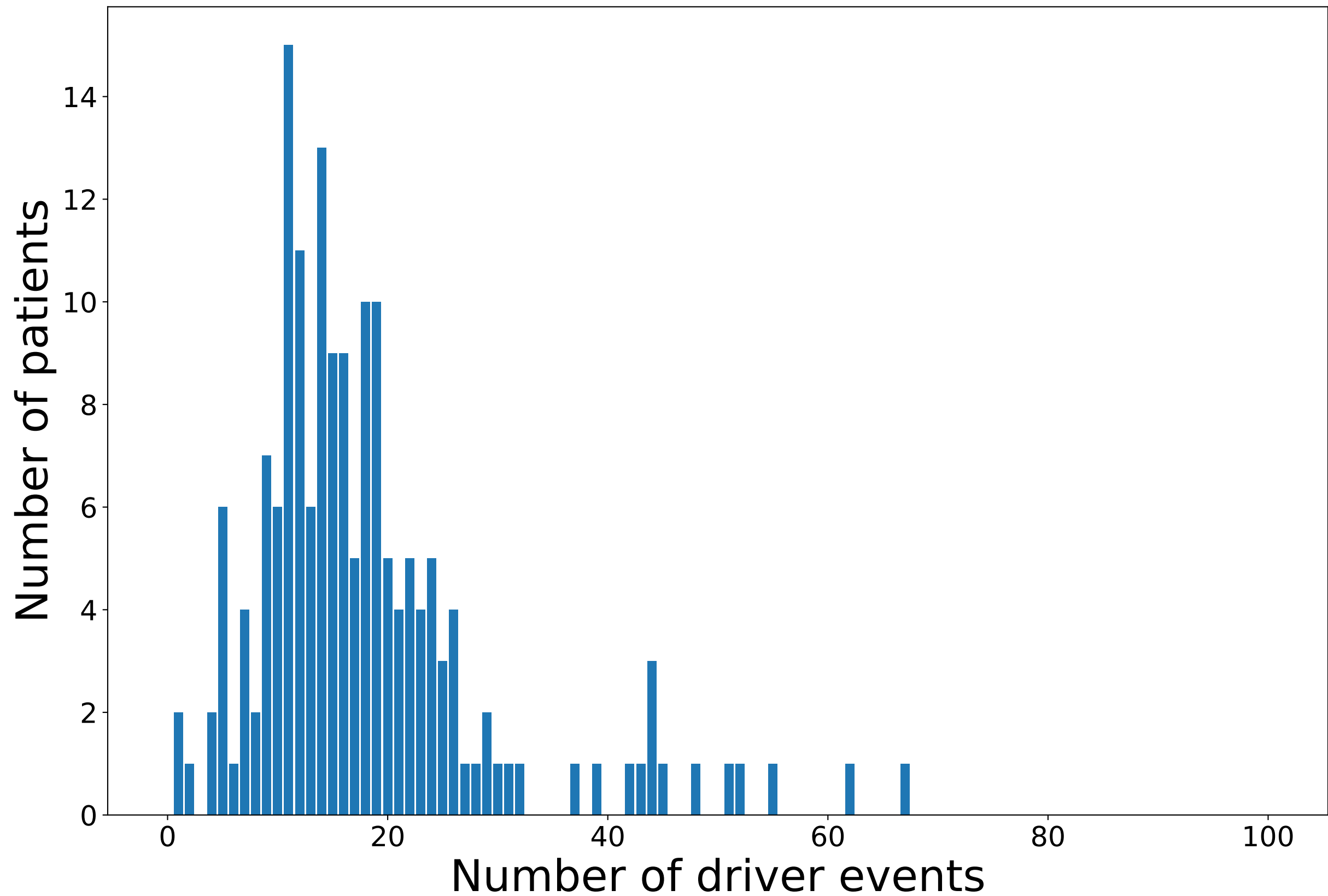

Supplement: S4 Files — (ZIP) [file pgen.1009996.s004.zip › Aneuploidy/PANCAN GISTIC2/patient distributions/2021_11_23_15_3_KIRP_MALE.pdf]

# BLCA\_FEMALE

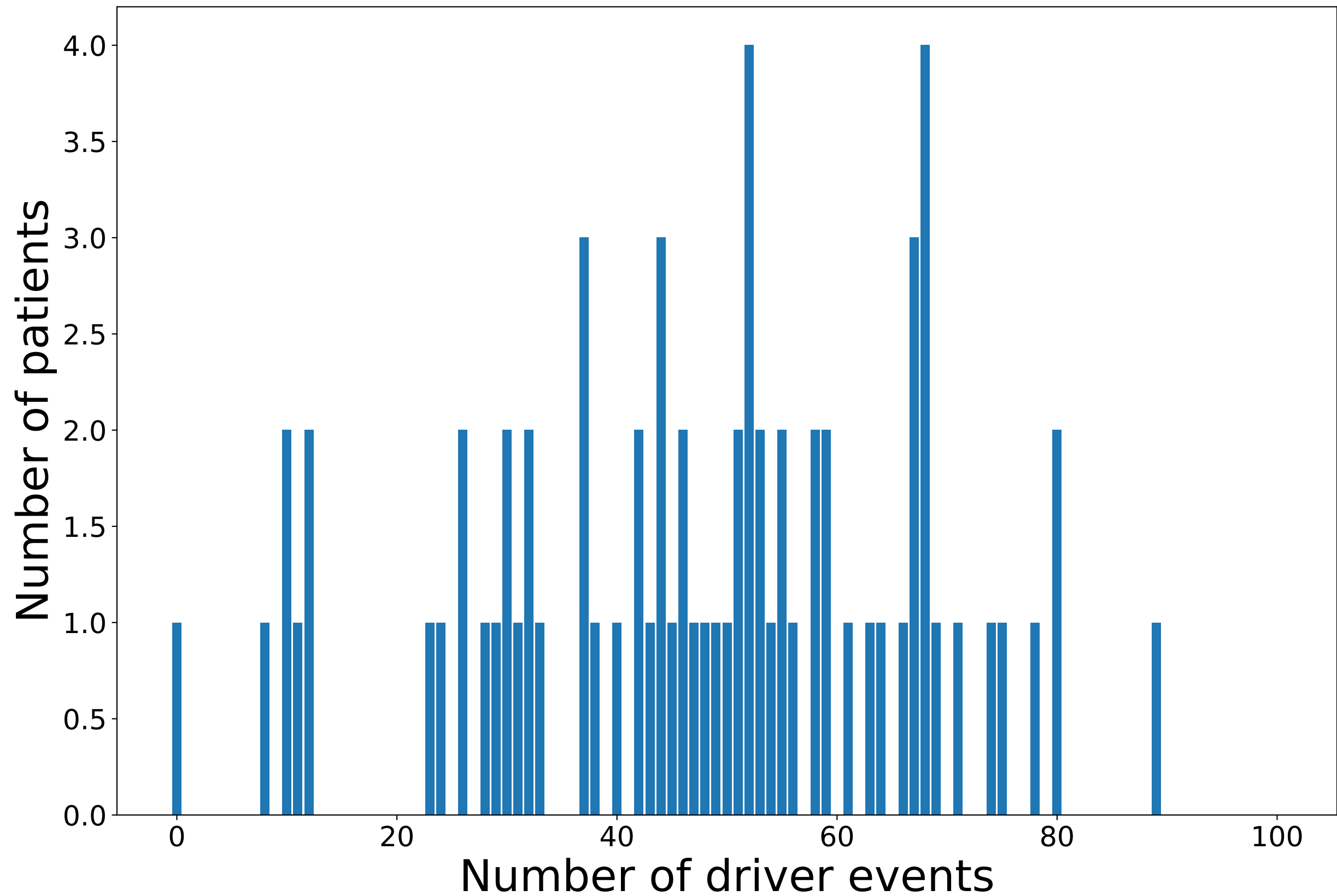

Supplement: S4 Files — (ZIP) [file pgen.1009996.s004.zip › Aneuploidy/PANCAN GISTIC2/patient distributions/2021_11_23_15_3_BLCA_FEMALE.pdf]

# HNSC\_MALE

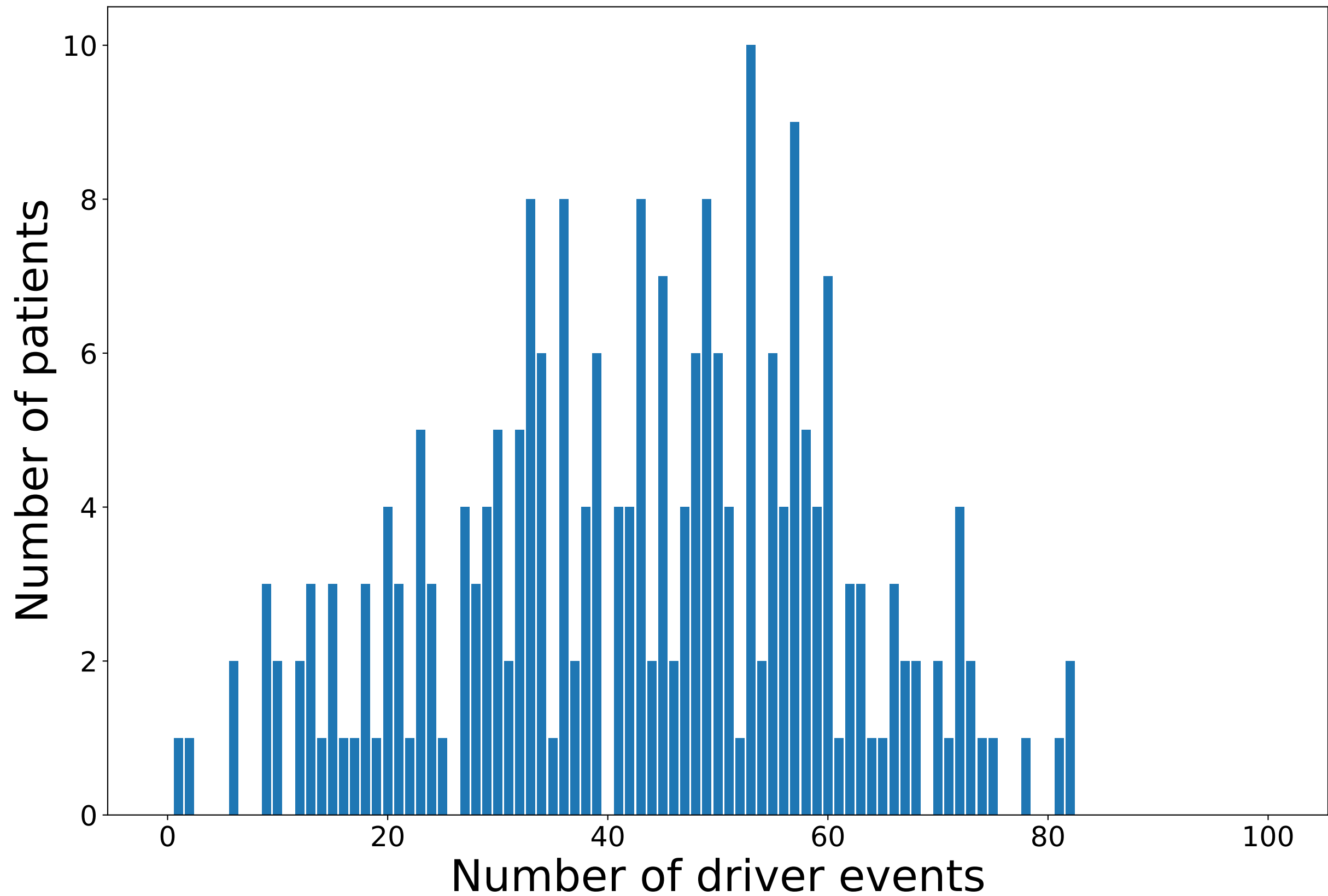

Supplement: S4 Files — (ZIP) [file pgen.1009996.s004.zip › Aneuploidy/PANCAN GISTIC2/patient distributions/2021_11_23_15_3_HNSC_MALE.pdf]

# LIHC\_FEMALE

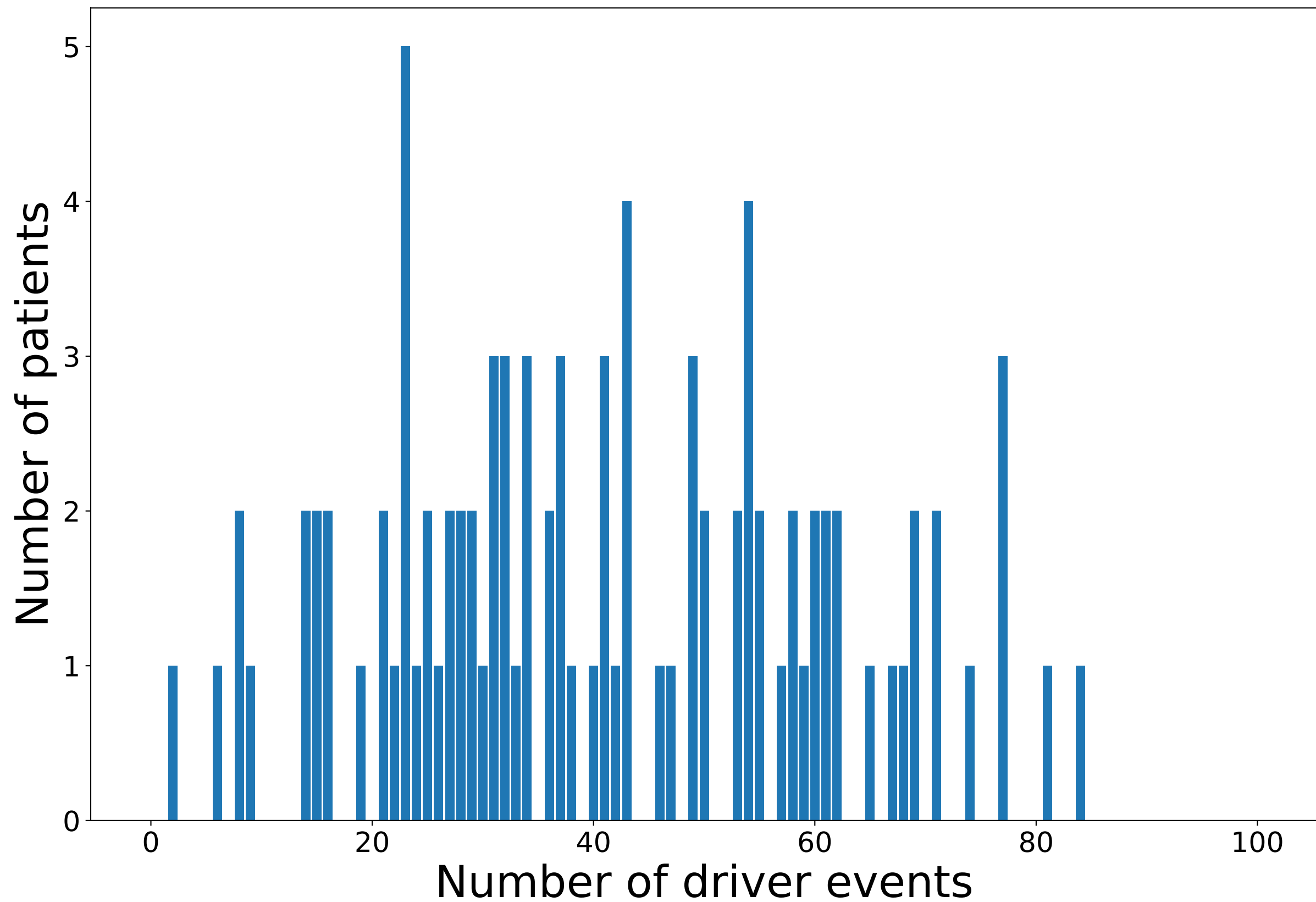

Supplement: S4 Files — (ZIP) [file pgen.1009996.s004.zip › Aneuploidy/PANCAN GISTIC2/patient distributions/2021_11_23_15_3_LIHC_FEMALE.pdf]

# KIRC\_FEMALE

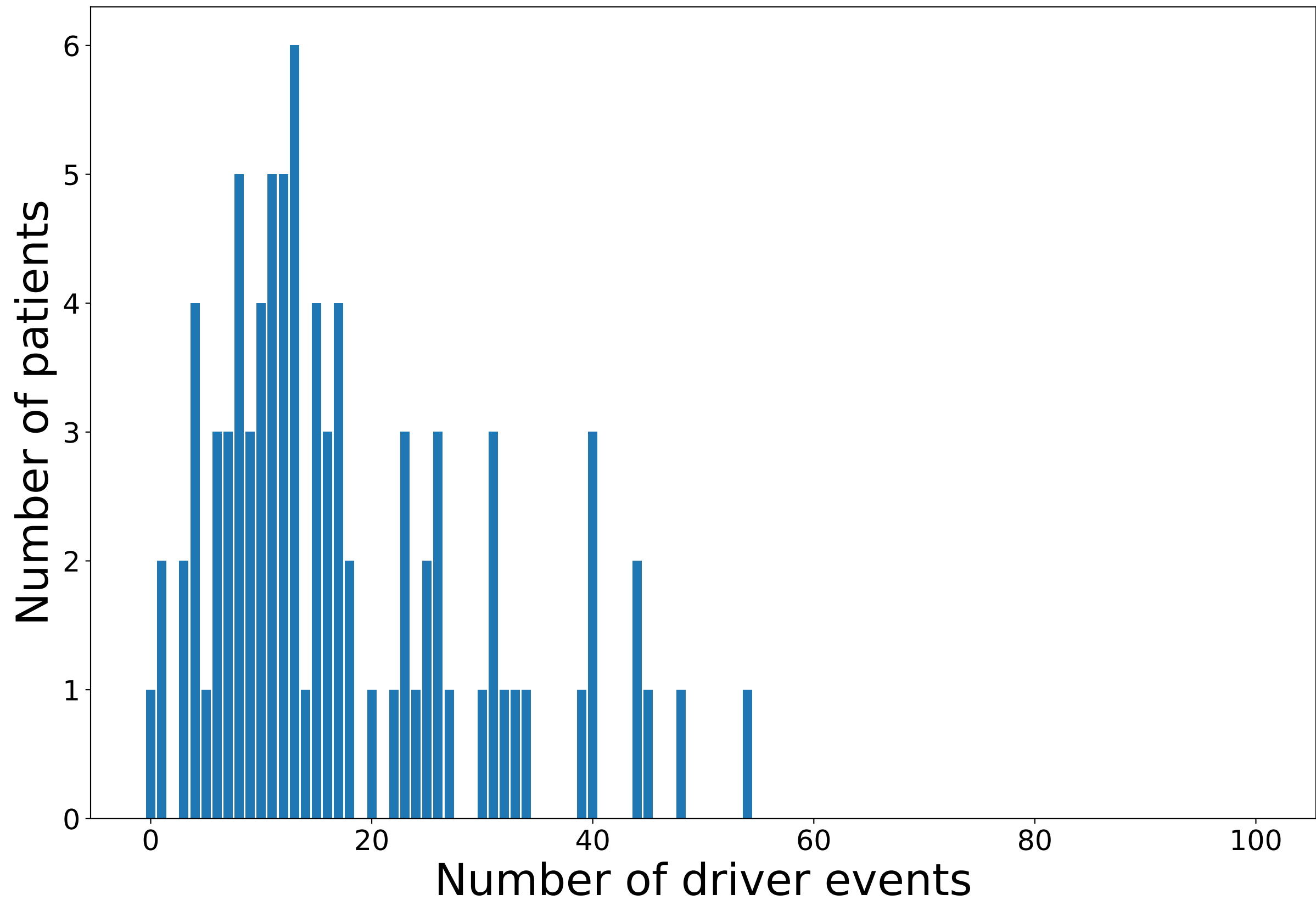

Supplement: S4 Files — (ZIP) [file pgen.1009996.s004.zip › Aneuploidy/PANCAN GISTIC2/patient distributions/2021_11_23_15_3_KIRC_FEMALE.pdf]

# SARC

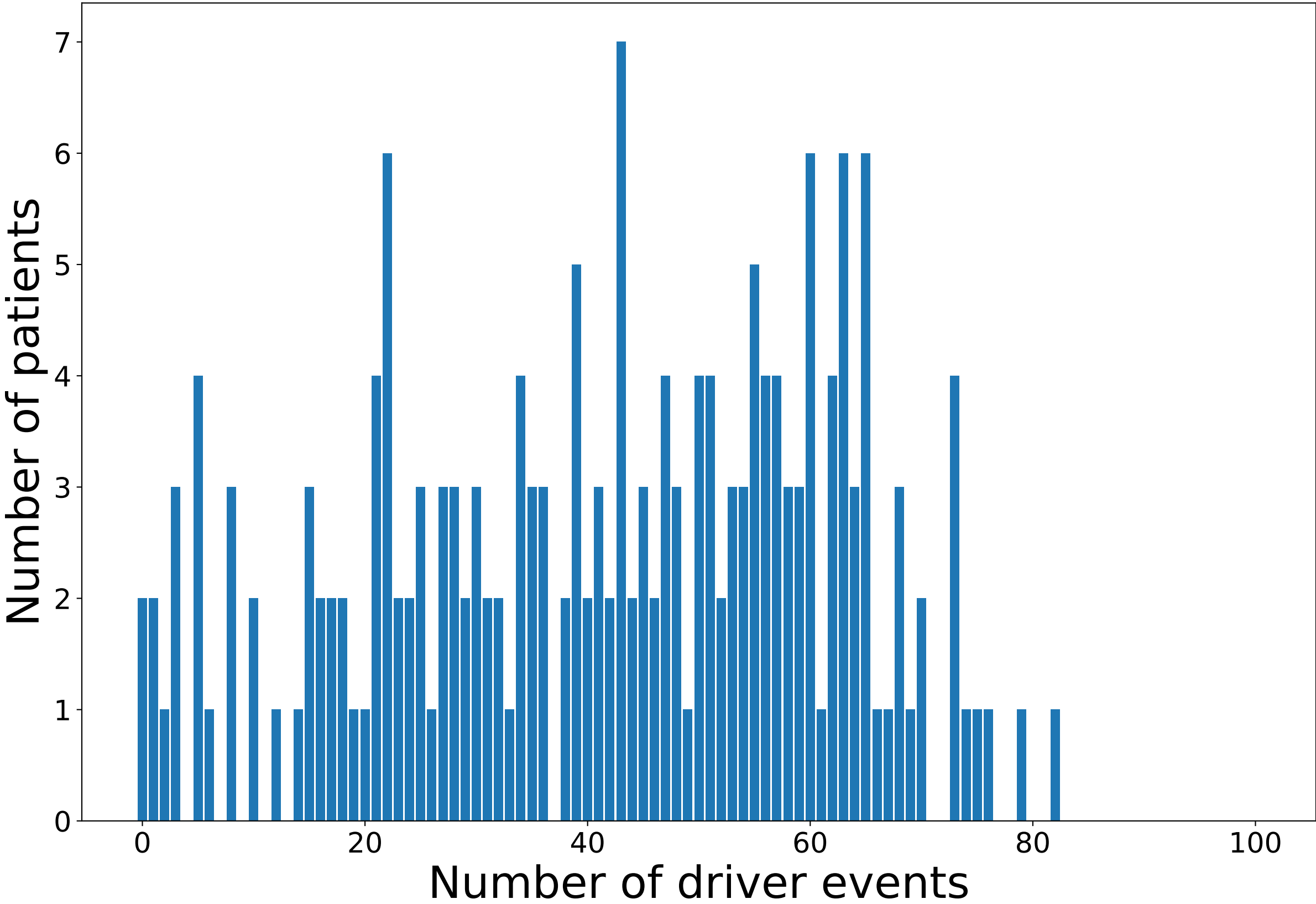

Supplement: S4 Files — (ZIP) [file pgen.1009996.s004.zip › Aneuploidy/PANCAN GISTIC2/patient distributions/2021_11_23_15_3_SARC.pdf]

# OV\_FEMALE

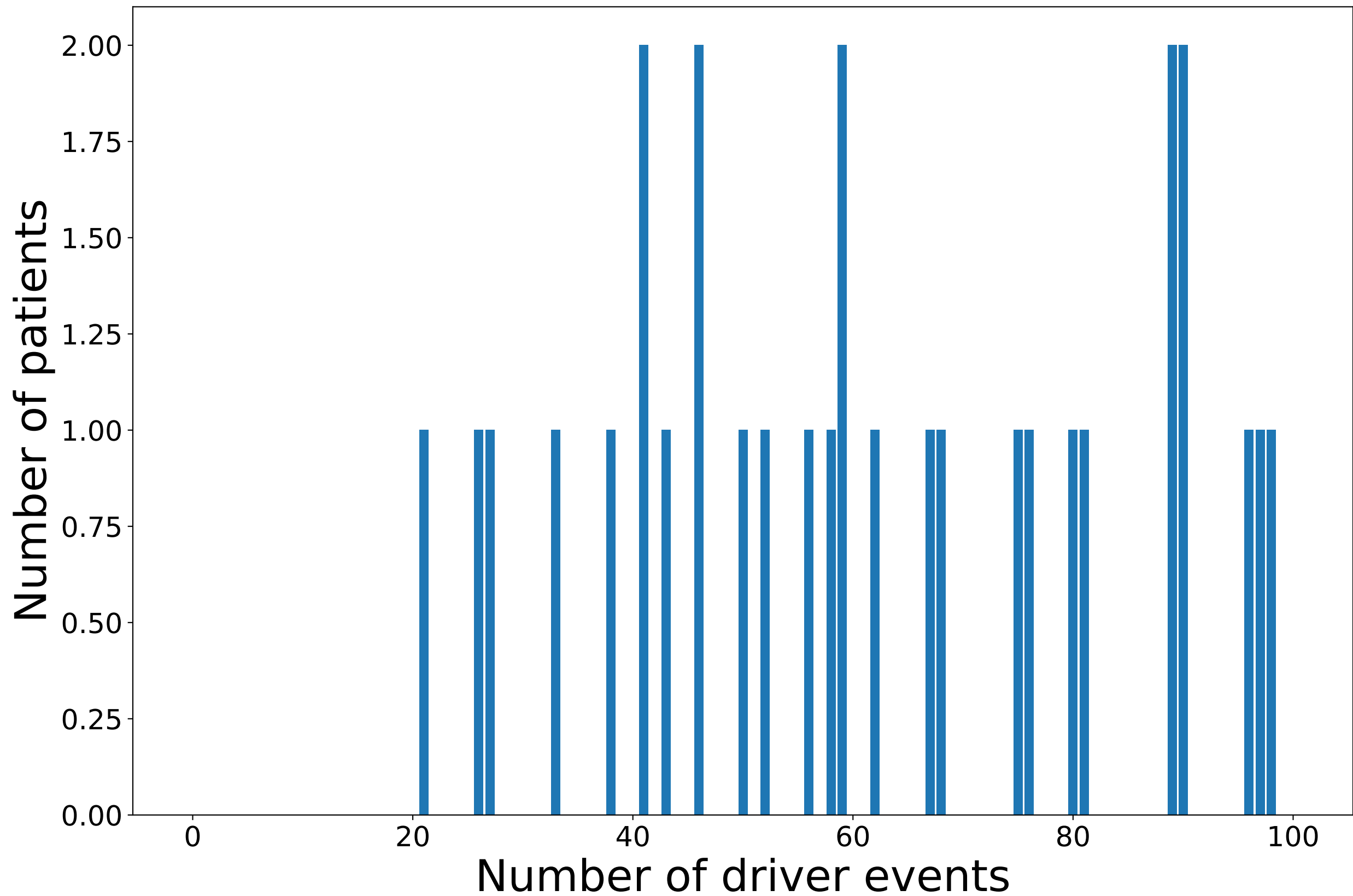

Supplement: S4 Files — (ZIP) [file pgen.1009996.s004.zip › Aneuploidy/PANCAN GISTIC2/patient distributions/2021_11_23_15_3_OV_FEMALE.pdf]

# LUAD

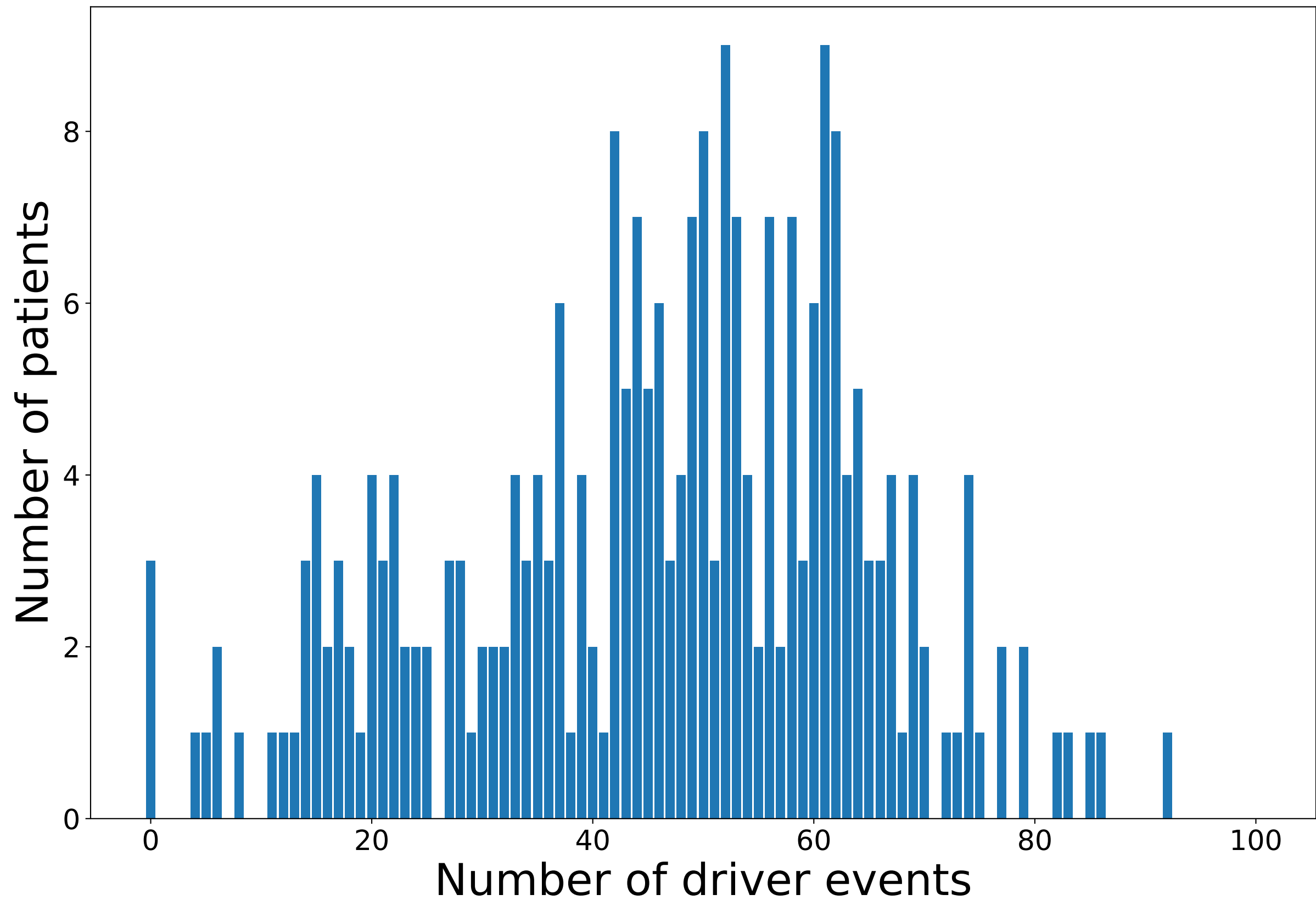

Supplement: S4 Files — (ZIP) [file pgen.1009996.s004.zip › Aneuploidy/PANCAN GISTIC2/patient distributions/2021_11_23_15_3_LUAD.pdf]

# BRCA\_FEMALE

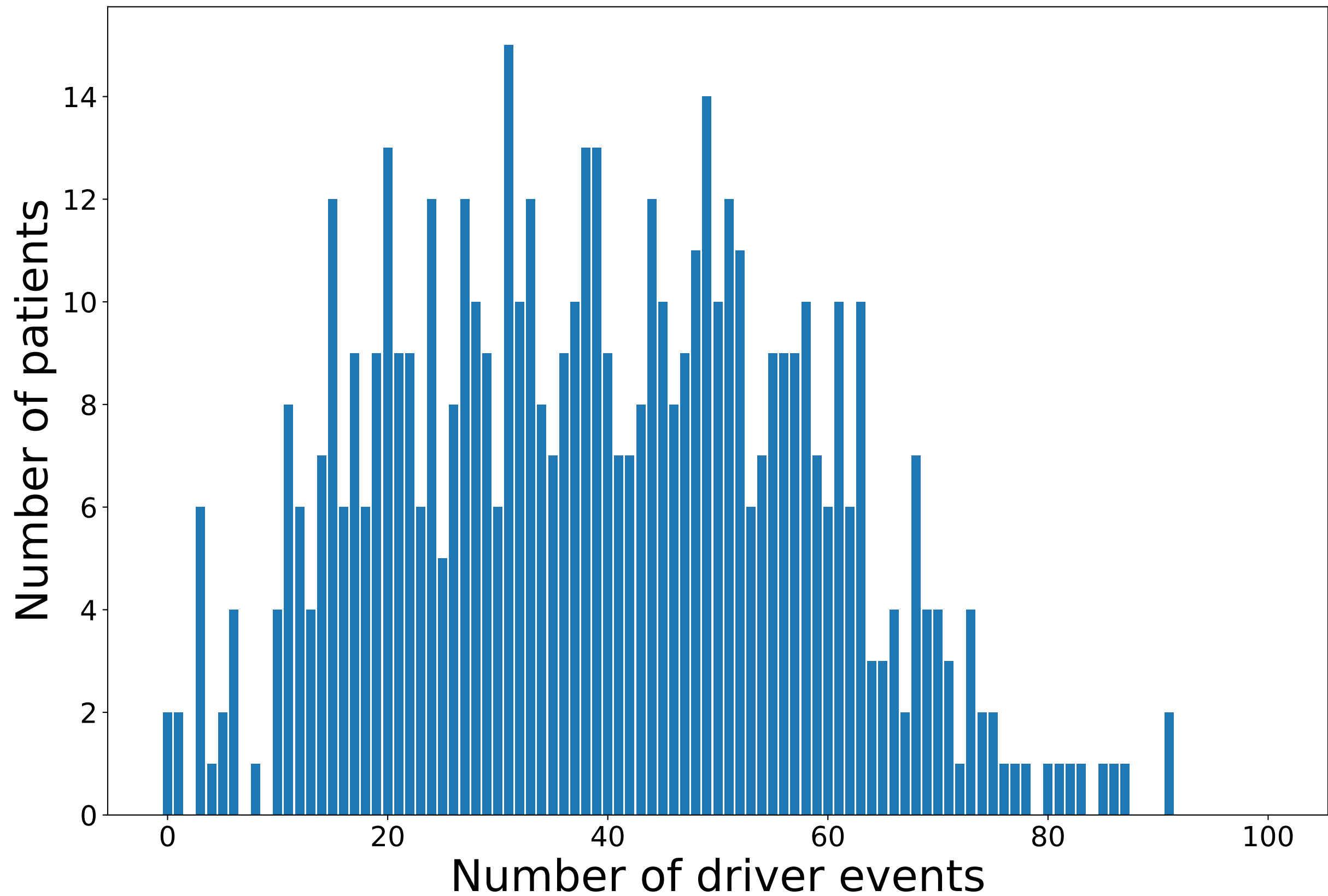

Supplement: S4 Files — (ZIP) [file pgen.1009996.s004.zip › Aneuploidy/PANCAN GISTIC2/patient distributions/2021_11_23_15_3_BRCA_FEMALE.pdf]

# ACC\_FEMALE

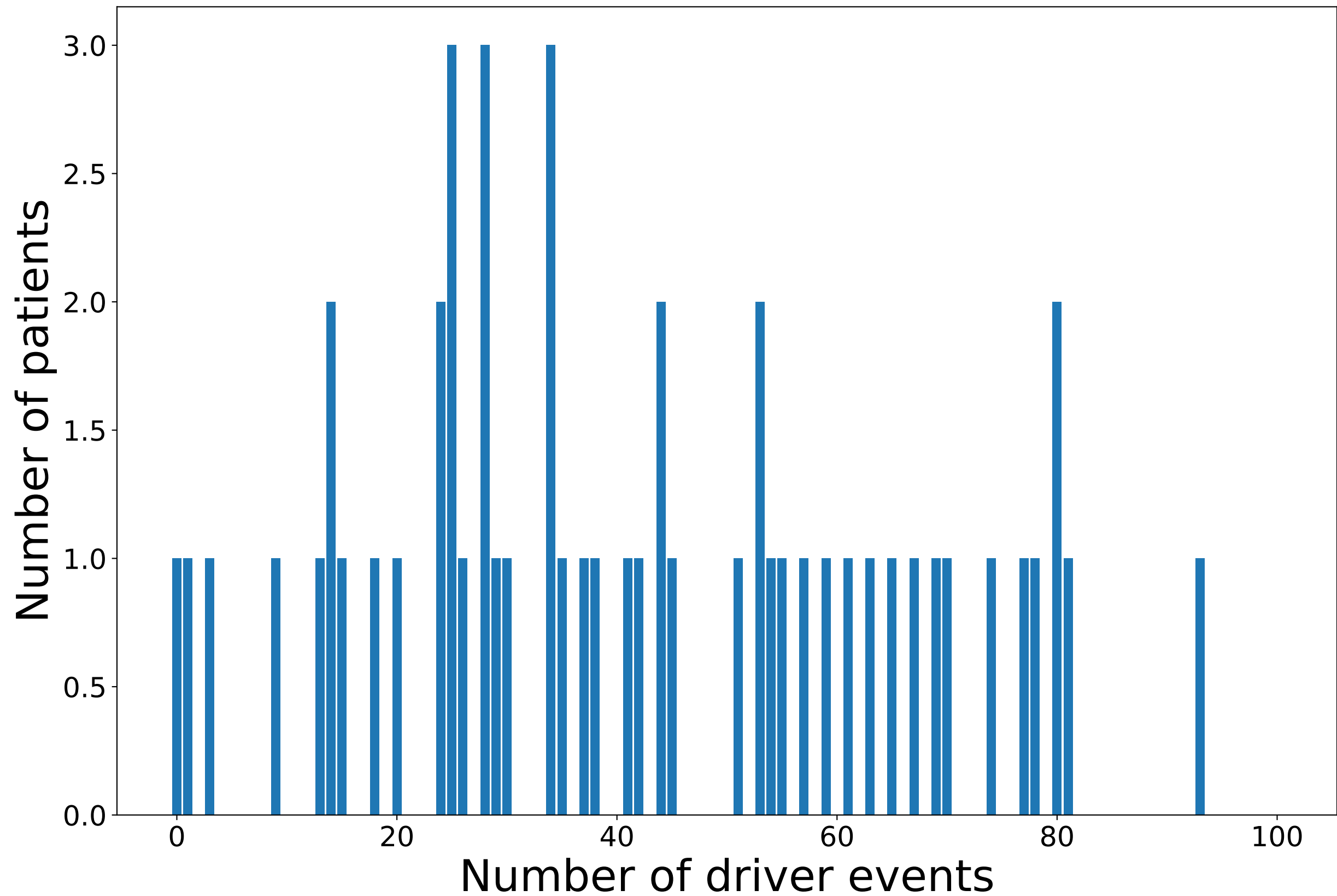

Supplement: S4 Files — (ZIP) [file pgen.1009996.s004.zip › Aneuploidy/PANCAN GISTIC2/patient distributions/2021_11_23_15_3_ACC_FEMALE.pdf]

# DLBC\_FEMALE

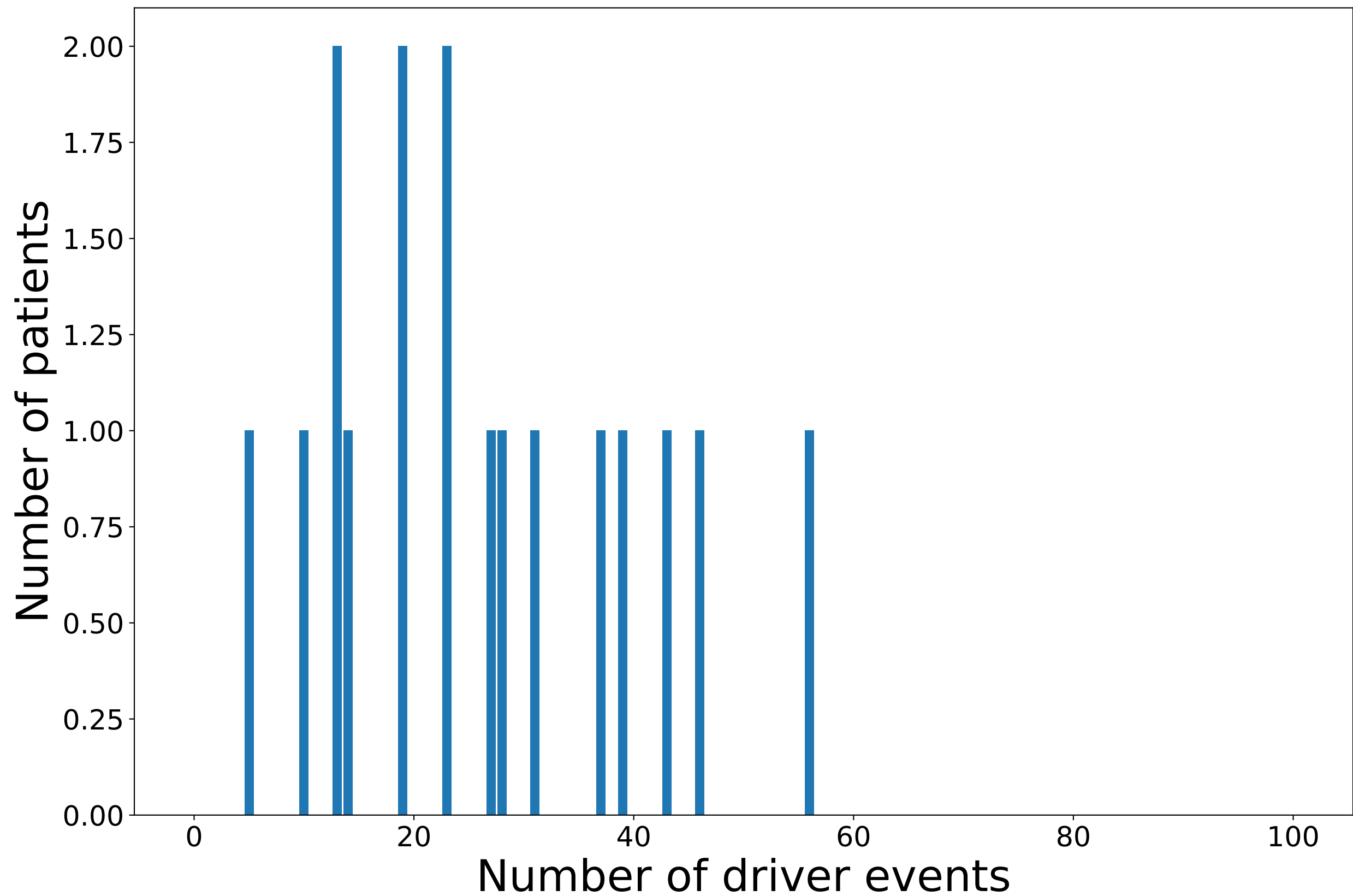

Supplement: S4 Files — (ZIP) [file pgen.1009996.s004.zip › Aneuploidy/PANCAN GISTIC2/patient distributions/2021_11_23_15_3_DLBC_FEMALE.pdf]

# ESCA\_MALE

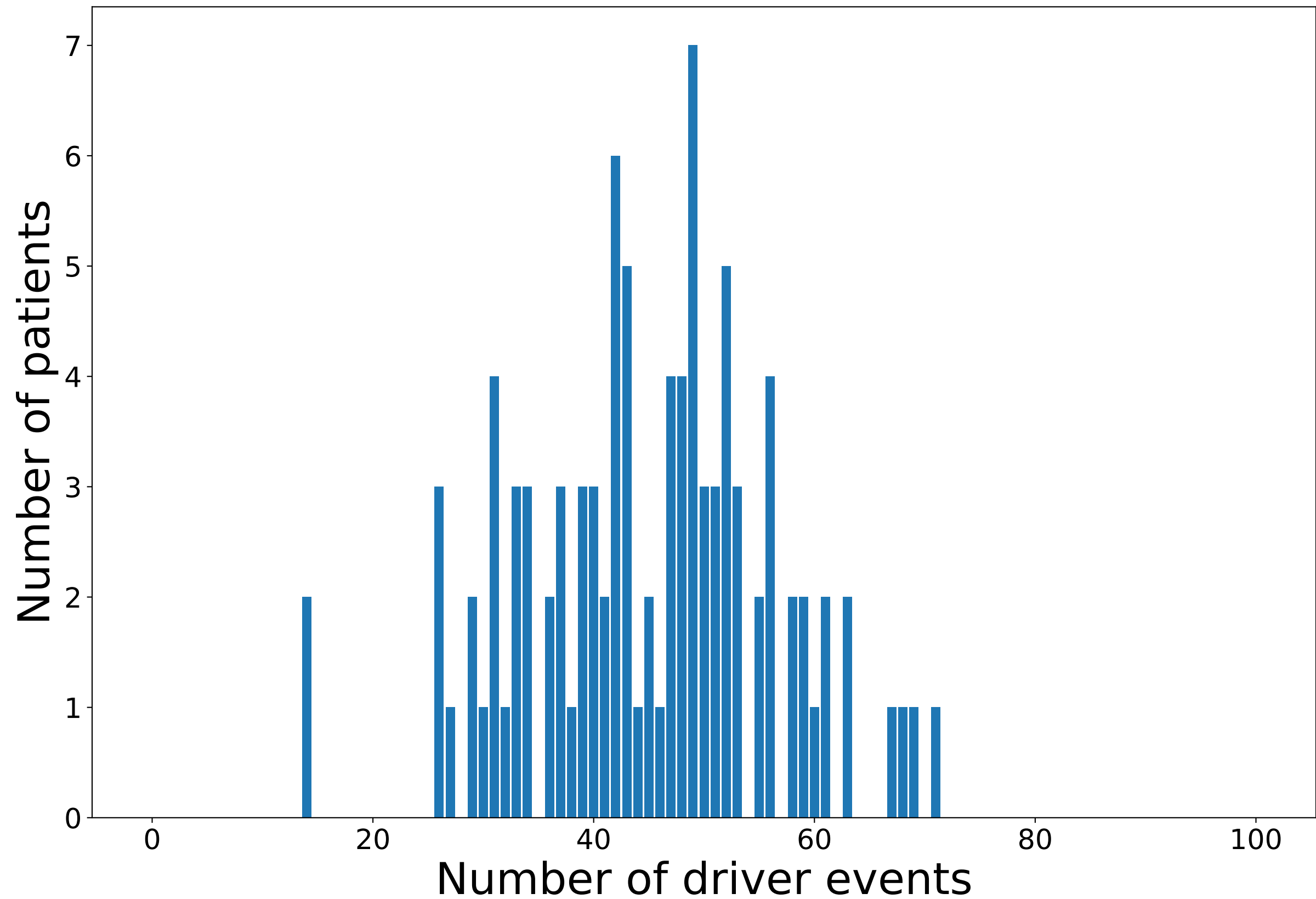

Supplement: S4 Files — (ZIP) [file pgen.1009996.s004.zip › Aneuploidy/PANCAN GISTIC2/patient distributions/2021_11_23_15_3_ESCA_MALE.pdf]

# KICH\_FEMALE

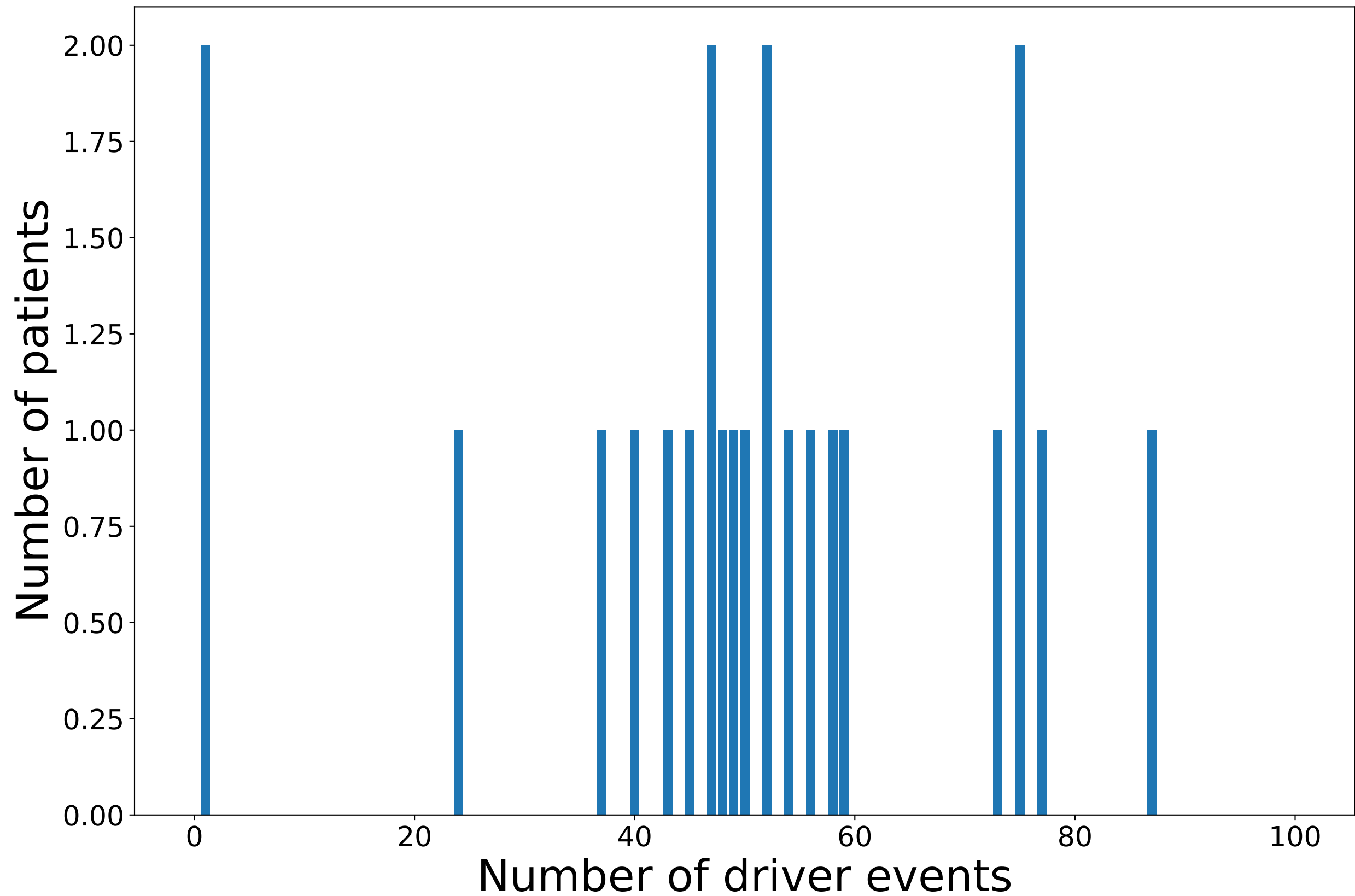

Supplement: S4 Files — (ZIP) [file pgen.1009996.s004.zip › Aneuploidy/PANCAN GISTIC2/patient distributions/2021_11_23_15_3_KICH_FEMALE.pdf]

PRAD

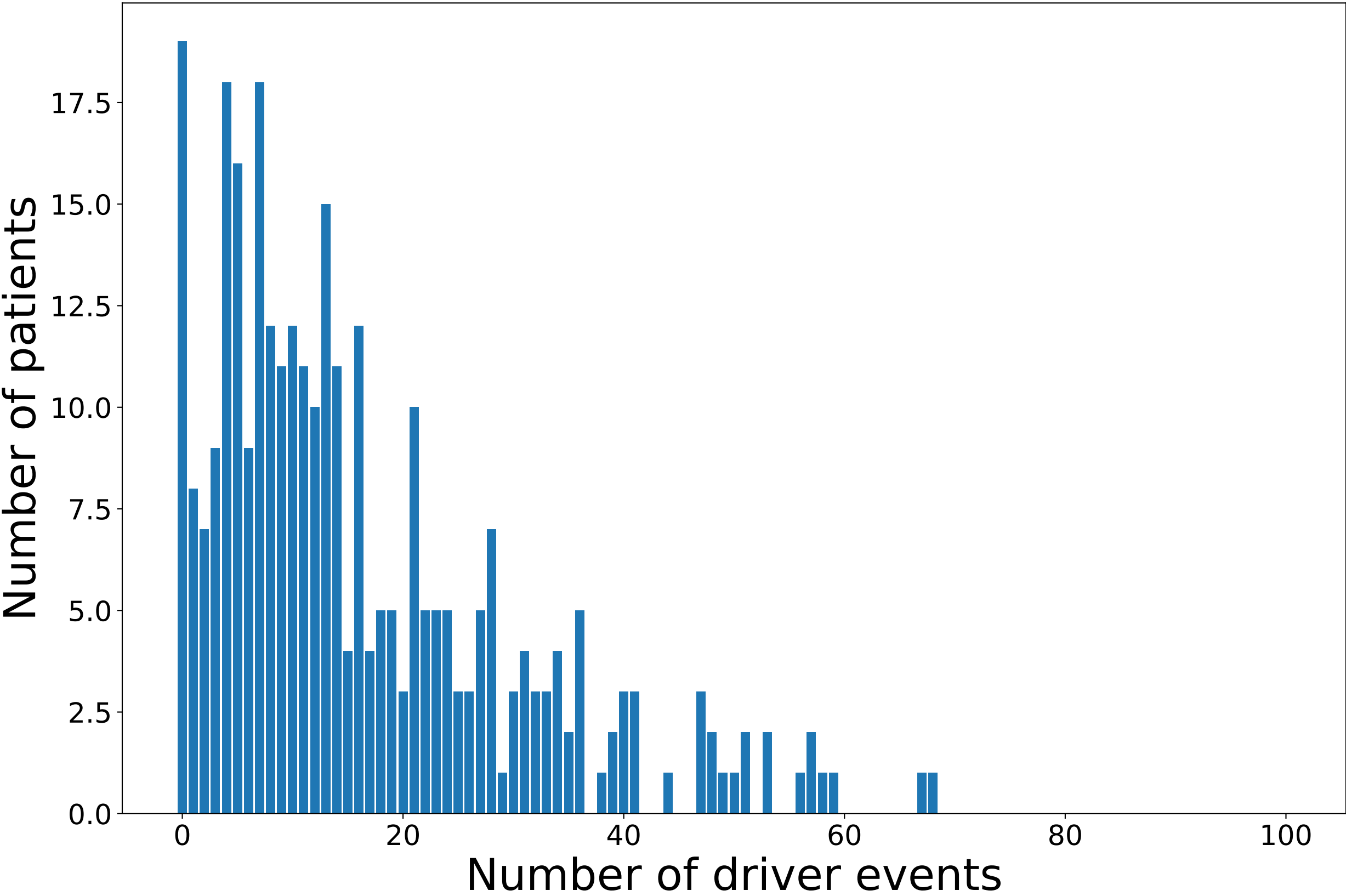

Supplement: S4 Files — (ZIP) [file pgen.1009996.s004.zip › Aneuploidy/PANCAN GISTIC2/patient distributions/2021_11_23_15_3_PRAD.pdf]

# LIHC\_MALE

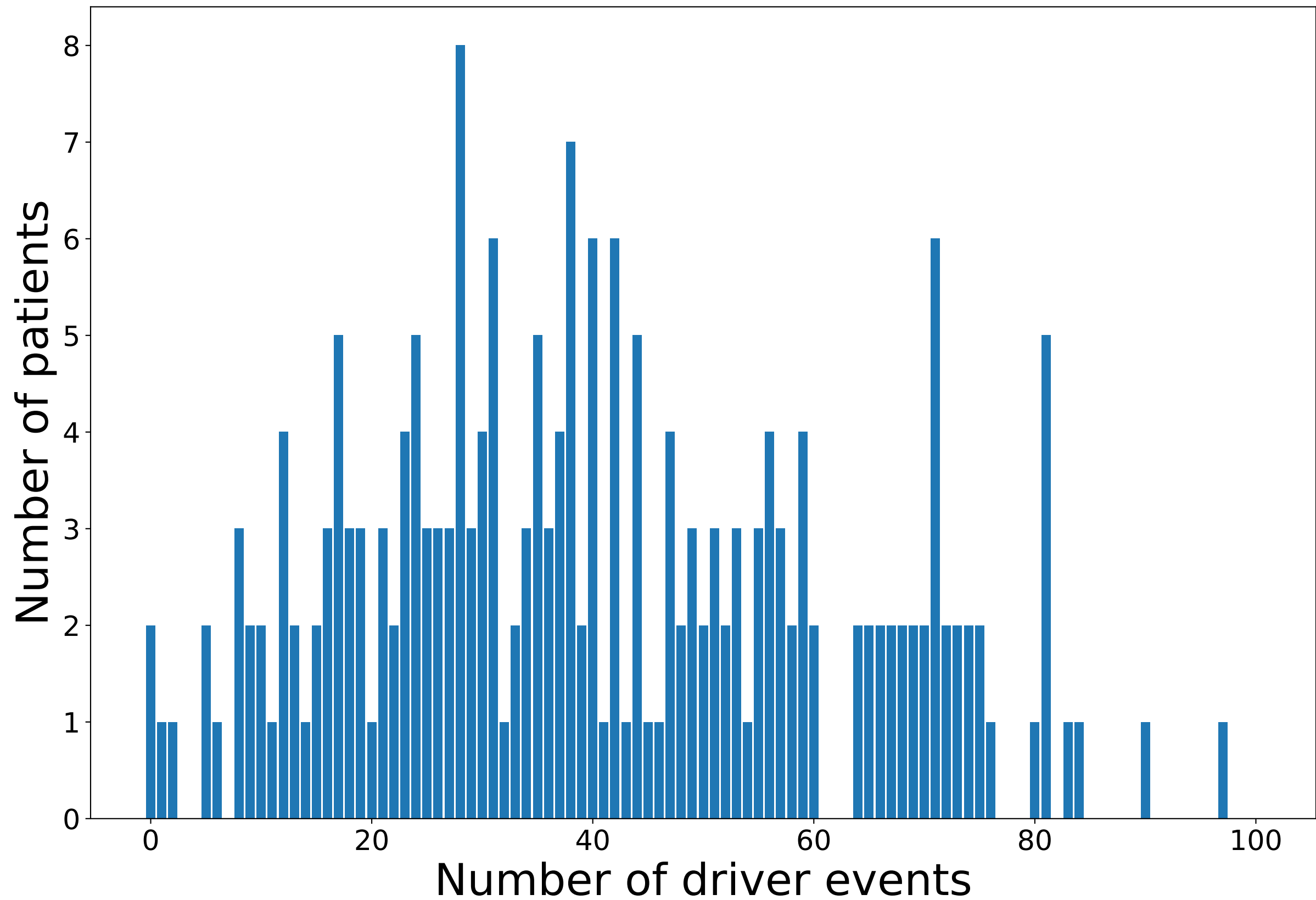

Supplement: S4 Files — (ZIP) [file pgen.1009996.s004.zip › Aneuploidy/PANCAN GISTIC2/patient distributions/2021_11_23_15_3_LIHC_MALE.pdf]

# ESCA\_FEMALE

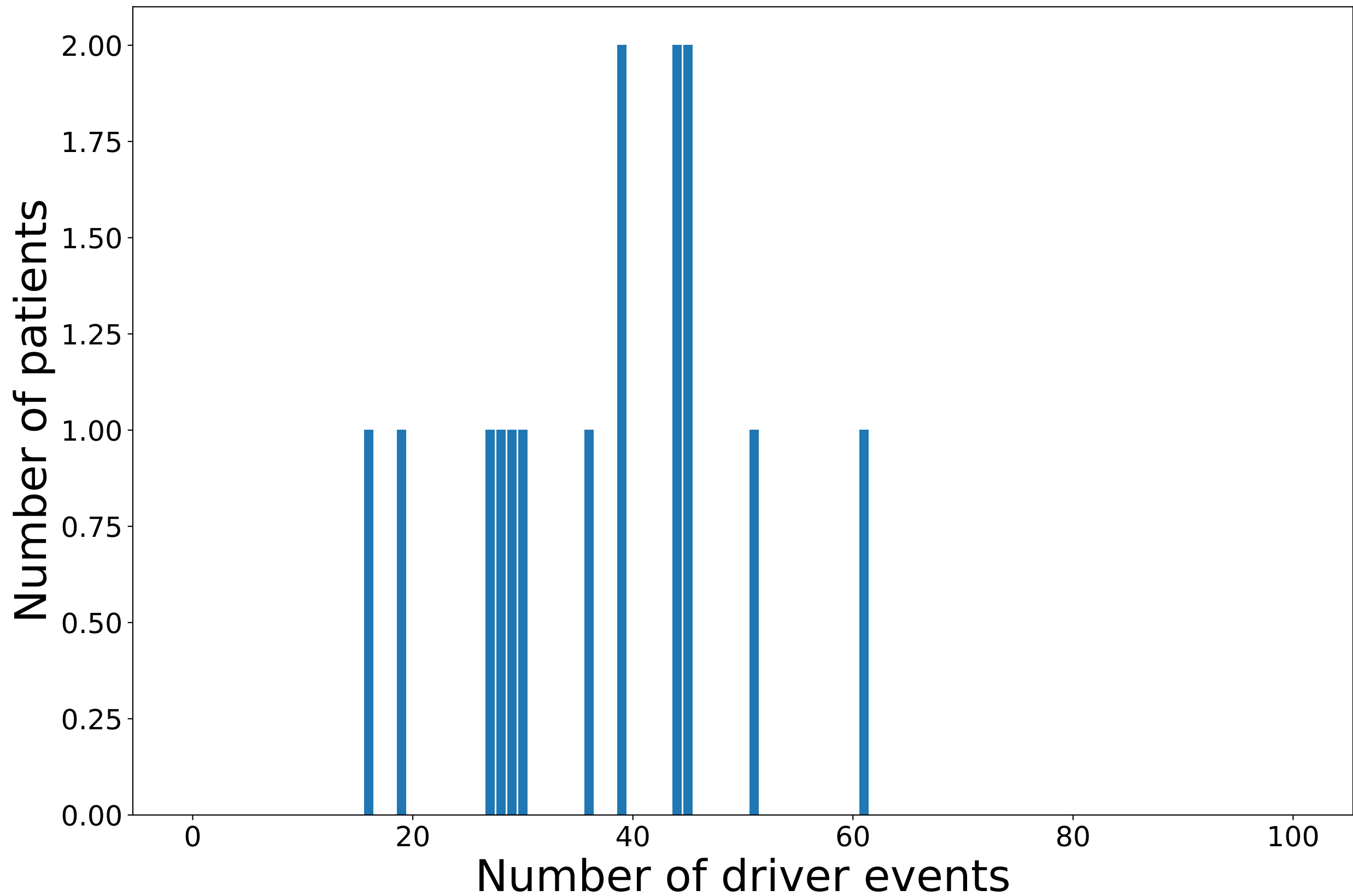

Supplement: S4 Files — (ZIP) [file pgen.1009996.s004.zip › Aneuploidy/PANCAN GISTIC2/patient distributions/2021_11_23_15_3_ESCA_FEMALE.pdf]

# CESC

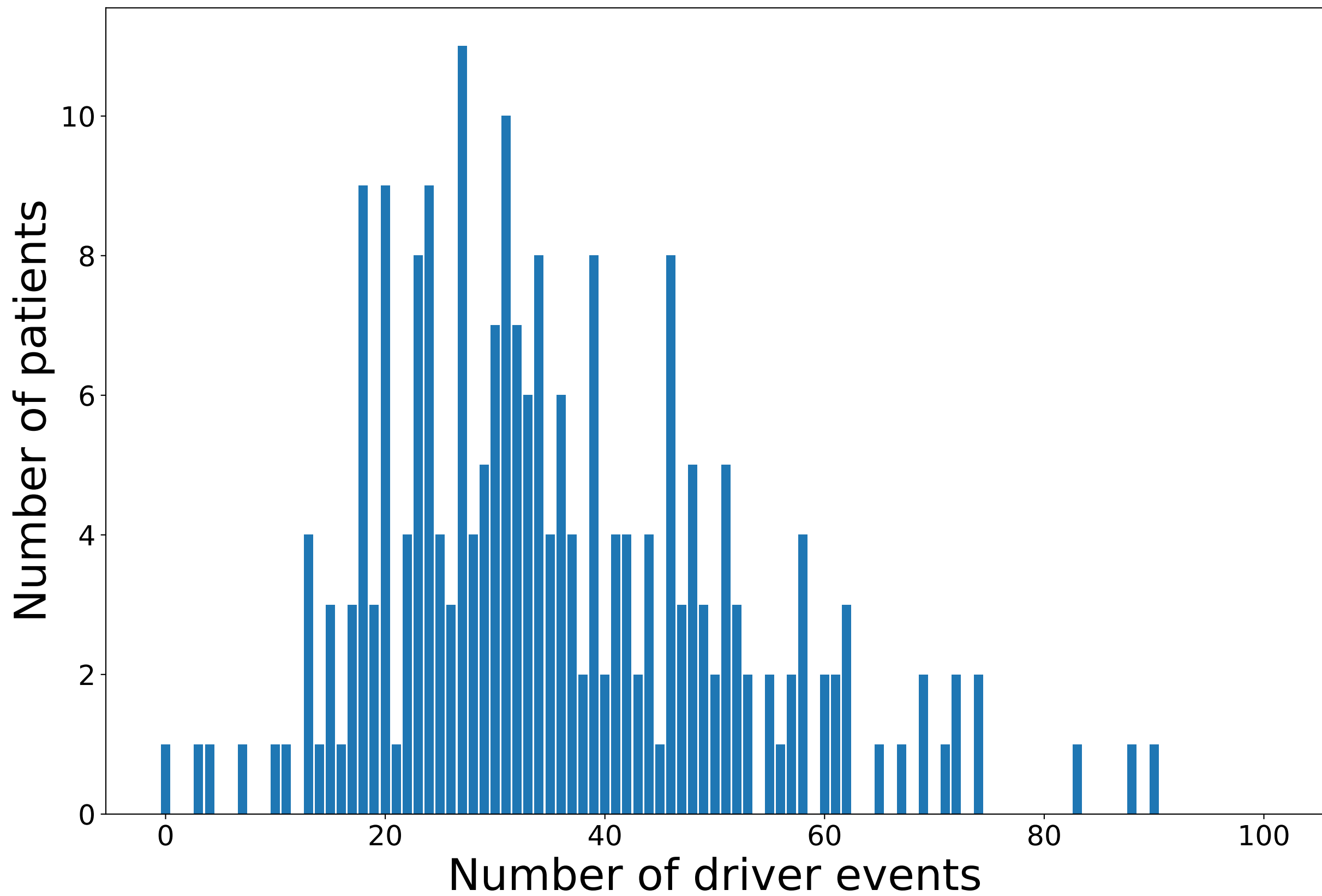

Supplement: S4 Files — (ZIP) [file pgen.1009996.s004.zip › Aneuploidy/PANCAN GISTIC2/patient distributions/2021_11_23_15_3_CESC.pdf]

# LUSC\_FEMALE

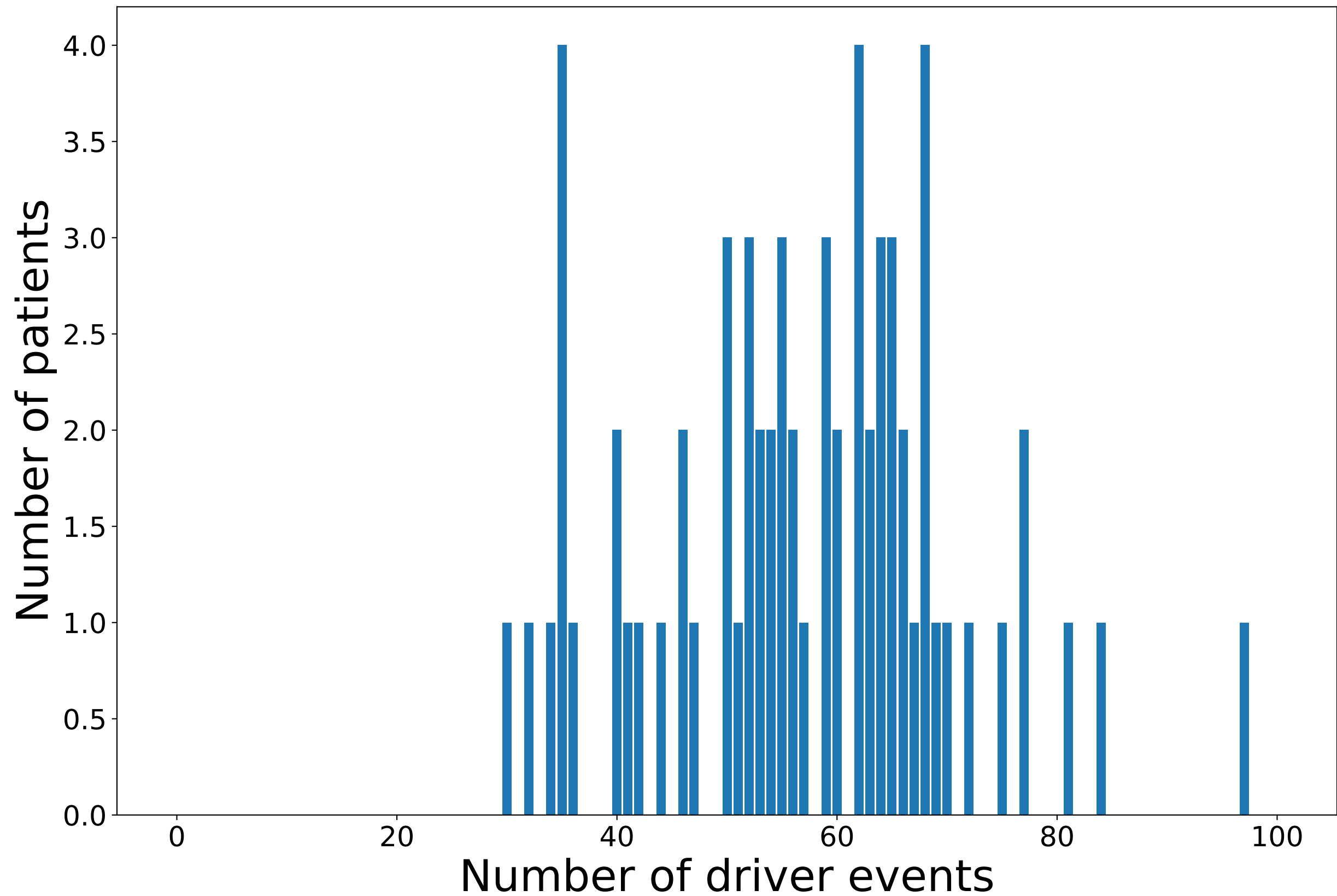

Supplement: S4 Files — (ZIP) [file pgen.1009996.s004.zip › Aneuploidy/PANCAN GISTIC2/patient distributions/2021_11_23_15_3_LUSC_FEMALE.pdf]

# UCEC\_FEMALE

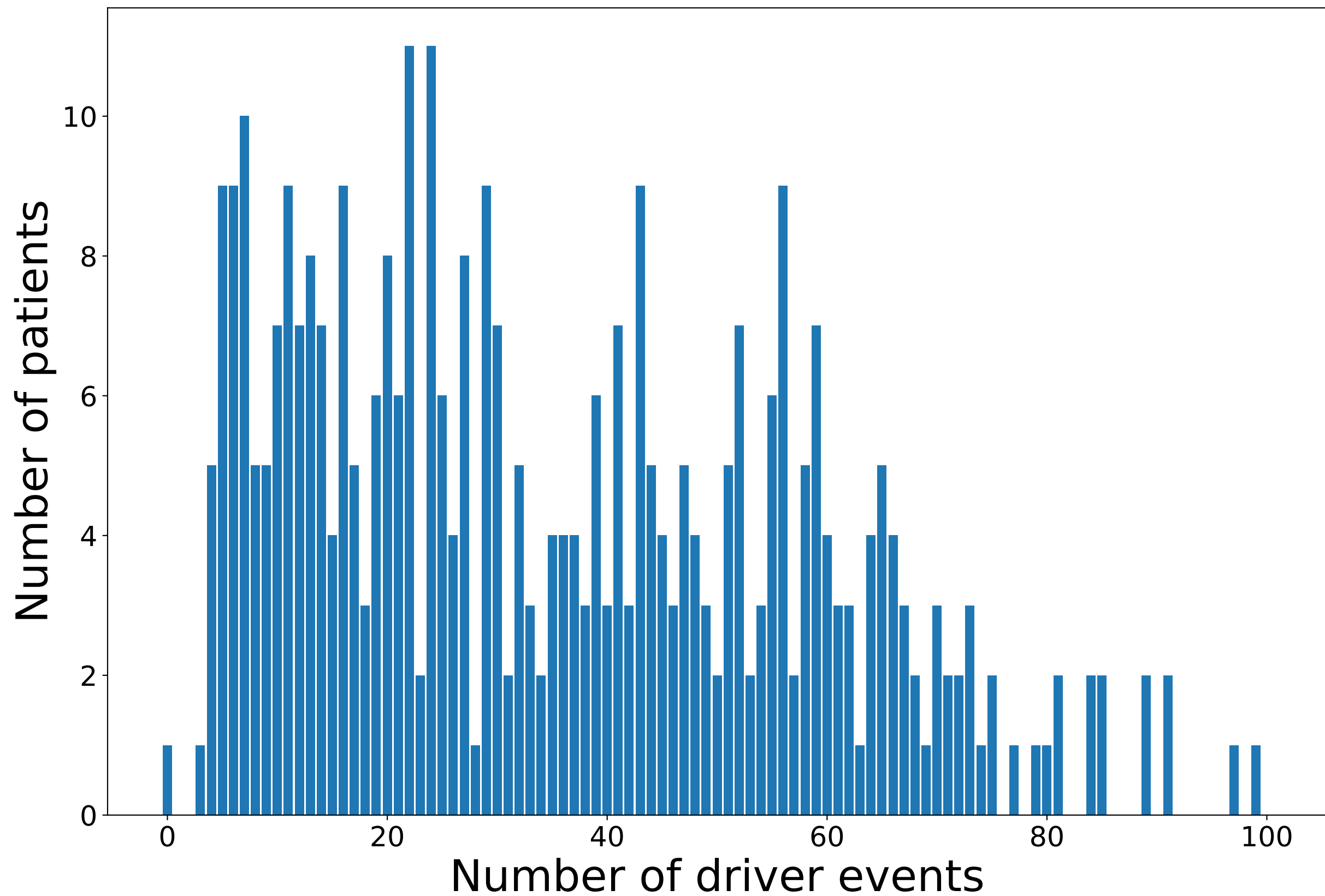

Supplement: S4 Files — (ZIP) [file pgen.1009996.s004.zip › Aneuploidy/PANCAN GISTIC2/patient distributions/2021_11_23_15_3_UCEC_FEMALE.pdf]

OV

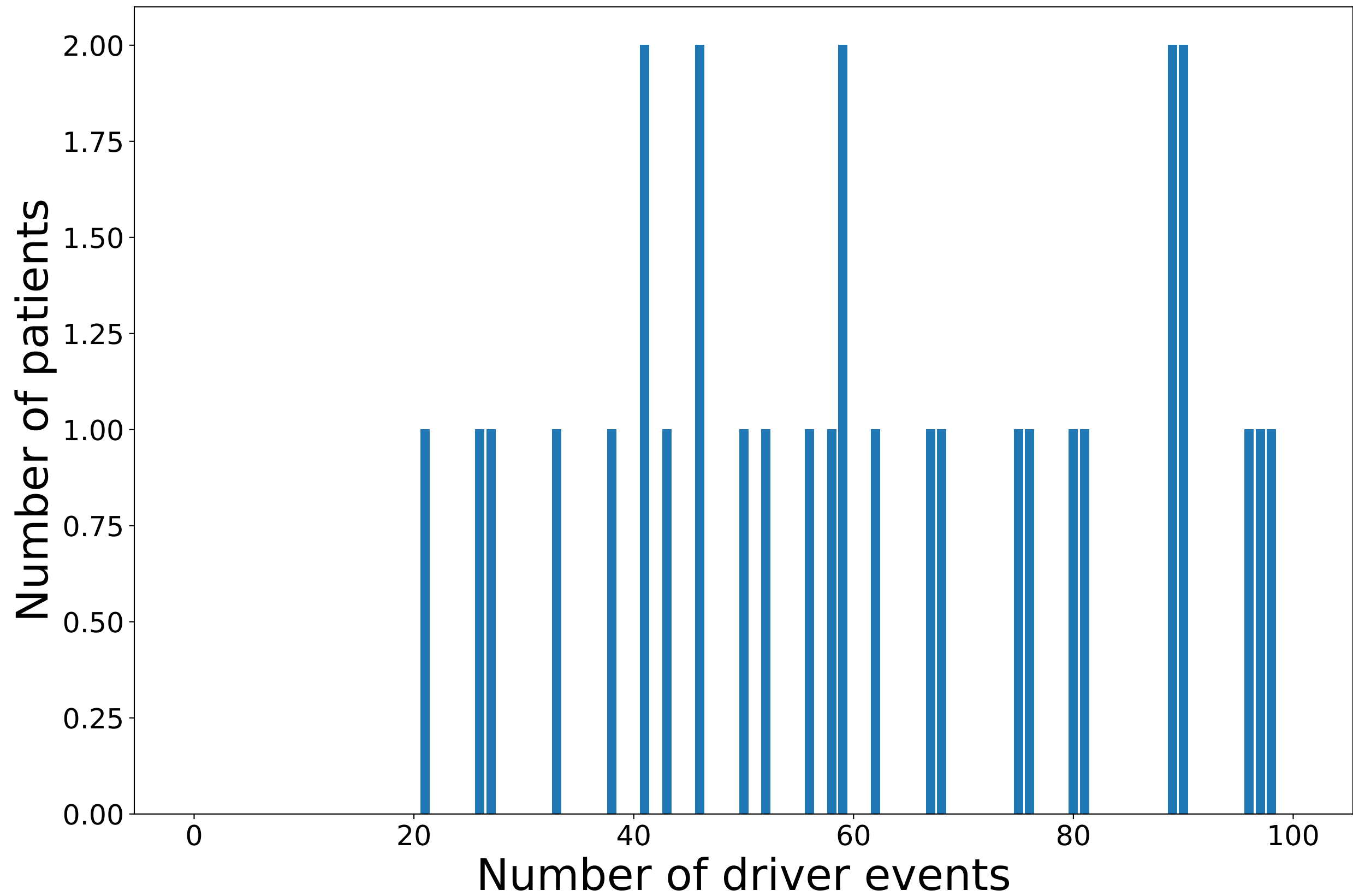

Supplement: S4 Files — (ZIP) [file pgen.1009996.s004.zip › Aneuploidy/PANCAN GISTIC2/patient distributions/2021_11_23_15_3_OV.pdf]

# LGG\_FEMALE

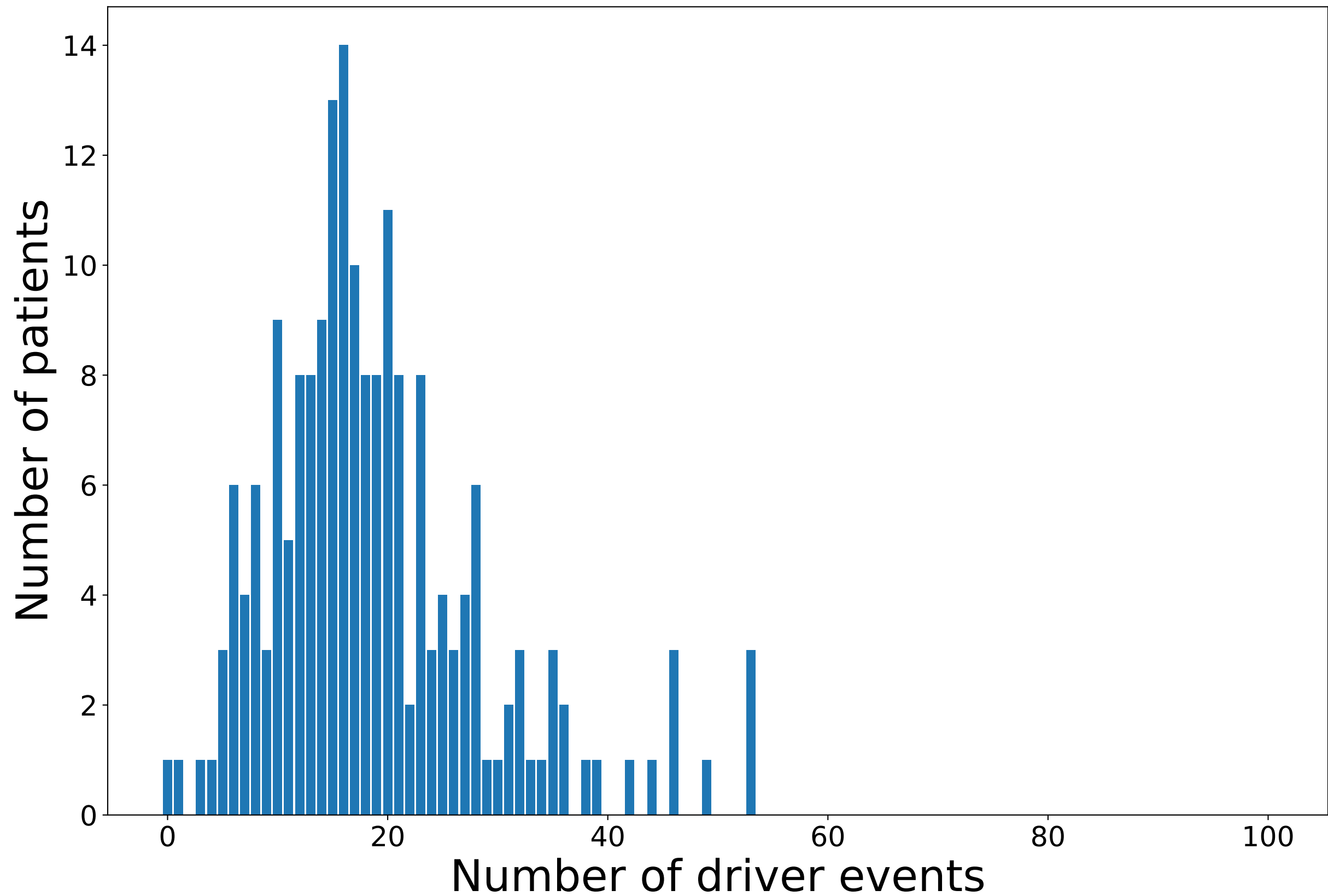

Supplement: S4 Files — (ZIP) [file pgen.1009996.s004.zip › Aneuploidy/PANCAN GISTIC2/patient distributions/2021_11_23_15_3_LGG_FEMALE.pdf]

# CESC\_FEMALE

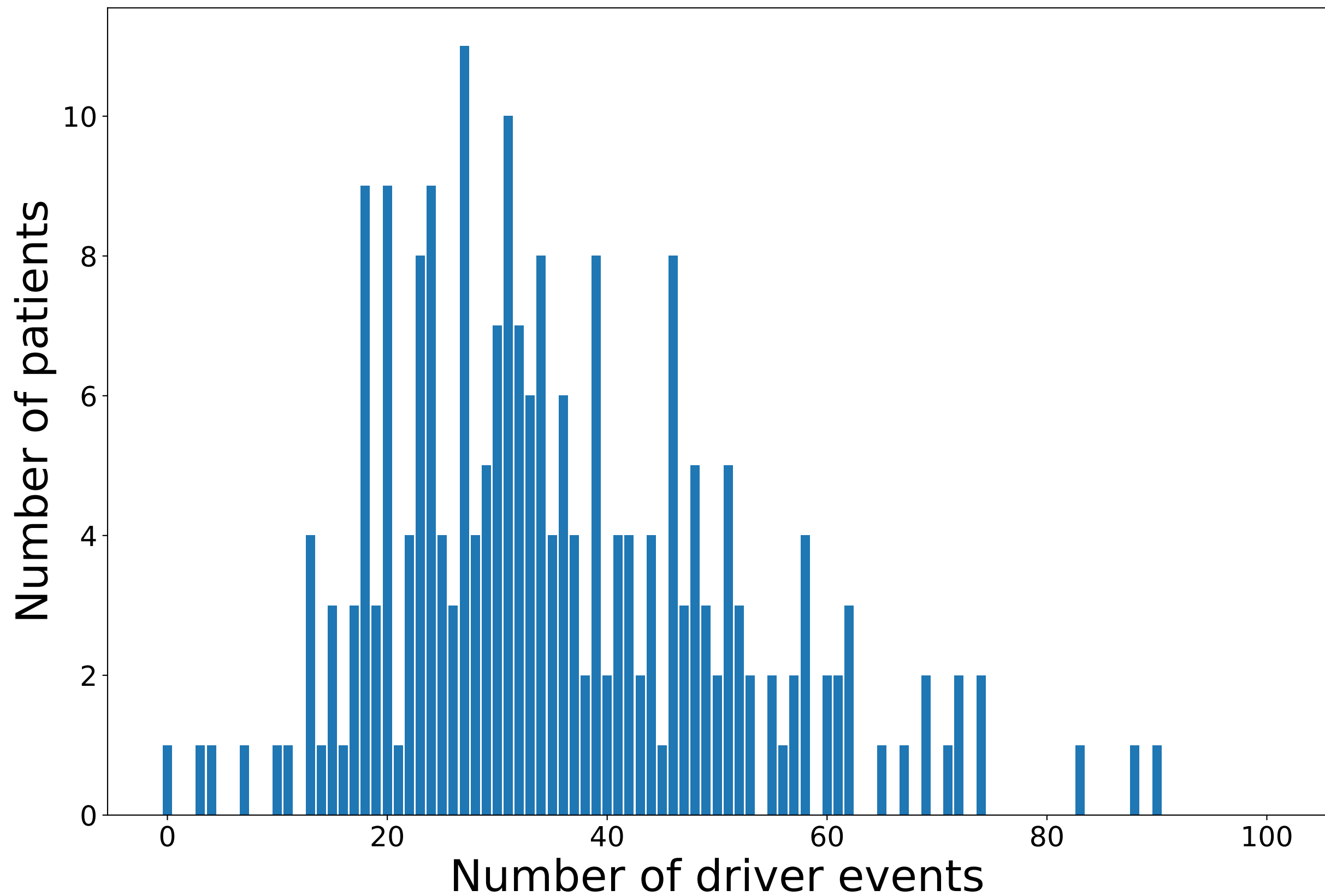

Supplement: S4 Files — (ZIP) [file pgen.1009996.s004.zip › Aneuploidy/PANCAN GISTIC2/patient distributions/2021_11_23_15_3_CESC_FEMALE.pdf]

# LGG

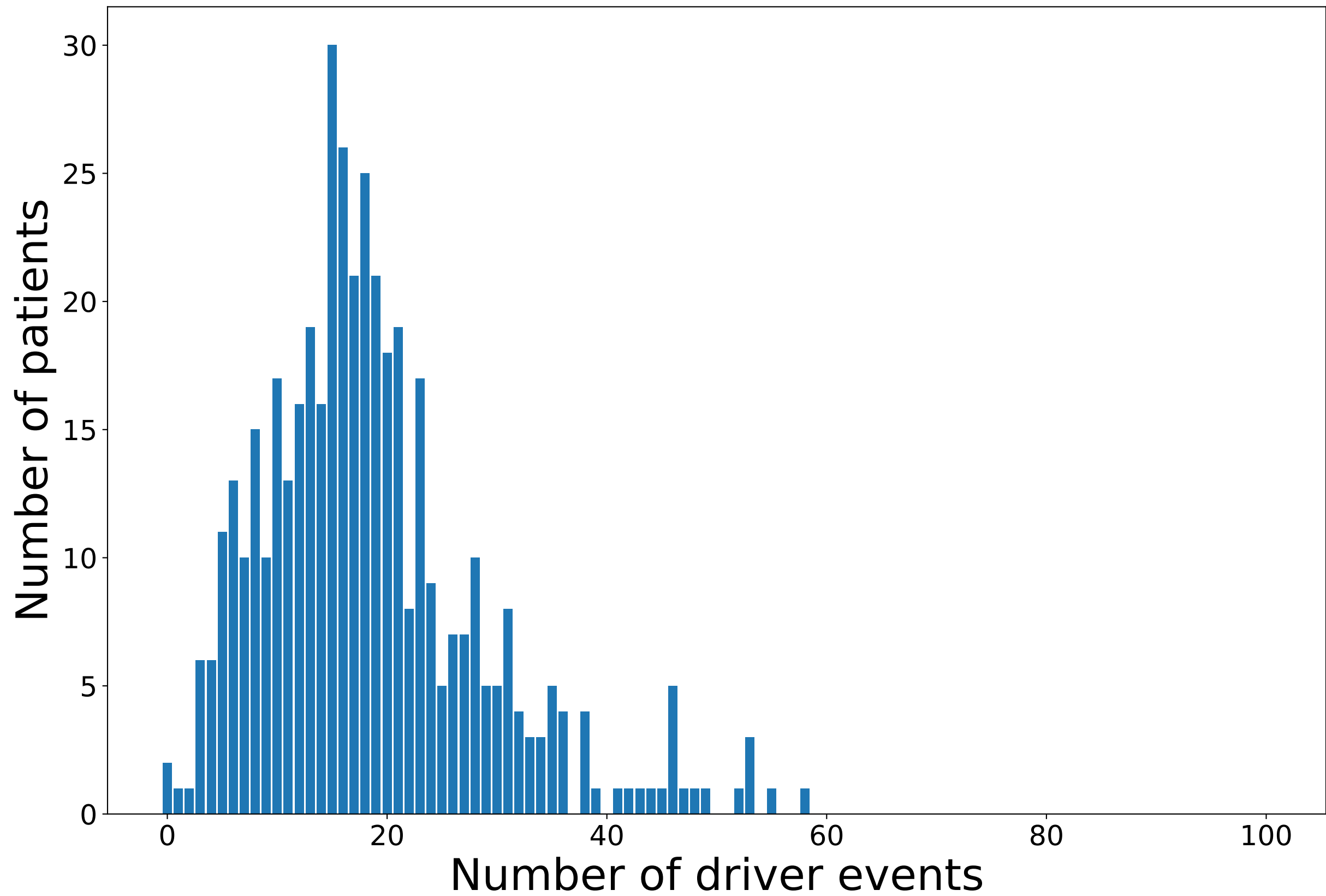

Supplement: S4 Files — (ZIP) [file pgen.1009996.s004.zip › Aneuploidy/PANCAN GISTIC2/patient distributions/2021_11_23_15_3_LGG.pdf]

# TGCT

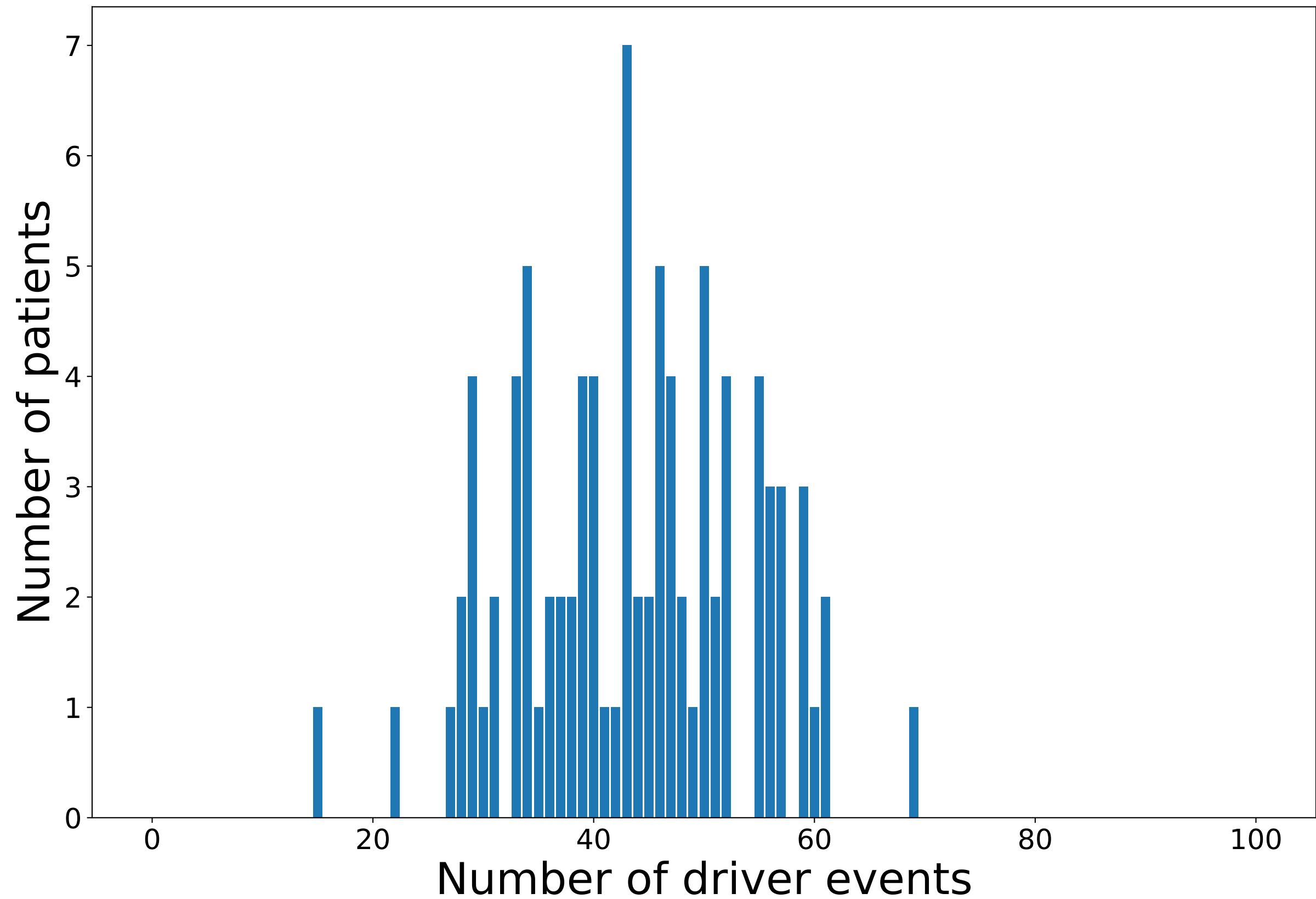

Supplement: S4 Files — (ZIP) [file pgen.1009996.s004.zip › Aneuploidy/PANCAN GISTIC2/patient distributions/2021_11_23_15_3_TGCT.pdf]

# PCPG\_FEMALE

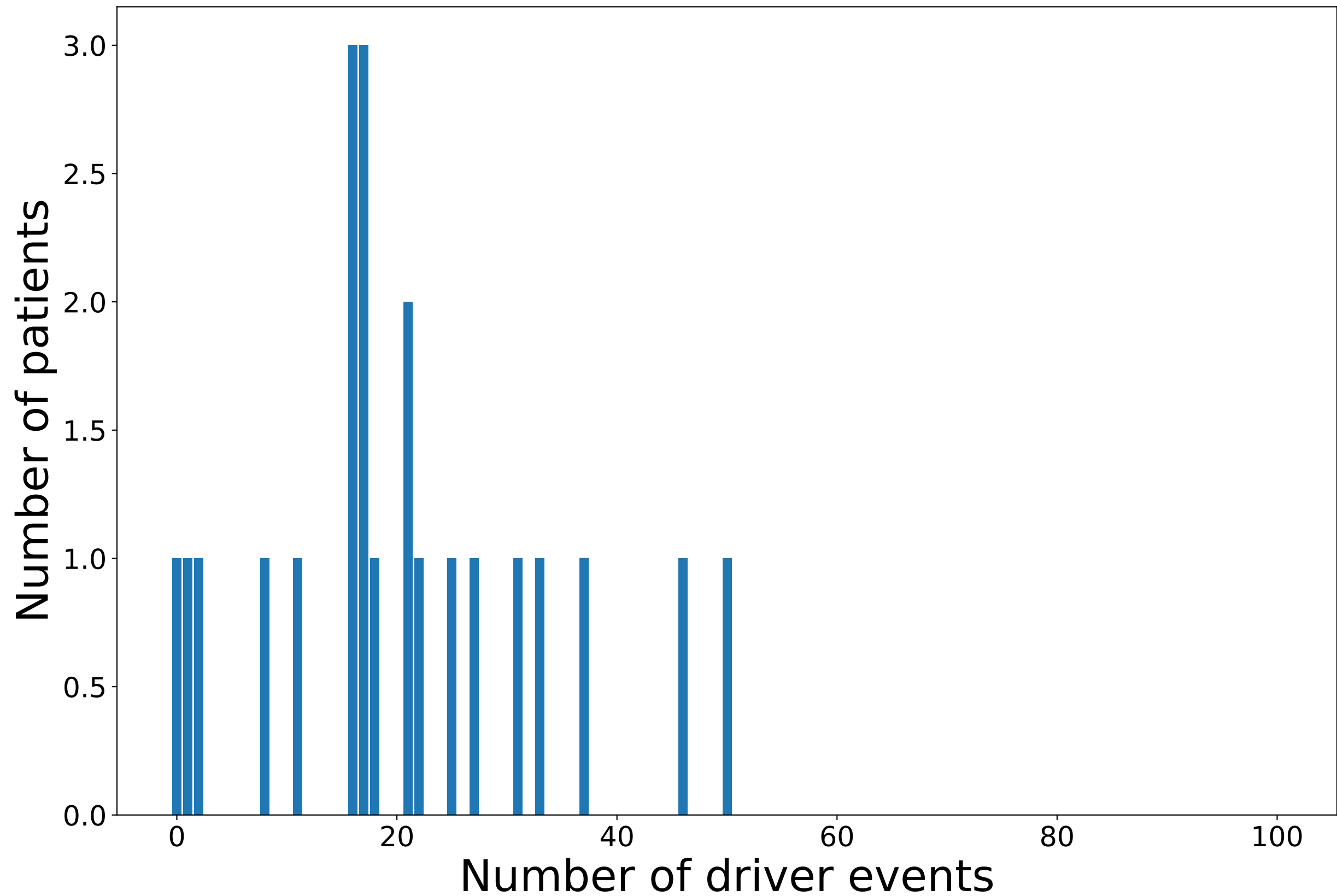

Supplement: S4 Files — (ZIP) [file pgen.1009996.s004.zip › Aneuploidy/PANCAN GISTIC2/patient distributions/2021_11_23_15_3_PCPG_FEMALE.pdf]

# GBM\_FEMALE

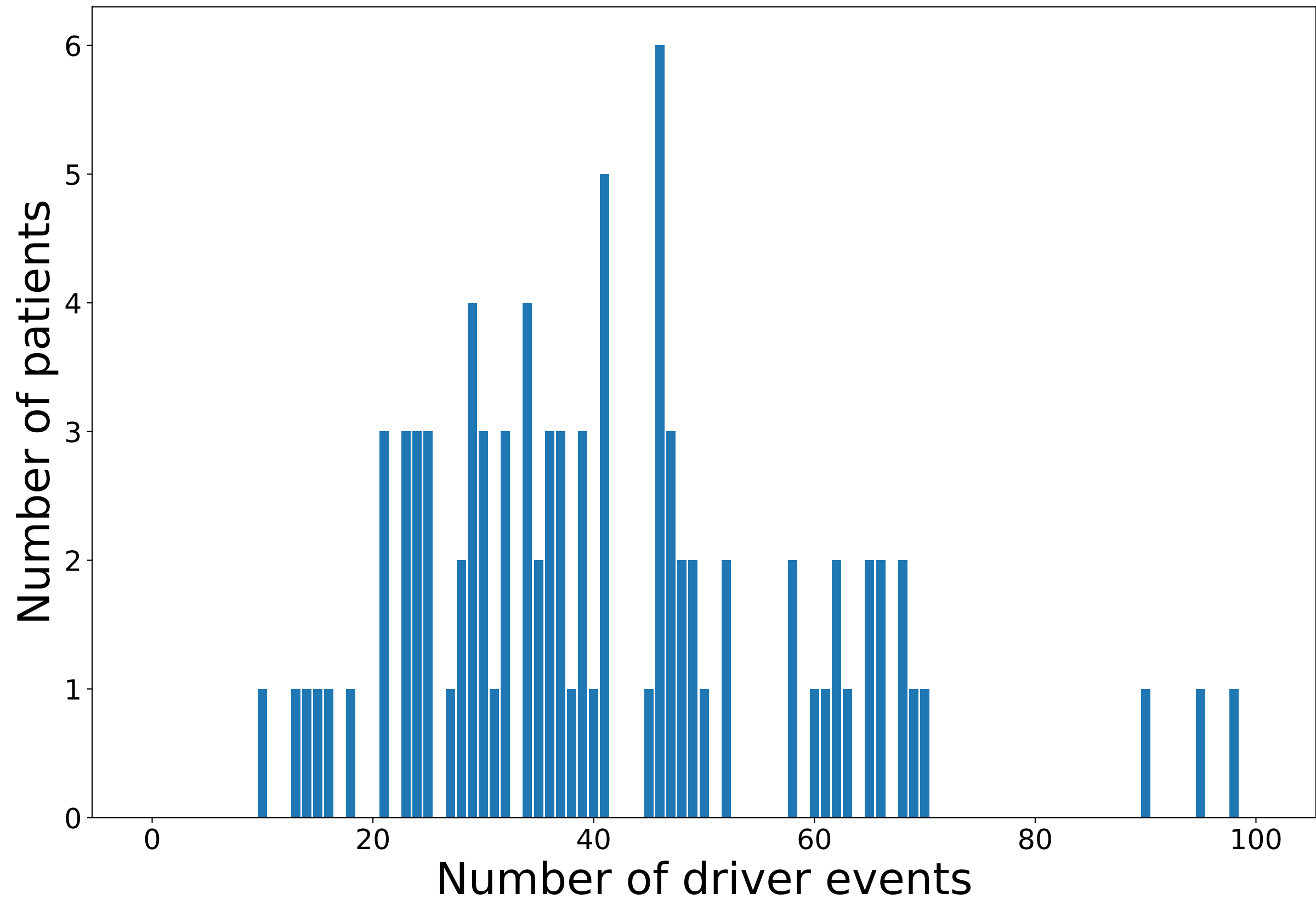

Supplement: S4 Files — (ZIP) [file pgen.1009996.s004.zip › Aneuploidy/PANCAN GISTIC2/patient distributions/2021_11_23_15_3_GBM_FEMALE.pdf]

# ACC

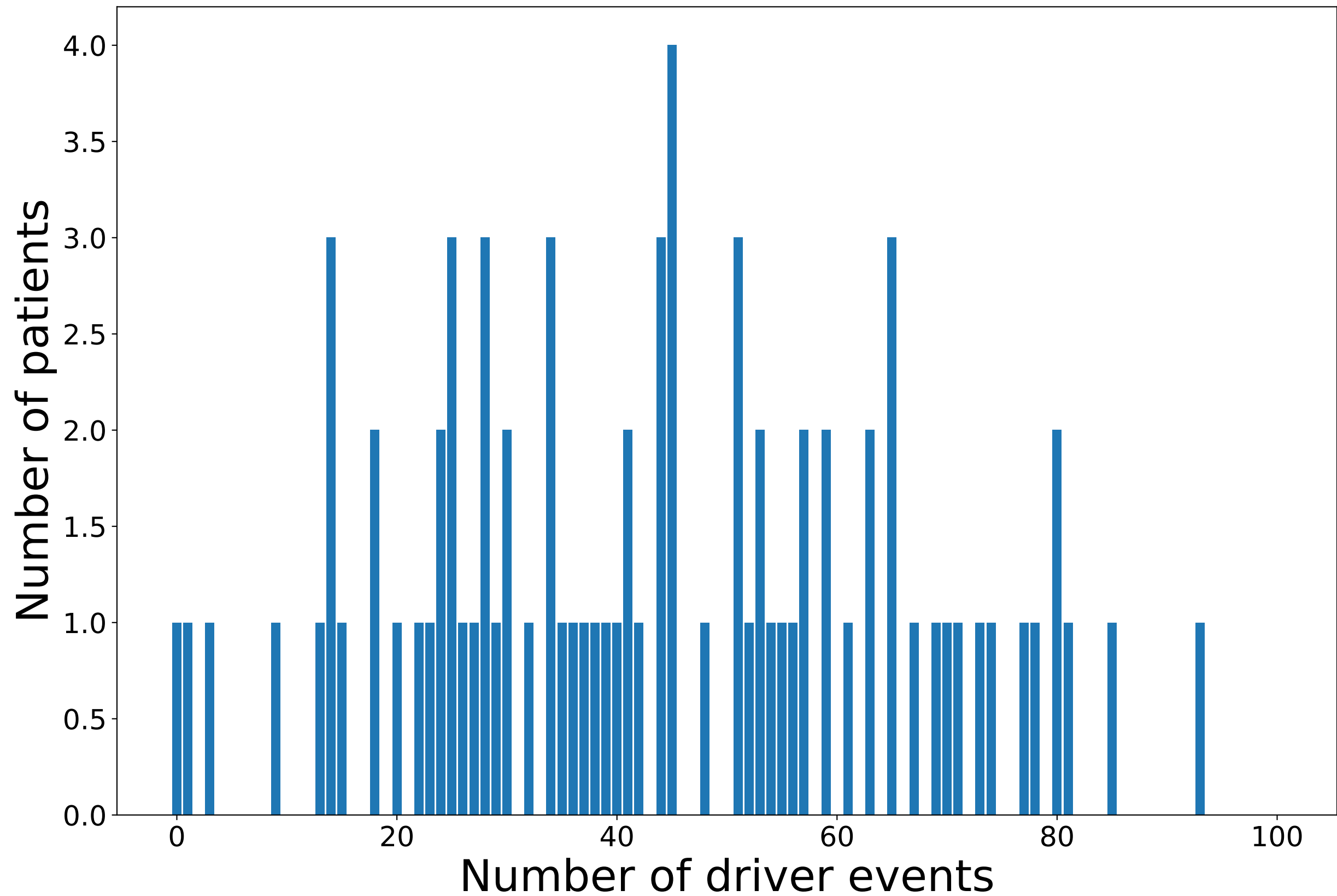

Supplement: S4 Files — (ZIP) [file pgen.1009996.s004.zip › Aneuploidy/PANCAN GISTIC2/patient distributions/2021_11_23_15_3_ACC.pdf]

# KIRC\_MALE

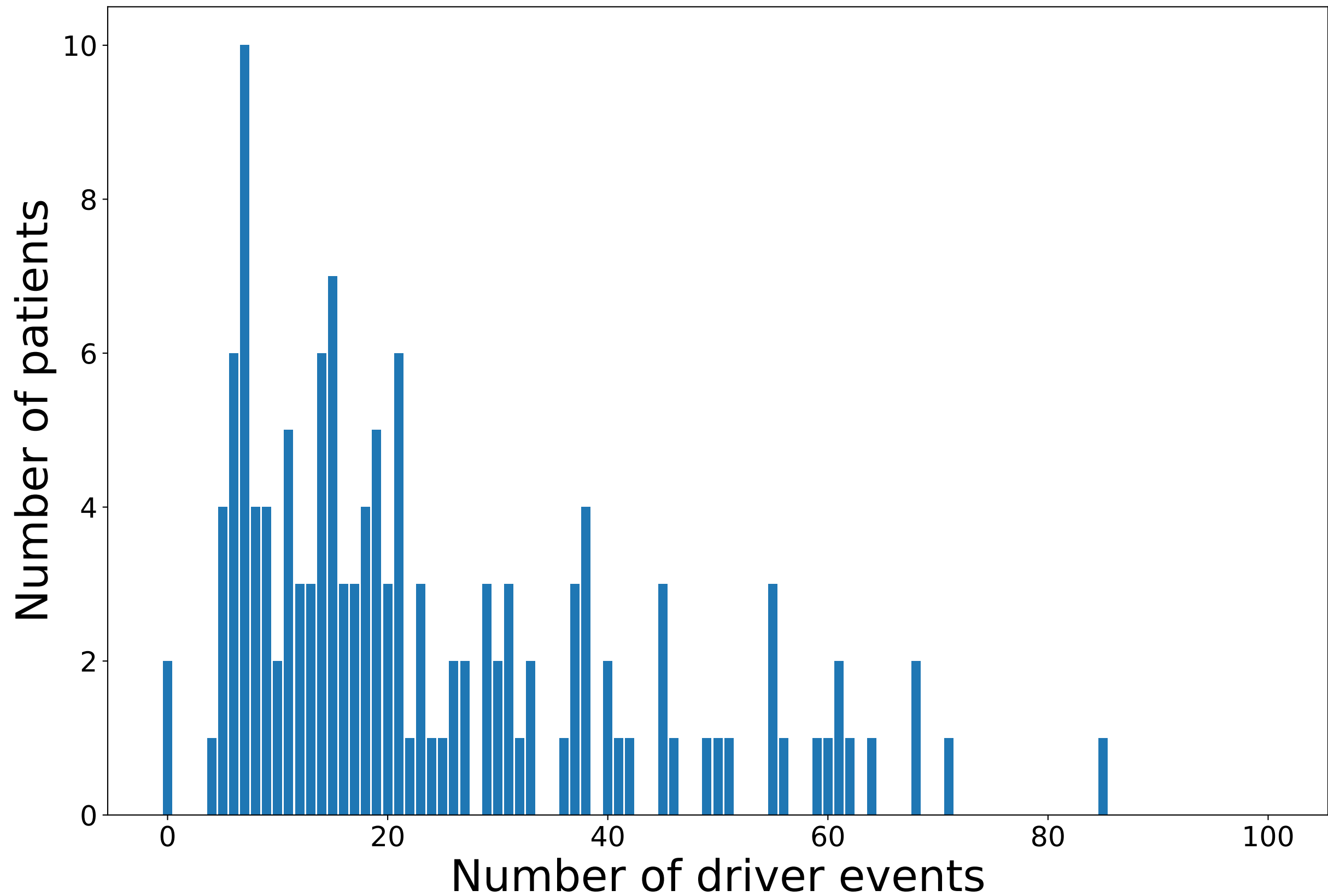

Supplement: S4 Files — (ZIP) [file pgen.1009996.s004.zip › Aneuploidy/PANCAN GISTIC2/patient distributions/2021_11_23_15_3_KIRC_MALE.pdf]

# PCPG

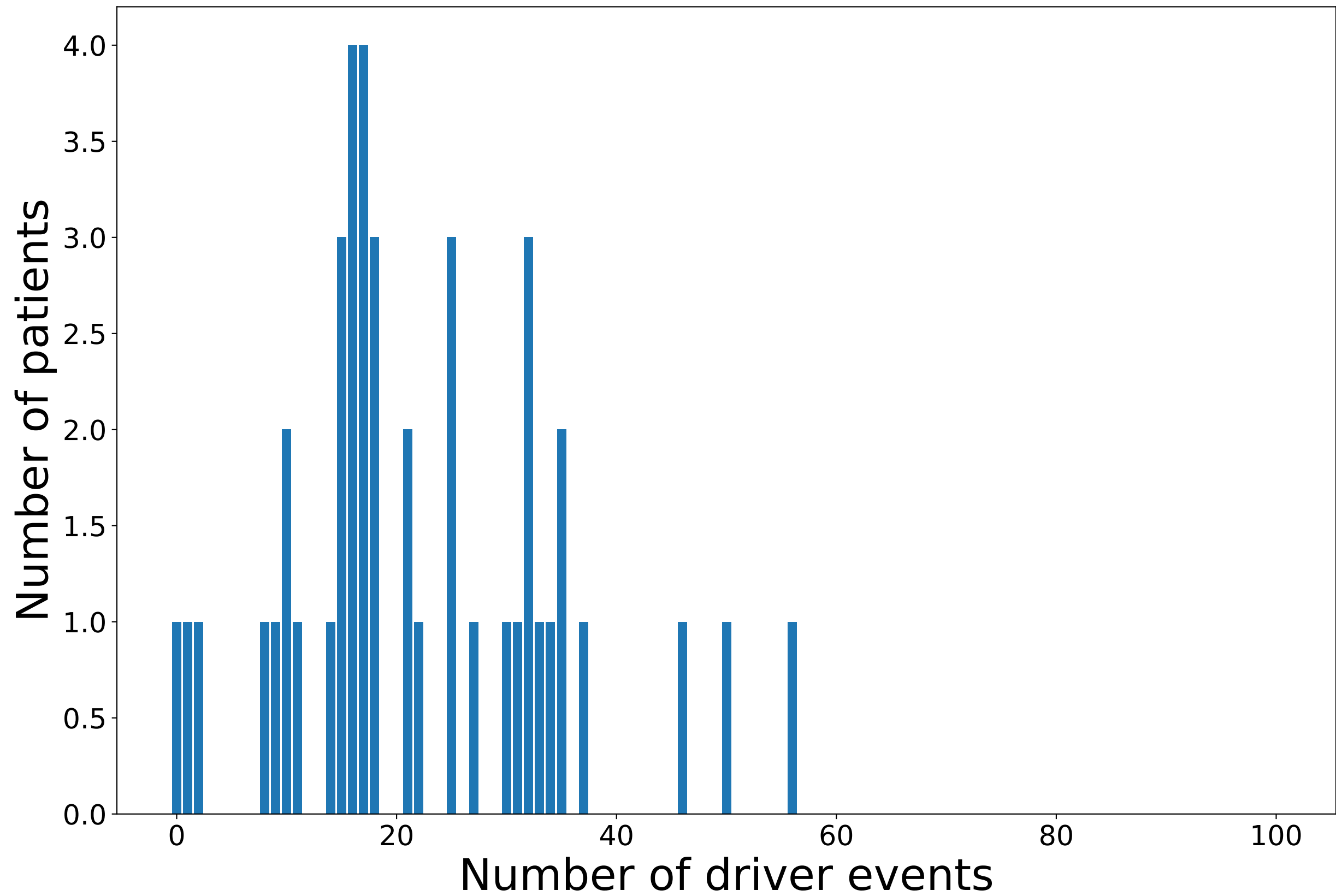

Supplement: S4 Files — (ZIP) [file pgen.1009996.s004.zip › Aneuploidy/PANCAN GISTIC2/patient distributions/2021_11_23_15_3_PCPG.pdf]

# BLCA\_MALE

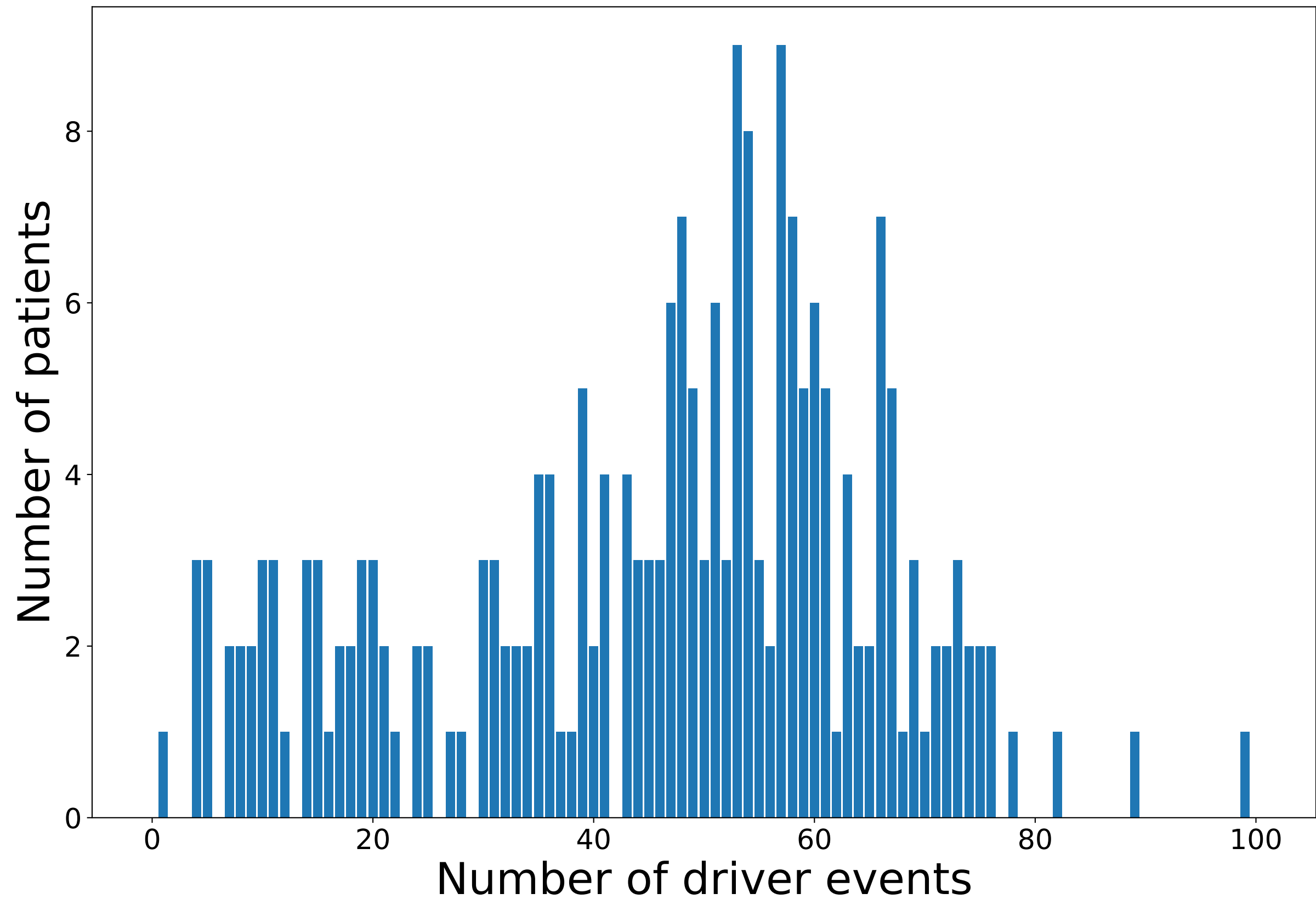

Supplement: S4 Files — (ZIP) [file pgen.1009996.s004.zip › Aneuploidy/PANCAN GISTIC2/patient distributions/2021_11_23_15_3_BLCA_MALE.pdf]

# LUSC

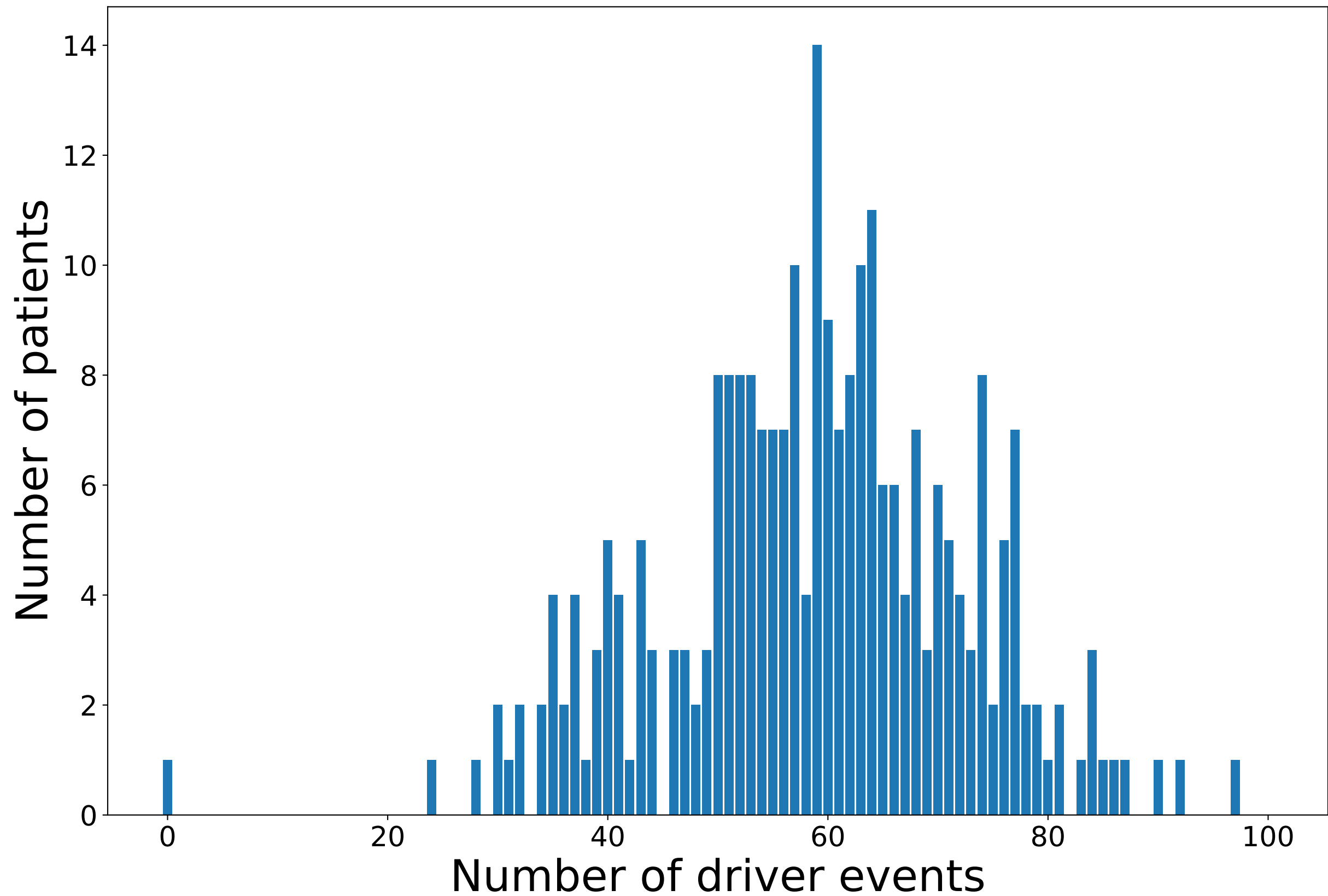

Supplement: S4 Files — (ZIP) [file pgen.1009996.s004.zip › Aneuploidy/PANCAN GISTIC2/patient distributions/2021_11_23_15_3_LUSC.pdf]

# CHOL\_MALE

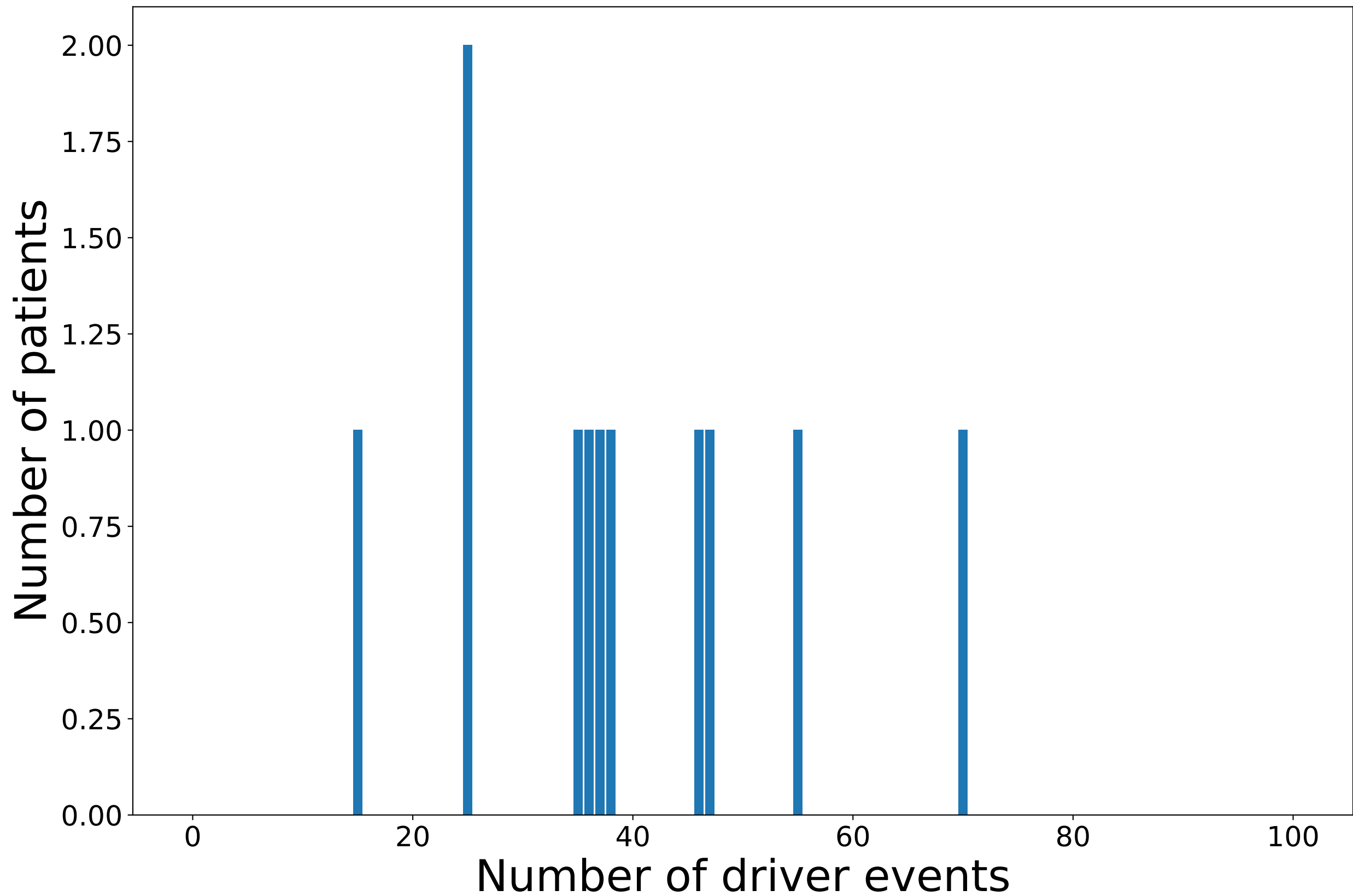

Supplement: S4 Files — (ZIP) [file pgen.1009996.s004.zip › Aneuploidy/PANCAN GISTIC2/patient distributions/2021_11_23_15_3_CHOL_MALE.pdf]

# COAD\_MALE

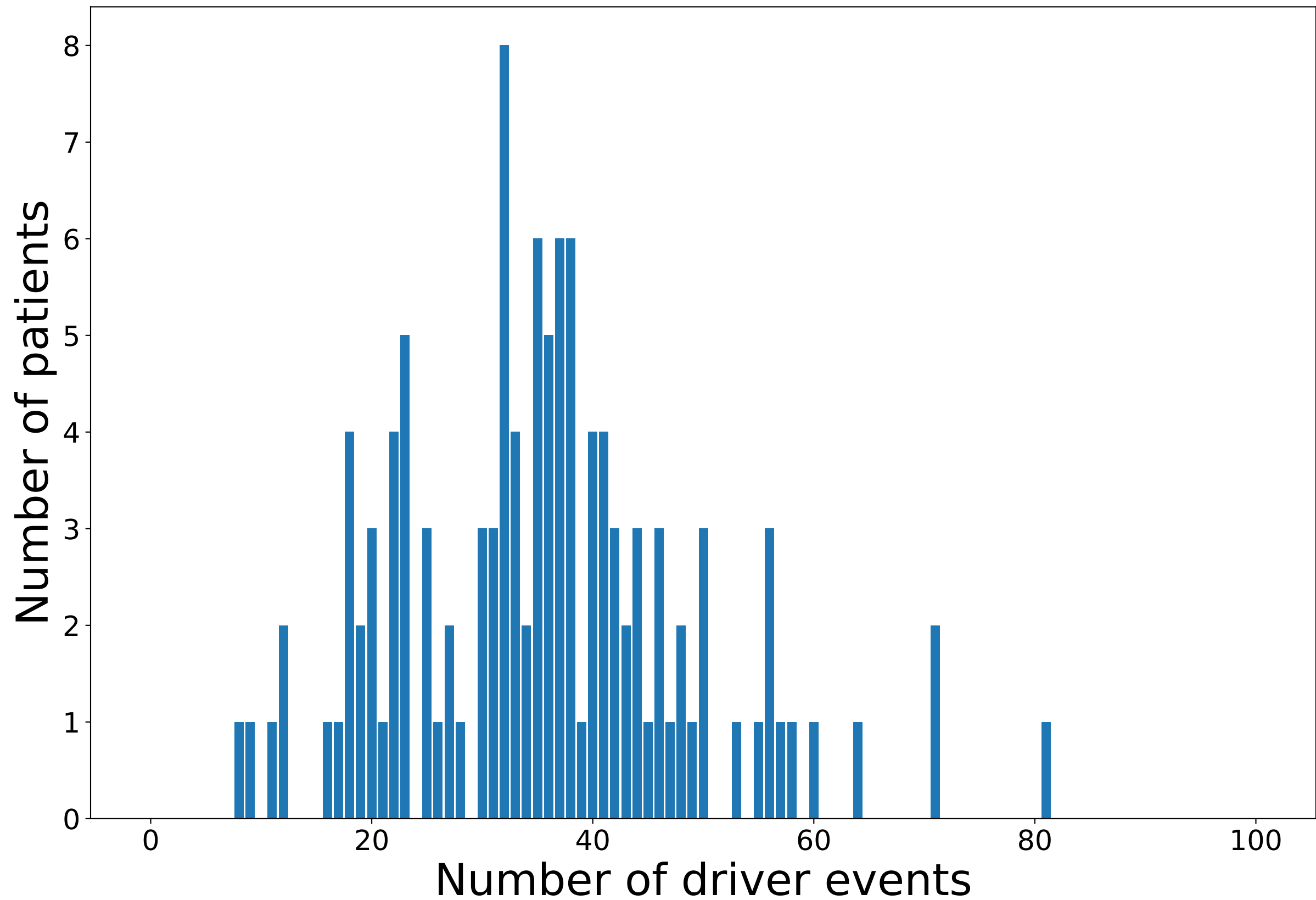

Supplement: S4 Files — (ZIP) [file pgen.1009996.s004.zip › Aneuploidy/PANCAN GISTIC2/patient distributions/2021_11_23_15_3_COAD_MALE.pdf]

# PANCAN

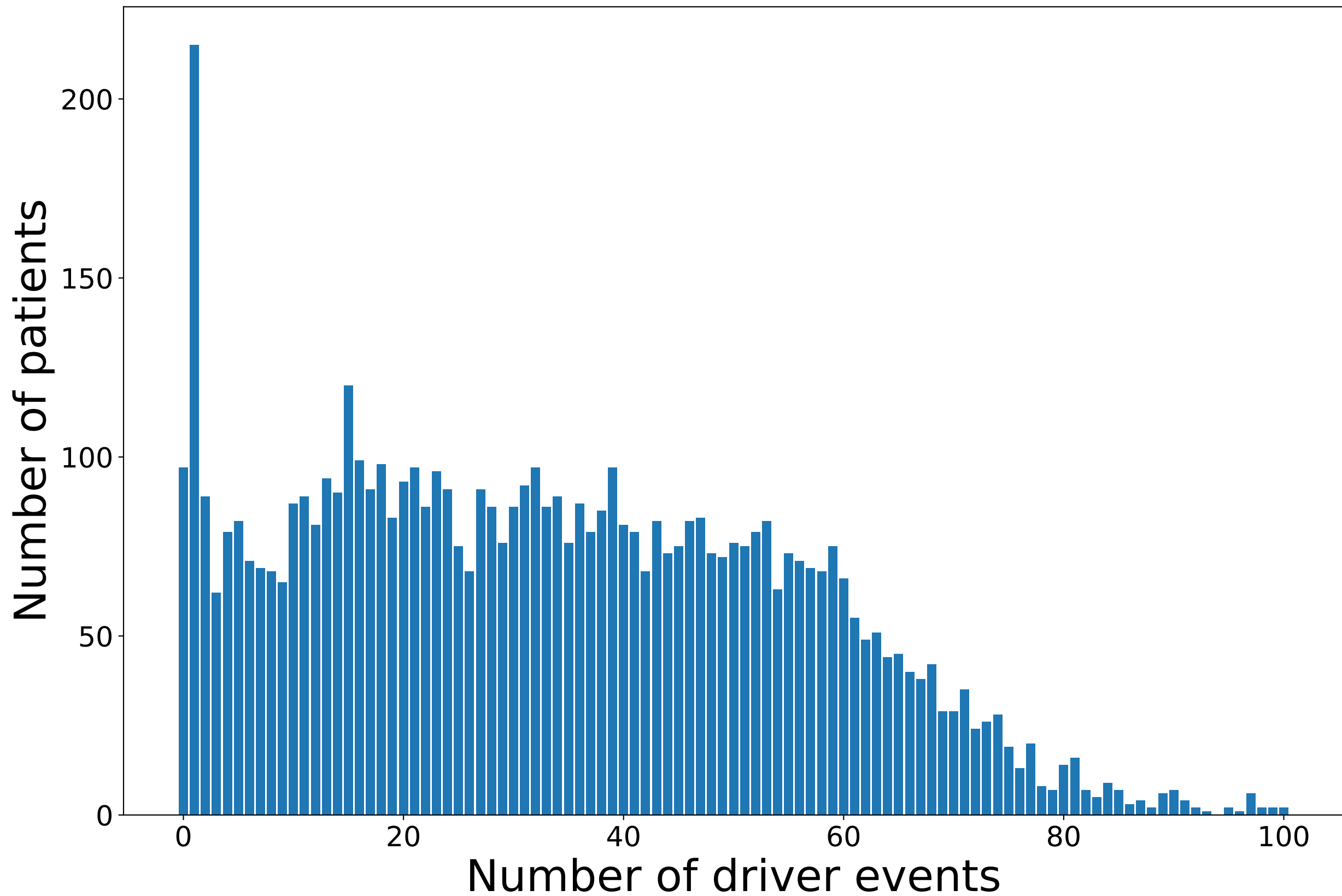

Supplement: S4 Files — (ZIP) [file pgen.1009996.s004.zip › Aneuploidy/PANCAN GISTIC2/patient distributions/2021_11_23_15_3_PANCAN.pdf]

# THYM\_FEMALE

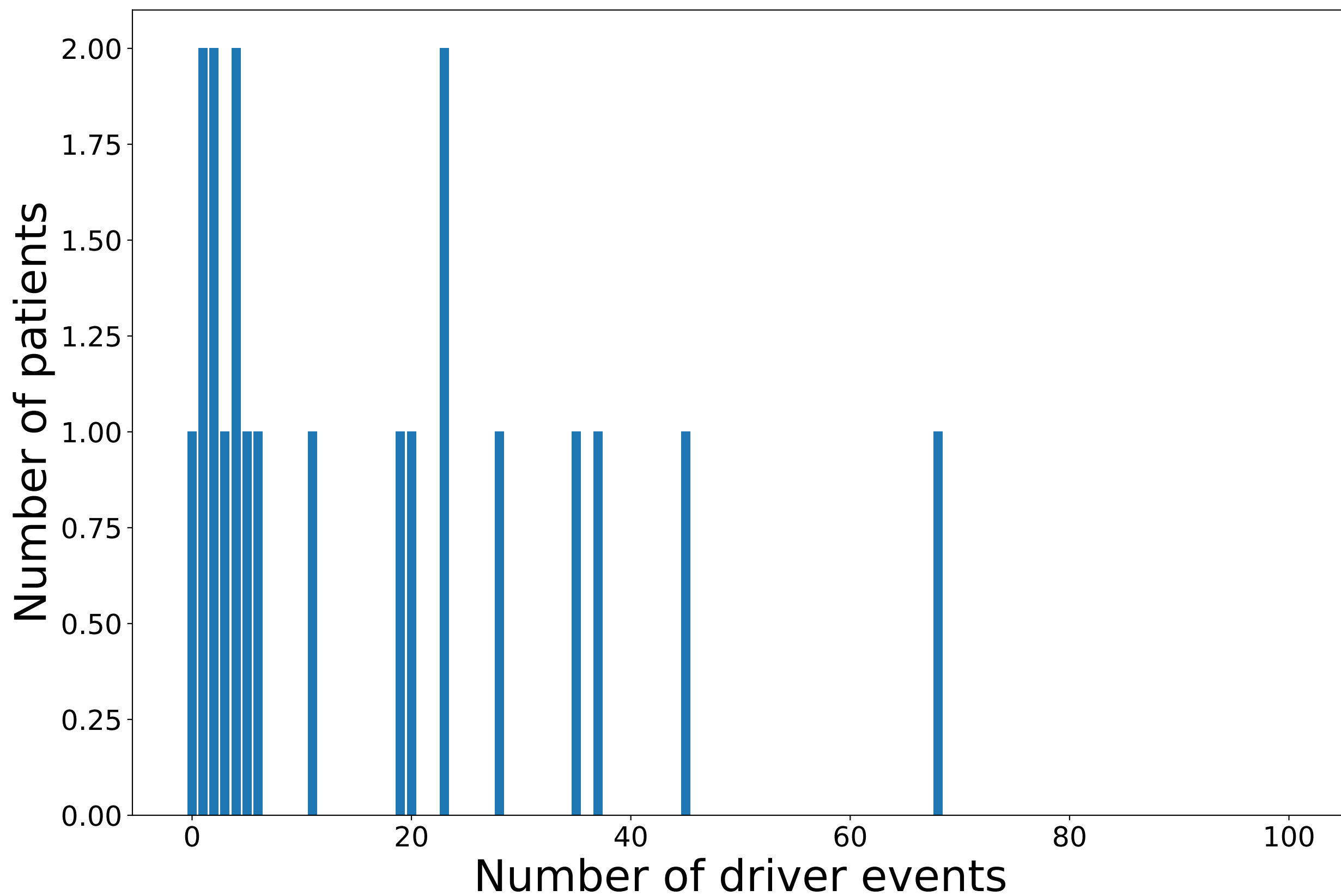

Supplement: S4 Files — (ZIP) [file pgen.1009996.s004.zip › Aneuploidy/PANCAN GISTIC2/patient distributions/2021_11_23_15_3_THYM_FEMALE.pdf]

Driver event distribution by total number of driver events per patient

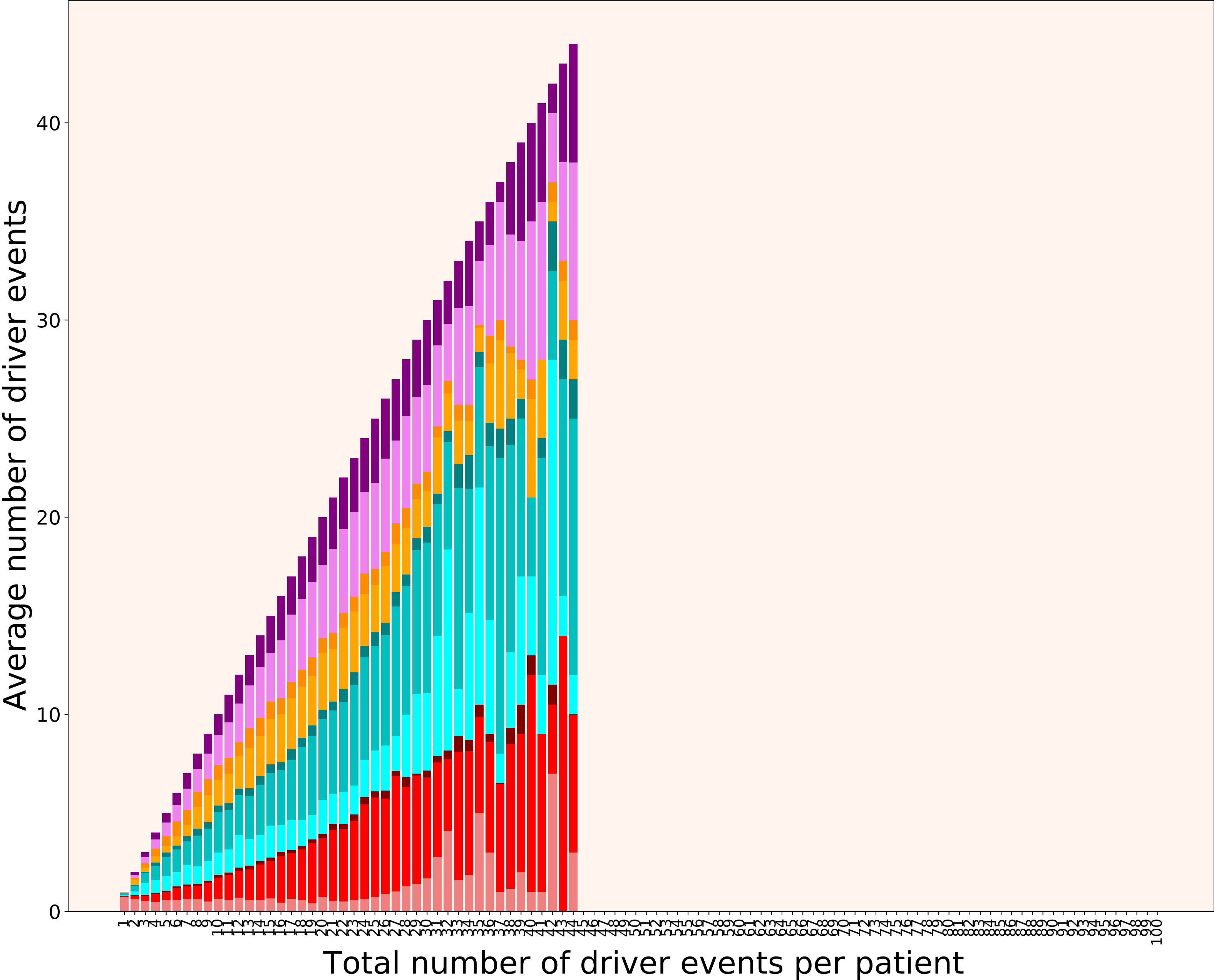

Supplement: S4 Files — (ZIP) [file pgen.1009996.s004.zip › Aneuploidy/COHORTS GISTIC2/cumulative histograms/2021_11_23_15_0_distribution_events_detailed.pdf]

Driver event distribution by age

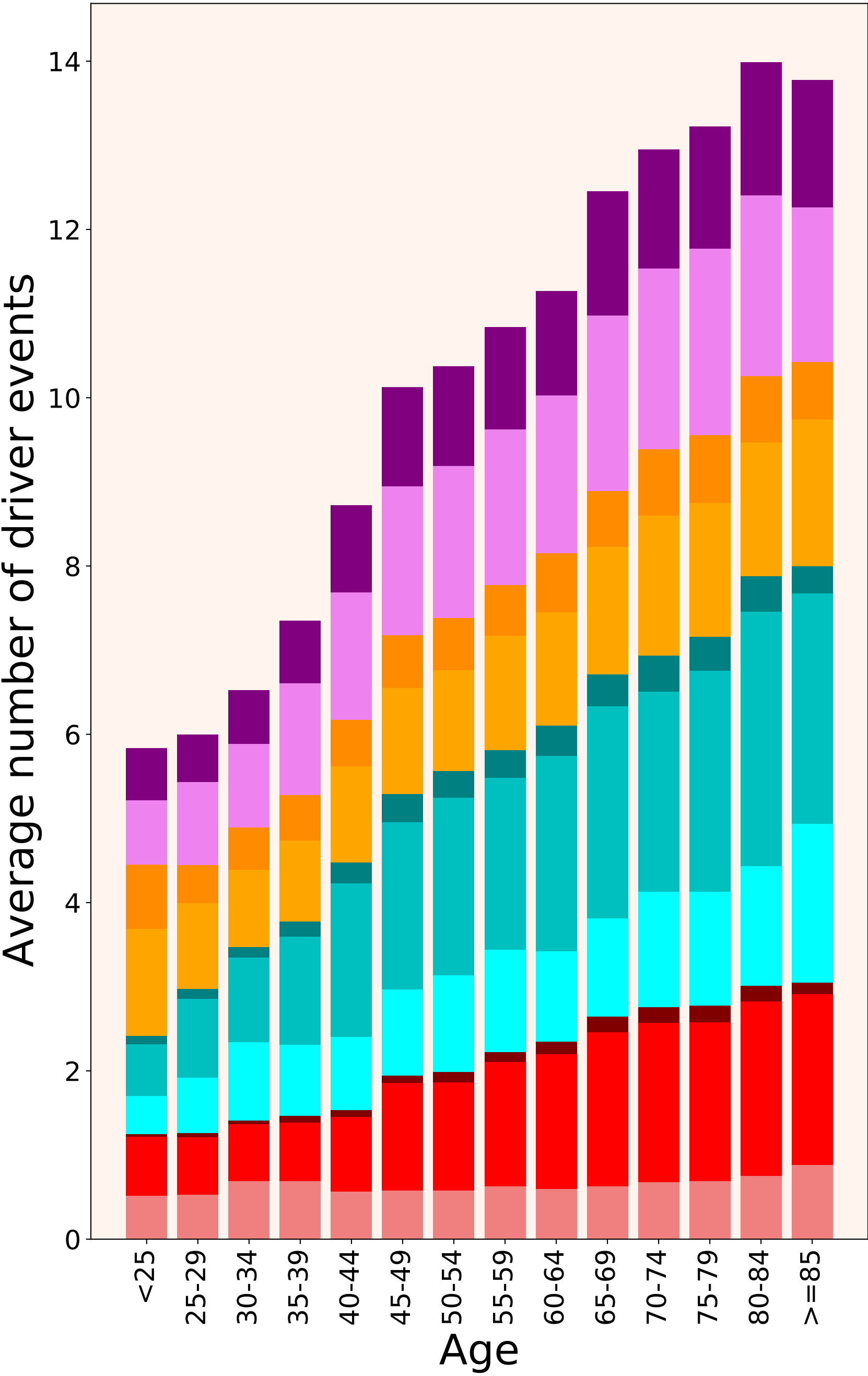

Supplement: S4 Files — (ZIP) [file pgen.1009996.s004.zip › Aneuploidy/COHORTS GISTIC2/cumulative histograms/2021_11_23_15_0_distribution_age.pdf]

Driver event distribution by total number of driver events per patient in females

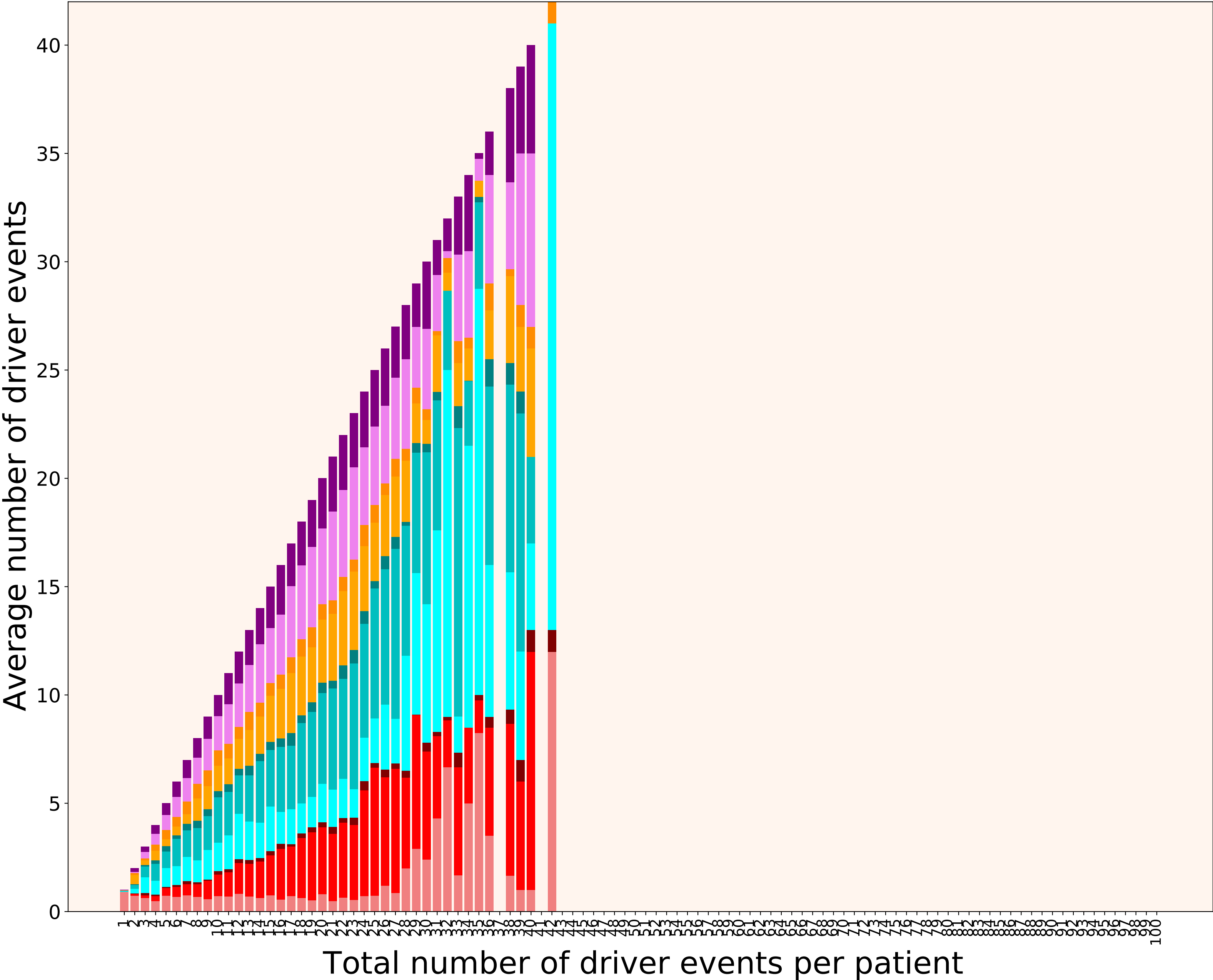

Supplement: S4 Files — (ZIP) [file pgen.1009996.s004.zip › Aneuploidy/COHORTS GISTIC2/cumulative histograms/2021_11_23_15_0_distribution_events_detailed_females.pdf]

Driver event distribution by cancer stage in females

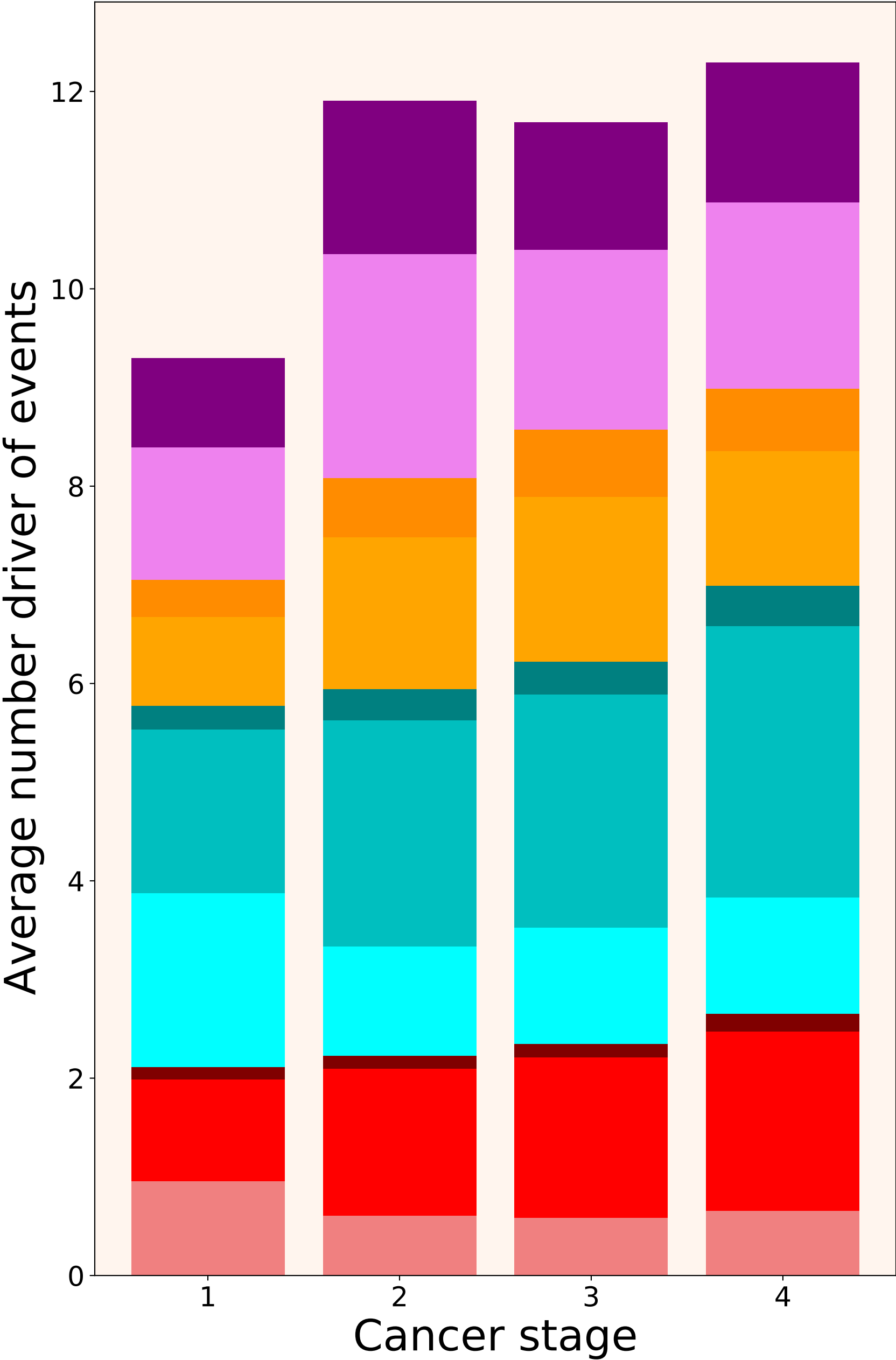

Supplement: S4 Files — (ZIP) [file pgen.1009996.s004.zip › Aneuploidy/COHORTS GISTIC2/cumulative histograms/2021_11_23_15_0_distribution_stages_females.pdf]

Driver event distribution by cancer type

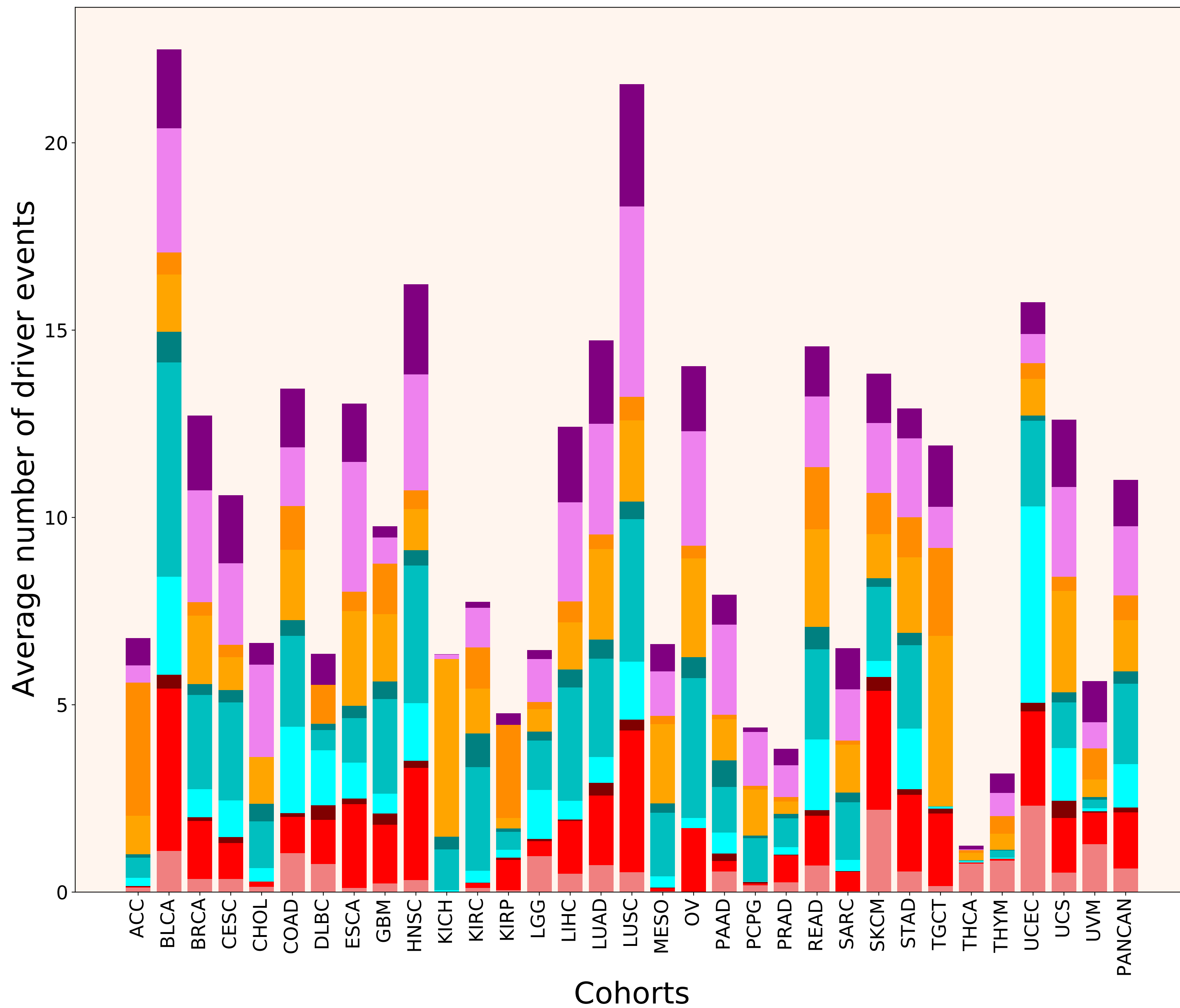

Supplement: S4 Files — (ZIP) [file pgen.1009996.s004.zip › Aneuploidy/COHORTS GISTIC2/cumulative histograms/2021_11_23_15_0_distribution_cohorts.pdf]

Driver event distribution by age in females

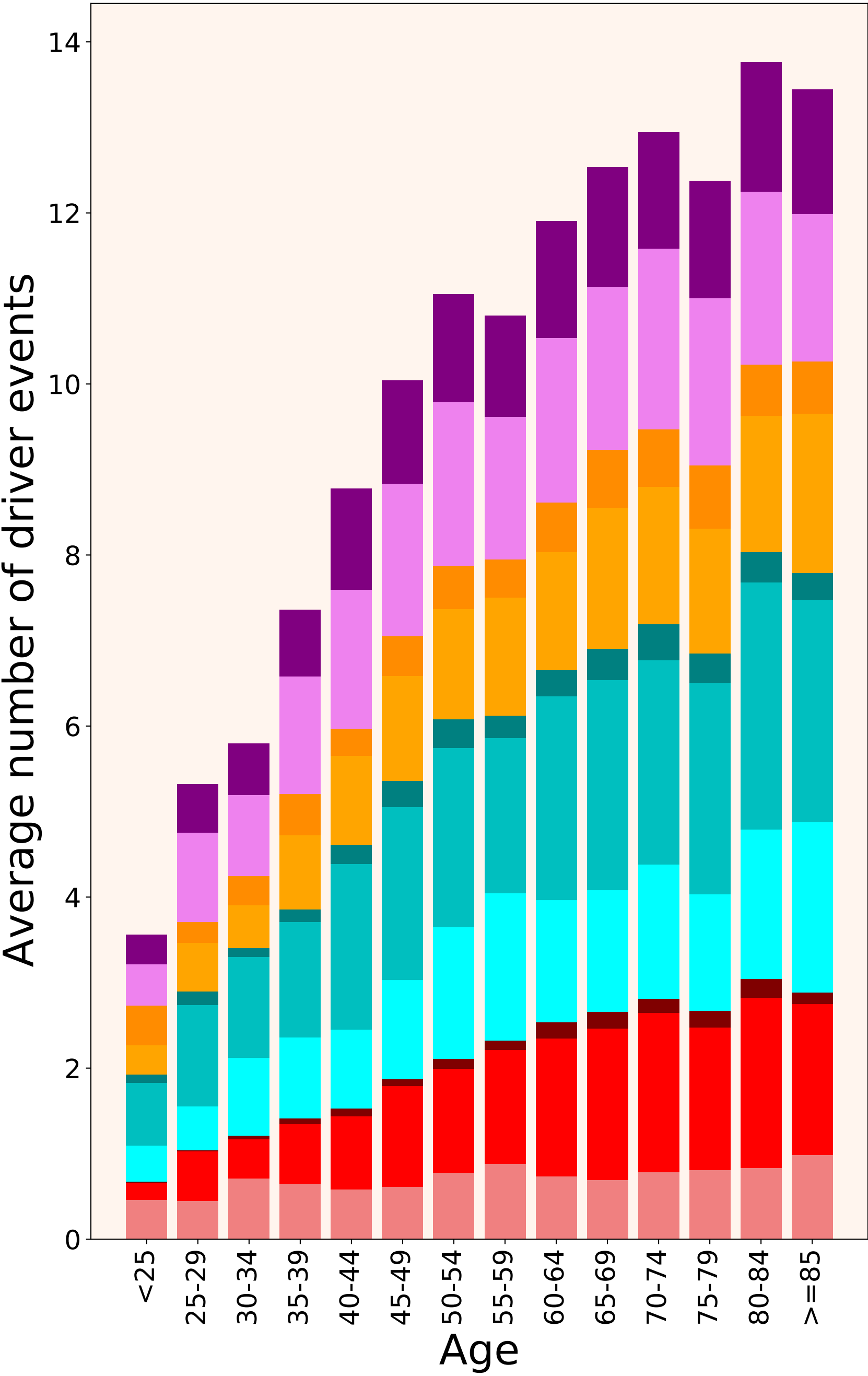

Supplement: S4 Files — (ZIP) [file pgen.1009996.s004.zip › Aneuploidy/COHORTS GISTIC2/cumulative histograms/2021_11_23_15_0_distribution_age_females.pdf]

Driver event distribution by gender

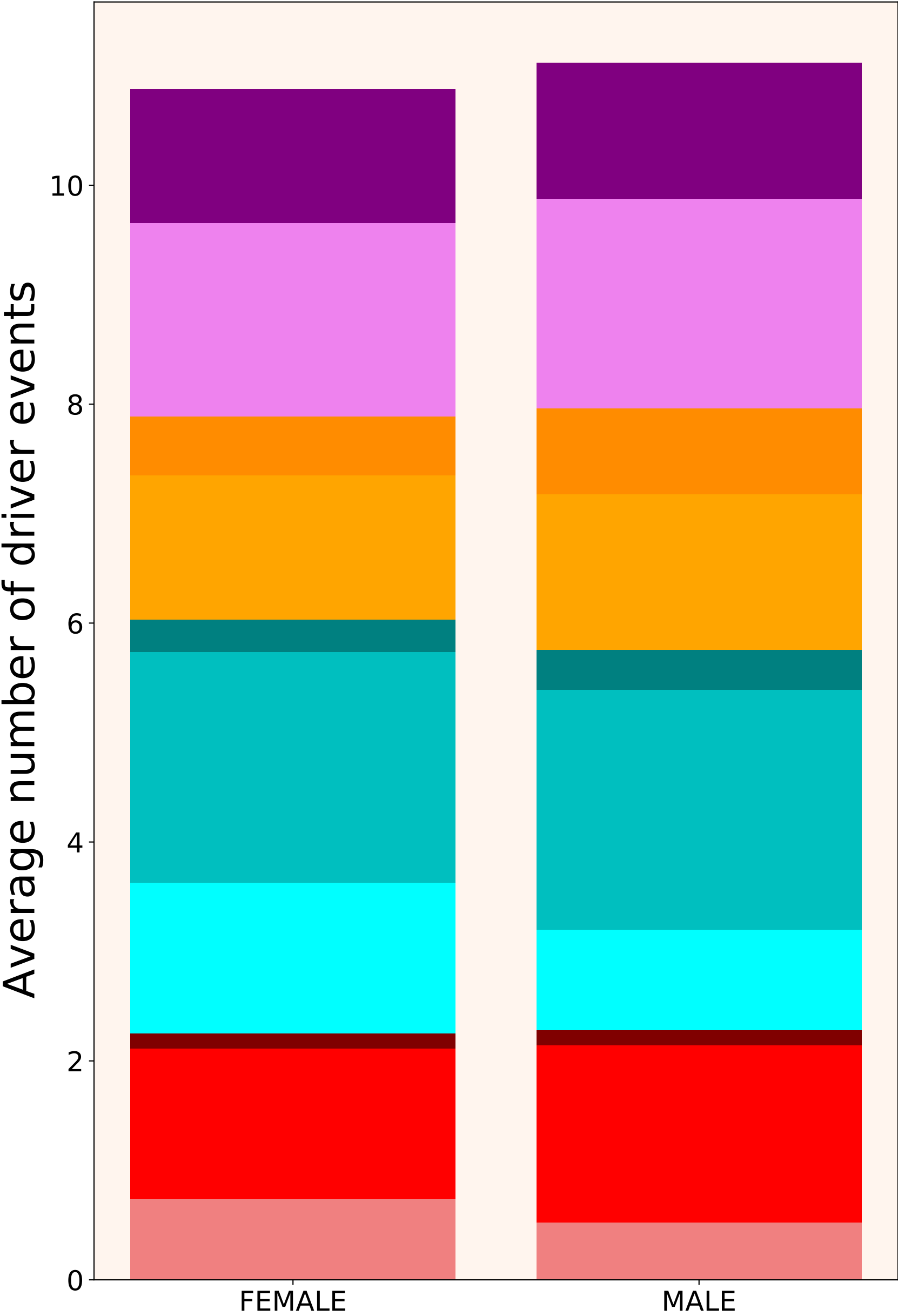

Supplement: S4 Files — (ZIP) [file pgen.1009996.s004.zip › Aneuploidy/COHORTS GISTIC2/cumulative histograms/2021_11_23_15_0_distribution_gender.pdf]

Driver event distribution by cancer stage

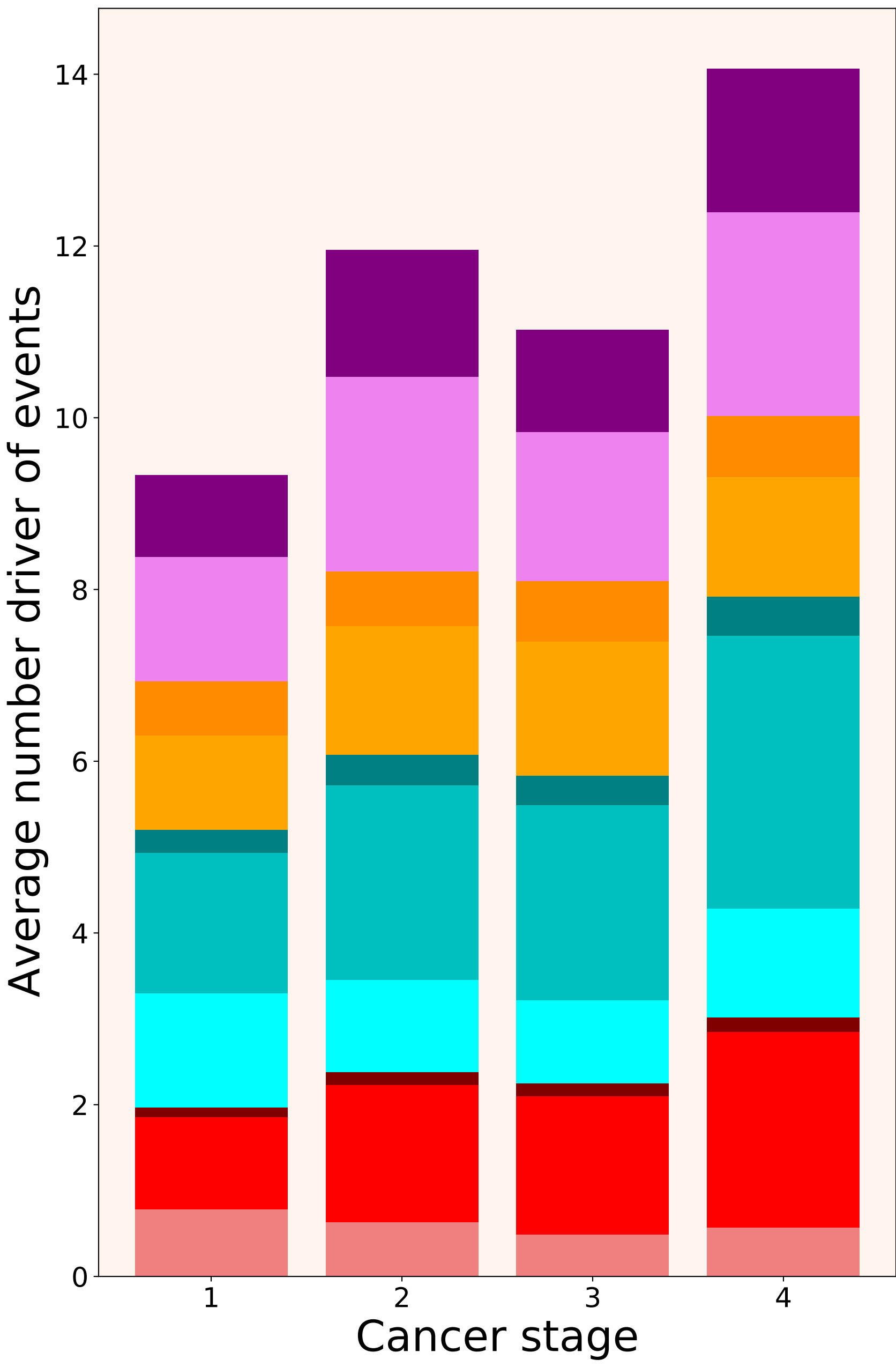

Supplement: S4 Files — (ZIP) [file pgen.1009996.s004.zip › Aneuploidy/COHORTS GISTIC2/cumulative histograms/2021_11_23_15_0_distribution_stages.pdf]

Driver event distribution by cancer stage in males

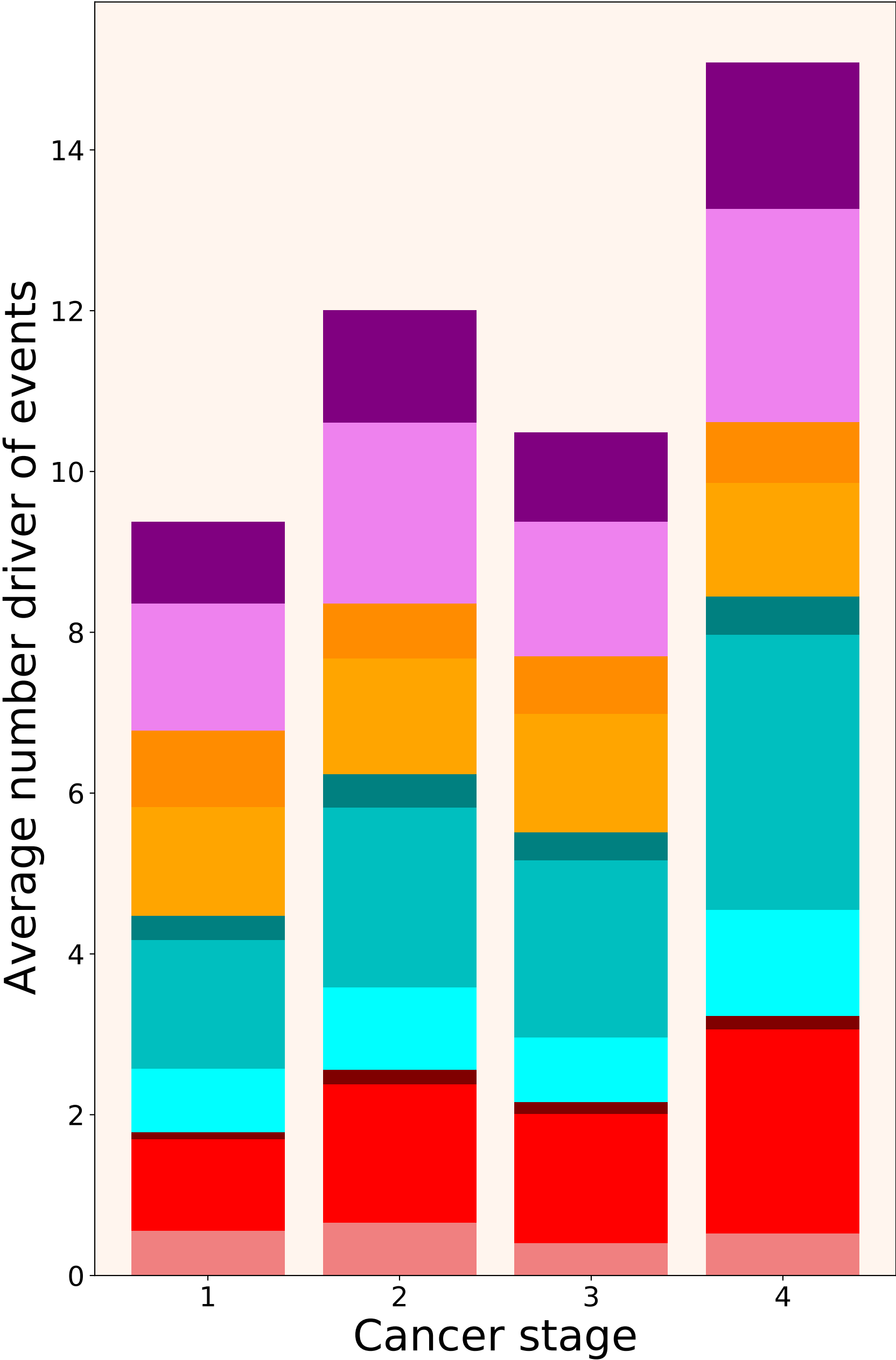

Supplement: S4 Files — (ZIP) [file pgen.1009996.s004.zip › Aneuploidy/COHORTS GISTIC2/cumulative histograms/2021_11_23_15_0_distribution_stages_males.pdf]

Driver event distribution by cancer type in females

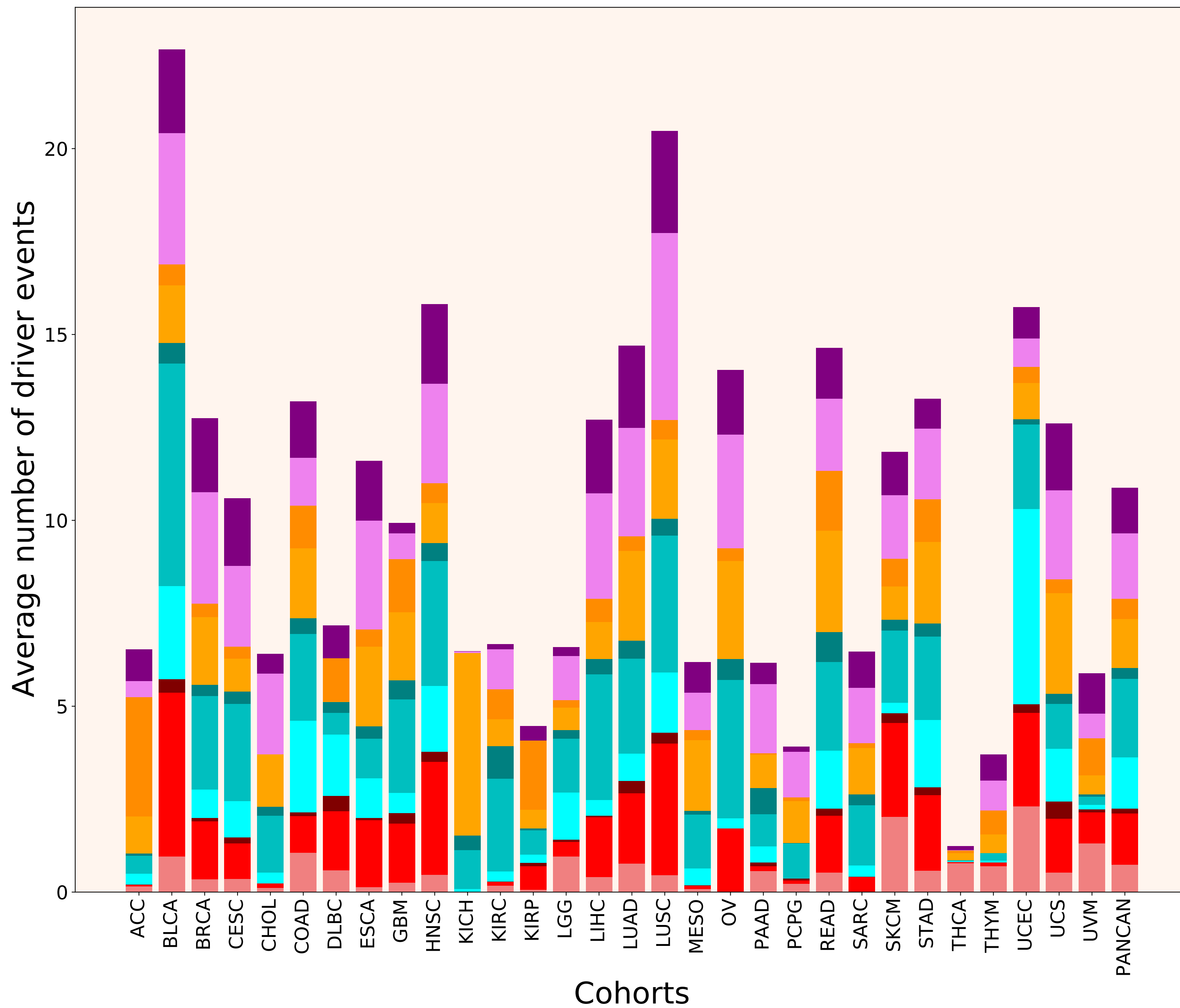

Supplement: S4 Files — (ZIP) [file pgen.1009996.s004.zip › Aneuploidy/COHORTS GISTIC2/cumulative histograms/2021_11_23_15_0_distribution_cohorts_females.pdf]

Driver event distribution by cancer type in males

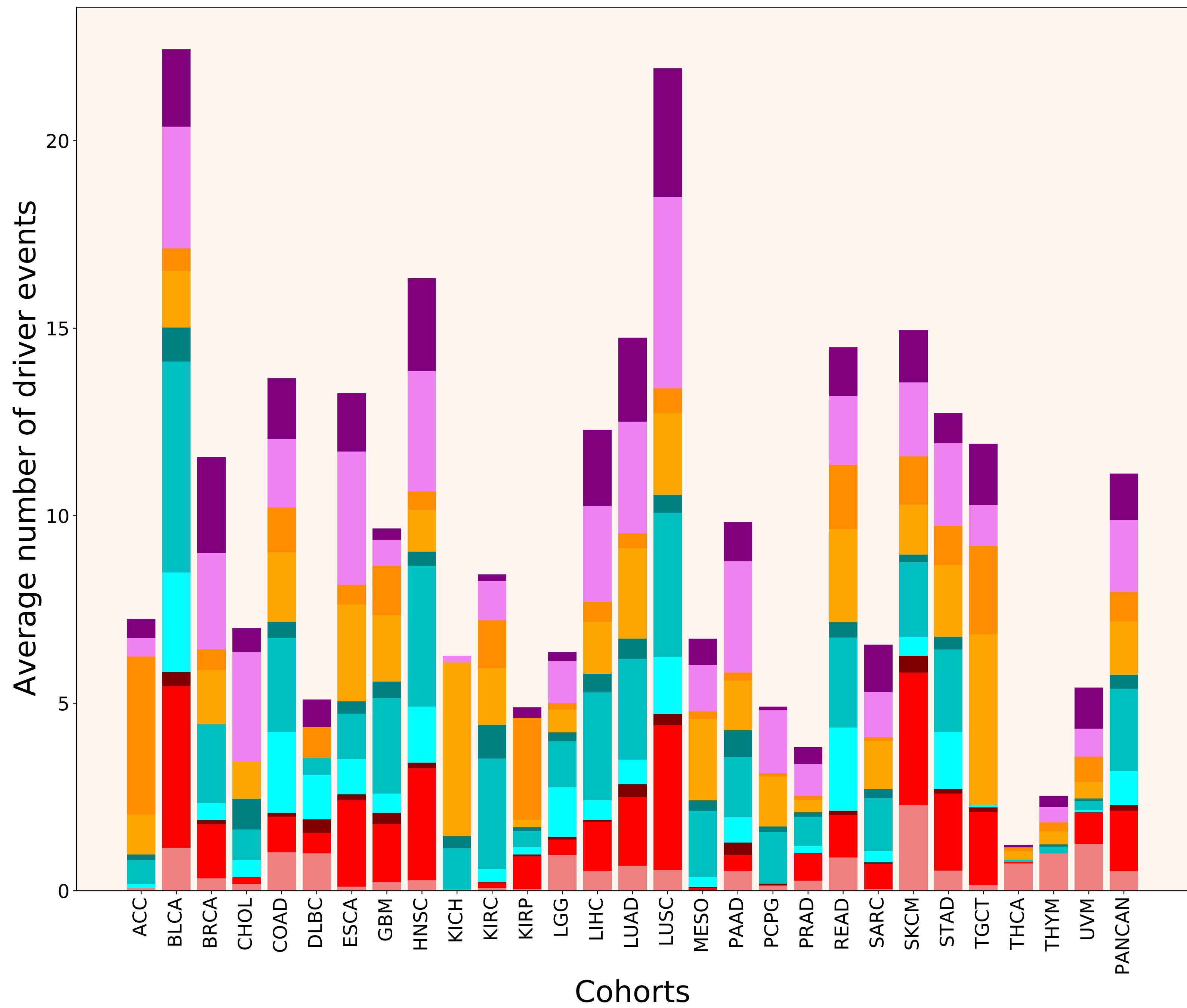

Supplement: S4 Files — (ZIP) [file pgen.1009996.s004.zip › Aneuploidy/COHORTS GISTIC2/cumulative histograms/2021_11_23_15_0_distribution_cohorts_males.pdf]

Driver event distribution by age in males

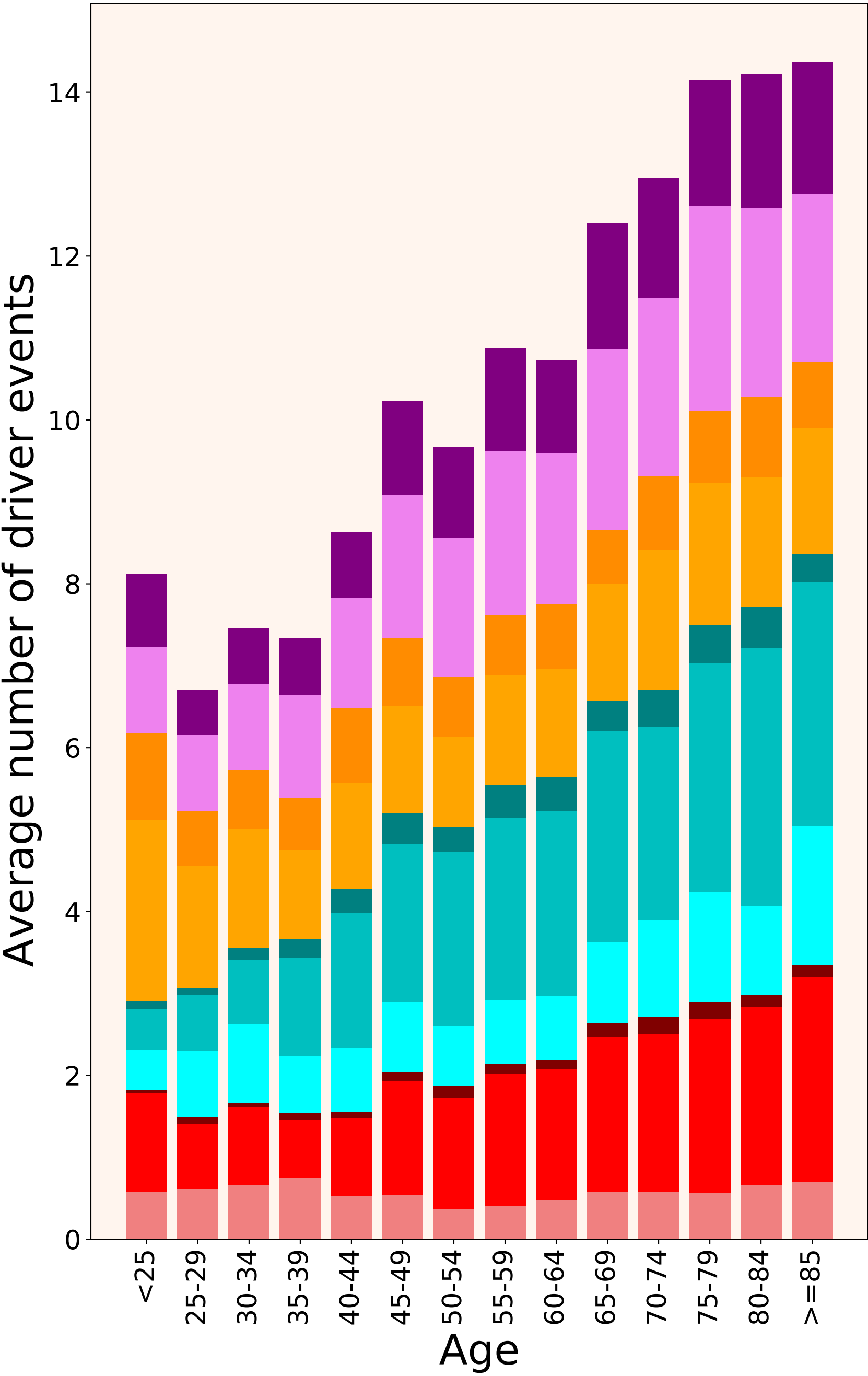

Supplement: S4 Files — (ZIP) [file pgen.1009996.s004.zip › Aneuploidy/COHORTS GISTIC2/cumulative histograms/2021_11_23_15_0_distribution_age_males.pdf]

Driver event distribution by total number of driver events per patient in males

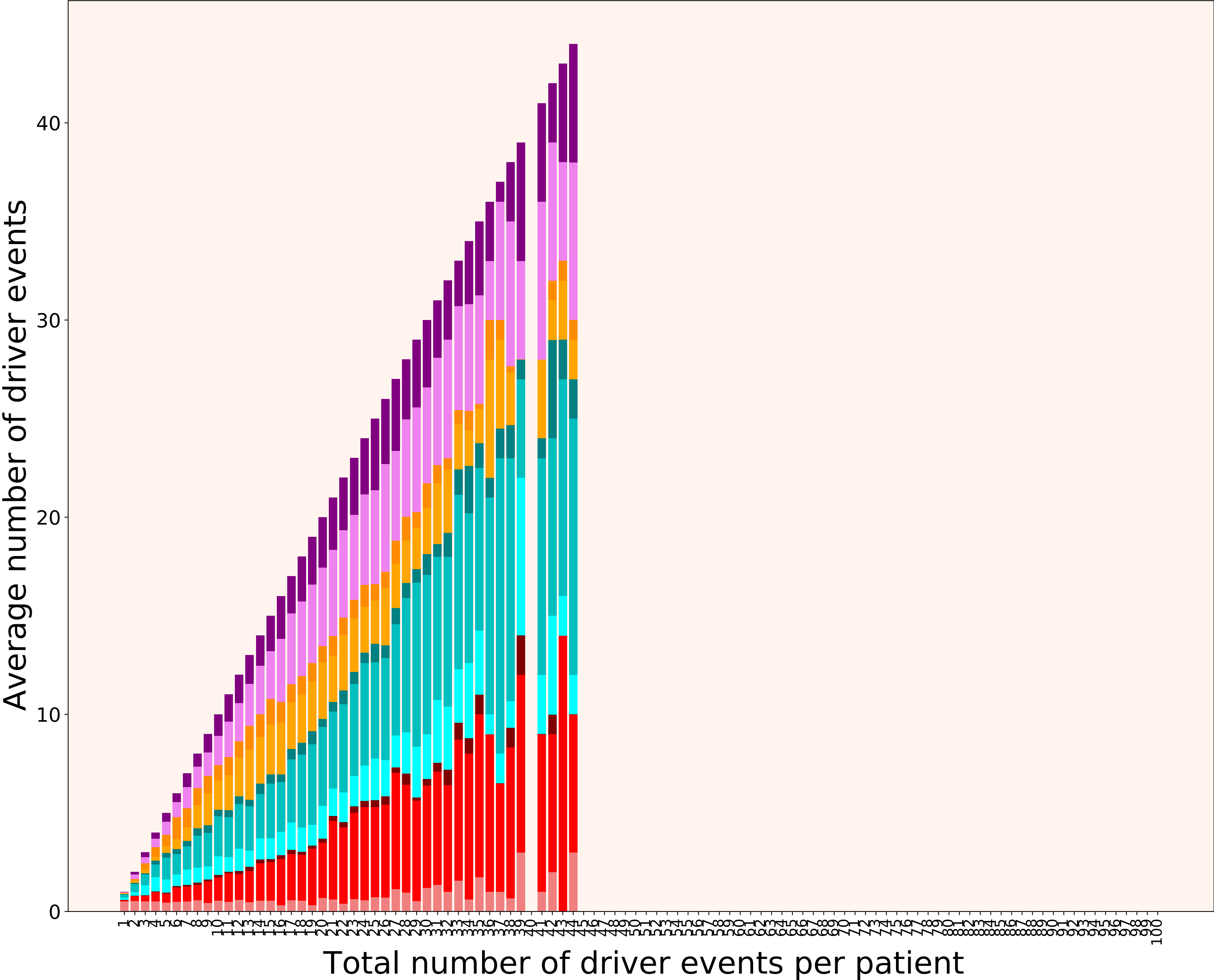

Supplement: S4 Files — (ZIP) [file pgen.1009996.s004.zip › Aneuploidy/COHORTS GISTIC2/cumulative histograms/2021_11_23_15_0_distribution_events_detailed_males.pdf]

# BRCA\_FEMALE

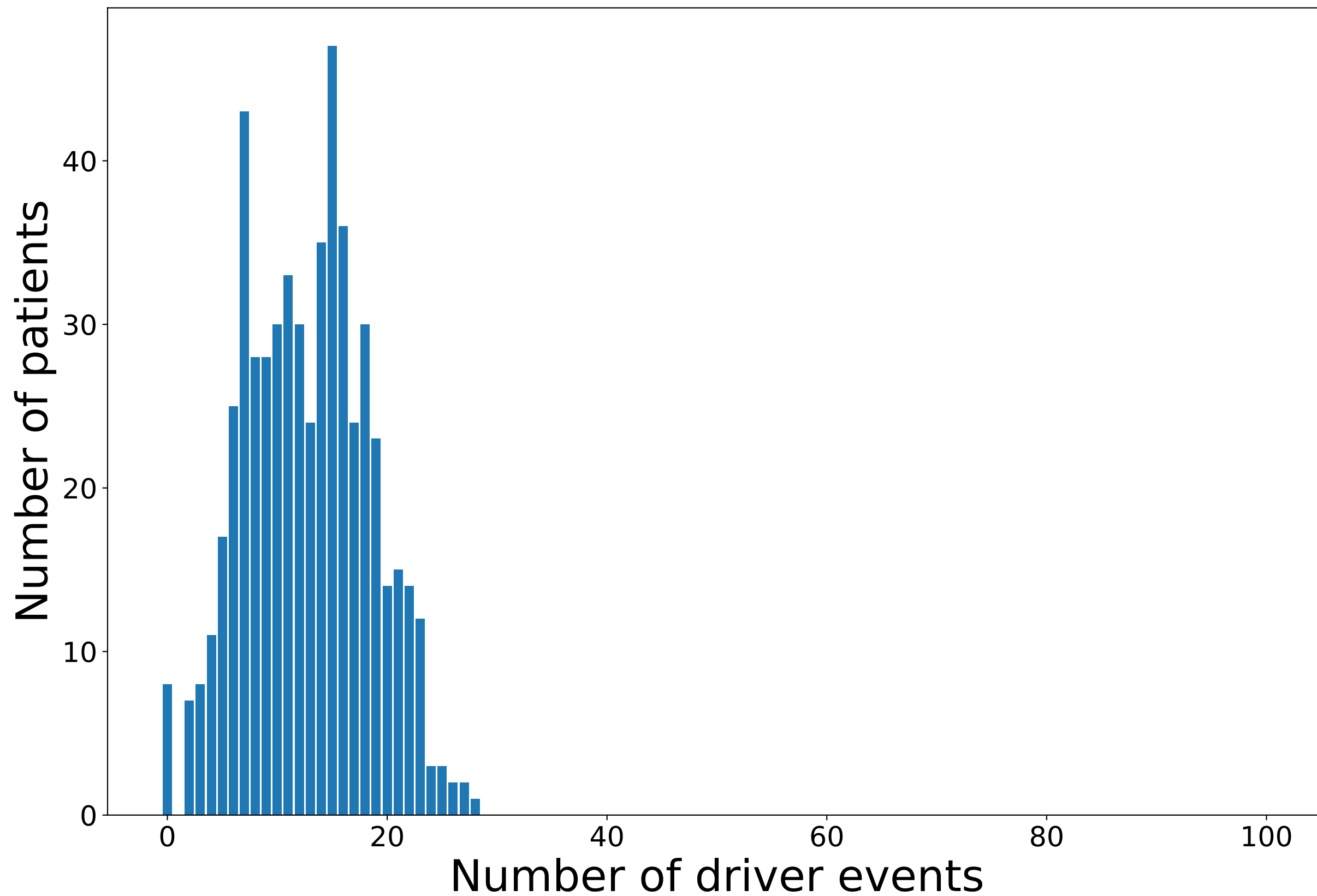

Supplement: S4 Files — (ZIP) [file pgen.1009996.s004.zip › Aneuploidy/COHORTS GISTIC2/patient distributions/2021_11_23_15_0_BRCA_FEMALE.pdf]

# PANCAN\_FEMALE

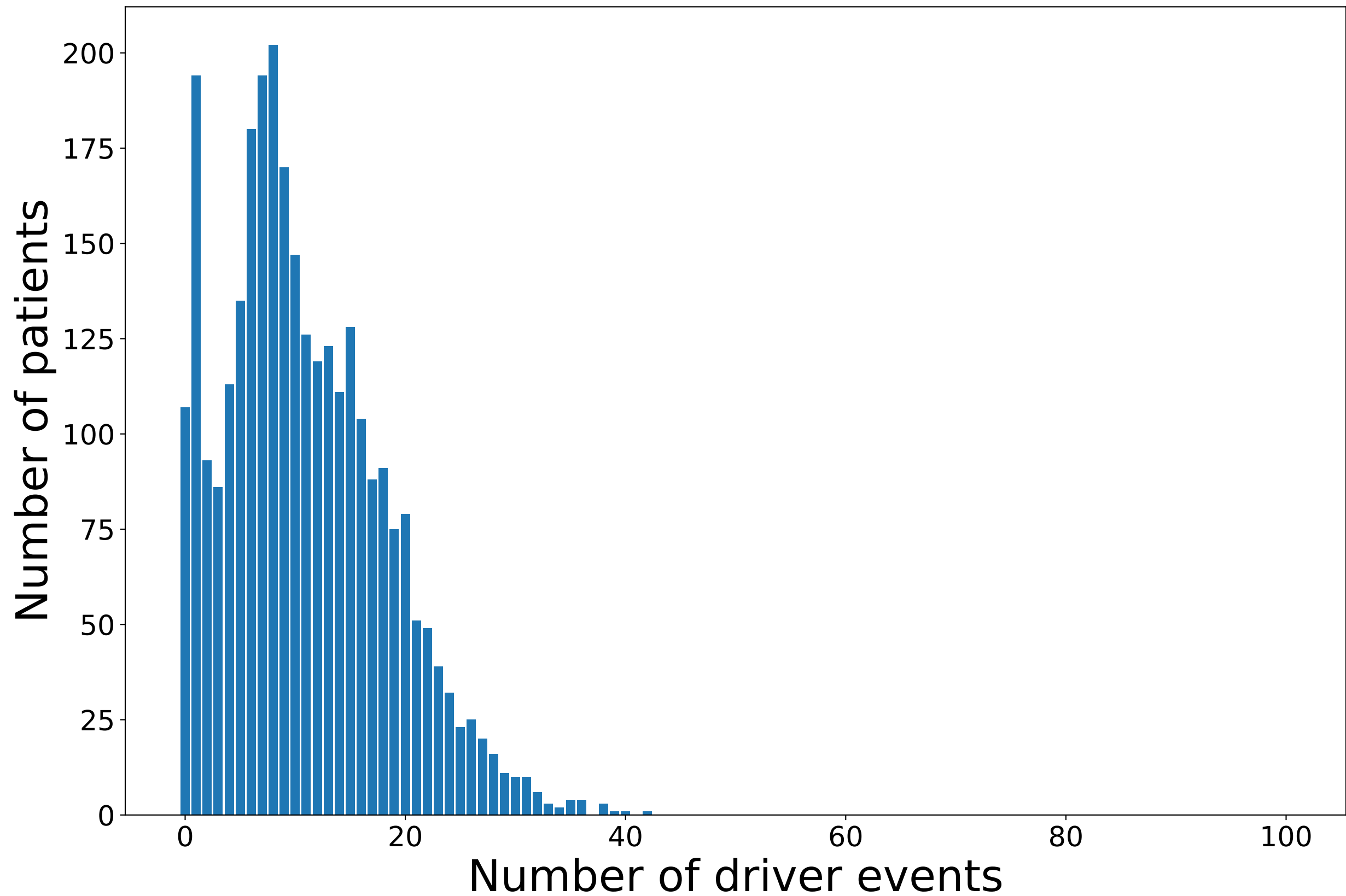

Supplement: S4 Files — (ZIP) [file pgen.1009996.s004.zip › Aneuploidy/COHORTS GISTIC2/patient distributions/2021_11_23_15_0_PANCAN_FEMALE.pdf]

# PAAD\_MALE

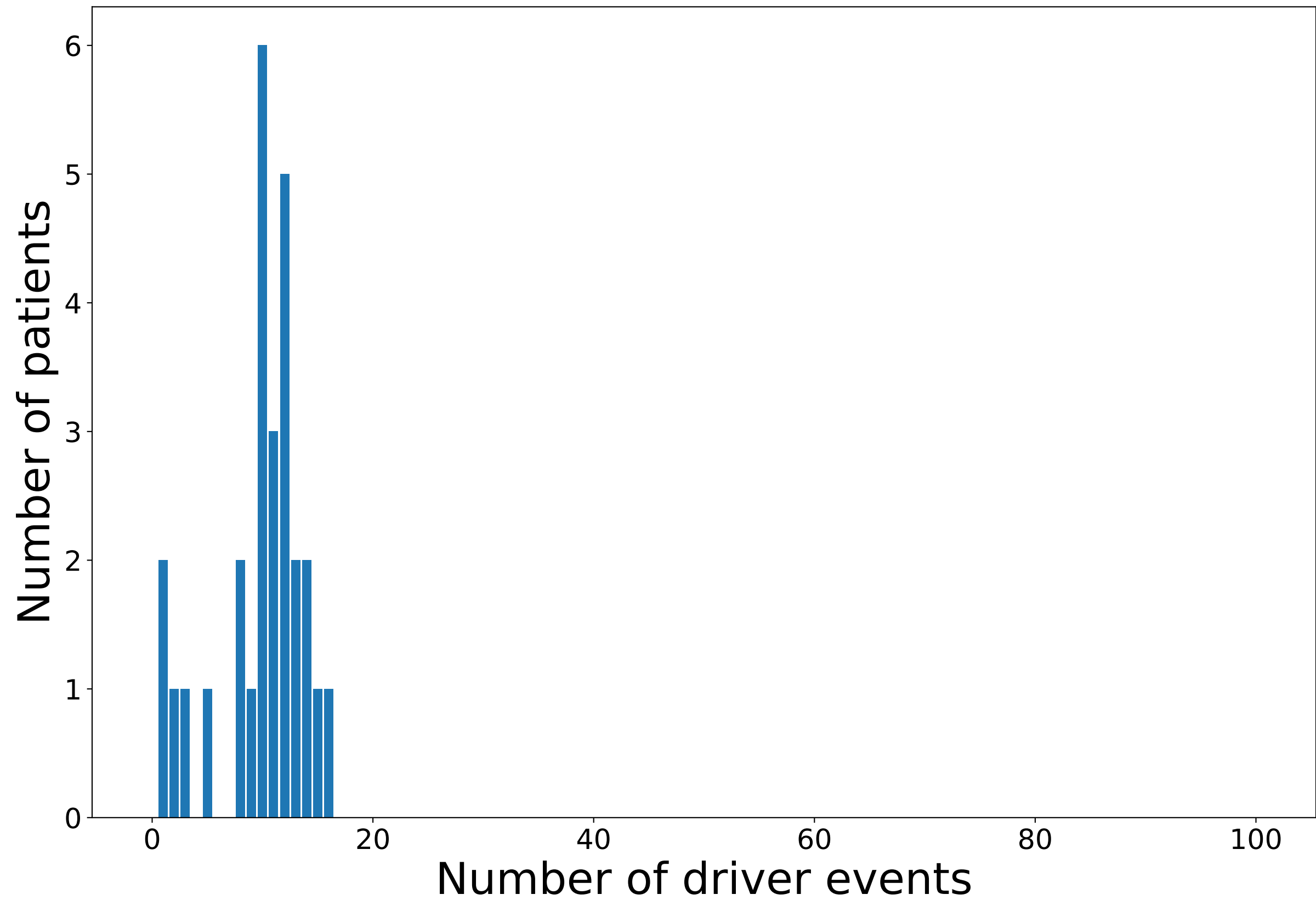

Supplement: S4 Files — (ZIP) [file pgen.1009996.s004.zip › Aneuploidy/COHORTS GISTIC2/patient distributions/2021_11_23_15_0_PAAD_MALE.pdf]

# PRAD

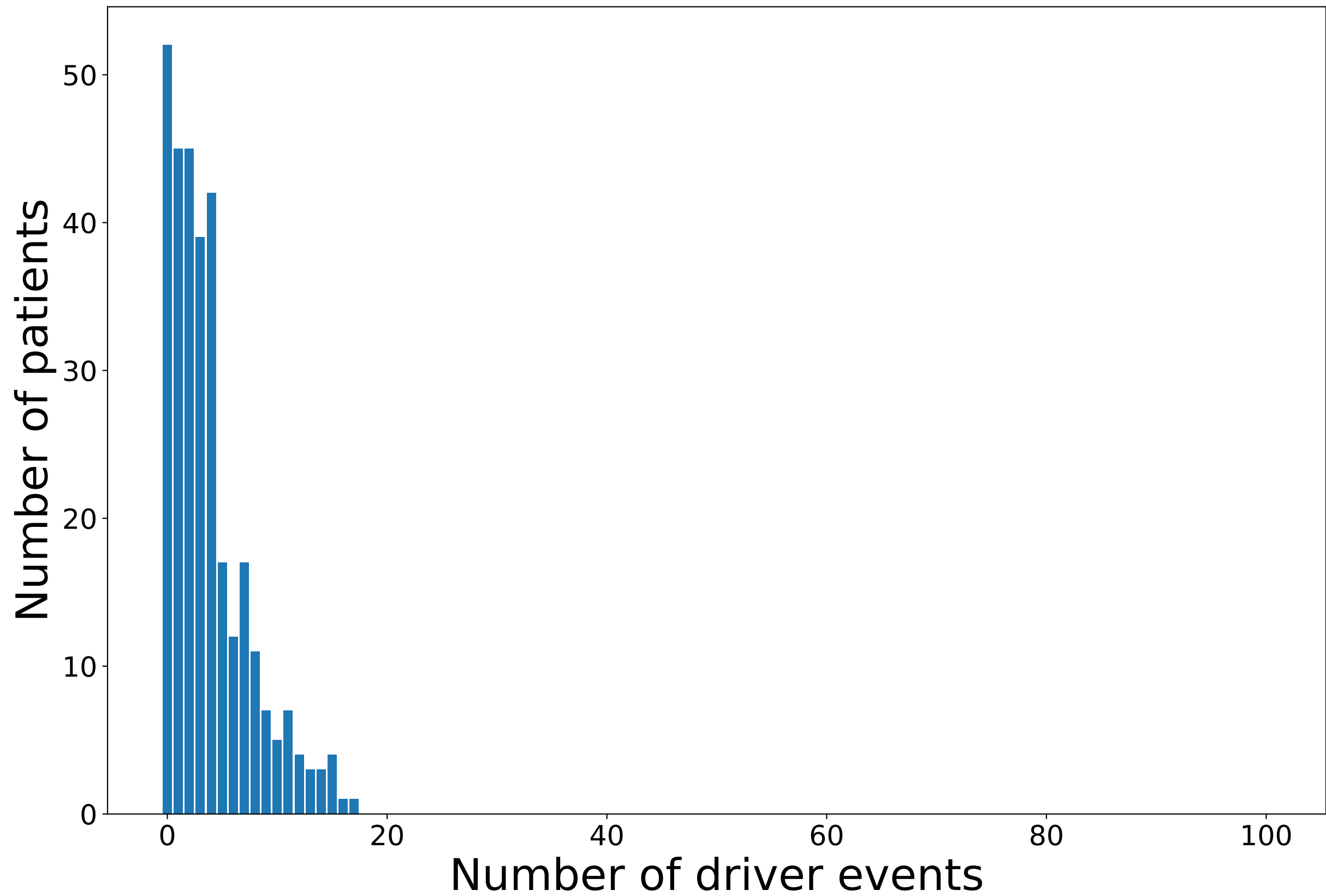

Supplement: S4 Files — (ZIP) [file pgen.1009996.s004.zip › Aneuploidy/COHORTS GISTIC2/patient distributions/2021_11_23_15_0_PRAD.pdf]

# ESCA\_FEMALE

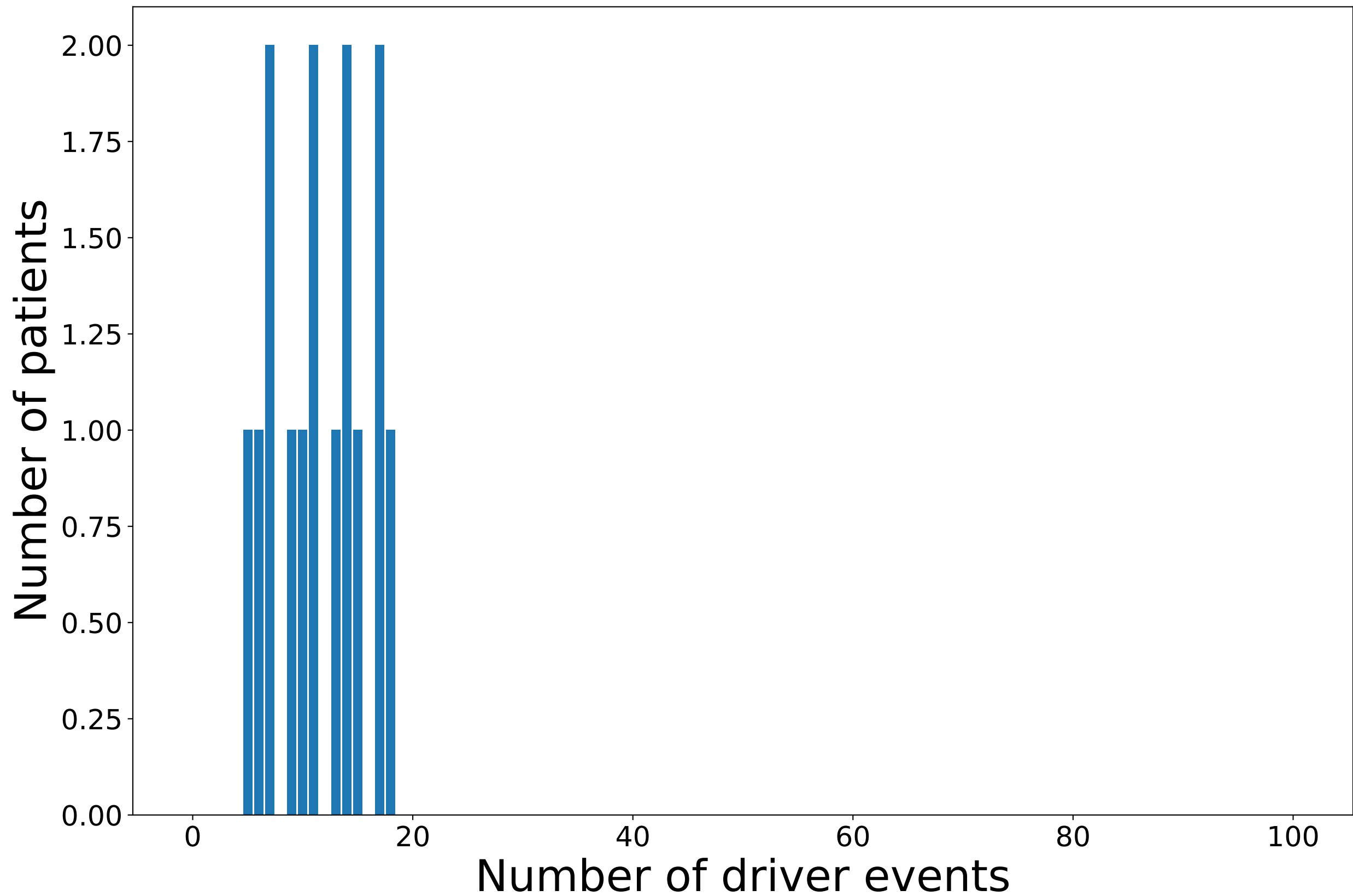

Supplement: S4 Files — (ZIP) [file pgen.1009996.s004.zip › Aneuploidy/COHORTS GISTIC2/patient distributions/2021_11_23_15_0_ESCA_FEMALE.pdf]

# SARC

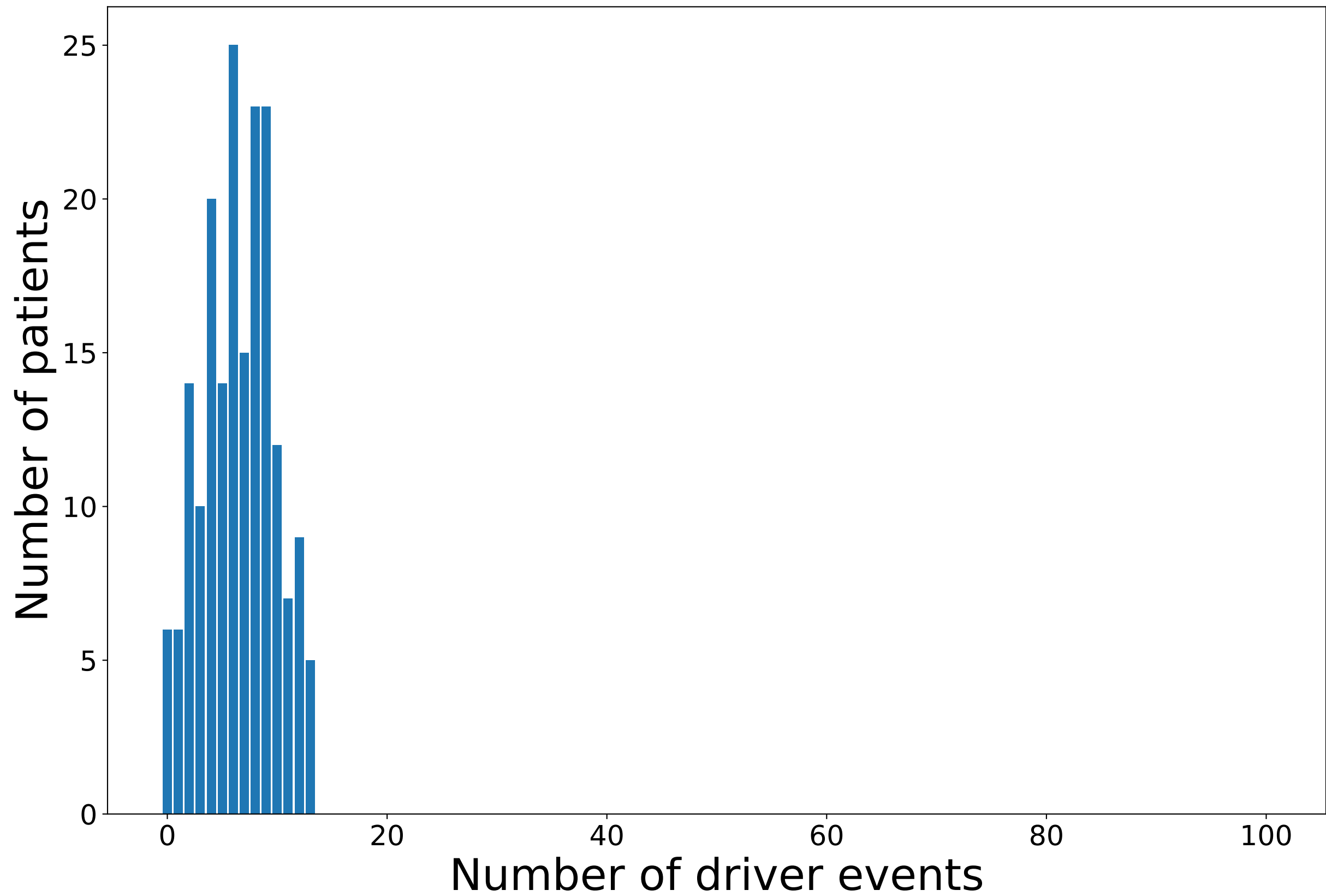

Supplement: S4 Files — (ZIP) [file pgen.1009996.s004.zip › Aneuploidy/COHORTS GISTIC2/patient distributions/2021_11_23_15_0_SARC.pdf]

# UCS

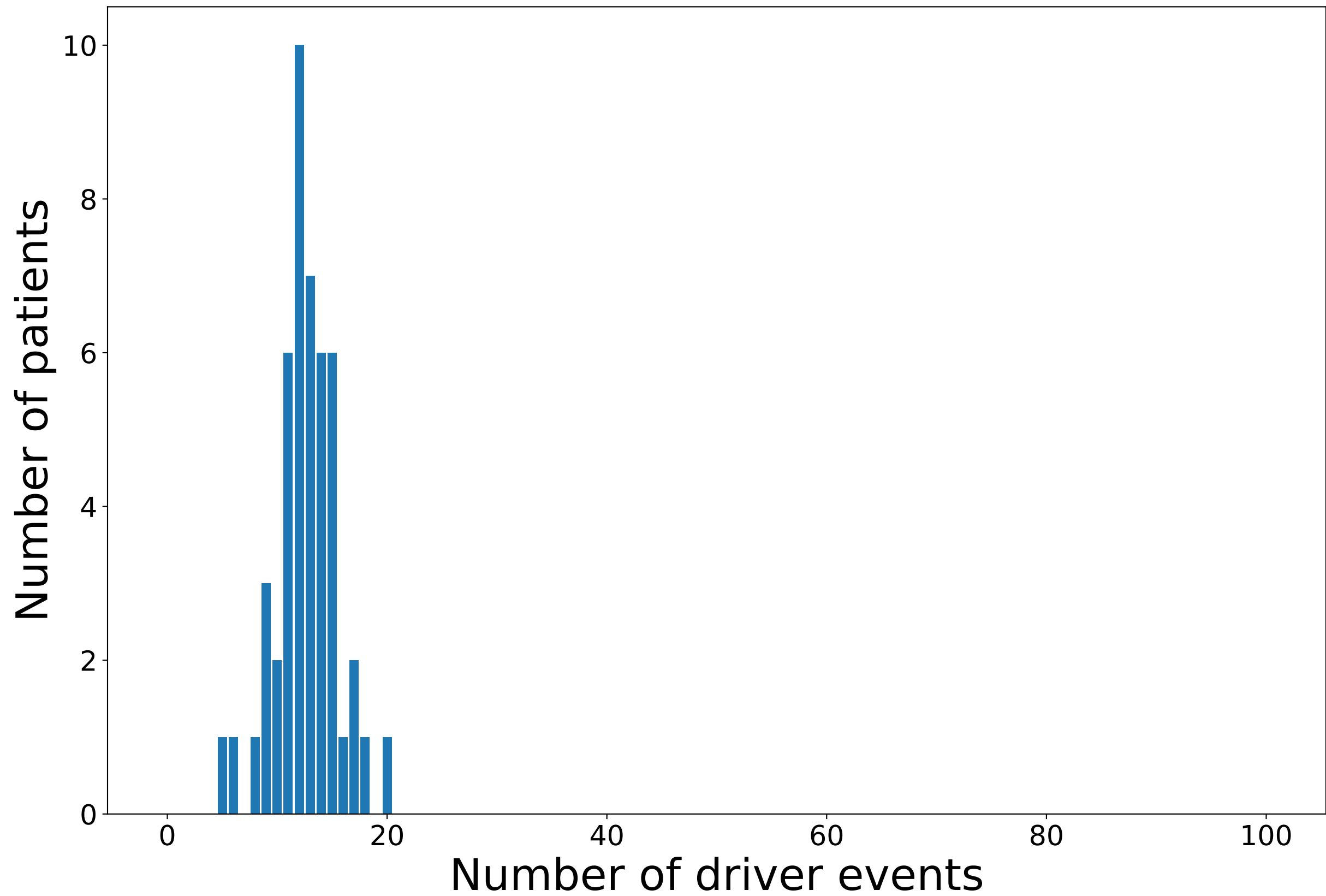

Supplement: S4 Files — (ZIP) [file pgen.1009996.s004.zip › Aneuploidy/COHORTS GISTIC2/patient distributions/2021_11_23_15_0_UCS.pdf]

# PRAD\_MALE

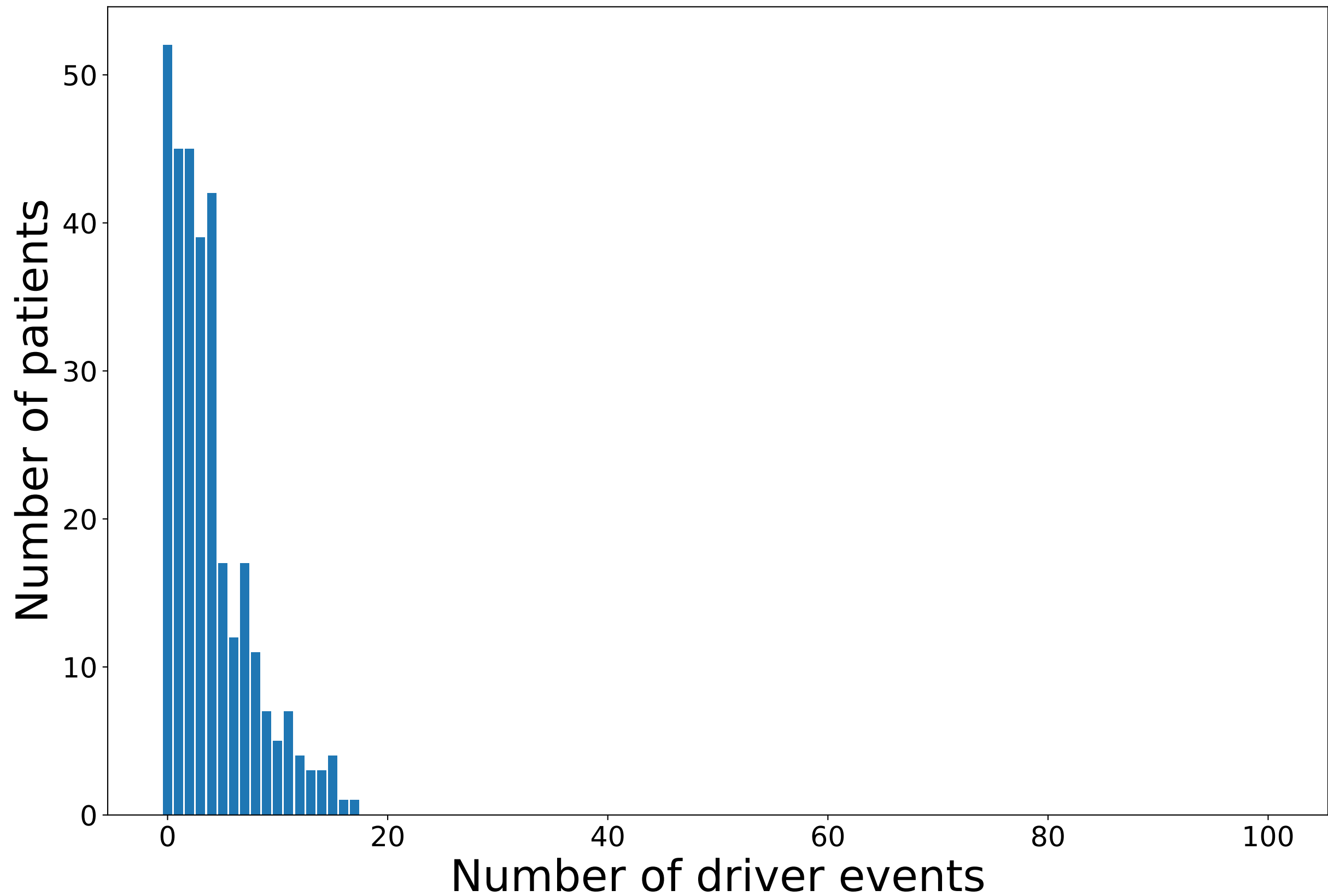

Supplement: S4 Files — (ZIP) [file pgen.1009996.s004.zip › Aneuploidy/COHORTS GISTIC2/patient distributions/2021_11_23_15_0_PRAD_MALE.pdf]

# KICH\_FEMALE

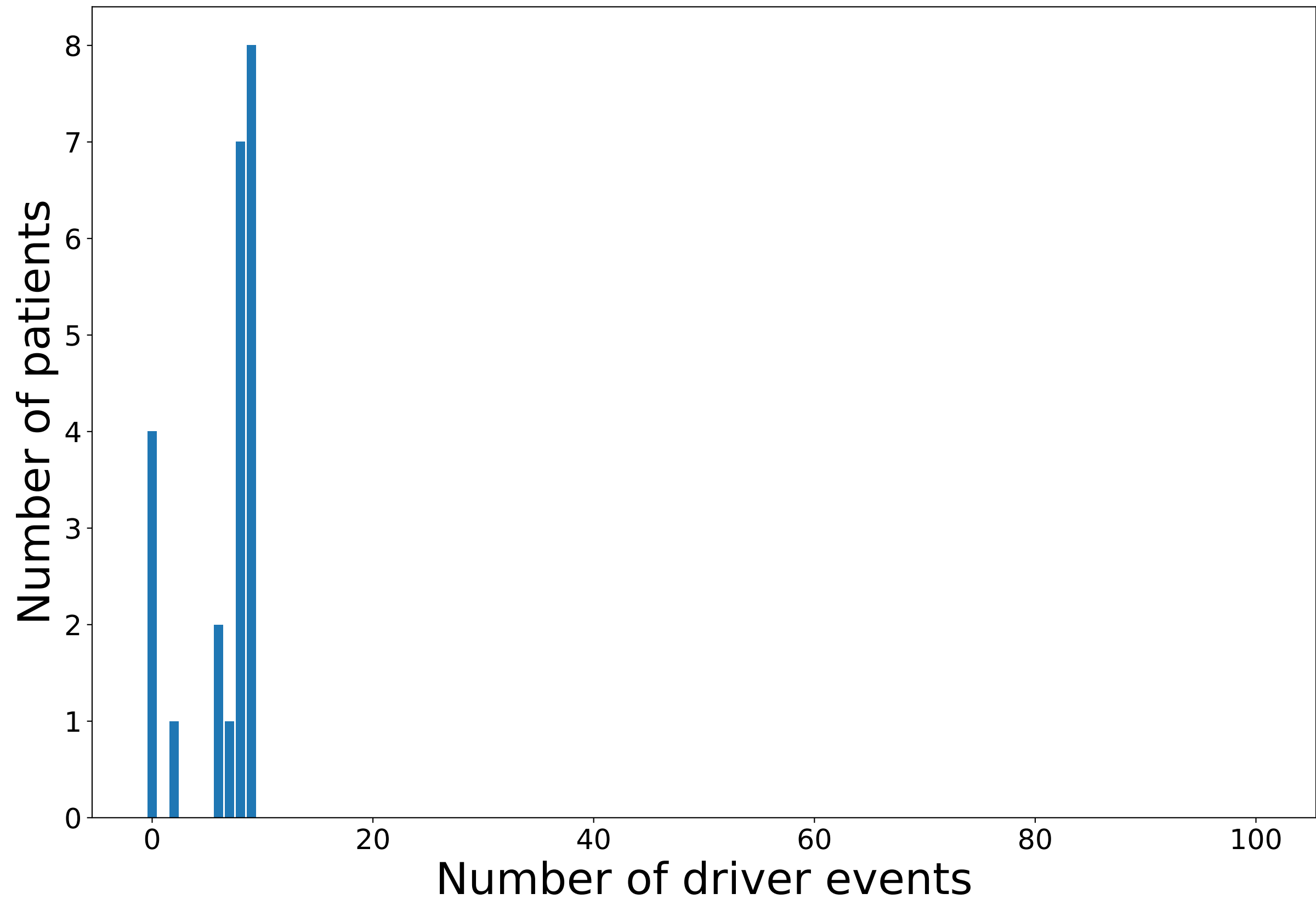

Supplement: S4 Files — (ZIP) [file pgen.1009996.s004.zip › Aneuploidy/COHORTS GISTIC2/patient distributions/2021_11_23_15_0_KICH_FEMALE.pdf]

# PANCAN

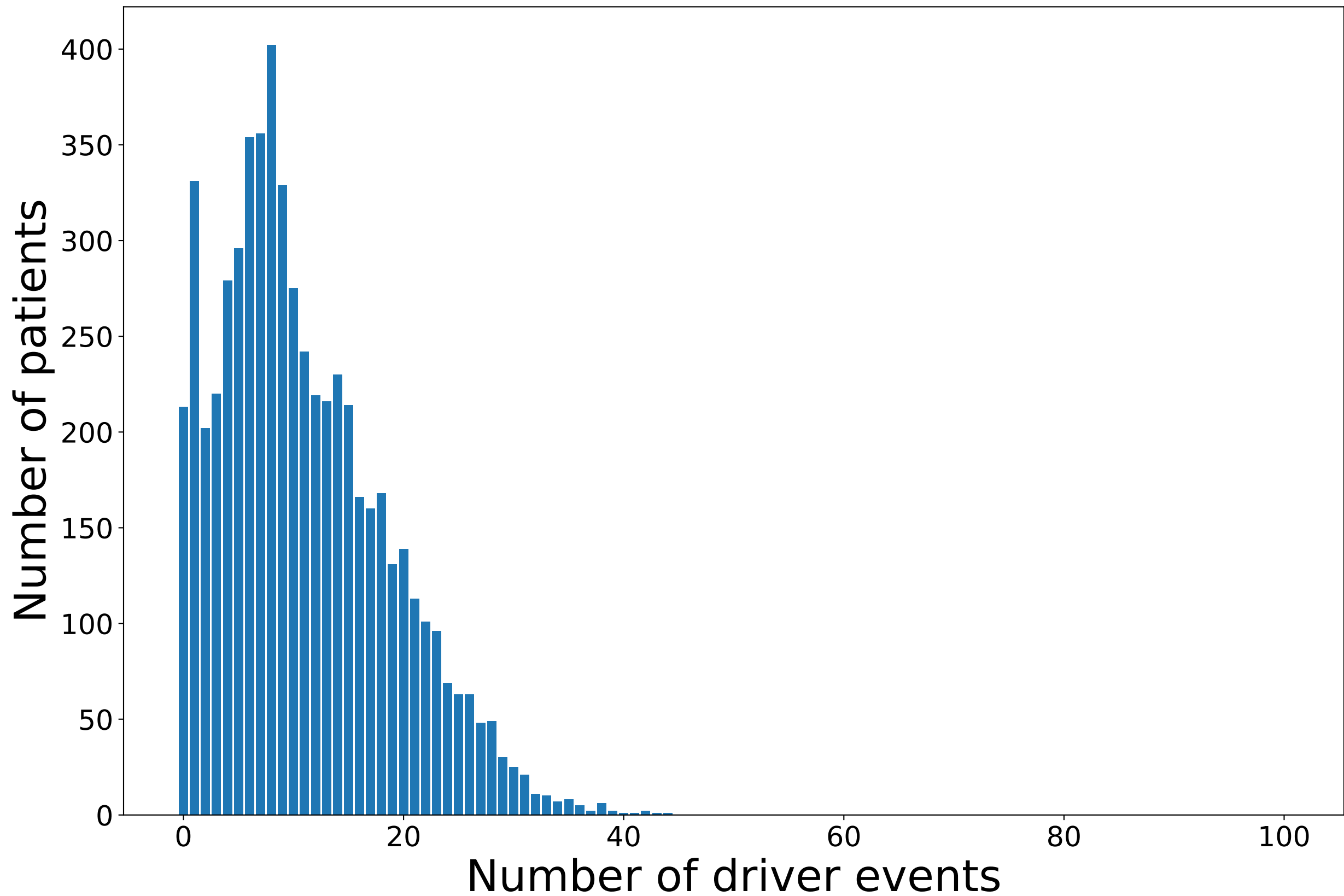

Supplement: S4 Files — (ZIP) [file pgen.1009996.s004.zip › Aneuploidy/COHORTS GISTIC2/patient distributions/2021_11_23_15_0_PANCAN.pdf]

# LUAD\_MALE

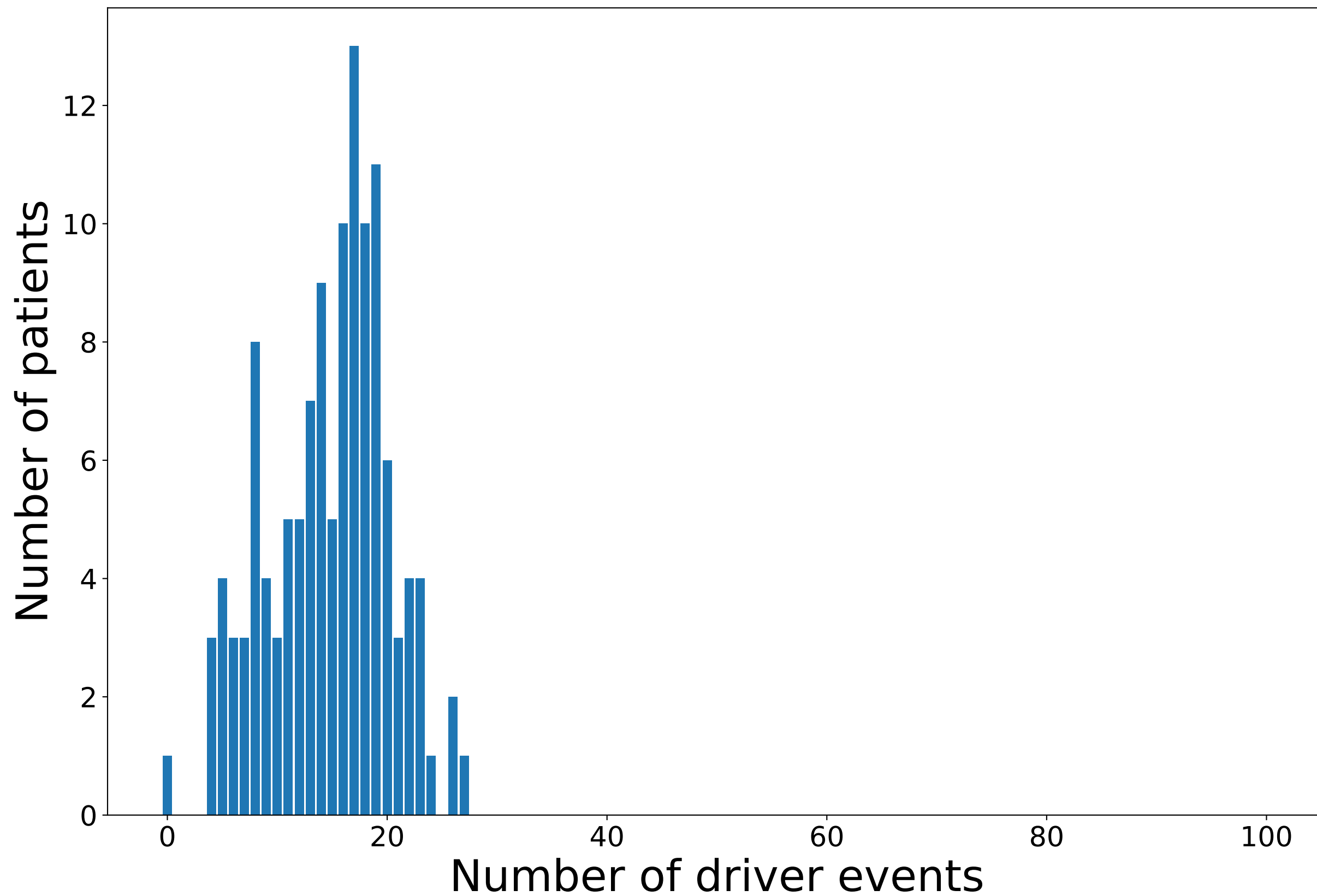

Supplement: S4 Files — (ZIP) [file pgen.1009996.s004.zip › Aneuploidy/COHORTS GISTIC2/patient distributions/2021_11_23_15_0_LUAD_MALE.pdf]

# DLBC\_FEMALE

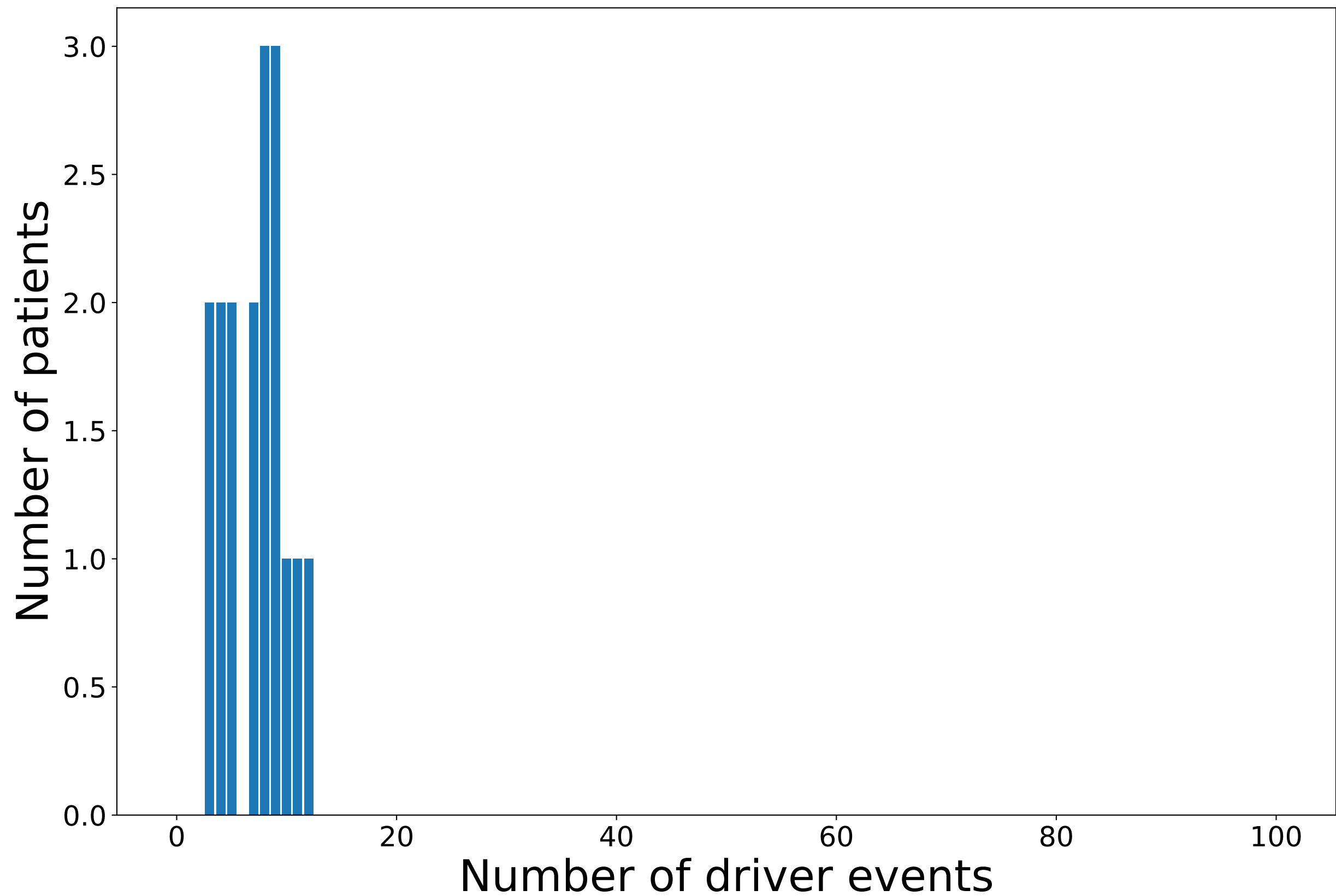

Supplement: S4 Files — (ZIP) [file pgen.1009996.s004.zip › Aneuploidy/COHORTS GISTIC2/patient distributions/2021_11_23_15_0_DLBC_FEMALE.pdf]

# LUAD

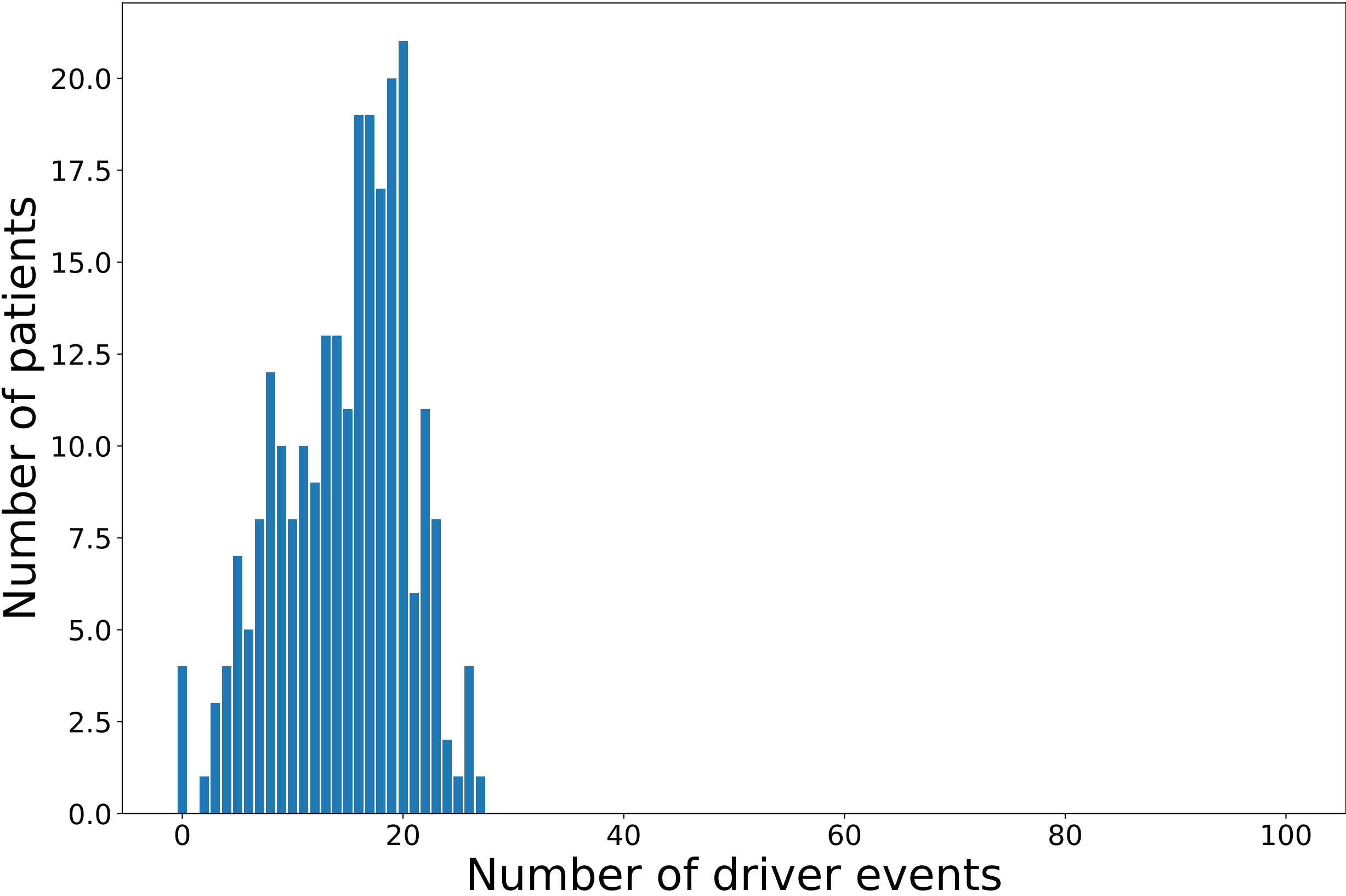

Supplement: S4 Files — (ZIP) [file pgen.1009996.s004.zip › Aneuploidy/COHORTS GISTIC2/patient distributions/2021_11_23_15_0_LUAD.pdf]

# LUSC\_MALE

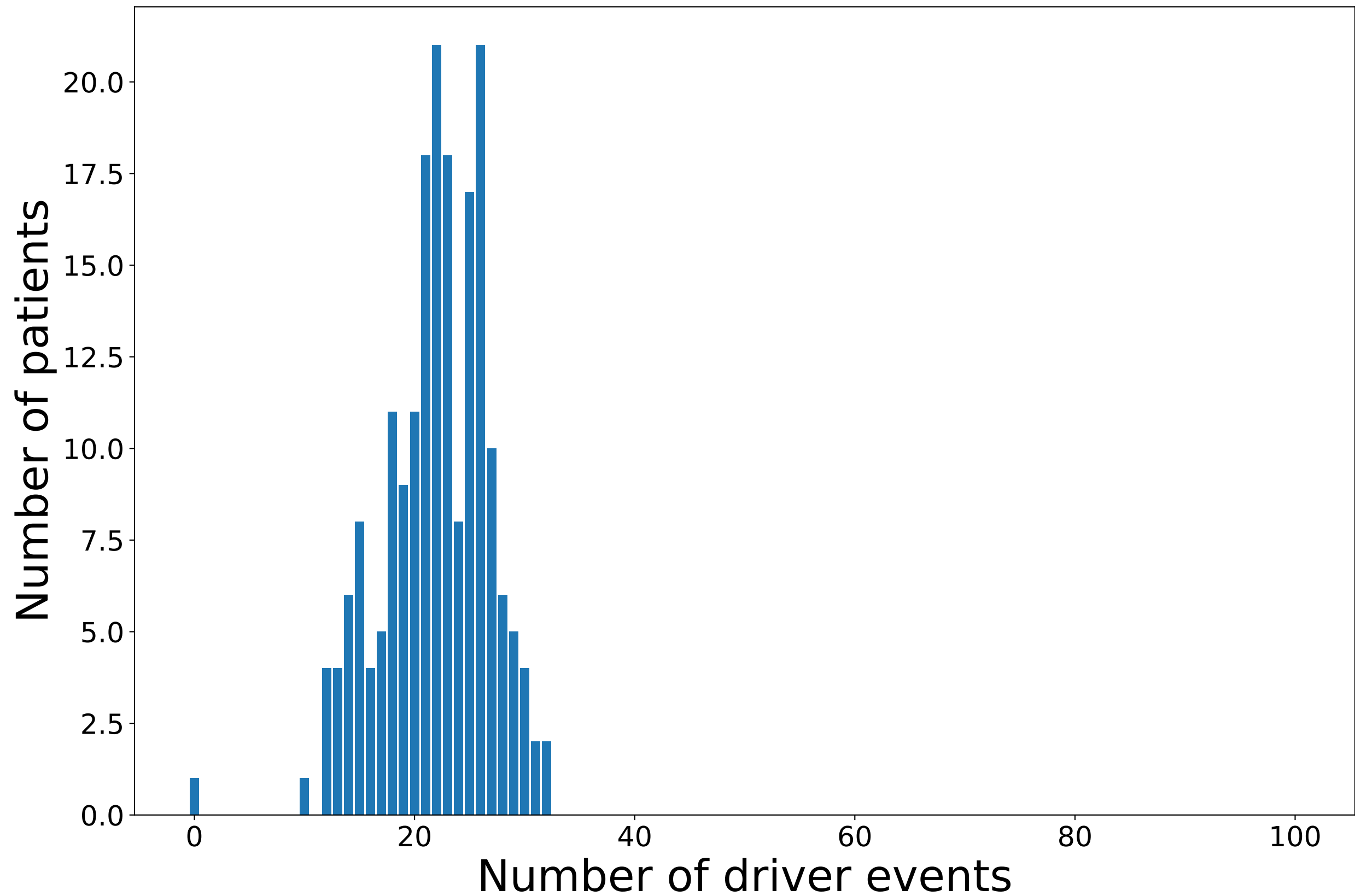

Supplement: S4 Files — (ZIP) [file pgen.1009996.s004.zip › Aneuploidy/COHORTS GISTIC2/patient distributions/2021_11_23_15_0_LUSC_MALE.pdf]

# UVM\_FEMALE

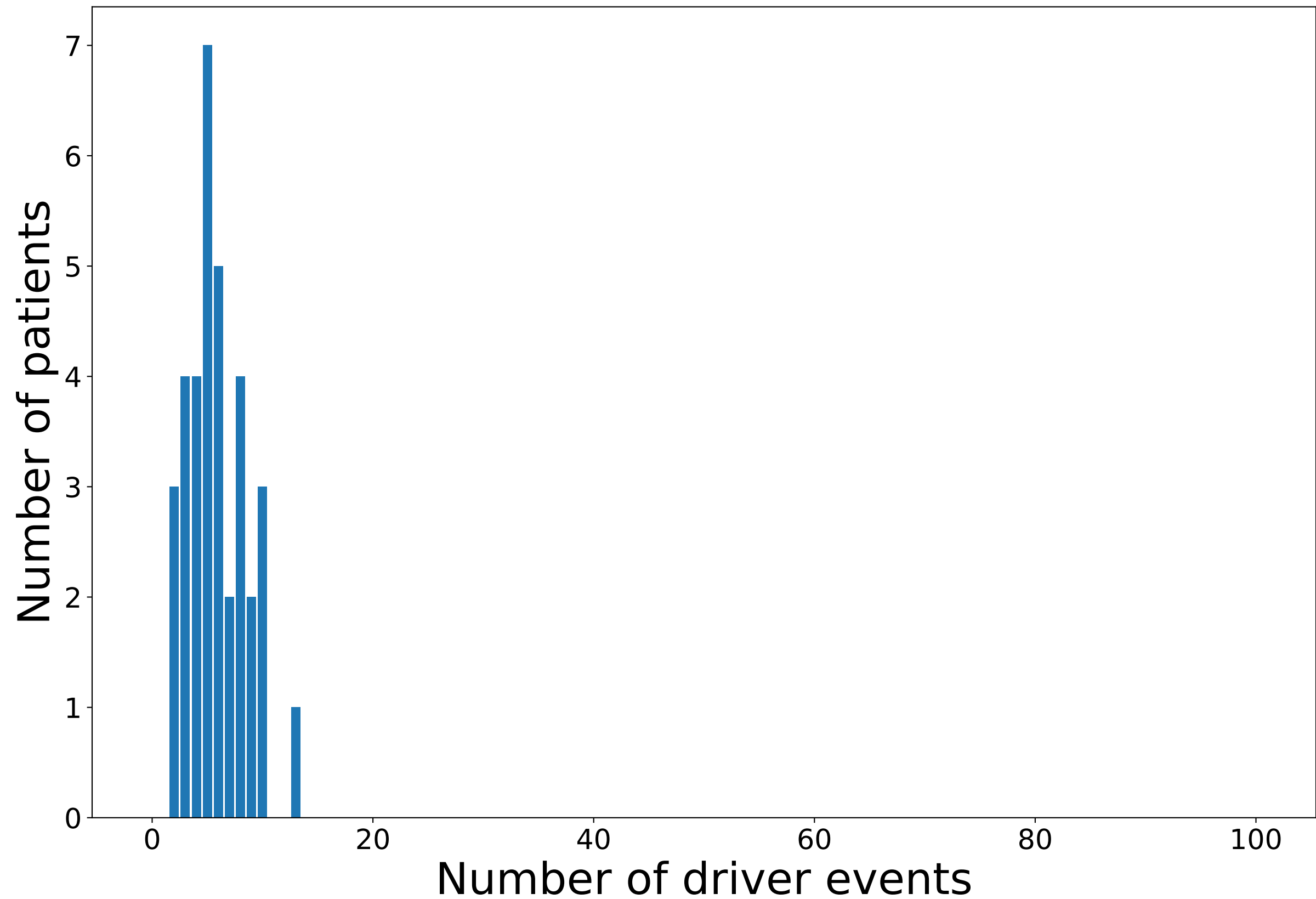

Supplement: S4 Files — (ZIP) [file pgen.1009996.s004.zip › Aneuploidy/COHORTS GISTIC2/patient distributions/2021_11_23_15_0_UVM_FEMALE.pdf]

# MESO\_MALE

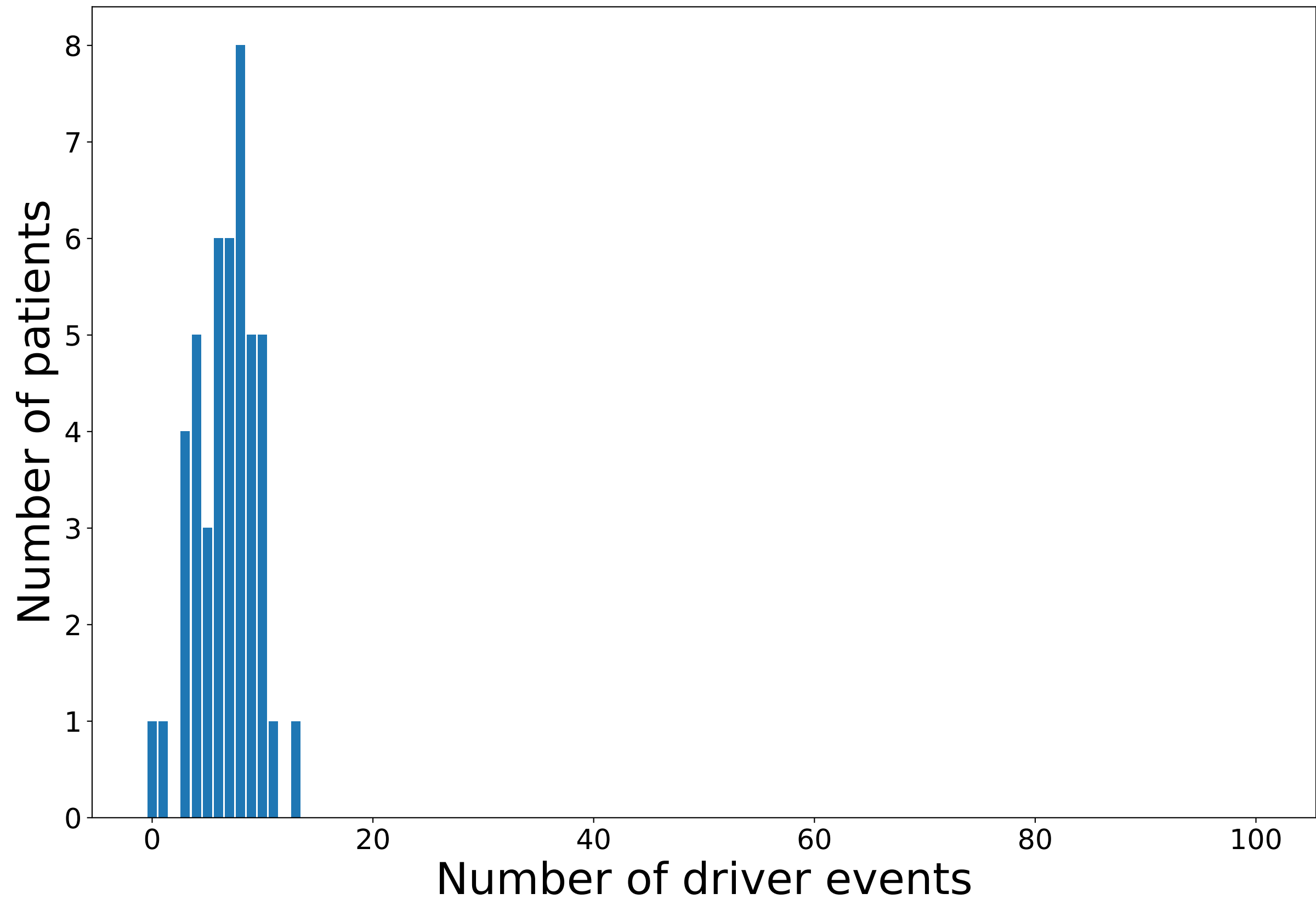

Supplement: S4 Files — (ZIP) [file pgen.1009996.s004.zip › Aneuploidy/COHORTS GISTIC2/patient distributions/2021_11_23_15_0_MESO_MALE.pdf]

# TGCT\_MALE

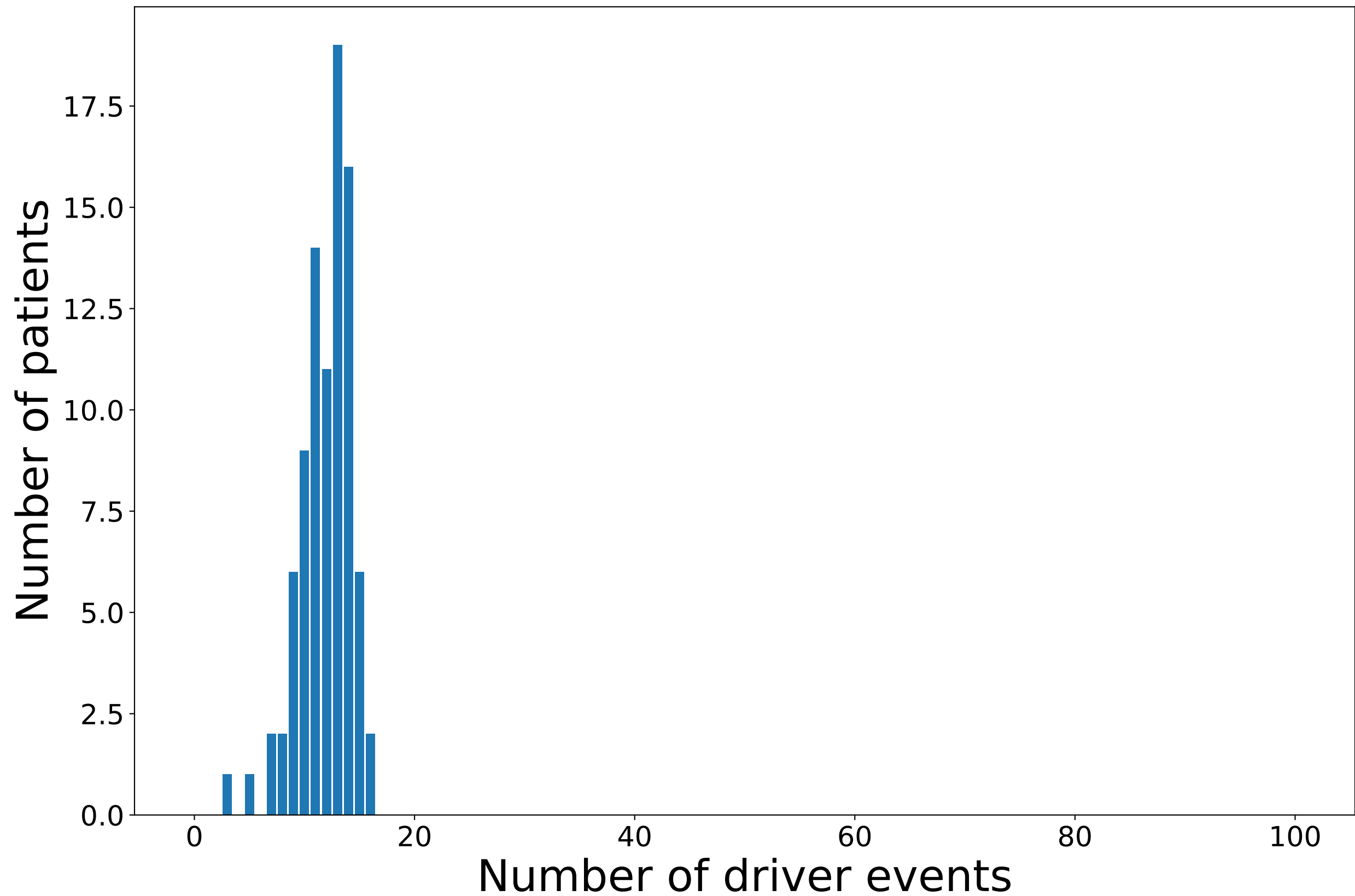

Supplement: S4 Files — (ZIP) [file pgen.1009996.s004.zip › Aneuploidy/COHORTS GISTIC2/patient distributions/2021_11_23_15_0_TGCT_MALE.pdf]

# KIRP\_FEMALE

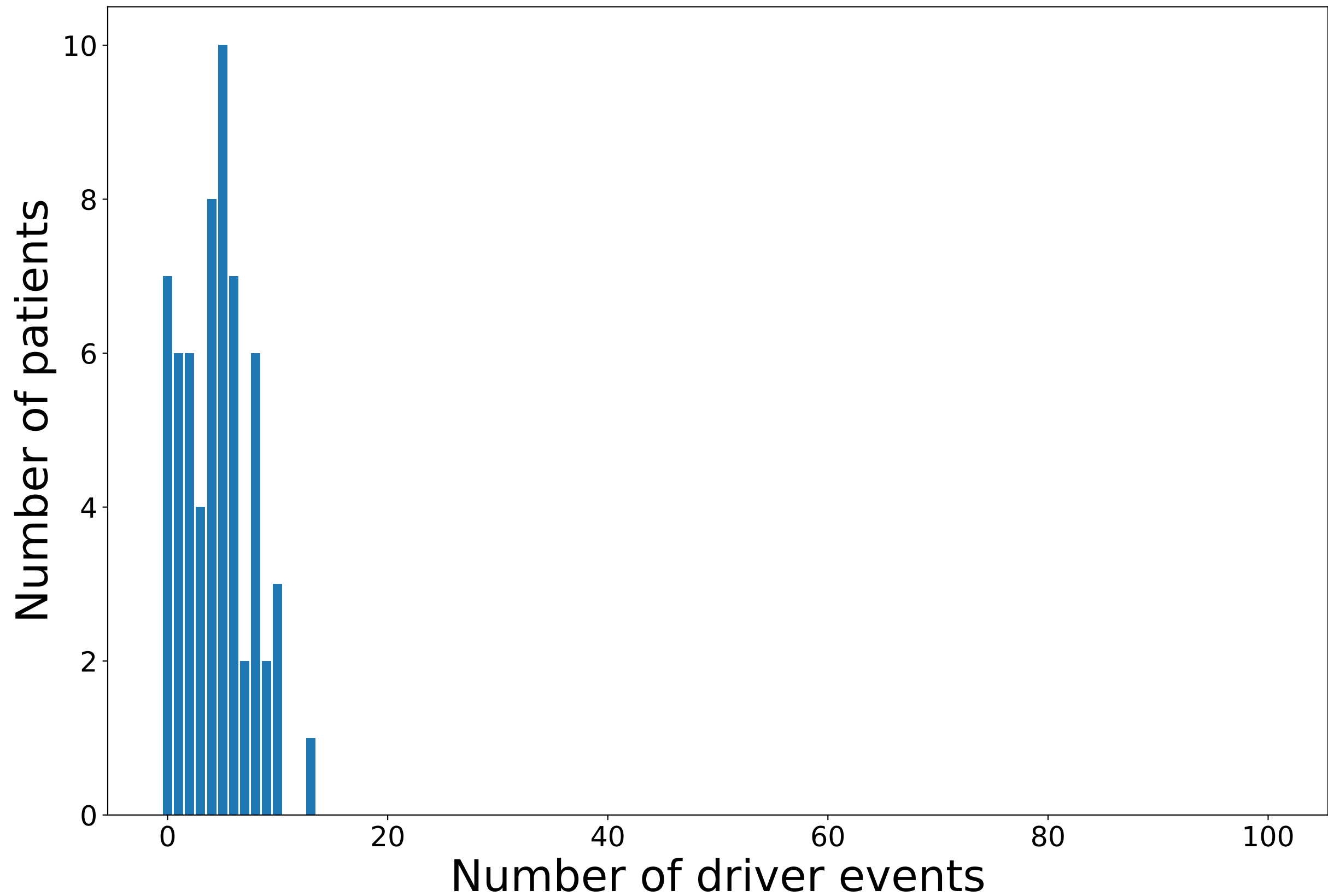

Supplement: S4 Files — (ZIP) [file pgen.1009996.s004.zip › Aneuploidy/COHORTS GISTIC2/patient distributions/2021_11_23_15_0_KIRP_FEMALE.pdf]

# CHOL\_FEMALE

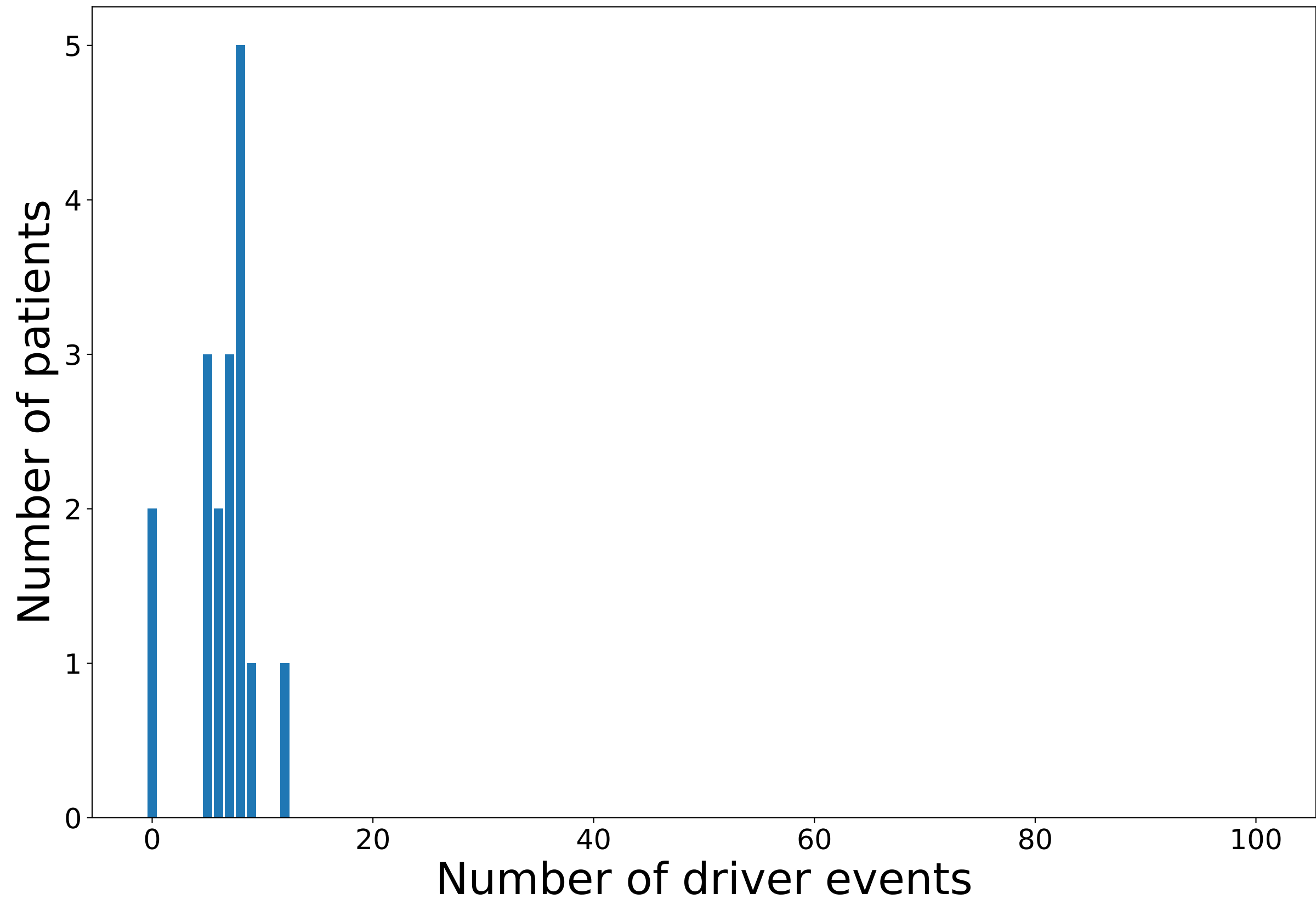

Supplement: S4 Files — (ZIP) [file pgen.1009996.s004.zip › Aneuploidy/COHORTS GISTIC2/patient distributions/2021_11_23_15_0_CHOL_FEMALE.pdf]

# SKCM\_MALE

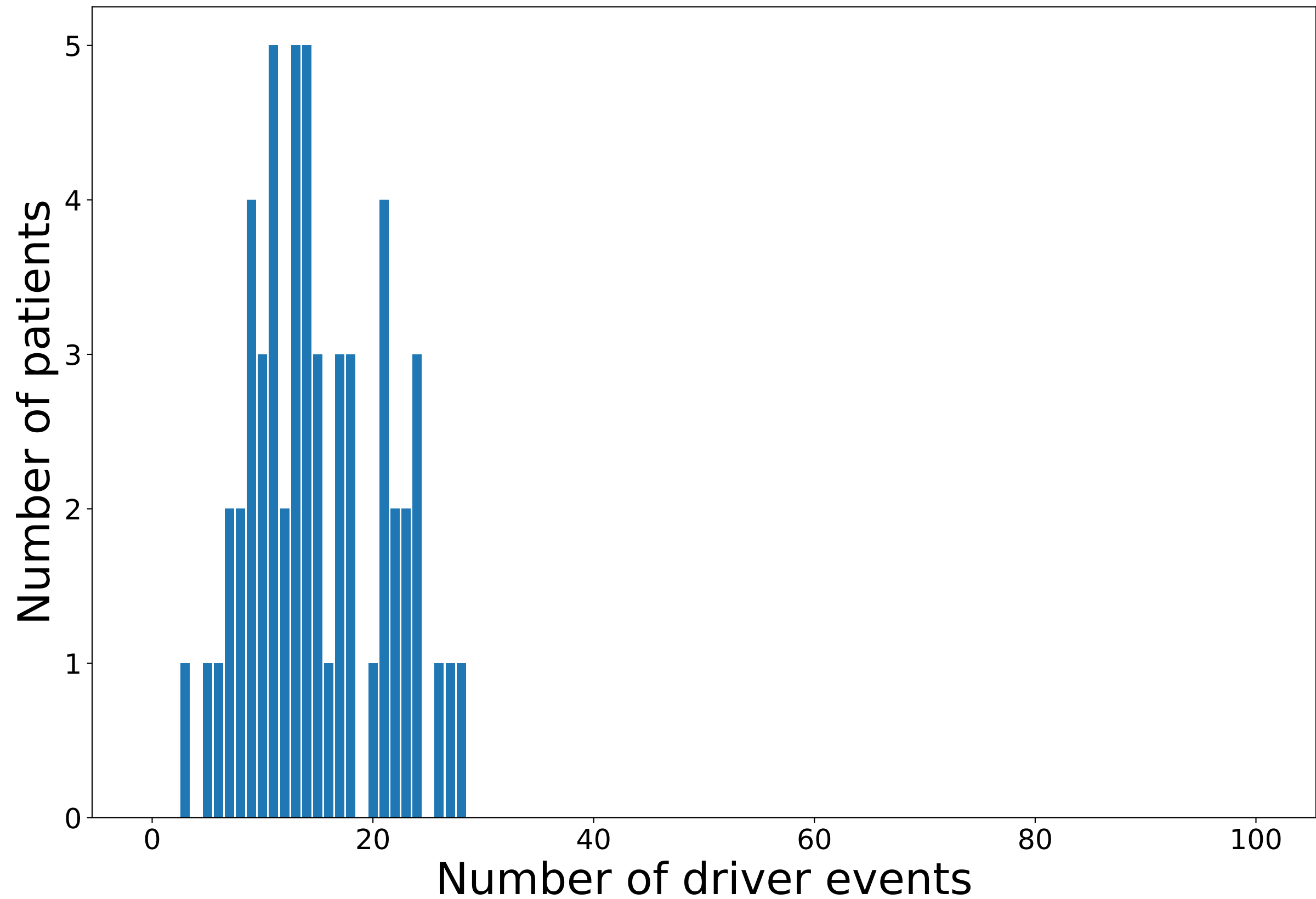

Supplement: S4 Files — (ZIP) [file pgen.1009996.s004.zip › Aneuploidy/COHORTS GISTIC2/patient distributions/2021_11_23_15_0_SKCM_MALE.pdf]

# KIRC\_FEMALE

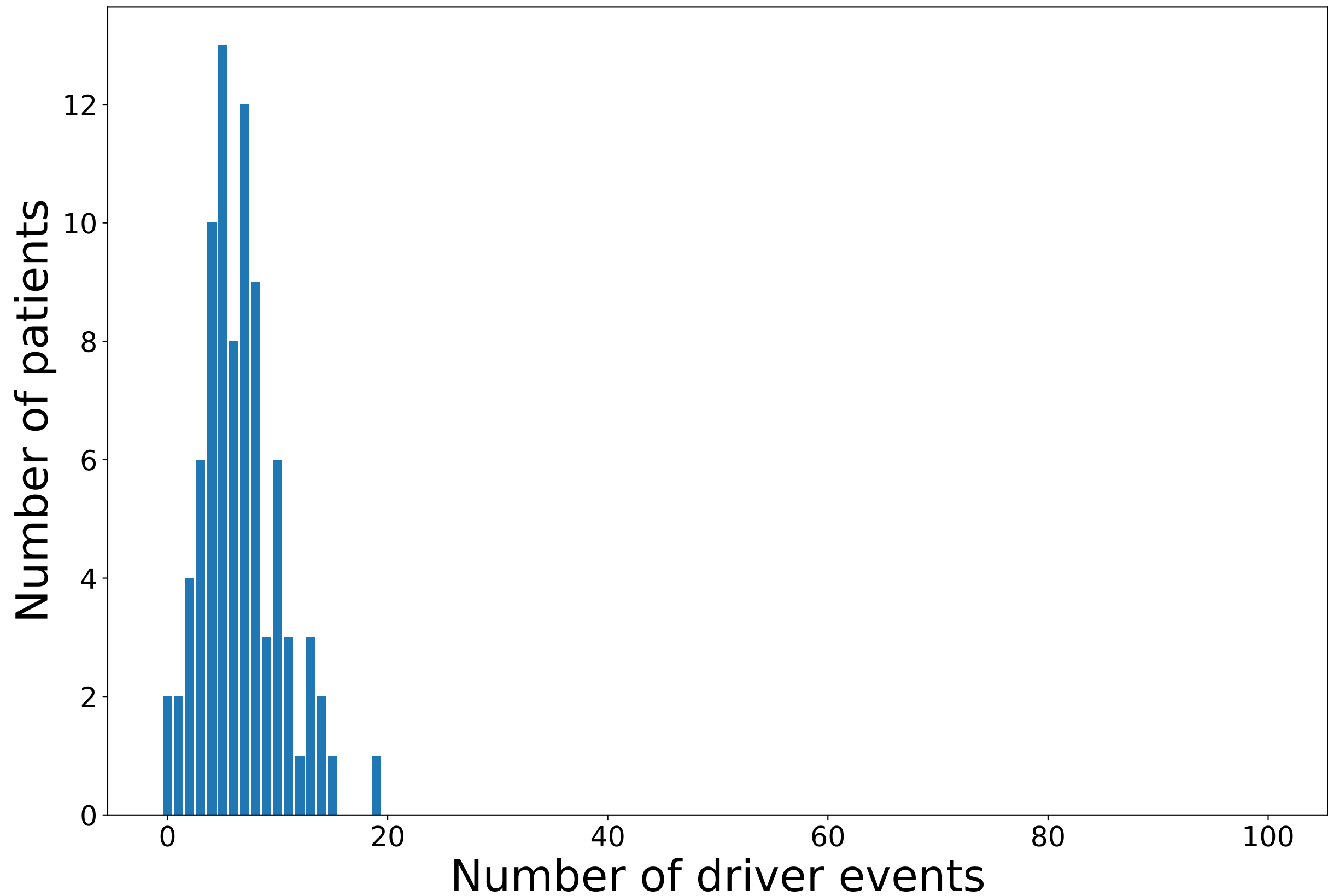

Supplement: S4 Files — (ZIP) [file pgen.1009996.s004.zip › Aneuploidy/COHORTS GISTIC2/patient distributions/2021_11_23_15_0_KIRC_FEMALE.pdf]

# ESCA

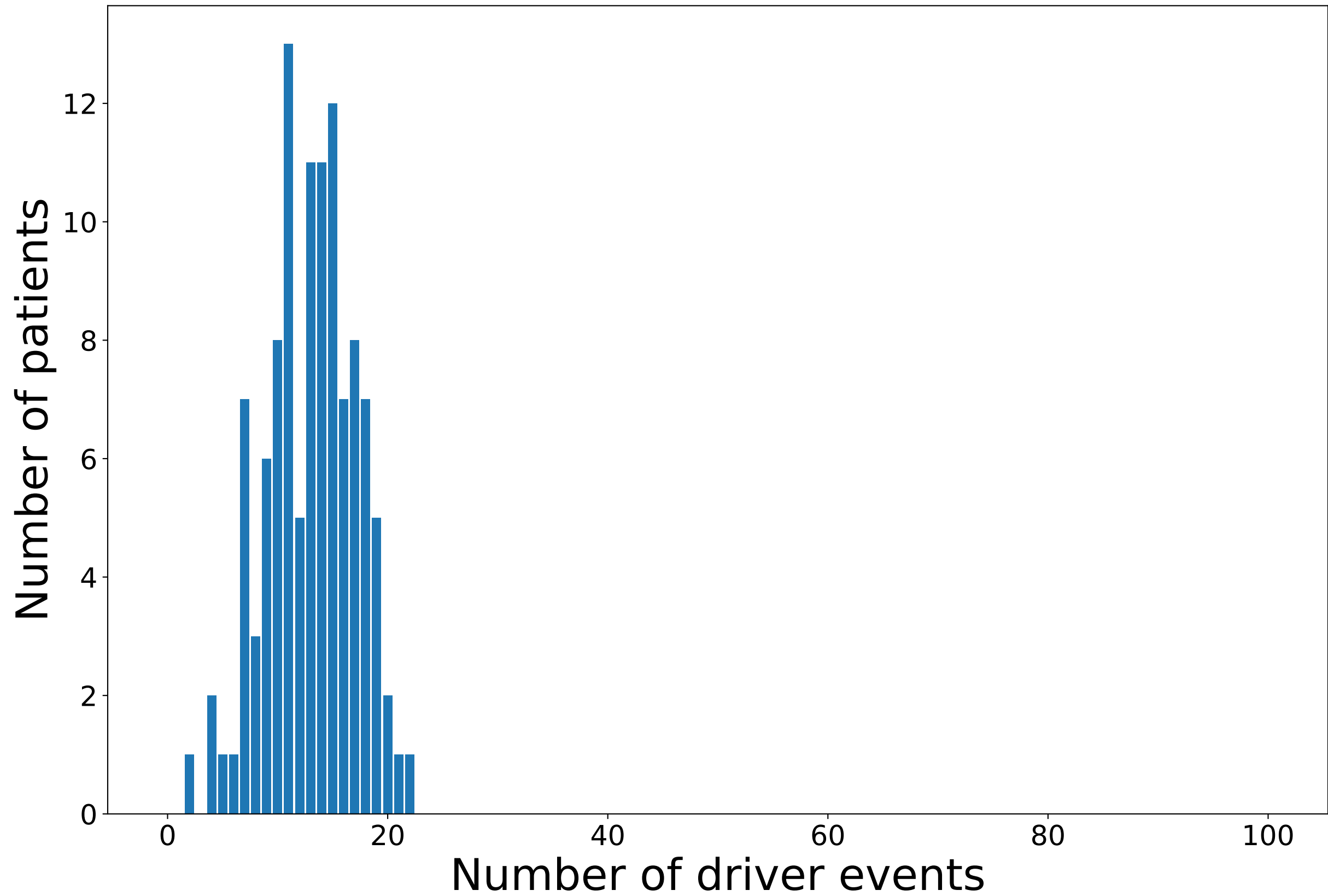

Supplement: S4 Files — (ZIP) [file pgen.1009996.s004.zip › Aneuploidy/COHORTS GISTIC2/patient distributions/2021_11_23_15_0_ESCA.pdf]

# LIHC\_FEMALE

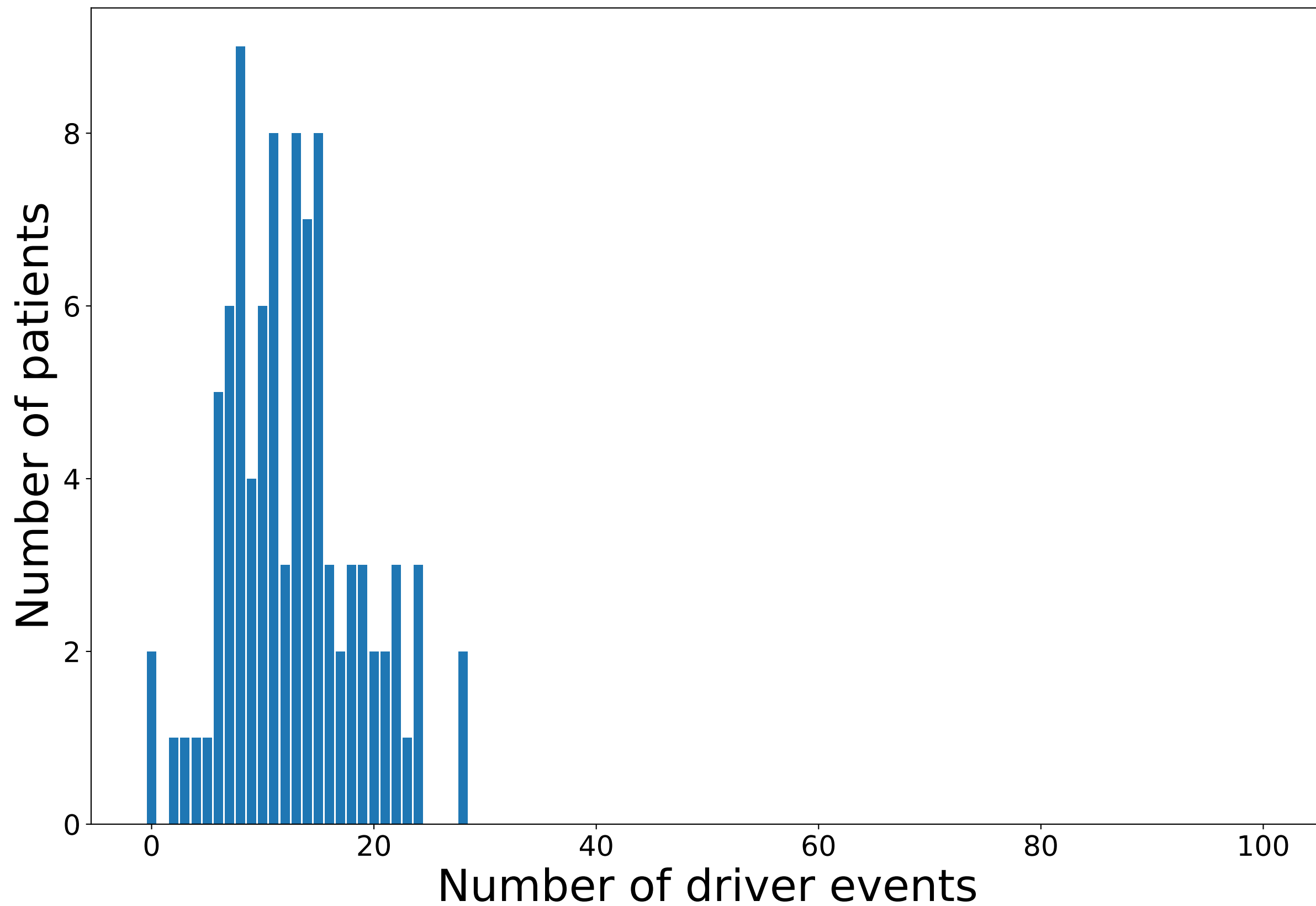

Supplement: S4 Files — (ZIP) [file pgen.1009996.s004.zip › Aneuploidy/COHORTS GISTIC2/patient distributions/2021_11_23_15_0_LIHC_FEMALE.pdf]

# SARC\_MALE

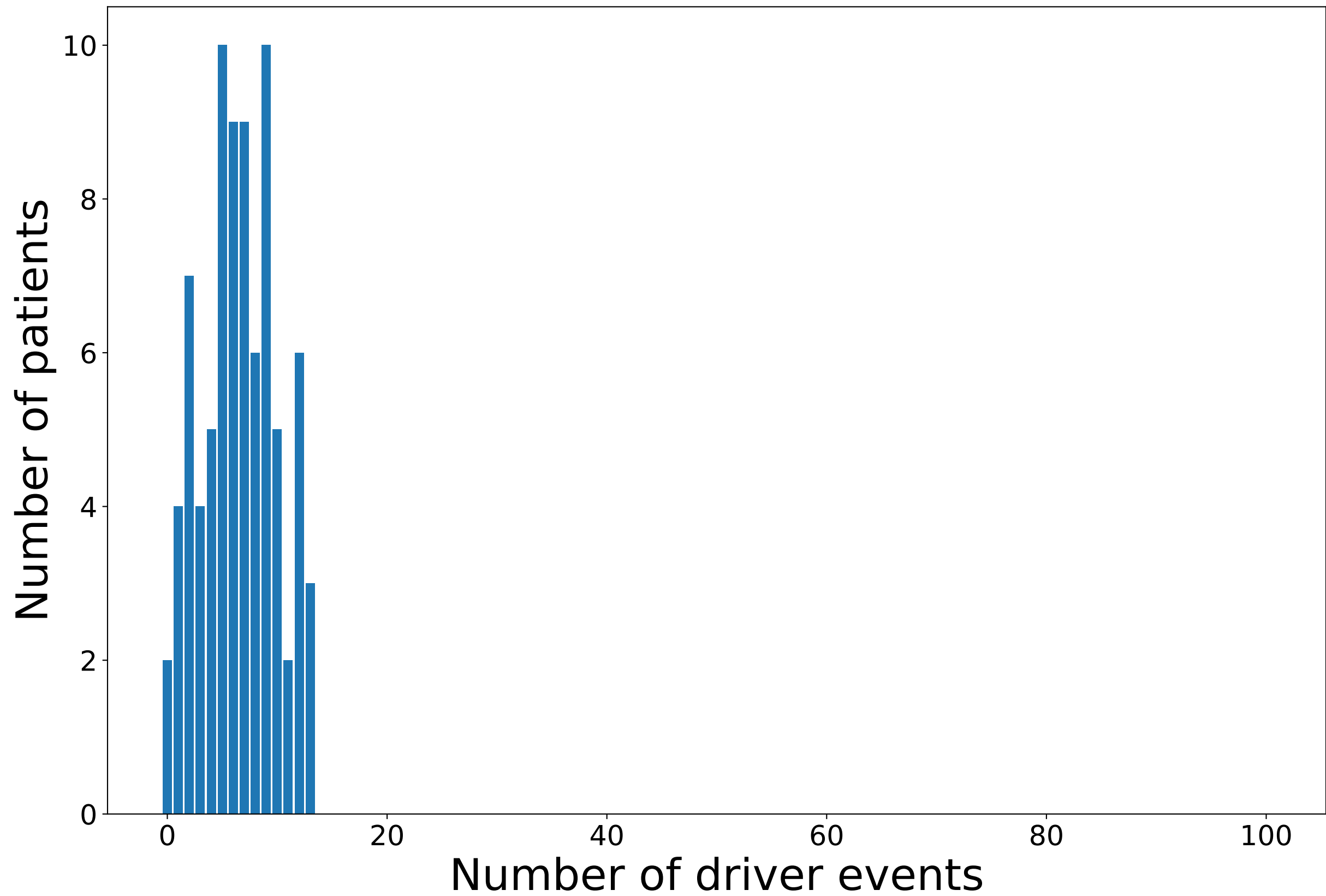

Supplement: S4 Files — (ZIP) [file pgen.1009996.s004.zip › Aneuploidy/COHORTS GISTIC2/patient distributions/2021_11_23_15_0_SARC_MALE.pdf]

# HNSC

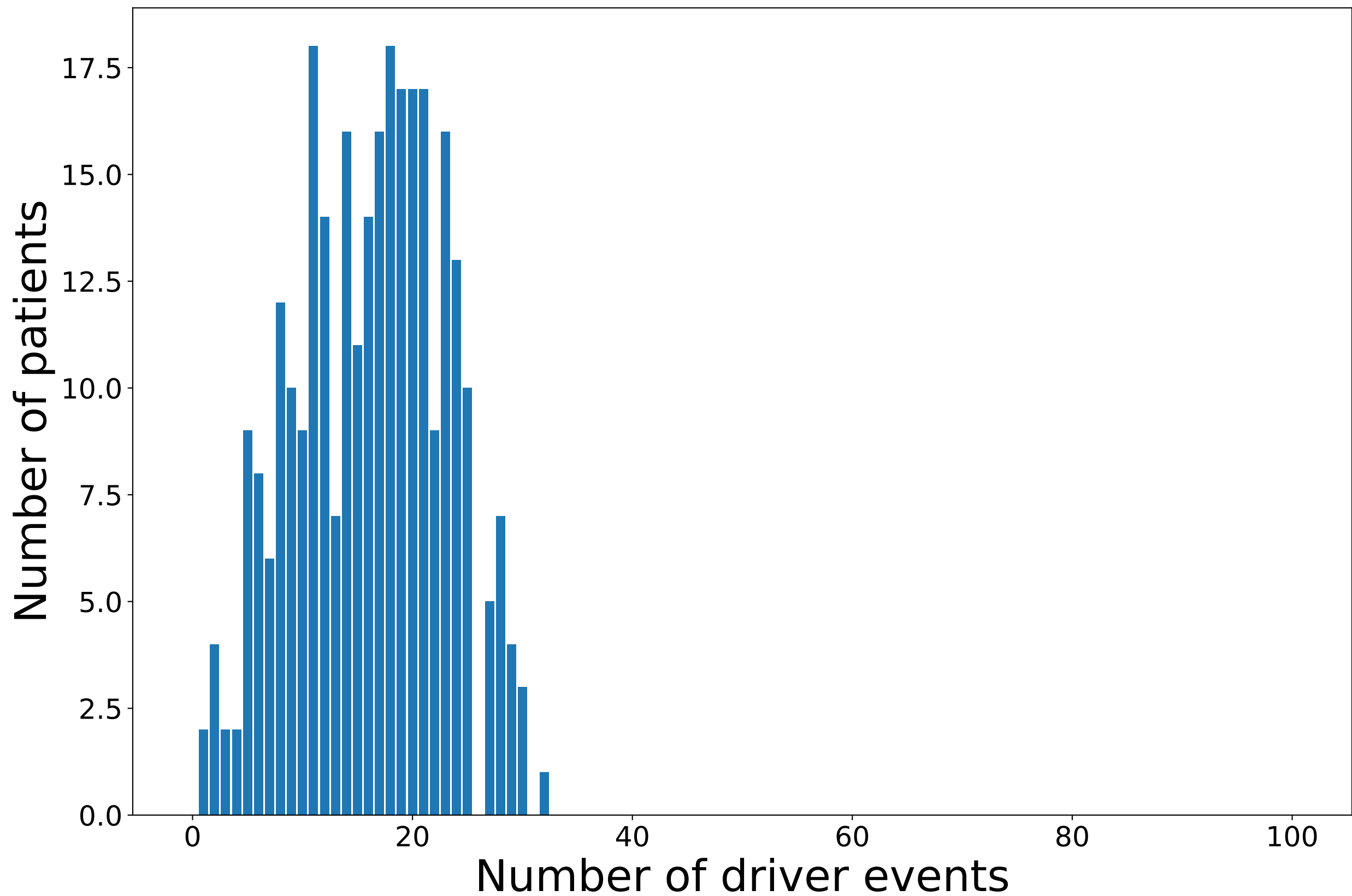

Supplement: S4 Files — (ZIP) [file pgen.1009996.s004.zip › Aneuploidy/COHORTS GISTIC2/patient distributions/2021_11_23_15_0_HNSC.pdf]
