# Supplementary material for: Comprehensive patient-level classification and quantification of driver events in TCGA PanCanAtlas cohorts
Source: PLoS Genet. 2022 Jan 14;18(1):e1009996. doi: 10.1371/journal.pgen.1009996 (PMC8759692; doi:10.1371/journal.pgen.1009996)
Supplement: S3 Files — (ZIP) [file pgen.1009996.s003.zip › COHORTS/cumulative histograms/Distribution_stages_cohorts/2021_11_23_14_20_distribution_stages_males_HNSC.pdf]

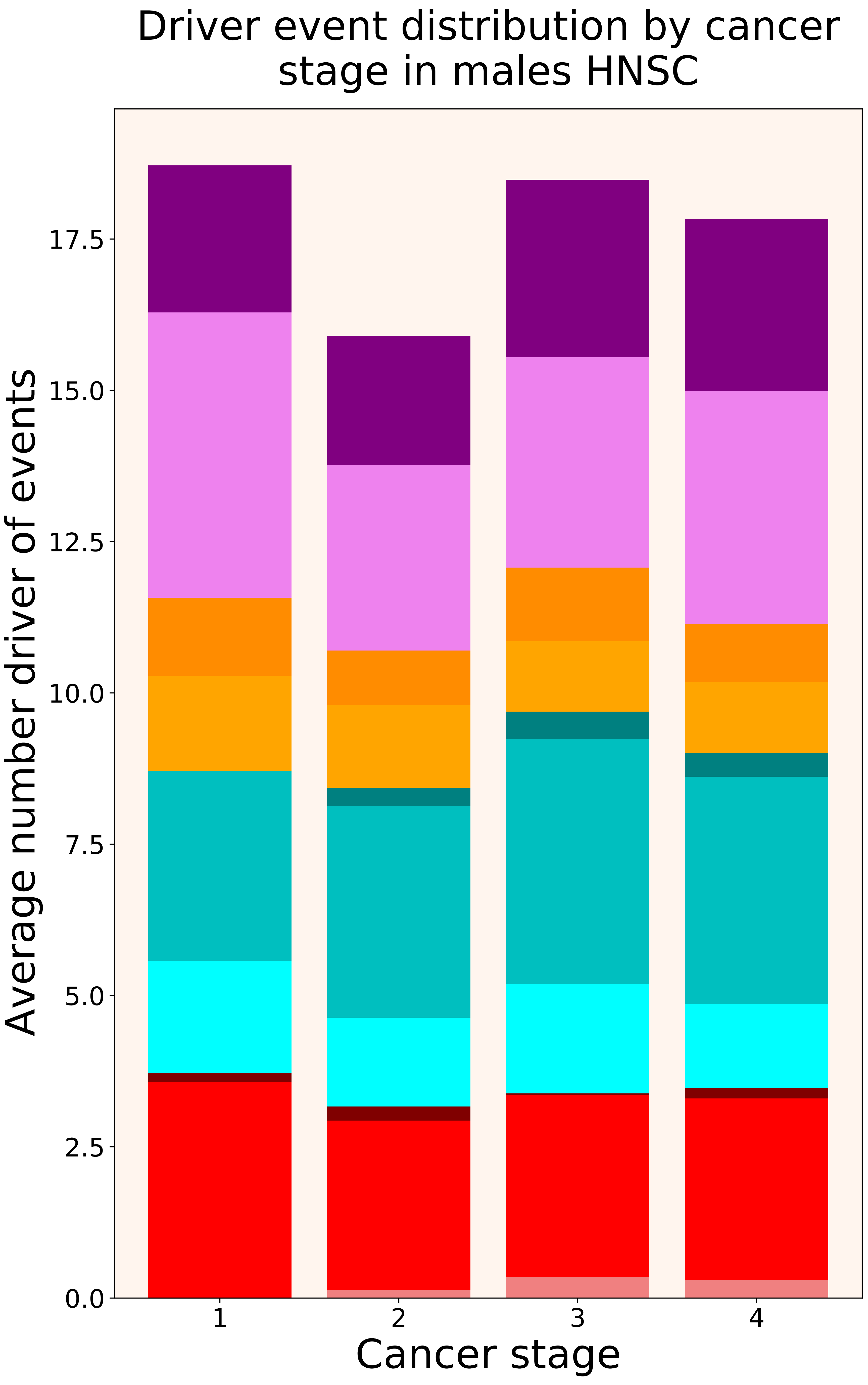

Driver event distribution by cancer stage in males HNSC
Average number driver of events
0.0
2.5
5.0
7.5
10.0
12.5
15.0
17.5
1
2
3
4
Cancer stage
